# 11th European Headache Federation Congress jointly with 31st Congress of the Italian Society for the Study of Headaches: Rome, Italy. 01-03 December 2017

**DOI:** 10.1186/s10194-017-0817-z

**Published:** 2017-11-30

## EHF INVITED SPEAKERS

### S1 Intracranial Hyper-and Hypotension

#### Jan Hoffmann

Headache is one of the most prominent symptoms during a change in intracranial pressure. As in these disorders headache is frequently unspecific, highly variable in its clinical presentation and may occasionally even mimic primary headaches including migraine, diagnosis may in some cases be challenging.

The clinical syndrome of idiopathic intracranial hypertension results from an increase in intracranial pressure without an identifiable cause. Patients suffer from an unspecific headache, which in most cases presents as a daily and bilateral headache without accompanying symptoms. However, an aggravation upon physical exercise, coughing and sneezing as well as nausea and photophobia may occur. In addition to the headache patients commonly suffer from a papilledema that leads to a progressive visual deficit which, if untreated, may results in a complete and irreversible visual loss. In addition patients may suffer from cranial nerve palsies, cognitive deficits, a pulsatile tinnitus and olfactory deficits adding to the significant loss in quality of life. Given the severity and potential irreversibility of these symptoms, a quick and accurate diagnosis as well as an early initiation of treatment is mandatory. Treatment usually consists of a combination of weight reduction and a pharmacological treatment with carbonic anhydrase inhibitors such as acetazolamide and topiramate. Invasive treatments should only be considered in exceptional therapy-resistant cases as long-term data regarding the safety and long-term benefit of these procedures is scarce.

In contrast to a chronic elevation in intracranial pressure which may be primary (idiopathic intracranial hypertension) or secondary, spontaneous intracranial hypotension is in almost all cases secondary to a meningeal rupture with a resulting leak of cerebrospinal fluid. The leaks are commonly localized in the cervicothoracic junction or along the thoracic spine. The clinical picture is dominated by an orthostatic headache which develops in temporal relation to a decrease in intracranial pressure. However, the time course of the orthostatic aggravation may vary substantially and with increasing disease duration may even disappear completely. The pain is thought to result from a slight downward displacement of the brain creating a painful traction of the dura mater. In many cases treatment is not necessary as the leak commonly heals within a few days or weeks causing a complete remission of the symptoms. If the leak persists and treatment becomes necessary an epidural blood patch should be the first step. If a spontaneous remission does not occur and repeated blood or fibrin sealant patches do not lead to a complete remission a surgical intervention may be considered.

### S2 Emerging non CGRP drug targets

#### Messoud Ashina

There is a huge unmet need for new specific acute and preventive drugs in migraine. Development of therapies to treat migraine has previously been hampered by a lack of biomarkers and predictive animal models. This situation has dramatically changed over the last couple of decades, not least as a consequence of the increasing use of a human migraine provocation model that demonstrates the importance of naturally occurring signaling molecules in migraine. New highly specific mechanisms have been discovered and because of this progress, new drug targets are in different stages of clinical development.

### S3 Emergency headaches

#### Luigi Titomanlio

Headache is one of the most common reasons for consultation in the pediatric emergency department (ED). Triage systems have been developed and adapted to the pediatric population to differentiate urgent from nonurgent patients, allowing appropriate and efficient management.In children with certain brain disorders, headache can be associated with focal neurologic signs or symptoms; these children represent a true diagnostic challenge to physicians, owing to the possibility of severe underlying disease. The differential diagnosis in children with headache and focal neurologic signs includes primary etiologies, such as migraine with aura, and secondary etiologies, such as trauma, infection, and vascular, neoplastic, and epileptic disorders. Achieving a diagnosis in children can be challenging at times; important reasons for this include poor description of pain by children and several childhood periodic syndromes that can be common precursors of migraine.

### S4 Hypothalamic Regulation in Headache

#### Arne May (a.may@uke.de)

##### University Clinic of Hamburg, Dept. of Systems Neuroscience

Migraine is a multiphasic disorder and understanding of its pathophysiology starts with the acknowledgment that migraine is not simply a disease of intermittently occurring pain, but that it involves processes that affect the brain over time. If one wants to interpret the most recent findings in migraine pathophysiology it is important to again discuss the clinical presentation of all phases of a migraine attack. There are three clinical features of migraine which point towards the limbic system and hypothalamus as attack generating brain structures. The first one is that almost all symptoms of the premonitory phase including yawning, tiredness and mood changes already point towards hypothalamic involvement. Secondly, the circadian rhythmicity of attacks and thirdly the association of attacks with hormonal status and the menstrual cycle. The hypothalamus has various neuroanatomical connections to pain modulating systems and also to the spinal trigeminal nuclei. The orexinergic system, which is known to regulate arousal and nociceptive processing as well as thermoregulation and autonomic functions, has only recently become a site of interest in migraine research. Another neurotransmitter system involving the hypothalamus is the central dopaminergic system. Recent neuroimaging studies in migraine patients undermine hypothalamic involvement in the premonitory and acute pain phase of migraine. Most recently one migraine patient went into the scanner daily over a whole month which included 3 spontaneous untreated headache attacks. Increased hypothalamic activation was seen in the prodromal phase (within the last 24 h before migraine headache onset) as compared to the interictal state. More importantly, the pain-related hypothalamic functional connectivity between the hypothalamus and the spinal trigeminal nuclei was significantly increased during the preictal phase as compared to the interictal phase. These data strongly suggest that the hypothalamus plays a crucial role in generating premonitory symptoms but also the migraine attack itself. Moreover, using a recently developed protocol for high resolution brainstem imaging of standardized trigeminal nociceptive stimulation, the anterior right hypothalamus (HT) was significantly stronger activated in CM as compared to healthy controls. These data corroborate a crucial role of the HT for migraine chronification but also as for the sustainment of acute migraine pain.

**References**

1. Schulte LH, Sprenger C, May A. Physiological brainstem mechanisms of trigeminal nociception: An fMRI study at 3T. NeuroImage 2015; 124: 518–525.

2. Schulte LH, Allers A, and May A. The hypothalamus as a mediator of chronic migraine: Evidence from high resolution fMRI. *Neurology* 2017 88:2011–2016

3. May A. Understanding migraine as a cycling brain syndrome: reviewing the evidence from functional imaging. Neurol Sci 2017; 38: 125–130

4. Holland PR, Goadsby PJ. Cluster headache, hypothalamus, and orexin. Curr Pain Headache Rep 2009; 13: 147–54

5. Alstadhaug KB. Migraine and the hypothalamus. Cephalalgia 2009; 29: 809–17.

### S5 CGRP CNS models in headache

#### U. Reuter

##### Charité Universitätsmedizin Berlin, Department of Neurology, Charitéplatz 1, 10117 Berlin, Germany

Immunohistological studies show widespread distribution of CGRP within the CNS, but the role and function of this neuropeptides in the brain and spinal cord are largely unknown. There is also increasing interest whether CGRP antagonists penetrate the blood brain barrier and abort migraine headaches in part via central mechanisms. As migraine is a CNS disorder a central abortive or preventative mechanisms is suspected for several years. In this lecture, we will evaluate the information derived from experimental CGRP studies within the CNS. We will analyze the role of CGRP in central sensitization as CGRP most likely facilitates nociceptive transmission. The contribution of CGRP to vasodilation in the CNS will also be discussed, and we look into imaging data of CGRP receptor antagonists in humans. Finally, we will illustrate the contribution of CGRP in an animal model of photophobia.

### S6 The classification of headache disorders has improved over the years

#### Henrik Schytz

The classification of headache disorders has improved over the years, but further work is needed to develop and improve headache diagnosis within headache subtypes. The lecture presents laboratory tests that might be useful in phenotyping and/or diagnosis of long-lasting headache disorders such as migraine, tension-type headache, trigeminal autonomic cephalalgias, trigeminal neuralgia and persisting secondary headaches.

### S7 CGRP PNS models in headache

#### A. Maassen van den Brink

##### Div. of Pharmacology, Dept. of Internal Medicine, Erasmus MC Rotterdam, The Netherlands

Calcitonin gene-related peptide (CGRP) is considered to be one of the main molecules in the pathophysiology of migraine. Currently, several drugs that target either the CGRP peptide or its receptor are in clinical studies for the prophylactic as well as the acute treatment of migraine. While CGRP is expressed abundantly in the central nervous system, it also plays an important role in the peripheral nervous system. Most antimigraine drugs that are currently in clinical development and target CGRP or its receptor (for example, the monoclonal antibodies) are not able to cross the blood brain barrier and thus do not reach the central nervous system, highlighting the importance of CGRP and its receptors at sites not protected by the blood brain barrier. These sites include the trigeminal ganglion, but also perivascular sensory afferents that may be involved in the pathophysiology of migraine as well as in the development of potential side effects. During the lecture, models and mechanisms important for the understanding of the role of CGRP in the peripheral nervous system will be discussed.

### S8 Understanding the non-pain phases of migraine: premonitory and postdromal

#### Peter J Goadsby

##### NIHR-Wellcome Trust King’s Clinical Research Facility, King’s College London, UK

Migraine is the most common cause of neurological disability worldwide [1]; it is a disorder of the brain with pan-sensory dysfunction [2]. Migraine has, in essence, three phases, prior to the canonical attack- the premonitory or prodromal phase, the attack itself, headache with or without aura, and the period after canonical attack, the postdrome.

The premonitory phase can occur from hours to days before the canonical attack. The symptoms include: neck discomfort, yawning, tiredness, concentration impairment, mood change, polyuria/polydipsia, or food cravings [3]. The symptoms can be seen in children, as they are in adults [4]. Moreover, there is evidence from functional imaging of activation in the region of the hypothalamus during the premonitory phase [5].

The postdrome phase occurs after the headache phase of the canonical attack is settling; it is typically settled in about half of patients in six hours. The most common symptoms are: feeling tired/weary, concentration impairment and neck discomfort [6]. Remarkably there is widespread reduction in brain blood flow in the postdrome [7], which reflects the phenotype well.

Understanding the non-pain phases of migraine will lead to be a better formulation of the pathophysiology of migraine and eventually to better treatment.

**References**

1. Disease GBD, Injury I, Prevalence C. Global, regional, and national incidence, prevalence, and years lived with disability for 310 diseases and injuries, 1990-2015: a systematic analysis for the Global Burden of Disease Study 2015. Lancet. 2016;388(10053):1545-602.

2. Goadsby PJ, Holland PR, Martins-Oliveira M, Hoffmann J, Schankin C, Akerman S. Pathophysiology of Migraine- A disorder of sensory processing. Physiological Reviews. 2017;97:553-622.

3. Giffin NJ, Ruggiero L, Lipton RB, Silberstein S, Tvedskov JF, Olesen J, et al. Premonitory symptoms in migraine: an electronic diary study. Neurology. 2003;60:935-40.

4. Karsan N, Prabakhar P, Goadsby PJ. Premonitory symptoms of migraine in childhood and adolescence. Current Pain and Headache Reports. 2017;21:34.

5. Maniyar FH, Sprenger T, Monteith T, Schankin C, Goadsby PJ. Brain activations in the premonitory phase of nitroglycerin triggered migraine attacks. Brain. 2014;137:232-42.

6. Giffin NJ, Lipton RB, Silberstein SD, Olesen J, Goadsby PJ. The migraine postdrome. An electronic diary study. Neurology (Minneap). 2016;87:1-5.

7. Bose P, Karsan N, O'Daly O, Zelaya F, Goadsby PJ. ALTERATIONS IN CEREBRAL BLOOD FLOW DURING THE POSTDROME PHASE OF A MIGRAINE ATTACK CAPTURED WITH ARTERIAL SPIN LABELLED (ASL) MRI. Cephalalgia. 2017;37:in press.

### S9 The Eurolight Project 2017

#### Christian Lampl

##### Headache Medical Center, Seilerstätte, Ordensklinikum Linz Barmherzige Schwestern, Austria

The Eurolight project, supported by the EC European Agency for Health and Consumers of the European Commission, was a data-gathering exercise undertaken primarily to inform health policy on headache disorders in the European Union (EU). This very large and complex study involved multiple collaborating partners (academic and lay) in ten countries, representing 60% of the adult population of the EU. The project took the form of surveys by structured questionnaire, conducted from November 2008 to August 2009. Questionnaires were analysed from 8,271 participants (58% female, mean age 43.4 y). Unadjusted lifetime prevalence of any headache was 91.3%. Gender-adjusted 1-year prevalences were 35.3% for migraine, 38.2% for TTH and 7.2% for headache on ≥ 15 d/mo. Personal impact was high, and included ictal symptom burden, interictal burden, cumulative burden and impact on others (partners and children). Mean per-person annual costs were €1,222 for migraine, €303 for tension-type headache. We confirmed that depression and especially anxiety are comorbid more than by chance with migraine. The level of this impact and its pervasiveness taken together with estimates of huge financial cost, have important implications for health policy in Europe. Eurolight 2017 should proceed with focusing on cluster headache and headache in the elderly.

### S10 Multidisciplinary approach to head pain

#### Rigmor Hoejland Jensen

##### Danish Headache Center, Rigshospitalet-Glostrup, University of Copenhagen, Denmark

***Background*** *:* Despite the very high prevalence of headaches, multidisciplinary headache clinics are still few and better documentation of their content and efficacy is needed.

***Objective*** *:* To describe the structure of a multidisciplinary approach and to characterize the patients and treatment results from existing centres. Further to describe the proposed organization of headache care in Europe.

The collaboration between European Headache Federation and Lifting The Burden has proposed a three-tiered structure for Headache Care in Europe. It is organised with the majority of patients (90%) treated in primary care, the more complex migraine and tension-type headache patients (7-8%) in secondary care with a headache specialist and a nurse whereas the most complex patients i.e. medication overuse headache, comorbidity, chronicity or rare headache disorders(2-3%) should be referred to a tertiary headache centre. At this level headache specialists and a multidisciplinary team should conduct more complex treatment, initiate research and education. The composition of the multidisciplinary team may vary, however and here there is no international consensus. Most centres include nurses, psychologists and in some countries also sports-therapists or physiotherapists.

A systematic review of 1300 patients from the tertiary Danish Headache Centre revealed that patients had a mean age of 43.7 years and the male/female ratio was 3/7. In total, headache frequency was reduced from 20 to 11 days (p<0.001) and the absence rate 5 to 2 days/month (p<000.1) after treatment. Predictors for good outcome was female gender, migraine, triptan overuse and a mean headache frequency of 10 days/month. In recent years more evidence from other centres has been provided and the positive outcome was confirmed, also in so called refractory patients.

***Conclusion*** *:* Treatments strategies to the complicated headache patients need individualization but the present evidence provide hope for the patients and a strong support for a multidisciplinary approach in a tertiary headache centre. The existing treatment strategies will be presented. Further discussion and evaluation of the elements and the outcome predictors are important for future planning.

### S11 GWAS studies in migraine

#### Arn M.J.M. van den Maagdenberg

##### Departments of Human Genetics & Neurology, Leiden University Medical Center, Leiden, The Netherlands

Migraine is a common debilitating brain disorder characterized by severe headache attacks with various associated neurological symptoms. About one-third of migraine patients experience an aura preceding the headache phase: hence migraine with and without aura. Many migraine patients also suffer from comorbid neurological disorders, such as epilepsy, depression and stroke. Migraine is a genetic disease with both environmental and genetic factors determining the susceptibility to attacks. Recent technological advances in genetic analysis, which allowed simultaneous testing of hundreds of thousands of single nucleotide polymorphisms (SNPs) in tens of thousands of migraine patients in genome-wide association studies (GWAS), made it feasible to identify robust gene variants for the common forms of migraine. Whereas GWAS performed in various migraine subtypes yielded different top hits for the different subtypes, additional analyses seem to point to a shared genetic underpinning in migraine. Identified gene variants point towards various molecular pathways, e.g. neuronal dysfunction, vascular integrity and function, and pain signaling. GWAS data sets, to some extent, can also been used to identify the type of brain cell involved in pathology. GWAS also enable the identification of (shared) genetic factors for diseases comorbid with migraine. Unlike gene mutations in monogenic migraine subtypes, the effect size of gene variants in common migraine is small, thus complicating direct translation to diagnostic tests, pathogenetic mechanisms, and treatment targets. In fact, strategies to properly address the biological role of these variants are still being developed. Further technological advances in genetic research, commonly labelled by “next generation sequencing” (NGS), make it feasible to identify gene variants/mutations at the DNA level at an unprecedented scale. The coming years will show the true impact of these combined genetic approaches on the identification of genes, pathological mechanisms, and diagnosis of patients in migraine.

### S12 Diagnostic tests for assessing patients with neuropathic pain

#### A Truini

##### Department of Neurology and Psychiatry, University Sapienza, Rome, Italy

Research has devised various techniques for investigating nociceptive and non-nociceptive somatosensory pathways in patients with neuropathic pain. The most widely agreed tools in use today include neurophysiological techniques and skin biopsy.

The standard neurophysiological techniques such as nerve conduction studies, trigeminal reflexes and somatosensory evoked potentials are mediated by large non-nociceptive afferent fibres (Aβ-fibres), and are widely used for assessing peripheral and central nervous system diseases.

Laser Evoked Potentials (LEPs) are the easiest and most reliable neurophysiological technique for assessing nociceptive pathway function. Laser-generated radiant heat pulses selectively excite free nerve endings in the superficial skin layers and activate Aδ and C nociceptors and evoke scalp potentials. In diseases associated with nociceptive-pathway damage, LEPs can be absent, reduced in amplitude or delayed in latency.

Skin biopsy is a reliable and minimally invasive tool for investigation of nociceptive fibres in human epidermis and dermis. Researchers have used this technique for assessing epidermal nerve fibres qualitatively and quantitatively. Skin biopsy can be done at any site of the body, with a disposable punch, using a sterile technique, and under local anaesthesia. Many investigators have used skin biopsy to investigate epidermal nerve fibres in various peripheral nerve diseases, such as diabetic neuropathy, infectious and inflammatory neuropathies and neuropathies associated with systemic diseases. In all studies, epidermal nerve fibre density was significantly lower in patients with neuropathy than in controls.

### S13 Neuromodulation and Headache. Future perspectives

#### Massimo Leone (Massimo.Leone@istituto-besta.it)

##### Neuroalgology Department, Fondazione I.R.C.C.S. Istituto Neurologico Carlo Besta, Milan, 20133, Italy

Patients suffering from chronic headaches challange health care systems. They are estimated to affect 3% of general population and carry a considerable disease burden. A proportion of chronic headache patients does not properly respond to prophylactic treatments or shows low tolerability profile and remains in need for alternative therapeutic strategies and options.

The improved understanding of head pain pathophysiology has focused attention on the role of neural structures both at peripheral and central nervous system level. Thus in the attempt to improve chronic intractable neurovascular headache (migraine and cluster headache) patients a number of neuromodulation procedures targeting peripheral and central nervous system structures have been tried.

So far, efficacy and safety of various non-invasive and invasive stimulation procedures and devices have been investigated. Vagus nerve stimulation, supraorbital stimulation and single-pulse transcranial magnetic stimulation are considered non invasive neurostimulation options. While invasive procedures are occipital nerve stimulation, sphenopalatine ganglion stimulation and hypothalamic deep brain stimulation. Years after their introduction there is still debate about their use and place in clinical practice.

Results from open label series and few controlled trials suggest the need of further investigations.

Criteria employed to define intractable headaches were given more than ten years ago (1). An ad hoc European Headache Federation expert board has reviewed these aspects (2). A still unsolved issue is the lack of adequate placebo to properly design randomized controlled trials in neurostimulation studies. In patients with chronic pain conditions interpretation of placebo effect is a challange particularly for headache specialists.In chronic migraine and chronic cluster headache patients occurrence of psychiatric comorbidities is frequently encountered. Also, occurrence of medication overuse headache – seen as an addiction behavior - is frequently observed both in chronic migraine and chronic cluster headache. The role of psychosocial factors driving drug overuse/addiction behavior in chronic headaches is undisputable. These factors are often a barrier when selecting patients for neurostimulation procedures.

Long term experience with deep brain stimulation of the posterior hypothalamic area in chronic cluster headache has suggested that the generator of the attacks is not there (3). Similarly other neurostimulation procedures tried in migraine and cluster headache have shown poor, unsatisfactory ability to stop ongoing attacks. These observations suggest either that these stimulation procedures are not able to switch off the attack generator or that there are multiple migraine/cluster pain generators.

**References**

1. Goadsby PJ, Schoenen J, Ferrari MD, Silberstein SD, Dodick D. Towards a definition of intractable headache for use in clinical practice and trials. Cephalalgia 2006; 26:1168–70

2. Martelletti P, Jensen RH, Antal A, Arcioni R, Brighina F, de Tommaso M, Franzini A, Fontaine D, Heiland M, Jürgens TP, Leone M, Magis D, Paemeleire K, Palmisani S, Paulus W, May A. Neuromodulation of chronic headaches: position statement from the European Headache Federation. J Headache Pain. 2013 Oct 21;14(1):86.

3. Leone M, Franzini A, Proietti Cecchini A, Bussone G. Success, failure and putative mechanisms in hypothalamic stimulation for drug resistant chronic cluster headache. Pain 2013; 154 (1): 89-94

### S14 What we should in the future

#### T.J. Nurmikko

##### The Walton Centre NHS Foundation trust

An underlying concept in the new ICHD-3 classification of trigeminal neuralgia is the postulation that clinical presentations matter because they reflect distinct pathophysiological mechanisms. Previous attempts to establish the connection between the two have yielded uncertain results as the authors have paid limited attention to individual clinical symptoms and signs. Yet, the relatively strict criteria for trigeminal neuralgia and its subgroups yield homogenous populations that allow advantage to be taken of the advances in neurophysiological and imaging methods. It is now possible to conduct subgroup-specific pathophysiological studies aimed at biomarkers that pave the way for precision diagnosis of TN and individualised therapy. An example of how this might be done comes from recent studies based on sensory profiling of peripheral neuropathic pain. In a large group of patients with three different diagnoses, cluster analysis of detailed sensory testing revealed three main sensory phenotypes [1], with the potential to allocate individual patients to these sensory groups [2]. For TN, a stratification based on the new classification and linked to patients’ symptoms, somatosensory profiles, and neurophysiological and neuroimaging data provides a unique opportunity to explore clinical questions that are even more ambitious than those for other neuropathic pains. In my presentation I will suggest a pathway as to how to accomplish this. I will start by arguing that the existing data are sufficient to recommend preferred treatment in selected cases. I will then highlight a number of clinically relevant research questions that can be answered by large-population multi-centre studies applying established methods ranging from QST and evoked potentials to structural and functional neuroimaging of the trigeminal system and linking them with clinical signs and symptoms. Alongside this, I will discuss the challenges of phenotype profiling that could guide pharmacotherapy with, e.g., Na_v_ 1.7 channel blockers or identifying genes that could make a subject susceptible to the development of TN.

**References**

1. Baron R, Maier C, Attal N, et al. Peripheral neuropathic pain: a mechanism-related organizing principle based on sensory profiles. Pain 2017;158:261-272.

2. Vollert J, Maier C, Attal N, et al. Stratifying patients with peripheral neuropathic pain based on sensory profiles: algorithm and sample size recommendations. Pain 2017158;14461455.

### S15 Posttraumatic headache in children and adolescents

#### Ishaq Abu-Arafeh

##### Consultant in paediatric Neurology, Royal Hospital for Children, Glasgow, UK

Headache is a common problem in children and adolescents with a prevalence of about 60%. Head injuries are also relatively common with an estimated incidence of 3/1000 children per year with 80-90% of cases are considered as minor injuries (Glasgow Coma Score 13-15). Mild head injury is associated with good recovery in most patients, but with a small risk of poor outcomes. Headache is the most common complication that occurs as an isolated symptom or can be a part of the post-concussion syndrome which can also include dizziness, fatigue, reduced ability to concentrate, psychomotor slowing, mild memory problems, insomnia, anxiety, personality changes and irritability Following head injuries, children may develop headache for the first time or have their previously experienced headache getting worse in severity or frequency. Post head injury headache is referred to as acute posttraumatic headache if it evolves within one week of the injury and resolves within 3 months and it is called chronic posttraumatic headache (CPTH) if it persisted for over 3 months.

Systematic review of the occurrence of headache after head injury shows that up to 40% of children complain of any type of headache following head injury and around 7% have CPTH as defined by the ICHD-2 and 3beta.

The pathophysiology of posttraumatic headache is not well understood, but likely to involve several mechanisms and factors. It is suggested that even minor head injury may cause a widespread stretching or shearing injuries to the axonal network. Psychosocial factors may also play a role in the pathogenesis of CPTH.

The clinical features of CPTH are similar to primary headache disorders phenotypes with the majority of children presenting with migraine-like headache and probable tension-type headache. Some children may have mixed or unclassified headache disorders. In the majority of children no investigations are necessary. However, neuroimaging and other investigations may be necessary in children with red flags or abnormal findings on neurological examination.

The management of children with CPTH should include reassurances, adequate pain relief and preventative treatment as appropriate. Multidisciplinary approach is necessary and should include support from clinical psychology and education to help the child achieve normal school attendance and education.

The prognosis of CPTH is generally good, but long term data are needed.

### S16 Contraception in Women with Seizure Disorder

#### György Bártfai

##### Department of Gynaecology and Obstetrics, University of Szeged

One third of women with epilepsy (WWE) are in reproductive age, and nearly 50% of their pregnancy are unplanned because of using an inappropriate method or failure of combined oral hormonal contraceptives (COCs). The interaction between enzyme inductive antiepileptics (EiAED) like carbamazepine, phenytoin, primidone, phenobarbitone, rufinamide, lamotrigine, topiramate and COCs is well-known. Therefore, while taking this medication, the risk of contraceptive failure is quite high.

The mechanism of action of enzyme-inductors is to modify the metabolism of the sexual steroids in the liver. Moreover, ethinylestradiol (EE) might modify the metabolism of certain antiepileptic drugs (glucuronization of lamotrigine). Therefore, the gynaecologist has to be careful when prescribing the pill or administering other types of hormonal contraceptives for WWE.

Knowing the interaction between antiepileptics and contraceptives is important to find the most effective medication with fewer side effects. The consequence of interaction between EiAED and COC as well as EE and AED (lamotrigine) may be: a) unwanted pregnancy; b) teratogenicity; negative effect on the cognitive and psychomotor functions of the child; and/or c) changes in seizure activity.

Nowadays, women with epilepsy do not always get the right information; thus, it is necessary to improve the cooperation and consultation between the epileptologist and the gynaecologist. The first meeting with the epileptologist or gynaecologist is equally important in choosing the right antiepileptic drugs and/or contraceptive method. The information is also needed even if the patient is sexually inactive.

### S17 CSD evolution in 2017

#### H. Bolay

Migraine is a complex neuronal disorder where the cortex has a key importance and characteristic headache attack is associated with multiple sensorial disturbances. A cerebral cortical phenomenon known as cortical spreading depression (CSD) was linked to lateralized headache. CSD is an intrinsic brain phenomenon to a noxious stimulus such as high potassium or trauma, and manifests as an extreme excitability state of the gray matter with massive depolarization of neuronal and glial membranes and redistribution of ions. Initial depolarization is replaced by a long-term depression in the neuronal activity which traverses whole hemisphere in case of lissencephalic brain with a rate at 3–6 mm/min. Propagating depolarization in the brain parenchyma leads to a release of various vasoactive and nociceptive ions and molecules. Vascular compartment reacts with initial hyperemia followed by long-term oligemia. It occurs in many species from rodents to primates, though it is hard to initiate and sustain its propagation in gyrencephalic brains. Spreading depression wave involves neuronal, glial and vascular cells, and leads remarkable effects on those compartments and overlying meningeal membranes with capability of triggering peripheral trigeminal fibers and second order trigeminal neurons in the brainstem nucleus, though its effect on subcortical structures are less known. CSD is implicated in the development of inflammatory response and releasing CGRP and nitric oxide from trigeminal nerve endings.

Animal studies investigating the mechanisms of migraine and CSD are usually conducted under anesthesia, despite the fact that pain is a conscious experience. Anesthesia have profound effects on the mechanisms by which CSD is initiated and propagated, and clearly prevents observation of any associated behavioral response. Therefore CSD studies in awake animals are crucial for translational migraine research. CSD in freely moving lissencephalic animals, led to reduced locomotor activity, freezing & grooming episodes and pain calls (Akcali et al, 2010).

Cerebral cortex and thalamus are inseparable in sensory processing and thalamic reticular nucleus (TRN) is the gatekeeper of sensory outflow to the cortex. CSD was shown to activate thalamic reticular nucleus (TRN) only in awake animals (Tepe et al, 2015). Electrocorticographic recordings demonstrated the direct propagation of CSD waves in to thalamic reticular nucleus. Activation of TRN was unilateral and ipsilateral to CSD and TNC. It was dependent on full conscious experience and highly vulnerable to anesthetics. CSD selectively activated visual sector of TRN, though other six TRN sectors of auditory, gustatory, visceral, somatosensoriyal, motor and limbic TRN were not affected by CSD. CGRP receptor antagonist MK-8825, reversed CSD induced freezing, grooming, wet dog shake behavior, reductions in von Frey thresholds and c-fos induction in TNC and TRN. However, MK-8825 did not block CSD waves and accompanied rCBF response (Filiz et al, 2017). MK-8825 did not exert any effect on CSD induced amygdala activation and anxiety behavior.

TRN is also involved in discrimination of sensory stimulus and transient disruption of sensorial perception during migraine headache attacks was reported (Boran et al, 2016). Disruption of temporal discrimination of two consecutive sensorial stimuli seems specific to migraine headache attacks (Vurallı et al, 2016, Vurallı et al, 2017).

Involvement of a strategic subcortical thalamic structure by a cortical event is important to explain several clinical features of migraine such as 1) Dysfunction of the GABAergic neurons in TRN would result in enhanced transmission of sensory information to the cortex and disruption of sensory discrimination 2) Photophobia and visual hallucinations of aura may reflect dysregulation of visual stimuli by the TRN, 3) TRN could play a role in either termination or initiation of an attack as sleep is closely related with migraine, attacks are often associated with the circadian cycle and are typically relieved by sleep, 4) Thalamo-cortical gating could be a novel target in migraine as valproate, triptans and CGRP antagonists MK-8825 suppressed CSD induced TRN activation.

### S18 Trigeminal Neuralgia and other facial pains

#### R. Benoliel

In this discussion, we will review the differential diagnosis of Trigeminal Neuralgia (TN) vis-à-vis other facial pains that may mimic TN’s features. Common misdiagnoses for TN include dental pathology, other regional neuralgias, short-lasting neuralgiform headaches with autonomic signs (SUNHA), cluster headache and theoretically an atypical (shorter) cluster-tic syndrome (CTS). More rarely there may be more sinister underlying disorders (tumors, multiple sclerosis) that induce TN-like syndromes. We will outline and highlight the salient features across disorders that will ensure correct diagnosis.

### S19 The concept of trigeminal neuralgia

#### Giorgio Cruccu

Trigeminal neuralgia (TN) is a neurological disease which is peculiar under several respects. The diagnosis of TN, in its typical presentation, in unmistakable on clinical grounds alone. Pain manifests with intense bursts that occur and end abruptly and usually last few seconds only. This type of pain is paradigmatic of what pain scholars call paroxysmal pain. The most common verbal descriptors are electric-shock like or stabbing. Unique to TN is the trigger mechanism. The attacks are evoked by innocuous stimuli in tiny zones of the extra- or intraoral trigeminal territories. The most frequent trigger maneuvers include activities of the daily life such as washing, cleaning, brushing the teeth or talking. Although the trigger zones shared by most patients are confined between the nostril and the lateral perioral region, any area innervated by the trigeminal nerve may do.

One aspect of pathophysiology is supported by established neurophysiologic, neuroimaging, and histologic evidence: the primary mechanism is focal demyelination of primary afferents near the entry (extra- or intra-axial) of the trigeminal root into the pons. A second pathophysiologic theory, admittedly more debatable, is that hyperexcitable primary afferents, in the area of focal demyelination, become a source of ectopic generation of impulses and ephaptic transmission (cross talk) from close, healthy nerve fibers. More supported by evidence from animal models is the generation of high-frequency discharges. A third potential step, with so far almost no sound evidence at all, is that the hyperactivity of primary afferents secondarily induces central sensitization of wide dynamic range neurons in the spinal trigeminal nucleus or even more central changes.

Finally, TN is unique also for its pharmacological and surgical treatment. TN is highly sensitive to voltage-gated, frequency-dependent sodium-channels blockers (and almost nothing else), and is the neuropathic pain condition that respond best to surgical lesions of the postganglionic primary sensory afferents.

### S20 The HUNT Studies

#### Mattias Linde

The Nord-Trøndelag Health study (HUNT), ongoing in Norway since 1984, is one of the worlds’ largest longitudinal epidemiological studies collecting comprehensive data on headache disorders. The speaker will present an overview of the methodological potentials and challenges of the HUNT survey. Results will be displayed regarding prevalences of the common headache disorders and their trends over time.

Most importantly, the HUNT-survey enables risk factor analyses. Findings will be reviewed for factors of life such as physical activity, substance use, head traumas, insomnia, and mortality. Also, associations between headache and women’s issues such as contraceptives, hormone replacement, pregnancy, and menarche have been studied and will be discussed. Finally, associations between intracranial abnormalities and headache disorders are now beginning to be published from a neuroimaging sub-study (HUNT MRI).

### S21 CSD in primary and secondary headaches

#### Cenk Ayata

Spreading depression (SD) is a wave of simultaneous and near-complete depolarization of virtually all cells in brain tissue associated with a transient “depression” of all spontaneous or evoked electrical activity in the brain. SD is widely accepted as the pathophysiological event underlying migraine aura, and may play a role in headache pathogenesis in secondary headache disorders such as ischemic stroke, subarachnoid or intracerebral hemorrhage, traumatic brain injury, and epilepsy. Here, we provide an overview of the pathogenic mechanisms and propose plausible hypotheses on the involvement of SD in primary and secondary headache disorders. SD can activate downstream trigeminovascular nociceptive pathways to explain the cephalgia in migraine, and possibly in secondary headache disorders as well. In healthy, well-nourished tissue (such as migraine), the intense transmembrane ionic shifts, the cell swelling, and the metabolic and hemodynamic responses associated with SD do not cause tissue injury; however, when SD occurs in metabolically compromised tissue (e.g. in ischemic stroke, intracranial hemorrhage, or traumatic brain injury), it can lead to irreversible depolarization, injury and neuronal death. Recent non-invasive technologies to detect SDs in human brain injury may aid in the investigation of SD in headache disorders in which invasive recordings are not possible. SD explains migraine aura and progression of neurological deficits associated with other neurological disorders. Studying the nature of SD in headache disorders might provide pathophysiological insights for disease and lead to targeted therapies in the era of precision medicine.

### S22 Headache in the Emergency Room

#### Anne Ducros

##### University of Montpellier, and Headache Centre, Neurology department, Montpellier University Hospital, France

The proportion of adult patients reporting non-traumatic headache as their major complaint at ER access ranges from 0.5 to 4.5%.The main objective is to identify the patients who require urgent investigations besause of a suspected serious secondary cause. Serious conditions are disclosed in 5-10% of the cases; the remaining patients have benign secondary headaches, or more frequently, primary headaches.

The crucial step in the diagnosis is the initial interview. Most patients presenting with headache as the chief complaint have a primary headache disorder, such as migraine or tension-type headache, the diagnosis of which relies on strict diagnostic criteria in the absence of any objective marker. Secondary headache disorders manifest as new-onset headaches that arise in close temporal association with the underlying cause.Secondary headache should be suspected in any patient without a history of primary headache who reports a new onset headache and in any patient with a new unusual headache that is clearly distinct from their usual primary headache attacks. Since many serious disorders, such as subarachnoid haemorrhage, can present with isolated headache and a normal clinical examination, diagnosis is reliant on clinical investigation.

Subarachnoid hemorrhage should be suspected in anyone with a sudden or a thunderclap headache. Diagnosis is based on plain brain computed tomography and, if tomogram is normal, on lumbar puncture. Reversible cerebral vasoconstriction syndrome should be suspected in anyone with recurrent thunderclap headaches over a few days. Cervical artery dissection, cerebral venous thrombosis, reversible cerebral vasoconstriction syndrome and pituitary apoplexy may present with isolated headache and normal physical examination, normal cerebral computed tomography and normal cerebrospinal fluid. When computed tomography and lumbar puncture are normal, other investigations are needed, including cervical and cerebral vascular imaging and brain magnetic resonance imaging.

Treatment of headaches in the ER should be based on the etiology. A severe migraine attack can be treated by SC sumatriptan, intravenous non-steroidal anti-inflammatory drugs and/or dopamine antagonists. The treatment of secondary headaches requires the treatment of the underlying cause and a symptomatic treatment based on intravenous acetaminophen or on opiates depending on the pain intensity.

### S23 Progestin-only contraception and beneficial effects on migraine

#### Gabriele S. Merki-Feld

In women migraine prevalence peaks during reproductive years. Menstruation is a significant risk factor for migraine with attacks most likely to occur between 2 days before the onset of menstruation and the first three days of bleeding. The pathophysiology of menstrual attacks involves estrogen withdrawal and potentially abnormal release of prostaglandins triggered by the end-cycle drop in estrogen level. Reproductive year are the life span during which many women require effective contraception.

Migraine with aura (MA) and to a lesser extent migraine without aura (MO) increase the risk for cardiovascular events, especially for stroke. There is a substantial elevation of these risks in migraineurs using combined contraceptive pills (COC). In additon it has been shown that COC can initiate migraine, worsen the course of migraine and induce a change from MO to MA. Several clinical trials report improvements in migraine frequency and intensity in users of the progestin-only pill (POP) with desogestrel 75microgram. Both, inhibition of ovulation and ist continous use contribute to reduce hormone flucutations during ist use. In contrast to COC, POP are not associated with an increased risk for stroke. The positive impact of this pill has been shown in MA and MO patients. In women with chronic migraine, the reduction in pain medications used contributes to prevent medication overuse headaches.

### S24 Current Consensus on Classification of the Trigeminal Neuralgia

#### Zaza Katsarava

##### Unna/Essen, Germany

Chapter 13 sets out a classification system for painful lesions of the cranial nerves and other facial pains based on a consensus between the International Headache Society (IHS) and the International Association for the Study of Pain (IASP).

The existing nosology of cranial-nerve pains does not fully portray the subtle differences between various conditions. However, rather than abandoning many long-established diagnostic terms, this classification retains them, providing detailed definitions for differential diagnoses and their types, subtypes and subforms.

There are several axes of classification: a) syndomology (neuralgia vs. neuropathy), b) location (central vs. peripheral neuropathic pain) and c) aethiology (classical, idiopathic or secondary).

The authors of the classification tried to incorporate the existing literature into the IHS classification system.

The current version defines the trigeminal neuralgia and trigeminal neuropathy. Trigeminal neuralgia is subdivided into classical (due to nerve-vascular compression, not purely a nerve vascular contact), idiopathic (unknown cause or nerve vascular contact, because the value of a nerve vascualr contact is unclear) and secondary (due to other disease). Base don the clinical presentation it is further characterised as TN with and without concomitant facial pain indicating pure response to treatment.

### S25 Traumas and headache

#### Mark Braschinsky (mark.braschinsky@kliinikum.ee)

##### Department of Neurology, Tartu University Clinics, Tartu 51014, Estonia

Headache following the trauma or so called post-traumatic headache is on of if not the most common secondary headache disorder, reaching approximately 4 % of all secondary headaches. According to the International Classification of Headache Disorders, 3rd edition (beta version) headache attributed to trauma or injury to the head and/or neck is divided into acute and persistent headache for each separate trauma mechanism – injury to the head, whiplash or craniotomy (performed for reasons other than traumatic head injury) [1]. The cut-line for distinguishing between an acute and persistent headache is defined to be 3 months: resolution of headache within this period complies with an acute, persistence for the longer time – with a persistent headache. Headache attributed to the injury to the head is further subclassified based on the severity of preceding trauma. Probably one of the most debated diagnostic criterions of this chapter is the time of onset of headache after a traumatic event. For the main classification it is agreed that causative relation between trauma and development of headache should be within 7 days after the trauma. However based on a data derived from reports of everyday clinical practice alternative criteria published under the Appendix allow the delayed onset of headache, reaching up to 30 days following the injury. Clinical phenotypes of post-traumatic headache are varying from mild tension-type-like to severe migrainous. Pathophysiological mechanisms of post-traumatic headaches remain largely unclear as a reason to the epidemiological data suggesting, that mild injury to the head represents a greater risk of developing persistent headache. The latter one causes a considerable reduction of health related quality of life and frequently is challenging in terms of treatment, requiring pharmacological (preventative medications) and non-pharmacological (cognitive behavioural treatment, physical therapy, counselling etc) approaches. For treatment resistant cases interventional procedures, usage of onabotulinum toxin A and neurostimulation have been reported to be potentially effective.

### S26 Within person variation in headache days in persons with migraine

#### Richard Lipton

**Objective**

To determine persistence of and transitions between episodic migraine (EM) and chronic migraine (CM) and to describe and model the natural variability of self-reported frequency of headache days

**Background**

Relatively little is known about the stability of headache days per month in persons with EM or CM over time. Within person variability in headache day frequency has implications for the diagnosis of CM, assessing treatment in clinical practice and for the design and interpretation of clinical trials.

**Methods**

The Chronic Migraine Epidemiology and Outcomes (CaMEO) Study is a longitudinal survey of a systematic sample of US adults with EM and CM identified by a web-questionnaire. A validated questionnaire was used to classify respondents with EM (<15 headache days/month) or CM (≥15 headache days/month) every 3 months for a total of 5 assessments. We modelled longitudinal transitions between EM and CM and, separately, headache day frequency per month using negative binomial repeated measures regression models (NBRMR). The NBRMR was parameterized using polynomial mixed effects to better account for cyclic variation.

**Results**

Among the 5,464 respondents with EM at baseline providing 4 or 5 waves of data, 5,048 (92.4%) had EM in all waves and 416 (7.6%) had CM in at least one wave. Among 526 respondents with CM at baseline providing 4 or 5 waves of data, 140 had CM in every wave (26.6%) and 386 (73.4%) had EM for at least one wave. Individual plots revealed striking within-person variations in headache days per month. The polynomial mixed effect NBRMR model revealed that the rate of headache days increased across waves of observation 19% more per wave for CM compared to EM (rate ratio [RR], 1.19; 95% CI, 1.13 – 1.26). The inclusion of a comprehensive covariate pool in the fully adjusted model increased this difference to a 26% increase per wave (RR, 1.26; 95% CI, 1.2 – 1.33).

**Conclusions**

Follow-up at 3 month intervals reveals a high level of short-term variability in headache days per month. As a consequence many individuals cross the arbitrary CM diagnostic boundary of ≥15 headache days per month over the course of one year. Nearly three forths of persons with CM at baseline drop below this diagnostic boundary at least once over the course of a year. These findings my influence case definitions of migraine subtypes, the design and interpretation of epidemiologic studies and clinical trials as well as the interpretation of change in headache days in clinical practice.

### S27 Migraine and the Glymphatic System

#### Rami Burstein and Aaron Schain

##### Department of Anesthesia, Critical Care and Pain Medicine, Beth Israel Deaconess Medical Center, Boston MA 02115 and Harvard Medical School, Boston, MA 02215, USA

Impairment of brain solute clearance through the recently described glymphatic system has been linked with traumatic brain injury, sleep deprivation, and aging. This lecture will summarize new data showing that cortical spreading depression (CSD), the neural correlate of migraine aura, closes the paravascular space and impairs glymphatic flow. This closure holds the potential to define a novel mechanism for regulation of glymphatic flow. It also implicates the glymphatic system in altered cortical and endothelial functioning of the migraine brain, which can explain the increased risk of stroke among migraine aura patients.

### S28 Photophobia and Hypothalamus

#### Rami Burstein, Rodrigo Noseda

##### Department of Anesthesia, Critical Care and Pain Medicine, Beth Israel Deaconess Medical Center, Boston MA 02115 and Harvard Medical School, Boston, MA 02215, USA

Many patients report that their need to avoid light is driven mainly by how unpleasant it makes them feel. This lecture will attempt to explain why is light unpleasant. The data presented will show that during migraine, light can trigger the perception of (a) hypothalamic-mediated autonomic responses such as chest tightness, throat tightness, shortness of breath, fast breathing, faster than usual heart rate, light-headedness, dizziness, nausea, vomiting, dry mouth, salivation, rhinorrhea, stuffy sinuses and lacrimation; (b) hypothalamic mediated non-autonomic responses such as thirst, hunger drowsiness, tiredness, sleepiness, fatigue, and yawning; (c) negative emotions such as intense, irritable, angry, nervous, hopeless, needy, agitated, sad, scared, cranky, upset, depressed, disappointed, jittery, worried, stressed, anxious, panic and fear; and (d) positive emotions such as happy, relaxing, soothing, and calming. The data presented will also show that retinal axons converge on dopaminergic/noradrenergic, histaminergic, orexinergic, MCHergic, oxytocinergic and vasopressinergic hypothalamic neurons that regulate autonomic functions and emotions. By defining better the aversive nature of light, the findings suggest that the retina and hypothalamus play a critical role in migraine-type photophobia and that photophobia may not depend on hyperexcitable visual cortex, as traditionally thought.

### S29 The Gymphatic System

#### Maiken Nedergaard

We have recently described a macroscopic pathway in the central nervous system – the glymphatic system that facilitates the clearance of interstitial waste products from neuronal metabolism. Glymphatic clearance of macromolecules is driven by cerebrospinal fluid (CSF) that flows in along para-arterial spaces and through the brain parenchyma via support from astroglial aquaporin-4 water channels. The glymphatic circulation constitutes a complete anatomical pathway; para-arterial CSF exchanges with the interstitial fluid, solutes collect along para-venous spaces, then drain into the vessels of the lymphatic system for ultimate excretion from the kidney or degradation in the liver. As such, this may after circulation represent a novel and unexplored target for prevention and treatment of neurodegenerative diseases.

### S30 A population based survey for headaches in greece

#### Dimos D. Mitsikostas^1^, Chrisanthy Arvanity^2^, Theodoros Constantinidis^3^, Manolis Dermitzakis^4^, Nikolaos Fakas^5^, Jobst Rudolf^6^, Michail Vikelis^7^, on behalf of the Hellenic Headache Society

##### ^1^First Neurology Department, Aeginition Hospital, School of Medicine, National & Kapodistrian University of Athens, Athens, Greece; ^2^Second Neurology Department, Attikon Hospital, School of Medicine, National & Kapodistrian University of Athens, Athens, Greece; ^3^Private Headache Clinic, Korinthos, Greece; ^4^Department of Neurology, “Geniki Kliniki” Euromedica, Thessaloniki, Greece; ^5^401 Army General Hospital of Athens, Neurology Department, Athens, Greece; ^6^Neurology Department, Papageorgiou Hospital, Thessaloniki, Greece; ^7^Headache Clinic, Mediterraneo Hospital, Glyfada, Greece

We aimed to investigate the prevalence of headache in General Population (adults 18-70 years old) in Greece. A quantitative study, using the form of computer-assisted telephone interviews (C.A.T.I.) was designed. A draft questionnaire consisting of 37 questions was delivered in 145 headache sufferers in a pre-study work to evaluate the diagnosis of the primary headache disorder according to ICH-3beta diagnostic criteria. After the analysis of this questionnaire the specific 37-item questionnaire was decided. In total, N=10,008 interviews, representative of the population of Greece in terms of gender, age, and area, based on the most recent census (ELSTAT, 2011) were performed using the structured evaluated questionnaire. Based on the above contacts, n=1,197 respondents (12% of the sample) were found to suffer from headaches that reduce their performance. The one-year prevalence of Migraine that reduces activity was 8.2% (n=0.6m population) of Tension-Type Headache (TTH) 3.8% (n=0.28m of population) and of Cluster Headache 0.01% (n=0.74K of population). Chronic migraine one-year prevalence was 1% (n=0.7K of population). Females tend to suffer more from migraines and TTH as well as ages 35-54. The average patients has been suffering from headaches for 12 years. Headaches typically occur once a month or more frequently, 8 days per month on average. Although patients rarely misss work due to headaches, they do report headache-induced reductions in performance around 3 days per month. Slighly less than half patients have felt bad/ humiliated because of headaches, while social/family obligations are affected 3 days per month on average. About one fifth of patients seek professional treatment for headaches, most of them in the private sector. The most popular specialty for headache treatment is neurologist, followed by internist. Regarding both prophylactic and acute treatment, patients prefer oral medication to injection, even if the former is administered more frequently. They also prefer oral medication/ injection to a stimulation device. The stimulation device seems to be more attractive to males. Painkillers also are by far the most common acute treatment for headaches and the vast majority of patients have never taken prophylaxis for headaches. Only a small fraction have stopped taking a prophylactic treatment due to adverse effects. Interstingly, patients would be willing to spend 20€ on average per month for headache treatment, on average.

### S31 The big CGRP flood - sources, sinks and signalling sites in the trigeminovascular system

#### Karl Messlinger

##### Institute of Physiology and Pathophysiology, Friedrich-Alexander-University of Erlangen-Nürnberg, 91054 Erlangen, Germany

Calcitonin gene-related peptide (CGRP), a neuropeptide previously known only by specialists interested in neurogenic inflammation, is now discussed throughout the communities of migraine researchers, headache therapists and even migraine patients. The reason for this surprising career of CGRP awareness is evident. CGRP is the main neuropeptide of a major part of nociceptive trigeminal afferents and is released upon their activation. Thus CGRP release is characteristic, though in no way specific, for the trigeminovascular system, which is regarded as the structural basis for headache generation. In fact, CGRP has been found at elevated concentrations in the cranial outflow during attacks of migraine and some trigemino-autonomic headaches; infusion of CGRP into patients suffering from primary headaches can cause head pain mimicking their spontaneous headache attacks; inhibiting CGRP or its receptors or its release can be preventive or therapeutic in those types of primary headaches. However, looking behind the curtain of impressive significance of this biomarker, broad gaps in our knowledge are visible concerning the sites of CGRP release, its flow through the meningeal compartments, the sites and mechanisms of actions and its elimination. With preclinical experiments we are only at the beginning to study these issues, which are increasingly important in the light of new pharmacological developments targeting CGRP and its receptors by antagonists or monoclonal antibodies, and keeping in mind possible risks of a long-term treatment with these substances. Trigeminal activity controlled by CGRP receptor activation could indeed be a pivot point in headache generation and therapy. However, measurable circulating concentrations of CGRP are far too low to explain any receptor effects, while it is difficult to assess its real concentrations near the likely release sites, namely the meningeal terminals of trigeminal afferents, the trigeminal ganglion and the central terminals in the trigemino-cervical brainstem complex. The central effects of CGRP as a synaptic neuromodulator could explain neuronal CGRP effects to some extent but big molecules like monoclonal antibodies are unlikely to pass the blood-brain barrier and may not be able to act there. Peripheral effects of CGRP are largely confined to its well-known vascular functions, while fast neuronal effects are not established so far in the trigeminal system. The trigeminal ganglion is a possible point of CGRP action but only few experiments have shown an impact on the signalling or metabolic changes of ganglion neurons. Therefore new experimental approaches are needed to uncover the secrets of the nociceptive CGRP signalling system and its therapeutic control.

### S32 EHF-LTB Aids to management of headache disorders in primary care (2^nd^ edition)

#### TJ Steiner^1,2^, R Jensen^3^, Z Katsarava^4,5^, M Linde^1,6^, EA MacGregor^7^, P Martelletti^8,9^, V Osipova^10,11^ and K Paemeleire^12^, on behalf of Lifting The Burden: The Global Campaign against Headache and the European Headache Federation

##### ^1^Department of Neuroscience, Norwegian University of Science and Technology (NTNU), Trondheim, Norway; ^2^Division of Brain Sciences, Imperial College London, London, UK; ^3^Danish Headache Centre, Department of Neurology, University of Copenhagen, Glostrup Hospital, Glostrup, Denmark; ^4^Department of Neurology, Evangelical Hospital Unna, Unna, Germany; ^5^Medical Faculty, University of Duisburg-Essen, Essen, Germany; ^6^Norwegian Advisory Unit on Headache, St Olavs Hospital, Trondheim, Norway; ^7^Centre for Neuroscience and Trauma, Blizard Institute of Cell and Molecular Science, Barts and the London School of Medicine and Dentistry, London, UK; ^8^Department of Clinical and Molecular Medicine, Sapienza University, Rome, Italy; ^9^Regional Referral Headache Centre, Sant’Andrea Hospital, Rome, Italy; ^10^Research Department of Neurology, First “I. Sechenov” Moscow State Medical University, Moscow, Russian Federation; ^11^ Research Center for Neuropsychiatry, Moscow, Russian Federation; ^12^ Department of Neurology, Ghent University Hospital, Ghent, Belgium

###### **Correspondence:** P Martelletti (paolo.martelletti@uniroma1.it)

Medical management of headache disorders, for the vast majority of people affected by them, can and should be carried out in primary care. It does not require specialist skills. Nonetheless, it is recognised that non-specialists throughout Europe may have received limited training in the diagnosis and treatment of headache.

This publication, in the *Journal of Headache and Pain*, provides a combination of educational materials and practical management aids. It is a product of the Global Campaign against Headache, a programme of action for the benefit of people with headache conducted by the UK-registered non-governmental organization *Lifting The Burden* (LTB) in official relations with the World Health Organization. It updates the first edition [1], published 10 years ago.

The content has been put together by a writing group of experts convened by *Lifting The Burden* in collaboration with the European Headache Federation (EHF). It has undergone review by a wider consultation group of headache experts, including representatives of the member national societies of EHF, primary-care physicians from eight countries of Europe, and lay advocates from the European Headache Alliance. While the focus is Europe, the inclusion in the consultation group of members from all six world regions has aimed for cross-cultural relevance of all content so that it is useful to a much wider population.

The *European principles of management of headache disorders in primary care*, laid out in 11 sections, are the core of the content. Each of these is more-or-less stand-alone, in order to act as practical management aids as well as educational resources. There is a set of additional practical management aids. An abbreviated version of the International Classification of Headache Disorders, 3rd edition (ICHD-3), provides diagnostic criteria for the few headache disorders relevant to primary care. A headache diary further assists diagnosis and a headache calendar assists follow-up. A measure of headache impact (the HALT-90 index) can be employed in pre-treatment assessment of illness severity, and an outcome measure (the HURT questionnaire) is a guide to follow-up and need for treatment-review. Five patient information leaflets are included, which may be offered to patients to improve their understanding of their headache disorders and their management.

LTB and EHF offer these aids freely available for use without restriction. We hope for benefits for both physicians and patients.

### S33 Combined hormonal contraception and migraine, WHO and EHF/ESCRH criteria and balancing risks and benefit

#### Simona Sacco (simona.sacco@univaq.it)

##### Neurology section, Department of Applied Clinical Science and Biotechnology, University of L’Aquila, L’Aquila, Italy

Several data indicate that migraine, especially migraine with aura, is associated with an increased risk of ischemic stroke and other vascular events. Of concern is whether the risk of ischemic stroke in migraineurs is magnified by the use of hormonal contraceptives (HCs). As migraine prevalence is high in women of reproductive age, it is common to face the issue of migraine and HC use in clinical practice.

To improve decision-making on the use of HCs in women with migraine, a selected group of representatives from the European Headache Federation (EHF) and the European Society of Contraception and Reproductive Health (ESC) developed a Consensus Statement on this topic. The document pointed out that evidence addressing the risk of ischemic stroke associated with the use of HCs is generally poor. All information relies on observational data, which may carry the risk of potential bias. Available studies had different settings and used different groups for comparing risks, limiting reliable comparison of studies as a pooled analysis of data. Most of the available studies were published several years ago and used compounds which are different from those available today. Additionally, in most studies not enough information is available regarding the type of HC considered and in most cases results are not provided according to migraine type. Despite those limitations, available data pointed toward an increased risk of ischemic stroke associated with the use of HCs in women with migraine. Literature indicated that, whereas combined HCs carry a certain risk of arterial ischemic events this does not happen for progestogens-only HCs which are considered safe in terms of cardiovascular risk even in the presence of associated risk factors. Considering those data, and unless studies will prove safety of the use of combined HCs in women with migraine, the recommendations from the Consensus Group gave priority to safety and suggested several limitations in the use of combined HCs in women with migraine. There are alternative methods to combined HCs which provide similar contraceptive benefits but that are much safer in terms of risks. Further research is need to address safety of newer compounds in women with migraine.

**References**

Sacco S, Merki-Feld GS, Ægidius KL, Bitzer J, Canonico M, Kurth T, Lampl C, Lidegaard Ø, MacGregor EA, MaassenVanDenBrink A, Mitsikostas D, Nappi RE, Ntaios G, Sandset PM, Martelletti P; on behalf of the European Headache Federation (EHF) and the European Society of Contraception and Reproductive Health (ESC). Hormonal contraceptives and risk of ischemic stroke in women with migraine: a consensus statement from the European Headache Federation (EHF) and the European Society of Contraception and Reproductive Health (ESC). J Headache Pain 2017;in press.

### S34 Neuropathic pain: basic concepts

#### Rolf-Detlef Treede

##### Department of Neurophysiology, Center for Biomedicine and Medical Technology Mannheim, Heidelberg University, Germany

Neuropathic pain is pain caused by a lesion or disease of the somatosensory nervous system. The term *lesion* is refers to nervous system damage demonstrated by imaging, neurophysiology, biopsies or surgical evidence. The term *disease* is used when the nervous system damage is due to a neurological disorder such as stroke or peripheral diabetes neuropathy. In peripheral neuropathic pain there is usually a mixture of damaged and undamaged axons within the peripheral nerve, leading to the clinical presentation with ongoing pain, sensory loss and sensory gain (hyperalgesia, allodynia). The clinical presentation in central neuropathic pain is similar, but the mechanisms are less well understood. Mechanisms of peripheral neuropathic pain include ectopic impulse generation, peripheral sensitization of undamaged nerve fibers, and central sensitization; the latter includes altered signal processing in the CNS due to changes in descending pain modulation. Neuropathic pain is included in the upcoming ICD-11 coding system, but not in the currently used classifications ICD-10 and ICD-9. For this reason the exact prevalence of neuropathic pain is not yet known, but is expected to be high due to the high prevalence of the underlying neurological disorders.

### S35 Migraine and cerebellum

#### Koppen Hille

A range of clinical neurophysiological and functional imaging studies have suggested that migraine might be associated with cerebellar dysfunction. These studies all had methodological short-comings to a greater or lesser extent. Therefore, it is still uncertain whether migraine is associated with cerebellar dysfunction, and, if so, to what extent and why. Is this presumed cerebellar dysfunction due to the increased prevalence of cerebellar ischemic lesions in migraine patients or is there a more functional explanation similar to what’s seen in familial hemiplegic migraine type 1 (FHM1)? Recent anatomical studies demonstrated that the output of the cerebellum targets multiple non-motor areas in the prefrontal and posterior parietal cortex. Neuro-anatomy and functions of the cerebellum will be reviewed as well as the evidence of cerebellar infarcts in migraineurs. In detail results of the population-based CAMERA II (Cerebral Abnormalities in Migraine, an Epidemiological Risk Analysis Cohort) study specific on cerebellar ischemia and cerebellar function will be discussed.

### S36 Neurophysiology of Headaches

#### Gianluca Coppola

##### G.B. Bietti Foundation-IRCCS, Research Unit of Neurophysiology of Vision and Neurophthalmology, Rome, Italy

During the last decades, the methods of neurophysiology proved to be very effective in disclosing subtle functional abnormalities of the brain of patients affected by primary headache disorders. These methods received several refinements during the last years, further improving our understanding of headaches pathophysiology. Abnormal increased responsivity was several times revealed with almost all the sensory modalities of stimulation in migraine between attacks, with its normalization during the attacks. Recently, authors observed that the degree of some neurophysiological abnormalities might depends on the distance from the last attack, i.e. on the point where the patient is recorded during the migraine cycle. Thalamic/thalamocortical drives were found to be less active interictally, but normally active ictally. Somatosensory cortex lateral inhibition, gating, and interhemispheric inhibition were altered in migraine, and may contribute to cortical hyperresponsivity and clinical features.

Cluster headache patients are characterized by a deficient habituation of the brainstem blink reflex during the bout, outside of attacks, on the affected side. Evidence for sensitization of pain processing was disclosed by studying temporal summation threshold of the nociceptive withdrawal reflex, which was less modulated by supraspinal descending inhibitory controls.

In conclusion, much has been discovered and much more needs to be investigated to better understand what causes, how it triggers, keeps and runs out recurrent primary headaches. Clarifying some of these mechanisms might help in the identification of new therapeutic targets.

### S37 Mechanisms of Photophobia

#### Andrew Russo

In this rejoinder to “Photophobia and Hypothalamus”, I will speculate on how the diverse collection of neuropeptides, including CGRP, in the hypothalamus might increase sensitivity to light. Within the brain, neuropeptides can modulate the strength of synaptic signaling even at a relatively large distance from their site of release. Given the evidence for CGRP in migraine and potential roles for other hypothalamic peptides, it seems likely that altered neuropeptide actions may be a general theme underlying the heightened sensory state of migraine. Towards this point, I will briefly discuss our preclinical CGRP and optogenetic studies using light aversive behavior in mouse models as a surrogate for migraine-associated photophobia. I will describe how both the brain and the periphery are susceptible to elevated CGRP and how CGRP appears to act by distinct mechanisms in these sites. In the CNS, we have identified the posterior thalamus as a likely site of CGRP action, which is in agreement with Burstein’s evidence that this region is a convergent relay point from the retina and dura. These ideas will be tied together in a speculative model that integrates peripheral and central CGRP actions in photophobia.

### S38 Classical trigeminal neuralgia – clinical and MRI findings

#### Stine Maarbjerg

##### Department of Neurology, Helse Fonna, Haugesund, Norway

**Background**

Classical trigeminal neuralgia (TN) is a unique neuropathic facial pain disorder. As there are no diagnostic tests to confirm the diagnosis, it relies on a thorough history and exam. MRI is used to exclude symptomatic trigeminal neuralgia, not to confirm the diagnosis of TN. Knowing how to interpret MRI findings is of importance with respect to surgical treatment options and their expected chance of a successful outcome.

**Results**

TN is characterized by paroxysms of unilateral intense pain usually in the 2^nd^ and 3^rd^ trigeminal branch. The pain quality is stabbing and the pain is typically evoked by sensory stimuli like light touch, brushing teeth, cold wind or eating. Up to half of the patients also have concomitant persistent pain. A smaller proportion of patients may have sporadic autonomic symptoms. The average age of disease onset is in the early fifties and TN is slightly more prevalent in women than in men.

As a general rule, the neurological exam is normal in TN patients. As objective signs of TN, patients may wince at pain paroxysms and may avoid shaving or brushing their teeth on the affected side. Some studies argue that a proportion of TN patients have subtle sensory abnormalities at bedside exam, primarily hypoesthesia. Studies using quantitative sensory testing also documented sensory changes in TN. Rather than indicating nerve damage, the findings may be explained by functional changes of the nervous system in response to severe pain.

There is widespread consensus that TN is associated to a neurovascular contact between the trigeminal nerve and a blood vessel in the prepontine course of the nerve. Emerging advanced imaging studies confirms that at the site of a neurovascular contact on the ipsilateral side of pain, there is of demyelination – a process that seems to be reversible in some patients after successful surgery. Imaging studies also consistently show that TN is strongly associated to a neurovascular contact with morphological changes of the trigeminal nerve, i.e. dislocation, distortion or atrophy of the trigeminal nerve. Meanwhile, only half of TN patients have morphological changes of the trigeminal nerve and there may be other unknown etiological factors causing TN.

**Conclusions**

The talk discusses the clinical features and the clinical and MRI findings of TN. The pearls and pitfalls of TN diagnosis and neuroimaging is discussed from both a clinical and a scientific perspective.

### S39 PACAP in migraine

#### László Vécsei^1,2^, Délia Szok^1^, János Tajti^1^

##### ^1^Department of Neurology, Faculty of Medicine, Albert Szent-Györgyi Clinical Center University of Szeged, H-6725 Szeged, Semmelweis u. 6, Hungary; ^2^MTA-SZTE Neuroscience Research Group, University of Szeged, H-6725 Szeged, Semmelweis u. 6, Hungary

###### **Correspondence:** László Vécsei (vecsei.laszlo@med.u-szeged.hu)

**Background**

Pituitary adenylate cyclase-activating polypeptide (PACAP) is a member of the vasoactive intestinal polypeptide (VIP)/secretin/growth hormone-releasing hormone/glucagon neuropeptide superfamily, widely expressed in vertebrate tissues [1]. The first evidence for potential role of PACAP in pathomechanism of migraine was the intravenous administration of PACAP-38 caused headache and vasodilatation in healthy subjects as well as in migraineurs, and lead to delayed-type migraine-like attacks [2]

**Materials and methods**

A systematic literature search was conducted to identify preclinical and clinical publications in the field of PACAP and migraine in the database of MEDLINE/PubMed up to 31 May 2017.

**Results**

Preclinical experiments revealed that both PACAP-27 and PACAP-38 were found elevated in the trigeminal nucleus caudalis of rats following electrical stimulation of the trigeminal ganglion or chemical stimulation by nitroglycerin of the trigeminovascular system [3]. A magnetic resonance imaging (MRI) angiographic study demonstrated that PACAP-38-induced headache was associated with prolonged dilatation of the middle meningeal arteries, but not of the middle cerebral arteries in healthy volunteers [4]. Another MRI trial concluded that PACAP-38-induced vasodilatation was longer lasting compared to VIP in migraineurs [5]. The recent functional imaging study pointed that intravenous PACAP-38-induced migraine attacks was associated with alterations in brain network connectivity [6]

Clinical investigation provided evidence of a clear association between migraine phases (during a spontaneous migraine attack versus pain-free period) and the alteration of plasma PACAP-38 level [7].

**Conclusions**

The activation and sensitization of the trigeminovascular system by vasoactive neuropeptides might be crucial factors of the migraine pathogenesis [8]. The recent preclinical and clinical studies suggest the importance of PACAP as a future biomarker of migraine headache.

**Acknowledgements**

This work was supported by the projects EUROHEADPAIN FP7-Health 2013-Innovation; Grant No. 602633; GINOP-2.3.2-15-2016-00034, KTIA_NAP_13-2014-0022.

**References**

1. Arimura A. PACAP: the road to discovery. Peptides. 2007; 28:1617-1619.

2. Schytz, H.W., Birk, S., Wienecke, T., Kruuse, C., Olesen, J., Ashina, M. PACAP38 induces migraine-like attacks in patients with migraine without aura. Brain. 2009; 132:16-25.

3. Tuka, B., Helyes, Z., Markovics, A., Bagoly, T., Nemeth, J., Mark, L., Brubel, R., Reglodi, D., Pardutz, A., Szolcsanyi, J., Vecsei, L., Tajti, J. Peripheral and central alterations of pituitary adenylate cyclase activating polypeptide-like immunoreactivity in the rat in response to activation of the trigeminovascular system. Peptides 2012; 33:307-316.

4. Amin, F.M., Asghar, M.S., Guo, S., Hougaard, A., Hansen, A.E., Schytz, H.W., van der Geest, R.J., de Koning, P.J., Larsson, H.B., Olesen, J., Ashina, M. Headache and prolonged dilatation of the middle meningeal artery by PACAP38 in healthy volunteers. Cephalalgia 2012;32: 140-149.

5. Amin, F.M., Hougaard, A., Schytz, H.W., Asghar, M.S., Lundholm, E., Parvaiz, A.I., de Koning, P.J., Andersen, M.R., Larsson, H.B., Fahrenkrug, J., Olesen, J., Ashina, M. Investigation of the pathophysiological mechanisms of migraine attacks induced by pituitary adenylate cyclase-activating polypeptide-38. Brain 2014; 137:779-794.

6. Amin, F.M., Hougaard, A., Magon, S., Asghar, M.S., Ahmad, N.N., Rostrup, E., Sprenger, T., Ashina, M. Change in brain network connectivity during PACAP38-induced migraine attacks: A resting-state functional MRI study. Neurology 2016; 86:180-187.

7. Tuka, B., Helyes, Z., Markovics, A., Bagoly, T., Szolcsanyi, J., Szabo, N., Toth, E., Kincses, Z.T., Vecsei, L., Tajti, J. Alterations in PACAP-38-like immunoreactivity in the plasma during ictal and interictal periods of migraine patients. Cephalalgia 2013; 33:1085-1095.

8. Vécsei L, Tuka B, Tajti J. Role of PACAP in migraine headaches. Brain 2014; 137:650-651.

### S40 Comorbidity Grounds

#### Noemi Faedda^1^, Vincenzo Guidetti^2^, Giulia Natalucci^2^

##### ^1^PhD program in Behavioural Neuroscience, Department of Paediatrics and Child and Adolescent Neuropsychiatry, Sapienza University of Rome; ^2^Department of Paediatrics and Child and Adolescent Neuropsychiatry, Sapienza University of Rome

###### **Correspondence:** Vincenzo Guidetti (vincenzo.guidetti@uniroma1.it)

Several studies are found a relationship between headache and psychiatric comorbidity in both children and adolescents [1-3]. The most frequently described comorbidities include anxiety, mood disorders [1], sleep disorder [2] and attention hyperactive disorder [3]. The association between headache and comorbidities has been interpreted in the light of different possible causal pathways. Psychiatric comorbidity may represent the consequence of a link between neurotransmitter systems involved in migraine and psychiatric disorder, such as depression and anxiety [4]. A central role is thought to be played by serotonergic receptors, adrenergic and dopaminergic D2 receptor genotype, that seem to be associated with migraine, major depression, generalized anxiety disorder, panic attacks and phobia [5]. It has been suggested that the patient's vulnerability to anxiety disorders and affective disorders as well as migraine might be attributed to the dysregulation of the serotonergic system [6]. Furthermore, it is possible that each disorder increases the risk of the other [4;7]. Twin studies have shown that the genetic liability related to migraine amounts to 40-60%, while the contribution of non-shared environmental factors has to be weighed in a range between 35 and 55% [8]. Therefore, the relevance of other mediating factors for the co-occurrence of headache and psychiatric comorbidity has to be taken into consideration. Recent research found that an insecure attachment may be a risk factor for an outcome of poor adaptation that includes chronic pain [9] and that pain perception may change in relation with specific attachment styles.

The ambivalent attachment seems to be the most common style among patients reporting high attack frequency and severe pain intensity and in children with this attachment style there is a relationship between high attack frequency and high anxiety levels [10]. Barone et al. [11] showed that higher is the attachment security, lower is the association between maternal stress and children’s behavioural problems in children and adolescents with headache. Although more studies are needed in order to detect the biological, genetic and environmental mechanisms underlying the relationship between headache and comorbidities, attachment styles can be regarded as one of the factors mediating this association [12].

**References**

1. Blaauw BA, Dyb G, Hagen K, Holmen TL, Linde M, Wentzel-Larsen T, Zwart JA. Anxiety, depression and behavioral problems among adolescents with recurrent headache: the Young-HUNT study. J Headache Pain. 2014;15:38.

2. Guidetti V, Dosi C, Bruni O. The relationship between sleep and headache in children: implications for treatment. Cephalalgia. 2014 Sep;34(10):767-76.

3. Parisi P, Verrotti A, Paolino MC, Ferretti A, Raucci U, Moavero R, Villa MP, Curatolo P. Headache and attention deficit and hyperactivity disorder in children: common condition with complex relation and disabling consequences. Epilepsy Behav. 2014;32:72-5

4. Antonaci F, Nappi G, Galli F, Manzoni GC, Calabresi P, Costa A. Migraine and psychiatric comorbidity: a review of clinical findings. J Headache Pain. 2011;12(2):115-25.

5. Peroutka SJ, Price SC, Wilhoit TL, Jones KW. Comorbid migraine with aura, anxiety, and depression is associated with dopamine D2 receptor (DRD2) NcoI alleles. Mol Med. 1998;4:14–21.

6. Liu H, Liu M, Wang Y, et al. Association of 5-HTT gene polymorphisms with migraine: a systematic review and meta-analysis. *J Neurol Sci* 2011; 305(1-2): 57-66.

7. Bellini B, Arruda M, Cescut A, Saulle C, Persico A, Carotenuto M, Gatta M, Nacinovich R, Piazza FP, Termine C, Tozzi E, Lucchese F, Guidetti V. Headache and comorbidity in children and adolescents. J Headache Pain 2013; 14:79.

8. Mulder EJ1, Van Baal C, Gaist D, Kallela M, Kaprio J, Svensson DA, Nyholt DR, Martin NG, MacGregor AJ, Cherkas LF, Boomsma DI, Palotie A. Genetic and environmental influences on migraine: a twin study across six countries. Twin Res. 2003 Oct;6(5):422-31.

9. Lumley MA, Cohen JL, Borszcz GS, Cano A, Radcliffe AM, Porter LS, Schubiner H, Keefe FJ. Pain and emotion: a biopsychological review of recent research. J Clin Psychol 2011; 67 (9): 942-968).

10. Esposito M, Parisi L, Gallai B, Marotta R, Di Dona A, Lavano SM, Roccella M, Carotenuto M. Attachment styles in children affected by migraine without aura. Neuropsychiatr Dis Treat. 2013;9:1513-9.

11. Barone L, Lionetti F, Dellagiulia A, Galli F, Molteni S, Balottin U. Behavioural problems in children with headache and maternal stress: is children's attachment security a protective factor? Inf. Child. Dev 2015; DOI: 10.1002/icd.1950.

12. Williams R, Leone L, Faedda N, Natalucci G, Bellini B, Salvi E, Verdecchia P, Cerutti R, Arruda MA, Guidetti V. The role of attachment insecurity in the emergence of anxiety symptoms in children and adolescents with migraine: an empirical study. J Headache Pain (In Press)

## SISC INVITED SPEAKERS

### S41 Application of “very low-calorie ketogenic diet” in migraine treatment

#### Cherubino Di Lorenzo^1^, Roberta Ienca^2^, Simona Sodano^2^, Gianluca Coppola^3^, Francesco Pierelli^4,5^

##### ^1^Don Carlo Gnocchi Onlus Foundation, Milan, Italy; ^2^Department of Experimental Medicine-Medical Physiopathology, Food Science and Endocrinology Section, Sapienza University, Rome, Italy; ^3^G.B. Bietti Foundation – IRCCS, Department of Neurophysiology of Vision and Neurophthalmology, Rome, Italy; ^4^Department of Medico-Surgical Sciences and Biotechnologies, Sapienza University of Rome, Rome, Italy; ^5^IRCCS – Neuromed, Pozzilli (IS), Italy

###### **Correspondence:** Cherubino Di Lorenzo (cherub@inwind.it)

**Background.** Metabolic syndrome and overweight are highly prevalent among migraineurs and the weight-loss was suggested as a useful strategy to improve both migraine and metabolic syndrome. Among different approaches to achieve a rapid weight loss, in the last years the very low-calorie diets (VLCDs), characterized by a dramatic caloric restriction (<800 Kcal/day), are gaining large dietician approval. Recently, we have observed that a particular version of VLCD characterized by very low-carbohydrate intake and Ketone bodies (KBs) production, named very low-calorie ketogenic diet (VLCKD), was able to induce a rapid improvement of headache in migraineurs. To assess if the favorable outcome on migraine was due to the caloric restriction, instead of KBs, we performed a double blind crossover study to compare headache modifications during a VLCD and a VLCKD in a population of overweighed and obese migraineurs.

**Methods**. Among patients referred to the Sapienza University Obesity Clinic, a neurologist specializing in headache recruited 35 migraineurs. After one month of headache diary recording, they started a 4-month weight-loss program characterized by the alternation of two VLCD protocols named “red” and “blue”; one of them was a VLCKD, the other a non-ketogenic VLCD. Randomly patients started with one of the two diets according to the following scheme: first VLCD, transition diet (progressive increase of calorie, up to 1200 Kcal/day), the other VLCD, and the second transition diet (Fig. 1). To verify variations in headache frequency, we used as baseline the month before the first VLCD and the first transition diet.

**Results**. Out 35 enrolled patients, six dropped at the first month of diet: all followed the “blue” diet; 29 completed the study. The primary endpoint was the responder rate (number of patients with a headache frequency reduction ≤50%): 26 of 29 patients (74.28% of intention to treat (ITT) patients) responded to the “red diet”, only 2 (5.7% of ITT patients) responded to the “blue diet”. When the blind was broken, we found out that the “red diet” was the ketogenic diet and the “blue diet” was the non-ketogenic.

**Conclusions**. Our results are suggestive for an outstanding protective effect of VLCKD in migraineurs. This positive outcome could be due to the KBs GABAergic, anti-inflammatory, and energetic properties. The 17% of dropout rate is in line with other similar studies and it is interesting to highlight that all the patients that drops did it during the first month of non-ketogenic VLCKD.

**Fig. 1 (abstract S41).**
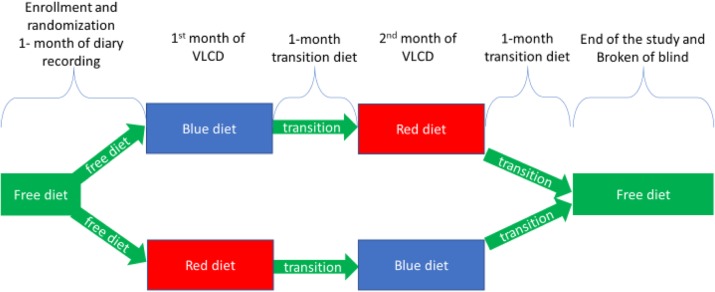
See text for description

### S42 Real Clinical Practice: Physiotherapy Evaluation of Disorders cranial-cervical-mandibular headaches in ebm

#### Riccardo Rosa (clinicadelmalditesta@gmail.com)

##### ^1^La Sapienza University of Roma, Italy; ^2^CLINICADELMALDITESTA, via campania 37, Roma, 00161, Italy

Headaches are one of the most disabling disorders [1]. The 3rd edition of the International Classification of Headache Disorders (ICHD-III) describes the diagnostic criteria of primary, secondary and other headache disorder types. Interestingly, Migraine and Cervicogenic Headache (CGH) , Tension Type Headache (TTH), Headache attributed to temporo-mandibular disorder (TMD), Headache attributed to cervical myofascial pain and Occipital Neuralgia share similarities in some criteria and clinical presentation. Moreover, Neck Pain associated disorders (NAD) and Temporo-Mandibular Disorders (TMD) are a very common presentation in primary headache population as Migraine and Tension Type Headache [2, 3]. Moreover, recent knowledge have suggested that physical examination for provocative procedures should be done on each patient with side- locked headaches as many of these headaches may closely mimic primary headaches [4]. There have been identified eleven physical tests to properly assess cervical disorders. When these dysfunctions are present, they support a reciprocal interaction between the trigeminal and the cervical systems as a trait symptom in migraine [6, 7]. The ICHD-III also does recommend the use of diagnostic criteria evolved by the International RDC/TMD Consortium Network and Orofacial Pain Special Interest Group to assess disorder involving structures in the temporomandibular region contributing to primary headache [8, 9]. In this presentation, an evidence based physical protocol of specific tests it will be provided by a physiotherapist to assess musculoskeletal disorders in the most common primary headaches as Migraine and Tension Type Headache. Moreover, the integration of this examination in a multidisciplinary team it will be discussed.

**References**

1. Stovner LJ. Migraine prophylaxis with drugs influencing the renin- angiotensin system. Eur J Neurol. 2007;14(7):713-4. doi:10.1111/j.1468- 1331.2007.01760.x.

2. Ashina S, Bendtsen L, Lyngberg AC, Lipton RB, Hajiyeva N, Jensen R. Prevalence of neck pain in migraine and tension-type headache: a population study. Cephalalgia. 2015;35(3):211-9. doi:10.1177/0333102414535110.

3. Tomaz-Morais JF, Lucena LB, Mota IA, Pereira AK, Lucena BT, Castro RD, Alves GA. Temporomandibular disorders is more prevalent among patients with primary headaches in a tertiary outpatient clinic. Arq Neuropsiquiatr. 2015 Nov;73(11):913-7. doi: 10.1590/0004-282X20150145.

4. Prakash S, Rathore C. Side-locked headache: an algorithm based approach. The Journal of Headache and Pain 2016; 17:95 doi:10.1186/s10194-016-0687-9

6. Luedtke K, Boissonnault W, Caspersen N, Castien R, Chaibi A, Falla D et al. International consensus on the most useful physical examination tests used by physiotherapists for patients with headache: A Delphi study. Man Ther. 2016;23:17-24. doi:10.1016/j.math.2016.02.010.

7. Luedtke K, Starke W, May A. Musculoskeletal dysfunction in migraine patients. Cephalalgia. 2017; Jan 1:333102417716934. doi: 10.1177/0333102417716934.

8. Headache Classification Committee of the International Headache Society (IHS). The International Classification of Headache Disorders, 3rd edition(beta version) 2013 Jul;33(9):629-808. doi: 10.1177/0333102413485658.

9. Schiffman E, Ohrbach R, Truelove E, et al. Diagnostic Criteria for Temporomandibular Disorders (DC/TMD) for Clinical and Research Applications: Recommendations of the International RDC/TMD Consortium Network and Orofacial Pain Special Interest Group. Journal of oral & facial pain and headache. 2014;28(1):6-27.

### S43 Pediatric Hedache

#### Massimiliano Valeriani (m.valeriani@tiscali.it)

##### Centro per lo Studio e la Cura delle Cefalee in Età Evolutiva, UO Neurologia, Ospedale Pediatrico Bambino Gesù, Rome, Italy; Center for Sensory-Motor Interaction, Aalborg University, Aalborg, Denmark

Headache represents the most common neurological symptom in pediatric age. Among the primary headaches, migraine is far more prevalent than tension-type headache and cluster headache. Though extremely rare at this age, also trigeminal autonomic cephalgias have been reported. The most frequent causes of pediatric secondary headaches are represented by respiratory tract infections, while potentially life-threatening diseases, such as brain tumors, are less common. However, especially in the emergency setting, the possibility that a headache attack is due to a brain tumor must be always considered. To avoid missing these cases, some headache characteristics (red flags) have been identified [1]. In pediatric age, as in adulthood, the diagnosis of primary headache is based on the International Classification of Headache Disorders III-beta version (ICHD-IIIb). However, while the most recent ICHD criteria improved the possibility to classify some patients, such as children with migraine with aura [2], they turned out to be unsuitable for others, such as young patients with primary headache. In particular, we have showed that more than 50% of children up to 6 years of age have a headache duration shorter than 30 minutes, thus they could not be classified either in migraine or in tension-type headache [3]. Several studies have shown the primary role played by psychological factors in determining the severity of migraine in children [4]. Therefore, a psychological examination is often mandatory, as part of the initial assessment of the patient. Lastly, when assessing a child with primary headache, possible comorbidities should be never forgotten, since addressing them can represent a crucial point for the treatment [5].

**References**

1. Papetti L, Capuano A, Tarantino S, Vigevano F, Valeriani M. Headache as an emergency in children and adolescents. Curr Pain Headache Rep 2015;19:3.

2. Balestri M, Papetti L, Maiorani D, Capuano A, Tarantino S, Battan B, Vigevano F, Valeriani M. Features of aura in paediatric migraine diagnosed using the and ICHD 3III beta criteria. Cephalalgia, submitted.

3. Torriero R, Capuano A, Mariani R, Frusciante R, Tarantino S, Papetti L, Vigevano F, Valeriani M. Diagnosis of primary headache in children younger than 6 years: A clinical challenge. Cephalalgia 2017;37:947-954.

4. Özge A, Yalin OÖ. Chronic Migraine in Children and Adolescents. Curr Pain Headache Rep 2016;20:14.

5. Guidetti V, Arruda M, Özge A. Headache and comorbidities in childhood and adolescence. Springer, 2017

### S44 Overuse of Headache Medications: from Neurochemistry to Pathophysiology. Clinical and Preclinical Clues

#### Alberto Chiarugi

##### Department of Health Sciences, Section of Clinical Pharmacology and Oncology, University of Florence, and Headache Center University Hospital, Italy

Whether medication-overuse headache (MOH) represents a distinct biological entity within the concept of chronic daily headache with specific neurobiological and genetic background is still a matter of debate. A great deal of interest has been directed at understanding the neurophysiological mechanisms that underlie MOH pathogenesis. Currently, two main, non-mutually exclusive hypotheses have been proposed. The first, stems from the apparent compulsive use of headache medications by MOH patients, and considers this disorder a sort of addiction to symptomatic remedies. The second shifts the focus from drug addiction to neural sensitization, claiming that triptan overuse triggers adaptations of the trigeminovascular system, thereby facilitating pain transmission and leading to a state of latent sensitization. However, whether the effects of triptans are shared by all symptomatic headache medications as a sort of signature of the complex neurophysiologic rearrangements occurring in MOH is still unknown, as well as the extent of neurotransmitter/neuromodulators involved. Answering these questions might be relevant to better understand the neurochemical mechanisms prompted by acute headache medications that underlie the pathophysiology of MOH and of chronic headache in general.

In this presentation, preclinical data will be presented showing that chronic exposure to eletriptan or indomethacin alter trigeminal ganglion gene expression patterns broadly and to a similar extend. Remarkably, qualitative transcriptomic analysis reveals that prolonged exposure to the two different symptomatic drugs triggers almost identical, increased expression of various genes coding for proteins involved in headache pathogenesis such as neuropeptides, their cognate receptors, TRP channels, prostanoid and NO synthesizing enzymes. These findings will be correlated with the clinical aspects of MOH.

### S45 The very low calorie ketogenic diet in the clinical practice

#### Roberta Ienca, Simona Sodano

##### ^1^Department of Experimental Medicine, Medical Pathophysiology, food science and endocrinology section, Sapienza University, Rome, 00100, Italy

###### **Correspondence:** Roberta Ienca (roberta.ienca@libero.it)

**Background**

The very low calorie diet (VLCD) is a dietetic regimen characterized by a daily energetic intake < 800 Kcal/day, also called “simulated fasting”. The dramatic caloric restriction promotes the fat metabolism, mimicking the starvation, even if meals replacements ad hoc developed accounts for essential nutrients, avoiding the malnutrition. Because of the extreme caloric restriction, this type of diet is very effective in weight loss, however, that characteristic also is the main limit of VLCD, since it is possible to follow this kind of dietetic regimen for a very limited period (usually 3-12 weeks).

**Materials and methods**

According to macronutrients intake, it is possible to divide all the VLCDs in two sub-groups: ketogenic (VLCKD) and non-ketogenic diets. The VLCKD is characterized by low-carbohydrate (about 30 g/day carbohydrates), low-fat (fixed 15 g lipids), and slightly high-protein (1.0–1.4 g/kg of ideal body weight), divided in four daily meals consisting of dietary products developed ad hoc and nutraceutical integrators (Table 1). Salads are allowed ad libitum dressed with a spoonful of olive oil. The non-ketogenic VLCD is characterized by a slightly limited carbohydrate intake (≅100 g/day carbohydrates), low-fat (≅10 g lipids), and normal-protein (≅0.8 g/kg of ideal body weight). Also in this kind of diet, there are 4-5 meals per day, mainly consisting in meal replacement products. Both in VLCD and VLCKD lipids are usually provided by olive oil or omega-3.

**Results**

There is a growing interest in the ketogenic form of the VLCD because several studies have shown a higher compliance of patients with this diet. The reason of this higher adherence to the diet is still under scrutiny but several reasons are called in cause: an appetite suppression induced by proteins and (maybe) by ketone bodies (KBs), or a modification in hormone secretion (insulin, glucagon, ghrelin, adipokines).

**Conclusion**

The real impact of ketogenic diets in weight loss is still disputed; in fact, on the long period there are not differences between low-carb and low-fat diets in terms of weight reduction and regain of lost weight after the diet. However, thanks to the higher compliance and the drastic caloric restriction, the VLCKDs seem to be a promising approach in the early management of obesity and in the preparation phase for patients that must undergo to bariatric or other types of surgical procedures.

**Table 1 (abstract S45).** See text for description

	Daily dose	RDA
Vit A	800 mcg	100% RDA
Vit B1	1.4 mg	100% RDA
Vit B2	1.6 mg	100% RDA
Vit B3	18 mg	100% RDA
Vit B5	6 mg	100% RDA
Vit B6	2 mg	100% RDA
Vit B8	150 mcg	100% RDA
Vit B9	200 mcg	100% RDA
Vit B12	1 mcg	100% RDA
Vit C	60 mg	100% RDA
Vit D	5 mcg	100% RDA
Vit E	10 mg	100% RDA
Ca	800 mg	100% RDA
Cr	7.5 mcg	15% RDA
Cu	0.6 mg	50% RDA
Mg	90 mg	30% RDA
Mn	1.75 mg	RDA 1-10 mg
Zn	7.5 mg	50% RDA

### S46 Headache and temporomandibular disorders: association or causality? The bio-psychosocial model applied to the clinical reasoning

#### Marco Testa

##### Department of Neuroscience, Rehabilitation, Ophthalmology, Genetics, Maternal and Child Health, University of Genova – Campus of Savona, 17100 Savona - Italy

Temporomandibular disorders (TMD) represent the main cause of orofacial pain of non-dental origin and comprehend several disturbances of the masticatory system characterized by myofascial pain of masticatory muscles or articular pain localized in the pre-auricular area. Moreover, TMD patients show temporomandibular joint sounds and deviation or limitation of the opening of the mouth.

Myofascial pain is a probable consequence of central nervous system mechanisms of convergence and activation of second order neurons with enlargement of the receptive field, reduced pain threshold and allodinia. Often there are accompanying symptoms like facial pain and headaches.

Headache is the most prevalent neurologic disorder, third most diffused health disturbance and the seventh cause of disability in the world. It can be primary, without apparent organic cause, or secondary to other pathologies.

Some epidemiological studies indicates that headache is more prevalent in TMD patients and TMD is more prevalent in subjects affected by headache. A stronger association exists between TMD and chronic migraine in comparison with other types of headache. Myofascial TMD is more frequently associated to headache than articular TMD. Nevertheless the methodological quality of the available studies is not optimal and many of them classify patients with anamnestic questionnaire that tend to overestimate the values of prevalence.

A growing body of literature suggests that the association between headache and TMD may be a manifestation of a central sensitization mechanism. Temporomandibular joint and muscles receive the sensitive innervation of the trigeminal nerve that supply also the cranial vascular structures likely involved in the etiology of the headache. The sensitization of the trigeminal caudate nucleus by the TMD symptoms can favor the triggering of migraine episode.

Beside the epidemiological studies and the neurophysiologic hypothesis, there are some initial clinical evidence that show how severity of TMD symptoms parallels an increase of frequency and intensity of migraine and the simultaneous treatment of both conditions results in better outcomes.

From a clinical perspective, a comprehensive assessment based on a biopsychosocial approach can provide relevant information to plan a contemporaneous treatment of TMD and headache, together with an intervention targeted to the reduction of psychosocial conditions that can elicit and maintain mechanisms of central sensitization likely responsible of the comorbidity of TMD and headache.

### S47 Tension-Type Headache and Central Sensitization: the Role of Physical Therapy According to EBM

#### Matteo Castaldo^1,2,3^ (matteo.castaldo@poliambulatoriofisiocenter.com)

##### ^1^Department of Health Science and Technology. Aalborg University, Aalborg, Denmark; ^2^Siena University, Siena, Italy; ^3^Poliambulatorio Fisiocenter, private practice, Parma, Italy

Tension-type headache (TTH) is the most common headache, with a lifetime prevalence ranging between 30% and 78% in the general population, and with a high socio-economic impact [1].

The exact pathophysiology is still unknown, but evidence supporting both peripheral and central mechanisms (i.e. central sensitization) is increasing [2,3].

In fact, the frequency of headache attacks has found to be related to the level of central sensitization [4].

However, not all TTH patients present with the same level of central sensitization and clinical presentation, but subgroups need to be identified in order to offer specific therapeutic programs [5].

Prolonged peripheral nociceptive input from the pericranial, neck, and shoulder regions (e.g. trigger points (TrPs), zygoapophyseal joints) may over time sensitize the central nervous system, transmitting nociceptive input to the trigemino-cervical nucleus caudalis [6].

In fact, it has been found that sustained stimulation of TrPs may induce central sensitization in healthy participants [7].

There is evidence supporting the role of TrPs as contributor to TTH, and that the referred pain elicited by TrPs stimulation reproduces the headache pattern in TTH patients [8].

The number of TrPs seems to be associated with the degree of widespread pressure pain hypersensitivity in TTH patients, supporting the role of TrPs on central sensitization: however the cross-sectional nature of the study does not allow to establish a cause and effect relationship between TrPs and central sensitization, as other variables may influence this association [9].

Physical therapy may be helpful for the management of TTH patients [10,11], as it may decrese the peripheral nociceptive input.

However, to nowdays, studies on treatment of TrPs in TTH are still few and more evidence is needed.

**References**

1. Stovner L, Hagen K, Jensen R, et al. The global burden of headache: a documentation of headache prevalence and disability worldwide. Cephalalgia 2007;27:193–210.

2. De Tommaso M and Fernández-de-Las-Peñas C. Tension type headache. Curr Rheumatol Rev 2016; 12: 127–139.

3. Andersen S, Petersen MW, Svendsen AS, et al. Pressure pain thresholds assessed over temporalis, masseter, and frontalis muscles in healthy individuals, patients with tension- type headache, and those with migraine: A systematic review. *Pain* 2015; 156: 1409–1423

4. Buchgreitz L, Lyngberg AC, Bendtsen L, et al. Frequency of headache is related to sensitization: a population study. Pain 2006; 123(1-2):19-27.

5. Fernández-de-Las-Peñas C, Benito-González E, Palacios-Ceña M, et al. Identification of subgroups of patients with tension type headache with higher widespread pressure pain hyperalgesia. J Headache Pain 2017; 18(1):43.

6. Bendtsen L, Fernandez-de-Las-Penas C. The role of muscles in tension-type headache. Curr Pain Headache Rep. 2011;15:451–458.

7. Xu YM, Ge HY, Arendt-Nielsen L. Sustained nociceptive mechanical stimulation of latent myofascial trigger point induces central sensitization in healthy subjects. J Pain. 2010;11:1348–1355.

8. Fernández-de-Las-Peñas C, Cuadrado ML, Arendt-Nielsen L, et al. Myofascial trigger points and sensitization: An updated pain model for tension-type headache. Cephalalgia 2007; 27: 383–393.

9. Palacios-Ceña M, Wang K, Castaldo M, et al. Trigger Points are associated with widespread pressure pain sensitivity in people with tension-type headache. Cephalalgia 2016 [Epub ahead of print].

10. Alonso-Blanco C, de-la-Llave-Rincón AI, Fernández-de-las-Peñas C. Muscle trigger point therapy in tension-type headache. Expert Rev Neurother 2012; 12(3):315-22.

11. Espí-López GV, Arnal-Gómez A, Arbós-Berenguer T,et al. Effectiveneess of physical therapy in patients with tension-type headache: literature review. J Jpn Phys Ther Assoc 2014; 17(1):31-38.

### S48 The use of nutraceuticals in migraine

#### Cherubino Di Lorenzo (cherub@inwind.it)

##### Don Carlo Gnocchi Onlus Foundation, Milan, Italy

**Background**

Migraine is related to the highest disability among headaches. Great efforts are faced to improve the outcome of forthcoming treatments. However, still now, many patients regard as unsatisfactory the low responder rate (about the half of patients) and adverse effects that current treatments account. Therefore, waiting for innovative, more tolerated and effective treatments, there is a large request for non-pharmacological approaches that in many cases have specific pathophysiological targets. Among these treatments, nutraceuticals has a leading role. The term “nutraceuticals” indicates any product derived from food or herbal sources, administrated at a specific dose, adequate to obtain some health benefits. Several nutraceutical products are proposed for migraine and sold around the world, but researchers adequately study only few compounds

**Methods**

Among studied nutraceuticals compounds, only few of them have studies of good quality in support. Moreover, also interactions among different molecules are not studied. We have reviewed literature data in order to find researches that support the use of nutraceutical molecules in migraine management.

**Results**

Available good quality data support the use of certain nutraceuticals, in particular riboflavin, coenzyme Q10, magnesium, butterbur, feverfew, and omega-3 polyunsaturated fatty acids. Even if not supported by double blind studies, recently some prospective observational studies about fixed combination of nutraceuticals were performed. For instance, it is the case of a combination of coenzyme Q10, feverfew and magnesium for migraine prophylaxis: a prospective observational study. A double blind versus placebo study about the effect of a fixed combination of riboflavin, coenzyme Q10, feverfew, andrographis and magnesium for migraine prophylaxis is currently in progress.

**Conclusions**

Usually patients appreciate nutraceuticals more than traditional drugs, since they are regarded as safe and of efficacy not inferior to other pharmacological products. Available data seem to support this widespread belief, but some concerns about the regulation of nutraceuticals and quality of some products, still remain.

### S49 Headaches and Algology (Pain Therapy)

#### M. Evangelista (maurizio.evangelista@unicatt.it)

##### Istituto di Anestesiologia, Rianimazione e Terapia del Dolore, Università Cattolica del Sacro Cuore, Roma, Italia

Contrary to what is generally thought of, headaches and algology (pain therapy) share many aspects.The first, and perhaps most important, aspect of sharing between headaches and chronic pain is the fact that both cause a negative impact on “quoad valetudinem” rather than “quoad vitam” [1]; in both cases it is possible to talk about under-sized and under-diagnosed diseases as well as primary health illnesses for the nation's health policy [2].Headaches and chronic non-oncological pain are two paradigms of chronic illness capable of generating enormous individual and social impact by disabling the sick person not only in the biological, but also in the psychological, professional, social and relational spheres. Both cause alterations in psychological equilibrium, secondary depression, loss of social and professional roles, which, in the most serious cases, can cause loss of work. Literature documents in both cases, headaches and chronic pain, a rise in direct costs but above all of the indirect ones with a huge burden of disease. Both are capable of generating a marked drop in the quality of life associated with a serious bio-psycho-social disability. Headaches and chronic pain, although distinct according to a topographical criterion, share many mechanisms and physiopathogenetic steps. One of the most current fields in which neurologists and pain therapists converge is the focus on neuroinflammation [3] and central sensitization[4], two key mechanism for triggering, maintaining, and subsequent perpetuation of pain: the pain as a symptom, filogenetically responsible for maintaining homeostasis of the organism against actual or potential damage, becomes unnecessary illness without any protective meaning. Another important shared pathogenetic passage is that of neuroimmune mechanisms, which interlink the immune system with the central nervous system[4]. Furthermore, numerous contribution to the scientific international literature highlight the need to modify the therapeutic approach, directing it towards a semeiotic criterion (pain phenothype: specific sign and symptoms of a certain type of pain in a specific moment), which is an epiphenomenon of underlyng pathogenetic mechanism, instead of basing it on a etiologic criterion[5]. This would enable a more appropriate prescription and greater efficiency, taking into primary consideration the possibility of getting back to everyday life rather than obtaining complete analgesia. In both cases, headaches and chronic pain, a therapeutic protocol should be effective as well as sustainable in terms of both biologic aspect (effectiveness/safety ratio) and acceptability (minimum interference with professional, relational and social life). All the above mentioned aspects are equally important but one of them can prevail over the others depending on patient characteristics and background. From that derives another shared aspect: the concept of personalized “dynamic” therapy, where the physician (neurologist or pain physician), once identified realistic objectives that the patient wants to achieve, has to define the best possible protocol basing on his expertise and on the avalaible treatments, as well as periodically re-evaluate the clinical trend in order to provide modifications or integrations to the therapy, if necessary [5]. In conclusion it can be stated that the aspects of sharing between headaches and chronic non-oncological pain are significantly greater than those that clearly divide them. this must therefore be an area where researchers’ efforts must converge to achieve the primary goal of recovering pain-related disability.

**References**

1. World Health Organization. International classification of functioning, disability and health (ICF). Geneva, World Health Organization, 2001

2. Steiner T.J Lifting the burden: The global campaign against headache. (2004) *Lancet Neurology*, 3 (4), pp. 204-205

3. Ru-Rong Ji Emerging targets in neuroinflammation-driven chronic pain. Nat Rev Drug Discov. 2014 Jul; 13(7)

4. Baron R Neuropathic pain: diagnosis, pathophysiological mechanisms, and treatment. Lancet Neurol. 2010 Aug;9(8):807-19. doi: 10.1016/S1474-4422(10)70143-5

5. Edwards RR Patient phenotyping in clinical trials of chronic pain treatments: IMMPACT recommendations. Pain. 2016 Sep;157(9):1851-71.

### S50 Neuroimaging and headaches

#### Paola Sarchielli, Laura Bernetti

##### Headache Center, Neurologic Clinic, Ospedale Santa Maria della Misericordia, University of Perugia Perugia Italy

Headache is a common clinical feature in neurological patients .Usually, neuroimaging is unnecessary in patients with episodic migraine or tension type headache with typical headache features and with a normal neurological examination. These patients do not have a higher probability of a relevant brain pathology compared to the general population.

A recent study, however, reported that neuroimaging is routinely ordered in outpatient headache even if guidelines specifically recommend against their use. In the same study, after 5 years, a patient with a new migraine has a 40% chance of receiving a neuroimaging examination[1].

Brain MRI with detailed study of the pituitary area and cavernous sinus, is recommended for all trigeminal autonomic cephalalgias TACs. Sometimes additional scanning of intracranial/cervical vasculature and/or the sellar/orbital/(para)nasal region are needed to exclude underlying pathological conditions [2].

Neuroimaging should be considered in patients presenting with atypical headache features, a new onset headache, change in previously headache pattern, headache abruptly reaching the peak level, headache changing with posture, headache awakening the patient, or precipitated by physical activity or Valsalva manoeuvre and abnormal neurological examination. Other condition for which MRI is recommended are: first onset of headache ≥50 years of age, trauma, fever, seizures, history of malignancy, history of HIV or active infections, and prior history of stroke or intracranial bleeding [2, 3].

A recent consensus recommends brain MRI for the case of migraine with aura that persists on one side or in brainstem aura. Persistent aura without infarction and migrainous infarction also require brain MRI, MRA and MRV. According the same consensus, fFor primary cough headache, exercise headache, headache associated with sexual activity, thunderclap headache and hypnic headache apart from brain MRI additional tests may be required [3].

Particularly in emergency room it is mandatory to exclude a secondary headache that requires special attention and further diagnostic workup. A careful patient history should be collected and additional ‘red flags’ should be detected at the physical examination to identify patients which can benefit of a MRI or CT scan to detect significant brain pathology. and make a correct diagnosis and receive an adequate and prompt therapeutic intervention.

CT scan is the first line neuroimaging examination. MRI offers a greater resolution and discrimination and might therefore be the preferred method of choice in non acute headache. In addition, radiation due to CT scanning may be avoided

Neuroimaging non conventional techniques are of little or no value in the clinical setting .but may contribute greatly to increasing understanding of the pathogenesis of primary headaches.

**References**

1. Callaghan BC, Kerber KA, Pace RJ, Skolarus L,Cooper W, Burke JF.Headache neuroimaging: Routine testing when guidelines recommend against them. Cephalalgia. 2015 Nov;35; 1144-52.

2. Sandrini G, Friberg L, Coppola G, Janig W, Jensen R, Kruit M, et al. europhysiological tests and neuroimaging procedures in non-acute headache (2nd edition) Eur J Neurol. 2011;18(3):373–81.

3. Mitsikostas DD, Ashina M, Craven A, Diener HC, Goadsby PJ, Ferrari MD et al.; EHF committee. European Headache Federation consensus on technical investigation for primary headachedisorders. J Headache Pain. 2015;17:5.

### S51 Risk factors for chronic migraine

#### Cristina Tassorelli^1,2^, Grazia Sances^1^, Sara Bottiroli^1^, Michele Viana^1^, Natascia Ghiotto^1^, Marta Allena^1^, Giorgio Sandrini^1,2^, Giuseppe Nappi^1^

##### ^1^Headache Science Center (HSC), C. Mondino National Institute of Neurology Foundation, Pavia, Italy; ^2^Dept of Brain and Behavioural Sciences, University of Pavia, Italy

###### **Correspondence:** Cristina Tassorelli (cristina.tassorelli@unipv.it)

Migraine frequency fluctuates over time. Chronic migraine is defined by the occurrence of headache on more than 15 days/month for at least 3 months. Chronic migraine affects 1–2% of the general population, and about 8% of patients with migraine, with an annual conversion rate of about 3%. In the literature, the most important recognized factors associated to chronic migraine are overuse of acute migraine medication, ineffective acute treatment, obesity, depression, presence of allodynia and stressful life events. These latter seem in particular relevant not only incidentally, as a precipitating/aggravating factor in the short term, but, most importantly and interestingly, as potential epigenetic modulators of disease severity over time. Other factors reported in studies are age, female sex and low educational status. Very recently, a large population study suggested that the presence of additional noncephalic pain site is a risk factor for migraine chronification.

For many of these factors the relationship with migraine chronification may however be bi-directional. For instance, in the case of depression, it is possible that depression may negatively affect the response of migraine to acute and prophylactic treatments, but it is also true the opposite: i.e. the recurrence of disabling headache attacks may alter the psychological well-being of a normothymic subject, thus leading to mood alterations. In the case of obesity, the association with chronic migraine may simply be ascribed to the effect of fat tissue in drug distribution.

Beside and beyond the putative biological factors that may cause a worsening of disease, several lines of evidence suggest that the progression from episodic to chronic migraine is associated to a progressive increase and stabilization of functional and anatomical changes associated to chronic sensitization. In this frame, it appears obvious that an additional cause for chronic migraine is quite likely represented by the low rate of prescription of preventive medications. According to the American Migraine Prevalence and Prevention Study, nearly 40% of migraine sufferers should be considered for preventive migraine therapy, while only 13% of all patients with migraine currently use preventive therapy to control their attacks. The underutilization of preventive drugs has several explanations ranging from drug-associated issues (limited efficacy, poor tolerability profile) to education of practitioners, pharmacists and patients, and it also involve the limited access to qualified care. Underutilization of preventative drugs also translate into a higher recourse to acute drugs, thus feeding on a vicious cycle that leads to negative consequences.

**Conflicts of interests**

CT has participated in advisory boards for Allergan and electroCore; she has lectured at symposia sponsored by Allergan; she is PI or collaborator in clinical trials sponsored by Alder, electroCore, Eli-Lilly and Teva. She has received grants from the European Commission, the Italian Ministry of Health and the Italian Ministry of University

**References**

Scher AI, Buse DC, Fanning KM, Kelly AM, Franznick DA, Adams AM, Lipton RB. Comorbid pain and migraine chronicity: The Chronic Migraine Epidemiology and Outcomes Study. Neurology. 2017 Aug 1;89(5):461-468. 1.

Silberstein SD, Diamond S, Loder E, et al. Prevalence of migraine sufferers who are candidates for preventive therapy: results from the American migraine study (AMPP) study. Headache 2005; 45: 770– 771.

Tassorelli C, Jensen R, Allena M, De Icco R, Katsarava Z, Miguel Lainez J, Leston JA, Fadic R, Spadafora S, Pagani M, Nappi G; COMOESTAS Consortium. The added value of an electronic monitoring and alerting system in the management of medication-overuse headache: A controlled multicentre study. Cephalalgia. 2016 [Epub ahead of print]

### S52 Comorbidities in primary headaches

#### Antonio Carolei^1,2^, Cindy Tiseo^1^, Diana Degan^1^

##### ^1^ Institute of Neurology, Department of Applied Clinical Sciences and Biotechnology, University of L’Aquila, via Vetoio, 67100 L’Aquila, Italy; 2 Department of Neurology and Stroke Unit, Avezzano Hospital, 67051, Avezzano, Italy

###### **Correspondence:** Antonio Carolei (antonio.carolei@univaq.it)

According to the International Classification of Headache Disorders, 3^rd^ edition (beta version) [1], primary headaches are classified as “migraine”, “tension-type headache”, “trigeminal autonomic cephalalgia”, and “other primary headache disorders”. To date, the majority of clinical studies concerning primary headaches and their comorbidities are focused on migraine. Comorbidities of migraine may include neurological and psychiatric conditions, as mood disorders (depression, mania, anxiety, panic attacks), epilepsy, essential tremor, stroke, and the presence of white matter abnormalities [2]. Particularly, a complex and bidirectional relation between migraine and stroke has been described, including migraine as a risk factor for cerebral ischemia, migraine caused by cerebral ischemia, migraine mimicking cerebral ischemia, migraine and cerebral ischemia sharing a common cause, and migraine associated with subclinical vascular brain lesions [2]. A recent meta-analysis pointed out that migraine is associated with increased ischemic stroke risk [3], and according to a systematic review and meta-analysis [4] the risk of hemorrhagic stroke in migraineurs is increased with respect to non-migraineurs. Besides, the risk of transient ischemic attack seems to be increased in migraineurs, although this issue has not been extensively investigated [5]. A recent systematic review and meta-analysis also describes an increased risk of myocardial infarction and angina in migraineurs compared to non-migraineurs [6]. Concerning the association between migraine and vascular risk factors (arterial hypertension, diabetes mellitus, dyslipidemia, obesity, alcohol consumption, family history of cardiovascular disease), a recent review [7] showed no solid evidence of an increased burden of conventional vascular risk factors in migraineurs, with the only exceptions of dyslipidemia and cigarette smoking, while a systematic review and meta-analysis regarding migraine and body mass index categories [8] found an increased risk of having migraine in underweight subjects and in obese women as compared with normal-weight subjects. Few studies investigated the comorbidities of tension-type headache (TTH), despite the fact that tension-type headache (TTH) is highly prevalent, and may be as debilitating as migraine [9]. It is noteworthy that, according to a review, TTH is associated with increased rate of affective distress [9]. Furthermore, some medical disorders may worsen a preexisting TTH, and it has been described the comorbidity of TTH with psychiatric disorders and fibromyalgia [10].

**References**

1. Headache Classification Committee of the International Headache Society (IHS). The International Classification of Headache Disorders, 3^rd^ edition (beta version). Cephalalgia. 2013; 33: 629–808.

2. Sacco S, Olivieri L, Bastianello S, Carolei A. Comorbid neuropathologies in migraine. J Headache Pain. 2006;7:222-230.

3. Spector JT, Kahn SR, Jones MR, Jayakumar M, Dalal D, Nazarian S. Migraine headache and ischemic stroke risk: an updated meta-analysis. Am J Med. 2010; 123:612-624.

4. Sacco S, Ornello R, Ripa P, Pistoia F, Carolei A. Migraine and hemorrhagic stroke: a meta-analysis. Stroke. 2013; 44:3032-3038.

5. Sacco S, Kurth T. Migraine and the risk for stroke and cardiovascular disease. Curr Cardiol Rep. 2014;16:524.

6. Sacco S, Ornello R, Ripa p, Tiseo C, Degan D, Pistoia F, Carolei A. Migraine and risk of ischaemic heart disease: a systematic review and meta-analysis of observational studies. Eur J Neurol. 2015; 22:1001-1011.

7. Sacco S, Degan D, Carolei A. Conventional vascular risk factors: Their role in the association between migraine and cardiovascular diseases. Cephalalgia. 2015; 35:146-164.

8. Ornello R, Ripa P, Pistoia F, Degan D, Tiseo C, Carolei A, Sacco S. Migraine and body mass index categories: a systematic review and meta-analysis of observational studies. J Headache Pain. 2015;16:27.

9. Heckman BD, Holroyd KA. Tension-type headache and psychiatric comorbidity. Curr Pain Headache Rep. 2006;10:439-447.

10. Sacco S, Ricci S, Carolei A. Tension-type headache and systemic medical disorders. Curr Pain Headache Rep. 2011;15:438-443.

### S53 Headeache in the Emergency Department

#### Vittorio Di Piero (vittorio.dipiero@uniroma1.it)

##### Department of Neurology and Psychiatry, “Sapienza” - University of Rome, Rome, Italy

Differentiating patients with life-threatening headaches from the overwhelming majority with primary headaches (eg migraine, tension or cluster headache) is an important issue in emergency department (ED). Patients with non-traumatic headaches are up to 4.5 per cent of adults looking for emergency visits (Torelli, 2010). Of these patients, only 20% had a secondary headache requiring diagnosis and hospitalization (Pari, 2015). On the other hand, 80% of these patients have a primary form, requiring evaluation and outpatient treatment. These numbers seem to remain constant in Western countries (Ramirez-Lassepas, 1997; Kowalski, 2004; Cvetkovic, 2007; Gaughran, 2014).

Primary headaches still pose an open challenge in the ED because the failure to recognize a secondary headache could cause potentially fatal consequences. Unfortunately, to date, there is still no a standard diagnostic procedure for headache in emergency conditions; although according to the diagnostic guidelines there are red flags that could help in the process, the positive predictive value of each severity indicator is not yet determined.

The problem of poor diagnostic sensitivity was attributed to IHCD-3 criteria rigidity in relation to primary headache diagnosis in emergency setting (Dutto, 2009, Swadron, 2010). Attempting to overcome the primary headache diagnostic problem in ED, the Canadian Emergency Association proposed simplified IHS criteria to be easily implemented in the ED environment (Ducharme, 1999). Alternatively, a different standardized work-up has been proposed for the most frequent headache scenarios in ED (Cortelli, 2004; Dutto, 2009).

A careful history and physical examination remain the most important part of the assessment of the headache patient; they enable the clinician to determine whether the patient is at significant risk for a dangerous cause of their symptoms and what additional workup is necessary. This presentation will discuss how to approach adults with headache in ED with an emphasis on those features that characterize high-risk headaches.

### S54 Migraine without aura, arthrogenic and myofascial cervical afferents: role of EBM physiotherapy

#### Firas Mourad^1,2,3^(firas.mourad@me.com)

##### ^1^“Tor Vergata” Roma University, Roma, Italy; ^2^Alumno de Doctorado, Escuela Internacional de Doctorado, Universidad Rey Juan Carlos, Alcorcon, Madrid, Spain; ^3^PHYSIOPOWER, viale Duca degli Abruzzi 107, Brescia, 25124, Italy

Headaches are one of the most disabling disorders [1]. That is, 50% of general population suffer from headache (HA) during any given year; moreover, 90% report a lifetime history of HA [1, 2].

Migraine is one of the most common type of headache with an estimated prevalence of 10% [3] of the general population.

The International Headache Society (IHS) classify Migraine as a primary headache. That is, the 3rd edition of the International Classification of Headache Disorders (ICHD-III) describes also the diagnostic criteria of each headache disorder types. Interestingly, Migraine and Cervicogenic Headache (CGH) share similarities in these criteria and clinical presentation. Moreover, Neck Pain associated disorders (NAD) is a very common presentation in Migraine population [4]. Thus, the muscolokeletal contribution in Primary Headaches is still debate in the literature [5]. Moreover, recent knowledge suggests that different clinical headache phenotypes arising from a common pathophysiology rather than an independent disorder [6]. That is, in the most prevalent headaches disorders (i.e. TTH, Migraine, CGH) the ascending pathway of trigeminovascular system and Trigemino Cervical Nucleus (TCN) play a primary role in the head | face pain etiopathogenesis [7, 8]. In this presentation, the role of the musculoskeletal inputs in primary headaches it will be provided. Moreover, evidences of the effectiveness of a manual therapy management provided by a physiotherapist and its integration in a multidisciplinary team it will be discussed.

**References**

1. Stovner LJ. Migraine prophylaxis with drugs influencing the renin-angiotensin system. Eur J Neurol. 2007;14(7):713-4. doi:10.1111/j.1468-1331.2007.01760.x.

2. Steiner TJ, Stovner LJ, Katsarava Z, Lainez JM, Lampl C, Lanteri-Minet M et al. The impact of headache in Europe: principal results of the Eurolight project. J Headache Pain. 2014;15:31. doi:10.1186/1129-2377-15-31.

3. Pietrobon D, Striessnig J. Neurobiology of migraine. Nat Rev Neurosci. 2003;4(5):386-98. doi:10.1038/nrn1102.

4. Ashina S, Bendtsen L, Lyngberg AC, Lipton RB, Hajiyeva N, Jensen R. Prevalence of neck pain in migraine and tension-type headache: a population study. Cephalalgia. 2015;35(3):211-9. doi:10.1177/0333102414535110.

5. Luedtke K, Boissonnault W, Caspersen N, Castien R, Chaibi A, Falla D et al. International consensus on the most useful physical examination tests used by physiotherapists for patients with headache: A Delphi study. Man Ther. 2016;23:17-24. doi:10.1016/j.math.2016.02.010.

6. Cady RK. The convergence hypothesis. Headache. 2007;47 Suppl 1:S44-51. doi:10.1111/j.1526- 4610.2007.00676.x.

7. Noseda R, Burstein R. Migraine pathophysiology: anatomy of the trigeminovascular pathway and associated neurological symptoms, CSD, sensitization and modulation of pain. Pain. 2013;154 Suppl 1. doi:10.1016/j.pain.2013.07.021.

8. Noseda R, Burstein R. Migraine pathophysiology: anatomy of the trigeminovascular pathway and associated neurological symptoms, cortical spreading depression, sensitization, and modulation of pain. Pain. 2013;154 Suppl 1:S44-53. doi:10.1016/j.pain.2013.07.021.

### S55 OnabotulinumtoxinA for migraine treatment

#### Andrea Negro^1,2^ (andrea.negro@uniroma1.it)

##### ^1^Regional Referral Headache Centre, Sant'Andrea Hospital, Via di Grottarossa 1035-1039, 00191; ^2^Department of Clinical and Molecular Medicine, Sapienza University of Rome, Italy

Since 2010 the armamentarium of preventative drugs for chronic migraine (CM) has become wider with the introduction of OnabotulinumtoxinA (Botox®). The European Headache Federation recognized the value of OnabotulinumtoxinA suggesting that, before labeling a patient as affected by refractory CM, a proper treatment with this drug needs to be completed [1]. In the last years several real-life prospective studies provided further evidence in clinical setting of OnabotulinumtoxinA 155-195 U efficacy for the headache prophylaxis in CM complicated by medication overuse headache (MOH) [2]. Recently we published the results of a prospective study on the long-term (2 years) efficacy and safety of a single dose of OnabotulinumtoxinA (155 or 195 U) in patients with CM plus MOH had failed previous preventative drugs and detoxification attempts [3]. Both the doses were effective and equally safe, but 195 U was more effective than 155 U in reducing headache days, migraine days, pain medication intake days and Headache Impact Test (HIT)-6 score. Even more, the 195 U dose superior efficacy was evident since the first injection and maintained over all the study period of 24 months. Interestingly we observed a progressive improvement in all the efficacy measures during the 2 years of follow-up with both the doses and significantly more with 195 U. Sometime a response appears only after the second or third injections. For this reason in selected cases can be useful to temporarily continue an oral preventative agent. CM patients close to developing MOH, patients with high frequency episodic migraine (HFEM), and those already abusing of drugs require special attention and maybe an be an “early treatment”. The NICE guidelines recommend OnabotulinumtoxinA only for patients who have already tried at least three different preventative drug treatments that have not worked. The chance to use it as first-line preventative treatment may shorten the period of chronicity and eventually prevent the developing of MOH. Another early use of OnabotulinumtoxinA could be in patients with HFEM. Several studies conducted before OnabotulinumtoxinA approval shown that it is ineffective in patients with episodic migraine [4]. Those studies had important limitations as range doses and injection paradigm. Furthermore, the population enrolled was represented in the majority by patients with low frequency episodic migraine (an average of 6-7 attacks per month). Interestingly post hoc analyses including episodic migraine patients with a greater mean frequency of headache days (≥12 and ≤15) suggested that they have a more robust response to OnabotulinumtoxinA [4].

**References**

1. Martelletti P, Katsarava Z, Lampl C, Magis D, Bendtsen L, Negro A, Russell MB, Mitsikostas DD, Jensen RH. Refractory chronic migraine: a consensus statement on clinical definition from the European headache federation. J Headache Pain 2014;28;15:47.

2. Negro A, Curto M, Lionetto L, Guerzoni S, Pini LA, Martelletti P. A critical evaluation on MOH current treatments. Curr Treat Options Neurol. 15;19(9):32.

3. Negro A, Curto M, Lionetto L, Martelletti P. A two years open-label prospective study of OnabotulinumtoxinA 195 U in medication overuse headache: a real-world experience. J Headache Pain 2015;17:1.

4. Shuhendler AJ, Lee S, Siu M, Ondovcik S, Lam K, Alabdullatif A, Zhang X, Machado M, Einarson TR. Efficacy of botulinum toxin type A for the prophylaxis of episodic migraine headaches: a meta-analysis of randomized, double-blind, placebo-controlled trials. Pharmacotherapy 2009;29:784–91.

### S56 Trigeminal autonomic cephalalgias (TACs)

#### Ferdinando Maggioni (ferdinando.maggioni@unipd.it)

##### Headache Centre, Department of Neurosciences, University of Padua, Italy

Trigeminal autonomic cephalalgias (TACs) are a group of primary headaches comprehending the following syndromes: episodic and chronic cluster headache (CH), episodic and chronic paroxysmal hemicrania (PH), short-lasting unilateral neuralgiform headache attacks, and hemicrania continua(HC) [1]. Their phenotypes are similar and attack duration is the main feature distinguishing the first three TACs. An accurate diagnosis is important because of their different response to treatments. Among TACs, CH is most common; however TACs are approximately at least 100 times less common than migraine. CH prevalence in adults is < 1% and interests specially the male population. CH typically occurs at the same time of the day, from once to eight times per day, and in the same period of the year. CH is featured by severe unilateral peri-orbital and/ or temporal pain lasting from 15 to 180 minutes if untreated, associated with at least one autonomic symptom (conjunctival injection, lacrimation, nasal congestion, rhinorrhea, facial sweating, miosis, ptosis and eyelid edema). Trigger factors can include alcohol, volatile chemicals or a warm environment (3). Acute therapy includes the use of oxygen at a rate of 12-15L/min for at least 15 minutes and triptans. Controlled trials have investigated the efficacy of subcutaneous sumatriptan, nasal sumatriptan, and nasal zolmitriptan. When a preventive medication is required, verapamil is the reference treatment. PH attack features are characterized by unilateral, often stabbing, headaches, shorter and more frequent than in cluster headaches. PH is responsive to treatment with indomethacin. Indomethacin dosages ranges from 25 to 75 mg, three times a day. SUNCT-SUNA attacks are very short in duration (seconds to minutes), triggered by touching the face or chewing, with associated autonomic features and occur up to hundreds of times per day. SUNCT-SUNA most often responds to lamotrigine, limited the evidences for topiramate, gabapentin, carbamazepine, duloxetine and oxcarbazepine. In HC, clinical attack features have been reported as unilateral, side-locked continuous pain (although interrupted by frequent severe exacerbations), associated with autonomic symptoms and responsive to indomethacin.

Therapeutic options in TACs are limited. In many patients the preventive treatment does not help to control attack frequency, or the acute drugs are not well tolerated or are contraindicated. For these reasons, after the discovery of the central role of the hypothalamus in TACs pathogenesis, neuromodulation techniques started. After the results obtained with hypothalamic deep brain stimulation in CH, other peripheral neuromodulation targets (occipital nerves, spinal cord, sphenopalatine ganglion, vagus nerve) were tried in the management of refractory CH and other TACs.

**References**

1. Headache Classification Committee of the International Headache Society: The international classification of headache disorders: 3th edition (beta version). Cephalalgia 2013, 33:629-808

2. Nesbitt AD, Goadsby PJ: Cluster Headache. Br Med J 2012 344:e2407.

3. Lainez M, Guillam_E. Cluster Headache and Other TACs: Pathophysiology and Neurostimulation Options. Headache 2017; 327-334.

### S57 Chronic Headaches Cefalee Croniche

#### Grazia Sances^1^, Sara Bottiroli^1^, Michele Viana^1^, Natascia Ghiotto^1^, Elena Guaschino^1^, Marta Allena^1^, Cristina Tassorelli^1-2^

##### ^1^Headache Science Center (HSC), C. Mondino National Institute of Neurology Foundation, Pavia, Italy; ^2^Dept of Brain and Behavioural Sciences, University of Pavia, Italy

###### **Correspondence:** Grazia Sances (grazia.sances@mondino.it)

Chronic headaches are a relevant health problem characterized by significant disability, poor quality of life and high economic burden (1). The most common forms include chronic migraine (CM) and medication overuse headache (MOH), which are frequently associated, given that the majority of CM sufferers do overuse acute medications (CM with MO). Chronic headaches represent a challenge for physicians, given their frequent resistance to therapies, risk of relapse and associated comorbidities. Their management includes several steps aimed to: 1) make a proper diagnosis excluding secondary forms; 2) identify exacerbating factors; 3) treat comorbidities; 4) identify and address medication overuse; 5) establish a therapeutic agreement with patient; 6) define an integrated care approach. Patient-history collection is crucial for defining headache onset and its life-long course, chronicization factors, and outcomes of previous therapies (acute and prophylactic).

Overused drug discontinuation is the first approach for MOH and it can be achieved via multiple modalities - in-patient or out-patient withdrawal procedures, advice alone – depending on several headache-associated or patient-associated factors. During withdrawal, adequate care is required to help the patient to go through the treatment phases, given the frequent occurrence of headache recrudescence.

Headache diaries represent useful tools in monitoring attacks frequency, detecting medication overuse, checking therapies outcomes, and assessing disability improvements. A relevant problem in MOH is the risk of relapse into overuse after successful withdrawal. There are only few controlled pharmacological trials on the management of MO in CM, which does not allow to derive precise figures on the risk of relapse into MO associated to specific therapies. Furthermore, the relapse risk is also influenced by psychological and clinical comorbidities. For instance, mood and personality disorders (e.g., anxiety, depression, obsessive-compulsive personality, or addictive behavior) are very common in patients with CM with MO. Psychological factors indeed seem to play a crucial role in predicting the outcome (e.g., reduction in headache attacks and medication intake or relapse into overuse) of detoxification and should be considered when treating these patients (2,3). The presence of other pain-syndrome comorbidities (e.g., fibromyalgia) can further adversely affect treatment response.

The pivotal role in the management of CM with MO is the “physician-patient alliance” deriving from the active involvement of the patient in all phases of her/his health-care management through a comprehensive rehabilitation counseling. Such a management is not limited to the earliest stages of treatment (diagnosis, therapies, detoxification), being very important also when the patients resume their everyday life (4). It is important to prevent unrealistic expectations (e.g., complete healing) and identify intermediate steps. In conclusion, the complete management of CM and MOH patients derives from an array of synergistic and multidisciplinary interventions aimed to improve patient’s quality of life, response to treatments and clinical outcome.

**Conflicts of interests**

None

**References**

1. Linde M, Gustavsson A, Stovner LJ, Steiner TJ, Barré J, Katsarava Z, Lainez JM, Lampl C, Lantéri-Minet M, Rastenyte D, Ruiz de la Torre E, Tassorelli C, Andrée C. The cost of headache disorders in Europe: the Eurolight project. Eur J Neurol. 2012 May;19(5):703-11.

2. Bottiroli S, Allena M, Sances G, De Icco R, Avenali M, Fadic R, Katsarava Z, Lainez MJ, Goicochea MT, Jensen RH, Nappi G, Tassorelli C; Comoestas Consortium. Changes in anxiety and depression symptoms associated to the outcome of MOH: A post-hoc analysis of the Comoestas Project. Cephalalgia. 2017 Jan [Epub ahead of print]

3. Bottiroli S, Viana M, Sances G, et al. Psychological factors associated to failure of detoxification treatment in chronic headache associated with medication overuse. Cephalalgia 2016; 36: 1356-1365.

4. Tassorelli C, Jensen R, Allena M, De Icco R, Sances G, Katsarava Z, Lainez M, Leston J, Fadic R, Spadafora S, Pagani M, Nappi G; the COMOESTAS Consortium. A consensus protocol for the management of medication-overuse headache: Evaluation in a multicentric, multinational study. Cephalalgia. 2014 Aug; 34(9):645-655.

### S58 Headache in the elderly

#### Carlo Lisotto

##### Headache Centre, Department of Neurology, Azienda Sanitaria Friuli Occidentale, Pordenone, Italy

**Background**

Headache prevalence is age-dependent and decreases progressively over time, especially starting from the age of 55-60. The incidence of primary headaches declines, whereas secondary headaches tend to occur more frequently with increasing age [1]. Although the prevalence of headache in the elderly is relevant, few studies have been conducted in patients over 65 so far.

**Materials and Methods**

The clinical records of 9075 consecutive outpatients aged over 18 referred to our Headache Centre from 2000 to 2015 were reviewed. Patients were diagnosed based on The International Classification of Headache Disorders, 3rd edition (beta version) criteria [2].

**Results**

Out of 9075 patients, a total of 469 (5.2%) were over 65 at their first observation. Primary headaches were diagnosed in 365 patients (80.5%, mean age 70.1 ± 4.7), secondary headaches in 64 cases (11.2%, mean age 74.1 ± 6.1), whereas painful cranial neuropathies and other facial pains were identified in 40 subjects (8.3%, mean age 77.1 ± 5.9). In the primary headache group the most common disorders were migraine without aura (26.0%), chronic tension-type headache (23.0%) and chronic migraine (20.3%). As for patients with migraine and chronic tension-type headache, the onset of headache occurred in most cases before 45, in particular in chronic migraine (89.2%), while in migraine with aura patients the headache started over 45 in 55.6% of cases. Secondary headaches were represented above all by cervicogenic headache, frequently associated with tension-type headache. Among cranial neuropathies, trigeminal neuralgia was by far the most commonly diagnosed headache.

**Conclusions**

In our population of elderly headache patients, migraine without aura, chronic tension-type headache and chronic migraine accounted for 61.3% of the total cases. There was a large majority of females in all the subgroups of headaches. In cluster headache, considered as a typical disorder of young men, we found indeed a slight preponderance of females. Migraine with aura not infrequently occurs in the elderly; this headache, as well as cluster headache, can even start, even rarely, over 65 and in such cases a differential diagnosis with a possible secondary disorder is mandatory. Among patients with chronic headaches, a medication overuse was found more frequently in chronic migraine (71.6%), than in chronic tension-type headache (33.3%). The choice of headache treatment is challenging, since specific guidelines are lacking and also because elderly patients commonly present with comorbidities. Further clinic-based studies should be carried out, with the aim to define possible therapeutic guidelines for these patients.

**References**

1. Schwaiger J, Kiechl S, Seppi K, Sawires M, Stockner H, Erlacher T, Mairhofer ML, Niederkofler H, Rungger G, Gasperi A, Poewe W, Willeit J. Prevalence of primary headaches and cranial neuralgias in men and women aged 55-94 years (Bruneck Study). Cephalalgia 2009;29: 179-187.

2. Headache Classification Committee of the International Headache Society (IHS). The International Classification of Headache Disorders, 3rd edition (beta version). Cephalalgia. 2013; 33:629-808.

3. Lisotto C, Mainardi F, Maggioni F, Dainese F, Zanchin G. Headache in the elderly: a clinical study. J Headache Pain. 2004; 5:36-41.

## EHF POSTER PRESENTATIONS

### P1 Anti-CGRP monoclonal antibodies for the treatment of chronic migraine: an overview of available results and comparison with the currently used prophylactics

#### Fenne Vandervorst, Laura Van Deun, Jacques De Keyser, Jan Versijpt

##### Department of Neurology, University Hospital Brussels, Brussels, Belgium

###### **Correspondence:** Fenne Vandervorst

Treatment of patients with chronic migraine remains challenging in daily clinical practice due to several factors: variable tolerability of the currently available medical treatments, frequent co-existence of medication overuse and lack of disease specific treatment strategies.

At this stage, Topiramate and OnabotulinumtoxinA are the only evidence based treatments for chronic migraine, of which only OnabotulinumtoxinA is FDA-approved.

Therefore, anti-CGRP monoclonal antibodies are considered an attractive treatment option for this patient population. Multiple studies with anti-CGRP monoclonal antibodies for the treatment of chronic migraine were conducted and some are still ongoing.

Here, we present an early overview on currently available efficacy results of anti-CGRP monoclonal antibodies in the treatment of chronic migraine. Secondly, these results are compared to the results of the currently used prophylactics for chronic migraine.

At present results from two phase 2 and two phase 3 trials are available.

In order to analyse these early results next to the efficacy results of established prophylactics for chronic migraine the ‘change in monthly migraine days compared to placebo’ of each agent will be presented.

Results from 1863 patients with chronic migraine, treated with anti-CGRP monoclonal antibodies are now available, compared to 688 patients treated with OnabotulinumtoxinA and 185 patients treated with Topiramate.

The overall mean reduction of monthly migraine days (compared to placebo) for the anti-CGRP monoclonal antibodies is -2,05 days. For Topiramate and OnabotulinumtoxinA these values are respectively -1,79 and -2 days.

In conclusion, the first efficacy results of anti-CGRP monoclonal antibodies in the treatment of chronic migraine are promising and at least comparable with the effect sizes of both Topiramate and OnabotulinumtoxinA. Combined with the fact that the anti-CGRP monoclonal antibodies have, already at this stage with several trials still ongoing, the highest number of patients studied ánd given their excellent tolerability, these new agents are emerging, from a clinical point of view, as a promising treatment for chronic migraine.

### P2 Biomarkers of inflammation in patients with Chronic Migraine after withdrawal from Medication Overuse: longitudinal changes and comparison with healthy subjects

#### Licia Grazzi^1^, Emanuela Sansone^1^, Alberto Raggi^2^, Matilde Leonardi^2^, Emilio Ciusani^3^, Elena Corsini^3^, Giovanni D’Andrea^4^, Andrea Bolner^4^, Francisco Salgado-García^5^, Frank Andrasik^5^, Domenico D’Amico^1^

##### ^1^Headache and Neuroalgology Unit; Neurological Institute “C. Besta” IRCCS Foundation; Milan; 20133; Italy; ^2^Neurology, Public Health and Disability Unit; Neurological Institute “C. Besta” IRCCS Foundation; Milan; 20133; Italy; ^3^Laboratory of Clinical Pathology and Medical Genetic; Neurological Institute “C. Besta” IRCCS Foundation; Milan; 20133; Italy; ^4^Research & Innovation (R&I); Padua; 35121; Italy; ^5^Department of Psychology; University of Memphis; Memphis, TN; 38152; USA

###### **Correspondence:** Domenico D’Amico

**Background**

Chronic Migraine (CM) is characterized by more than 15 days/month for more than three months, and is frequently associated to the overuse of acute medications. In recent years, non-pharmacological treatments have been proposed and, among them, Mindfulness is one of the most promising^1^ and showed to be comparable to pharmacological prophylaxis^2^. It was also shown that patients undergoing pharmacological prophylaxis and Mindfulness sessions had similar baseline levels for biomarkers and in part underwent significant improvement^3^. Here we aim to address improvement in biomarkers by comparing results on patients with those of healthy controls.

**Materials and methods**

Two group of patients, one receiving pharmacological prophylaxis alone and one treated with six group session of Mindfulness-based training, were followed-up at 3, 6 and 12 months. We compared patients and control for white blood cell (WBC) count, neutrophils, total lymphocytes and lymphocyte subsets CD3, CD4, CD8, CD19 at each point with Mann-Whitney test.

**Results**

Data were available for 34 patients (17 per group: no differences were found cross-sectionally, and the longitudinal course was similar) and 34 controls. Compared to controls, patients reported higher baseline levels for WBC, neutrophils and lymphocyte subsets CD4. All levels, except lymphocyte subsets CD4, became similar to that of controls from the third month, and CD4 became comparable at six months from withdrawal.

**Conclusions**

Some biomarkers of inflammation were altered in patients with CM associated to medication overuse: from the third month after withdrawal patients show a reduction of inflammation that is maintained at 12 months. Pharmacological prophylaxis and Mindfulness showed comparable results.

**Fig. 1 (abstract P2).**
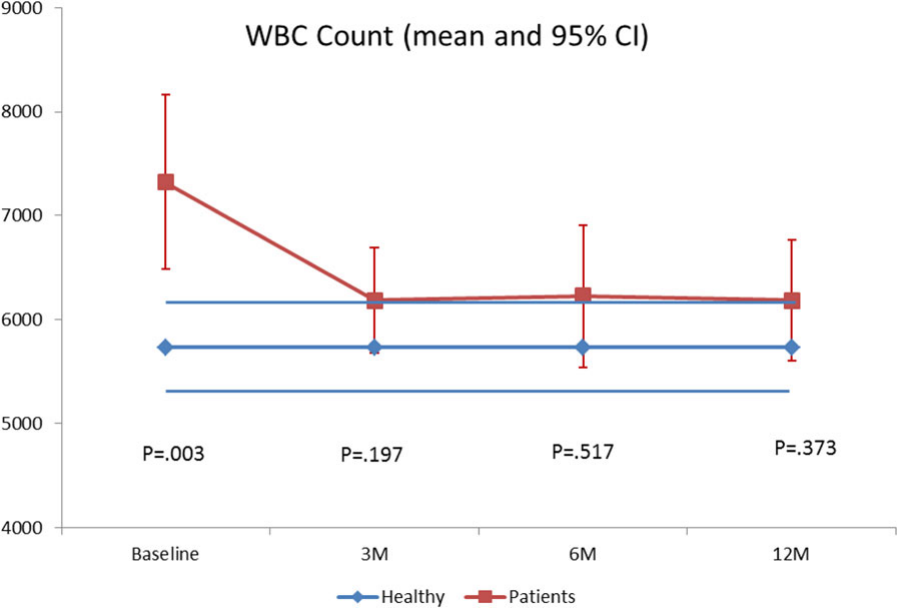
WBC Count (mean and 95% CI)

**Fig. 2 (abstract P2).**
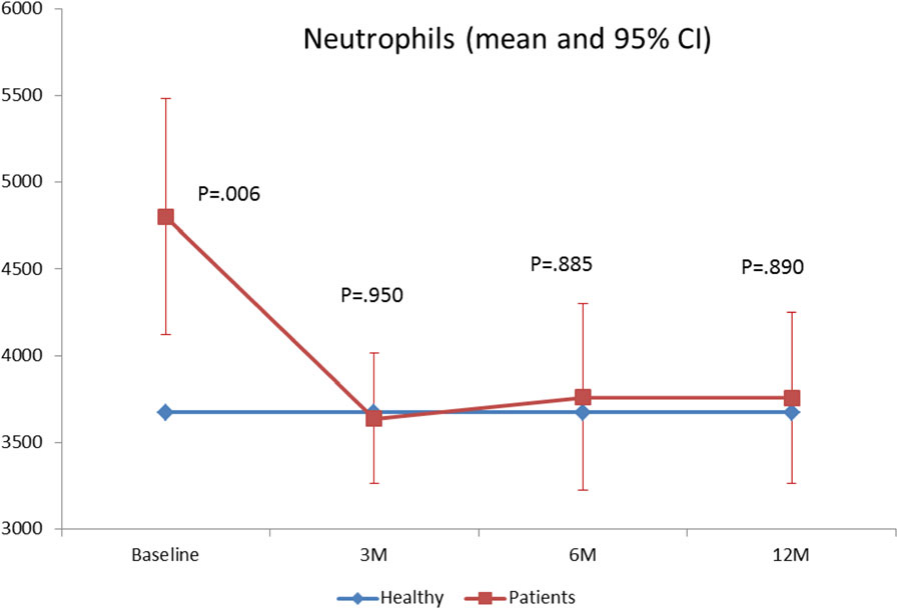
Neutrophils (mean and 95% CI)

**Fig. 3 (abstract P2).**
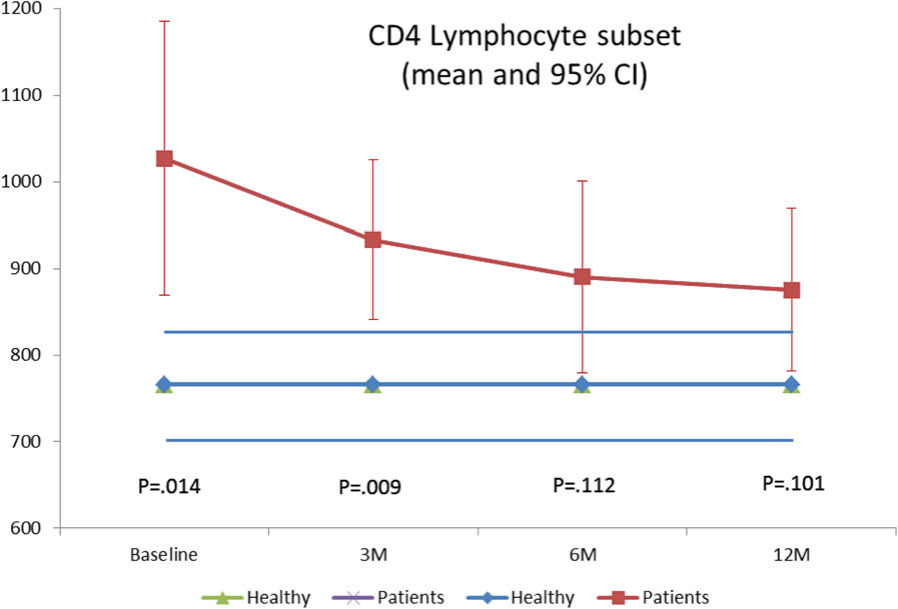
CD4 Lymphocyte subset (mean and 95% CI)

**Table 1 (abstract P2).** See text for description

	WBC	Neutrophils	Lymphocytes	CD3	CD4	CD8	CD19
Healthy Controls	5732 [5299-6165]	3674 [3233-4010]	1836 [1703-1971]	1366 [1259-1473]	766 [703-829]	559 [486-632]	196 [171-222]
Patients-T0	7322 [6489-8156]	4801 [4120-5482]	2019 [1773-2265]	1577 [1364-1790]	1027 [869-1185]	568 [491-645]	219 [186-253]
P-value	.003	.006	.611	.362	.014	.949	.627
Patients-3M	6185 [5681-6690]	3639 [3263-4016]	1899 [1730-2267]	1475 [1332-1617]	933 [841-1026]	550 [481-619]	196 [166-226]
P-value	.197	.950	.677	.253	.009	.827	.663
Patients-6M	6226 [5544-6908]	3762 [3226-4297]	1829 [1659-1998]	1399 [1263-1535]	890 [779-1001]	525 [475-576]	190 [156-224]
P-value	.517	.885	.820	.969	.112	.748	.264
Patients-12M	6182 [5601-6763]	3758 [3266-4250]	1802 [1639-1965]	1367 [1230-1504]	875 [781-969]	515 [461-570]	186 [157-214]
P-value	.373	.890	.598	.729	.101	.480	.278

**References**

1) Andrasik F, et al. Mindfulness and headache: A “new” old treatment, with new findings. Cephalalgia. 2016;36(12):1192-1205.

2) Grazzi L, et al. Mindfulness and pharmacological prophylaxis after withdrawal from medication overuse in patients with Chronic Migraine: an effectiveness trial with a one-year follow-up. J Headache Pain. 2017;18(1):15.

3) Grazzi L, et al. Mindfulness and pharmacological prophylaxis have comparable effect on biomarkers of inflammation and clinical indexes in chronic migraine with medication overuse: results at 12 months after withdrawal. Neurol Sci 2017;38(Suppl 1):173-175.

### P3 Outcome of microvascular decompression in trigeminal neuralgia is highly dependent on sex and degree of neurovascular contact – A prospective systematic study using independent assessors

#### Tone B. Heinskou1, Per Rochat2, Stine Maarbjerg1, Frauke Wolfram3, Jannick Brennum2, Jes Olesen1, Lars Bendtsen1

##### 1Danish Headache Center, Department of Neurology, Rigshospitalet. Faculty of Health and Medical Sciences, University of Copenhagen., Glostrup; 2Department of Neurosurgery, Rigshospitalet. Faculty of Health and Medical Sciences, University of Copenhagen., Copenhagen; 3Department of Diagnostics, Rigshospitalet. Faculty of Health and Medical Sciences, University of Copenhagen., Glostrup, Denmark

###### **Correspondence:** Tone B. Heinskou

**Objectives:** Microvascular decompression (MVD) is first choice neurosurgical treatment option in medically refractory trigeminal neuralgia patients with an MRI verified neurovascular contact (NVC). There is a lack of high-quality prospective, systematic studies, using independent assessors of outcome of the procedure. Here we aimed to evaluate whether sex and degree of NVC can predict outcome of MVD.

**Methods:** Clinical characteristics and outcome data were systematically recorded prospectively from consecutive trigeminal neuralgia patients, using standardized semi-structured interviews and schemes. A pre-surgical 3.0 Tesla MRI was performed to evaluate the degree of NVC blinded to symptomatic side. The patients were assessed before and 3, 6 and 12 months after MVD by a neurologist at the Danish Headache Center. Department of Neurosurgery had no influence on recording or evaluation of data. Data from a self-completed 12 months post-surgical questionnaire including items on pain intensity, complications and satisfaction, were also recorded. The primary outcome was pain relief according to the Barrow Neurological Institute pain score (BNI I-VB), Table 1. Secondary outcome was patient satisfaction.

**Results:** From May 2012 to February 2016, 27 men and 33 women had completed one year follow- up. Mean age at operation was 59.9 years (range 28-80 years). Mean duration of disease was 6.6 years (range 1-40 years). Thirty-three patients (55%) had NVC with morphological changes.

Forty-three (72%) patients had an excellent outcome defined as ‘no pain, no medication’ (BNI I). Nine (15%) patients had a good outcome, while eight patients (13%) had poor outcome.

At multiple logistic regression the odds ratio between NVC with displacement or atrophy of the trigeminal nerve and excellent outcome was 5.2 (95% CI 1.3 – 20.1, P = 0.0183) and the odds ratio between sex (male vs. female) and excellent outcome was 10.6 (95% CI 2.0 – 56.1, P = 0.0057). There was no significant interaction between sex and severe NVC (p = 0.56).

**Conclusion:** These high-quality prospective data using independent assessors demonstrate that patients with morphological changes of the trigeminal nerve and male sex have a considerably better chance of an excellent outcome of MVD. These data should guide patients and physicians in decision-making before neurosurgery.

### P4 Headache Clinical Refractoriness

#### Christian Lampl

##### Headache Medical Center, Seilerstätte, Ordensklinikum Linz Barmherzige Schwestern, Austria

In the past years a unifying definition of refractory headache (rH) has been extensively discussed but, to date, has not been agreed upon. It is widely agreed, that refractoriness, for whatever category and disease, implies a high burden with tremendous impact in health related quality of life (HRQoL). Despite that fact, an overall accepted definition of rH would be more than important for managing and triaging patients to an appropriate level of care and for determining eligibility for epidemiological and clinical studies. What are the critical issues so far: (i) there is no standardized definition of rH; (ii) at the time of first diagnosis headache patients do not necessarily become refractory immediately, nor do they mandatorily remain refractory throughout the course of their disease; (iii) due to the necessity that most patients should be treated rapidly after diagnosis response to medication often is assessed without a pretreatment baseline and it remains unclear whether or not so-called refractory patients have had a substantial response to treatment; (iv) headache pain and associated symptoms are frequently intermittent, making this disease different from others that have been examined for treatment resistance; (v) the natural history is not known. For all these purposes the Board of the European Headache Federation (EHF) felt the need to develop new consensus criteria that define refractory chronic migraine (rCM) and refractory chronic cluster headache (rCCH). These new definitions of rCM and rCCH, which were agreed upon within the EHF, allows us to separate patients into categories of refractory and non-refractory, being important for clinicians, clinical and epidemiological trials.

**References**

1. Silberstein S, Dodick D, Pearlman S (2010) Defining the pharmacologically intractable headache for clinical trials and clinical practice. Headache 50:1499–1506

2. Schulman E, Lake A, Goadsby P, Peterlin BL, Siegel SE, Markley HG, Lipton RB (2008) Defining refractory migraine and refractory chronic migraine: proposed criteria. Headache 48:778–782

3. Stovner LJ, Hagen K, Jensen R, Katsarava Z, Lipton R, Scher A, Steiner T, Zwart JA (2007) The global burden of headache: a documentation of headache prevalence and disability worldwide. Cephalalgia 27:193–210

4. Goadsby PJ, Zanchin G, Geraud G, de Klippel N, Diaz-Insa S, Gobel H, Cunha L, Ivanoff N, Falques M, Fortea J (2008) Early vs. non-early intervention in acute migraine- ‘Act when Mild (AwM)’. A double-blind, placebo-controlled trial of almotriptan. Cephalalgia 28:383–391

### P5 Sphenopalatine ganglion block using Tx360 device. First results in refractory chronic migraine in Spain

#### Jose M Sanchez, Maria Rico, Elena Ameijide, Maria Castanon

##### Hospital Universitario Central de Asturias, Neurology, Oviedo, Spain

###### **Correspondence:** Jose M Sanchez (jmsancheza@gmail.com)

**Background**

Despite current treatments 2-4 % of migraneurs become chronic (1). This is a very disabling condition leading to a high suffering and low quality of life (2). Inhibiting sphenopalatine ganglion (SPG) could reduce the frequency of the crisis (3), but its access is quite difficult requiring aggressive methods (4). Tx360 device is a nasal applicator made of plastic material easing the access to the SPG and the application of local anaesthetic in its vicinity with minor inconveniencies (5).

**Materials and methods**

Twelve blocks (three each week during four weeks), were done with Bupivacaine 0,5% (0,3 cc each nostril), using the Tx360 device. We evaluate at the end of the 12th block (four weeks), efficacy parameters (mean reduction of headache days and pain intensity in a visual analogue scale), impact (Headache Impact Test [HIT-6]), and quality of life (Migraine-Specific Quality of Life Questionary [MSQ]), tools. We also analysed 30% and 50% response rates.

**Results**

Nine patients refractories to habitual treatments were treated (2 M, 7 F; mean age 48,1 ± 16). There was analgesic abuse in 8. At the 12th block there were a significant reduction of mean headache days (17,6 vs. 25,8, p 0,034), pain intensity (5,4 vs. 8,1, p <0,001), and mean analgesic consumption (13,6 vs. 54,6, p <0,000002). There were also a significant reduction in mean HIT6 (57,85 vs. 66,9), and MSQ (40,14 vs. 57,22). Forty five per cent had a reduction ≥50% of headache days. There were no significant adverse events but minor and transient local discomfort.

**Conclusions**

Repetitive blocks of the SPG with the Tx360 device seem to be an effective treatment in chronic migraine, even with analgesic abuse, with only minor adverse events. These benefits were evident both in headache days and in quality of life measures. Although encouraging these results must be confirmed in a greater number of patients, and know how long they will last.

**References**

1. Buse DC, Manack AN, Fanning KM, Serrano D, Reed ML, Turkel CC, Lipton RB. (2012) Chronic migraine prevalence, disability, and sociodemographic factors: results from the American Migraine Prevalence and Prevention Study. Headache. 2012;52:1456–1470

2. Steiner TJ, Birbeck GL, Jensen RH, Katsarava Z, Stovner LJ, Martelletti P. Headache disorders are third cause of disability worldwide. J Headache Pain. 2015;16:58.

3. Narouze S, Kapural L, Casanova J, et al. Sphenopalatine ganglion radiofrequency ablation for the management of chronic cluster headache. Headache. 2009;49:571–577.

4. Candido KD, Massey ST, Sauer R, Darabad RR, Knezevic NN. A novel revisión to the classical transnasal topical sphenopalatine ganglion block for the treatment of headache and facial pain. Pain Physician. 2013;16:E769-78

5. Cady RK, Saper J, Dexter K, Cady RJ, Manley HR. Long-term efficacy of a double-blind, placebo-contolled, randomized study for repetitive sphenopalatine blockade with bupivacaine vs. saline with the Tx360 device for treatment of chronic migraine. Headache. 2015;55:529-42.

### P6 Efficacy of erenumab (AMG 334) in chronic migraine patients with prior prophylactic treatment failure: Subgroup analysis of the phase 2, randomised, double-blind, placebo-controlled study

#### Messoud Ashina^1^, Stewart J Tepper^2^, Jan Lewis Brandes^3^, Uwe Reuter^4^, Guy P Boudreau^5^, David Dolezil^6^, Sunfa Cheng^7^, Dean Leonardi^7^, Robert A Lenz^7^, Jan Klatt^8^, Daniel D Mikol^7^

##### ^1^Danish Headache Center and Department of Neurology, Rigshospitalet Glostrup, Faculty of Medical and Health Sciences, University of Copenhagen, Copenhagen, Denmark; ^2^Geisel School of Medicine at Dartmouth, Hanover, NH; ^3^Nashville Neuroscience Group and Vanderbilt University School of Neurology, Nashville, TN; ^4^Department of Neurology, Charité Universitätsmedizin Berlin, Berlin, Germany; ^5^Headache unit Neurology department University Hospital Center of Montreal Qc. Canada; ^6^DADO MEDICAL s.r.o., Prague Headache Center, Czech Republic; ^7^Amgen Inc., Thousand Oaks, CA; ^8^Novartis Pharma AG, Basel, Switzerland

###### **Correspondence:** Messoud Ashina (ashina@dadlnet.dk)

**Background**

Erenumab, a fully human monoclonal antibody, selectively targets the CGRP receptor. A phase 2, 12-week randomised, double-blind, placebo-controlled study demonstrated efficacy of erenumab (70 mg and 140 mg) in patients with chronic migraine (CM), with a safety profile comparable to placebo. We report a prespecified subgroup analysis on prior prophylactic treatment failure (≥1, ≥2 and never failed) due to lack of efficacy and/or poor tolerability.

**Methods**

Patients (N=667; aged ≥18–65 years) with CM (≥15 headache days/month; ≥8 migraine days) were randomised (2:2:3) to once-monthly subcutaneous erenumab 70 mg, 140 mg or placebo. Efficacy endpoints were change in monthly migraine days (MMD), achievement of ≥50% reduction in MMD, change in monthly acute migraine-specific medication treatment days, and change in cumulative monthly headache hours. Assessments compared weeks 9–12 to baseline. No correction for multiple comparisons was performed.

**Results**

With erenumab 70 mg and 140 mg, there were greater reductions at week 12 in MMD and more patients achieved ≥50% reduction in MMD versus placebo in all three subgroups. Moreover, greater reduction in monthly acute migraine-specific medication treatment days was observed with erenumab 70 mg and 140 mg in patients who previously failed prophylactic medications versus placebo. Cumulative monthly headache hours reduced with erenumab 140 mg versus placebo in patients who failed prophylactic medications. Notably, placebo effect was greatest in patients who had never failed a prophylactic medication. Across endpoints, reductions were greater with erenumab 140 mg than 70 mg.

**Conclusion**

Numerically, erenumab 140 mg showed better efficacy in patients with CM who previously failed ≥1 or ≥2 current standard of care prophylactic medication(s).

### P7 Sequential presentation of ipsilateral supraorbital and lacrimal neuralgias in a patient

#### Chavarría Miranda, A.; Talavera De la Esperanza, B.; Martínez Pías, E.; Trigo López, J.; Gómez López de San Román, C.; Ruiz Piñero, M.; Pedraza Hueso, M.I.; Guerrero Peral, Á.L.; García Azorín, D

**OBJECTIVE:**

The first trigeminal nerve branch is divided in three main branches: lacrimal nerve (LN), frontal nerve, which divides into supraorbital (SON) and supratrochlear nerves and nasociliar nerve. We describe the case of a patient diagnosed of supraorbital nerve neuralgia who developed an ipsilateral lacrimal neuralgia.

**PATIENT AND METHODS:**

47-year-old woman with prior medical history of Crohn disease treated with Adalimumab and Azatioprine. She complained about a oppressive continuous pain, of 5/10 intensity according to Analogic Visual Scale circumscribed to the left supraciliar region, with 2-3 seconds length superimposed paroxysms of 8/10 intensity. In the physical examination we detected tenderness at the palpation of the supraorbital notch. She was treated by anesthetic lidocaine blockade successfully and was managed during 6 years with blockades every 3-10 months.

**RESULTS:**

In a regular consult she complained of a new oppressive pain of 6/10 intensity in the left superoexternal periorbital region, with 3 seconds stabbing paroxisms of 8/10 intensity. In the exam she presented pain at the palpation of lacrimal nerve and circumscribed hypoesthesia in the lacrimal nerve territory. We only performed SON blockade first but the superoexternal pain persisted, so we performed a specific lacrimal nerve blockade with pain cessation, confirming the diagnosis of Lacrimal Neuralgia. A facial, orbital and cranial CT did not show any abnormality.

**CONCLUSSION:**

Sequential presentation of pain in contiguous nervous branches in the absence of structural lesions supports the epicranial nature of the trigeminal terminal branches neuralgias.

**Consent for publication:** The authors declare that written informed consent was obtained for publication.

### P8 Cognitive impairment in episodic and chronic migraineurs and tension-type headache suffers

#### A. Bianchi, R. Monastero, M. Davì, F. Brighina, C. Camarda

##### Department of Experimental Biomedicine and Clinical Neurosciences, University of Palermo, Italy

###### **Correspondence:** A. Bianchi (alessia.bianchi@unipa.it)

**Background.** Migraine and tension-type headache are highly prevalent brain disorders characterized by recurrent painful attacks that lead to a highly disabling condition, particularly when chronic. Headache suffers frequently reported cognitive deficits, nonetheless previously data regarding cognitive impairment are inconclusive. The aim of this hospital-based study was to compare cognitive performance in subjects affected by different headache types including: migraine without aura (MWA), chronic migraine (CM), tension type headache (TTH) and chronic tension type headache (CTTH).

**Materials and methods.** We studied 307 patients, 246 woman and 61 male consecutively referred to the Adult Headache Centre, Neurological Unit of the University of Palermo during a 2-year period. Headache diagnoses were established according to the ICHD-III criteria. Each patient carried out a comprehensive neuropsychological evaluation including: MiniMental – State Examination (MMSE), Rey Auditory Verbal Learning Test (episodic memory), Token Test (verbal comprehension), Frontal Assessment Battery (executive functioning), and Visual Search (selective attention). Impaired cognitive domains were computed according to age- and education Italian normative data (dichotomised variable of impaired versus not impaired). The presence of co-existing anxiety and depression was evaluated with the Hospital Anxiety and Depression Scale.

**Results.** In our sample mean age was 49,9±12,7 years for women (80%) and 50,8 ±14,07 years for men (20%) (Table 1). Mean age was higher in CTTH (55,8 ±13,7; p≤.0001). The most frequent form of headache was MWA (45%) followed by TTH (27,7%), CM (14%) and CTTH (13,7). The mean MMSE score was 28,2 ± 1,6 with no statistical difference between sex and headache types. Depression and anxiety were more frequent in female [HAD-A: 9,9 ± 4,5 versus 7,5 ± 4,4; p≤.0001) HAD-D (7,5 ± 3,9 versus 6,1 ± 3,9; p≤.0001)]. Sixty-one (19.9%) patients reported impairment in at least one cognitive domain; CTTH suffers reported the highest prevalence of cognitive deficits (28,6%), even it did not reach statistically significance (Table 2). Just one patient reported a praxis deficit. In our sample, after multiple logistic regression analysis, cognitive impairment seems not to be influenced by headache type even when data are adjusted by age and educational status.

**Conclusion.** Cognitive impairment is rather frequent in our hospital-based cohort. It was reported in about 20% of the population study, while the prevalence estimated in general population is about 5-6%. In our sample, cognitive deficits were more frequent in CTTH. Future research on bigger sample will evaluate cognitive performance in different headache types versus controls.

**Table 1 (abstract P8).** Characteristics of study population

Study population n: 307		
	Number:	Percent
Sex		
Male	61	19,9%
Female	246	80,1%
Age group (year-old)		
<21	5	1,6
21 – 30	20	6,5
31 – 40	38	12,4
41 – 50	94	30,6
51 – 60	86	28,0
61 – 70	48	15,6
≥71	16	5,2
Headache type		
Migraine without aura	138	45
Chronic Migrain	43	14,0
Tension type headache	84	27,7
Chronic tension type headache	42	13,7
Education (years of school		
0	3	1,0
1– 5	85	27,7
6– 8	104	33,9
9– 13	79	25,7
>13	36	11,7
Marital status		
Unmarried	44	14,3
Married	234	76,2
Divorced	29	9,4
Mean ± DS		
MMSE	28,2 ± 1,6	
HAD_A	9,4 ± 4,6	
HAD_D	7,2 ± 4,0

**Table 2 (abstract P8).** Prevalence of impairment in different cognitive domains stratified by headache type

Prevalence of cognitive deficit n = 61						
	Prevalence of attentive deficit	Prevalence of esecutive deficit	Prevalence of language deficit	Prevalence of memory deficit	Prevalence of praxis deficit	Prevalence of cognitive deficit in ≥1 domain
Migraine without aura	11 (8%)	8 (5,8%)	2 (1.4%)	18 (13%)	0 (0,0%)	29 (21.0%)
Chronic Migrain	4 (9,3%)	2 (4,7%)	0 (0,0%)	4 (9,3%)	1 (2,3%)	6 (14.0%)
Tension type headache	7 (8,3%)	4 (8%)	2 (2,4%)	7 (8,3%)	0 (0,0%)	14 (16.7%)
Chronic tension type headache	5 (11,9%)	6 (14,3%)	2 (4.8%)	6 (14,3%)	0 (0,0%)	12 (28.6%)

**Acknowledgements**

Financial support: no specific financial support was received for this study.

**References**

1) Rist PM, Kurth T. Migraine and cognitive decline: a topical review. Headache. 2013 Apr; 53(4):589-98.

2) Gil-Gouveia R, Oliveira AG, Martins IP. Assessment of cognitive dysfunction during migraine attacks: a systematic review. J Neurol. 2015 Mar;262(3):654-65.

### P9 A multicenter, prospective, randomized, open-label study to compare the efficacy, safety, and tolerability of onabotulinumtoxinA and topiramate for headache prophylaxis in adults with chronic migraine: the FORWARD study

#### John F. Rothrock^1^, Aubrey Manack Adams^2^, Esther Jo^3^, Xiang Zhao^4^, Andrew M. Blumenfeld^5^

##### ^1^George Washington School of Medicine, Washington, DC, 20037, USA; ^2^Global Medical Affairs, Allergan plc, Irvine, CA, 92623-9534, USA; ^3^Bioststistics, Allergan plc, Irvine, CA, 92623-9534, USA; ^4^Statistics, Pharmaceutical Product Development, LLC, Austin TX, 78744, USA; ^5^Headache Center of Southern California, The Neurology Center, Carlsbad, CA, 92024, USA

###### **Correspondence:** John F. Rothrock (jrothrock@mfa.gwu.edu)

**Background**

To compare the efficacy, safety, and tolerability of onabotulinumtoxinA and topiramate for preventive treatment of chronic migraine (CM) in adults.

**Materials and Methods**

The FORWARD Study randomized adults with CM (1:1) to receive 155 U onabotulinumtoxinA every 12 weeks (±7 days) for 3 treatment cycles or topiramate 50-100 mg/day administered up to week 36. Patients who discontinued topiramate at any time were allowed the option of crossing-over to receive onabotulinumtoxinA at the next scheduled office visit (week 12 up to week 36; Fig. 1). The primary efficacy measure was a dichotomous variable (responder/nonresponder) defined as the proportion of patients with ≥50% reduction in headache days during the 28-day period before week 32 (weeks 29-32). A baseline last observation carried forward imputation method was utilized to impute missing data replacing the missing value with the baseline value if the responder rate was missing at week 32 for any reason. Adverse events (AEs) were monitored. Safety data include AEs from randomization and cross-over phases.

**Results**

282 patients were enrolled (onabotulinumtoxinA, n=140; topiramate, n=142) at 35 US sites. Patients were primarily female (n=239, 84.8%); mean (SD) baseline headache days (onabotulinumtoxinA, 22.1 [4.6]; topiramate, 21.8 [4.8]) were similar across treatment groups. 148 patients completed treatment as randomized (onabotulinumtoxinA, n=120 [85.7%]; topiramate, n=28 [19.7%]) through week 32. Primary reasons for withdrawal were ineffective treatment (onabotulinumtoxinA, n=7 [5.0%]; topiramate, n=28 [19.7%]) and AEs (onabotulinumtoxinA, n=5 [3.6%]; topiramate, n=72 [50.7%]). 80 topiramate patients crossed-over to onabotulinumtoxinA.

OnabotulinumtoxinA demonstrated significantly higher proportion of patients with ≥50% reduction in headache frequency compared to baseline vs topiramate (40.0% vs 12.0%, respectively; adjusted OR, 5.0 [95% CI, 2.7-9.2]; *P*<0.001) at the week-32 assessment.

AEs were reported by 45.5% of onabotulinumtoxinA and 76.8% of topiramate patients; serious AEs by 1.4% and 4.2%, respectively. Only sinusitis was reported in ≥5% of 220 patients receiving onabotulinumtoxinA at any time; a number of individual AEs were reported in ≥5% receiving topiramate (Table 1). Treatment-related AEs were reported by 17.3% of onabotulinumtoxinA and 69.0% of topiramate patients. One serious AE (*nephrolithiasis*) was reported as related to topiramate.

**Conclusions**

In this open-label study, preventive treatment of adults with CM with onabotulinumtoxinA demonstrated a more favorable tolerability profile than topiramate. When using imputation methods accounting for differences in discontinuation rates, onabotulinumtoxinA was more effective than topiramate based on ≥50% responder rates and headache day reduction.

**Fig. 1 (abstract P9).**
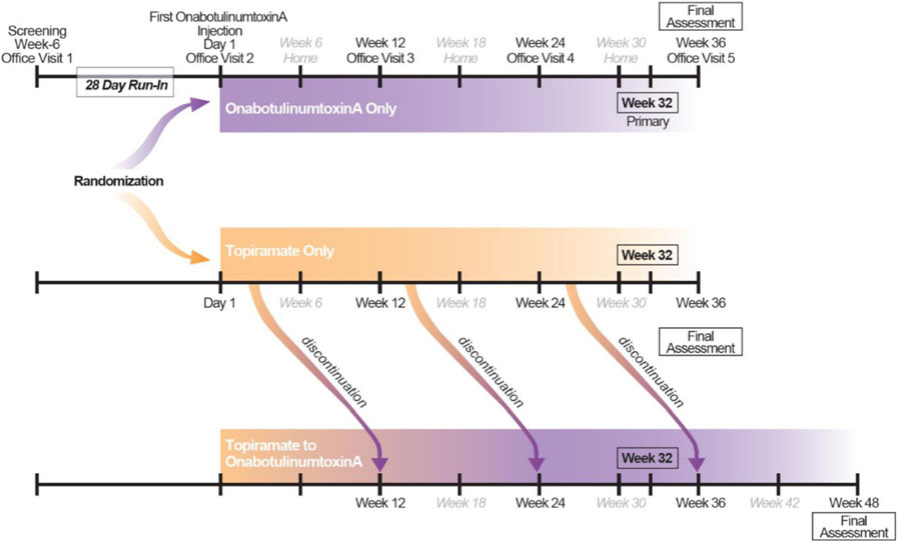
FORWARD Study methodology

**Table 1 (abstract P9).** Adverse events in ≥5% of Patients in Any Treatment Group

Patients with Adverse Event (AE), % (n)	OnabotulinumtoxinA (n=220)	Topiramate (n=142)	Total (N=282)	Switched to OnabotulinumtoxinA (n=80)
Any AE	45.5 (100)	76.8 (109)	62.4 (176)	41.3 (33)
Cognitive disorder	0.5 (1)	12.7 (18)	6.4 (18)	1.3 (1)
Disturbance in attention	0	7.7 (11)	3.9 (11)	0
Dizziness	2.7 (6)	12.7 (18)	8.2 (23)	1.3 (1)
Migraine	2.7 (6)	2.1 (3)	2.5 (7)	5.0 (4)
Paraesthesia	0.5 (1)	31.0 (44)	16.0 (45)	0
Sinusitis	5.5 (12)	6.3 (9)	5.7 (16)	6.3 (5)
Nausea	0.5 (1)	13.4 (19)	7.1 (20)	0
Neck pain	4.5 (10)	2.1 (3)	4.3 (12)	6.3 (5)
Fatigue	0.5 (1)	13.4 (19)	7.1 (20)	0
Depression	1.8 (4)	5.6 (8)	3.5 (10)	2.5 (2)
Vision blurred	2.7 (6)	7.0 (10)	5.0 (14)	3.8 (3)
Decreased appetite	0	10.6 (15)	5.3 (15)	0

**Funding**

Allergan plc

**Trial Registration**

ClinicalTrials.gov, NCT02191579

### P10 Chronic migraine treatment with erenumab: Responder rates

#### Hans-Christoph Diener^1^, Jan Brandes^2^, David Dolezil^3^, Marshall C Freeman^4^, Peter J McAllister^5^, Paul Winner^6^, Sunfa Cheng^7^, Dean K Leonardi^7^, Robert A Lenz^7^, Daniel D Mikol^7^

##### ^1^University Duisburg-Essen, Essen, Germany; ^2^Nashville Neuroscience Group, Nashville, TN, USA; ^3^Prague Headache Center, DADO MEDICAL s.r.o., Prague, Czech Republic; ^4^Headache Wellness Center, Greensboro, NC, USA; ^5^New England Institute for Neurology and Headache, Stamford, CT, USA; ^6^Palm Beach Headache Center, West Palm Beach, FL, USA; ^7^Amgen Inc., Thousand Oaks, CA, USA

###### **Correspondence:** Hans-Christoph Diener (Hans.Diener@uk-essen.de)

**Background**

Erenumab (AMG 334) is a human anti-calcitonin gene-related peptide (CGRP) receptor antibody being evaluated as preventive treatment for chronic migraine (CM). When assessing efficacy of CM treatments by responder rates, there is an unmet need for more effective treatments.

**Methods**

In a prospective exploratory analysis of data from a phase 2 study (NCT02066415) in patients with CM (≥15 headache days/month over 3 months with ≥8 migraine days), patients (N=667) were randomised to erenumab (70 mg or 140 mg once monthly) or placebo. This analysis included calculation of proportions of patients with ≥50%, ≥75%, or 100% reduction in monthly migraine days (MMD) from baseline to last 4 weeks of a 12-week double-blind phase. P-values are based on odds ratios (ORs) from placebo and are not adjusted for multiple comparisons.

**Results**

Mean (SD) baseline MMD were 18.0 (4.6). Significantly higher proportions of patients treated with erenumab 70 mg or 140 mg experienced a ≥50% reduction from baseline in MMD compared with placebo at Week 12 (39.9% and 41.2%, vs 23.5%; OR: 2.2 [p<0.001] and 2.3 [p<0.001]). The ≥75% responder rates were higher for patients treated with erenumab 70 mg or 140 mg compared with placebo (17.0% and 20.9%, vs 7.8%; OR: 2.4 [p=0.002] and 3.1 [p<0.001]). Likewise, the 100% responder rates were higher for patients treated with erenumab 70 mg or 140 mg compared with placebo (4.3% and 2.7%, vs 0.4%; OR: 12.6 [p=0.002] and 8.1 [p=0.026]).

**Conclusions**

Erenumab treatment resulted in higher proportions of patients with CM experiencing ≥50%, ≥75%, and 100% reduction in MMD as compared with placebo.

### P11 Systematic Cochrane review of botulinum toxins for the prevention of migraine in adults

#### Alexandra Sinclair^1^, Clare P Herd^2^, Claire L Tomlinson^3^, Caroline Rick^3^, WJ Scotton^1^, Julie Edwards^4^, Natalie Ives^3^, Carl E Clarke^2^

##### ^1^Institute of Metabolism and Systems Research, University of Birmingham, Birmingham, UK; ^2^Institute of Applied Health Research, University of Birmingham, Birmingham, UK; ^3^Birmingham Clinical Trials Unit, University of Birmingham, Birmingham, UK; ^5^Department of Neurology, Sandwell and West Birmingham Hospitals NHS Trust, Birmingham, UK

**Objectives**

To assess the effects of botulinum toxins versus placebo, active treatment or different dose for prevention of episodic or chronic migraine in adults.

**Background**

Many migraine patients suffer prolonged and frequent migraine attacks despite optimised acute and prophylactic treatments. Botulinum toxin type A has been licensed for use in chronic migraine in some countries, based largely on two commercially sponsored trials.

**Methods**

Relevant trials were identified through electronic searches of Cochrane Central Register of Controlled Trials, Medline, Embase, and trials registries, handsearching reference lists and citation searches on key publications, and correspondence with manufacturers. We included randomised, double-blind, controlled trials. Twelve week time-point data following final round of treatment was analysed.

**Results**

Twenty-eight trials (N=4192) were eligible for inclusion. No trial carried out long term follow up. All larger trials (N>100) were at high risk commercial sponsorship bias, otherwise trial quality was mixed. Botulinum toxin was compared with placebo in 23 trials. Four trials (N=1497) of botulinum toxin in chronic migraineurs showed a reduced frequency of -3.1 migraine days/month (95% confidence interval (CI) -4.73 to -1.41) compared with placebo. Addition of one trial (418 participants) in episodic migraine lowered this pooled estimate of effect to -2.39 days/month (95% CI -4.02 to -0.76), still in favour of botulinum toxin. Secondary efficacy measures were inconsistent. Data for number of migraine attacks from six trials including chronic and episodic migraineurs showed no significant between group difference (P=0.30), but severity of migraine (10 cm visual analogue scale), was improved by -3.30 points (95% CI -4.16 to -2.45) more with active treatment. Global assessment and quality of life measures were poorly reported. Botulinum toxin had a relative risk of treatment related adverse events of twice that seen for placebo (2.18, 95% CI 1.73 to 2.75). Insufficient data was available to establish any dose-response relationship for any outcome measure. Three trials of comparisons with oral prophylactic agents independently reported no significant between group differences for a variety of diary data outcomes but meta-analysis was not possible. Compared with oral treatments, botulinum toxin showed a reduced relative risk of treatment-related adverse events of 0.76 (95% CI 0.59 to 0.98).

**Conclusions**

In chronic migraine, botulinum toxin type A reduces frequency of migraine by three days/month, reduces migraine severity by 30% and has a favourable safety profile compared with other preventative drugs. Evidence to support or refute the efficacy of botulinum toxin in episodic migraine was not identified.

### P12 Complete detoxification is the most effective treatment of medication-overuse headache: A randomized controlled open-label trial

#### Louise N Carlsen, Signe B Munksgaard, Rigmor H Jensen, Lars Bendtsen

##### Danish Headache Center, Department of Neurology, Rigshospitalet-Glostrup, Lars Bendtsen; Ndr. Ringvej 69, 2600 Glostrup, Denmark

###### **Correspondence:** Lars Bendtsen (lars.bendtsen@regionh.dk)

**Background:** There is lack of evidence on how to detoxify medication-overuse headache (MOH).

The aim was to compare the effect of complete stop of acute medication with restricted intake.

**Methods:** MOH-patients were included in a prospective, outpatient study and randomized to two-month detoxification with either A) no analgesics or acute migraine-medication, or B) acute medication restricted to two days/week. Detoxification was followed by preventives if indicated. Patients were followed-up at 2, 6 and 12 months. Percentage reduction in headache-days/month after 6 months was the primary outcome.

**Results:** We included 72 MOH-patients with a primary migraine and/or tension-type headache diagnosis. Fifty-nine completed detoxification, 58 (81%) were followed-up at month 6 and 53 (74%) at month 12. At month 6, program-A reduced headache-days/month by 46% (95% CI 34–58) compared with 22% (95% CI 11–34) in program-B (p=0.005), and 70% in program-A versus 42% in program-B were reverted to episodic headache (p=0.04). Migraine-days/month were reduced by 7.2 in program-A (p<0.001) and 3.6 in program-B (p=0.002) after 6 months.

**Conclusion:** Both detoxification programs were very effective. Detoxification without analgesics or acute migraine-medication was the most effective program.

**Trial registration:** Clinicaltrials.gov (NCT02903329).

### P13 Sphenopalatine ganglion block using Tx360 device. First results in refractory chronic cluster headache in Spain

#### Jose M Sanchez, Maria Rico, Maria Castanon, Elena Ameijide

##### Hospital Universitario Central de Asturias, Neurology, Oviedo, Spain

###### **Correspondence:** Jose M Sanchez (jmsancheza@gmail.com)

**Background**

Despite current preventive treatments almost 20% of patients with cluster headache become chronic [1], with severe repercussion in his/her daily activities and poor quality of life. Inhibiting sphenopalatine ganglion (SPG) could suppress the crisis [2], but its access is quite difficult requiring aggressive methods [3]. Tx360 device is a nasal applicator made of plastic material easing the access to the SPG and the application of local anaesthetic in its vicinity with minor inconveniencies [4].

**Materials and methods**

Twelve blocks (three each week during four weeks), of the SPG were done with Bupivacaine 0,5% (0,3 cc each nostril), using the Tx360 device. We evaluate at the end of the 12th block (four weeks), efficacy parameters (mean reduction of attack frequency and headache days), impact (Headache Impact Test [HIT-6]), and quality of life (Migraine-Specific Quality of Life Questionary [MSQ]), tools. We also analysed 30% and 50% response rates.

**Results**

Five patients refractories to standard oral therapies were treated (4 M, 1 F; mean age 41,6 ± 11,8). At the 12th block there was a significant reduction in mean attack frequency (6 vs. 15, p < 0,00002), and mean pain intensity (7 vs. 9,6, p< 0,005), not in mean headache days (18,6 vs 26, p 0,15). There was a significant reduction in mean HIT-6 (63 vs. 71), and MSQ (57 vs. 68). Four patients (80%), had a 50% or greater reduction in attack frequency, and two (20%), in headache days. There were no significant adverse events but minor and transient local discomfort; only one patient suffer a syncope two hours after the second block, probably not related to the procedure.

**Conclusions**

Repetitive blocks of the SPG with the Tx360 device seem to be an effective treatment in chronic cluster headache, with minor adverse events. These benefits were evident both in attack frequency and in quality of life measures. Although encouraging these results must be confirmed in a greater number of patients, and know how long they will last. This therapy probably should be tried before invasive treatments, with more serious adverse events.

**References**

1. Goadsby PJ. Pathophysiology of cluster headache: A trigeminal autonomic cephalalgia. Lancet Neurol. 2002;1:251-257.

2. Tepper SJ, Caparso A. Sphenopalatine Ganglion (SPG): Stimulation, Mechanism, Safety, and Efficacy. Headache. 2017;57:14-28.

3. Narouze S, Kapural L, Casanova J, et al. Sphenopalatine ganglion radiofrequency ablation for the management of chronic cluster headache. Headache. 2009;49:571–577.

4. Candido KD, Massey ST, Sauer R, Darabad RR, Knezevic NN. A novel revisión to the classical transnasal topical sphenopalatine ganglion block for the treatment of headache and facial pain. Pain Physician. 2013;16:E769-78.

### P14 Are there gender differences related to cost of disease in patients with Medication Overuse Headache receiving structured withdrawal?

#### Grazzi Licia^1^, D’Amico Domenico^1^, Emanuela Sansone^1^, Matilde Leonardi^2^, Raggi Alberto^2^

##### ^1^Headache and Neuroalgology Unit; Neurological Institute “C. Besta” IRCCS Foundation; Milan; 20133; Italy; ^2^Neurology, Public Health and Disability Unit; Neurological Institute “C. Besta” IRCCS Foundation; Milan; 20133; Italy

###### **Correspondence:** Grazzi Licia

**Background**

Medication Overuse Headache (MOH) impacts on patients’ daily life and is associated to increased burden and cost^1^. Our aim is to explore gender differences with regard to cost and treatments.

**Materials and methods**

Direct (medical and non-medical) and indirect cost were directly gathered from patients and referred to the previous three months. Direct cost included medications for acute treatment and prophylaxis, diagnostic procedures, visits, complementary treatments and informal care. Indirect costs were referred to missed workdays and workdays with headache, and we relied on patients’ report on their salaries and judgement on their overall level of performance for days worked with headache.

**Results**

A total of 159 patients (25 males – 15.7%) were included.

With regard to indirect costs, males had higher salaries (202 Vs. 103 €/day; P<.001) and were less frequently unemployed (9.5% Vs. 27%). Despite there were no differences on lost workdays and of days worked with headache, indirect costs were higher among males (2998 Vs. 1321 €/3-months; P=.022).

With regard to direct costs, there were no differences connected to the overall amount and cost of drugs for prophylaxis and for acute management, despite males consumed more triptans (89 Vs. 61 over 3 months; P=.019). Direct medical cost were comparable across gender, while non-medical cost were mostly reported and were higher for females (177 Vs. 19 €/3-months; P=.012). Taken as a whole, direct costs were higher among females (1359 Vs. 794 €/3-months; P=.046).

Total cost were higher for males, but not to a significant extent (3792€ Vs. 2680€ over three months).

**Conclusions**

Cost of MOH at the time point of withdrawal are high and widespread. Males reported higher indirect cost, likely due to higher salaries, while females reported higher direct cost, likely due to higher non-medical ones. However, overall costs were similar across gender. Taken as a whole, our data indicate that the annual cost per case of non-treated MOH might be approximately 11400€: considering that MOH prevalence is 2.1% among people aged 18-65^2^ (i.e. around 39 millions), the global annual cost would be 9336.6 million €.

**References**

1) Steiner TJ, et al GBD 2015: migraine is the third cause of disability in under 50s. J Headache Pain. 2016;17:104.

2) Allena M, et al. Impact of headache disorders in Italy and the public-health and policy implications: a population-based study within the Eurolight Project. J Headache Pain. 2015;16:100.

### P15 Optimal response to onabotulinumtoxina in chronic migraine: evaluation in a series of 124 patients

#### D García-Azorín, M Ruiz, Mi Pedraza, Al Guerrero

**Background:** OnabotulinumtoxinA (OnabotA) is considered a safe and effective preventive therapy in Chronic Migraine patients, as has been shown in the PREEMPT clinical program and in real-life setting. Though previously mentioned in literature, a possible excellent response to this therapy has not been previously assessed in clinical practice. We aimed to analyze the response to OnabotA, including characteristics of optimal responders in a series of CM patients.

**Materials and Methods:** We included 124 CM patients (108 females, 16 males) treated with OnabotA according to the PREEMPT paradigm in a headache unit. They had been previously treated with topiramate and at least one other medication from beta-blocker and flunarizine for at least three month, as recommended in local guidelines. Monthly headache and migraine days before and after OnabotA injections were recorded in a diary. Patients were considered as responders when a reduction of monthly headache days by at least 50% was achieved, and, among them, as optimal responders if the reduction obtained was over 75%.

**Results:** Mean age at first procedure was 41.8 ± 11.4 years (18-71). Latency between migraine onset and inclusion was 24 ± 12.9 years (2-61), and between CM onset and inclusion 39.7 ± 44.2 months (6-240). We classified 99 patients (79.8%) as responders and, among them, 30 (30.3) were considered as optimal responders. Among responders group, both age at inclusion (40.5±11 vs 47±12, p:0.02) and latency between migraine onset and OnabotA therapy (22.3±11.71 vs 20.4±15.4 years, p:0.021) were significantly decreased. Nevertheless, when comparing optimal responders with rest of responders we found no differences.

**Conclusion:** An optimal response to the first procedures of OnabotA is not exceptional in CM patients. It is advisable to consider this type of response in order to look for its predictors.

### P16 N=1 statistical approaches to examine within-individual risk factor profiles of ICHD-3beta classified migraines *versus* non-migraine headaches

#### Ty Ridenour^1^, Francesc Peris^2^, Gabriel Boucher^2^, Alec Mian^2^, Stephen Donoghue^2^, Andrew Hershey^3^

##### ^1^Behavioral and Urban Health, RTI International, Research Triangle Park, NC, 27709, USA; ^2^Curelator, Inc., Cambridge, MA, 02142, USA; ^3^Cincinnati Children’s Hospital Medical Center, Cincinnati, 45229, USA

**Background**

To what extent do migraines differ from non-migraine headaches (per ICHD-3beta criteria) in underlying pathophysiology? This study examined risk factors associated with (a) occurrence and (b) severity of both migraine vs non-migraine headaches. Because profiles of headache triggers / protectors vary greatly among patients, analyses were conducted at the individual level and their results then used to draw sample aggregate conclusions. For example, among participants who experienced a trigger, the proportion for whom the trigger was associated with only migraines, only non-migraine headaches, or both, was evaluated.

**Materials and methods**

Participants were 479 individuals with both migraines and non-migraine headaches identified by clinician referral or via the internet and registered to use a novel digital platform (Curelator HeadacheTM). Participants completed baseline questionnaires and entered daily data on headache occurrence, severity (level of pain), ICHD-3beta migraine symptom criteria, and exposure to 70 migraine risk factors. Nearly 88% of participants were female, 41% were US residents and 40% were UK residents. Cox regression tested associations between binomial occurrence of a (non)migraine headache and risk factors. Hierarchical linear modeling that was tailored for N=1 analysis (mixed model trajectory analysis or MMTA) tested associations between risk factors and pain severity of (non)migraine headaches. MMTA controlled for patient-specific time-related trends in pain severity (mild – moderate – severe), autocorrelation, and used conservative statistical tests for N=1 analyses.

**Results**

Regarding headache severity, 50% of risk factors were statistically associated with both migraine and non-migraine headaches whereas the other half were unique to one form of headache. However, within individuals, the particular risk factors that were associated with either form of headache varied greatly. Moreover, regarding specific risk factors, few individuals’ triggers / protectors were associated with both forms of headache. To illustrate, Fig. 1 presents the proportion of participants whose protector was associated with both forms of headache (rather than only one form).

**Conclusions**

Results suggest that risk factors associated with occurrence of migraines both overlap and differ from the factors of migraine severity. Moreover, these two sets of associations differ between migraine and non-migraine headaches. These observations imply that etiological factors differ between types of headaches. Results further suggest that treatment of migraines could aim to not only prevent attacks, but also reduce the pain (and thus impairment) that patients experience during a migraine headache, a strategy that could be particularly important for patients with chronic migraines.

**Fig. 1 (abstract P16).**
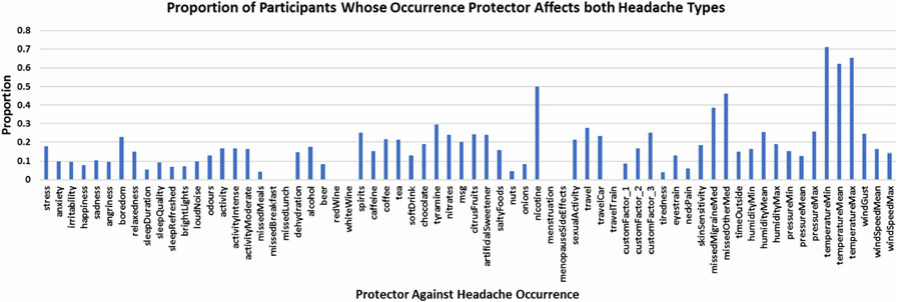
Proportion of Participants Whose Occurence Protector Affects both Headache Types

### P17 N=1 statistical approaches to examine risk factor profiles of ICHD-3beta classified migraines within individuals

#### Ty Ridenour^1^, Francesc Peris^2^, Gabriel Boucher^2^, Alec Mian^2^, Stephen Donoghue^2^, Andrew Hershey^3^

##### ^1^Behavioral and Urban Health, RTI International, Research Triangle Park, NC, 27709, USA; ^2^Curelator, Inc., Cambridge, MA, 02142, USA; ^3^Cincinnati Children’s Hospital Medical Center, Cincinnati, 45229, USA

**Background**

This investigation compared two within-individual analytic approaches to understand daily migraine occurrence and severity patterns in relation to a spectrum of suspected risk factors*.* Cox regression modelled migraine occurrence whereas headache severity was modelled using a form of hierarchical linear modeling tailored for intensive within-person analyses. These two techniques were compared in terms of which risk factors were identified as possible “triggers” of migraine occurrence versus possibly contributing to severity of a migraine.

**Materials and methods**

Participants were 479 individuals with migraines identified by clinician referral or via the internet and registered to use a novel digital platform (Curelator HeadacheTM). Participants completed baseline questionnaires and then entered daily data on headache occurrence and severity (level of pain), ICHD- 3beta migraine criteria, and exposure to 70 migraine risk factors. Nearly 88% of participants were female, 41% were US residents and 40% were UK residents. Risk factors spanned emotions, sleep qualities, environment and weather, lifestyle, diet, substance use, and travel. Cox regression modelled the binomial occurrence of migraine attacks per individual participant; hazard ratios quantified their strength of association with suspected triggers. The continuous measure of severity of migraine headache was modelled using mixed model trajectory analysis (MMTA), a form of hierarchical linear modeling. MMTA statistically controlled for patient-specific time-related trends in pain severity, autocorrelation, and used statistical tests that generate conservative estimates for N=1 analyses.

**Results**

Numerous risk factors were associated with occurrence and severity of migraine headaches. Cox regression detected potential triggers that were associated only with occurrence (not severity) of migraine attacks. Consistent with past evidence, the profile of risk factors that were associated with occurrence and severity of migraines varied considerably among patients, demonstrating that comprehensive clinical research on migraines requires analytics at the N=1 level. Moreover, “profiles” of triggers and protectors varied considerably among individuals (Fig. 1), suggesting that studies which only consider sample-aggregate results do not generalize to many migraine patients.

**Conclusions**

Cox regression and MMTA each provide unique insights regarding within-person patterns and correlates of migraine occurrence and severity, respectively. Cox regression’s detection of unique risk factors for occurrence of migraine headaches suggests that different risk factors are associated with occurrence of migraine attacks versus severity of migraine pain. Moreover, treatment of migraine headaches could aim to not only prevent occurrence of attacks, but also reduce pain level (and thus impairment) during a migraine headache, which could be especially important for patients with chronic migraines.

**Fig. 1 (abstract P17).**
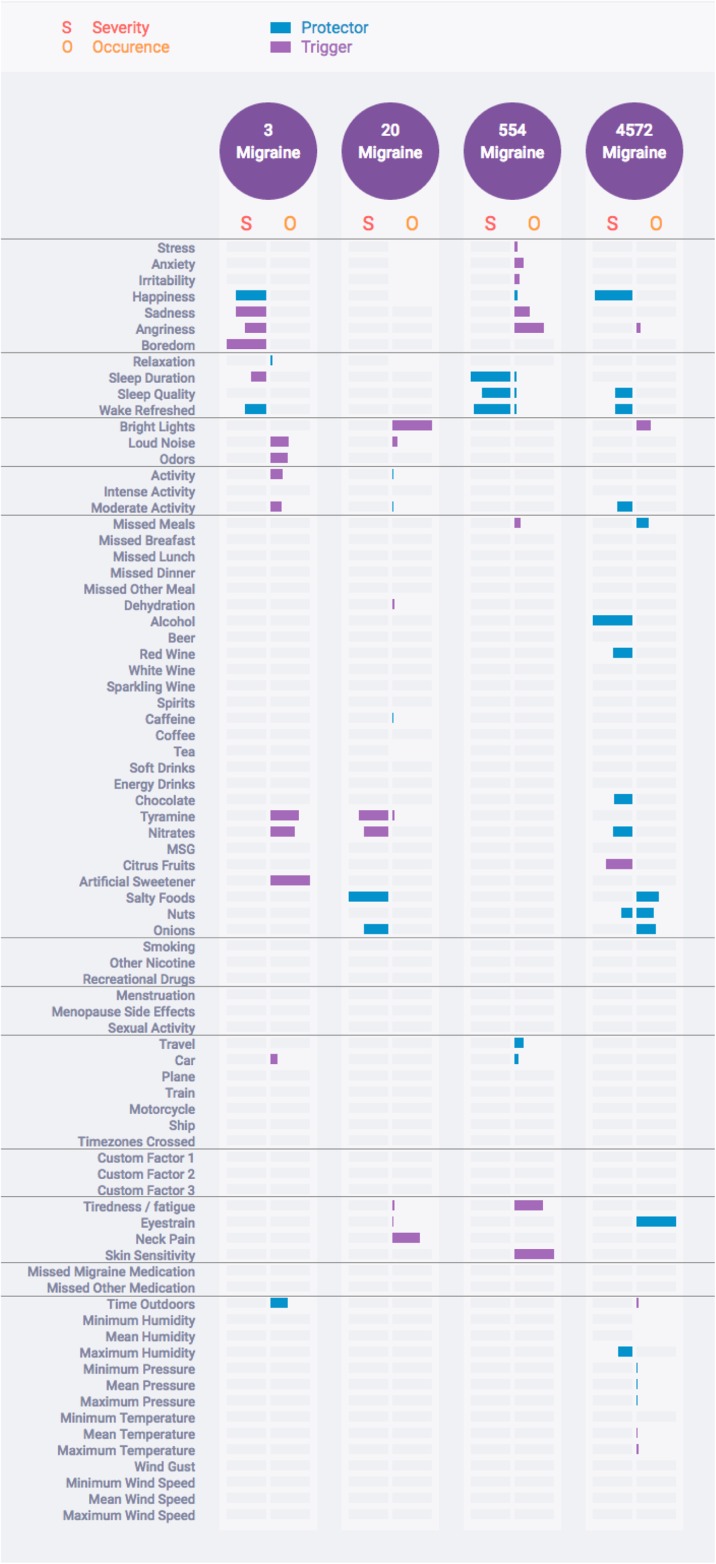
See text for description

### P18 Reliability and validity of a questionnaire for detecting cluster headache among headache patients

#### Pil-Wook Chung^1^, Soo-Jin Cho^2^, Kwang-Yeol Park^3^, Mi-Ji Lee^4^, Chin-Sang Chung^4^, Byung-Su Kim^5^, and Korean Cluster Headache Registry Group

##### ^1^Department of Neurology, Kangbuk Samsung Hospital, Sungkyunkwan University School of Medicine, Seoul; ^2^Department of Neurology, Dongtan Sacred Heart Hospital, Hallym University College of Medicine, Hwaseong; ^3^Department of Neurology, Chung-Ang University Hospital, Chung-Ang University, Seoul; ^4^Department of Neurology, Neuroscience Center, Samsung Medical Center, Sungkyunkwan University School of Medicine, Seoul; ^5^Department of Neurology, Bundang Jesaeng Hospital, Seoul, Republic of Korea

###### **Correspondence:** Pil-Wook Chung

**Background**

Cluster headache is a severe debilitating primary headache disorder. Because cluster headache is very rare compared with migraine, cluster headache is frequently misdiagnosed and neglected. We developed an 8-item self-administered questionnaire tool for detecting cluster headache among patients with primary headache disorder, and test its reliability and validity compared with neurologist’s diagnosis.

**Materials and Methods**

The candidate items were developed based on the diagnostic criteria of cluster headache from the international classification of headache disorder 3^rd^ edition beta version and expert opinions. The total score was calculated from the sum of positive response to each items (ranging 0 to 8). The questionnaire was self-administered during the first visit to headache clinic before neurologist’s diagnosis. The reliability and validity were tested among patients with various primary headache disorders

**Results**

In total, 342 patients were enrolled: 28 with cluster headache, 254 with migraine, 44 with tension-type headache, and 16 with primary stabbing headache. Cronbach alpha was 0.619 and the areas under the curve were 0.922 in receiver operating characteristic curves for all 8 items. Using the total score of 5 as cut-value, sensitivity and specificity were 83.3% and 90.9% for definite episodic cluster headache among 342 patients. The validity was similar for differentiating cluster headache from migraine. Remission or cluster period did not influence the detection rate.

**Conclusions**

This preliminary self-administered questionnaire for cluster headache is reliable and useful tool. It may be suitable for detecting cluster headache among primary headache disorders.

### P19 Postdural Puncture Headache after Cervical Medial Branch Block

#### Young In Lee^1^, Donggyu Han^2^, Yoo Jung Rark^2^, Eung Don Kim^1^

##### ^1^Department of Anesthesiology and Pain Medicine, Daejeon St. Mary’s Hospital, College of Medicine, The Catholic University of Korea ; ^2^Department of Anesthesiology and Pain Medicine, Saint Vincent’s Hospital, College of Medicine, The Catholic University of Korea

###### **Correspondence:** Young In Lee (ehs99@catholic.ac.kr)

Cervical medial branch block (MBB) is a frequently performed procedure for management of neck pain that rarely has complications [1]. With fluoroscopic guidance, the procedure is considered a relatively safer procedure than epidural block [2,3]. We report a case of a 27-year-old woman presenting with postural headache after cervical MBB. Although no specific evidence of dural injury was found in her cervical MRI (Fig. 1), dural penetration by inappropriate needle placement was suspected after reviewing fluoroscopic images of the procedure (Fig. 2). After conservative treatment, including bed rest and analgesic treatment, the patient completely recovered without any neurological complications. Complications associated with MBB are rare and previous case reports have focused only on infection or vascular injection as etiologies. This is the first report of complications related to dural puncture after cervical MBB. Our findings suggest that misplacement of the block needle by inaccurate alignment of both sides of the cervical articular pillar, assessed by fluoroscopic view during the procedure, can result in dural injury.

**Fig. 1 (abstract P19).**
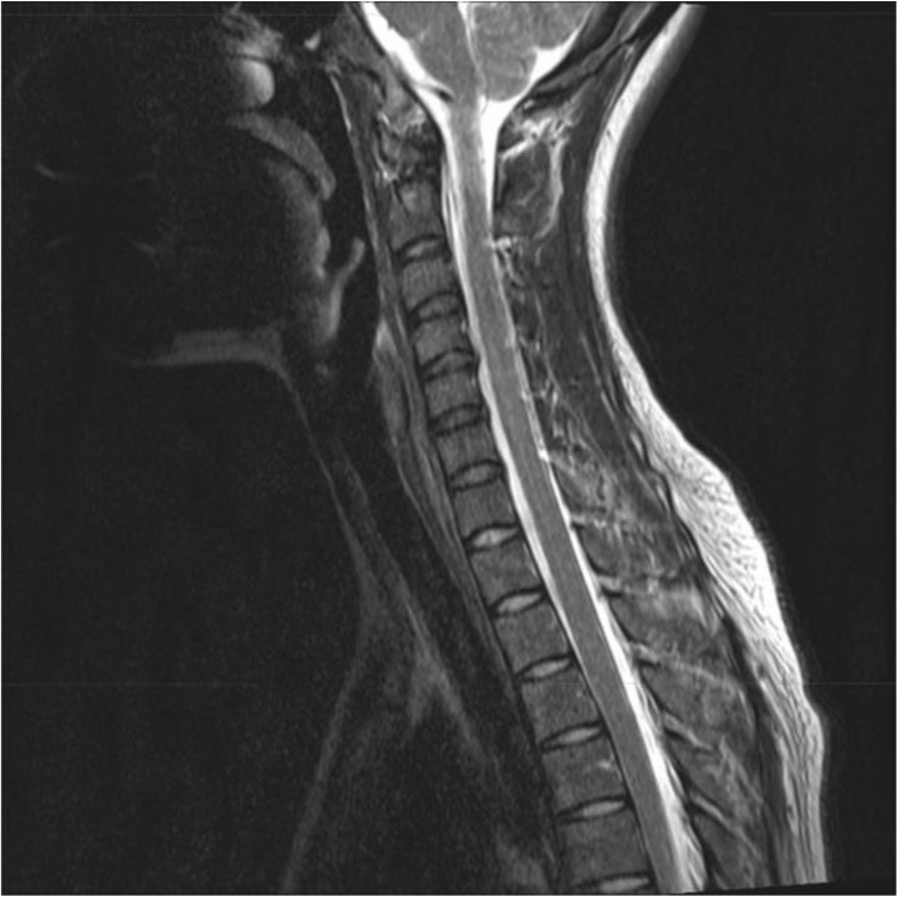
Cervical MRI of the patient

**Fig. 2 (abstract P19).**
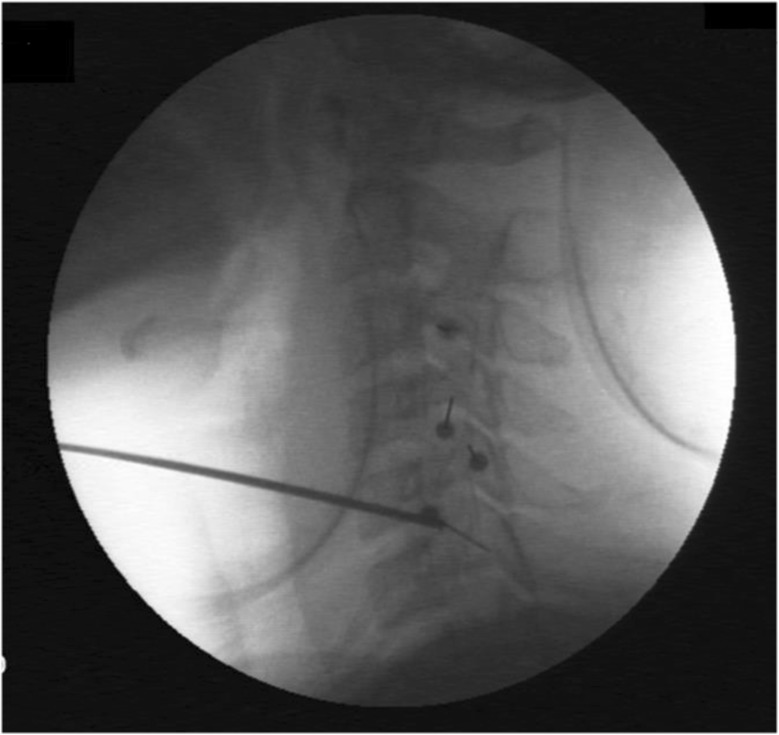
Fluoroscopic lateral view during cervical medial branch block

**Consent for publication:** The authors declare that written informed consent was obtained for publication.

**References**

1. Bogduk N, Marsland A. The cervical zygapophysial joints as a source of neck pain. *Spine* 1988; 13:610-7.

2. Barnsley L, Lord S, Bogduk N. Comparative local anaesthetic blocks in the diagnosis of cervical zygapophysial joint pain. *Pain* 1993; 55:99–106.

3. Verrills P, Mitchell B, Vivian D, Nowesenitz G, Lovell B, Sinclair C. The incidence of intravascular penetration in medial branch blocks: cervical, thoracic, and lumbar spines. *Spine* 2008; 33:E174–7.

### P20 Cervical Medial Branch Block using Botulinum Toxin Type A in Patient with Cervicogenic Headache

#### Young In Lee^1^, Donggyu Han^2^, Eung Don Kim^1^, Yoo Jung Rark^2^

##### ^1^Department of Anesthesiology and Pain Medicine, Daejeon St. Mary’s Hospital, College of Medicine, The Catholic University of Korea; ^2^Department of Anesthesiology and Pain Medicine, Saint Vincent’s Hospital, College of Medicine, The Catholic University of Korea

###### **Correspondence:** Young In Lee (genial7@naver.com); Yoo Jung Rark (genial7@naver.com)

**Background**

Cervicogenic headache (CGH) is defined as headache originating from various neck conditions. Transcutaneous electrical nerve stimulation, nerve block, botulinum toxin (BoNT) injection and radiofrequency neurotomy have been recommended for treatment of medically intractable CGH [1]. A few theories have been proposed to explain the analgesic effect of BoNT. Some clinical trials of injecting BoNT near the targeted nerves have shown its effectiveness in pain relief [2,3].

However up to this point, there is no report regarding the effectiveness of BoNT when used in middle cervical medial branch block (MBB) for the treatment of CGH. We hereby report a case where BoNT was used in cervical MBB to treat cervicogenic headache.

**Case Report**

A 54 year-old male patient visited our pain clinic, complaining cervicogenic headache and neck pain. The C-spine MRI revealed the osteoarthritis at the facet joints of left C 3-4, 4-5 and 5-6. The MBB was performed at left C3, 4 and 5 under fluoroscopy (Figs.1, 2 and 3). 1.2 ml of 1% lidocaine was injected at each medial branch of C 3 to 5. The NRS for cervicogenic headache decreased from 6 to 3 after the block but without long lasting effect. After another trial of MBB with similar result, we decided to use botulinum toxin under the hypothesis that it would provide longer pain relief than diagnostic local anesthetics. 1.8ml of 1% lidocaine and BoNT (BOTOX® Type A, Allergan Inc., Irvine, CA, USA) 50 U were mixed to 1.8ml of normal saline, and 1.2ml of the mixture was injected at each level. The patient’s pain immediately decreased from NRS 6 to 3, and the effect lasted even after 3 months.

**Conclusion**

The use of botulinum toxin in middle cervical MBB may be effective in treating cervicogenic headache.

**Consent for publication:** The authors declare that written informed consent was obtained for publication.

**Fig. 1 (abstract P20).**
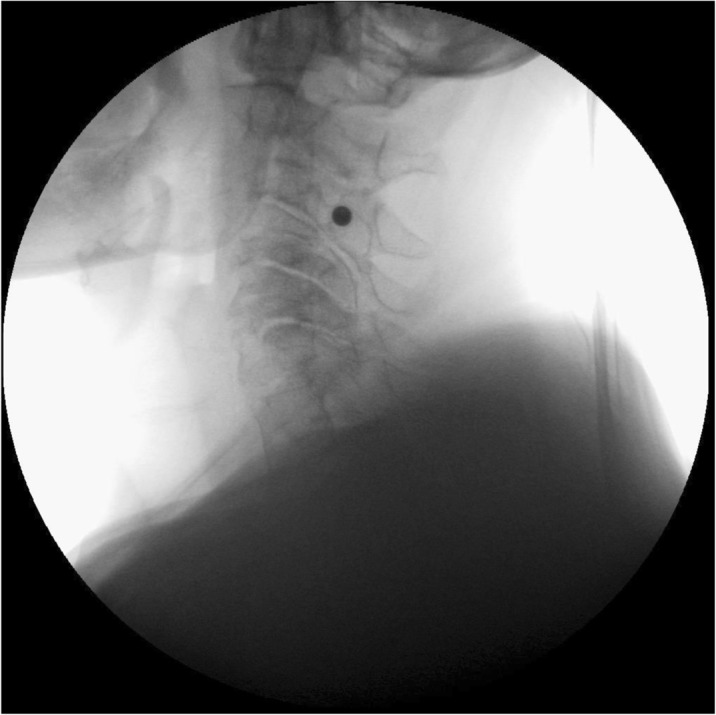
Fluoroscopic lateral view during cervical medial branch block at left C3, 4 and 5

**Fig. 2 (abstract P20).**
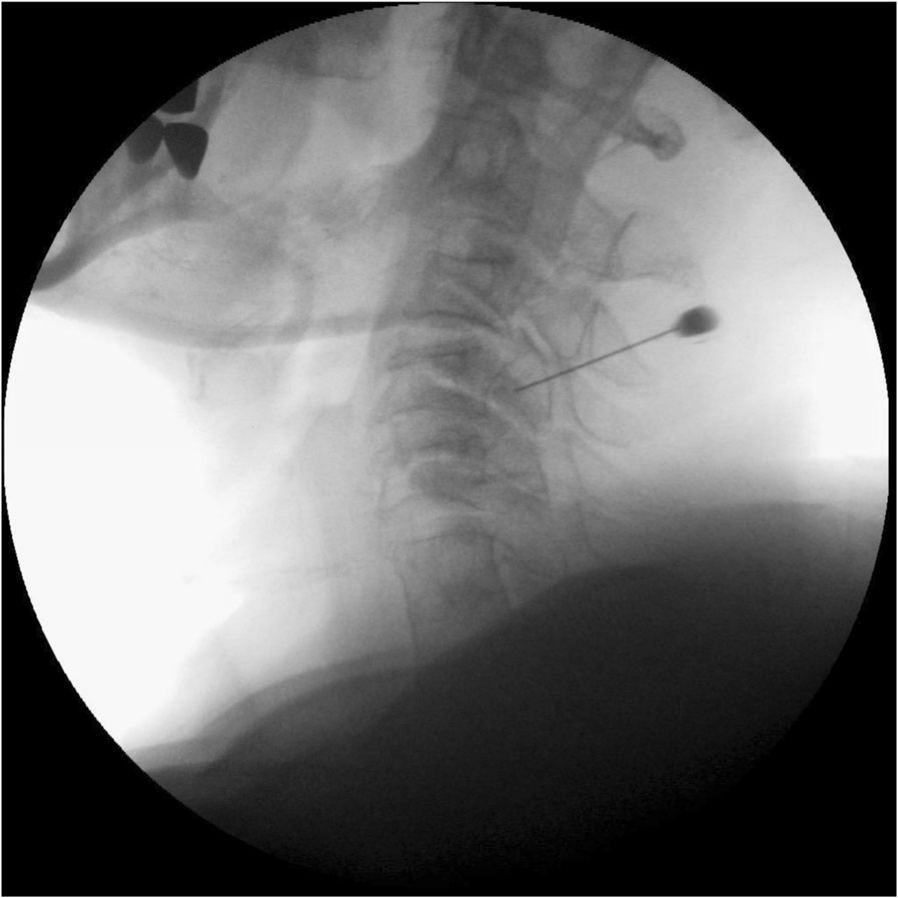
Fluoroscopic lateral view during cervical medial branch block at left C3, 4 and 5

**Fig. 3 (abstract P20).**
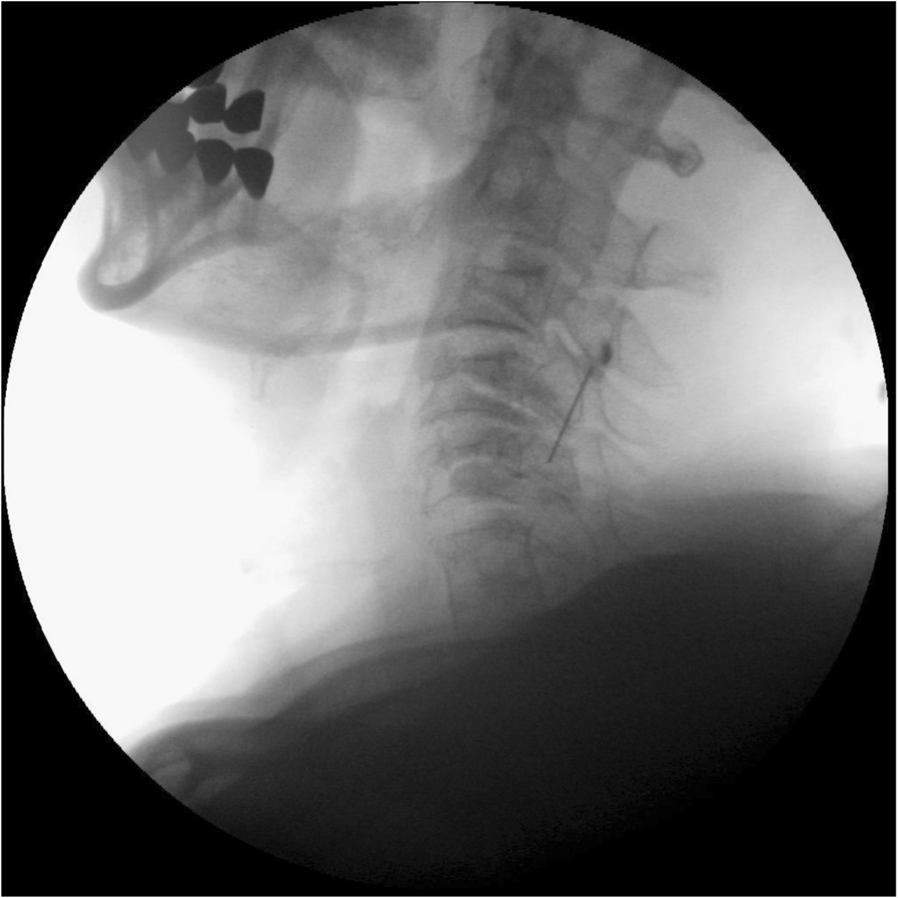
Fluoroscopic lateral view during cervical medial branch block at left C3, 4 and 5

**References**

1. Park SW, Park YS, Nam TK, Cho TG. The effect of radiofrequency neurotomy of lower cervical medial branches on cervicogenic headache. *J Korean Neurosurg Soc.* 2011; 50: 507-11.

2. Park JH, Park HJ. Botulinum toxin for the treatment of neuropathic pain. *Toxins* 2017; *9* : 260.

3. Kapural L, Stillman M, Kapural M, McIntyre P, Guirgius M, Mekhail N. Botulinum toxin occipital nerve block for the treatment of severe occipital neuralgia: a case series. *Pain pract.* 2007; 7: 337-40.

### P21 Chronic Migraine Treatment and Work Productivity

#### Valeria Canzonetta^1^, Valerio De Angelis^2^, Elena Rogante^1^, Eleonora Sabatelli^2^, Salvatore Sarubbi^1^, Alice Sparagna^1^, Denise Erbuto^1^, Marco Innamorati^1^, Maurizio Pompili^1^, Paolo Martelletti^2^

##### ^1^Department NESMOS, Sapienza Univeristy of Rome, Italy; ^2^Department of Clincal and Molecular Medicine, Sapienza University of Rome, Italy

###### **Correspondence:** Valeria Canzonetta

**Background**

Migraine is one of the most frequent neurological diseases. Specific features of migraine are moderate to severe pain associated with photophobia, phonophobia, nausea and/or vomit and it is worsened by physical activity. It lasts 4 to 72 hours [1]. Several studies showed that 2,5% of Episodic Migraines (EM) can evolve in Chronic Migraine (CM), characterized by high frequency of attacks (≥ 15days/month for more than 3 months with at least 8 attacks having the features of migraine headache) [2]. The pain intensity and the frequency may cause a chronic excessive intake of medication for acute or symptomatic treatment of migraine (triptans, AINS drugs, analgesics) leading to Medication Overuse Headache (MOH) [1].

Chronic migraine condition has a negative influence on many aspects of life, particularly on productivity at work. The American Migraine Prevalence and Prevention Study (AMPP) showed that migraine has a role both on absenteeism and presenteeism (reduced performance while at work) [3].

The present study is a preliminary analysis on a sample with CM treated with OnabotulinumtoxinA to examine work productivity in association with sociodemographic and psychosocial variables.

**Materials and methods**

104 patients with a diagnosis of chronic migraine, undergoing OnabotulinumtoxinA treatment (13 males; 91 females; mean age = 48,38 ± 9,85) have been enrolled in the Regional Referral Headache Centre of Sant'Andrea Hospital in Rome. Sample healthy control has been matched for age and sex.

All participants signed a consent form.

For this study the following tests have been used: Endicott Work Productivity Scale (EWPS, Endicott J., 1997) is a self-report questionnaire to evaluate work productivity through behaviours and feelings that are likely to reduce efficiency; Quality of Life Enjoyment and Satisfaction Questionnaire (Q-LES-Q, Endicott et al., 1993), Beck Depression Inventory (BDI, Beck AT, 1961), State and Trait Anxiety Inventory (Spielberger C, 1970); IPDQ (Innamorati et al., 2009).

Data have been analyzed using correlations and mean comparisons.

**Results**

There is no significant difference in work productivity between the clinical group (M = 17,22; ± 12,31) and healthy controls (M = 17,98; ± 11,7).

In the clinical sample there is a strong negative correlation among EWPS and Q-LES-Q scores (-,490: p<.000). Furthermore, there is a strong correlation between less work productivity and high scores in BDI (,445; p< .000), STAI (.499; p< .000), and IPDQ (.532; p< .000).

**Conclusions**

CM condition in patients undergoing treatment with OnabotulinumtoxinA is not associated to decreasing work productivity and efficiency.

**References**

1. Headache Classification Commitee of the International Headache Society (IHS). The International Classification of Headache Disorders 3^rd^ edition (β version). Cephalalgia. 2013;33:629-808.

2. Negro A, Curto M, Lionetto L, Guerzoni S, Pini LA, Martelletti P. A Critical Evaluation on MOH Current Treatments. Current Treatment Options in Neurology. 2017;19:32.

3. Stewart WF, Wood GC, Manack A, Varon SF, Buse DC, Lipton RB. Employment and work impact of chronic migraine and episodic migraine. Journal of occupational and environmental medicine. 2010;52:8-14.

### P22 A systematic literature review and meta-analysis of prevalence estimates in migraine

#### Akaterini Bilitou^1^, Pamela Vo^2^, Sandra Lopez-Leon^3^, Sneha Kelkar^4^, Claudia Cheung^4^, Emily Gao^5^, Keith A. Betts^6^, Zhou Zhou^5^

##### ^1^Novartis Global Services Centre, Patient Access Services, Dublin;^2^Novartis Pharma AG, Basel, Switzerland;^3^Novartis Pharmaceuticals Corporation, East Hanover, NJ, USA;^4^Analysis Group, New York/USA;^5^Analysis Group, Boston/USA;^6^Analysis Group, Los Angeles/USA

###### **Correspondence:**Akaterini Bilitou (katerina.bilitou@novartis.com)

**Introduction**

Reported epidemiological estimates in migraine vary widely across studies, mainly because of geographic variations, differences in study characteristics and diagnostic criteria used over time. This study aimed to identify and summarize population-based prevalence estimates of migraine in the last decade, after the revised International Classification of Headache Disorders (ICHD)-II definition.

**Methods**

A systematic literature review (SLR) of epidemiological studies in migraine published from 2006 until October 2016 from MEDLINE and EMBASE was conducted according to the Preferred Reporting Items for Systematic Reviews and Meta-Analyses (PRISMA) guidelines. In addition, conference abstracts published in the past 3 years (2014-2016) and references of most recent SLRs published were also reviewed. The quality of the extracted articles was evaluated using the modified Newcastle-Ottawa Scale for cohort studies. Random-effect models based on the DerSimonian and Laird method were used for meta-analysis of selected studies upon feasibility and quality assessment. The results were reported as forest plots displaying prevalence estimates and corresponding 95% confidence intervals (CI).

**Results**

A total of 1,133 records were identified and screened, and 77 publications reporting results for 51 unique studies met study criteria for data extraction (Fig. 1). In total 31 studies reported 1- year migraine prevalence, while 7 studies reported lifetime prevalence. Significant variation was observed between studies; ICHD-II was the most commonly used diagnostic criteria, while 25 out of 77 publications did not report the diagnostic criteria used. The estimated migraine prevalence by any definition was 14.2% (n=19, range 5.7% to 25.6%, 95% CI 13%-15.5%). The prevalence of migraine was higher among studies based on ICHD-II or later definitions (15.1%, 95% CI 13.6%-16.6%) than those using other migraine definitions (11.4%, 95% CI 10.4%-12.5%). The prevalence of episodic and chronic migraine were 13.6% (95%CI 10.4%-16.7%) and 0.9% (95%CI 0.32%-1.39%), respectively.

**Conclusion**

Despite marked heterogeneity of epidemiological studies across the literature, this study provided an updated systematic summary and evidence synthesis of the available prevalence estimates in migraine during the last decade.

**Fig. 1 (abstract P22.):**
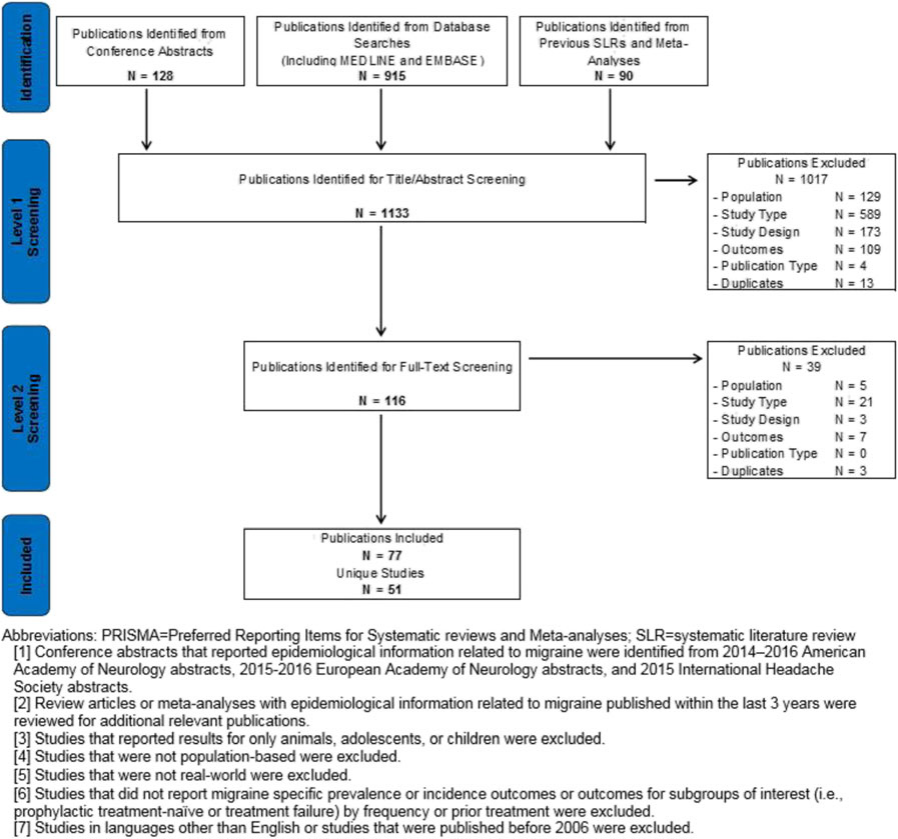
PRISMA diagram for the SLR of epidemiologic studies in migraine

**Competing interest**

This study was funded by Novartis AG, Switzerland.

### P23 Anxiety and Depression in Patients with Cluster Headache

#### Kwang-Yeol Park^1^; Soo-Jin Cho^2^; Byung-Kun Kim^3^; Pil-Wook Chung^4^; and Korean Cluster Headache Registry Group

##### ^1^Department of Neurolgy, Chung-Ang University, Seoul, Korea; ^2^Department of Neurology, Dongtan Sacred Heart Hospital, Hallym University, Hwaseong, Korea; ^3^Department of Neurolgy, Seoul Eulji Hospital, Eulji University, Seoul, Korea; ^4^Department of Neurology, Kangbuk Samsung Hospital, Sungkyunkwan University, Seoul, Korea

**Background**: While cluster headache is one of the most disabling pain, it has been less well studied compared with migraine. We hypothesized that, considering its severity and chronicity, cluster headache could bring about anxiety or depression, but the degree of psychological symptoms might be different between active attack period and remission period. Our aim was to investigate the association between cluster headache and anxiety or depression during with or without headache period.

**Methods**: This is a preliminary analysis of ongoing prospective registry enrolling patients with cluster headache from 17 clinics in South Korea since June 2016. The diagnosis of cluster headache was made according to the ICHD-III beta criteria. We assessed anxiety and depression using the Generalized Anxiety Disorder 7 (GAD-7) and Patient Health Questionnaire 9 (PHQ-9) scales. Control subjects without cluster headache were recruited from 3 outpatient clinics. Linear regression was performed to compare the GAD-7 and PHQ-9 scores between groups.

**Results**: A total of 78 patients (male 61, 78%) with cluster headache and 72 controls (male 60, 83%) were enrolled. Mean ages are 37 ± 11 years in cases and 37 ± 9.2 years in controls. GAD-7 scores were 7.9 ± 6.0 in cases during active attack period, 2.8 ± 3.4 in cases during remission period, and 3.4 ± 3.8 in controls. PHQ-9 scores were 7.7 ± 6.6 in cases during active attack period, 3.3 ± 4.4 in cases during remission period, and 3.7 ± 4.1 in controls. On multivariable analysis, compared with control, cluster headache was significantly associated with GAD-7 (beta 4.4; 95% confidence interval 2.7 to 6.0; p value < 0.001) and PHQ-9 (3.9; 2.1 to 5.6; <0.001). Also, compared with remission, active cluster headache was significantly associated with GAD-7 (4.7; 1.4 to 8.1), but if was marginally associated with PHQ-9 (3.2; -0.3 to 6.8; 0.07). However, compared with control, remission status of cluster headache was not associated with GAD-7 (-0.88; -3.1 to 1.3; 0.43) and PHQ-9 (-0.80; -3.2 to 1.7; 0.52).

**Conclusion**: Our study suggests that active attack of cluster headache is associated with increased anxiety and depression compared with remission status or control. However, remission period is not associated with anxiety or depression.

### P24 Benefit-Risk Assessment of Migraine Prophylaxis Treatments Using Likelihood of Being Helped or Harmed (LHH)

#### Pamela Vo^1^, Shihua Wen^1^, Marie-Josee Martel^2^, Dimos Mitsikostas^3^, Uwe Reuter^4^, Jan Klatt^1^

##### ^1^Novartis Pharma AG, Switzerland; ^2^Xcenda, UK; ^3^1st Neurology Department, Aeginition Hospital, National and Kapodistrian University of Athens, Greece; ^4^Department of Neurology, Charité Universitätsmedizin Berlin, Germany

###### **Correspondence:** Pamela Vo (pamela.vo@novartis.com)

**Background**

A key measure of successful therapy in migraine is the ability to sustain efficacy. Numerous prophylactic medications are available for this chronic disorder; however most of them have major shortcomings primarily due to variable efficacy and poor tolerability due to side effects. This study aimed to evaluate the benefit-risk of novel CGRP receptor antagonist erenumab, relative to other approved prophylactic migraine therapies.

**Materials and Methods**

Potential trials for inclusion were identified via a published systematic literature review [1] updated up to November 2016 using MEDLINE. As at the time of evaluation erenumab’s complete pivotal trial results were unpublished, clinical study reports were used (NCT02066415, NCT02456740). The ≥50% responder rates and discontinuations due to adverse events were defined as primary efficacy and tolerability variables to generate numbers needed to treat (NNT) and harm (NNH). The LHH as a quantitative benefit-risk measure was computed based on the ratio of NNH/NNT. Sensitivity analyses were conducted using alternative efficacy and tolerability data.

**Results**

Of 146 articles assessed, 9 RCTs (11 articles) met inclusion/exclusion criteria and were deemed of high quality per the Jadad score. Propranolol, topiramate, and onabotulinumtoxinA (the latter approved for CM only) were retained as comparators as they are approved for migraine prophylaxis and available in the majority of European countries. Table 1 shows an NNT of around 6 in both CM and EM for erenumab. This low NNT is numerically comparable to topiramate and onabotulinumtoxinA and show the strong treatment benefit of erenumab. NNH showed substantial differences among treatments, with higher numbers indicating better tolerability for erenumab. A favorable relative benefit-risk was seen for erenumab with LHHs of 41.7 and 166.7 for CM and EM respectively. In comparison, LHHs were lower in CM for topiramate (1.6 and 3.3) and onabotulinumtoxinA (4.3), and in EM, for topiramate (1.6) and propranolol (2.2). Sensitivity analyses showed results’ robustness despite residual variations and overall magnitude of LHH consistently favored erenumab.

**Conclusions**

While all prophylactic migraine treatments were more likely to help than harm (LHH > 1), erenumab showed LHHs of high magnitude, providing additional evidence to support the favorable benefit-risk profile of erenumab to patients across the entire spectrum of migraine compared with other migraine prophylactic treatments available in Europe.

**Table 1 (abstract P24).** NNT, NNH and LHH for CM and EM prophylactic treatments

	Chronic migraine (CM)				Episodic migraine (EM)		
	Erenumab 140 mg	Topiramate 100 mg		Onabotulinum-toxin A	Erenumab 140 mg	Topiramate 100 mg	Propranolol 160 mg
Data source :	NCT 02066415	Silberstein et al 2007 & 2009	Diener et al 2007	Dodick et al 2010	NCT 02456740	Bussone et al 2005	Diener et al 2004
NNT ≥50% RR (95%CI)	6 (4,12)	13 (NE, NE)	4 (3, 10)	9 (6, 15)	6 (4, 9)	5 (4, 6)	5 (4, 10)
NNH % d/c due to AEs (95%CI)	250 (NE, NE)	21 (NE, NE)	13 (NE, NE)	39 (23, 100)	1000^a^ (NE, NE)	8 (6, 13)	11 (6, 72)
LHH NNH/NNT (95%CI)	41.7 (5.3, 301.8)	1.6 (0.0, 112.8)	3.3 (0.7, 364.9)	4.3 (1.9, 11.4)	166.7 (9.0, 299.3)	1.6 (1.1, 3.1)	2.2 (0.8, 14.5)

*CI* confidence interval, *50% RR* 50% responder rate, *NE* not estimable (risk difference CI overlaps zero)

^a^Discontinuation rate (d/c) for erenumab was lower than placebo, yielding a negative absolute risk reduction. Conservative imputation of 0.1% absolute difference used to calculate erenumab’s NNH (otherwise not evaluable)

**References**

1. Jackson JL, Cogbill E, Santana-Davila R, Eldredge C, Collier W, Gradall A, et al. A Comparative Effectiveness Meta-Analysis of Drugs for the Prophylaxis of Migraine Headache. PLoS ONE. 2015; 10(7): e0130733.

**Funding**: This study was funded by Novartis AG, Switzerland.

**Acknowledgements**

This poster has been previously presented at the 18^th^ Congress of the International Headache Society, 7-10th September 2017, Vancouver, Canada.

### P25 Healthcare resource utilization among migraine sufferers in the EU5 from the patient perspective

#### Pamela Vo^1^, Aikaterini Bilitou^2^, Juanzhi Fang^3^, Annik Laflamme^1^, Shaloo Gupta^4^

##### ^1^Novartis Pharma AG, Switzerland; ^2^Novartis Global Services Centre, Ireland; ^3^Novartis Pharma, USA; ^4^Kantar Health, USA

###### **Correspondence:** Pamela Vo (pamela.vo@novartis.com)

**Objectives**

Migraine is a disabling neurological condition. The purpose of this study was to understand the incremental burden of migraine on healthcare resource utilization (HRU) in adults in Europe from the National Health and Wellness Survey (NHWS), a self-administered, internet-based questionnaire.

**Methods**

A retrospective, cross-sectional analysis of NHWS responses collected in 2016 from the EU5 (France, Germany, Italy, Spain, and UK) was performed. Adult (≥18 years old) respondents with a self-reported migraine diagnosis who completed the migraine module and with migraine experienced for ≥4 headache days in the past month were matched by propensity scores using sociodemographic characteristics to respondents without migraine (controls). HRU was evaluated via the number of healthcare provider (HCP) visits, emergency department (ED) visits and hospitalizations in the past six months. Mann-Whitney and Chi-square tests were used to determine significant differences between groups.

**Results**

Among respondents with migraine (≥4 headache days/month; n=218), 79.4% were female, the mean age was 43.25 years (SD = 13.48), and 60.1% were married or living with partner. Furthermore, 39.9% completed a university education and 62.4% were employed. Analysis of the propensity score-matched sample of 218 migraineurs and 218 controls showed that in the 6 months prior to questionnaire completion, the mean number of HCP visits (8.48 vs. 5.13, p<0.001) and ED visits (0.46 vs. 0.21, p=0.011) were significantly higher for migraine patients than those without migraine. The number of hospitalizations was higher among migraine patients (0.18 vs. 0.11, p=0.056) but marginally significant. Compared with matched controls, a significantly higher proportion of migraine respondents had ≥1 visits to a general/family practitioner (77.1% vs. 67.4%, p=0.025), neurologist (13.8% vs. 3.7%, p<0.001), and psychiatrist (13.3% vs. 3.2%, p<0.001) in the prior 6 months. The proportion of individuals with ≥1 ED visit was significantly higher for migraine patients than those without migraine (20.6% vs. 12.4%, p=0.02) whereas the proportion hospitalized (12.8% vs. 7.3% p=0.056) was slightly higher, but marginally significant.

**Conclusions**

Results demonstrated a statistically significant increase in HRU in terms of HCPs, neurologists, psychiatrists, and ED visits for migraine patients compared with non-migraine controls. To help reduce the burden of migraine on the European healthcare system better treatment options for migraineurs should be investigated.

**Table 1 (abstract P25).** Results on HRU after propensity score matched analysis with non-migraine controls across EU5

	Non-migraine controls (N=218)	Migraine (N=218)	p-value
Number of HCP visits in the past 6 months (mean, SD)	5.13 (6.86)	8.48 (10.89)	<0.001^a^
Number of ED visits in the past 6 months (mean, SD)	0.21 (0.79)	0.46 (1.20)	0.011^a^
Number of hospitalizations in the past 6 months (mean, SD)	0.11 (0.53)	0.18 (0.54)	0.056^a^
Visited General Practitioner/Family Practitioner in the past 6 months (n, %)	147 (67.4%)	168 (77.1%)	0.025^b^
Visited Neurologist in past 6 months (n, %)	8 (3.7%)	30 (13.8%)	< 0.001^b^
Visited Psychiatrist in past 6 months (n, %)	7 (3.2%)	29 (13.3%)	< 0.001^b^
Visited ED in the past 6 months (n, %)	27 (12.4%)	45 (20.6%)	0.020^b^
Hospitalized in the past 6 months (n, %)	16 (7.3%)	28 (12.8%)	0.056^b^

^a^Mann-Whitney test

^b^Chi-square test

**Funding**: This study was funded by Novartis AG, Switzerland.

**Acknowledgements**

This poster has been previously presented at the 18^th^ Congress of the International Headache Society, 7-10th September 2017, Vancouver, Canada.

### P26 Burden of migraine in the 5EU from the patient perspective: A cross-sectional analysis of National Health and Wellness Survey (NHWS) data

#### Pamela Vo^1^, Juanzhi Fang^2^, Aikaterini Bilitou^3^, Annik K. Laflamme^1^, Shaloo Gupta^4^

##### ^1^Novartis Pharma AG, Basel/Switzerland; ^2^Novartis Pharmaceuticals Corporation, NJ/USA; ^3^Novartis Global Services Centre, Patient Access Services, Dublin/Ireland; ^4^Kantarhealth, Princeton NJ/USA

###### **Correspondence:** Pamela Vo (pamela.vo@novartis.com)

**Introduction**

Migraine is one of the most disabling neurological conditions worldwide. The purpose of this study was to characterize the incremental burden of migraine on quality of life (QoL), productivity, and healthcare resource utilization (HRU) by the frequency of migraine in adults using European data from the National Health and Wellness Survey (NHWS), a self-administered, internet-based questionnaire.

**Methods**

A retrospective, cross-sectional analysis of responses from the 2016 NHWS was performed using data from the France, Germany, Italy, Spain, and UK (5EU). Adult NHWS respondents with a self-reported migraine diagnosis who completed the migraine module were matched by propensity scores to those without migraines (controls) using sociodemographic characteristics. Outcomes of interest analyzed were from EQ-5D, SF-36v2, HRU and the Work Productivity and Activity Impairment (WPAI) questionnaires. Migraine respondents were stratified by frequency of migraines (headache days/month): 4-7 episodic migraine (EM), 8-14 EM, and chronic migraine (≥15; CM). Independent sample t-tests were used to determine significant differences between controls and the frequency of migraine groups.

**Results**

Results from the propensity score matched analysis demonstrated that migraineurs reported statistically significant lower QoL and higher HRU as compared to their matched controls. Impairment while at work and total activity impairment was statistically significant higher among all migraineurs compared to matched controls (Table 1).

**Conclusion**

Migraine is a chronic disorder negatively affecting multiple domains of individuals’ lives. This study demonstrated that there is a statistically significant incremental burden due to migraine on QoL, HRU and work productivity amongst the migraineurs in comparison to matched controls.

### P27 Understanding the impact of migraine on work productivity using self-reported migraine diary data using the Migraine Buddy application in Europe

#### Nicolas Paris^1^, Pamela Vo^2^, Tomas Valena^3^, Aikaterini Bilitou^4^, Frederic de Reydet de Vulpillieres^2^, Juanzhi Fang^5^, Christel Naujoks^2^, Francois Everhard^2^, Francois Cadiou^1^

##### ^1^Healint Pte. Ltd, Singapore 118520; ^2^Novartis Pharma AG, Switzerland; ^3^Novartis s.r.o., Czech Republic; ^4^Novartis Global Services Center Dublin, Ireland; ^5^ Novartis Pharmaceuticals Corporation, East Hanover, NJ 07936-1080, USA

###### **Correspondence:** Aikaterini Bilitou (katerina.bilitou@novartis.com)

**Introduction**

The purpose of the study was to evaluate the impact of migraine on work productivity as perceived by individuals suffering from migraine in the real world using a self-reported smartphone application called Migraine-Buddy©.

**Methods**

A retrospective, cross-sectional analysis was conducted using data captured through Migraine-Buddy© from adult, self-diagnosed migraineurs in 17 European countries. Data were analyzed for the most recent 28-day period reported by migraineurs during the study period June 2015-July 2016. Data from chronic migraine (CM: ≥15 headache days/month, N=900), 4-7 episodic migraine (EM) (n=1500) and 8-14 EM (n=1500) individuals were randomly selected based on data completeness (fill rate >70%). Descriptive analysis was performed.

**Results**

A total of 10,347, 11,301 and 6,504 migraine records were retrieved from CM, 8-14 EM and 4-7 EM individuals, respectively corresponding to a total of 16,815, 14,398, and 7,693 migraine days. Among employed migraineurs (n=3,106) who declared ‘work’ either as their migraine location or in ‘affected activities’ at least once, an average of 57.4, 27.7 and 15.5 work days missed per year were estimated as reported by CM (n=730), 8-14 EM (n=1237) and 4-7 EM (n=1139) sufferers, respectively. The most commonly reported triggers of absenteeism-related migraines were psychological (38%), sleep (34%), nutrition (25%) and/or menstruation (23%). Employed sufferers reporting absenteeism recorded symptoms relating to pain/body, mood/cognition disturbances, environmental handicap and depression among others (Table 1).

**Conclusion**

Migraine is reported to have a considerable impact in the lives of affected individuals with symptoms impacting the work productivity of employed migraineurs irrespective of migraine frequency.

**Table 1 (abstract P27).** Symptoms reported by working migraineurs at least once in absenteeism-related migraine records. Number and proportion of users reporting each of the symptoms are shown (n=3106)

Symptoms	CM (n=730)	8-14EM (n=1237)	4-7EM (n=1139)	Total (n=3106)
Pain/Body	684 (94%)	1145 (93%)	1024 (90%)	2853 (92%)
Mood and cognition	661 (91%)	1114 (90%)	972 (85%)	2747 (88%)
Environmental handicap	643 (88%)	1073 (87%)	944 (83%)	2660 (86%)
Depression	436 (60%)	590 (48%)	435 (38%)	1461 (47%)
Sleep alterations	296 (41%)	435 (35%)	256 (22%)	987 (32%)
Others	271 (37%)	296 (24%)	213 (19%)	780 (25%)
No symptoms	126 (17%)	274 (22%)	161 (14%)	561 (18%)

Note: each user could specify more than one symptom per record and therefore numbers do not add up to 100%. Environmental handicap includes ringing in ears (tinnitus), sensitivity to light, noise or smell; mood and cognition symptoms include nausea, anxiety, confusion, blurred vision, moodiness, or giddiness. Analysis of record-level data showed consistent results with the user-level data analysis.

*CM* chronic migraine, *EM* episodic migraine

**Funding**: This study was funded by Novartis AG, Switzerland.

**Acknowledgements**

This poster has been previously presented at the 3^rd^ Congress of the European Academy of Neurology in Amsterdam, June 24-27, 2017.

### P28 A descriptive analysis of the burden of migraine based on self-reported migraine diary data using the Migraine Buddy application in Europe

#### Nicolas Paris^1^, Pamela Vo^2^, Tomas Valena^3^, Aikaterini Bilitou^4^, Frederic de Reydet de Vulpillieres^2^, Juanzhi Fang^5^, Christel Naujoks^2^, Francois Everhard^2^, Francois Cadiou^1^

##### ^1^Healint Pte. Ltd, Singapore 118520; ^2^Novartis Pharma AG, Switzerland; ^3^Novartis s.r.o., Czech Republic; ^4^Novartis Global Services Center Dublin, Ireland; ^5^Novartis Pharmaceuticals Corporation, East Hanover, NJ 07936-1080, USA

###### **Correspondence:** Aikaterini Bilitou (katerina.bilitou@novartis.com)

**Introduction**

Migraine is a neurological disorder that can cause severe disabling pain. The purpose of the study was to describe the burden of migraine on health-related quality of life (HRQOL) as perceived by individuals suffering from migraine in the real world using a self-reported mobile application.

**Methods**

A retrospective, cross-sectional analysis was conducted using data captured through the Migraine-Buddy© smartphone application from adult, self-diagnosed migraineurs in several European countries including the UK, France, and Spain. Data were analyzed for the most recent 28-day period reported by migraineurs during the study period (June 2015-July 2016). Migraine respondents (n=3900) were randomly selected based on data completeness (fill rates >70%) and stratified by migraine headache days/month: 4-7 episodic migraine (EM) (n=1500), 8-14EM (n=1500), and chronic migraine (≥15; CM) (n=900). Descriptive analysis was performed.

**Results**

More than 95% of 3900 self-reported migraineurs reported that migraine negatively impacted their daily activities in at least one migraine attack. Attacks were estimated to affect 50.5% (184.4 days/year), 26.9% (98 days/year) and 14.5% (53 days/year) of their calendar year among CM, 8-14EM, and 4-7EM groups, respectively. On average, 44.8% CM, 40.9% 8-14EM and 34.7% of 4-7EM sufferers respectively reported anxiety and/or depression symptoms during migraine attacks. Social or home activities, productivity, or sleep were highly impacted in migraineurs (Table 1). Triptans (31.9%), nonsteroidal anti-inflammatory drugs (28.7%), acetaminophen (18.9%) and opioids (8.4%), and were self-reported as the most common medicines used by migraineurs across migraine records (n=28152).

**Conclusion**

This study highlights the high burden of migraine on HRQOL and overall well-being of individuals suffering from migraines irrespective of migraine frequency.

**Funding**: This study was funded by Novartis AG, Switzerland.

**Table 1 (abstract P28).** Impact of migraine on daily activities as reported by migraineurs in at least one of their migraine records. Number and proportion of users, by migraine frequency and overall, reporting type of activity affected is shown

Type of activity affected	CM (n=900)	8-14 EM (n=1500)	4-7 EM (n=1500)	Total (n=3900)
Home activities	520 (58%)	985 (66%)	933 (62%)	2438 (63%)
Productivity	590 (66%)	993 (66%)	841 (56%)	2424 (62%)
Social activities	553 (61%)	882 (59%)	736 (49%)	2171 (56%)
Sleep	470 (52%)	827 (55%)	676 (45%)	1973 (51%)
Others	268 (30%)	298 (20%)	204 (14%)	770 (20%)
Any activity	855 (95%)	1447 (96%)	1430 (95%)	3732 (96%)

Others include affected activities that do not fit in the displayed categories. Migraine Buddy users could specify more than one activity affected in their records; pooled results across all migraine records are presented for the most recent 28-day period reported by migraine patients during the study (June 2015-July 2016)

*CM* Chronic Migraine, *EM* Episodic Migraine

**Acknowledgements**

This poster has been previously presented at the 3^rd^ Congress of the European Academy of Neurology in Amsterdam, June 24-27, 2017.

### P29 Evaluating clinically meaningful within-subject change in functioning associated with migraine prevention using the Migraine Physical Function Impact Diary (MPFID)

#### Ariane K Kawata^1^, Asha Hareendran^2^, Jiat-Ling Poon^1^, Andrew Thach^3^, Pooja Desai^3^, Yumi Kubo^3^, Daniel D Mikol^3^, David W Dodick^4^, Richard B Lipton^5^, Stewart J Tepper^6^

##### ^1^Evidera, Bethesda, MD, USA; ^2^Evidera London, UK; ^3^Amgen Inc., Thousand Oaks, CA, USA; ^4^Department of Neurology, Mayo Clinic Arizona, Phoenix, AZ, USA; ^5^Montefiore Medical Center, Albert Einstein College of Medicine, Bronx, NY, USA; ^6^Geisel School of Medicine at Dartmouth, Department of Neurology, Dartmouth-Hitchcock Medical Center, Lebanon, NH, USA

###### **Correspondence:** Ariane K Kawata (ariane.kawata@evidera.com)

**Background**

Monthly Migraine Physical Function Impact Diary (MPFID) domain scores (Impact on Everyday Activities [EA, 7 items] and Physical Impairment [PI, 5 items]) range from 0–100 (higher score=greater impact). A Global Impact on Everyday Activities score (G-EA) is generated from a single item. We report a clinically meaningful within-patient change (CMWPC) in migraine impact scores and evaluation of CMWPCs in the STRIVE study (NCT02456740) in subjects with episodic migraine (EM).

**Methods**

CMWPCs for MPFID were developed using anchor- and distribution-based methods using data pooled across treatment groups from the ARISE study (NCT02483585) and an observational study of patients who recently initiated or changed their migraine preventive regimen. Clinically relevant anchor variables (≥30% and ≥50% reduction in monthly migraine days [MMD] and ≥20% and ≥50% reduction in MPFID G-EA) were used to estimate average within-subject point change from baseline in MPFID domain scores; distribution-based estimates based on variability were considered supportive. These CMWPCs were used to examine the proportion of responders to treatment in a post-hoc analysis of the STRIVE study. Cumulative distribution function (CDF) plots demonstrated percentage of subjects within each treatment group achieving the range of CMWPCs from baseline in MPFID domain scores.

**Results**

Estimates from the ARISE study and observational study suggested that CMWPCs starting at 3-point change in MPFID EA and PI domains represented CMWPC. In the STRIVE study, larger proportions of erenumab-treated subjects than placebo achieved a ≥5-point reduction (pre-specified endpoint) from baseline to mean of weeks 13–24 in PI (140mg: 42.5%; 70mg: 39.1% vs placebo: 30.1%, both p<0.05) and EA domain scores (140mg: 50.3%; 70mg: 49.0% vs placebo: 34.5%, both p<0.001). The CDF plots showed that more subjects in 140mg and 70mg erenumab groups than placebo had greater reductions in EA and PI domain scores. The erenumab groups had consistently larger proportions of responders than placebo starting as low as a 3-point change from baseline score and across a range of CMWPCs.

**Conclusion**

Reductions starting at 3 points in MPFID domains are representative of CMWPCs. Treatment with erenumab 140mg and 70mg in the STRIVE study was related to clinically meaningful reductions in the impact of migraine on physical functioning versus placebo, based on greater proportions on erenumab experiencing within-subject change ≥5 points. This supports the utility of MPFID as a marker for migraine clinical benefit and demonstrates the value of erenumab as a preventive therapy to improve functioning in subjects with EM.

### P30 The Trigger Avoidance Model of Headaches and Learning to Cope with Triggers: An update

#### Paul R Martin (paul.martin@griffith.edu.au)

##### School of Applied Psychology and Menzies Health Institute Queensland, Griffith University, Mount Gravatt, Queensland, Australia, 4122

**The Trigger Avoidance Model of Headaches**

The *Trigger Avoidance Model of Headaches* (TAMH) has been proposed to explain how triggers acquire the capacity to precipitate headaches [1,2,3]. The Model is based on the theory that a critical process in the development of a headache disorder may be trying to avoid the triggers resulting in a sensitisation process such that tolerance for triggers diminishes. The conceptual underpinning of the TAMH comes from cognate literatures such as the anxiety literature as there is much evidence that efforts to avoid stimuli/situations that elicit anxiety resulting in short exposure to those stimuli, will lead to those stimuli eliciting more anxiety in the future. Treatment of anxiety disorders is based on the reverse principle of prolonged exposure to anxiety-eliciting situations, leading to desensitisation. The TAMH has received support in a series of laboratory investigations that demonstrate short exposure to headache triggers results in sensitisation, whilst prolonged exposure leads to desensitisation [4,5,6,7].

**Learning to Cope with Triggers**

Advice to avoid triggers as a means of preventing headaches, has been the standard for decades. The implication of the TAMH is that such advice may be counterproductive for some triggers as it could lead to reduced tolerance for those triggers. Based on the TAMH, it has been argued that counselling avoidance should be replaced with a philosophy of *Learning to Cope with Triggers* (LCT) [1,2,3]. LCT specifies use of exposure-based strategies for triggers such as stress, negative affect, and sensory triggers (visual disturbance, noise); and avoidance strategies for triggers that are not consistent with a healthy lifestyle (e.g., hunger, dehydration, lack of sleep). LCT has been evaluated in a randomised controlled trial with the following outcomes [8]. Changes in headaches and medication consumption (in parentheses) from pre- to post-treatment were (a minus sign indicates improvement): Waiting-list, +11.0% (+15.4%); Avoidance, -13.2% (-9.0%); and LCT, -35.9% (-27.9%). Avoidance did not differ significantly from Waiting-list for headaches or medication use, but LCT differed significantly from Waiting-list for both measures. Three illustrative case studies of LCT have since been published describing the use of exposure techniques with the triggers of stress/anger, tiredness and heat [9]. Coping with triggers is now beginning to achieve recognition in the literature. For example, the European Federation of Neurological Societies guidelines on treatment of tension-type headache include “Identification of trigger factors should be performed, as coping with trigger factors may be of value (Martin & MacLeod, 2009)” [10].

**References**

1. Martin PR, MacLeod C. Behavioral management of headache triggers: Avoidance of triggers is an inadequate strategy. Clinical Psychology Review. 2009; 29:483-495.

2. Martin PR. Managing headache triggers: Think ‘coping’ not ‘avoidance’. Cephalalgia. 2010; 30:634-637.

3. Martin PR. Behavioral management of migraine headache triggers: Learning to cope with triggers. Current Pain and Headache Reports. 2010; 14:221-227.

4. Martin PR. How do trigger factors acquire the capacity to precipitate headaches? Behaviour Research and Therapy. 2001; 39:545-554.

5. Martin PR, Reece J, Fordyce M. Noise as a trigger for headaches: Relationship between exposure and sensitivity. Headache. 2006; 46:962-972.

6. Martin PR, Lae L, Reece J. Stress as a trigger for headaches: Relationship between exposure and sensitivity. Anxiety, Stress and Coping. 2007; 20:393-407.

7. Martin PR. Headache triggers: to avoid or not to avoid, that is the question. Psychology and Health. 2000; 15:801-809.

8. Martin PR, Callan M, Reece J, MacLeod C, Kaur A, Gregg K, Goadsby PJ. Behavioral management of the triggers of recurrent headache: A randomized controlled trial. Behaviour Research and Therapy. 2014; 61:1-11.

9. Martin PR, Callan M, Kaur A, Gregg K. Behavioral management of headache triggers: Three case examples illustrating a new effective approach (Learning to Cope with Triggers). Behaviour Change. 2015; 32:202-208.

10. Bendtsen L et al. EFNS guideline on the treatment of tension-type headache – Report of an EFNS task force. European Journal of Neurology. 2010; 17:1318-1325.

### P31 The intermediary relationship between analgesic/abortive medication use, sleep quality, and headache frequency: A potential new partial mechanism contributing to medication overuse headache

#### Daniel P Sullivan, Paul R Martin

##### School of Applied Psychology and Menzies Health Institute Queensland, Griffith University, Mount Gravatt, Queensland, Australia, 4122

###### **Correspondence:** Daniel P Sullivan (daniel.sullivan5@griffithuni.edu.au)

**Background**

Evidence that sleep dysfunction can play a role in headaches has been well established. We have previously reported various sleep factors are correlates of headaches, particularly sleep quality [1]. Medication overuse headache develops when acute or symptomatic headache medications are used too frequently. Given commonly used medications such as NSAIDs and opioids disrupt sleep architecture [2, 3], we sought to test the hypothesis that greater use of acute medications would be associated with more frequent headaches, as mediated by sleep quality.

**Method**

Participants (*n* = 370, 85% female) were recruited through headache and pain websites, and University recruitment channels. Utilising a cross-sectional design, participants completed a battery of online measures including headache type/frequency and use of headache analgesic/abortive medications, and sleep variables such as sleep quality and duration.

**Results**

Regression models found medication use (MU) and sleep quality (SQ) to be significant predictors of migraine frequency, *F* (2, 367) = 78.21, *p* < .001, *R* ^*2*^ = .3; and non-migraine headache frequency, *F* (2, 367) = 98.67, *p* < .001, *R* ^*2*^ = .35. Mediation analysis revealed MU had a significant direct effect on migraine (Predictor coefficient = .417, *p* < .001), which was mediated by SQ (Mediator coefficient =.048, 95%CI .023, .083, κ^2^ = .061). A significant direct effect for MU was found for non-migraine headaches (PC = .524, *p* < .001), and that effect was also mediated by SQ (MC = .03, 95%CI .006, .06, κ^2^ = .039).

**Conclusions**

This paper provides a foundation for future investigations using stronger prospective designs to examine if medication overuse headache is partly mediated by the deleterious effect some medications may have on sleep architecture and quality. Such studies may employ prospective designs and sophisticated laboratory measurements rather than self-report. Another avenue for investigation is whether the effect of headache relieving medications can be augmented with adjunctive therapy for sleep (e.g., Melatonin).

**Fig. 1 (abstract P31).**
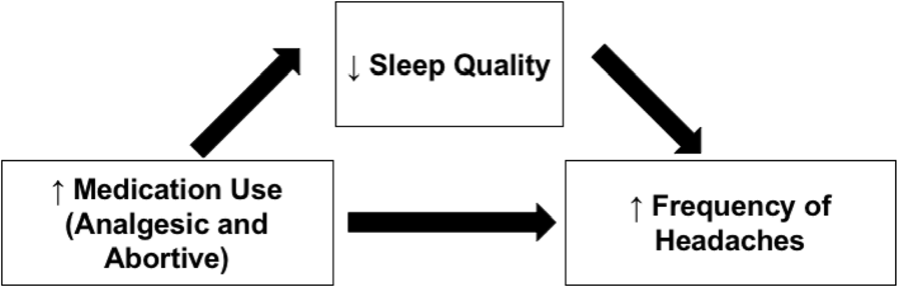
Graphical representation of the mediatory relationship between medication and sleep quality, and headache

**References**

1. Sullivan DP, Martin PR: Sleep and headaches: Relationships between migraine and non-migraine headaches and sleep duration, sleep quality, chronotype, and obstructive sleep apnoea risk. Australian Journal of Psychology. 2017; 69:210-217.

2. Murphy PJ, Badia P, Myers BL, Boecker MR, Wright KP, Jr.: Nonsteroidal anti-inflammatory drugs affect normal sleep patterns in humans. Physiol Behav. 1994; 55:1063-1066.

3. Dimsdale JE, Norman D, DeJardin D, Wallace MS: The effect of opioids on sleep architecture. J Clin Sleep Med. 2007; 3:33-36.

### P32 State and Trait anger and its expression in migraine and cluster headache

#### Marialuisa Rausa^1,3^, Sabina Cevoli^2^, Giulia Giannini^1^, Valentina Favoni^1^, Sara A Contin^3^,Donatella Ballardini^3^, Pietro Cortelli^1,2^, Giulia Pierangeli^1,2,3^

##### ^1^Department of Biomedical and Neuromotor Sciences DIBINEM, University of Bologna, Bologna,40100, Italy; ^2^IRCCS Istituto delle Scienze Neurologiche di Bologna, 40100, Italy; ^3^Centro Gruber. Service for the Diagnosis and Treatment of Eating Disorders. Service for the Diagnosis and Treatment of Anxiety and Psychosomatic Disorders, Bologna,40100, Italy

###### **Correspondence:** Marialuisa Rausa (m.rausa@gmail.com)

**Background:**

Negative affective states, like anger and fear, are deeply involved in the emotional experience of pain [1]. It is reported that individuals with migraine and tension type headache are more likely to hold their anger-in than controls. One study showed that headache patients hold their anger-in more than controls, even after controlling for depression and anxiety [2]. However, no study evaluated anger in cluster headache (CH) and differences between migraine (M) and CH patients. The objective is to evaluate differences between M and CH patients in anger levels and in anger expression.

**Materials and Methods:**

134 M patients and 105 CH patients were administered the State Trait Anger Expression Inventory (STAXI-2, 56 items), composed by seven subscales. State Anger refers to the intensity of the individual’s angry feelings at the time of testing. Trait Anger evaluates person’s general predisposition to become angry. Anger expression is measured by: Anger Expression Out (the extent to which anger could be expressed in an outwardly manner), Anger Expression In (the extent to which anger is suppressed), Anger Control Out (prevent explosive manifestations of anger), Anger Control In (try to relax and reduce angry feelings) and Anger Expression index (an overall index of the individual tendencies to express anger).

**Results:**

CH patients have higher scores than M patients (p<0.05) in State Anger. Moreover CH patients have higher scores in Anger Expression-Out (p<0.05). No differences were found in trait anger subscales (tab.1). In particular, in sub-group analysis, patients with CH during cluster period have higher state anger than chronic migraine patients, while CH patients in headache free period did not differ from M patients.

**Conclusions:**

The results indicate that M and CH patients differ in state anger, but not in trait anger, suggesting that there are no dispositional differences in anger feeling. In CH, especially during cluster period, was detected higher intensity of anger feeling during the time of testing. This data support the bio-behavioral hypothesis of different behavioral response to pain in M and CH patients (sickness behavior vs defense reaction) [3], and add new information about emotional regulation involved during headache’s attack.

**Table 1 (abstract P32).** Mean scores at Staxi-2

STAXI-2	Migraine patients (n=134)	Cluster headache patients (n=105)	p
State Anger	17,51	20,11	P<0.05
Trait Anger	5,42	6,15	ns
Anger Expression index	46,27	47,29	ns
Anger Expression in	20,25	19,76	ns
Anger Expression out	16,99	18,32	P<0.05
Anger Control in	18,48	18,79	ns
Anger Control out	20,48	20,00	ns

**References**

1. Venable VL, Carlson CR, Wilson J. The role of anger and depression in recurrent headache. Headache 2001;41:21–30.

2. Nicholson RA, Gramling SE, Ong JC, Buenevar L: Differences in anger expression between individuals with and without headache after controlling for depression and anxiety. Headache 2003;43:651-663.

3. Montagna P, Pierangeli G, Cortelli P.The primary headaches as a reflection of genetic darwinian adaptive behavioral responses. Headache. 2010 Feb;50(2):273-89. Epub 2009 Dec 21.

### P33 A Phase 1 Study to Assess the Pharmacokinetics, Safety, Tolerability and immunogenicity of Fremanezumab doses (225 mg, 675 mg and 900 mg) in Japanese and Caucasian Healthy Subjects

#### Orit Cohen-Barak^1^, Xiaojun Hu^1^, Michele Rasamoelisolo^1^, Nicola Faulhaber^1^, Paul Yeung1, Esther Yoon^2^, Mohit Gandhi ^3^, Ernesto Aycardi^1^

##### ^1^Global Research and Development, Teva Pharmaceutical Industries, Netanya, Israel; ^2^PAREXEL International, Los Angeles; ^3^PRA Health Sciences, Lenexa, United States

###### **Correspondence:** Orit Cohen-Barak

**Objectives**

Fremanezumab is a fully humanized IgG2Δa monoclonal antibody that selectively blocks CGRP isoforms (α- and β) from binding to the CGRP receptor. Fremanezumab was effective and well-tolerated as a preventive treatment of episodic migraine and chronic migraine in phase 2 and phase 3 trials. The present study evaluated the pharmacokinetic profile, safety, and immunogenicity of fremanezumab doses tested in the phase 2 and 3 trials (225mg, 675mg and 900mg) following single administration in Japanese (n=32) and Caucasian (n=32) healthy subjects.

**Methods**

Japanese and Caucasian healthy subjects were enrolled into 1 of 4 cohorts: cohorts 1 and 3 were Japanese and cohorts 2 and 4 were Caucasians. Subjects in each cohort were randomly assigned to 1 of 4 treatments: 225, 675, or 900 mg fremanezumab, or placebo. In the first cohort only, a dose escalation scheme was applied where study drug was not escalated to the next dose level unless the safety and tolerability of the previous doses were acceptable by sponsor and clinical team. Caucasian subjects were matched to Japanese subjects based on gender, age (± 10 year) and BMI (±20%). PK and immunogenicity sampling and safety & tolerability assessments occurred during 13 clinic visits including 1 inpatient visit from day -1 to day 6 and 12 ambulatory visits between post treatment days 8-225.

**Results**

Sixty-two subjects out of 64 completed the study; 2 Japanese subjects (1 225mg and 1 900mg fremanezumab) withdrew consent because of family emergencies. Overall median Tmax was similar across doses and ranged from 5 to 7 days. Mean half-lives were similar across doses (range 32.23 to 36.15 days). No differences due to race/ethnicity. Increases in Cmax and AUCs were slightly greater than dose proportional for both Japanese and Caucasian subjects. Fremanezumab exposures were generally higher with lower body weights. No deaths or SAEs; most frequently occurring AEs (≥2 subjects) were injection site reactions, abdominal pain, headache, upper respiratory tract infection, constipation and nasopharyngitis. Local tolerability of the SC fremanezumab injection was comparable between Japanese and Caucasian subjects. No treatment-induced anti-drug-antibodies occurred and there were no clinically meaningful changes in laboratory findings.

**Conclusion**

Overall fremanezumab was safe and well tolerated following SC single doses (225, 675, or 900mg). Pharmacokinetic exposure parameters per dose were similar for Japanese and Caucasians. Half-life following SC injections support the once monthly SC injections of 225mg and quarterly SC injections of 675mg as a treatment doses.

**Trial registration**

Clinicaltrials.gov NCT02673567

**Competing Interest**

O. Cohen-Barak Conflict with: Teva Pharmaceutical Industries, Conflict with: Teva Pharmaceutical Industries, X. Hu Conflict with: Teva Pharmaceutical Industries, M. Rasamoelisolo Conflict with: Teva Pharmaceutical Industries, N. Faulhaber Conflict with: Teva Pharmaceutical Industries, P. Yeung Conflict with: Teva Pharmaceutical Industries, Conflict with: Teva Pharmaceutical Industries, E. Yoon Conflict with: PAREXEL International, M. Gandhi Conflict with: PRA Health Sciences, E. Aycardi Conflict with: Teva Pharmaceutical Industries, Conflict with: Teva Pharmaceutical Industries

### P34 Fremanezumab increases the maximum number of consecutive headache free days for patients with high frequency episodic migraine

#### Robert Noble, Ernesto Aycardi, Marcelo Bigal, Mirna McDonald, Pippa Loupe, Investigators of the EM study

##### ^1^Statistics, Teva Global Medical Affairs, Hamilton; ^2^Global Clinical Development; ^3^Clinical Development, Teva Global Research and Development, Frazer; ^4^Academic Affairs and Network, Teva Global Research and Development, Overland Park , United States

**Background**

Fremanezumab is a fully humanized IgG2Δa monoclonal antibody found to be effective and well-tolerated as a preventive treatment for migraine in phase 2 and 3 studies. Previously reported data has shown that on headache free days, patients on fremanezumab treatment had greater number of days in which they were able to work/study normally, less time with difficulty in concentration, and more time engaged and interested in daily activities. The present report describes a Bayesian model performed to evaluate the number of consecutive headache free days in the high frequency episodic migraine (HFEM) study.

**Methods**

This was a randomized 3-month phase 2 study comparing two doses of fremanezumab (225 mg and 675 mg) with placebo. During a 28 day run-in period, patients were screened and trained to capture daily headache information using an electronic headache diary. Following the run-in period, patients who were 80% compliant in diary entry and had migraine headaches at least 8 but less than 14 days per 28 days were randomized and treated subcutaneously once every 28 days with either 225 mg or 675 mg fremanezumab or placebo. Compared to placebo, both doses of fremanezumab significantly reduced the primary endpoint of the HFEM study, change in the number of migraine days at month 3 relative to baseline; herein we assessed in a post-hoc analysis whether there were differences in the maximum number of consecutive headache free days among the treatment arms. Due to the skewed nature of the distribution of maximum headache free days, the data were modeled assuming a geometric likelihood. Bayesian model using non-informative priors on the headache rate was used.

**Results**

In the posterior distributions for the maximum number of consecutive headache free days (Fig. 1), there is a separation between the curves for participants taking fremanezumab 225mg (n=96) and 675mg (n=97) compared to those taking placebo (n=104). Posterior probabilities and 90% credible intervals for the differences between the fremanezumab 225 mg arm and placebo were 0.965 [0.316,6.709] and for fremanezumab 675 mg arm vs placebo 0.993 [1.553,8.322]. Patients in the fremanezumab 225 mg group had mean(SD) maximum number of consecutive headache free days of 14.5(15.0), 675 mg group experienced 15.9(14.8) days and the placebo group 11.12(10.6) days.

**Conclusion**

The results of this post-hoc analysis suggest that HFEM patients taking fremanezumab at doses of 225mg and 675mg can expect to have a greater maximum number of consecutive headache free days than patients on placebo.

**Trial registration**

Clinicaltrials.gov NCT02025556

**Competing Interest**

Fremanezumab HFEM Study supported by Teva Pharmaceutical Industries Global Research and Development, Netanya Israel. RN, EA, MEB, and PL are employees of Teva Pharmaceutical Industries, Israel.

### P35 A Phase 1 Study to Assess the Pharmacokinetics, Safety, Tolerability and immunogenicity of Fremanezumab doses (225 mg, 675 mg and 900 mg) in Japanese and Caucasian Healthy Subjects

#### Orit Cohen-Barak, Xiaojun Hu, Michele Rasamoelisolo, Nicola Faulhaber, Paul Yeung, Esther Yoon, Mohith Gandhi and Ernesto Aycardi

**Background**. Fremanezumab (formerly TEV-48125) is a fully humanized IgG2Δa monoclonal antibody that selectively blocks both CGRP isoforms (α- and β) from binding to the CGRP receptor. Fremanezumab was effective and well-tolerated as a preventive treatment of episodic migraine and chronic migraine in phase 2 and phase 3 trials. The present study evaluated the pharmacokinetic profile, safety, and immunogenicity of fremanezumab doses tested in the phase 2 and 3 trials (225mg, 675mg and 900mg) following single administration in Japanese (n=32) and Caucasian (n=32) healthy subjects.

**Methods.** Japanese and Caucasian healthy subjects were enrolled into 1 of 4 cohorts: cohorts 1 and 3 were Japanese and cohorts 2 and 4 were Caucasians. Subjects in each cohort were randomly assigned to 1 of 4 treatments: 225, 675, or 900 mg fremanezumab, or placebo. In the first cohort only, a dose escalation scheme was applied where study drug was not escalated to the next dose level unless the safety and tolerability of the previous doses were acceptable by sponsor and clinical team. Caucasian subjects were matched to Japanese subjects based on gender, age (± 10 year) and BMI (±20%). PK and immunogenicity sampling and safety & tolerability assessments occurred during 13 clinic visits including 1 inpatient visit from day -1 to day 6 and 12 ambulatory visits between post treatment days 8-225.

**Results**: Sixty-two subjects out of 64 completed the study; 2 Japanese subjects (1 225mg and 1 900mg fremanezumab) withdrew consent because of family emergencies. Overall median T_max_ was similar across doses and ranged from 5 to 7 days. Mean half-lives were similar across doses (range 32.23 to 36.15 days). No differences due to race/ethnicity. Increases in C_max_ and AUCs were slightly greater than dose proportional for both Japanese and Caucasian subjects. Fremanezumab exposures were generally higher with lower body weights. No deaths or SAEs; most frequently occurring AEs (≥2 subjects) were injection site reactions, abdominal pain, headache, upper respiratory tract infection, constipation and nasopharyngitis. Local tolerability of the SC fremanezumab injection was comparable between Japanese and Caucasian subjects. No treatment-induced anti-drug-antibodies occurred and there were no clinically meaningful changes in laboratory findings.

**Conclusions**. Overall fremanezumab was safe and well tolerated following SC single doses (225, 675, or 900mg). Pharmacokinetic exposure parameters per dose were similar for Japanese and Caucasians. Half-life following SC injections support the once monthly SC injections of 225mg and quarterly SC injections of 675mg as a treatment doses.

**Fig. 1 (abstract P35).**
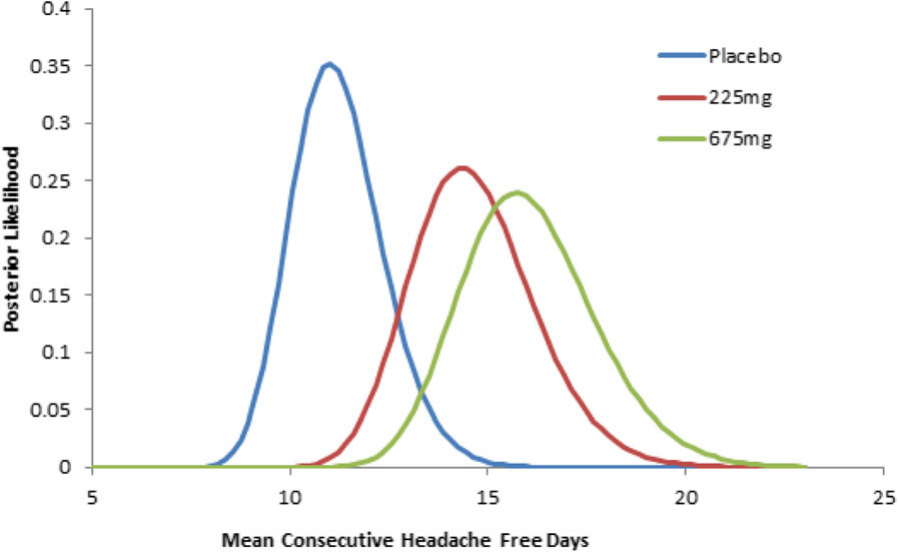
Posterior Mean Maximum Headache Free Day Distrivutions

### P36 Analysis of injection site reactions across four placebo controlled trials of erenumab for migraine prevention

#### Julio Pascual^1^, David Doležil^2^, Brendan Davies^3^, Hernan Picard^4^, Frank Hong^5^, Feng Zhang^4^, Fei Xue^4^, Dan Mikol^4^, Jan Klatt^5^

##### ^1^Service of Neurology, University Hospital Marqués de Valdecilla and IDIVAL, Santander, Spain; ^2^DADO MEDICAL sro, Prague Headache Center, Prague, Czech Republic; ^3^Department of Neurology, Royal Stoke University Hospital, Stoke-on-Trent, UK; ^4^Amgen Inc., Thousand Oaks, CA, USA; ^5^Novartis Pharma AG, Basel, Switzerland

###### **Correspondence:** Julio Pascual (juliopascual@telefonica.net)

**Background**

Erenumab is a fully human monoclonal antibody that selectively inhibits the calcitonin gene-related peptide (CGRP) receptor under investigation for migraine prevention. Erenumab is administered monthly by subcutaneous injection. Here we report the incidence of injection site reaction-related adverse events (ISR-AEs) observed in erenumab clinical trials in subjects with episodic or chronic migraine.

**Methods**

Data were obtained from four placebo-controlled clinical trials (clinicaltrials.gov NCT01952574, NCT02066415/NCT02174861, NCT02456740, NCT02483585). Analysis was performed for two periods: the 12-week double-blind placebo-controlled treatment period (DBTP; erenumab and placebo) and the entire erenumab exposure period (EEP), including the open-label extension phase (erenumab only). AEs were graded according to Common Terminology Criteria Version 4.03.

**Results**

Over the 12-week DBTP, incidence of ISR-AEs was 3.2%, 5.6%, and 4.5% in the placebo, erenumab 70 mg, and erenumab 140 mg groups, respectively (Table 1).

Over the EEP, which extended erenumab exposure to median 46 weeks (mean 47, range 0–159), incidence of ISR-AEs was 6.1% and 4.2% in the erenumab 70 mg and 140 mg groups, respectively (Table 2)

Over the EEP across both doses, most ISR-AEs were mild (Grade 1). Moderate ISR-AEs (Grade 2) were injection site erythema (four subjects, 0.2%), injection site pain (three subjects, 0.1%), and injection site reaction, injection site induration, and injection site urticaria (one subject each, <0.1%). There were no ISR-AEs of Grade >2, and no serious ISR-AEs. Across 2519 subject years of erenumab exposure, one subject (<0.1%) discontinued due to injection site pain, one due to injection site rash, and one due to injection site urticaria.

**Conclusion**

ISR-AEs occurred in a small proportion of subjects treated with either dose of erenumab, with little change over time. Most ISR-AEs were mild and did not require discontinuation.

**Table 1 (abstract P36).** ISR-AEs over the 12-week DBTP, n (%)

	Erenumab, mg		
	Placebo (N=1043)	70 (N=893)	140 (N=507)
Any ISR-AE	33 (3.2)	50 (5.6)	23 (4.5)
ISR-AEs with >0.5% frequency in any group			
Injection site pain	18 (1.7)	33 (3.7)	8 (1.6)
Injection site erythema	2 (0.2)	9 (1.0)	10 (2.0)
Injection site pruritus	3 (0.3)	4 (0.4)	4 (0.8)

**Table 2 (abstract P36).** ISR-AEs over the EEP

	Erenumab, mg	
	70 (N=2128)^a^	140 (N=1198)^a^
Subjects who experienced an ISR-AE, n (%)	130 (6.1)	50 (4.2)
Total time at risk summed across subjects, years	1738	649
Exposure-adjusted subject incidence rate per 100 subject-years	7.5	7.7

^a^Subjects who received >1 dose level were counted in both dose levels

### P37 Ipoglycemia a trigger factor for migraine chronification in migraineurs: diagnosis with glycemic load

#### Claudio Mostardini

##### Headache Center, Ospedale G.B. Grassi, ASL Roma 3

Chronification of migraine has many known factors and many others under study, like psychological, psychiatric, metabolic factors, metabolic poor management of therapy, etc.

Simple and cost-effective parameters, to identify target for new therapy have always been the target of research. Careful observation of headache diaries shows sometimes that some migraine attacks may be recurrent and methodically after 2-3h after meals, especially if carbohydrates rich, such as breakfast and lunch in the Mediterranean diet.

The fact that nutritional and metabolic factors can worse and chronify migraine is well known, but body weight and body mass index (BMI) are probably not the only elements to evaluate in these patients.

We therefore identify patients with chronic migraine diagnosis in which a diary with a methodically post-prandial attack, we performed a glycemic and insulin-like curve of with a load of 75g glucose and subsequent withdrawals at 0-60-120-180 minutes. We collected also data on headache/day frequency per month, BMI, anamnestic presence of polycystic ovary in the woman.

We currently recruited 28 subjects, 24 women, 4 men 32.6 years of age, BMIs between 34 and 18 with an average of 26.9, mean headache days per month of 18,2.

Comparing the glycemic curves of these patients it is noted that baseline blood glucose was for everyone within the norm parameters, but in 24 of 28 patients between 2 and 3 hours the blood glucose decreased below baseline and in 20 below normal values, (hypoglycaemia), no patient has exceeded 200mg /dl.

For insulinemic values, all patients showed normal baseline values that increase after 60 minutes reaching > 10 times of baseline, with gradual reduction over the next 3h.

Based on the data obtained, we have diagnosed metabolic syndrome in 6 patients, for the correlation between hyperinsulinaemia and high BMI, and insulin resistance related to polycystic ovary syndrome in 22 patients with hypoglicemya and PCOS history.

Both group of patients with metabolic syndrome and insulin resistance, were then subjected low charb diet. Preliminary data show that there is a rapid reduction in body weight and the reduction/ansence of post meal migraine attack with a mean reduction of more than 50% on headache days.

**References**

Barbanti P, Fofi L, Aurilia C, Egeo G, Caprio M. Ketogenic diet in migraine: rationale, findings and perspectives. Neurol Sci. 2017 May;38(Suppl 1):111-115

### P38 Peripheral Vagal Nerve Stimulation attenuates migraine aura: a case report

#### Grazia Sances^1^, Vito Bitetto^1^, Eric J Liebler^2^, Roberto De Icco^1^, Michele Viana^1^, Cristina Tassorelli^1,3^

##### ^1^Headache Science Center (HSC), C. Mondino National Institute of Neurology Foundation, Pavia, 27100, Italy; ^2^Electrocore LLC, Basking Ridge, NJ, 07920, USA; ^3^ Dept of Brain and Behavioural Sciences, University of Pavia, 27100, Italy

###### **Correspondence:** Grazia Sances (grazia.sances@mondino.it)

**Background**

Noninvasive neuromodulation techniques promise efficacy in the treatment of migraine [1]. Interestingly, Ayata et al. have recently reported that noninvasive direct vagus nerve stimulation (nVNS) significantly suppresses spreading depression susceptibility in the occipital cortex in rats [2]. Here we describe a case of a female patient whose aura was repeatedly and consistently shortened by peripheral vagal nerve stimulation using the gammaCore device.

**Case report**

The subject is a 38-year-old female patient with both migraine with (MA) and without aura (MwA), diagnosed according to the ICHD-3 beta criteria [3] The reported mean frequency of MwA days per month was 7-8, while the frequency of MA attacks was 4-5 *per year.* Usual aura consisted in monolateral negative scotoma with gradual onset, progressively evolving in homonymous hemianopia (either left or right) and followed by unilateral paresthesias with a progressive involvement of the arm and hemiface. The full aura duration was 1-2 hours, being followed almost immediately by severe unilateral migraine (either left or right) resistant to triptans. MRI and angio-MRI showed a paraphysiological vascular variation of the anterior communicating artery which devoid of clinical relevance. The patient was taking cinnarizine, 25 mg daily, for migraine prevention. She was also on combined oral contraception since 2013, without any worsening effect of hormonal treatment on either MA or MwA. Medical history showed only an asymptomatic iron deficient anemia.

The patient first used the gammaCore device during a clinical study for the acute treatment of migraine attacks. She was trained to deliver one 120” stimulation per side of the neck at the beginning of the attack, regardless of the location of the pain.

During the open label phase of the study, the patient had the possibility to treat multiple migraine attacks. During the 3 treated MA attacks, the patient recorded on the electronic diary a consistent effect of nVNS in terms of: reduction of the duration of the visual symptoms (from the usual 60-120 minutes to 1-2 minutes), the complete prevention of the somatosensory aura and the prevention and/or reduction of the painful phase.

**Conclusions**

Previous data from the literature clearly show that, in animal studies, nVNS modulates multiple pain pathways and decreases cortical spreading depression. VNS activates nucleus tractus solitarius, locus ceruleus and dorsal raphe nuclei, all of which can modulate CSD susceptibility [2]. To the best of our knowledge this is the first documented report on the effectiveness of nVNS using gammaCore on aura symptoms and, together with the experimental data cited above, it provides a rationale for assessing the potential effect of nVNS in the acute treatment of MA.

**Conflicts of interests**

CT advisory board fees from Allergan S.p.A. and electroCore, LLC

EL electroCore, LLC

**Consent to publish**

The patient signs a consent form and she gives consent for information about herself to be published.

**References**

1. Puledda F, Goadsby P.J. An Update on Non-Pharmacological Neuromodulation for the Acute and Preventive Treatment of Migraine. Headache. 2017; 57(4):685-691.

2. Chen SP et al. Vagus Nerve Stimulation Inhibits Cortical Spreading Depression. Pain. 2016; 157(4): 797–805.

3. Headache Classification Committee of the International Headache Society (IHS); The International Classification of Headache Disorders, 3^rd^ edition. Cephalalgia. 2013; 33(9) 629-808.

### P39 Cortical laminar necrosis in a 14-year old patient with status migrainosus

#### L. M. Messina, C. Gallo, V. Raieli, E. Correnti, F. Vanadia

Cortical laminar necrosis (CLN) is a permanent brain injury radiologically with gyriform distribution. CLN is caused by cerebral energy depletion and it is characterised by high-intensity cortical lesions on T1-weighted MRI images which follow the gyral anatomy of the cerebral cortex. In children, cortical laminar necrosis is usually seen in the subacute or chronic phase of brain damage in hypoxic-ischemic encephalopathy. Other possible etiologies are metabolic disorders, hypoglycemia, renal and hepatic dysfunction and immunosuppressive chemotherapy. CLN may also be seen in patients with encephalitis, but it's an uncommon finding in migraine and status migrainosus with or without aura.

We present the case of a 14-year old male patient, without known history of migraine or neurologic issues, who came to our attention for the manifestation of an acute and long persistent (>72h) symptomatology characterized by headache, confusional state, dysarthria, aphasia and visual disturbances. Clinical evaluation, laboratory tests and instrumental examinations were performed during the hospitalization. Submitted to a first emergency RMN, a localized edema in the supramarginal gyrus in left brain hemisfere has been identified as a possible vascular etiology. Supramargynal gyrus (Broadmann's area 40), a portion of the parietal lobe, is considered to be part of Wernicke's area and it is probably involved with language perception and processing. At a RMN control, it was possible to highlight a laminar necrosis of the cortical-pial zone in that area. This case appears to be interesting for the uncommon correlation between cortical laminar necrosis and status migrainosus with aura as well as for the site of the hypoperfusion revealed through RMN and for the atypical progression of the aura.

**Consent for publication:** The authors declare that written informed consent was obtained for publication.

**References**

- Adrià Arboix, Sebastià González-Peris, Elisenda Grivé, María-José Sánchez, Emili Comes. Cortical laminar necrosis related to migrainous cerebral infarction. World Journal of Clinical Cases 2013 November 16; 1(8): 256-259

- Migraine and ischaemic vascular events. Cephalalgia 2007; 27: 965-975

- Tietjen EG. Migraine and ischaemic heart disease and stroke: potential mechanisms and treatment implications. Cephalalgia 2007; 27: 981-987

- Bousser MG, Welch KM. Relation between migraine and stroke. Lancet Neurol 2005; 4: 533-542

- Komiyama M, Nakajima H, Nishikawa M, Yasui T. Serial MR observation of cortical laminar necrosis caused by brain infarction. Neuroradiology 1998; 40

- Headache Classfication Subcommittee of the International Headache Society. The International Classification of Headache Disorders: 2nd edition. Cephalalgia 2004; 24 Suppl 1: 9-160

- A Donaire, M Carreno, B Go’mez, P Fossas, N Bargallo’, R Agudo, M Falip, X Setoaı’n, T Boget, T Raspall, V Obach, J Rumia. Cortical laminar necrosis related to prolonged focal status epilepticus. J Neurol Neurosurg Psychiatry 2006;77:104–106.

- Alexander M. McKinney, Mehmet Teksam, Ross Felice, Sean O. Casey, Ronald Cranford, Charles

- L. Truwit, and Stephen Kieffe. Diffusion-Weighted Imaging in the Setting of Diffuse Cortical Laminar Necrosis and HypoxicIschemic Encephalopathy. AJNR Am J Neuroradiol 25:1659–1665, November/December 2004

- T. Niwa N. Aida A. Shishikura K. Fujita T. Inoue. Susceptibility-Weighted Imaging Findings of Cortical Laminar Necrosis in Pediatric Patients. AJNR 29 Oct 2008

- Sethi N K, Torgovnick J, Macaluso C, Arsura E. Cortical laminar necrosis following anoxic encephalopathy. Neurol India 2006;54:327

### P40 Visual snow in a patient with occipital stroke: do we have to rethink migrainous infarction pathophysiology?

#### Teresa Catarci (teresa.catarci@aslroma2.it)

##### Headache Centre, Azienda Sanitaria Locale Roma 1-2, Rome, Italy

**Background**

Visual snow (VS) has recently been described as a distinct event from persistent migraine aura and classification criteria have been proposed [1]. The latest classification of headache disorders of 2015 [2] describes migrainous infarction (MI) as a typical aura that persists and neuroimaging shows an ischaemic lesion in the contralateral cortex.

We describe the case of a patient who reported short-lasting daily symptoms of VN in his entire visual field, for about 40 years, until the day where he woke up with a continuous right sided visual snow phenomenon and was later diagnosed a left occipital stroke.

**Case report**

On January 2017 a 74 years-old male was referred to our headache outpatient clinic with the diagnosis of cerebral ischaemia and migraine aura. Nine months before he was admitted to the emergency department due to persistent visual symptoms started 8 hours before: at neurological examination he had a left visual field impairment, therefore he was admitted to the neurological ward with the diagnosis of possible protracted migraine aura. He was discharged 6 days later, with the diagnosis of cerebral ischaemia due to occlusion of right posterior inferior cerebral artery. Few weeks after the discharge, a paroxysmal atrial fibrillation was detected on 24-hours ECG and a treatment with novel anticoagulants started. One month later a visual field detected superior right homonymous quadrantopia.

When we first visited the patient, he referred that, prior to the stroke, he had been suffering over the last 40 years, of sudden impairment of vision, lasting for about 5 minutes, similar to a badly-tuned tv channel, with little spots moving in the entire visual field, with little if no disability. He also referred no significant headache ever. After the occurrence of the stroke the same symptoms became persistent and localised at his left visual field. Neurological examination disclosed, in addition to the known visual field impairment, bilateral hypoacusia with left tinnitus. Therefore, a diagnosis of persistent partial VN phenomenon secondary to occipital stroke was done and Magnetic Resonance Imaging (MRI) prescribed together with a visual field: no changes were later reported in the follow-up visit in both exams.

**Conclusions**

We report, for the first time, the case of a patient whose temporary daily VN phenomenon reversed to a persistent one, in the visual field affected by ischaemic occipital stroke, as typically happens in migraine stroke. This fact may open a new scenario in the pathophysiogenesis of MI that is believed to be due to either vasoconstriction or protracted oligoemia after an aura [3], while visual snow phenomenon has been associated to an increase of blood flow [4]. We hypothesise that, in our patient, the occipital lesion disrupted inhibitory circuits producing quadrantopic persistent VN. The same mechanism could be hypothesised for MI, where the aura could be the result rather then the actual cause of the stroke itself.

**Consent for publication:** Written informed consent was obtained

**References**

1. Shankin CJ, Maniyar FH, Digre KB and Goadsby P. ‘Visual snow’ – a disorder distinct from persistent migraine aura. Brain 2017:137; 1419-1428

2. Olesen J. Headache classification committee of the international headache society (IHS). The international classification of headache disorders, 3^rd^ edition (beta version). Cephalalgia 2013;33:629-808.

3. Gryglas A, Smigiel R. Migraine and stroke: What’s the link? What to do? Curr Neurol Neurosci Rep. 2017;17-22.

4. Schankin CJ, Manyiar FH, Sprenger T et al. Brain structural and neurometabolic correlates of visual snow disorder*. Neurology* 2015;84 (Supplement) P1.291

### P41 Treatment patterns and medication use among migraine patients in Finland

#### Timo Purmonen, Hanna Wahlman, Minna Korolainen

##### Novartis Finland Oy, Espoo, 02130, Finland

###### **Correspondence:** Timo Purmonen

**Background**

Migraine is a disabling neurological disease. While it is most common among working-age population, it presents significant indirect costs to society, especially when migraine attacks are frequent. The aim of this study was to assess the prevalence of migraine, and proportion of patients needing prophylactic treatment in different age-groups. In addition, we aimed to define the current primary care migraine treatment patterns in Finland.

**Materials and methods**

We conducted a retrospective register-based study based on prescription data from primary healthcare electronic medical records. The data set covers an overall population of 2,1M inhabitants. Patients were included in the study cohort if they had ≥ 1 prescription for an acute or prophylactic migraine medication during 1.1.2012-30.9.2016. Patients of all ages were included in the cohort. An open text search was combined to search based on ICD-10 and ATC-codes, in order to confirm the accuracy of migraine-related use.

**Results**

Altogether 61,077 migraine patients were identified. Among these patients 94% were receiving at least one acute treatment (n=57,186), and 20% at least one prophylactic treatment (n=12,082). The prevalence of migraine was 2,9% in the overall population, and 4,2%-5,3% among age-groups between 15-55 years. The most commonly prescribed treatments for acute events were triptans (56%) and NSAIDs (38%). Among the prescribed prophylactic treatments, the most common were beta-blockers (48%), tricyclic antidepressants (24%), angiotensin-receptor-blockers (14%), and anti-epileptics (7%). The most common combination of two acute treatments (NSAID and triptan) were prescribed to 19% of the patients (n=11,625). Prophylaxis was mainly based on monotherapy, but it was often combined with an acute treatment. The most common combination of acute and prophylactic treatment was triptans and beta-blockers. This combination was prescribed for 32% (n=3,832) of patients on prophylactic treatment.

**Conclusions**

Medical treatment of migraine in Finnish primary care is based on combinations of various substances. Migraine was most common among ages between 15-55 years. Among all patients, 20% received prophylactic treatment.

### P42 The impact of fremanezumab on headache-related disability in patients with chronic migraine using the Headache Impact Test

#### Paul K Winner^1^, Timothy Fitzgerald^2^, Sanjay K Gandhi^2^, Paul P Yeung^2^, Joshua M Cohen^2^, Yuju Ma^2^, Ernesto Aycardi^2^

##### ^1^Palm Beach Neurology, West Palm Beach, Florida, 33407, USA; ^2^Teva Pharmaceutical Industries, Frazer, Pennsylvania, 19355, USA

###### **Correspondence:** Paul K Winner

**Background**

Patients with chronic migraine (CM) experience substantially impaired daily functioning and reduced quality of life, with daily or near-daily headache attacks. In clinical trials, fremanezumab, a fully humanized monoclonal antibody that selectively targets calcitonin gene-related peptide, reduced the frequency, severity, and duration of headaches in patients with CM. The impact of migraine cannot be fully understood only by assessment of headache frequency; the validated 6-item Headache Impact Test (HIT-6) was used to assess the effect of fremanezumab versus placebo on headache-related disability.

**Methods**

In this multicenter, randomized, double-blind, placebo-controlled, Phase III study, eligible patients with CM were randomized 1:1:1 to receive subcutaneous injections of fremanezumab quarterly dosing (675 mg at baseline and placebo at Weeks 4 and 8), fremanezumab monthly dosing (675 mg at baseline and 225 mg at Weeks 4 and 8), or placebo at each time point over a 12-week treatment period. As a secondary endpoint, change in HIT-6 score was evaluated from baseline to 4 weeks after administration of the last dose of study drug. HIT-6 scores range from 36 to 78, with higher scores indicating a greater impact of headache on daily life. Efficacy analyses were performed in the full analysis set (FAS; all randomized patients who received at least one dose of study drug and had at least 10 days of post-baseline efficacy assessments on the primary endpoint) and repeated for the per-protocol analysis set (PPS). The data were analyzed using the analysis of covariance approach, with baseline HIT-6 score and years since onset of migraine used as covariates. *P*-values for treatment comparisons were based on the Wilcoxon rank-sum test.

**Results**

Treatment with both fremanezumab dose regimens yielded significant improvements in disability, as measured by the reductions in HIT-6 scores from baseline to 4 weeks after administration of the last study dose. In the FAS, the least-squares mean ± standard error changes from baseline with fremanezumab quarterly dosing (–6.4±0.45 points) and monthly dosing (–6.8±0.44 points) were greater than with placebo (–4.5±0.45 points); resulting in significant treatment differences (relative to placebo) in HIT-6 score change for fremanezumab-treated patients (quarterly: –1.9±0.49 points, *P*=0.0004; monthly: –2.4±0.49 points, *P*<0.0001). Similar treatment differences were observed in the PPS (quarterly: –2.1±0.51 points, *P*=0.0001; monthly: –2.3±0.51 points, *P*<0.0001).

**Conclusions**

In this Phase III study, fremanezumab treatment demonstrated a significant improvement in headache-related disability in patients with CM.

**Trial registration**

ClinicalTrials.gov NCT02621931

**Competing Interest**

Paul K Winner has been an investigator in clinical trials sponsored by Teva, Amgen, Genetech, Novartis, Allergan, AstraZeneca, Biogen Idec, and Ipsen. He has participated in advisory boards for Teva, Amgen, Avinar, Novartis, and Allergan, and has been on a speaker’s bureau for Allergan, Avinar and Teva.

All other authors are employees of Teva Pharmaceutical Industries.

### P43 Development of the Italian version of the “identify chronic migraine” (IT-ID-CM)

#### Simona Sacco^1^, Silvia Benemei^2^, Sabina Cevoli^3,^ Gianluca Coppola^4^, Pietro Cortelli^3^, Francesco De Cesaris^2^, Cristiano De Marco^5^, Cherubino Di Lorenzo^4^, Luana Evangelista^1^, Pierangelo Geppetti^2^, Andrea Negro^5^, Giulia Pierangeli^3^, Francesco Pierelli^4^, Luigi Alberto Pini^6^, Francesca Pistoia^1^, Antonio Russo^8^, Cristina Tassorelli^7^, Gioacchino Tedeschi^8^, Paolo Martelletti^5^

##### ^1^Department of Applied Clinical Sciences and Biotechnology, Section of Neurology, University of L’Aquila, L’Aquila, Italy; ^2^ Department of Health Sciences, Section of Clinical Pharmacology and Oncology, University of Florence, Florence, Italy; ^3^ Department of Biomedical and Neuromotor Sciences, University of Bologna, Bologna, Italy; ^4^ Department of Medico-Surgical Sciences and Biotechnologies, Sapienza University Polo Pontino, Latina, Italy; ^5^ Department of Clinical and Molecular Medicine, Regional Referral Headache Centre, Sant'Andrea Hospital, Sapienza University, Rome, Italy; ^6^ Headache and Drug Abuse Research Centre, Policlinico Hospital, University of Modena e Reggio Emilia, Modena, Italy; ^7^ Headache Science Center, C. Mondino National Neurological Institute, Pavia, Italy Dept. of Brain and Behavioral Sciences, University of Pavia, Italy; ^8^ Department of Medical, Surgical, Neurological, Metabolic and Aging Sciences, Second University of Naples, Naples, Italy

**Background**

Chronic migraine is an underdiagnosed and undertreated condition. Tools to improve chronic migraine detection by health care professionals may promote proper referral to dedicated care. Recently, the self-administered tool ‘Identify Chronic Migraine (ID-CM)’ was developed and validated to help clinicians to identify patients likely to suffer from CM. The aim of the present study was to develop the Italian version of the ID-CM (IT-ID-CM).

**Material and Methods**

One participant in the study translated the original version of the ID-CM into Italian. Then, a meeting was held to share the draft of the IT-ID-CM among study participants and discuss possible issues related to the translation and future validation. After the meeting, each study participant was asked to compare the draft of the IT-ID-CM with the original version and provide comments and suggestions. The suggestions were reviewed by two study participants, and a revised draft was developed. The draft was back-translated into English and compared with the original version to develop the final IT-ID-CM.

**Results**

We developed a fully comprehensible and accurate Italian translation of the ID-CM consistent with the original English text (Figure).

**Conclusions**

The IT-ID-CM is now available for evaluation in the clinical setting. The next steps foresee the validation of the tool in specialized and primary care settings, and the creation of an application for smartphones, tablets and desktop computers, which will help to promote the diffusion of the IT-ID-CM.

**Acknowledgements**

*This project was supported by an unconditional grant from Allergan Italy to the Fondazione Italiana per lo Studio delle Cefalee Onlus.*

**References**

Lipton RB, Serrano D, Buse DW, Pavlovic JM, Blumenfeld AM, Dodick DW, Aurora SK, Becker WJ, Diener H-S, Wang S-J, Vincent MB, Hindiyeh NA, Starling AJ, Gillard PJ, Varon SF, Reed ML (2016) Improving the detection of chronic migraine: Development and validation of Identify Chronic Migraine (ID-CM). Cephalalgia 36:203–215.

### P44 Importance of Anatomical Diagnostic Approach for Migraine and Tension type headache using Acupuncture and Meridian of Korean Hand Therapy

#### SoonWon Park^1^, Kyuhyun Park^2^, Taewoo Yoo^3^

##### ^1^Dept. of Neurology, Bongseng Memorial Hospital, Busan, Korea; ^2^Emeritus Prof. Pusan National University, Busan, Korea; Dept. of Neurology, Jungang Nara Hospital, Busan, Korea; ^3^Korean Hand Acupuncture Therapy Institute, Seoul, Korea

###### **Correspondence:** Kyuhyun Park (qhynbak@pusan.ac.kr)

**Background**

The current diagnosis of primary headache has not been sufficient for appropriate treatment. We used to make diagnosis based on the history taking and criteria of International Headache Classification. One of criteria is location such as unilateral or bilateral. Correct anatomical diagnosis might be a clue to the problem. There are no standardized methods to decide the location (side and sites) in practice. Traditional Acupuncture and Meridian System has been used for long in oriental medicine. It is well described, but it is complicated to use easily. It is difficult to resolve the underdiagnosis and undertreatment of headache. Therefore, we propose the Acupuncture and Meridian of Korean Hand Therapy (KHT) as a tool for diagnosis of migraine and tension type headache.

**Subjects and methods**

This procedure was performed during physical examination based on history taking including detailed palpation on the affected regions at Department of Neurology, Pusan National University Hospital from March, 2009 to February, 2012. The 200 primary headache patients who had no other neurological or systemic diseases were included. We checked pain sites or tender points on both sides of head using Acupuncture points and Meridian of Gallbladder (CM1-12) and Urinary Bladder (CI1-8) of KHT.

**Results**

We identified several tender points, which were connected to each other. The points were closely related with the Acupuncture and Meridian of KHT, which were different from depending on the type of headache. The pain sites and tender points of primary headache presented as Gallbladder and Urinary Bladder Meridian of KHT. The migraine belonged to Gallbladder and tension-type headache belonged to Urinary Bladder Meridian, respectively. Also mixed type headache belonged to various combined Gallbladder and Urinary Bladder Meridian. The pure migraine groups are divided into three, pure tension type headache groups are three and mixed form headache group are nine, respectively.

**Conclusion**

The pain location of primary headache patients is closely related with Acupuncture and Meridian System of KHT. Application of this method might improve the diagnostic accuracy of primary headache such as migraine and tension-type headache.

### P45 Use of nutraceutical Clevia, in Pure Menstrual Migraine

#### Claudio Mostardini

##### Headache Center, Ospedale G.B. Grassi, ASL Roma 3

The use of nutraceuticals in the prevention of migraine is increasingly spreading, both for a more holistic approach to pathology and for a patient’s specific request to use less “traditional drugs” and ultimately for their effectiveness, which it is proving to be higher than expectations.

In this sense, Clevia combines the analgesic activity of the three major furanodienes of Commirhora myrrha (MyrLiq), the activity of compensating metabolic mytocondrial deficits of riboflavin, the effect of coenzyme Q10 (CoQ10) that improves energy metabolism neuronal, and last vasoactive and scavenger activity of Gingko Biloba and its derivatives.We decided to test this nutraceutical medicine in one of the most difficult forms of migraine, that is pure menstrual migraine, consisting of migraine attacks closely related to the menstrual cycle.

We selected 7 women with age between 25 and 46 with migraine attacks exclusively during the menstrual phase, with the possibility of using estroprogestin therapy. Patients were given a headache diary that monitored the last three menstrual cycles.Patients had an average of 3.8 days headache per period with an average pain intensity evaluated with a numerical rating scale (NRS) of 8.5 and a use of about 6.2 pain killer / menstrual cycle. Patients were then treated with Clevia 1 cp for about 15 days a month starting 10 days before the menstrual cycle covering the whole cycle for 3 months. Therefore, the previously monitored parameters, headache days, mean pain intensity, number of killer pain per menstrual cycle were compared with those of patients without clevia.

The results have shown that although there was no significant reduction in the number of headache days per period, with a 3.8 to 3 day variation, a marked reduction in pain intensity was observed, which was more manageable with the usual pain killer and consequently even less use of the same, thus resulting in an average pain intensity of NRS of 8.8 to 5.5 with a reduction of Pain Killer from 7.8 to 4 per period.Finally, NRS evaluated the degree of satisfaction of patients with clevia therapy on various parameters (ease of use, side effects, clinical improvement, costs, etc.) compared to non-use with a mean satisfaction value of 7, 8 ie medium / high.This study highlights how the use of nutraceuticals such as Clevia can be useful in the management of migraine patients and in particular of once with pure menstrual migraine.

**References**

4. I-H-S Classificazione Internazionale delle Cefalee III beta Cephalalgia. 2013 Jul;33(9):629-808

5. Germano A, Occhipinti A, Barbero F, Maffei ME. A Pilot Study on Bioactive Constituents and Analgesic Effects of MyrLiq®, a Commiphora myrrha Extract with a High Furanodiene Content.Biomed Res Int. 2017;2017:3804356.

6. Thompson DF, Saluja HS. Prophylaxis of migraine headaches with riboflavin: A systematic review. J Clin Pharm Ther. 2017 Aug;42(4):394-403.

### P46 Maternal alexithymia and attachment style: which relationship with their children's headache features and psychological profile?

#### Samuela Tarantino^1^, Laura Papetti^1^, Cristiana De Ranieri^2^, Angela Rocco^2^, Valeria Valeriano^2^, Francesca Boldrini^2^, Barbara Battan^1^, Maria Francesca Paniccia^2^, Federico Vigevano^1^, Simonetta Gentile^2^, Massimiliano Valeriani^1,3^

##### ^1^Headache Center, Division of Neurology; ^2^Unit of Clinical Psychology, Ospedale Pediatrico Bambino Gesù, IRCCS, Piazza Sant’Onofrio 4, Rome, Italy; ^3^Center for Sensory-Motor Interaction, Aalborg University, Aalborg, Denmark

###### **Correspondence:** Samuela Tarantino

**Background.** A growing body of literature has showed a relationship between insecure “attachment style” and somatic symptoms. In a recent study, we found an association between ambivalent attachment style, migraine severity and psychological symptoms in children/adolescents. There is evidence that caregivers’ attachment styles and their way of management/expression of emotions can influence children’s psychological profile and pain expression. To date, data dealing with headache are scarce. We aimed to study the role of maternal alexithymia and attachment style on their children’s migraine severity (intensity and frequency) and psychological profile (anxiety, depression, somatization and attachment style).

**Materials and methods.** We enrolled 84 consecutive patients suffering from migraine without aura (female: 45, male: 39; age range 8-18 years; mean age 11.8 ± 2.4 years). Patients were divided into two groups according to frequency of the migraine episodes (high or low). According to headache attack intensity, patients were classified into two groups (mild and severe pain). Children’s psychological profile was assessed by SAFA Anxiety, Depression and Somatization scales. Attachment style was measured by the semi-projective test SAT for patients and ASQ questionnaire for mothers. Maternal alexithymia levels were evaluated by TAS-20.

**Results**. We found a significant higher score in maternal alexithymia levels in children classified as “ambivalent”, compared to those classified as “avoiding” (Total scale: p= 0.011). Alexithymia levels also correlated with children’s psychological profile. A positive correlation has been identified between mothers’ TAS-20 Total score and the children's SAFA-A Total Score (p=0.026). In particular, positive correlations were found between maternal alexithymia and children’s “separation anxiety” subscale (p=0.009) and “school anxiety” (p=0.015). Maternal “externally oriented thinking” subscale correlated with SAFA-A “school anxiety” subscale (p=0.050). Moreover, we found a correlation between TAS-20 Total score and SAFA-D “Feeling of guilt” subscale (p=0.014). Our data did not show any relationship between TAS-20 and ASQ questionnaires and children’s migraine intensity and frequency.

**Conclusions**. Maternal alexithymia and attachment style have no impact on children's migraine frequency and intensity. However, our results suggest that, although maternal alexithymic traits don’t play a causative role on children’s migraine severity, they show a relationship with patients’ attachment style and psychological symptoms, which in turn may impact on migraine severity.

### P47 Post accidental dural puncture headache: 3 years of obstetric experience using conservative treatment

#### Paolo Diamanti^1^, Laura Toscani^1^, Luigi Farina^1^, Maurizio Evangelista^2^

##### ^1^Cristo Re Hospital, U.O.C Anestesia e Rianimazione,Via delle Calasanziane 25, 00167 Rome, Italy; ^2^Università Cattolica del Sacro Cuore, Istituto di Anestesiologia, Rianimazione e Terapia del Dolore,Largo Francesco Vito, 1, 00168 Rome, Italy

###### **Correspondence:** Paolo Diamanti (diamantipaolo1@tin.it)

**Background**

Accidental dural puncture represents the most frequent complication of peridural analgesic techniques.

It can cause headache variable in terms of intensity and duration, sometimes requiring invasive treatments.

Accidental dural puncture headache treatment can be either conservative or interventional.

The first one only include prophylactic measures ( bed rest, fluid therapy, analgesia) while the second envisages the epidural blood patch execution.

In our birth centre (2000 deliveries for year) epidural technique is adopted for both labour analgesia and anaesthesia for cesarian section.

The aim of this observational study is to evaluate the effectiveness of preventive/conservative treatment of women affected by post dural puncture haedache in the obstetrics setting.

**Material and Methods**

We collected data from January 2014 to December 2016.

Primary end point was headache development, secondary the duration of in hospital stay.

The diagnosis of accidental dural puncture headache was carried out following the criteria of The International Classification of Headache Disorders 3rd edition.

All the patients who experienced dural puncture during the procedure received preventive/conservative treatment for headache development.

Treatment consisted in 48 hours bed rest, oral or ev fluid therapy (at least 2000ml/day), and 1g paracetamol PRN up to a maximum of 3g.

**Results**

On a total of 5898 delivery we practiced epidural analgesia/anaesthesia on 4128 women with an incidence of accidental dural puncture of 0.43 % (18 patients).

Anthropometric data of the included patients are reported in Table 1.

Among the treated patients only 2 (11%) women developed headache requiring analgesics administration.

These patients were discharged from the hospital in 4.18 days on average.

None experienced headache for longer than two days and in none of the selected cases epidural blood patch was required.

**Conclusion**

In our experience preventive/conservative treatment has proven to be effective on obstetrics patient with dural puncture. The complication prolonged patients hospital stay only in two cases and for three and four days only.

**Table 1 (abstract P47).** See text for description

Age	Weight	Height	BMI
33.7	73.6	164.8	27.09

**References**

1. Headache Classification Committee of the International Headache Society (IHS) The International Classification of Headache Disorders 3rd edition Cephalalgia 2013;33:629-808

2. Sprigge J. S., Harper S. J. Accidental dural puncture and post dural puncture headache in obstetric anaesthesia: presentation and management: A 23-year survey in a district general hospital Anaesthesia, 2008, 63, pages 36–43

### P48 Pediatric use of Tanacetum Parthenium, Griffonia Simplicifolia an Magnesium in the prophylaxis and symptomatic treatment of headache attacks

#### Anna Rita Bellomo^1^, Federica Di Ruscio^2^, Lorenzo Toni^3^ (tonilor@tin.it)

##### ^1^G.B.Grassi Hospital,Rome,Italy,00122; ^2^Medical and Surgery School Campus Bio-medico University, Rome Italy,00128; ^3^Department of Mental Health ASL RM/3, Rome,Italy,00125

###### **Correspondence:** Anna Rita Bellomo^1^ (annaritabellomo62@gmail.com); Federica Di Ruscio (federica.diruscio@gmail.com)

**Aim**: The purpose of this study is to evaluate the use and the self-perceived efficacy and tolerability of three nutraceutical components - Tanacetum parteninum, Griffonia simpliciofila and Magnesium - in children and adolescents with primary headaches without other comorbidities.

**Background:** Although pharmacological treatments are the first choice for migraine, adverse effects and contraindications limit the use of drugs in children. There is increasing evidence for the efficacy and tolerability of some complementary approaches as nutraceuticals in the management of headache disorders. Nutriceuticals are complementary therapies that include dietary supplements in form of vitamins and minerals. In the last few years, some nutraceutical preparations as magnesium, CoQ10, vitamin D, melatonin and others have been proposed as potential treatment for headache also in childhood. Triptans could be used more frequently as first or almost second choice for treating migraine attack in adolescents. [5]

**Methods**: 20 children (age 5 – 16 years) with ≥ three migraine attacks per month are treated with Aurastop. The treatment period was 3 months following a 4 week baseline period without prophylactic treatment. Patients were assessed before treatment and at the end of the 3-month-treatment-phase for days with migraine, migraine pain, burden of disease (HIT-6) and subjective evaluation of efficacy.

**Discussion**: Feverfew (Tanacetum parteninum) has potential function in reducing aura duration and complexity [1] through Parthenolide inhibition of nitroglycerin-induced neuronal activation in specific brain nuclei, as dorsal root ganglia. Griffonia simpliciofila has a role in reducing NMDA-receptors aberrant activity in trigeminal-vascular system, as well as in cortical spreading depression, (CSD) developing. The activity of its precursor (kynurenic acid) acting as an endogenous NMDA receptor antagonist [2]; Magnesium, is involved in numerous enzyme reactions and important for energy metabolism. Deficiencies in magnesium may lead to neuronal dysfunction and are found in individuals with headache attacks; it has been associated with CSD, neurotransmitter release, platelet aggregation and vasoconstriction all of which are important aspects of pathophysiology [3].

**Conclusion:** All these observations are aimed at testing the synergistic effect of AurastopR as symptomatic treatment of migraine aura and related symptoms in childhood and prophylaxis of headache attacks too, as a already been done in adults[4]. The preliminary results of the study, that are still ongoing, are encouraging, and Tanaceutum partenum, Magnesium and 5HTP with their joint action would seem to have important impact in reducing the pain intensity and the frequency of headache.

**References**

1) Tassorelli, C., Greco, R., Morazzoni, P., Riva, A., Sandrini, G. and Nappi, G. (2005) Parthenolide Is the Component of Tanacetum parthenium That Inhibits Nitroglycerin-Induced Fos Activation: Studies in an Animal Model of Migraine. Cephalalgia: An International Journal of Headache, 25, 612-621. 10.1111/j.1468-2982.2005.00915.x

2) Chauvel, V., Vamos, E., Pardutz, A., Vecsei, L., Schoenen, J. and Multon, S. (2012) Effect of Systemic Kynurenine on Cortical Spreading Depression and Its Modulationby Sex Hormones in Rat. Experimental Neurology, 236, 207-214. 10.1016/j.expneurol.2012.05.002

3) Sun-Edelstein C., Mauskopm A. (2011) Alternative Headache Treatments: Nutraceuticals, Behavioral in Headache Current, American Headache Society, Willey Blackwell and Physical Treatments

4) Zavarise P., Dalla Volta G. (2017) A Combination of Tanacetum parthenium,Griffonia simplicifolia and Magnesium(Aurastop®) as Symptomatic AcuteTreatment for Migraine Aura:A Retrospective Cohort Study. In Open Access Library Journal, Volume 4, e3660, ISSN Online: 2333-9721, ISSN Print: 2333-9705

5) Toldo I., Rattin M., Perissinotto E., Survey on treatments for primary headaches in 13 specialized juvenile Headache Centers: The first multicenter Italian study, European Journal of Paediatric Neurology

### P49 The impact of fremanezumab on migraine-specific health-related quality of life and overall health status in chronic migraine

#### Richard B Lipton^1^, Sanjay K Gandhi^2^, Timothy Fitzgerald^2^, Paul P Yeung^2^, Joshua M Cohen^2^, Yuju Ma^2^, Ernesto Aycardi^2^

##### ^1^Albert Einstein College of Medicine, Bronx, New York, 10461, USA; ^2^Teva Pharmaceutical Industries, Frazer, Pennsylvania, 19355, USA

###### **Correspondence:** Richard B Lipton

**Background**

Chronic migraine (CM) is characterized by frequent attacks, which adversely affect health-related quality of life (HRQoL). In clinical trials, fremanezumab, a fully humanized monoclonal antibody that selectively targets calcitonin gene-related peptide, reduced the frequency, severity, and duration of headaches in patients with CM. This study measured HRQoL using the Migraine-Specific Quality of Life (MSQoL) questionnaire and health status using the EuroQol 5-dimension 5 response level (EQ-5D-5L) questionnaire in patients treated with fremanezumab versus placebo.

**Methods**

In this multicenter, randomized, double-blind, placebo-controlled study, patients with CM were randomized 1:1:1 to receive subcutaneous injections of fremanezumab quarterly dosing (675 mg at baseline and placebo at Weeks 4 and 8), fremanezumab monthly dosing (675 mg at baseline and 225 mg at Weeks 4 and 8), or placebo at each time point over a 12-week treatment period. The MSQoL questionnaire (version 2.1) assessed the role function-restrictive (RR), role function-preventive (RP), and emotional function (EF) domains (range 0–100; higher scores indicate better HRQoL). The EQ-5D-5L questionnaire allowed patients to report their general health status on a visual analog scale (VAS, range 0–100; higher scores indicate better health). MSQoL domains were analyzed using a mixed-effects repeated-measures model (with years since onset of migraine and baseline MSQoL domain score as covariates). EQ-5D-5L analyses were conducted using an analysis of covariance approach (with years since onset of migraine and baseline EQ-5D-5L score as covariates).

**Results**

The study included 375 patients in each fremanezumab treatment group and 371 patients in the placebo group. Compared with placebo, fremanezumab treatment resulted in significant improvements in MSQoL scores from baseline to Week 12. For the RR domain of MSQoL, there were significant treatment differences with each fremanezumab group relative to placebo (quarterly: 5.6±1.4, *P*<0.0001; monthly: 6.3±1.4, *P*<0.0001). The RP and EF domains also showed significant treatment differences (*P*<0.05). Significant improvements with fremanezumab were observed as early as 4 weeks after the first dose and were sustained at all pre-defined assessments. As measured by the EQ-5D-5L VAS, fremanezumab-treated patients reported larger improvements in overall health status than those given placebo (4.6±1.1 [quarterly] and 4.8±1.1 [monthly] versus 2.2±1.1 [placebo]), resulting in significant treatment differences (quarterly: 2.4±1.2, *P*=0.0402; monthly: 2.6±1.2, *P*=0.0291).

**Conclusions**

These results indicate that fremanezumab improves migraine-specific QoL and overall health status of patients with CM, highlighting the positive impact of fremanezumab on CM patients’ ability to engage in and perform work and daily activities.

**Trial registration**

ClinicalTrials.gov NCT02621931

**Competing Interest**

**Richard B Lipton** is a consultant to Teva Pharmaceutical Industries.

**All other authors** are employees of Teva Pharmaceutical Industries.

### P50 Clinical presentation and diagnostic evaluation of idiopathic intracranial hypertension in children and adolescents

#### Barbara Battan, Laura Papetti, Irene Salfa, Federico Vigevano, Massimiliano Valeriani

##### ^1^Headache Center, Child Neurology Unit, Bambino Gesu’ Children’s Hospital, Rome, Italy

###### **Correspondence:** Barbara Battan (barbara.battan@opbg.net)

**Background**

Idiopathic intracranial hypertension (IIH) or pseudotumor cerebri is a syndrome characterized by signs and symptoms of increased intracranial pressure in the absence of a secondary cause. The aim of the study is to report the IIH clinical presentation in children and adolescents presenting to our hospital during a 10-year period.

**Materials and methods**

Retrospective study, between January 2007 and January 2017, of IIH patients, younger than 15 years, was conducted. Modified Dandy criteria were used for IIH diagnosis. The patients were analysed according to age (≤10 and 11-15 years).

**Results**

Thirty-four patients, ranging from 3.8 to 15 years, were included. Thirteen patients were younger than 11 years (38.2%), while twenty-one patients were 11-15 years old (61.7%). Twenty-nine patients (85.2%) were obese (weight centile ≥ 90%). Mean cerebrospinal fluid opening pressure was 422 mm H2O (260-890 mmH2O). The most common presenting symptoms were headache (94.1%), vomiting (29.4%), dizziness (11.76%), blurred vision or diplopia (67.6%). Sixth nerve palsy occurred in 11 children (32.3%). In general, headache did not respond to pain medication. All our patients showed papilledema. Diagnostic evaluation included neuroimaging studies and ultrasound-based optic nerve sheath diameter (ONSD) measurement. In 6 patients (15%), MRI or CT showed signs of empty sella syndrome, while in 9 patients (26.4%) ultrasound ONSD measurement showed optic nerve sheath distension. There were no significant differences between the age groups in both clinical presentation and instrumental findings. Treatment included weight loss and acetazolamide (maximum 5mg/kg/die) in 28 patients (82.3%). Furosemide was added to acetazolamide in 2 patients (5.8%) and in 2 other patients was necessary added Delatcortene (5.8%). All patients fully recovered and none of them complained visual loss in the follow-up.

**Conclusion**

IIH should be considered in children with new-onset headache. Clinical headache presentation can be variable, although vomiting and visual symptoms are frequently associated. To exclude a secondary cause, neuroimaging should be performed. ONSD measurement may be useful as an additional tool to identify patients with IIH. Early diagnosis and treatment for IIH can prevent potential visual loss that remains the major morbidity. Acetazolamide and weight loss remain the most effective treatments in children.

### P51 Onabotulinumtoxin A in a patient with ileointestinal bypass and chronic migraine: a case report

#### Ottavio Di Marco, Stefania Di Mauro, Fernando Ferrauti

##### ASL Frosinone

**Background**

Migraine is a common, chronic, incapaciting disorder, characterized by attacks of severe headache. Episodic migraine can progress to chronic migraine, which is defined as headache on ≥15 days/month for ≥3 months of which ≥8 days [1]. Onabotulinumtoxin A was approved in Italy in 2013 for symptom relief in patients with chronic migraine who have failed, or do not tolerate, oral prophylactic treatments [2].

**Case Report**

We describe the clinical case of a 36-year-old female patient who refers to the onset of cefalalgic pain at puberty, described with compression-type pain, diffused, with intolerance to light, hight intensity that prevented her from studying and lasted about 1-3 days. In the following years there was a further worsening of the frequency and intensity of the crisis. During the same period she began to take on weight, until serious obesity. She therefore carried out many hospital admissions, with the assumption of various anti-inflammatory therapies, until morphine. At 23 years she has been submitted to intestinal bypass surgery. Approximately 4-5 years ago, there were a re-onset of migraine, which gradually increased in intensity, not including anti-inflammatory therapies, with many accesses to Emergency Unit and use of opioid. Becouse of the ileointestinal bypass, shehad poor absorption with no repetition to prophylactic medical therapy. In September 2016 she was included in botulinum toxin treatment, meeting the criteria for chronic treatment-resistant migraine. Already after the second application, there was a regression of symptoms, a reduction of intensity and frequency of attacks and recovery of daily life activities.

**Conclusion**

There is good clinical evidence that treatment with onabotulinumtoxinA leads to a reduction of monthly headache days and improves quality of life [3], also in this case report in which other treatment were no possible and not efficacy due to ileointestinal bypass, with secondary malabsorption.

**Consent for publication:** The authors declare that written informed consent was obtained for publication.

**References**

1. Headache Classification Committee of the International Headache Society (IHS) (2013) International classification of headache disorders, 3rd edition (beta version). Cephalalgia 33:629–808

2. Russo A, Silvestro M, Tessitore A, Tedeschi G. The “Ram’s Horns Sign”: A Case Report of an Unusual Side Effect of OnabotulinumtoxinA in a Chronic Migraine Patient. Headache. 2016;56(10):1656-1658

3. Claus M Escher, Lejla Paracka, Dirk Dressler, and Katja Kollewe. Botulinum toxin in the management of chronic migraine: clinical evidence and experience. Ther Adv Neurol Disord. 2017 Feb; 10(2): 127–135.

### P52 The impact of headache free days on headache-related disability and productivity among people with migraine with ≥4 headache days in the past month

#### Lulu Lee^1^, Jvawnna Bell^2^, Timothy Fitzgerald^2^, Joshua M. Cohen^2^

##### ^1^Kantar Health, 1810 Gateway Drive, Suite 120, San Mateo, CA, 94404, USA; ^2^Teva Pharmaceutical Industries, Frazer, Pennsylvania, 19355, USA

###### **Correspondence:** Lulu Lee

**Objectives:** Determine the relationship between headache-related quality of life measures and headache free days (HFDs) among patients with ≥4 headache days in the past month.

**Methods:** The 2016 US National Health and Wellness Survey (NHWS; N=97,503) is a self-administered, sample of adults (≥18 years). Patients with a migraine diagnosis and reported experiencing ≥4 headache days a month were considered at risk for disease progression. Primary independent variable was the number of HFDs as both a continuous (30-number of HFDs in the past 30 days) and categorical (0-10;11-20;21-26 HFDs) measure. HFDs was used as a predictor in separate generalized linear models (GLMs).

Outcomes included patient reported number of days absent from work and days of household activities missed due to migraine, estimated annual indirect costs due to work productivity loss (assessed via Work Productivity and Activity Impairment Questionnaire). Headache-related disability was measured via the Headache Impact Test (HIT-6).

**Results:** Using HFDs as a continuous variable in the multivariable regression, each HFD was found to be associated with a 0.15 (regression coefficient) point reduction in HIT-6 scores. As a categorical variable, each 10 day increase in HFDs was associated with significantly lower HIT-6 total scores (adjusted means=66.59 [0-10 HFDs], 65.66 [11-20], 63.91 [21-30], all p<0.02).

Each HFD associated with 0.95 (Rate Ratio [RR]) times days of work missed due to migraines and 0.95 (RR) times days of household activities missed due to migraines. Thus, each HFD reduces both number of work days missed and number of days of household activities missed by 5%.

Increasing the number of HFDs from 0-10 to 21-26 (adjusted means=4.44 vs. 1.46, p=0.002) and from 11-20 to 21-26 (3.36 vs 1.46, p=0.009) categories was associated with significantly fewer work days missed due to migraine. Similarly, increasing the number of HFDs from 0-10 to 11-20 (adjusted means = 22.99 vs. 9.72, p<0.001) and from 0-10 to 21-26 (22.99 vs. 7.34, p=0.001) categories was associated with significantly fewer days of household activity missed due to migraine.

Increasing the number of HFDs from 0-10 to 21-26 per month was associated with significantly lower indirect costs.

**Conclusions:** Increasing the number of HFDs is associated with a decrease in headache-related disability among those with migraine who are at risk for disease progression. Migraine places a substantial indirect cost burden on this patient population and increasing total HFDs may help to reduce this burden.

### P53 Early Onset of Action with Fremanezumab Versus Placebo for the Preventive Treatment of Chronic Migraine

#### Paul Yeung^1^, Ernesto Aycardi^1^, Marcelo Bigal^1^, Tricia Blankenbiller^1^, Melissa Grozinski-Wolff^1^, Ronghua Yang^1^, Yuju Ma^1^, Jan Brandes^2^

##### ^1^Teva Pharmaceutical Industries, Frazer, Pennsylvania, 19355, USA; ^2^Nashville Neuroscience Group, Nashville, TN 37203, USA

###### **Correspondence:** Paul Yeung

**Background:** Migraine prevention is intended to reduce the frequency, severity, and disability associated with migraine attacks, and faster onset of action could increase benefit to patients with migraine. Fremanezumab is a fully humanized monoclonal antibody targeting the calcitonin gene-related peptide (CGRP) ligand, a preventive treatment designed to specifically target a pathophysiologic mechanism of migraine. This analysis assesses the early onset of action of fremanezumab in the prevention of chronic migraine.

**Methods:** A 16-week, multicenter, randomized, double-blind, placebo-controlled, parallel-group study comparing the efficacy, safety, and tolerability of 2 subcutaneous dose regimens of fremanezumab and placebo (PBO) in adults with Chronic Migraine (CM). Patients maintained a 28-day daily diary during baseline period, and throughout treatment period. Patients were assigned randomly to 1:1:1 ratio to 1 of 3 treatment groups: (1)monthly dosing: 675 mg of fremanuzemab followed by 225 mg of fremanezumab at months 2 and 3, (2)quarterly dosing: 675 mg of fremanuzemab at month 1, followed by placebo injections at months 2 and 3, and (3)monthly administration of matching placebo. The secondary endpoint, the mean change from baseline (28-day run-in period) to the 12-week randomization period in the monthly average number of migraine days, and results at Weeks 1, 2, 3 and 4 were also assessed using a mixed-effect model for repeated measures.

**Results:** Statistically significant reduction in the number of monthly headache days of at least moderate severity was experienced during the 12-week period after 1st dose for both fremanezumab dosing regimens [monthly (-4.6 days);quarterly (-4.3 days); p<0.0001] vs. placebo (-2.5 days), and during the 4-week period after 1st dose, for both dosing regimens (p<0.0001). Fremanezumab resulted in significant reduction in the weekly number of headache days of at least moderate severity:

- At Week 1, (-1.1 days; p<0.0001) versus placebo (-0.5 days)
- At Week 2, (-1.2 days; p<0.0001) versus placebo (-0.5 days)
- At Week 3, (-1.2 days; p<0.0001) versus placebo (-0.6 days)
- At Week 4, (-1.1 days; p=0.0006) versus placebo (-0.7 days)

Posthoc analysis indicated that more patients reported no headache of at least moderate severity with fremanezumab (69%; p=0.0036) versus placebo (61%) by the next day following first injection.

**Conclusion:** Onset of action with fremanezumab occurred rapidly for preventive treatment of migraine. Significant improvement was maintained for both monthly and quarterly subcutaneous injections.

### P54 Burden of illness among treated migraine patients with ≥4 headache days in the past month

#### Jvawnna Bell^1^, Lulu Lee ^2^,Timothy Fitzgerald^1^, Joshua M. Cohen^1^

##### ^1^Teva Pharmaceutical Industries, Frazer, Pennsylvania, 19355, USA; ^2^ Kantar Health, 1810 Gateway Drive, Suite 120, San Mateo, CA, USA 94404

###### **Correspondence:** Jvawnna Bell

**Objectives**: To determine the burden of illness among patients treated for migraine with ≥4 headache days in the past month.

**Methods**: The data source was the 2016 US National Health and Wellness Survey (NHWS; N=97,503), a self-administered, nationally representative sample of adults (≥18 years). Respondents were included in this analysis if they self-reported a diagnosis of migraine, experienced ≥ 4 headache days in the past 30 days, and were currently using a prescription treatment for migraine. Using propensity score matching to reduce bias, respondents meeting the above criteria were matched with people without migraine on age, gender, comorbidities (Charlson Comorbidity Index), annual household income, education, insurance status, body mass index (BMI), and smoking status. Outcomes included mental health comorbidities, work productivity and activity impairment as measured by the Work Productivity and Activity Impairment Questionnaire (WPAI), health utilization in the past 6 months (i.e., healthcare provider (HCP) visits, emergency room visits, and hospitalizations), and estimated annual indirect and direct costs. Post-match, groups were compared using One-Way ANOVAs and chi-square tests on outcomes.

**Results**: There were 197 respondents in each cohort. A statistically significantly greater proportion of treated migraine patients reported being diagnosed with depression than non-migraine controls (58.4% vs. 27.9%, p<0.001). A greater portion of treated patients also reported being on long-term disability compared to non-migraine controls (13.7% vs. 5.6%,p<0.003). Treated migraine patients reported greater losses in work productivity and increases in activity impairment. Compared to non-migraine controls, treated patients experienced greater absenteeism (11.8% vs. 6.3%, p=0.03), presenteeism (36.0% vs. 17.5%, p<0.001), overall work impairment (40.9% vs. 20.9%, p<0.001), and activity impairment (45.4% vs. 25.4%, p<0.001). These patients also reported more HCP visits (7.55 vs. 4.43, p<0.001) and ER visits (0.48 vs. 0.25, p=0.030) compared to non-migraine controls in the previous 6 months. Greater work productivity loss among treated migraine patients resulted in higher estimated annual indirect costs ($14,770.57 vs. $5,764.93, p<0.001) compared to non-migraine patient controls. Treated migraine patients utilized more healthcare services than non-migraine patients ($24,499.90 vs. $15,318.91, p=0.013).

**Conclusions**: The overall burden associated with migraine is substantial despite the availability of treatment options. Migraine patients in this survey reported a higher percentage of depression, long-term disability, work productivity loss, absenteeism, presenteeism, activity impairment, and use more health care services compared to people without migraine. As a result, patients treated for migraine incurred substantially greater direct and indirect costs compared to non-migraine controls.

### P55 Efficacy and Safety of Motilitone in Migraine Patients with Gastrointestinal Symptoms

#### Dong Wook Kim^1^ and Kwang Ki Kim^2^

##### ^1^Department of Neurology, Konkuk University School of medicine, Seoul, Korea; ^2^Department of Neurology, Dongguk University Ilsan Hospital, Ilsan, Korea

**Background**: Nausea and vomiting are a frequent accompaniment of migraine and prokinetic medications are frequently used in its management. Most prokinetic medications that are used in migraine are dopamine antagonists and therefore have the potential to cause drug-induced extrapyramidal symptoms. We evaluate the efficacy and safety of motilitone, a recently developed prokinetic drug, in migraine patients with gastrointestinal symptoms.

**Materials and Methods**: From two outpatient neurological clinics, we prospectively included 100 migraine patients with nausea and vomiting and treated them with medications including motilitone. For the evaluation of safety, we used drug-induced extrapyramidal symptoms (DIEPS) questionnaire, and the clinical improvement was assessed with clinical global clinical impression of change (C-GIC) and patients’ global clinical impression of change (P-GIC).

**Results**: Among the 100 patients, only two patients described the presence of mild extrapyramidal symptoms (one with difficulty in standing up from chair and the other with difficulty in find movements), but it was not certain that these extrapyramidal symptoms were associated with motiltione treatment and both patients refused to discontinue motiltione with the clinical improvement. The mean values of C-GIC and P-GIC were 3.26±0.8 and 3.19±1.1 respectively, which indicate mild improvement with the treatment.

**Conclusions**: Our study shows that medication including motiltione for migraine patients with gastrointestinal symptoms is effective and there is low risk of clinically significant extrapyramidal symptoms. Further studies are necessary to accurately evaluate the risk of extrapyramidal symptoms with motilitone treatment in patients with migraine.

### P56 Topiramate is more effective than acetazolamide at lowering intracranial pressure in healthy rodents

#### Hannah Botfield^1,2†,^ William J Scotton^1,2,3†^, Maria Uldall^4^, Connar Westgate^1,2^, James Mitchell^1,2,3^, Rigmor Jensen^4^, Alex Sinclair^1,2,3^

##### ^1^Institute of Metabolism and Systems Research, University of Birmingham, Edgbaston, B15 2TT, UK; Centre for Endocrinology, Diabetes and Metabolism, Birmingham Health Partners, B15 2TH, UK; ^3^Department of Neurology, University Hospitals Birmingham NHS Foundation Trust, B15 2TH, UK; ^4^Danish Headache Center, Clinic of Neurology, Rigshospitalet-Glostrup, University of Copenhagen, Nordre Ringvej 69, 2600 Glostrup, Denmark

###### **Correspondence:** Alex Sinclair (a.b.sinclair@bham.ac.uk)

^†^Joint first author

**Background**

Management of Idiopathic Intracranial Hypertension (IIH) aims to reduce intracranial pressure (ICP). Acetazolamide is the most commonly used drug, with class 1 evidence demonstrating modest improvement in patients with mild visual loss. Other drugs used include topiramate, furosemide, amiloride and octreotide, despite there being little mechanistic or clinical evidence to support their use. The aim of this study was to ascertain which of these drugs has the greatest effect on lowering ICP *in vivo*.

**Materials and methods**

Using a validated epidural ICP recording method, we measured changes in ICP in conscious female rodents after subcutaneous administration of clinical and high doses of drug over 2 hours (the time to peak serum concentration). Drugs evaluated, with clinical and high doses, were: acetazolamide (1g and 4g), topiramate (50mg and 200mg), furosemide (40mg and 240mg), amiloride (5mg and 20mg) and octreotide (350μg and 2mg). In addition, we measured ICP for 12 hours after oral administration of equivalent high doses of acetazolamide and topiramate. Dose conversion between rodents and humans followed the US Food and Drug Administration (FDA) guidance.

**Results**

At clinical doses, subcutaneous administration of topiramate lowered ICP by 32% (*p*=0.0009). There was no significant reduction in ICP noted with acetazolamide (-19%), amiloride (-11%), furosemide (-1%) or octreotide (-1%). At high doses, subcutaneous administration of topiramate lowered ICP by 21% (*p*=0.015) whilst there was no significant reduction in ICP noted with high subcutaneous doses of acetazolamide (-20%), furosemide (-13%), amiloride (-27%) and octreotide (-18%). Oral administration of equivalent high doses of topiramate lowered ICP by 22% (*p*=0.018), compared to only a 5% reduction with acetazolamide (*p*=>0.999).

**Conclusions**

Our in-vivo studies have demonstrated that, at both clinical and high subcutaneous doses, as well as high oral doses, administration of topiramate significantly lowers ICP. Other drugs tested, including the acetazolamide the current first line oral therapy in IIH, did not significantly reduce ICP. In the clinical setting topiramate may have additional advantages in IIH due to migraine prevention and weight loss effects, though the potential for negative impact on mood and cognition must also be considered. Future clinical trials evaluating efficacy and side effects of topiramate in IIH would be of interest.

### P57 National awareness campaign to prevent medication-overuse headache in Denmark

#### Louise Ninett Carlsen^1^, Maria Lurenda Westergaard^1^, Mette Bisgaard^1^, Julie Brogaard Schytz^2^, Rigmor Højland Jensen^1^

##### ^1^Danish Headache Center, Neurology Department, Rigshospitalet, Ndr. Ringvej 69, 2600 Glostrup, Copenhagen, Denmark; ^2^Association of Danish Pharmacies, Copenhagen, Denmark

###### **Correspondence:** Rigmor Højland Jensen (rigmor.jensen@regionh.dk, +45 38 63 30 59)

**Background:** Medication-overuse headache (MOH) is prevalent but in principle preventable. The objective is to describe the Danish national awareness campaign for MOH.

**Methods:** The Danish Headache Center, the Association of Danish Pharmacies, and headache patient organizations implemented a four-month MOH awareness campaign in 2016. Target groups were the general public, general practitioners, and pharmacists. Key-messages were: overuse of pain-medication can worsen headaches; pain-medication should be used rationally; and MOH is treatable. A range of communication technologies was used. A survey on the public’s awareness of MOH was conducted.

**Results:** The Danish adult population is 4.2 million. Online videos were viewed 297,000 times in three weeks. All 400 pharmacies received campaign materials. Over 28,000 leaflets were distributed. Two radio interviews were conducted. A television broadcast about headache reached an audience of 520,000. Forty articles were published in print media. Information was accessible at 32 reputable websites and 5 online news-agencies. Three scientific papers were published. Information was available at an annual conference of general practitioners including a headache lecture. The survey showed an increase in percentage of the public who knew about MOH (from 31% to 38%).

**Conclusion**: A concerted campaign to prevent MOH can be implemented through involvement of key stakeholders.

### P58 Alcohol as a risk factor for migraine attacks: an exploration

#### Pablo Prieto^1^, Gabriel Boucher^1^, Stephen Donoghue^1^, Alec Mian^1^, Noah Rosen^2^

##### ^1^Curelator Inc., Cambridge; ^2^Northwell Health, New York, United States

###### **Correspondence:** Pablo Prieto

**Background**

Alcohol has long been suspected as a migraine trigger commonly resulting in avoidance and possible impact on quality of life, but numerous studies have been inconclusive [1]. To explore this question we statistically compare intake of alcohol and occurrence of migraine.

**Methods**

Individuals with migraine registered to use Curelator Headache (1) through a physician ‘coupon referral’ program, (2) via the Curelator website or (3) the iOS App Store and answered questions about personal suspected triggers, including alcohol, and their importance (0=none; 10=maximal). Curelator Headache was used daily for at least 90 days, entering details about headaches and tracking factors that may affect migraine occurrence. Alcohol consumption was recorded daily as ‘YES/NO’ and if YES by type and units. After 90 days all factors were analyzed and for each individual the association of alcohol intake with attacks was determined [2].

**Results**

Of 531 individuals with migraine (Table 1), alcohol was suspected as a risk factor by 304 (57%).

Consumption of alcohol was significantly different between those who did and those who did not suspect it. A quarter of users never consumed alcohol and 32% of those who did had inadequate data for analysis (*e.g.* avoidance of alcohol or regular consumption so insufficient variability). In alcohol consumers, comparisons between those who *did not vs* those who *did* suspect alcohol as a trigger showed no association with migraine in the majority in both groups; alcohol as a potential trigger in 6% vs 8%; and as a potential protector in a 4% vs 7%.

No association was found between degree of suspicion of alcohol and the percentage of individuals with a confirmed association (Table 2).

Previous day analysis (Table 3) found association in different proportions.

**Conclusions**

Despite the belief that alcohol is a common risk factor for migraine, in the majority of users no association was found. Alcohol intake was less frequent in people not suspecting alcohol, possibly because abstainers would also not suspect alcohol as risk factor. Irrespective of the degree to which a user suspected alcohol as a trigger, more than 80% of users showed no association between alcohol and migraine. Same day intake was as often found to be associated with risk reduction (potential protector) as with risk increase (potential trigger) (7% each), but delayed association was mostly found as a potential trigger (17%). Our data do not support the hypothesis that alcohol is a common trigger for migraine.

**Table 1 (abstract P58).** User characteristics and risk factor associations in those suspecting alcohol as a risk factor, those who did not and all users

	Total	Did not consume alcohol	Consumed Alcohol	Adequate data for analysis	Potential trigger	Potential protector	No Association
Suspected	304 (57.3%)	23 (8%)	281 (92%)	198 (72%)	16 (8%)	14 (7%)	168 (75%)
Not suspected	227 (42.7%)	110 (48%)	117 (52%)	74 (64%)	4 (5.5%)	4 (5.5%)	66 (89%)
Totals	531	133 (25%)	398 (75%)	272 (68.3%)	20 (7%)	18 (7%)	234 (86%)

**Table 2 (abstract P58).** Association analysis by degree of suspicion

	Total	Potential trigger	Potential protector	No Association
Mildly suspected	75	4 (8%)	11 (21%)	35 (71%)
Moderately suspected	99	8 (11%)	10 (14%)	76 (75%)
Highly suspected	154	5 (5%)	16 (18%)	57 (77%)

**Table 3 (abstract P58).** Association by timing of alcohol intake

	Lagged analysis (previous day)			Same-day analysis		
	Potential trigger	Potential protector	No Association	Potential trigger	Potential protector	No Association
Suspected	34 (16.1%)	1 (0.5%)	176 (83.4%)	16 (8%)	14 (7%)	168 (75%)
Not suspected	15 (20.6%)	2 (2.7%)	56 (76.7%)	4 (6%)	4 (4%)	66 (89%)
Totals	49 (17%)	3 (1%)	232 (82%)	20 (7%)	18 (7%)	234 (86%)

**References**

[1] Panconesi A. Alcohol and migraine: trigger factor, consumption, mechanisms. A review. J Headache Pain (2008) 9:19–27

[2] Peris F, Donoghue S, Torres F, Mian A, Wöber C. Towards improved migraine management: Determining potential trigger factors in individual patients. Cephalalgia. 2017 Apr;37(5):452-463

### P59 Medication use and overuse patterns in a cohort of US and UK individuals with migraine using a digital platform

#### Pablo Prieto^1^, Gabriel Boucher^1^, Stephen Donoghue^1^, Stephen D. Silberstein^2^

##### ^1^Curelator Inc., Cambridge, United States; ^2^Jefferson Headache Center, Thomas Jefferson University, Philadelphia, United States

###### **Correspondence:** Pablo Prieto

**Background**

Overuse of acute medications may worsen migraine and lead to medication overuse headache (MOH) [1]. Here we describe medication use and identification of overuse (MO) in users of a digital platform for migraine (Curelator Headache^TM^). The objective is to compare medication use and possible overuse patterns in individuals’ with both chronic (CM) and episodic (EM) migraine from the US and the UK.

**Methods**

Individuals with migraine registered to use Curelator Headache (1) through a physician ‘coupon referral’ program, (2) via the Curelator website or (3) the iOS App Store and and was used daily for at least 90 days entering details about headaches and medications used acutely and chronically. Acute medication use was analyzed at the level of individual drug names and MO was defined according to ICHD-3 beta criteria; other reported medication was not included in the analysis.

**Results**

Individuals from the USA (*n* = 261) and the UK (*n* = 216) entered 20,353 (USA) and 17,965 (UK) headache instances. Only 6 (2.3%) US and 4 (1.8%) UK users did not use any acute medication for their headaches.

The average number of medications *per user* was significantly higher for CM than EM but significantly higher in EM than in CM group in both countries on a *per headache* basis (Table 1).

MO was significantly higher in the US EM (21%) than the UK EM group (12%). MO groups have similar usage of medication, but CM & no MO users typically use much less medication per headache than their EM & no MO peers.

Triptans and NSAIDs are most frequently used by EM users in both countries and significantly more frequently than in CM cohorts. NSAIDs are most frequently used by CM users in both countries. While usage of analgesic combinations by CM users is similar in both countries, UK CM users use triptans more frequently than in the US. Opioids were used more frequently by US CM users than UK CM users.

**Conclusion**

Curelator Headache identified MO in 29% of individuals in a predominantly physician referred group (US) and in 19% in a predominantly population recruited group (UK). CM and EM users showed different frequency of medication use and overuse, similar to previously reported [2]. The higher ratio of medication use in the EM MO subgroup compared to the CM MO subgroup indicates that a MO pattern may be identifiable in EM individuals.

**Table 1 (abstract P59).** Medication use description

Variable / Cohort	US						UK					
	CM	EM	CM+MO	CM+nMO	EM+MO	EM+nMO	CM	EM	CM+MO	CM+nMO	EM+MO	EM+nMO
Average medication classes per user	2.45	2.19	2.80	1.89	2.53	2.10	2.82	2.45	3.11	2.53	2.48	2.45
Average medications per user	3.55	2.81	4.13	2.63	3.33	2.67	3.58	2.91	4.26	2.89	2.95	2.90
Average medications classes per headache	0.81	0.94	1.16	0.24	1.19	0.83	0.72	1.01	1.04	0.37	1.25	0.96
Average medications per headache	0.86	0.97	1.23	0.24	1.22	0.84	0.74	1.02	1.07	0.38	1.28	0.97

**References**

[1] Tassorelli C, et al. The added value of an electronic monitoring and alerting system in the management of medication-overuse headache: A controlled multicentre study. Cephalalgia. 2016 Jul 20

[2] Scher AI et al. Patterns of medication use by chronic and episodic headache sufferers in the general population: results from the frequent headache epidemiology study. Cephalalgia. 2010 Mar;30(3):321-8

### P60 Individual self-prediction of migraine attacks: longitudinal analysis of cohort of migraine patients using a digital platform

#### Pablo Prieto^1^, Gabriel Boucher^1^, Alec Mian^1^, Noah Rosen^2^

##### ^1^Curelator Inc., Cambridge; ^2^Northwell Health, New York, United States

###### **Correspondence:** Pablo Prieto

**Background:** As a critical component towards self-management of their condition we examine the individual ability of patients to predict their attacks 24 hrs in advance. Prediction of attacks might be expected to be difficult as migraine premonitory symptoms, and the potential risk factors that trigger them, show significant inter-individual variation [1] and possibly also intra-individual variation.

Accurate prediction may impact quality of life, allow optimal timing of medication dosing and may also lead to understanding of the profiles and “best practice” of good predictors. Thus, the objective is to understand and compare ability of episodic migraineurs to self-predict attacks on an individual level.

**Methods:** Individuals with migraine registered to use a digital platform (Curelator Headache™) via website or the App Store (iOS only) and on a daily basis for at least 90 days entered about lifestyle factors, possible headaches, and medications as well as migraine expectation for the next 24 hours (low/moderate/high). Patients with at least 10 low and 10 high expectations instances of migraine were included in the analysis. Prediction was considered successful when 24hr expectation of migraine was high and an attack occurred on the next day; or 24 hr expectation was low and was followed by a migraine free day.

**Results:** Of 497 episodic migraineurs examined in the study, 146 met the criteria for analysis. Good predictors were defined as having an accuracy of ≥75% at predicting an attack or a migraine-free day; bad predictors were defined as those with ≤25% accuracy predicting a migraine or a migraine-free day.

In this study we found 9% (*n*= 13) were good migraine predictors and 27% (*n*=40) were defined as bad migraine predictors (Table 1), and both groups stood up as different from the rest of the sample with statistical significance (*p*<0.001). Only 5% (*n*=7) were good migraine and migraine-free predictors.

**Conclusion:** A substantial proportion (64%, *n*= 93) of users predict their migraine with only moderate accuracy (>25% but <75%). A small group (27%, *n*=40), were considered bad predictors with ≤25% accuracy. A smaller group (9%, *n*=13) were found to be good predictors with ≥75% accuracy and just 5% (*n*=7) were able to accurately predict migraine or migraine-free days. A next step would be to understand the in possible differences risk factors and premonitory symptoms that these two groups may exhibit and are possibly using for prediction of their attacks.

**Table 1 (abstract P60).** Ability to predict migraine or a migraine-free day

	Good predictors (≥75% accuracy)	Rest	Bad predictors (≤25% accuracy)
Migraine prediction	9%	64%	27%
Migraine free prediction	71%	28.3%	0.7%
Both	5%	95%	0%

**References**

[1] Peris F, Donoghue S, Torres F, Mian A, Wöber C. Towards improved migraine management: Determining potential trigger factors in individual patients. Cephalalgia. 2017 Apr;37(5):452-463

### P61

**Withdrawn**

### P62 A rapidly progressive cerebral hematoma secondary to cerebral venous sinus thrombosis (CVST) - A Case Report

#### Andrea Giorgetti (andrea.giorgetti@asst-ovestmi.it), Elena P. Verrengia, Patrizia Perrone

##### Department of Neuroscience, Neurology and Stroke Unit, Legnano Hospital, Italy

**Background**

Intracranial hemorrhage (ICH) accounts for up then 15% of strokes. When hemorrhage is secondary to cerebral venous sinus thrombosis (CVST) the diagnosis is sometimes difficult to establish. The early diagnosis of CVST is important to ensure best management including best medical treatment, mechanical trombectomy or surgical interventions.

**Case Report**

A 51 year-old woman was admitted in Emergency Room with unusual acute severe headache and seizure involving left-side limbs. Recent past medical history was significant for ulcerative colitis with ongoing steroid and anti-inflammatory therapy. Neurological evaluation revealed facial left nerve palsy and mild left upper limb weakness without Brudzinski and Kernig’s signs and no papilledema on clinical examination. A cerebral computed tomography (CT) scan showed hyperdensity of parietal right lobe such as intraparenchymal hemorrhage with surrounding large hypodensity area. The day after a cerebral magnetic resonance imaging (MRI) revealed a large parenchymal hematoma of right parietal-occipital lobe and surrounding hypodense ischemic injury with initial “mass effect”; a magnetic resonance imaging with angiovenous sequences showed a filling defect of transverse and sigmoid right sinuses.. The patient was clinically unchanged with persistent headache (VAS 8/10) but alert, oriented with slight life-sided deficits. Suddenly she was treated with low-molecular-weight heparin (LMWH) high dose therapy. After 48 hours of hospitalization, she developed hyperpyrexia, hemiplegia of left limbs and became comatose due to a “mass effect” produced by unilateral edematous venous infarction and parenchymal hemorrhage detected by cerebral CT. Decompressive surgery with hematoma evacuation was performed with good outcome. Medical treatment after surgery included steroid, anticonvulsants, anticoagulation. 3 months later the patient presented a nearly complete recovery (median Rankin Scale score 1): she was able to walk and to perfom basic daily activities.

**Conclusions**

CVST is a possible cause of cerebral venous infarct or parenchymal hematoma. Headache represents the most common symptoms of this condition (80-90% of patients); underlyng risk factors to develop CVST could be inflammatory bowel disease such as ulcerative colitis and treatment like corticosteroids. The use of MRI is essential for identify CVST and understand the location, volume and severity of hemorrhage. When CVST is suspected, is important to avoid a delay in diagnosis and treatment. Mortality in untreated cases ranges from 13 to 48%of patients and is due to large parenchymal lesions causing herniation. Decompressive surgery in these cases is lifesaving and related to good functional outcome even in patients with severe clinical conditions.

**References**

1. Heit Jeremy J, Iv Michael, Wntermark Max. Journal of Stroke. 2017;19(1):11-27

2. Ferro Jose’ M, Crassard Isabelle, Coutinho Jonathan M, Canha˜ Patrícia, Barinagarrementeria Fernando, Cucchiara Brett, Derex Laurent, Lichy Christoph, Masjuan Jaime, Massaro Ayrton, Matamala Gonzalo, Poli Sven, Saadatnia Mohammad, Stolz Erwin, Viana-Baptista Miguel, Stam Jan, Bousser Marie-Germaine. Second International Study on Cerebral Vein and Dural Sinus Thrombosis (ISCVT 2) Investigators. Stroke. 2011;42:2825-2831.

### P63 Benign intracranial hypertension in children can be due to hypoparathyroidism: a case-report

#### Giorgia Sforza^1,3^, Annalisa Deodati^2^, Laura Papetti^1^, Barbara Battan^1^, Paolo Curatolo^3^, Federico Vigevano^1^, Massimiliano Valeriani^1^

##### ^1^Headache Center, Child Neurology Unit, Bambino Gesu’ Children’s Hospital, Rome, Italy; ^2^Child Endocrinology Unit, Bambino Gesu’ Children’s Hospital, Rome, Italy; ^3^Child Neurology and Psychiatry Unit, Tor Vergata University of Rome, Italy

###### **Correspondence:** Giorgia Sforza (sforzagiorgia@gmail.com)

**BACKGROUND**

To present the rare case of a 9-year-old girl with idiopathic intracranial hypertension (IIH) secondary to hypoparathyroidism (HPTH) and our work-up, including physical examination, blood tests, diagnostic imaging, and lumbar puncture.

**CASE REPORT:**

We present a 9-year old female patient who was hospitalized for headache associated with nausea and vomiting for 3 weeks. She underwent ophthalmologic examination which showed papilledema. She had never had cramps, paraesthesias or tetany. Lumbar puncture (LP) revealed an opening pressure of 65 cm H_2_O. CSF analysis and brain CT scan were normal. The patient was started on acetazolamide 375 mg/die. However, a low serum calcium level (6.3 mg/dL) was found, thus leading us to suspect HPTH. Indeed, phosphorus was 10.2 mg/dL, parathormone was very low (3 pg/mL). Chvostek and Trousseau signs scored positive. Neck ultrasonography showed normal thyroid, while parathyroids were not viewable. Oral supplementation with calcitriol (0.50 mcg/day) and calcium (500 mg/day) was started.

**CONCLUSIONS**

IIH is defined as an elevated intracranial pressure (>25 cmH2O) without clinical, laboratory or radiological evidence of hydrocephalus, infection, tumor or vascular abnormality. Annual incidence is 1-2 per 100,000. Several hypotheses have been proposed for the IIH pathophysiology, but none of them has reached a general consensus. Rare cases of IIH secondary to HPTH have been described ^[1]^. It is supposed that hypocalcemia causes a decrease in the CSF absorption at level of the arachnoidal granulations ^[2].^ Interestingly, our patient did not present with the typical neurological HPTH symptoms, such as tetany, cramps, paraesthesias, seizures, behavioral disorders, and intracranial calcifications. Only the serum calcium dosage led us to suspect this condition. Therefore, we recommend that possible HPTH should be always checked in children with clinical findings of benign intracranial hypertension.

**Consent to publish**

Written informed consent has been obtained from the parents

**References**

1. Aragones JM, Alonso-Valdés F. Hipertensiòn intracraneal benigna secundaria a hipoparatiroidismo. Rev Neurology 2014; 58: 94.

2. Sambrook MA, Hill LF. Cerebrospinal fluid absorption in primary hypoparathyroidism. J Neurol Neurosurg Psychiatry 1977; 40:1015-7.

### P64 Headache following head injury: A population-based longitudinal cohort study (HUNT)

#### Lena Hoem Nordhaug^1^; Knut Hagen^1, 2^; Anne Vik^1, 3^; Lars Jacob Stovner^1, 2^; Turid Follestad^4^, Torunn Pedersen^5^; Gøril Bruvik Gravdahl^2^, Mattias Linde^1, 2^

##### ^1^Department of Neuromedicine and Movement Science (INB), Faculty of Medicine and Health Sciences, Norwegian University of Science and Technology, Trondheim, Norway; ^2^Norwegian Advisory Unit on Headaches, St. Olavs University Hospital, Trondheim, Norway; ^3^Department of Neurosurgery, St. Olavs University Hospital, Trondheim, Norway; ^4^Department of Public Health and Nursing, Faculty of Medicine and Health Sciences, Norwegian University of Science and Technology, Trondheim, Norway; ^5^Division of Mental Health and Addiction, Oslo University Hospital, Oslo, Norway

###### **Correspondence:** Lena Hoem Nordhaug (lena.h.nordhaug@ntnu.no)

**Objective**

To explore whether subjects exposed to head injury more often developed a new headache or experienced exacerbation of previously reported headache compared to non-exposed.

**Methods**

This population-based historical cohort study included headache data from two large epidemiological surveys performed with an 11-year interval. This was linked with data from hospital records on exposure to head injury occurring between the health surveys. Participants in the surveys who had not been hospitalized because of a head injury comprised the control group. The head injuries were classified according to the Head Injury Severity Scale (HISS). Multinomial logistic regression was performed to investigate the association between head injury and new headache or exacerbation of pre-existing headache in a population with known pre-injury headache status, controlling for potential confounders.

**Results**

The exposed group consisted of 294 individuals and the control group of 25,662 individuals. In multivariate analyses, adjusting for age, sex, anxiety, depression, education level, smoking and alcohol use, mild head injury increased the risk of new onset headache suffering (OR 1.74, 95% CI 1.05 – 2.87), stable headache suffering (OR 1.70, 95% CI 1.15 – 2.50) and exacerbation of previously reported headache (OR 1.93, 95% CI 1.24 – 3.02). The reference category was participants without headache in both surveys.

**Conclusion**

Individuals exposed to a head injury were more likely to have new onset and worsening of pre-existing headache and persistent headache, compared to the surrounding general population. The results support the entity of the ICHD-3 beta diagnosis “persistent headache attributed to traumatic injury to the head”.

### P65 Pediatric headache clinics: toward in-pediatric emergency department models

#### Laurence Geffroy^1^, Silvana De Lucia^1^, Luigi Titomanlio^1,2^

##### ^1^APHP-Hospital Robert Debré, Department of Pediatric Emergency Care, APHP-Hospital Robert Debré, Paris, France; ^2^APHP-Hospital Robert Debré, Pediatric Migraine and Neurovascular Diseases Unit, Paris, France

###### **Correspondence:** Laurence Geffroy

**BACKGROUND**: Headache is a frequent pediatric complaint in the emergency department (ED). Although medical protocols are available at the ED, diagnosis and therapy of children with headache are challenging, due to wide spectrum of headache aetiologies. Aim of the study was to analyze the impact of the creation of a Headache Clinic in our pediatric university hospital.

**METHODS**: Review of all medical records of children 3-18 years consulting at the PED for headache as the main complaint, starting from Jan 2011, when the Headache Clinic was created, until Dec 2016. Statistical analysis was performed by GraphPad Instat 3.10.

**RESULTS**: Over the study period, 502,456 patients consulted our PED, of whom 5,185 for headache (1%). Characteristics of these patients and results of statistical analysis are summarized in Table 1.

**CONCLUSIONS**: The creation of a Headache Clinic in our hospital allowed an improvement of the quality of care in our region, reducing the delay of specialist assessment. On the other hand, although the number of headache patients consulting at the PED did not significantly increase, we observed a significant temporal trend in terms of headache severity at triage, an increased length of stay, more complementary investigations, and increased admission rate. So, the positive effects of the creation of the Headache Clinic are actually counterbalanced by PED overload.

To continue caring headache children with high standards, the next step would be the implementation of the Headache Clinic in the PED. Although this may require some structural costs to provide a dedicated area, health benefits would be huge. The presence of a headache team at the PED (doctors, nurses, psychologists) would result in an important gain of time for emergency physicians, in the reduction of the length of stay at the PED (less waiting time for specialists, reduction of unuseful tests), in a better multidisciplinary care, and in the reduction of parental stress (with reduction of return visits and medical shopping). Prospective analysis at both short- and long- term is required before concluding about the usefulness of this approach and of its generalisability to other PEDs.

**Table 1 (abstract P65).** See text for description

	2011	2012	2013	2014	2015	2016	*p*
PED consultants for headache (% on total)	647 (0.8%)	829 (1%)	824 (1%)	955 (1.1%)	933 (1.1%)	997 (1.1%)	-
Male gender	360 (55.6%)	453 (54.6%)	440 (53.4%)	490 (52.1%)	498 (53.4%)	527 (52.9%)	-
Age (mean, years)	8.9	9	9.2	9.3	9.3	9.8	<.001
Arrival during night shift	273 (42.1%)	298 (35.9%)	266 (32.3%)	318 (33.2%)	312 (33.4%)	318 (31.8%)	-
Arrival during weekend shift	153 (23.6%)	210 (25.3%)	219 (26.5%)	248 (25.9%)	233 (25%)	244 (24.5%)	-
*Triage severity*							-
Low	309 (47.8%)	404 (48.8%)	394 (47.8%)	430 (45%)	404 (43.3%)	429 (43.1%)	
Intermediate	272 (42 %)	326 (39.3%)	322 (39%)	385 (40.3%)	383 (41.1%)	397 (39.8%)	
Urgent	66 (10.2%)	99 (11.9%)	108 (13.2%)	40 (14.7%)	146 (15.6%)	171 (17.1%)	<.05
Context of head trauma	122 (18.9%)	198 (23.9%)	193 (23.4%)	224 (23.5%)	184 (19.7%)	193 (19.7%)	
Dismissed without investigations	392 (60.6%)	411 (49.6%)	400 (48.6%)	387 (40.5%)	417 (44.7%)	382 (38.3%)	<.05
Head CT scans at PED (% on all headaches)	29 (4.5%)	14 (1.7%)	25 (3%)	23 (2.4%)	16 (1.7%)	39 (3.9%)	
Admission following PED (% on all headaches)	40 (6.2%)	48 (5.8%)	52 (6.3%)	58 (6.1%)	91 (9.8%)	100 (10%)	<.05
Length of stay at the PED (mean, min)	197	193	205	203.5	211.4	230.2	<.0001

### P66 Individual identification of factors associated with reduced risk of migraine attacks: potential ‘protectors’

#### Stephen Donoghue^1^, Francesc Peris^1^, Gabriel Boucher^1^, Alec Mian^1^, Christian Wöber^2^

##### ^1^Curelator Inc, suite 4503, 5th floor, 195 Binney, Cambridge, MA, USA 02142; ^2^Headache Group, Department of Neurology, Medical University of Vienna, Vienna Austria

###### **Correspondence:** Stephen Donoghue

**Background**

Curelator Headache™ is a digital platform that collects daily data about an individual’s migraine attacks and up to 80 factors (mood, stress, diet, activity, weather etc.) which may affect their occurrence and then statistically identifies factor-attack associations. Hence we identify potential triggers (both “true” triggers and premonitory symptoms) [1]. Our analysis also identifies factors associated with *reduced* risk of attack occurrence: we call these potential ‘protectors’ [2]. Here we compare potential protectors in individuals with episodic (EM) or chronic (CM) migraine.

**Methods**

Individuals with migraine registered to use Curelator Headache via 1) a physician ‘coupon referral’ program, 2) the Curelator website or 3) the App Store (currently iOS only). They used Curelator Headache daily for 90 days, entering details about headaches and tracking factors that may affect migraine attack occurrence, after which factor-attack associations were analyzed for each individual [1].

**Results**

557 individuals were included: demographic data are shown in Table 1:

Overall, potential protectors were found in 471 (84.4%) individuals: on average individuals had 3.0 (CI 0.20) such associations; range 0 - 16. The factors most commonly found (>5% individuals) were physical activity, happiness, being relaxed, sleep quality, sleep duration, sleep quality, waking refreshed, caffeine, mean outside temperature and humidity, time outdoors.

In general the same factors appeared as ‘protectors’ in both groups, although for some there was a higher proportion of EM individuals, possibly reflecting real differences or increased difficulty of identifying risk factors in individuals with frequent attacks.

**Conclusions:**

Identifying ‘protectors’ may be clinically useful for people with migraine. However it is important to guide each individual to think about their behaviours and self-analyse *the context* in which ‘protectors’ appear to affect their condition. For example, apparent ‘protectors’ may be due to avoidance immediately before/during attacks (e.g. nicotine, physical activity) or to actions taken to mitigate a factor effect (e.g. avoiding going out on hot days).

Our data support what many clinicians and people with migraine believe intuitively, i.e that moderate activity, staying relaxed, and trying to maintain good sleep hygiene reduce risk of attacks. In addition warm weather may have a protective role. Caffeine appears protective for some people (but can be a trigger for others).

**Fig. 1 (abstract P66).**
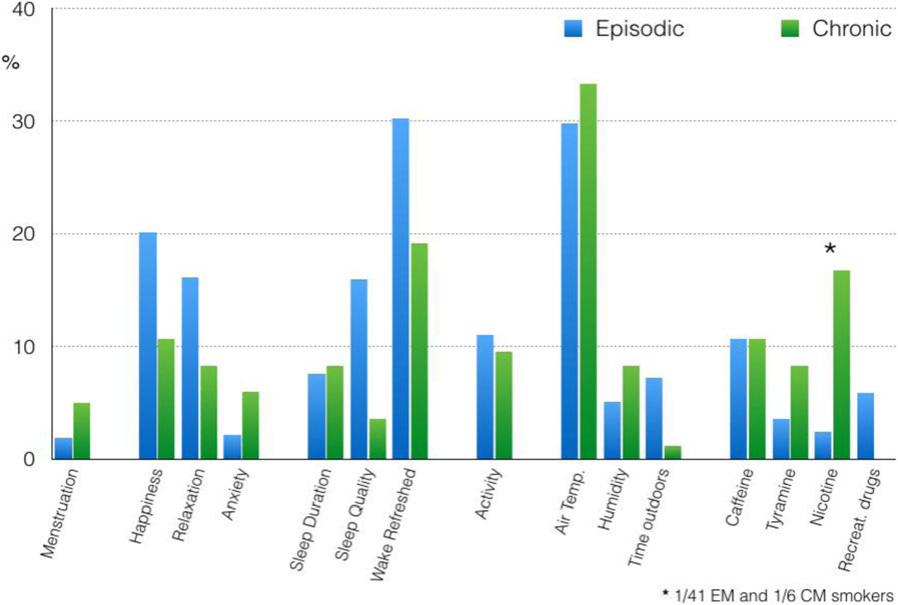
Shows the proportion of individuals in each group for whom the most common ‘protectors’ were identified

**Table 1 (abstract P66).** Demographics

	All	EM	CM
Total number in population	557	473	84
Mean Age (years) (SD)	42.9 (13.4)	43.5 (13.2)	39.4 (14.2)
Age range (years)	11 - 75	11 -75	15 - 69
No. Females (%)	488 (87.6%)	409 (86.5%)	79 (94%)
Female - menstrual cycles (%)	292 (52.4%)	240 (50.7%)	52 (61.9%)
Female - menopausal (%)	90 (16.2%)	80 (16.9%)	10 (11.9%)
Female - non-menstrual or menopausal (%)	106 (19.0%)	89 (18.8%)	17 (20.2%)

**References**

1. Peris F et al. Towards improved migraine management: determining potential trigger factors in individual patients. Cephalalgia 2016; 37(5): 452-463

2. Donoghue S et al. Identification of ‘protectors’ - factors associated with reduced risk of migraine attacks: some surprising observations and interpretations. Headache 2016; 56 Suppl.1: 36.

### P67 Self-reported triggers vs prospectively statistically determined factors associated with attacks in individuals with episodic and chronic migraine

#### Stephen Donoghue^1^, Gabriel Boucher^1^, Francesc Peris^1^, Alec Mian^1^ and Paul R. Martinl^2^

##### ^1^Curelator Inc., Cambridge, MA, USA; ^2^School of Applied Psychology, Griffith University, Queensland, Australia

###### **Correspondence:** Stephen Donoghue

**Background**

In this study we compare in individuals with episodic (EM) or chronic (CM) migraine their suspected, self-reported triggers *versus* risk factors identified statistically. Migraine attacks may be triggered by combinations of internal and external factors which differ markedly between individuals [1]. Most migraineurs suspect a range of triggers, usually based on a combination of popular literature and (unreliable) retrospective recall which is subject to misinterpretation and recall bias. Few have been tested for causality but, when done, triggering of attacks has usually been inconsistent and avoidance has had limited effect [2]. Previously we reported that less than 20% of suspected self-reported triggers were shown to be statistically associated with attacks when using individuals’ *prospective* data collected using a digital platform (Curelator Headache™) [3].

**Methods**

Individuals with migraine registered to use Curelator Headache via 1) a physician ‘coupon referral’ program, 2) the Curelator website or 3) the App store (currently iOS only) and completed a questionnaire about personal suspected triggers and their importance (1=low; 10=maximal). They used Curelator Headache daily for 90 days, entering details about headaches and tracking factors that may affect migraine attack occurrence. After 90 days factors were analyzed for each individual [4] and those significantly associated with attacks were compared with self-reported triggers.

**Results**

488 individuals were included: demographic data are shown in Table 1:

Overall, individuals each suspected between 3 and 44 different triggers; mean (SD) = 23.2 (12.4). Most frequently, and often most strongly, suspected were: menstruation, stress, anxiety, irritability, angriness, sleep quality and duration, neck pain, eye strain, bright lights, odors, loud noise, fatigue, activity, travel, weather, dehydration, missed meals, alcohol.

Table 2 shows how many *suspected* triggers were confirmed statistically or shown to have no association with migraine attack risk.

The CM group overall *suspected* more triggers per person but fewer of these were statistically associated with migraine attacks (*p* = 0.008). In the EM group stress, anxiety, sadness, tiredness/fatigue, neck pain/tension, eye strain head/neck skin sensitivity and menstruation were more commonly confirmed as potential triggers.

**Conclusions**

EM and CM groups differ in the proportion of suspected risk factors (potential triggers) confirmed statistically. This may represent real differences between the groups, i.e ability to self-recognise triggers correctly, or difficulty in detecting some associations in the CM group. Nevertheless, the difference contrasts with the lack of differences in self-reported suspected triggers between EM and CM groups.

**Table 1 (abstract P67).** Demographics

	All	EM	CM
Total number in sample	488	391	97
Mean Age (years) (SD)	43.0 (13.4)	43.7 (13.2)	40.3 (14.0)
Age range (years)	11 - 75	11 -75	15 - 69
No. Females (%)	440 (90.2%)	348 (89.0%)	92 (94.8%)
Female - menstrual cycles (%)	275 (56.4%)	215 (55.0%)	60 (61.9%)
Female - menopausal (%)	87 (17.8%)	72 (18.4%)	15 (15.5%)
Female - non-menstrual or menopausal (%)	78 (16.0%)	61 (15.6%)	22 (22.7%)

**Table 2 (abstract P67).** See text for description

	All (N = 488)	EM (N = 391)	CM (N = 97)
Mean no. suspected per individual (95% CI) (range)	23.2 (1.1) (3 - 44)	22.9 (1.2) (3 - 44)	24.3 (2.4) (5 - 43)
% of total suspected triggers identified statistically	11.5	12.2	8.9
Mean no. of *previously suspected* triggers statistically identified (95% CI) (range)	2.7 (0.21) (0 - 12)	2.8 (0.24) (0 -12)	2.2 (0.39) (0 - 8 )
Mean no. of *previously suspected* triggers with no association with attacks (95% CI) (range)	14.2 (0.74) (0 - 33)	13.8 (0.81) (0 - 33)	15.5 (0.74) (1 - 33)
Mean no. *previously suspected* triggers with insufficient data to determine statistical association (95% CI) (range)	5.1 (0.37) (0 - 23)	5.0 (0.42) (0 - 23)	5.4 (0.74) (0 - 18)
Mean no. of *unsuspected* triggers statistically identified (95% CI) (range)	1.3 (0.15) (0 -9)	1.4 (0.17) (0 - 9)	1.1 (0.30) (0 - 9)

**References**

1. Spierings EL et al. Sufficiency and necessity in migraine: how do we figure out if triggers are absolute or partial and, if partial, additive or potentiating? Curr Pain Headache Rep 2014; 18: 455-461.

2. Martin PR. Behavioural management of migraine headache triggers: learning to cope with triggers. Curr Pain Headache Rep 2010; 14: 221-227.

3. Donoghue S et al. Migraineurs’ suspected triggers compared with scientifically determined associations using a daily diary and statistical analysis platform. Headache 2016; 56 (Supp 1): 35.

4. Peris F et al. Towards improved migraine management: determining potential trigger factors in individual patients. Cephalagia 2016; 37(5): 452-463

### P68 Vertigo and Migraine

#### Elena Filatova, Tatiana Ivanova

##### Institute of Professional Education, Chair of Neurology. I.M. Sechenov First Moscow State Medical University 8-2 Trubetskaya st., Moscow 119991, Russia

###### **Correspondence:** Tatiana Ivanova (itamail@mail.ru)

Vestibular migraine (VM) is the most common cause of episodic vertigo in adults as well as in children. (1).The exact neural mechanisms of vestibular migraine are still unknown. Vestibular migraine often begins several years after typical migraine and has a variable clinical presentation. The neurological and otoneurological examination of patients with vestibular migraine is mostly normal and diagnosis are usually based on clinical history. Treatment trials of vestibular migraine are scarce and therapeutic recommendations are based on migraine guidelines.

**Objective:** The aim of study is to determine the mechanisms of vertigo and dizziness in migraine patients

**Methods:** This study recruited 152 patients with migraine with aura and migraine without aura as defined by ICHD-3. VM was diagnosed by (ICHD-3) Diagnostic criteria.(2) We analyzed the medical history of migraine patients with vertigo and dizziness, all patients had a neurological examination, and questionnaire survey by scales: Dizziness Handicap Inventory (DHI) scale (3)), HADS , HIT-6. Patients with vertigo additionally had an otoneurological examination for excluding peripheral vestibulopathy.

**Results:** The main study group consisted of patients with vestibular migraine (VM) – 13,8% (n=21), and controls were patients with dizziness - 42,2% (n=64) . There were no significant differences in sex, age, VAS, HADS , HIT-6 in main and control group . Chronic migraine and migraine without aura was more prevalent in patients with VM ( СМ: 88.8%, n=16 vs. 36.4%, n=12, respectively). DHI-positional scale in VM was higher, then in dizziness patients (15,43±5,9 vs 12,28±6,9,p<0,05) Vertigo manifest during migraine attack and dizziness was permanent. VM patients have more often motion sickness , subjective hearing loss, although otoneurologist found no current disease. More then 50% of VM patients had family history of hearing loss or dizziness.

**Conclusion:** VM is mostly observed in patients with chronic migraine and migraine without aura. Our study suggests the role of central sensitization of the vestibular nuclei in the patients with preexisting pathology of the vestibular system in VM pathogenesis. The leading part, of central sensitization in the genesis of vertigo in migraine patients are warranted to pave the way to potential new therapies for this condition.

**References**

1. Stolte B., Holle D., Naegel S., Diener H.C., Obermann M. Vestibular migraine. Cephalalgia. 2014

2. The International Classification of Headache Disorders, 3rd edition (beta version).Cephalalgia. 2013;33(9):629-808.

3. Jacobson GP, Newman CW. The development of the Dizziness Handycap Inventory. Arch Otolaryngol Head Neck Surg. 1990, 116(4): 424-7.

### P69 Characterising the effect of lumbar puncture on headache in idiopathic intracranial hypertension

#### Andreas Yiangou^1,2^, James Mitchell^1,3^, Keira Annie Markey^1,2^, William Scotton^1,2^, Peter Nightingale^5^, Ryan Ottridge^6^, Susan Mollan^1,4^, Alexandra Sinclair^1,3^

##### ^1^Institute of Metabolism and Systems Research, University of Birmingham, Edgbaston, B15 2TT, UK; ^2^Centre for Endocrinology, Diabetes and Metabolism, Birmingham Health Partners, B15 2TH, UK; ^3^Department of Neurology, University Hospitals Birmingham NHS Foundation Trust, B15 2TH, Birmingham, UK; ^4^Birmingham Neuro-Ophthalmology Unit, Ophthalmology Department, University Hospitals Birmingham NHS Foundation Trust, B15 2TH, Birmingham, UK; ^5^Wellcome Trust Clinical Research Facility, Queen Elizabeth Hospital, Birmingham, UK; ^6^Birmingham Clinical Trials Unit, School of Cancer Sciences, Robert Aitken Institute, University of Birmingham, Birmingham, UK

###### **Correspondence:** Andreas Yiangou (a.yiangou@bham.ac.uk)

**Introduction**

Headache is the most prevalent symptom in idiopathic intracranial hypertension (IIH). This study aimed to evaluate the temporal change in headache severity in the week following lumbar puncture (LP) in patients with active IIH.

**Methods**

Headache severity was prospectively recorded using the numeric rating scale (NRS) 0 (no pain) to 10 (most severe pain) immediately prior to and following LP (1,4 and 6 hours and then daily to 7 days). Demographic data and variables hypothesised to impact on the post-LP headache severity were recorded.

**Results**

52 IIH patients were recruited with mean BMI 41 ± 10 Kgm^-2^, LP opening pressure 33.2 ± 6.1 cmCSF and headache severity 3.6 ± 2.8. Exacerbation of headache was noted in 64% with 30% experiencing an exacerbation ≥4 NRS. In the whole cohort, a small improvement in headache severity was noted 1 hour in 58% (-1.1 ± 2.6 NRS (p<0.001)) and this was maintained at 7 days in 47% (-1.0 ± 2.7 NRS (p=0.004)). In those with severe headaches pre-LP (NRS 7-10), 75% improve at 1 hour (-3 ± 3.7 NRS, p=0.024) and 67% improved at 7 days (-3.0 ± 2.8 NRS, p=0.012) whilst deterioration was uncommon (8% at 1 hour and zero at 7 days). In those with moderate headaches pre-LP (NRS 4-6), 91% improve at 1 hour (-2.2 ± 1.6 NRS, p<0.001) and 61% improved at 7 days (-1.7 ± 2.3 NRS, p=0.007) and deterioration was uncommon (4% at 1 hour and 22% at 7 days). Amongst, those with mild headaches pre-LP (NRS 1-3), no significant improvement was noted and the likelihood of deterioration was higher (50% at 1 hour and 19% at 7 days). In those with no headache pre-LP, 20% will experience deterioration at 1 hour and 27% at 7 days. There was no relationship between the response of the headache severity post-LP and BMI, height, skin to dura depth, LP opening or closing pressure, CSF volume withdrawn, number of LP attempts, CSF red blood cell count, acute analgesics, acetazolamide use and Frisén papilloedema grade.

**Conclusion**

The majority of IIH patients will experience deterioration in headache at some point during the week post-LP. Headache severity pre-LP significantly influenced the likelihood of improving or deteriorating after LP. Additionally, we noted that the improvement at 1 hour post-LP was maintained at 7 days. Characterisation of headache outcomes post-LP in IIH has relevance when counselling patients about the procedure.

**Trial registration**

ClinicalTrials.gov Unique identifying numbers: NCT02017444, NCT02124486.

### P70 Effectiveness of a digital platform for headache training of specialists

#### Edvige Correnti^1^, Filippo Brighina^2^, Antonino Sandullo^3^, Marcello Romano^4^, Francesca Marchese^1^, Carmela Loiacono^1^, Luca Messina^11^, Vincenzo Raieli^5^

##### ^1^Child Neuropsychiatry School – University of Palermo –Italy 90100; ^2^Department of Experimental Biomedicine and Clinical Neurosciences - University of Palermo-Italy 90100; ^3^Primary Care Dept. –ASP 1 Agrigento- Italy 92100; ^4^Neurology Dept.- Cervello-Villa Sofia Ospedali Riuniti –Palermo –Italy 90100; ^5^UO NPI- P.O. Cristina - ARNAS Civico Palermo –Italy 90100

###### **Corrispondence:** Vincenzo Raieli (v.raieli@alice.it)

**Background.** Headache represents a peculiar condition, that is often underdiagnosed and undertreated, with remarkable dissatisfaction of the patients. An adequate training is crucial for all the headache specialists[1,2]. Considering the unsatisfying results obtained with standard updating courses and the rising need of a continuous training, a digital platform was developed in the last two years as a tool of update.

**Methods.** A digital platform, has been activated since the 1^st^ October 2014. The platform is easily accessible to doctors by free membership, validated by the administrators of the group. Repeatedly in two years Administrators invited to subscription members of scientific societies potentially interested to headache. The users have access to the platform’s whole material, that includes scientific articles, e-books, presentations and images. They can directly share their material, discuss on clinical cases and submit new ones. The system allows to monitor the participation of members in terms of platform access, resource upload and / or download, introduction of cases and participation in their discussion. Finally, we sent a questionnaire to understand the reasons of use or not use of platform by registered members.

**Results .**The platform currently boasts 39 members. The resources in the platform consist of many different materials concerning headache ( more than 100 articles dedicated to migraine aura, several books, many slide libraries on different headache topics, collections of artistic or neuroimaging images related to headaches etc...). In the last year 316 files have been downloaded, 5 discussions have been started with 22 contributors. 15 of 37 members have never carried out any action. The last year number of uploaded files amounts to 74, but 90% of the contributions is due to a restricted group. There were no significant differences in use of platform between members of society for study of headache and other specialists (see Table 1). The results of questionnaire show summarily that first reason of non-use of platform is the lack of time.

**Conclusions.** After 2 years of activity, a the data show that about 40% of the members have never carry out any operation and the remaining mainly used the platform in a passive way, through the resources downloading. Even if the platform appears to be an easily accessible, interactive and not expensive instrument, the passive use suggests that a critical aspect for medical education on the diagnosis and treatment of headaches lies more in the doctors’ ability to invest themselves emotionally on these issues.

**Table 1 (abstract P70).** Utilization of Headache digital platform by members

Academic groups	members	Members with no actions performed	Login	Downloaded files	Uploaded files	Clinical cases submitted	Contribution on clinical cases
Sisc (Italian society for the study of headache) members	18	5	112	146	68	4	20
SINPIA (Italian society of neuropsychiatry for childhood and adolescence) members	11	6	28	139	3	1	2
Other specialists	8	4	7	31	3	0	0
Total	37	15	147	316	74	5	22

**References**

1. Raieli V. ,Compagno A., Puma D., La Vecchia M., Pandolfi E., La Franca G., Ragusa D. Headache: What do children and mothers expect from paeditricians? Headache, 2010, 50: 2, 290-300;

2. Brighina F., Raieli V., La Pegna G., Lanaia F.. Disability and social impact of headaches and migraine. The role of information and cooperation among patient, general practitioner and specialist: A project of the SISC Sicilia. Giornale delle Cefalee.2006; 2:10-12.

### P71 Tanacethum Parthenium , 5 - hydroxy tryptophan and magnesium (Aurastop©) efficacy in episodic migraine prevention. A multicentric observational study

#### Federico Mainardi^1^, Paola Merlo^2^, Ferdinando Maggioni^3^, Giorgio Zanchin^3^, Giorgio Dalla Volta^4^

##### ^1^Headache Centre, Neurological Division, SS Giovanni e Paolo Hospital, Venice; ^2^Headache Centre of Neurological Division of Gavazzeni Hospital, Bergamo; ^3^Headache Centre, Department of Neurosciences, Padua University, Padua; ^4^Headache Center of Neurological Unit of Istituto Clinico Citta’ di Brescia, Brescia

###### **Correspondence:** Giorgio Dalla Volta (dalla@numerica.it)

**Background**: Each component of the novel phytotherapic combination of Tanacethum Parthenium (150 mg), 5-hydroxy tryptophan (20 mg) and magnesium (185 mg) (Aurastop©) acts on a different target among the main mechanisms involved in the pathophysiology of migraine: sensitization of trigeminal vascular system, central sensitization and activation of the “migraine generator” located in the brainstem, through glutammate and kynurenine pathway. Aim of this study is to test the effectiveness of Aurastop© in the prophylaxis of migraine without aura.

**Materials and methods**: Sixty consecutive patients (F: n=37, M: n=23, mean age: 37.5±17.1) presenting with an ICHD-3 beta diagnosis of migraine without aura (MO) were enrolled in the survey and treated with Aurastop© twice a day for a period of 3 months. Diary cards were filled in during a 3-months period prior the beginning of the survey and during the 3-months duration of the study. A preventative treatment had been started previously and continued during the study in 5 cases (propranolol: n=2; amitriptyline: n=2; onabotulinumtoxin A: n=1).The reduction of MO attacks per month was assessed as the primary end-point; the reduction of headache days per month, the intensity of the pain and the patient’s satisfaction were considered as secondary end-points.

**Results**: A statistically significant reduction of both MO attacks and number of headache days per month was observed. Moreover, a sensible reduction of the intensity of the pain was reported. The secondary end-point regarding the satisfaction of the patients was achieved, as participants agreed when a new cycle of Aurastop© was proposed. No side effects were reported. The effectiveness appeared since the first month of intake and was maintained during the three months of therapy .

**Conclusion**: In this observational open study, Aurastop© appears to be effective and safe in the preventive treatment of MO.

**References**

Curto M, Lionetto L, Negro A, Capi M, Fazio F, Giamberardino MA, Simmaco M, Nicoletti F, Martelletti P. Altered kynurenine pathway metabolites in serum of chronic migraine patients. J Headache Pain. 2015; 17: 47.

Geppetti P, Bernabei S, De Cesaris F. CGRP receptors and TRP channels in migraine. J Headache Pain. 2015; 16(Suppl 1): A21.

Diener HC, Pfaffenrath V, Schnitker J, Friede M, Henneicke-von Zeppelin HH. Efficacy and safety of 6,25 mg t.i.d feverfew CO2-extract ( MIG-99) in migraine prevention – a randomized, double blind, multicenter, placebo controlled study. Cephalalgia. 2005; 25: 1031-41.

### P72 Association of Tanacethum Parthenium, 5-Hydroxytryptophan and Magnesium (Aurastop®) in the treatment of migraine aura: an observational study

#### F. Antonaci^1-3^, V.Rebecchi^4^, G. Sances^1^, P.Merlo^5^, A.Giorgetti^6^, F. Di Palma^7^, E. Matta^8^, C.Dall’ Occhio^9^, C.Tassorelli^1-3^, G.Dalla Volta^10^, On behalf of Società Italiana per lo Studio delle Cefalee (SISC–Lombardia) - Italia

##### ^1^Headache Science Centre, Istituto Neurologico Nazionale Mondino, Pavia; ^2^ UC Neurologia Speciale d'Urgenza, Istituto Neurologico Nazionale, Pavia; ^3^ Dipartimento di Scienze del Sistema Nervoso e del Comportamento Università di Pavia; ^4^ Centro Cefalee UOC Neurologia -Varese- ASST Settelaghi – Univ. Insubria; ^5^ U.O.Neurologia- Centro Cefalee, Humanitas Gavazzeni, Bergamo; ^6^ Centro Cefalee, Dipartimento di Neuroscienze H di Legnano ASST Ovest milanese; ^7^ Centro Cefalee UOC Neurologia della ASST Lariana-Ospedale S. Anna di Como; ^8^ Centro Cefalee UOC neurologia ASST Bergamo ovest; ^9^ UO Neurologia, ASST Pavia. Ospedale Civile, Voghera; ^10^ Centro Cefalee U.O Neurologia - Istituto Clinico Citta’ di Brescia - Brescia

###### **Correspondence:** G.Dalla Volta (dalla@numerica.it)

**INTRODUCTION:** A new phytotherapic combination of Tanacethum Parthenium (150 mg), 5 - Hydroxy tryptophan (20 mg) and Magnesium (185 mg) (Aurastop®) is now available for migraine patients. The three components may tackle the main mechanisms involved in the pathophysiology of migraine with aura: Cortical Spreading Depression, sensitization of trigeminal vascular system, central sensitization

**AIM:** The purpose of this open study was to evaluate the efficacy of the combined action of Tanacethum Parthenium, 5-Hydroxy tryptophan and magnesium in the reduction/disappearance of the aura phenomenon and reduction of its disability when taken at the aura onset.

**MATERIALS AND METHODS:** Study of a population of 200 patients aged between 18 and 65 years (mean age: 33.00 yrs ), 117 F and 83 M, suffering from migraine with aura referred to 8 Lombardia Headache Centers. Patients were either without preventive therapy or without changing the prophylaxis during the aura treatment. Inclusion criteria: suffering of aura with at least 20 minutes duration. Patients retrospectively filled a aura diary for the description of the aura features in the past 3 episodes. Then they treated 3 consecutive aura attacks with a tablet of Aurastop® at the onset of the aura. In case of headache they were instructed to take another 1 tablet at the headache onset. The effect of the Aurastop® was prospectively recorded using the aura diary in order to evaluate: duration of the aura, disability caused by the aura, number/efficacy of habitual symptomatic treatment

**RESULTS:** We recorded a total of 600 aura episodes from a total of 200 subjects: Aurastop® produced more than 50% reduction in duration in 180 patients ( 90%of the total ) and disability in 185 patients( 92,5%). Notably 35% of the patients did not use other symptomatic treatment due to the fact that headache intensity was less severe after Aurastop®.

**DISCUSSION AND CONCLUSIONS:** The combination of tanacethum partenum, Mg and 5-HTP, (Aurastop®), could be an effective symptomatic treatment for migraine aura. Randomised controlled trials are still required to confirm these results .

**References**

- Curto M, Lionetto L, Negro A, Capi M, Fazio F, Giamberardino MA, Simmaco M, Nicoletti F, Martelletti P. Altered kynurenine pathway metabolites in serum of chronic migraine patients. Journal of Headache pain 2015 Dec; 17(1):47.

- Geppetti et al.: CGRP receptors and TRP channels in migraine. The Journal of Headache and Pain 2015 16(Suppl 1):A21

- Pietrobon D, Moskowitz MA. Chaos and commotion in the wake of cortical spreading depression and spreading depolarizations. Nat Rev Neurosci. 2014;15:379–93.

### P73 Medical (respiratory, sleep, cardiovascular and gastrointestinal) comorbidities of migraine: Results from the Chronic Migraine Epidemiology and Outcomes (CaMEO) study

#### Richard B. Lipton^1^, Vincent T. Martin^2^, Michael L. Reed^3^, Kristina M. Fanning^3^, Aubrey Manack Adams^4^, Dawn C. Buse^5^

##### ^1^The Saul R. Korey Department of Neurology, Albert Einstein College of Medicine, Bronx, NY, USA; ^2^University of Cincinnati Headache and Facial Pain Center, University of Cincinnati College of Medicine, Cincinnati, OH, USA; ^3^Vedanta Research, Chapel Hill, NC, USA; ^4^Global Medical Affairs, Allergan plc, Irvine, CA, USA; ^5^Montefiore Headache Center, Bronx, NY, USA

###### **Correspondence:** Richard B. Lipton (Richard.Lipton@einstein.yu.edu)

**Background**

Many comorbidities associated with migraine have a higher relative frequency in chronic migraine (CM) than episodic migraine (EM). The objective of this study was to replicate and extend work on comorbid medical conditions in a systematically recruited sample of people with migraine.

**Materials and Methods**

Data from the prospective web-based baseline survey of the Chronic Migraine Epidemiology and Outcomes (CaMEO) Study were used to identify people, recruited from an online panel using quota sampling, with EM and CM based on criteria modified from the International Classification of Headache Disorders, third edition, beta version. Participants completed a Comorbidities/Endophenotypes module that assessed 64 symptoms and conditions. Respondents were asked (1) if they ever had a specific symptom (“Self-Reported [SR]”) and, if present, (2) if the SR symptom or condition had been confirmed/diagnosed by a “doctor” (“SR-physician diagnosis [SR-PD]”). Chi-square analysis was used to compare the proportion of people with each symptom or condition among respondents with EM vs. CM. This report presents data on the Respiratory, Sleep Disorder, Cardiovascular, and Gastrointestinal comorbidity categories.

**Results**

Available CaMEO respondents with migraine (16,763) were sent the Comorbidities/Endophenotype module and 12,810 (76.4%) provided valid responses: 11,699 with EM; 1,111 with CM. Compared with the EM group, the CM group had a similar mean age (EM, 41.3 years; CM, 41.9 years), was more likely to be female (EM, 74.2%; CM, 81.5%; *P*<0.001) and white (EM, 84.0%; CM, 88.7%; *P*<0.001), and had a mean higher body mass index (EM, 27.7 kg/m^2^; CM, 28.7 kg/m^2^; *P*<0.001). The relative frequencies were significantly higher for 29 (93.5%) of the 31 SR symptoms and SR-PD conditions assessed. Conditions or groups of conditions with relative frequencies >10% higher in CM than EM included allergies/hay fever/allergic rhinitis (EM, 37.4%; CM, 51.0%), sinusitis/sinus infection (EM, 47.3%; CM, 58.8%), insomnia (EM, 35.6%; CM, 50.2%), vertigo/dizziness/balance problems (EM, 17.8%; CM, 29.7%), and gastroesophageal reflux disease (EM, 14.3%; CM, 24.4%; Fig. 1).

**Conclusions**

Overall, significantly more respondents with CM vs. EM reported medical comorbidities. Mechanisms explaining this association might include manifestations of migraine, direct causality (e.g., CM causes the comorbidity), reverse causality (e.g., the condition increases the risk of CM), and shared genetic or environmental risk factors. Confounding or detection bias (i.e., “Berkson’s Bias”) could also contribute. Future analyses will address naturally occurring subgroups (taxa) defined by migraine phenotypes and comorbidities and assess the relationships of these groups to external validators such as treatment response and clinical course.

**Fig. 1 (abstract P73).**
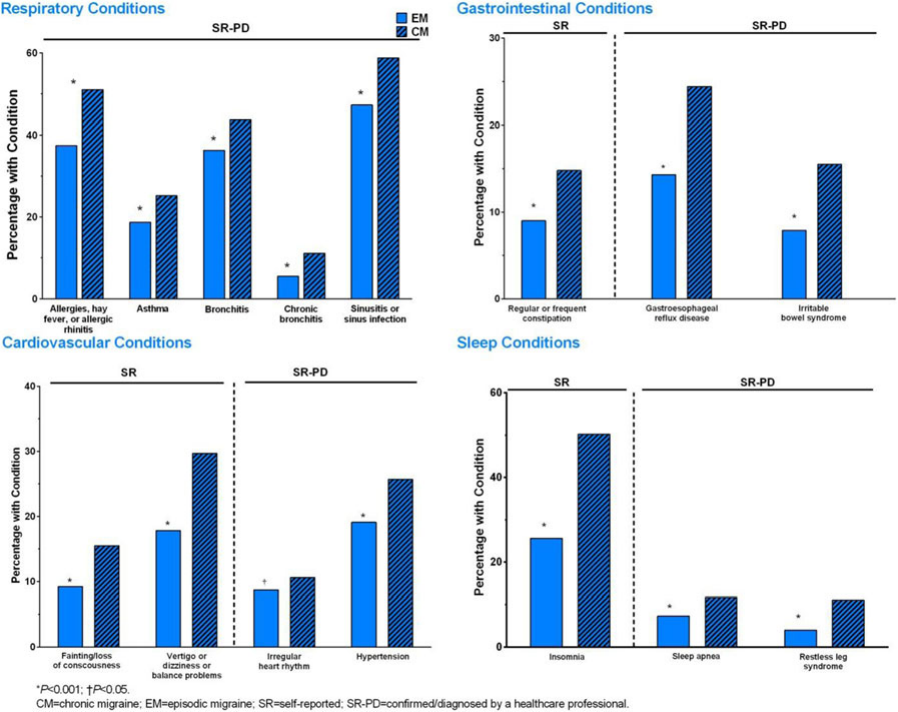
EM and CM respondents self-reporting (SR) an assessed symptom or a physician diagnosis (SR-PD) of a condition

**Acknowledgments**

Editorial support for development of this abstract was provided by Lee B. Hohaia, PharmD, at Complete Healthcare Communications, LLC (Chadds Ford, PA), a CHC Group company, and funded by Allergan, plc (Dublin, Ireland).

### P74 Development of a claims-based algorithm for use in patients with migraine to identify potentially undiagnosed chronic migraine patients

#### Jelena M. Pavlovic^1^, Justin S. Yu^2^, Stephen D. Silberstein^3^, Michael L. Reed^4^, Steve H. Kawahara^5^, Robert P. Cowan^6^, Firas Dabbous^7^, Karen L. Campbell^2^, Anand S. Shewale^2^, Riya Pulicharam^5^, Jonathan W. Kowalski^2^, Hema N. Viswanathan^2^, Richard B. Lipton^1^

##### ^1^Montefiore Medical Center, Bronx, NY, USA; ^2^Health Economics and Outcomes Research, Allergan plc, Irvine, CA, 92612, USA; ^3^Jefferson Medical Center, Philadelphia, PA, USA; ^4^Vedanta Research, Chapel Hill, NC, USA; ^5^DaVita Medical Group, El Segundo, CA, USA; ^6^Stanford University School of Medicine, Stanford, CA, USA; ^7^Independent Consultant, La Jolla, CA, USA

###### **Correspondence:** Justin S. Yu (Justin.Yu@Allergan.com)

**Background**

Published surveys have demonstrated that 75-80% of persons meeting the criteria for chronic migraine (CM) do not report having received an accurate diagnosis [1,2]. The primary objective of this study was to develop a claims-based algorithm for use in patients with migraine to identify potentially undiagnosed CM patients.

**Materials and Methods**

An observational study using claims data and survey data was conducted in a large United States medical group. Eligible patients had continuous enrollment and a migraine diagnosis (ICD-9/10 code of 346.xx/G43.xxx) in the 12-months prior to the screening date. Patients were excluded if they had a prior CM diagnosis (346.7x/G43.7xx) or migraine-related onabotulinumtoxinA claim. The Semi-structured Diagnostic Interview (SSDI) served as the gold standard for identifying CM and was administered to a convenience sample by trained clinicians. The SSDI included 31 questions related to headache frequency, symptoms, disability, medication use, and diagnosis.

A multivariate logistic regression model was used to identify potential predictors of CM based on claims data obtained in the 12 months prior to the screening date. Over 40 potential predictors for CM, identified from the literature and headache expert input, were evaluated for model inclusion. Variables that were significantly different in bi-variate analyses (p<0.05) between SSDI+ (CM) and SSDI- (non-CM) patients were included; each variable was categorized based on the data distribution and clinical relevance. The c-statistic, sensitivity, and specificity were calculated.

**Results**

Of the 108 patients who were included, 64 were SSDI+ and 44 were SSDI- for CM. Four statistically significant predictors of CM status were identified. Patients with ≥15 claims for acute treatment of migraine (including opioids) were nearly six times as likely to have CM as those with <15 claims (OR=5.87, 95% CI=1.34, 25.63); patients with ≥24 visits of any type (outpatient, inpatient, and emergency room visits) were nearly three times as likely to have CM as those with <24 visits (OR=2.80, 95% CI=1.08-7.26); females were 9 times as likely to have CM (OR=9.17, 95% CI=1.26-66.51); patients with claims for ≥2 unique migraine preventive classes were more than 4 times as likely to have CM as those without claims for preventive treatments (OR=4.40, 95% CI=1.19, 16.22). The c-statistic, sensitivity, and specificity for the model were 0.80, 78%, and 73%, respectively.

**Conclusions**

The claims-based algorithm for identification of undiagnosed CM patients demonstrated acceptable sensitivity and specificity, and can be used in health care settings to optimize the diagnosis and management of CM patients who may not be otherwise detected.

**Trial registration**

Not applicable

**Consent to publish**

Not applicable

**References**

1. Dodick D, Loder E, Manack Adams A, Buse D, Fanning K, Reed M, Lipton R. Assessing Barriers to Chronic Migraine Consultation, Diagnosis, and Treatment: Results from the Chronic Migraine Epidemiology and Outcomes (CaMEO) Study. Headache. 2016;56(5):821-834.

2. Bigal M, Serrano D, Reed M, Lipton R. Chronic migraine in the population. Neurology. 2008;71:559-566.

### P75 The relationship between pain, psychiatric, and endocrine/neurological comorbidities of migraine: Results from the Chronic Migraine Epidemiology and Outcomes (CaMEO) study

#### Richard B. Lipton^1^, Vincent T. Martin^2^, Michael L. Reed^3^, Kristina M. Fanning^3^, Aubrey Manack Adams^4^, Dawn C. Buse^1^

##### ^1^The Saul R. Korey Department of Neurology, Albert Einstein College of Medicine, Bronx, NY, USA; ^2^University of Cincinnati Headache and Facial Pain Center, University of Cincinnati College of Medicine, Cincinnati, OH, USA; ^3^Vedanta Research, Chapel Hill, NC, USA; ^4^Global Medical Affairs, Allergan plc, Irvine, CA, USA

###### **Correspondence:** Richard B. Lipton (Richard.Lipton@einstein.yu.edu)

**Background**

Migraine is comorbid with various conditions, many with a greater relative frequency in chronic migraine (CM) versus episodic migraine (EM). The objective of this study was to replicate and extend work on the comorbidity of pain, psychiatric, and endocrine/neurological symptoms and conditions in a systematically recruited sample of people with EM and CM.

**Materials and Methods**

Data from the prospective web-based baseline survey of the Chronic Migraine Epidemiology and Outcomes (CaMEO) Study were used to identify people, recruited from an online panel using quota sampling, with EM and CM based on criteria modified from the International Classification of Headache Disorders, third edition, beta version. Participants completed a Comorbidities/Endophenotypes module that assessed 64 symptoms (e.g., neck pain) and conditions (e.g., rheumatoid arthritis). Respondents were asked (1) if they ever had a specific symptom (“Self-Reported [SR]”) and, if present, (2) if the SR symptom or condition had been confirmed/diagnosed by a “doctor” (“SR-physician diagnosis [SR-PD]”). Chi-square analysis was used to compare the relative frequency of symptoms and conditions in respondents with EM versus CM. This report presents data on symptoms and conditions from the Pain, Psychiatric, and Endocrine/Neurological comorbidity categories.

**Results**

Available CaMEO respondents with migraine (16,763) were sent the Comorbidities/Endophenotype module and 12,810 (76.4%: EM, 11,699; CM, 1,111) provided valid responses. Compared with the EM group, the CM group had a similar mean age (EM, 41.3 years; CM, 41.9 years), was more likely to be female (EM, 74.2%; CM, 81.5%; *P*<0.001) and white (EM, 84.0%; CM, 88.7%; *P*<0.001), and had a mean higher body mass index (EM, 27.7 kg/m^2^; CM, 28.7 kg/m^2^; *P*<0.001). The relative frequencies were significantly higher for 24 (85.7%) of the 28 SR symptoms and SR-PD conditions assessed (Fig. 1). 5 of these conditions had relative frequencies >10% higher in CM than EM: chronic back pain (EM, 22.5%; CM, 37.6%), chronic pain (EM, 7.4%; CM, 22.2%), neck pain (EM, 38.1%; CM, 55.3%), anxiety (EM, 25.7%; CM, 42.2%), and depression (EM, 28.1%; CM, 45.6%).

**Conclusions**

Overall, significantly more respondents with CM versus EM reported having specific symptoms or conditions. Mechanisms explaining this association might include direct causality (e.g., CM causes the comorbidity), reverse causality (e.g., the condition increases CM risk), and shared genetic or environmental risk factors. Confounding, or detection bias (i.e., “Berkson’s Bias”) could also contribute.

**Fig. 1 (abstract P75).**
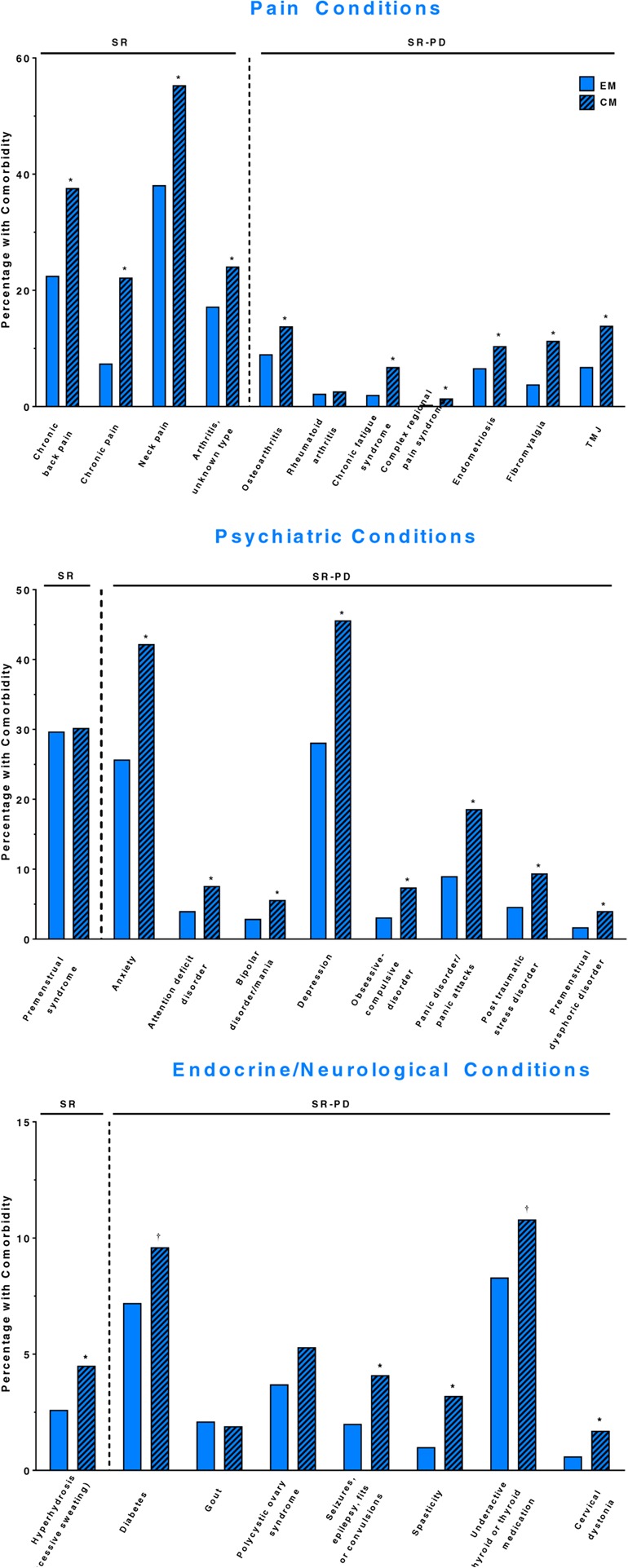
EM and CM respondents self-reporting (SR) an assessed symptom or a physician diagnosis (SR-PD) of a condition

**Acknowledgments**

Editorial support for development of this abstract was provided by Lee B. Hohaia, PharmD, and Dana Franznick, PharmD, at Complete Healthcare Communications, LLC (Chadds Ford, PA), a CHC Group company, and funded by Allergan, plc (Dublin, Ireland).

### P76 Are there sex differences among self-report pain features in patients with migraine? A pilot study

#### Lidiane L. Florencio^1^, Gabriela F. Carvalho^1^, Carina F. Pinheiro^1^, Marcela B. Mendes^1^, Mariana T. Benato^1^, Samuel S. Lodovichi^1^, Fabiola Dach^2^, Débora. Bevilaqua-Grossi^1^

##### ^1^ Department of Biomechanics, Medicine and Locomotor Apparatus Rehabilitation –Ribeirão Preto Medical School, University of São Paulo, Ribeirão Preto-SP, Brazil; ^2^ Department of Neurosciences and Behavioral Sciences – Ribeirão Preto Medical School, University of São Paulo, Ribeirão Preto-SP, Brazil

###### **Correspondence:** Lidiane L. Florencio

**Background:** Greater prevalence of migraine in women and the fact that women seek out medical advice more often than men have lead to a presumption that migraine is a female disorder. Possibly, this influenced to the actual under-diagnosis of migraine and to the suboptimal headache management in men. Therefore, clinicians and researchers are being encouraged to enhance the knowledge among possible sex differences in migraine clinical profile and comorbidities. Accordingly, the aim of the present study is to explore the self-related pain features, regarding migraine and neck pain, among patients with migraine of both sexes.

**Materials and Methods:** Patients with migraine, aging between 18 and 55 years old, were trialed in the university-based headache center from November 2015 to July 2017. Migraine diagnosis was provided by an expert neurologist according to the beta version of third edition of International Headache Disorders Classification. Exclusion criteria were other concomitant headache, fibromialgia, history of neck or head trauma. A structured questionnaire was applied to verify migraine and neck pain characteristics. The outcomes of interest were frequency of migraine, years living with pain conditions and its intensity. Severity of the disabilities related to migraine and to neck pain were respectively assessed by the Migraine Disability Assessment and the Neck Disability Index. Additionally, presence and severity of cutaneous allodynia was verified by 12 item Allodynia Symptom Checklist. Groups were compared using *t* test for independent samples and distribution among questionnaires classifications were tested by the Fisher’s exact test. All statistical analysis was performed by SPSS 20.0 adopting a level of significance of 0.05.

**Results:** Groups did not differ regarding migraine features and migraine related disability (p>0.05) (Table 1). Neck pain was reported by 83% of women group (n=10) and by 42% of men group (n=5) (p=0.09). Women presented more intense neck pain (p=0.045) and greater neck related disability (p=0.002) than men (Table 1). They also reported more symptoms of allodynia (Table 1; p=0.005). The prevalence of cutaneous allodynia was 92% (n=11) in the women’s group and 77% (n=8) in the men’s group; severe and mild allodynia were the most frequent classification respectively (Fig. 1). No difference could be evidenced among severity distribution of cutaneous allodynia, migraine- and neck-related disability (Fig. 1).

**Conclusion:** For instance, findings suggest that female sex may be associated with a higher frequency of cutaneous allodynia symptoms and worse severity of neck pain and neck related disability in migraineurs.

**Fig. 1 (abstract P76).**
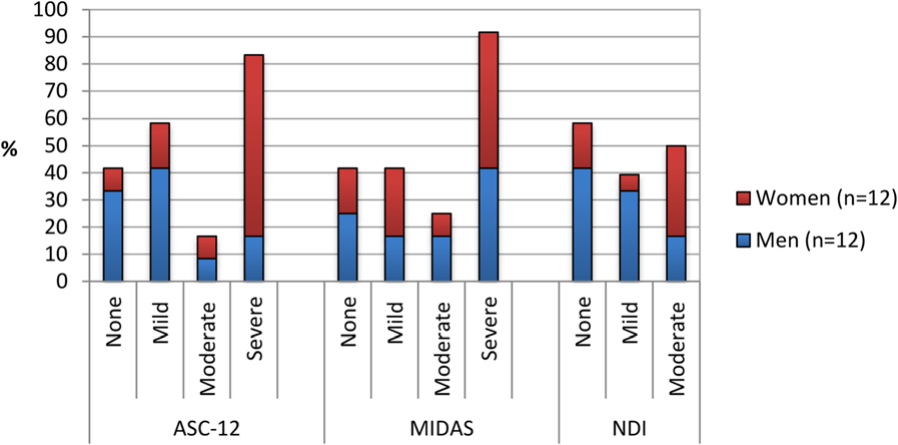
Prevalence of the severity classifications of cutaneous allodynia measured by the 12 item Allodynia Symptom Checklist (ASC-12); migraine related disability assessed using the Migraine Disability Assessment (MIDAS) and; neck related disability assessed by the Neck Disability Index (NDI)

**Table 1 (abstract P76).** Sample characteristics considering self-report pain features and disability related to migraine and neck pain

	Men with migraine (n=12)	Women with migraine (n=12)
Age (years)	34.2 (8.1)	34.3 (10.6)
ASC-12	4.2 (3.3)^*^	9.1(4.5)
Migraine		
Frequency (days/moth)	16.3 (9.8)	15.4 (8.5)
Intensity (0-10)	8.3 (1.4)	7.9 (2.3)
Onset (years)	15.2 (13.0)	19.6 (18.7)
MIDAS	19.3 (14.1)	34.1 (32.5)
Neck pain		
Intensity (0-10)	2.7 (3.7)^*^	5.7 (3.0)
Onset (years)	2.8 (6.0)	6.6 (5.8)
NDI	6.7 (6.5)^*^	12.6 (5.6)

Abbreviations: *ASC-12* 12 item Allodynia Symptom Checklist, *MIDAS* Migraine Disability Assessment, *NDI* Neck Disability Index

^*^P<0.05

### P77 Analysis of endurance capacity of cervical extensors and flexors muscles in individuals with migraine and controls: a pilot study

#### Iuri V De Oliveira^1^, Samuel S Lodovichi^1^, Lidiane L Florencio^1^, Carina Pinheiro^1^, Fabiola Dach^1^, Debora Bevilaqua Grossi^1^

##### ^1^ Department of Biomechanics, Medicine and Rehabilitation of Locomotor Apparatus, Ribeirão Preto Medical School, University of São Paulo, Ribeirão Preto, São Paulo, 14049-900, Brazil

###### **Correspondence:** Iuri V De Oliveira (iurivaloti@gmail.com)

**BACKGROUND**

Patients with migraine often present neck pain. Additionally, migraine has been associated to an altered neck muscle performance characterized by increased activity of the superficial cervical extensor muscles and decreased cervical extensor strength. [1,2] Despite the evidences of the impaired endurance capacity in neck pain sufferers [3,4], neck muscles endurance has never been reported in subjects with migraine. Accordingly, our aim was to evaluate the endurance capacity of neck muscles in individuals with migraine and controls.

**MATERIALS AND METHODS**

Fifteen subjects with migraine were screened from a headache tertiary clinic and fifteen headache-free controls from general population. The participants responded to a previously structured questionnaire containing general information, such as sex, age, weight, and height (Table 1). Endurance test of flexors and extensors was held by a blind evaluator. The flexor endurance was performed with the patient lying in the supine position and positioning the head and neck in slight flexion. The examiner placed the hand under the head, and the test was terminated if the head touched the hand of the examiner or if the subject cannot sustain the position. The extensor endurance test was performed with the patient lying prone. A cervical range-of-motion device was placed on the head to maintain the head's alignment with the horizontal plane. A Velcro band was fixed at the head of the patient and a 2-kg weight was attached to the Velcro band, placed around the participants’ head. The test was terminated when the subject was no longer able to hold the position, or if the head position changed more than 5° from the starting position[5]. Holding time (s) of both positions were recorded with a chronometer. Approval for the study was obtained from the Human Ethics Committee of the University of Sao Paulo (6861/2016), and each subject gave informed consent before testing. Groups were compared using Independent-samples t tests adopting a level of significance of 0,05).

**RESULTS**

Migraine group presented lower holding time in the flexor endurance test when compared to the control group. At the extensor endurance test there was no significant difference (Table 2).

**CONCLUSIONS**

Preliminary findings suggest that subjects with migraine present worse performance of neck flexor endurance characterized by a decreased holding time when compared to controls.

**Table 1 (abstract P77).** Sample characterization

	Control (*n*=15)	Migraine (*n*=15)
Age (y)	25,06(4,07)	29,93 (7,17)
Weight(kg)	64,38(11,35)	68,4 (11,28)
Height (cm)	164(8)	165 (7)

Data are expressed by mean (standard deviation)

**Table 2 (abstract P77).** Holding time of endurance tests

Endurance	Control (*n*=15)	Migraine (*n*=15))	*p*
*Flexion (s)*	81,33(51,06)	46,73(31,34)	0,03
*Extension (s)*	351(138,42)	242,20(169,24)	0,07

Data are expressed by mean (standard deviation)

**References**

1. Florencio LL, de Oliveira AS, Carvalho GF, et al. Cervical Muscle Strength and Muscle Coactivation During Isometric Contractions in Patients With Migraine: A Cross-Sectional Study. Headache. 2015; 55(10): 1312-1322

2. Florencio LL, de Oliveira AS, Lemos TW, Carvalho GF, Dach F, Bigal ME, Falla D, Fernández-de-las-Peñas C, Grossi DB. Patients With Chronic, but not Episodic, Migraine Display Altered Activity of Their Neck Extensor Muscles. J Electromyogr Kinesiol. 2016; (30): 66–72

3. Falla D, Lindstrom R, Rechter L, Boudreau S, Petzke F. Effectiveness of an 8-week exercise programme on pain and specificity of neck muscle activity in patients with chronic neck pain: A randomized controlled study. Eur J Pain, 2013. (17): 1517-1528

4. Jull GA, Falla D, Vicenzino B, Hodges PW. The effect of therapeutic exercise on activation of the deep cervical flexor muscles in people with chronic neck pain. Man Ther 2009;14(6):696-7

5. Edmondston SJ, Wallumrød ME, MacLéid F, Kvamme LS, Joebges S, Brabham GC. Reliability of isometric muscle endurance tests in subjects with postural neck pain. J Manipulative Physiol Ther 2008; (31):348-354

### P78 Light-induced discomfort changes semi-static posture control of migraine patients

#### Carina F Pinheiro^1^, Renato Moraes^2^, Lais Sestari^1^, Gabriela F Carvalho^1^, Anamaria S Oliveira^1^, Fabiola Dach^1^, Débora B Grossi^1^

##### ^1^Department of Biomechanics, Medicine and Rehabilitation of Locomotor Apparatus, Ribeirão Preto Medical School, University of São Paulo, Ribeirão Preto, São Paulo, 14049-900, Brazil; ^2^School of Physical Education and Sports of Ribeirão Preto, University of São Paulo, Ribeirão Preto, São Paulo, 14049-900, Brazil

###### **Correspondence:** Carina F Pinheiro (carinafp@hotmail.com)

**Background:** Patients with migraine have showed balance deficits with increased postural oscillation during quiet stance, alterations in gait mobility, reaction time, and movement velocity in comparison to control subjects [1,2]. Light sensibility is a migraine feature that happens mostly during headache attacks, but also can be present in lower intensity during the interictal period, contributing to migraine disability [3]. However, it has not been established if the exposition to different light intensities modifies the semi-static posture control of migraine patients in the interictal period. Our purpose was to investigate bipedal postural control of migraine women under different light intensities.

**Materials and Methods:** Fourteen woman with migraine [4] were selected from a headache tertiary clinic and from general population. All participants aged between 18 and 55 years-old (mean 29.3, 95% CI 23.6 to 35.0) with body mass index lower than 30 kg/cm^2^ (mean 23.6, 95%CI 23.2 to 24.0). The exclusion criteria were: volunteers affected by vascular and/or neurological systemic conditions, presence of other concomitant headache, movement coordination impairment or any musculoskeletal disability that could interfere in the test performance. Participants were exposed to gradual light increase from 300 to a maximum of 2000 lux, and were asked about the visual discomfort intensity (VAS) induced by the light. For balance assessment, participants stood upright in a bipedal position on a force platform for 30 seconds in the following light conditions: 1) visual threshold, in which light induce the minimal visual discomfort; 2) visual discomfort, in which light induce moderate-to-intense visual discomfort; and 3) control, with only ceiling light room (270 lux). Participants performed three trials for each condition. Light intensity was measured by a digital luximeter positioned at participant’s eye level. All participants were assessed during the interictal period.

A repeated measures ANOVA with LSD post-hoc (p<0.05) was used to compare the center of pressure (CoP) ellipse area and the CoP speed among the three light conditions.

**Results:** We found a median visual threshold equal to 450 lux (IQR 400 to 600) and a median visual discomfort of 2000 lux (IQR 1900 to 2000). Migraineurs showed larger CoP area at the visual discomfort condition compared to the visual threshold and control conditions (p<0.05). CoP speed at the visual discomfort condition was higher than the other conditions tested, but with no statistical difference (Table 1).

**Conclusion:** Visual discomfort induced by light intensity manipulation increased the CoP area of migraineurs patients in bipedal quiet stance.

**Table 1 (abstract P78).** CoP area and CoP speed at three light conditions - mean and 95% confidence interval

	Visual threshold	Visual discomfort	Control
CoP area (cm^2^)	3.54 (0.87 to 6.20)^a^	5.03 (1.82 to 8.25)	1.52 (0.99 to 2.06)^a^
CoP speed (cm/sec^2^)	1.74 (1.35 to 2.14)	1.78 (1.45 to 2.10)	1.49 (1.39 to 1.59)

^a^different from the visual discomfort condition (p<0.05)

**References**

1. Carvalho GF, Chaves TC, Dach F, Pinheiro CF, Gonçalves MC, Florencio LL, et al. Influence of migraine and of migraine aura on balance and mobility - A controlled study. Headache. 2013;53(7):1116–22.

2. Carvalho GF, Bonato P, Florencio LL, Pinheiro CF, Dach F, Bigal ME, et al. Balance Impairments in Different Subgroups of Patients With Migraine. Headache J Head Face Pain. 2017; Mar;57(3):363-374

3. Harriott AM, Schwedt TJ. Migraine is Associated With Altered Processing of Sensory Stimuli. Curr Pain Headache Rep. 2014;18(11).

4. Olesen J. The International Classification of Headache Disorders, 3rd edition. Cephalagia. 2013;33(9):629–808.

### P79 Poor sleep quality in probable migraine: a population-based study

#### Tae-Jin Song^1^, Soo-Jin Cho^2^, Won-Joo Kim^3^, Kwang Ik Yang^4^, Chang-Ho Yun^5^, Min Kyung Chu^6^

##### ^1^Department of Neurology, Ewha Womans University School of Medicine, Seoul, Korea; ^2^Department of Neurology, Dongtan Sacred Heart Hospital, Hallym University College of Medicine, Hwaseong, Korea; ^3^Department of Neurology, Gangnam Severance Hospital, Yonsei University, College of Medicine, Seoul, Korea; ^4^Department of Neurology, Soonchunhyang University College of Medicine, Cheonan Hospital, Cheonan, Korea; ^5^Clinical Neuroscience Center, Department of Neurology, Seoul National University Bundang Hospital, Seongnam, Korea; ^6^Department of Neurology, Kangnam Sacred Heart Hospital, Hallym University College of Medicine, Seoul, Korea

###### **Correspondence:** Min Kyung Chu (chumk@hallym.ac.kr)

**Background:** Although sleep problems are common among headache sufferers,[1] there is little knowledge of the association of poor sleep quality with probable migraine (PM) in general population. In the present study, we aimed to 1) describe the prevalence of poor sleep quality in PM in a general population-based sample; 2) compare the prevalence of poor sleep quality and components of Pittsburgh Sleep Quality Index (PSQI) score among migraineurs, participants with PM, and non-headache individuals and 3) assesse the clinical impact of poor sleep quality among participants with PM.

**Materials and methods:** We used the data of Korean Headache-Sleep Study (KHSS) in the present study.[2] The KHSS is nation-wide population-based survey regarding headache and sleep for adults aged 19 – 69 years. The KHSS used 2-stage clustered random sampling method which was proportional to population distribution in all Korean territories. Diagnoses of migraine and PM were based on criteria A to D for migraine without aura (code 1.1) in the International Classification of Headache Disorders-3 beta.[3] We investigated the components of PSQI and defined poor sleep quality as PSQI score > 5.[4]

**Results:** In a representative sample of 2,695 individuals, 143 (5.3%), 379 (14.1%) and 715 (26.5 %) had migraine, PM, and poor sleep quality, respectively (Table 1). The PM participants with poor sleep quality were noted in 134 (35.4%). The prevalence of poor sleep quality was lower among individuals with PM compared to those with migraine (35.4% vs. 47.6%, p = 0.011) but higher than those with non-headache (17.9%, p < 0.001). Among components of PSQI, sleep latency (p < 0.001), sleep duration (p < 0.001), sleep disturbance (p < 0.001), daytime functioning (p < 0.001), and use of sleeping medication (p < 0.001) scores were higher in participants with PM compared to non-headache participants (Table 2). The PM participants with poor sleep quality had more frequent headache (median [interquartile range]) (2.0 [0.3 – 4.0/month] vs. 1.0 [0.3 – 2.0]/month, p = 0.001), visual analogue scale score for headache intensity (6.0 [4.0 – 7.0] vs. 5.0 [3.5 – 6.0], p = 0.003), and headache impact test-6 score (50.0 [44.0 – 58.0] vs. 44.0 [40.0 – 50.0], p < 0.001) than those who without poor sleep quality.

**Conclusions:** Approximately 35% of participants with PM had poor sleep quality. Poor sleep quality was associated with increased headache frequency, intensity and impact of headache among PM in general population setting.

**Fig. 1 (abstract P80).**
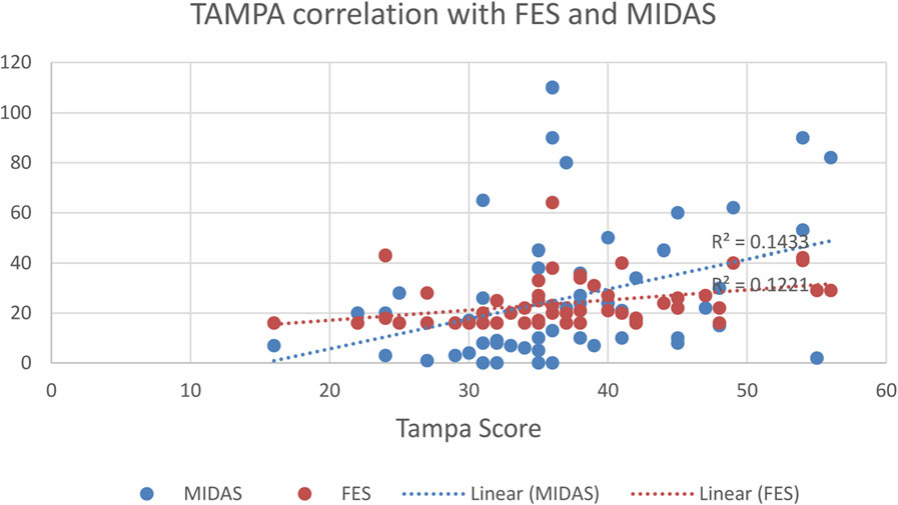
Pearson’s correlation between TAMPA with FES and with MIDAS among migraineurs, p<0.05

**Table 1 (abstract P79).** Sociodemographic characteristics of survey participants; the total Korean population; and cases identified as migraine, probable migraine and poor sleep quality

	Survey participants N (%)	Total population N (%)	p	Migraine N, % (95% CI)	PM N, % (95% CI)	Poor sleep quality N, % (95% CI) (PSQI> 5)
Sex						
Men	1,345 (49.3)	17,584,365 (50.6)	0.854^a^	36, 2.7 (1.8-3.5)	136, 10.1 (8.5-11.8)	334, 24.8 (22.5-27.1)
Women	1,350 (50.7)	17,198,350 (49.4)		107, 7.9 (6.5-9.4)	243, 17.9 (15.8-19.9)	381, 28.2 (25.8-30.6)
Age						
19–29	542 (20.5)	7,717,947 (22.2)	0.917^a^	25, 4.5 (2.7-6.2)	69, 12.6 (9.8-15.4)	153, 28.3 (24.4-32.0)
30–39	604 (21.9)	8,349,487 (24.0)		42, 7.0 (4.9-9.1)	102, 16.8 (13.7-19.8)	136, 22.5 (19.2-25.9)
40–49	611 (23.1)	8,613,110 (24.8)		39, 6.5 (4.5-8.4)	102, 16.8 (13.9-19.8)	167, 27.3 (23.8-30.9)
50–59	529 (18.9)	6,167,505 (17.7)		22, 4.1 (2.4-5.9)	62, 11.6 (8.8-14.4)	160, 30.2 (26.3-34.2)
60–69	409 (15.6)	3,934,666 (11.3)		15, 3.9 (2.0-5.7)	44, 11.2 (8.1-14.2)	99, 24.2 (20.0-28.4)
Size of residential area						
Large city	1,248 (46.3)	16,776,771 (48.2)	0.921^a^	76, 6.1 (4.8-7.5)	180, 14.4 (12.4-16.3)	338, 27.1 (24.6-29.6)
Medium-to-small city	1186 (44.0)	15,164,345 (43.6)		48, 4.0 (2.9-5.2)	174, 14.7 (12.7-16.7)	303, 25.5 (23.1-28.0)
Rural area	261 (9.7)	2,841,599 (8.2)		19, 7.4 (4.2-10.6)	25, 9.7 (6.1-13.3)	74, 28.4 (22.8-33.9)
Education level						
Middle school or less	393 (14.9)	6,608,716 (19.0)	0.752^a^	22, 5.5 (4.2-7.7)	44, 11.5 (8.4-14.7)	110, 28.0 (23.5-32.4)
High school	1,208 (44.5)	15,234,829 (43.8)		60, 5.0 (3.8-6.3)	178, 14.7 (12.7-16.7)	317, 26.2 (23.8-28.7)
College or more	1,068 (39.6)	12,939,170 (37.2)		60, 5.6 (4.3-7.0)	155, 14.4 (12.3-16.5)	281, 26.3 (23.7-29.0)
Not responded	26 (9.6)			1, 3.8 (0.0-11.8)	2, 7.7 (0.0-18.7)	7, 26.9 (8.7-45.2)
Total	2695 (100.0)	34,782,715 (100.0)		143, 5.3 (4.5-6.2)	379, 14.1 (12.7-15.4)	715, 26.5 (24.9-28.2)

*N* number, *CI* 95% confidence interval, *PM* probable migraine, *PSQI* Pittsburgh Sleep Quality Index, Variables are presented as number (%) or number, % (95% confidence interval)

^a^Compared gender, age group, size of residential area, and education level between the sample of the present study and the total population of Korea

**Table 2 (abstract P79).** Total and subcomponents score of PSQI of individual with non-headache, PM and migraine

	Non-headache individuals N = 1,422 (52.8%)	PM N = 379 (14.1%)	Migraine individuals N = 143 (5.3%)	p value
Subjective sleep quality	2.0 (2.0-2.0)	2 (2.0-2.0)	2.0 (2.0-2.0)	0.879
Sleep latency	1.0 (0.0-1.0)	1.0 (0.0-2.0)^a^	1.0 (0.0-2.0)^a^	<0.001
Sleep duration	0.0 (0.0-1.0)	0.0 (0.0-1.0)^a^	0.0 (0.0-1.0)	<0.001
Habitual sleep efficacy	0.0 (0.0-0.0)	0.0 (0.0-0.0)	0.0 (0.0-0.0)	0.244
Sleep disturbance	1.0 (0.0-1.0)	1.0 (1.0-1.0)^ab^	1.0 (1.0-2.0)^a^	<0.001
Use of sleeping medication	0.0 (0.0-0.0)	0.00 (0.0-0.0)^a^	1.0 (0.0-1.0)^a^	<0.001
Daytime functioning	0.0 (0.0-1.0)	1.0 (0.0-1.0)^a^	1.0 (0.0-1.0)^a^	<0.001
Total	4.0 (3.0-5.0)	5.0 (4.0-6.0)^a^	5.0 (4.0-7.0)^a^	<0.001

*PSQI* Pittsburgh Sleep Quality Index, *PM* probable migraine, Variables are presented as median (interquartile range)

^a^Significantly different compared to individuals with non-headache

^b^Significantly different compared to individuals with migraine

**References**

1. Rains JC, Poceta JS, Penzien DB. Sleep and headaches. Curr Neurol Neurosci Rep. 2008;8(2):167-75.

2. Song TJ, Cho SJ, Kim WJ, Yang KI, Yun CH, Chu MK. Anxiety and depression in probable migraine: A population-based study. Cephalalgia. 2016 Jun 1. pii: 0333102416653235

3. Headache Classification Committee of the International Headache S. The International Classification of Headache Disorders, 3rd edition (beta version). Cephalalgia. 2013;33(9):629-808.

4. Buysse DJ, Reynolds CF, 3rd, Monk TH, Berman SR, Kupfer DJ. The Pittsburgh Sleep Quality Index: a new instrument for psychiatric practice and research. Psychiatry Res. 1989;28(2):193-213.

### P80 Correlation and differences between kinesiophobia, fear of falling and disability among women and men with migraine

#### Gabriela Ferreira Carvalho^1^; Lidiane L Florêncio^1^, Carina Ferreira Pinheiro^1^, Marcela B. Mendes^1^, Mariana T. Benato^1^, Samuel S. Lodovichi^1^, Fabiola Dach^2^, Débora Bevilaqua-Grossi^1^

##### ^1^Department of Biomechanics, Medicine and Locomotor Apparatus Rehabilitation – Ribeirão Preto Medical School, University of São Paulo, Ribeirão Preto-SP, Brazil. ^2^Department of Neurosciences and Behavioral Sciences – Ribeirão Preto Medical School, University of São Paulo, Ribeirão Preto-SP, Brazil

###### **Correspondence:** Gabriela Ferreira Carvalho

**Background:** Migraine could be associated to balance changes and self-report of falls due malfunctioning of the cerebellum and inner ear. Further knowledge regarding features related to balance such as kinesiophobia, fear of falling, dizziness handicap and migraine disability are important for proper management among migraineurs.

**Objective:** To investigate the presence of kinesiophobia, fear of falling, dizziness handicap and migraine disability and its correlation among women and men with migraine.

**Methods**: Patients diagnosed with migraine according with the ICHD-III were screened from a tertiary headache center and sorted in two groups: women (n=38) and men (n=17). Subjects with systemic diseases, vestibular disease history, presence of other headache diagnosis or any musculoskeletal impairment were excluded. A structured interview was conducted in order to obtain information regarding demographics including age, BMI, presence of dizziness, physical activity level and headache features. Subsequently it was applied the following questionnaires: Tampa Kinesophobia Scale, Falls Efficacy Scale (FES), Dizziness Handicap Inventory (DHI) and Migraine Disability Assessment (MIDAS). Demographics were contrasted using a one-tailed t-test, questionnaires scores were compared with a Mann-Whitney for independent samples and its classifications among groups with Fisher’s Exact Test. Pearson’s correlation between the questionnaires was calculated based on Cohen classification (0.1-0.3: weak, 0.3-0.5: moderate and >0.5 large correlation). The analysis was performed in the SPSS 21.0 software with a fixed significant level at 5%.

**Results:** Women and men group of migraineurs did differ in the following outcomes: age (p=0.12), BMI (p=0.13), migraine frequency, intensity and headache onset (p>0.09). However, the level of physical activity was different between women and men (p=0.04). No differences according with the sex was found in the FES, TAMPA and MIDAS questionnaires, and scores for most of them were considered moderated-to-high, even though presence of kinesiophobia was identified just in man (DHI=0.08, FES=0.17, MIDAS=0.06, TAMPA=0.08), Presence and handicap due dizziness were higher in women than men (p<0.05) (Table 1). Moderate significant correlations were found between TAMPA and FES (r=34), and for TAMPA and MIDAS (r=41) (Fig. 1).

**Conclusion:** Few studies address men with migraine and these results highlight the absence of significant differences in headache disability, kinesiophobia, fear of falling, according with sex, with found scores moderate-to-high for all migraineurs. Dizziness handicap Kinesiophobia is correlated to headache disability and fear of falling in migraineurs. Those aspects are related to migraineurs’ quality of life and might be addressed during clinical assessment.

**Fig. 1 (abstract P96).**
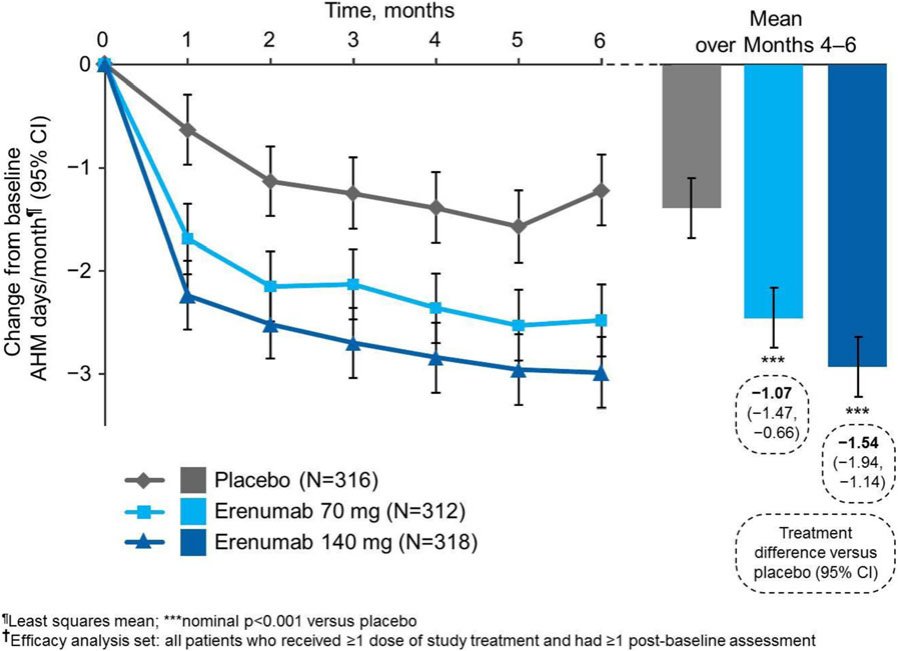
Change from baseline in AHM days/month^†^

**Table 1 (abstract P80).** Average and 95%CI of sample characteristics and questionnaires scores among men and women with migraine.

	Men with migraine (n=17)	Women with migraine (n=38)
Age (years)	33.8 (7.3)	34.2 (11.3)
BMI	27.2 (4.7)	27.2 (5.8)
Migraine		
Frequency (days/month)	17.0 (9.0)	17.3 (10.7)
Intensity (0-10)	8.5 (1.4)	7.9 (2.2)
Onset (years)	14.0 (12.6)	14.1 (10.0)
Level of physical activity^*^		
Inactive	11%	12%
Minimally active	27%	47%
Active	51%	29%
Hepa active	11%	12%
MIDAS	17.3 (13.7)^*^	32.0 (37.7)
FES	22.2 (11.9)	24.8 (8.7)
Tampa	39.2 (8.1)	35.3 (8.7)
Dizziness		
Self-report	24%^*^	63%
DHI	34.5 (16.1)^*^	47.4 (20.5)

Abbreviations: *BMI* Body Mass Index, *MIDAS* Migraine Disability Assessment, *FES* Falls Efficacy Scale, *DHI* Dizziness Handicap Inventory

^*^p<0.05

### P81 Handl syndrome in pediatric age

#### Irene Salfa, Laura Papetti, Barbara Battan, Romina Moavero, Massimiliano Valeriani

##### Pediatric Headache Center, Children Hospital Bambino Gesù of Rome, Italy

**OBJECTIVES**

The syndrome of transient headache and neurologic deficits with cerebrospinal fluid lymphocytosis (HaNDL) is a rare syndrome of unclear pathogenesis characterized by one or more episodes of severe headache, transient neurologic deficits and lymphocytic pleocytosis in the cerebrospinal fluid, seldom reported in paediatric age. In most cases it is a benign and self limited disorder, although it may mimic various serious, including life-threatening, diseases, such as stroke and meningoencephalitis, which is why vigorous tests should be sought before this diagnosis of exclusion can be reached.

**METHODS/RESULTS**

We report three cases of HaNDL occurred in 2 boys (14 years and 10 years old) and in a 17 years old girl. Each patient presented with headache, altered conscious state and papilledema associated with different neurological symptoms such as dysarthria, hemiplegia, pernicious vomiting, ideomotor slowing and psychomotorr agitation. None of them had fever and there was no evidence of meningeal irritation.

They received Ceftriaxone, Aciclovir, and Dexamethasone for possible encephalitis and/ or autoimmune disorders. Clinical manifestations were compatible with a variety of disorders including structural brain lesions, meningitis, seizures, autoimmune, vasculitic and paraneoplastic disorders. We performed neuroimaging examinations (CT scan and MRI of the brain), EEG and serum/CSF studies for infectious, autoimmune and vasculitic diseases. All of these aetiologies were ruled out. In one case, a complete tox screen was added and it resulted negative. The laboratory finding common to all three cases was a clear CSF lymphocytic pleocytosis and an elevated opening pressure during lumbar puncture. The intracranial hypertension treated in all three cases with acetazolamide per os with complete remission. In one case, it was necessary the admission in the intensive care unit because of the worsening of psychomotorr agitation of the patient, requiring sedation and endotracheal intubation. All three patients recovered without any neurological sequelae during the follow up.

**CONCLUSION**

The possibility of HANDL should be considered in patients presenting with unusual patterns of headache and transient neurological symptoms. It is most commonly diagnosed in the third or fourth decades of life and is rare in the paediatric population. However, awareness of HANDL existence also in children and adolescents can avoid unnecessary and potentially harmful investigations and therapies.

### P82 Usage of Quetiapine in Profilactic Treatment of Chronic Migraine

#### Tetiana Maikova, Sergiy Lukashov, Ekaterina Dzevitskaya, Mariya Kuts

##### Headache Center, Kyiv, Ukraine, 04070

###### **Correspondence:** Tetiana Maikova (maykova@headache.com.ua)

**Background.** The treatment and prevention of migraine attacks has for long yielded insufficient results. A usual level of result achieved (using standard treatment methods) (i.e. 50% reduction in frequency of attacks) [1] does not produce satisfactory quality of life for the patients. Neither does it reduce the problem of resistant attacks.

We conducted the current study with the goal of increasing efficacy of the prevention of migraine attacks using quetiapine as an additional (supplementary) medicine in patients suffering from Chronic Migraine [2] and already taking anticonvulsants.

**Materials and methods.** 118 male and female patients from 18 to 50 y.o., suffering from Chronic Migraine were included in this study. Pregnant and nursing mothers and any patient suffering from somatic illnesses were excluded from the sample.

Patients were prescribed topiromat (up to 300 mg/per day) for a 6-month period, but were transferred to sodium valproat (up to 30 mg/kg of weight) in cases of non-response. In cases of response to treatment quetiapine was added as a supplementary (secondary) medicine after 6-month treatment with sodium valproat. A single dose of quetiapine was prescribed at night. The dosage was titrated from 25 mg to 400 mg for a period of 1-1.5 months until a therapeutic effect was attained . The treatment using topiromat+quetiapine or sodium valproat+ quetiapin lasted for a period of 6 months.

**Results.** We observed in 68.5% of patients complete absence of attacks as result of supplementing quetiapine to the achieved existing level of their individual therapeutic dosage. In 32.5% of cases frequency of attacks was no more than 2 per month. In 1%, frequency of attacks were remained up to 4 per month. All observed attacks were relieved rapidly by NSID or triptans.

**Conclusions.** Quetiapine shows a high efficacy as a supplementary (secondary) medicine when insufficient results were achieved over a 6 months period when prescribing topiromat or sodium valproat respectively.

The following side-effects were observed when dosage of quetiapine was rapidly increased (up to 100mg per 1-1.5 months): daytime drowsiness for the first 10 days of medication in doses up to 100 mg/per day. This side-effect disappeared when the pain subsided. In 3 patients, congestion of nasal passages was observed before sleep, which was also reduced with increased dosage.

In addition to the pain relief achieved, the following concomitant disorders were reduced: anxiety and asthenia, insomnia, hiperalgomenorroea. The total effect was a general enhancement of the patients’ quality of life.

We suggest that increased level of transmissions of serotonin may be underlying reason basis of high efficacy of quetiapine in resistant and chronic forms of migraine (with and without aura).

**Referenses**

1. Martelletti P., Katsarava Z., Lampe C., et al (2014) Refractory chronic migraine: a consensus statement of clinical definition from the European Headache Federation. J. Headache Pain 28: 15-47 (PubMed).

2. Headache Classification Committe of the Internationa lHeadache Society (2013). The International Classification of Headache Disorders, 3^rd^ edition. Cephalalgia 33: 629-808 (PubMed).

### P83 Ice cream headache and rock and roll

#### Federico Mainardi^1^, Giorgio Zanchin^2^, Ferdinando Maggioni^2^

##### ^1^Headache Centre, Neurological Division, SS Giovanni e Paolo Hospital, Venice, Italy; ^2^Headache Centre, Department of Neurosciences, Padua University, Padua, Italy

###### **Correspondence:** Federico Mainardi (fmainardi@iol.it)

**Background**: *Headache attributed to ingestion or inhalation of a cold stimulus* is currently codified in Chapter 4 of the International Classification of Headache Disorders 3 beta (ICHD 3 beta) [1] at the first sub-digit of 4.5 *Cold stimulus headache* (CSH). Although the exact prevalence of CSH in the general population has not been assessed, it appears to be not so uncommon: a 15% lifetime prevalence was found in the general population aged 25-64 [2], raising to 41% in 13-15 years old adolescent [3].

**Case report**: The renowned journal Billboard published the comment of a live performance of the American rocker Bruce Springsteen, held at the Metlife Stadium (East Rutherford, New Jersey) on August 30, 2016. During the show, the rocker swallowed a cold beverage and, immediately after, turned to the public, saying that he had got an ice cream headache: “*In a comical moment, Springsteen put the song on pause for a moment when he stopped to enjoy a cold drink from the audience. “I’ll be with you in a minute,” he said, later taking another swig and chugging it like a teenager. “I got an ice cream headache”*” [4]. From this brief comment, we can infer that criteria B and C for 4.5.1 *Headache attributed to ingestion or inhalation of a cold stimulus* are satisfied, but not criterion A. Therefore, a diagnosis of 4.5.3.2 *Probable headache attributed to ingestion or inhalation of a cold stimulus* could be proposed.

**Discussion**: CSH, as well as *External pressure headache* (EPH), previously considered as secondary headaches and codified in chapter 13, have been moved in Chapter 4 of the current classification, within Chapter 4. *Other primary headaches*. The reason of this modification is justified by the fact that *“they are brought on by physiological (non-damaging) stimuli*” [1]. However, both of these forms of headache are strictly related to the application of recognizable factors, fulfilling criterion C of general diagnostic criteria for secondary headache.

**Conclusion**: This curious anecdote testifies that the intensity of CSH may be so severe to induce a temporary interruption of a concert held in front of a crowd of enthusiast fans. In general terms, a causative factor, independently from its nature, is clearly recognizable for inducing both CSH and EPH. Therefore, the primary or secondary nature of these forms of headache and their classification will be discussed on the basis of the literature on this subject.

**References**

1. Headache Classification Committee of the International Headache Society. The International Classification of Headache Disorders: 3^rd^ edition (beta version). Cephalalgia. 2013; 33: 629-808.

2. Rasmussen BK, Olesen J. Symptomatic and nonsymptomatic headaches in a general population. Neurology. 1992; 42: 1225-1231.

3. Fuh JL, Wang SJ, Lu SR, Juang KD. Ice-cream headache--a large survey of 8359 adolescents. Cephalalgia. 2003; 23: 977-981.

4. http://www.billboard.com/articles/news/7494667/bruce-springsteen-record-metlife-stadum (visited 6.8.2017).

### P84 Implementation of improved working practices in a chronic migraine Botox® clinic has increased treatment capacity by up to 60% with the same resources

#### Rebecca Stuckey, ^1^ April Bostock, ^1^ Stuart Weatherby, ^1^ Gemma Taylor, ^1^ Donna Clewer, ^1^ Paul Button, ^2^ Julie Button^2^

##### ^1^Neurology Department, Plymouth Hospitals NHS Trust, UK; ^2^ProcEx Solutions Ltd., Wales, UK

**Objective:** Chronic migraine is defined as headaches occurring on 15 or more days each month, at least half of which have migraine symptoms. Botox® (onabotulinum toxin A) is used extensively in the UK National Health Service for the treatment of chronic migraine. We provide this treatment regimen to our patients in a nurse-led clinical service. The demand for the treatment is increasing and we needed to find more efficient ways of running the service to meet the increasing demands. In 2016 we had a waiting list for treatment with a number of patients breaching the 18 week ‘treat-by-date’. Our main objective was to eliminate the waiting list by treating substantially more patients in each clinic session and avoiding any further breaches of the 18 week guideline. To do this we needed to develop an efficient and standardised process without adding additional resources into the service.

**Method:** We undertook a structured work improvement program partnering with a company called *ProcEx Solutions Ltd*. We utilised the model of firstly measuring our current process (assessment) and then secondly implementing standardised improvements (kaizen) as identified in the measurement phase. Measurements were made of nurse walking distances due to clinic room set-up, time taken to reconstitute and draw up Botox and we also assessed the amount of ‘down-time’ in the clinics. Different categories of patients were assessed and we observed that certain patients would require significantly more clinic time than others. We were allocating 30 minute time slots to all patients but we realised that we could segregate the patients into categories of 15 and 30 minute time slots. The table below illustrates the increase in annual capacity by using this approach.

**Table:** 

Clinics	Clinic Times	Frequency	Current Annual Capacity	New Annual Capacity
Tuesday AM	09:00-12:30	Weekly	336	546
Wednesday AM	09:00-12:30	Monthly	96	156
Wednesday PM	13:30-16:30	Monthly	84	132
Thursday PM	13:30-16:30	Monthly	84	132
Friday PM	13:30-16:30	Fortnightly	168	264
			768	1230
			% Increase	60%

**Results:** We realised additional clinic capacity of 462 treatments per year, an increase of 60%. We observed actual increases from 292 treatments in the second half of 2016 to 395 treatments in the first half of 2017, an actual increase of 35%, with the same number of staff.

**Conclusion:** The assessment of our working practices has identified opportunities to improve our efficiencies. The subsequent implementation of an improved process has enabled us to treat significantly more patients without increasing the number of clinical staff. We continue to see further increases in the number of patients we are able to treat.

### P85 Zonisamide for headache prophylaxis in dementia

#### Yunju Choi^1^, Seung H. Lee^2^, Myeong K. Kim^2^, Kyung W. Kang^2^

##### ^1^Department of neurology, Presbyterian Medical Center, Jeonju, Republic of Korea, 54987; ^2^Department of neurology, Chonnam National University Hospital, Gwangju, Republic of Korea, 61469

###### **Correspondence:** Yunju Choi (neurologist16@gmail.com)

**Background**

Zonisamide is an antiepileptic drug that shows broad spectrum of anticonvulsant activity. Furthermore, recent studies have demonstrated additional therapeutic use in migraine prophylaxis [1]. Zonisamide, a sulfonamide analog, is a drug with mechanisms of action similar to topiramate. Also these two drugs have similar adverse effects, such as weight loss, ureteric stone. In contrast with topiramate, zonisamide has no cognitive impairment or learning difficulty [2]. Therefore zonisamide could be one of the therapies for prevention of headache in patients with dementia.

**Materials and Methods**

We enrolled 10 Alzheimer’s disease patients with migraine headache, retrospectively. For headache, we investigated headache frequency with severity by using their headache diary and visual analog scales (VAS). Mini-mental status examination (MMSE), clinical dementia rating (CDR) was checked on visit and 3 months later.

**Results**

8 patients were male. The average add-on dose of zonisamide was 230 (range: 200-300). Frequency and severity of headache was also improved. The baseline average VAS was 6.9 (range: 6-8) and follow-up VAS was 3.8. The headache frequency declined from 16.4 times per month to 8.3. There is no significant change on MMSE and CDR.

**Conclusions**

Our findings support the use of zonisamide and an effective therapy for headache prophylaxis in patients with dementia.

**References**

1. Assarzadegan F, Tabesh H, Hosseini-Zijoud SM, Beale AD, Shoghli A, Ghafoori Yazdi M, et al. Comparing Zonisamide With Sodium Valproate in the Management of Migraine Headaches: Double-Blind Randomized Clinical Trial of Efficacy and Safety. Iran Red Crescent Med. J. 2016;18:e23768.

2. Lee HJ, Son JM, Mun J, Kim DW. Safety and Efficacy of Zonisamide in Patients with Epilepsy: A Post-Marketing Surveillance Study. J Epilepsy Res. 2015;5:89-95.

### P86 Postural headache and spontaneous intracranial hypotension: clinical features, neuroimaging and quality of life improvements in patients treated with blind epidural blood patch: a case series

#### Marco Mercieri^1^, Riccardo Chierichini^1^, Barbara Silvestri^2^, Roberto Arcioni^1^

##### ^1^ Department of Medical and Surgical Science and Translational Medicine, Sapienza University of Rome and Pain Therapy Unit, Sant'Andrea Hospital, Rome, IT; ^2^ Unit of Anaesthesia, Intensive Care and Pain Management, Campus Bio-Medico University, Rome, Italy

###### **Correspondence:** Marco Mercieri (marco.mercieri@uniroma1.it)

Spontaneous Intracranial Hypotension (SIH) is a rare syndrome (incidence: 5/100.000) [1] reportedly described over the past years [1,2]. Although SIH can produce a broad spectrum of clinical presentations, most patients experience postural headache. A conservative treatment is advocated as first-line therapy for patients with SIH, but in refractory cases an epidural blood patch (EBP) may be attempted [3,4]. We aimed to assess the efficacy of blind lumbar EBP in a case series of patients with postural headache and spontaneous intracranial hypotension.

**Methods.** We retrospectively analysed patients with SIH treated with EBP in our Centre. Information regarding demographics, radiology and clinic follow up was extracted from an electronic patient record system. The patients were asked to answer questions regarding their daily life activities using the 12-Items Short Form Survey (SF-12) questionnaire before and after 1 month from the procedure (following our Pain Unit protocols). Data regarding their global impression of change 1-week after treatment were collected. All patients underwent Brain MRI after 6-month from BP to evaluate radiologic resolution and the data were compared to the clinical evolution.

**Results.** Five patients who underwent lumbar epidural blood patching were analysed (3 female; mean age 41 years). The site of Cerebrospinal Fluid leak was evident in 4 patients (3 through spinal MRI, 1 through Cisternography) (Tab. 1). Three patients reported instantaneous headache resolution, 2 responded only partially, having symptoms persisting for 1-2 weeks before recovering and developed a chronic headache without postural component. All patients reported improvements in Physical Component Summary and Mental Component summary after the procedure (p<0,02), and all patients had impression of benefit 1-week after treatment. MRI showed normal findings at 6-month follow-up.

**Conclusions.** A blind blood patch procedure can provide complete resolution in headache symptoms in selected patients. Our patients who did not achieve instantaneous complete symptoms resolution after the procedure, still showed improvements in their quality of life and reported a noticeable change after the procedure, showing that EBP might be beneficial also for those cases in which complete relief is not instantaneous.

**Table 1 (abstract P86).** See text for description

Case n.	Sex	Age	Spine NMR	Cisternography	Leakage	Blood administered (ml)	n° of Blood Patches
1	F	35	+		T2-T3	20	1
2	M	57	-	+	L2-L3	20	1
3	M	43	+		C7-T1	20	1
4	F	45	+		T12-L1	20	2
5	F	27	-		/	20	1

Note: + = evidence of liquor leakage, - = not evidence of liquor leakage

**References**

1 Kranz, Peter G., Michael D. Malinzak, Timothy J. Amrhein, and Linda Gray. “Update on the Diagnosis and Treatment of Spontaneous Intracranial Hypotension.” *Current Pain and Headache Reports* 21, no. 8 (August 2017): 37. doi:10.1007/s11916-017-0639-3.

2 Limaye, Kaustubh, Rohan Samant, and Ricky W. Lee. “Spontaneous Intracranial Hypotension: Diagnosis to Management.” *Acta Neurologica Belgica* 116, no. 2 (June 2016): 119–25. doi:10.1007/s13760-015-0577-y.

3 Davidson, Benjamin, Farshad Nassiri, Alireza Mansouri, Jetan H. Badhiwala, Christopher D. Witiw, Mohammed F. Shamji, Philip W. Peng, Richard I. Farb, and Mark Bernstein. “Spontaneous Intracranial Hypotension: A Review and Introduction of an Algorithm For Management.” *World Neurosurgery* 101 (May 2017): 343–49. doi:10.1016/j.wneu.2017.01.123.

4 Mokri, B., D. G. Piepgras, and G. M. Miller. “Syndrome of Orthostatic Headaches and Diffuse Pachymeningeal Gadolinium Enhancement.” *Mayo Clinic Proceedings* 72, no. 5 (May 1997): 400–413. doi:10.1016/S0025-6196(11)64858-1.

### P87 Retinal migraine: three case reports

#### Vlasta Vukovic Cvetkovic, Lars Bendtsen, Jes Olesen

##### Danish Headache Center, Rigshospitalet-Glostrup, Copenhagen, Denmark

###### **Correspondence:** Vlasta Vukovic Cvetkovic (vlasta.vukovic@uclmail.net)

**Background:** Retinal migraine is a rare form of migraine. According to the ICHD-3 beta criteria the diagnosis requires an aura consisting of fully reversible monocular positive and/or negative visual phenomena (e.g., scintillations, scotomata or blindness) confirmed during an attack by 1. clinical visual field examination and/or 2. the patient’s drawing of a monocular field defect and at least two of the following three characteristics: 1. the aura spreads gradually over ≥5 min, 2. aura symptoms last 5-60 min, 3. aura is accompanied, or followed within 60 min, by headache.

**Case reports:** We present three cases of patients with retinal migraine, they all fulfill ICHD criteria but have some “case specific” interesting features:

1. A 29-year old woman with aura always on her left eye. The patient experiences first a slight pressure beyond her eye, followed by an aura described as slowly blackening of her visual field in all 4 quadrants, or scintillating scotoma, lasting 3-5 minutes, followed by complete normalization of her visual field. In 50% of attacks she has a subsequent mild headache lasting 10-30 minutes. She has 1-2 attacks per month. Case specific: headache is present in only 50% of attacks.
2. A 40-year old woman with aura always on her left eye, beginning with palpitation, sense of fear, followed by grey scintillating scotoma, followed by complete blindness, with complete resolution after 10 minutes. Aura is followed by a mild headache lasting couple of hours to several days. She has 1 attack per month. Case specific: has additional “autonomic aura symptoms”.
3. A 69-year old man, with migraine without aura and separately sudden attacks of complete visual loss lasting from 10 seconds to 10 minutes on right or left eye, rarely both. Simultaneously pressing headache behind the affected eye, duration 5 minutes to 6 hours with foto-and fonofobia, slight nausea and aggravation by physical activity. He has 3 attacks per month. Case specific: pt. experiences sudden visual loss, not march, symptoms are always reversible.

Common to all cases: never typical (hemianoptic) visual aura, sensory, speech or motor aura; accompanying headache is of mild intensity, in 2 cases not migrainous; in 2 cases aspirin had no effect on migraine.

**Conclusion:** Although rare, retinal migraine should not be misdiagnosed as amaurosis fugax, papilledema or optic disc disease, transient visual obscuration or Uhthoffs phenomenon.

**Consent for publication:** The authors declare that written informed consent was obtained for publication.

### P88 Changes in grey matter volume and functional connectivity in cluster headache versus migraine

#### Silvia Benemei^1^, Antonio Giorgio^2^, Jian Zhang^2^, Chiara Lupi^1^, Marzia Mortilla^3^, Antonio Federico^2^, Pierangelo Geppetti^1^, Francesco De Cesaris^1^, Nicola De Stefano^2^

##### ^1^Department of Health Sciences, University of Florence, Florence, Italy; ^2^Department of Medicine, Surgery and Neuroscience, University of Siena, Siena, Italy; ^3^Anna Meyer Children’s University Hospital, Florence, Italy

###### **Correspondence:** Silvia Benemei

**Background**

Recent MRI studies have shown structural and functional abnormalities in the brain of cluster headache (CH) and migraine (M) patients. However, no between-group comparison of MRI measure has been performed.

**Materials and methods**

Multimodal MRI was acquired on a 3T MR scanner in age-matched patients with CH (n=12), M without aura (n=13), both during attack-free period, and normal controls (NC, n=13). MRI processing was performed with FSL. Voxelwise analyses of variance were done with nonparametric permutation testing (p<= 0.05, corrected).

**Results**

Compared to NC, higher grey matter volume (GMV) occurred in CH in the cerebellum and occipital fusiform gyrus and in M in the lateral occipital cortex (LOC). GMV was lower than in NC in the inferior frontal gyrus of CH and in the lingual gyrus of M. Compared to M, GMV of CH was higher in the cerebellum and lower in the frontal pole and LOC. Functional connectivity (FC) was, compared to NC, higher in CH in the default mode, working memory and executive networks (DMN, WMN, EN) and altered in DMN of M, with lateral increase and medial decrease. FC in WMN and EN of CH was also higher than M. FC between cerebellar and temporo-insular networks was higher in CH than M.

**Conclusions**

The brain of attack-free CH seems to be characterized, compared to M, by (i) GMV changes, with decrease in a classical pain processing region (frontal cortex) and increase in an atypical region (cerebellum) and (ii) increased FC in key cognitive networks, with a possible maladaptive role.

### P89 Intercepting migraine in its path towards chronicity

#### Angela Koverech, Paolo Martelletti^1,2^

##### ^1^Department of Clinical and Molecular Medicine, Sapienza University, Rome Italy; ^2^Regional Referral Headache Centre, Sant’Andrea Hospital, Via di Grottarossa 1035, 00189 Rome, Italy

###### **Correspondence:** Paolo Martelletti (paolo.martelletti@uniroma1.it)

Today there is the need of analysis of individual monoclonal antibody studies for CGRP or CGRPr in migraine, and also on the future role of these drugs in a disease with such a large impact on the general population. Fourteen percent of the global population suffers from migraine, and we now have to imagine how to proceed in the dissemination of these new drugs. Excluding the prophylaxis of the episodic form of migraine, we will have to postpone for ethical and pharmacoeconomic reasons the choice to treat with CGRP(r) mAbs the very low frequency migraine subpopulation (4-6 migraine days per month). Unfortunately, the tight application of existing guideline-recommended drugs could make this choice difficult. We must instead focus attention towards the highly disabling and costly forms; otherwise we would hypothesize treating almost one billion people with migraine. However, only 2% of the world's population suffers from chronic migraine (with or without pharmacological abuse of acute drugs – analgesics, non-steroidal anti-inflammatory drugs, triptans, opioids, ergot derivatives, barbiturates, caffeine, etc. and their various combinations) and this chronic form is the current true challenge in headache management. We should attempt to prevent the chronicization of these patients, who constitute the core group with personal-social-working burden, and who fluctuate in a pre-chronic phase with high-frequency migraine crisis but who are not yet chronic. Only in this way will we reduce the huge number of chronic migraine patients, who stray towards medication overuse headache (MOH) and are at risk of refractoriness. And only in this way will we reduce the cascade of multi-systemic pathologies (gastrointestinal, renal, cardiovascular, cerebral, psychiatric, etc.) originating from acute drug abuse, which are often not recognized or directly correlated with it. This is a top priority, because we know that the rehabilitation procedures for chronic migraine complicated by MOH present with relapse in 50% of patients within the first 12 months. Another priority area of intervention is refractory migraine, as clinically defined by the European Headache Federation. Refractory migraine is very hard to treat and has been the target for a number of ineffective invasive techniques. These priority areas could represent immediate targets for the application of the new CGRP mAbs.

**Reference**

Martelletti P. The application of CGRP(r) monoclonal antibodies in migraine spectrum: needs and priorities. Biodrugs 2017 in press.

### P90 Induction of migraine-like photophobia by PACAP-38: A potential target in migraine treatment

#### Adisa Kuburas^1^, Bianca N. Mason^2^, Maria-Christina M. Loomis^4^, Leon F. Garcia-Martinez^4^, Andrew F. Russo^1,2,3^

##### ^1^Department of Molecular Physiology and Biophysics; ^2^Molecular and Cellular Biology Program, ^3^Veterans Affairs Medical Center; University of Iowa, Iowa City, Iowa 52242; ^4^Alder Biopharmaceuticals; Bothell, Washington 98011

###### **Correspondence:** Andrew F. Russo

Migraine is a disabling neurological disorder and a significant public health problem. The pathophysiology of migraine is not well understood, but studies have shown that calcitonin gene-related peptide (CGRP) plays a strong role. Recently there has been a significant interest for role of pituitary adenylate cyclase-activating polypeptide-38 (PACAP38) in migraine. We have previously shown that administration of PACAP38 induces photophobia similar to CGRP-induced photophobia in wild-type CD1 mice. As a surrogate to photophobia, we measured light aversion behavior. The objective of this study is to determine if pretreatment with anti-PACAP antibodies will inhibit the PACAP-induce light aversion. We have also used anti-PACAP and anti-CGRP antibodies to better understand the relationship between PACAP and CGRP in their involvement in migraine.

Our results show that intraperitoneal (ip) injection of anti-PACAP38 antibody was able to inhibit PACAP-induced light-aversive response in CD1 mice compared to mice treated with control antibody. In addition, PACAP38-induced increase in resting time in the dark, reflecting pain as experienced during migraine, was inhibited by anti-PACAP antibody. Our results also suggest that PACAP and CGRP may work through different pathways as the cross antibody treatment was not able to inhibit PACAP or CGRP-induce light aversion. Future work will attempt to understand more about PACAP38 in migraine and to explore PACAP as a potential novel target in migraine therapy.

### P91 Burden and impact of migraine: a caregiver’s perspective

#### Elena Ruiz de la Torre^1^, Paolo Martelletti^2,3^, Audrey Craven^1,4^, Donna Walsh^4^, Simon Evans^5^, Paula Dumas^6^, Hans-Christoph Diener^7^, Michel Lanteri-Minet^8^, Todd J. Schwedt^9^, Jean-Pierre Malkowski^10^, Monisha Sodha^11^, Susann Walda^11^, Anne Aronsson^11^, Annik Laflamme^10^, Pamela Vo^10^

##### ^1^European Headache Alliance, Brussels, Belgium; ^2^European Headache Federation, Rome, Italy; ^3^Department of Clinical and Molecular Medicine, Sapienza University of Rome, Rome, Italy; ^4^European Federation of Neurological Associations; ^5^Migraine Action, Leicester, LE1 6NB, UK; ^6^Migraine Again, USA; ^7^Department of Neurology and Headache Center, University of Duisburg-Essen, Essen, Germany; ^8^Département d’Evaluation et Traitement de la Douleur, Centre Hospitalo-Universitaire de Nice, Hôpital de Cimiez, 06300 Nice, France; ^9^Department of Neurology, Mayo Clinic, USA; ^10^Novartis Pharma AG, 4002 Basel, Switzerland; ^11^GfK Switzerland, 4058 Basel, Switzerland

###### **Correspondence:** Pamela Vo (pamela.vo@novartis.com)

**Background**

Migraine has far-reaching impacts on the family of individuals with migraine but limited evidence is available to describe this reality. To better understand the full burden of migraine, this study sought to describe the impact of migraine from the caregiver’s perspective.

**Materials and Methods**

This cross-sectional study was conducted using Online Bulletin Boards (OBB), an interface developed for online survey, discussions and interactions led by a trained facilitator over 4 consecutive days. Caregivers aged ≥25 years who were caring for an adult migraine patient in their household were recruited to participate in 3 OBBs in Germany, Italy and USA (1/country). Participants were blinded to each other and agreed to partake daily for ≥30 minutes in the OBBs, where they were asked to respond to specific questions on migraine and provide their perspectives on statements and other participants’ blinded responses. All responses were aggregated by country and qualitatively analyzed.

**Results**

A total of 30 caregivers participated in this pilot phase of a large global study (10/country). All caregivers reported that they were highly involved in the management of migraine and that they spent an average of 15 hours/month supporting their family member suffering from migraine. 83% of respondents (n=25/30) reported feeling stressed, exhausted and overwhelmed with the amount of work resulting from their caregiving activities. They described themselves as powerless witnesses of the frequent and intense suffering of their loved ones, wanting to be helpful while knowing the limits of what they could do. 60% of caregivers (n=18/30) reported a worsening of personal relationships over time due to migraines. Regardless of whether they were employed, unemployed or retired, 83% of caregivers (n=25/30) reported that their caregiver role affected their lives due to the significant changes it imposed on their own daily routines/schedules. This affected private life, social engagements, relationships, and professional obligations, including fear of losing employment. Most caregivers (87%, n=26/30) reported ambivalent feelings, being torn between commitment, self-sacrifice and resentment. All agreed that migraine patients were thankful for their help and assistance.

**Conclusions**

Caregiving has a major impact on the lives of close relatives supporting migraine patients. This study highlights how the burden of migraine extends beyond the patients, with substantial functional and emotional impacts of the disease on caregivers. This pilot confirmed the unique opportunity that this type of study provides for insights into the caregivers’ experience and unmet needs in the current treatments available to migraine patients.

**Funding**

This study was funded by Novartis AG, Switzerland.

**Acknowledgements**

This poster has been previously presented at the 18th Congress of the International Headache Society, 7-10th September 2017, Vancouver, Canada.

### P92

**Withdrawn**

### P93 Real-world patient perspective on the burden and impact of migraine

#### Elena Ruiz de la Torre^1^, Paolo Martelletti^2,3^, Audrey Craven^1,4^, Donna Walsh^4^, Simon Evans^5^, Paula Dumas^6^, Hans-Christoph Diener^7^, Michel Lanteri-Minet^8,^ Todd J. Schwedt^9^, Jean-Pierre Malkowski^10^, Monisha Sodha^11^, Susann Walda^11^, Anne Aronsson^11^, Annik Laflamme^10^, Pamela Vo^10^

##### ^1^ European Headache Alliance, Brussels, Belgium; ^2^ European Headache Federation, Rome, Italy; ^3^ Department of Clinical and Molecular Medicine, Sapienza University of Rome, Rome, Italy; ^4^ European Federation of Neurological Associations; ^5^ Migraine Action, Leicester, LE1 6NB, UK; ^6^ Migraine Again, USA; ^7^ Department of Neurology and Headache Center, University of Duisburg-Essen, Essen, Germany; ^8^ Département d’Evaluation et Traitement de la Douleur, Centre Hospitalo-Universitaire de Nice, Hôpital de Cimiez, 06300 Nice, France; ^9^ Department of Neurology, Mayo Clinic, USA; ^10^Novartis Pharma AG, 4002 Basel, Switzerland; ^11^ GfK Switzerland, 4058 Basel, Switzerland

###### **Correspondence:** Pamela Vo (pamela.vo@novartis.com)

**Background**

Migraine is a prevalent condition affecting about 11% of the adult population. It has debilitating symptoms and affects patient functioning. The present study was undertaken to understand the full burden and impact of migraine in everyday life from the patient’s point of view.

**Materials and Methods**

This cross-sectional study was conducted using Online Bulletin Boards (OBB). This interface was developed for online survey, discussions and interactions led by a trained facilitator over a period of 4 consecutive days. Adults with chronic and episodic migraine aged between 25 and 60 years old were recruited to participate in 6 OBBs established in Germany, Italy and USA (2 per country). Participants were blinded to each other and agreed to partake for at least 30 minutes each day in the OBBs, where they were asked to respond to specific questions on migraine and to provide their perspective on statements and other participants’ blinded responses. All responses were aggregated by country and qualitatively analyzed.

**Results**

A total of 60 migraine patients participated in this pilot phase of a large global study (20 per country). About half (47%, n=28) reported having been diagnosed with migraine, either by a general practitioner (GP) or neurologist within the year following the date of their 1^st^ symptoms. Table 1 summarizes the 3 most common migraine attack triggers, symptoms, and coping mechanisms reported by patients. All respondents reported important limitations resulting from migraines in private, professional and social aspects of life, mainly the disruption of daily routines, significant strain on personal relationships, difficulty caring for children, and missed days of work, deadlines, or social events. Anxiety and frustration were most frequently reported as emotional consequences of migraine in private/social life (92% and 72%) and work (97% and 88%). 87% of patients (n=52) had seen a physician for migraine management but many (85%, n=51) did not consult regularly, especially if their diagnosis had occurred long ago. Two thirds (n=38 / 63%) of respondents reported getting functional and emotional support from family and friends, but wished for improved understanding/compassion from others and more efficacious medications.

**Conclusions**

This study highlights the substantial functional and emotional burden migraine exerts on individuals, as well as the significant unmet needs that remain for these patients despite currently available care and treatment options.

**Table 1 (abstract P93).** Main Migraine Triggers, Symptoms, and Coping Mechanisms

	3 Most Common per Category		
Migraine attack triggers	Bright light	Loud/repetitive sounds	Stress
	97%(n=58)	93%(n=56)	93%(n=56)
Migraine symptoms	Pounding/throbbing	Photophobia	Sensitivity to sound and noise
	97%(n=58)	97%(n=58)	93%(n=56)
Coping mechanisms	Lying down in a darkened room	Avoidance of sounds/noise	Medication use
	97%(n=58)	95%(n=57)	77%(n=46)

**Funding**

This study was funded by Novartis AG, Switzerland.

**Acknowledgements**

This poster has been previously presented at the 18th Congress of the International Headache Society, 7-10th September 2017, Vancouver, Canada.

### P94 Efficacy of Onabotulinumtoxin A and Physical Therapy combined treatment in chronic migraine

#### Antonio Granato^1^; Antonella Monticco^2^; Manuela Deodato^2^; Ugogiulio Sisto^1^; Mariana Ridolfi^1^; Roberto Marcovich^2^; Paolo Manganotti^1^

##### ^1^Department of Medical, Technological and Translational Sciences, Headache Centre, University of Trieste, Italy; ^2^University of Trieste, Italy

**Background**

Physical therapy (PT) is used as single or complementary treatment for headache. Onabotulinum toxin A (BoNT-A) has proven to be effective in chronic migraine (CM) prophylaxis. Currently no clinical studies about BoNT-A *plus* PT combined treatment (CT) are available.

**Objectives**

To compare the efficacy of BoNT-A *plus* PT combined treatment with the single therapeutic options in CM.

**Material and methods**

A three-arm randomized perspective study of patients suffering from CM admitted to the Headache Centre of the University of Trieste was performed. All the patients underwent a clinical postural evaluation at the beginning of the study. They had a one-month observation period (baseline), then they were treated with BoNT-A, PT or CT. BoNT-A was administred following the “fixed site/fixed dose” and “follow-the-pain” protocol, PT was based on combination of postural advice, relaxation training, exercises and manual treatment on trigger points, reinforcement and postural modification. Number of responders (>50% reduction of headache days), headache days, headache hours, symptomatic drug intake, disability (MIDAS) in baseline and at the three-month follow-up visit were analyzed with SPSS 21.0.

**Results**

We enrolled 25 patients (22 F, 3 M; mean age 54 [34-79] years), 13 patients were treated with BoNT-A, 8 patients with CT, 4 patients with PT. Both groups of patients treated with BoNT-A and CT had a reduction of number of headache days (BoNT-A: Baseline=26 [16-30] vs Three-month visit=12 [4-30] (p=0.003); CT: Baseline=21 [13-30] vs Three-month visit=11 [3-29] (p=0.012)), symptomatic drugs (BoNT-A: Baseline=21 [6-57] vs Three-month visit=14 [0-21] (p=0.025); CT: Baseline=22 [11-41] vs Three-month visit=9 [1-41] (p=0.018)), and MIDAS (BoNT-A: Baseline=93 [62-210] vs Three-month visit=57 [10-179] (p=0.002); CT: Baseline=85 [35-124] vs Three-month visit=34 [12-187] (p=0.03)). The reduction of headache days, symptomatic drugs and MIDAS were comparable in both BoNT-A and CT patients (p=NS). Only patients treated with BoNT-A improved in total headache hours (BoNT-A: Baseline=241 [46-737] vs Three-month visit=121 [12-214] (p=0.005)). Percentage of responders was higher in CT than in BoNT-A group (CT=62% vs BoNT-A= 38%). Effectiveness measures did not improved in the four patients who received only PT.

**Conclusions**

Physical therapy is an effective add-on treatment to BoNT-A for CM prophylaxis. Patients treated with combined therapy BoNT-A *plus* PT had a higher percentage of responders than patients treated with only BoNT-A. The other treatment efficacy measures equally reduced in both CT and BoNT-A groups. The few patients treated with only PT did not improved, however a higher sample is required.

### P95 The antimigraine butterbur ingredient, isopetasin, desensitizes peptidergic nociceptors *via* the TRPA1 channel activation *in vitro*

#### De Logu F^1^, Benemei S^1^, Li Puma S^1^, Marone IM^1^, Coppi E^1^, Ugolini F^1^, Liedtke W^2^, Pollastro F^3^, Appendino G^3^, Geppetti P^1^, Materazzi S^1^ and Nassini R^1^

##### ^1^Department of Health Sciences, Section of Clinical Pharmacology and Oncology, University of Florence, Florence, Italy; ^2^Departments of Neurology, Anesthesiology and Neurobiology, Clinics for Headache, Head-Pain and Trigeminal Sensory Disorders, Duke University, Durham, NC 27710 USA; ^3^Department of Pharmaceutical Sciences, University of Eastern Piedmont, Novara, Italy

###### **Correspondence:** De Logu F

For hundreds of years, butterburs (Petasites), herbaceous perennial plants belonging to the Asteraceae, which includes also Tanacetum parthenium L., have been used by folk medicine of northern Eurasia and America for therapeutic purposes, including treatment of fever, respiratory diseases, spasms, and pain. Among the number of compounds contained in common butterbur [Petasites hybridus (L.) Gaertn.], the major constituents, petasin and isopetasin, are considered responsible for the antimigraine effects of the herbal extract. The mechanism of the antimigraine action of butterbur [*Petasites hybridus* (L.) Gaertn.] is unknown. Here, we investigated the ability of isopetasin, a major butterbur constituent, to specifically target the transient receptor ankyrin 1 (TRPA1) channel and to affect functional responses relevant to migraine.

Single cell calcium imaging and patch-clamp recordings in human and rodent TRPA1-expressing cells, neurogenic motor responses in isolated rat urinary bladder, release of calcitonin gene related peptide (CGRP) from mouse spinal cord *in vitro,* were examined*.* Isopetasin produced (i) calcium responses and currents in rat/mouse trigeminal ganglion (TG) neurons and in cells expressing the human TRPA1, (ii) substance P-mediated contractions of isolated rat urinary bladders and (iii) CGRP release from mouse dorsal spinal cord, responses that were selectively abolished by TRPA1 genetic deletion/pharmacological antagonism. Preexposure to isopetasin produced marked desensitization of allyl isothiocyanate (AITC, TRPA1 agonist)- or capsaicin (TRPV1 agonist)-evoked currents in rat TG neurons, contractions of rat urinary bladder and CGRP release from central terminals of primary sensory neurons.

TRPA1 agonism by isopetasin results in excitation of neuropeptide-containing nociceptors that is followed by remarkable neuronal desensitization. Such attenuation in pain and neurogenic inflammation may account for the antimigraine action of butterbur.

### P96 Use of acute headache and migraine medications in patients with episodic migrainein the STRIVE Phase 3 trial of erenumab for migraine prevention

#### Uwe Reuter^1^, Jo Bonner^2^, Gregor Broessner^3^, Yngve Hallstrom^4^, Hernan Picard^5^, Sunfa Cheng^5^, Feng Zhang^5^, Dan Mikol^5^, Jan Klatt^6^

##### ^1^Department of Neurology, Charité Universitätsmedizin Berlin, Berlin, Germany; ^2^Mercy Research, St Louis, MO, USA; ^3^Department of Neurology, Medical University of Innsbruck, Innsbruck, Austria; ^4^Stockholm Neuro Center, Stockholm, Sweden; ^5^Amgen Inc., Thousand Oaks, CA, USA; ^6^Novartis Pharma AG, Basel, Switzerland

###### **Correspondence:** Uwe Reuter (uwe.reuter@charite.de)

**Background**

Erenumab is a fully human monoclonal antibody that selectively inhibits the calcitonin gene-related peptide (CGRP) receptor. In the STRIVE placebo-controlled Phase 3 trial in patients with episodic migraine (EM; clinicaltrials.gov NCT02456740), erenumab 70 mg and 140 mg administered monthly by subcutaneous injection achieved the primary endpoint of significantly reducing the number of monthly migraine days versus placebo. Erenumab also achieved the secondary endpoint of reducing the number of days/month versus placebo on which patients used acute migraine-specific medications (AMSM, triptans or ergotamines; −0.9 and −1.4 days/month averaged over Months 4−6 for erenumab 70 mg and 140 mg, respectively [p<0.001 for each dose]). This outcome, measured for the overall study population, was achieved despite ~40% of patients not taking AMSM at baseline, for whom change could only be ≥0. Here we present two further analyses: (A) change in any acute headache medication days/month (AHM, including AMSM as well as paracetamol, NSAIDS etc,) in the overall study population as 97.7% used AHM during baseline; (B) change in AMSM days/month in the subgroup who used AMSM during baseline.

**Methods**

Patients recorded medication use with a daily eDiary throughout the 4-week baseline period and subsequent 6-month double-blind treatment period. The number of days per month on which AMSM and AHM were taken was calculated. Change from baseline was analyzed using a linear mixed effects model including covariates of treatment, visit, treatment by visit interaction, stratification factors (region and migraine prophylaxis medication status), and baseline value. Nominal p-values were provided without multiplicity adjustment.

**Results**

(A) During baseline, the mean number of AHM days/month in the overall population was 6.91, 6.58, and 6.60 in the placebo, erenumab 70 mg, and erenumab 140 mg groups, respectively. Erenumab significantly reduced AHM days/month versus placebo from Month 1 onward (Fig. 1). (B) During baseline, 561 of 955 patients (58.7%) used AMSM. Mean number of AMSM days/month in patients who used AMSM during baseline was 5.68, 5.67, and 5.67 in the placebo, erenumab 70 mg, and erenumab 140 mg groups, respectively. Erenumab significantly reduced AMSM days/month versus placebo; treatment difference −1.57 days (−2.04, −1.10; p<0.001) for 70 mg and −2.30 days (−2.77, −1.83; p<0.001) for 140 mg, averaged over Months 4−6.

**Conclusion**

Erenumab significantly reduced both AHM and AMSM days/month versus placebo in patients with EM.

**Fig. 1 (abstract P105).**
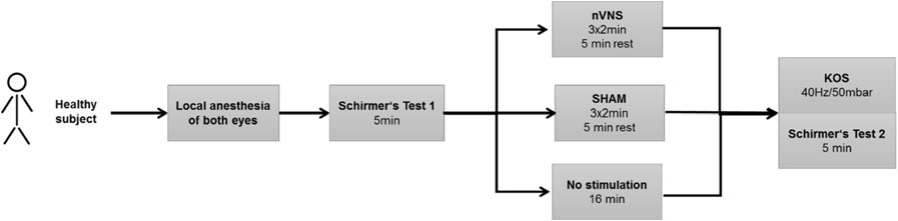
Study design

### P97 Ex-Migraine: Clinical and Neurophysiological Analysis

#### Vera Osipova^1^, Alexey Sergeev^2^, Elena Snopkova^1^, Ada Artemenko^1^, Guzial Tabeeva^2^, Zaza Katsarava^3,4^

##### ^1^Research Department of Neurology, Sechenov University, Moscow, Russia; ^2^Department of Neurology and Neurosurgery, Sechenov University, Moscow, Russia; ^3^Department of Neurology, University of Duisberg-Essen, Essen, Germany; ^4^Department of Neurology, Evangelical Hospital Unna, Unna, Germany

**Background**

The evolution of migraine (M) after the age of 50 has been insufficiently studied; sub-population with former M was not studied at all. Aim of investigation was two-fold: 1. to present clinical portrait of patients with former/Ex-Migraine (Ex-M) with special focus on retrospective analysis of M in the active period of the disease (at the age of 30-40) and 2. to study the level of cortical excitability in elderly Ex-M subjects.

**Material and methods**

The Ex-M group (n=18) was inhomogeneous and composed of subjects with complete regress of M attacks (both pain and M aura; CRM, n=11, m. age 60.0±9) and those keeping M aura only - painless M/Late-life Migraine Accompaniments (LLMAs, n=7, m.age 54.0±7). All patients underwent clinical interview focused on present and former clinical manifestations and confirming past-diagnosis of M using Migraine History Inventory specially elaborated by us for this study. Cortex excitability (amplitude and habituation for the N75-P100 component) was measured using pattern-reversal visual evoked potentials (PR-VEP)

**Results**

Compared to subjects with LLMAs in patients with CMR migraine terminated a decade later (39±9 vs 49.7, p<0.05), in the active period of disease the majority of patients had MoA (72.7% vs 42.9%, p<0.05), more long (5.0±8h vs 24.0±18h) and typical attacks with throbbing pain and vomiting; subjects with LLMAs more often suffered from MA. At the time of investigation CRM-Group had completely normal amplitude and habituation for the N75-P100 component, while LLMAs-Group demonstrated the signs of cortical hyperexcitability typical for actual M, namely, significant increase in total N75-P100 (p<0.05) and more marked dishabituation

**Conclusions**

1. LLMAs-Group (painless M with preserved aura) initially more often had MA, short and less typical attacks and even after loosing pain attacks keep the signs of cortical hyper excitability. It could be presumed that the phenomenon of cortical hyperexcitability in patients of any age serves the basis for preservation of M aura/LLMAs but not M pain.
2. Ex-M elderly patients who are completely free of both pain and M aura and have normal level of cortical excitability and habituation initially used to have MoA with long and typical attacks. Total normalization of brain reactivity could be regarded as a principal neurophysiological factor for complete regress of both pain and aura.

### P98 Reducing the impact of migraine on functioning: Results from the STRIVE trial, a phase 3, randomised, double-blind, placebo-controlled study of erenumab in patients with episodic migraine

#### Dawn C Buse^1^, Richard B Lipton^1^, Daniel D Mikol^2^, Andrew V Thach^2^, Pooja Desai^2^, Hernan Picard^2^, Yumi Kubo^2^, Asha Hareendran^3^, Ariane K Kawata^4^

##### ^1^Department of Neurology, Albert Einstein College of Medicine and Montefiore Medical Center, Bronx, NY, USA; ^2^Amgen Inc., Thousand Oaks, CA, USA; ^3^Evidera, London, UK; ^4^Evidera, Bethesda, MD, USA

###### **Correspondence:** Dawn C Buse (DBUSE@montefiore.org)

**Background**

The Migraine Functional Impact Questionnaire (MFIQ) is a newly developed 31-item patient-reported outcome instrument assessing the impact of migraine on functional outcomes over 7 days. The MFIQ includes 4 domains: impact on physical function, usual activities, social function, and emotional function with scores ranging from 0–100 (higher scores indicate greater impact; negative change scores represent reduction in impact [improvement]). In this phase 3 trial (NCT02456740), we evaluated the effect of erenumab, which is being developed as a preventive treatment for migraine in adults, on functional outcomes using the MFIQ.

**Methods**

In STRIVE, a total of 955 patients with episodic migraine aged 18–65 years were randomised 1:1:1 to receive monthly subcutaneous placebo, erenumab 70 or 140 mg. The MFIQ was completed at baseline and every 4 weeks for 24 weeks. The MFIQ scores were evaluated as change from baseline to the last three months of the double-blind treatment phase (defined as the average of scores in Months 4–6 [Weeks 13–24]). Pairwise comparisons of least squares mean changes from baseline in MFIQ domain scores were assessed for each treatment vs placebo. The p-values were nominal and not adjusted for multiplicity.

**Results**

Baseline MFIQ scores were similar in the erenumab and placebo groups in all 4 MFIQ domains (70 mg: mean±standard deviation [SD] 33.3±20.9; 140 mg: 32.0±21.2; placebo: 33.1±24.5). Greater reductions in impact from baseline were observed for each MFIQ domain with erenumab compared with placebo (Table 1).

**Conclusions**

Over 24 weeks, compared with placebo, patients treated with erenumab experienced greater reductions in the MFIQ scores, reflecting less impact of migraine on their physical functioning, usual activity, and social and emotional functioning, with numerically greater reductions for 140 mg compared with 70 mg. These improvements complement the efficacy of erenumab demonstrated by the clinical outcome measures in the STRIVE study.

**Table 1 (abstract P98).** Changes from baseline in the Migraine Functional Impact Questionnaire domain scores

	Least squares mean changes (95% confidence interval)			p-values (Erenumab vs Placebo)	
	Erenumab 70 mg	Erenumab 140 mg	Placebo	Erenumab 70 mg	Erenumab 140 mg
Physical function	−13.7 (−15.5, −11.9)	−15.1 (−17.0, −13.3)	−9.4 (−11.3, −7.6)	<0.001	<0.001
Usual activities	−12.3 (−13.9, −10.6)	−13.4 (−15.1, −11.8)	−8.3 (−10.0, −6.6)	<0.001	<0.001
Social function	−13.2 (−14.9, −11.4)	−14.5 (−16.2, −12.8)	−9.5 (−11.3, −7.7)	0.003	<0.001
Emotional function	−16.4 (−18.4, −14.5)	−18.4 (−20.3, −16.5)	−11.2 (−13.1, −9.2)	<0.001	<0.001

### P99 Reducing impaired days: Results from the STRIVE trial, a phase 3, randomised, double-blind study of erenumab for episodic migraine

#### Asha Hareendran^1^, Dawn C Buse^2^, Richard B Lipton^2^, Martha S Bayliss^3^, Daniel D Mikol^4^, Dennis A Revicki^5^, Feng Zhang^4^, Pooja Desai^4^, Hernan Picard^4^, Ariane K Kawata^5^

##### ^1^Evidera, London, UK; ^2^Montefiore Medical Center, Albert Einstein College of Medicine, Bronx, NY, USA; ^3^Optum, Lincoln, RI, USA; Amgen Inc., Thousand Oaks, CA, USA; Evidera, Bethesda, MD, USA

###### **Correspondence:** Asha Hareendran (asha.hareendran@evidera.com)

**Background**

Migraine Physical Function Impact Diary (MPFID) is a 13-item patient-reported outcome (PRO) measure that assesses the impact of migraine on Everyday Activities (EA) and Physical Impairment (PI) domains. We report the effect of erenumab, a preventive treatment for episodic migraine (EM) in adults, on monthly days with impairment as measured by the MPFID.

**Methods**

The MPFID was completed using an electronic diary every evening during a global, placebo-controlled, double-blind, 6-month, phase 3 STRIVE trial (NCT02456740) in which 955 adults with EM (18–65 years) were randomised 1:1:1 to subcutaneous, monthly placebo or erenumab 140mg or 70mg. Reponses to items in the MPFID EA and PI domains are on a 1–5 scale, with higher numbers indicating greater negative impact. A day with a response ≥3 on at least one item in a domain was defined an ‘impaired day’ (ID) for that domain, (i.e. EA-ID and PI-ID). Mean monthly number of IDs were summarised for the 4-week baseline period and each subsequent 4-week period. Changes from baseline in mean monthly EA-ID and PI-ID over the final 3 months (months 4–6) of the double-blind treatment phase (DBTP) were assessed as pre-specified exploratory endpoints in the STRIVE trial; primary and secondary endpoints are reported separately. All p-values are descriptive and not adjusted for multiplicity.

**Results**

At baseline, subjects in the erenumab and placebo groups had a similar number of mean monthly EA-ID (140mg: mean±standard deviation 6.62±4.20; 70mg: 7.21±4.56; placebo: 7.12±4.85) and PI-ID (140mg: 5.81±4.32; 70mg: 6.09±4.60; placebo: 6.20±5.05). Over the final 3 months of the DBTP, greater reductions from baseline in EA-IDs and PI-IDs were observed with erenumab 140mg and 70mg groups than placebo. For EA-IDs, subjects treated with erenumab 140mg (Least Squares [LS] mean=−3.01 days (95% confidence interval [CI]: −3.45,−2.57) and 70mg (−2.83 days [−3.27,−2.39]) experienced larger reductions compared with placebo (−1.71 [−2.16,−1.27], both p<0.001). Greater reductions in mean monthly PI-ID days were also observed in the erenumab groups (140mg: LS mean=−2.51 days (95% CI: −2.93,−2.09); 70mg: −2.25 days [−2.68,−1.83]) compared with placebo (−1.16 [−1.59,−0.74], both p<0.001).

**Conclusion**

Compared with placebo, EM subjects treated with erenumab 140mg and 70mg experienced greater reductions from baseline in mean monthly MPFID EA-ID and PI-ID during the 6 month DBTP. Numerically greater reductions were observed with 140mg than 70mg. Erenumab-treated patients experience reductions in functional impairment due to migraine, which complements improvements observed with standard efficacy measures.

### P100 Patient-reported outcomes in patients with chronic migraine receiving placebo or erenumab (AMG 334) in a phase 2, randomised, double-blind study

#### Richard B Lipton^1^, Stewart J Tepper^2^, Uwe Reuter^3^, Stephen Silberstein^4^, Walter Stewart^5^, Dean Leonardi^6^, Pooja Desai^6^, Sunfa Cheng^6^, Daniel D Mikol^6^, Robert A Lenz^6^

##### ^1^Department of Neurology, Albert Einstein College of Medicine, Bronx, New York, NY, USA; ^2^Geisel School of Medicine at Dartmouth, Hanover, NH, USA; ^3^Department of Neurology, Charité Universitätsmediz in Berlin, Berlin, Germany; ^4^Jefferson Headache Center, Thomas Jefferson University, Philadelphia, PA, USA; ^5^Sutter Health, Walnut creek, CA, USA; ^6^Amgen Inc., Thousand Oaks, CA, USA

###### **Correspondence:** Richard B Lipton (Richard.Lipton@einstein.yu.edu)

**Background**

Migraine is a disabling disease associated with substantial burden on patients and society. Erenumab (AMG334) is a fully human anti-calcitonin gene-related peptide (CGRP) receptor monoclonal antibody in clinical development for migraine prevention. The objective of this study was to evaluate patient-reported outcomes in a phase 2 clinical trial of erenumab for chronic migraine (CM) (NCT02066415).

**Methods**

667 adults with CM were randomised (3:2:2) to monthly subcutaneous placebo or erenumab 70 mg or 140 mg. Primary and secondary endpoints were assessed at Week 12. Exploratory endpoints included: change from baseline in migraine-specific quality of life (QoL) measured by the Migraine-Specific QoL Questionnaire (MSQ), headache impact measured by the Headache Impact Test (HIT-6), and migraine-related disability measured by the Migraine Disability Assessment Test (MIDAS). No formal hypothesis was tested; p-values (placebo vs erenumab dose-groups) are descriptive.

**Results**

Baseline scores were similar between groups. Improvements were observed for all endpoints in both erenumab groups at Week 12. The mean (95% CI) changes for placebo vs 70 mg and 140 mg groups, respectively, were 11.8 (9.4, 14.1) vs 17.7 (14.9, 20.6), p=0.002 and 19.1 (16.3, 22.0), p<0.001 for MSQ role function-restrictive scores, 8.9 (6.8, 11.0) vs 13.0 (10.5, 15.6), p=0.013 and 13.8 (11.3, 16.4), p=0.003 for MSQ role function-preventive scores, and 9.9 (7.3, 12.5) vs 18.2 (15.0, 21.3), p<0.001 and 18.8 (15.6, 21.9), p<0.001 for MSQ emotional-function scores. Mean changes in HIT-6 scores were −3.1 (−3.9, −2.3) for placebo vs −5.6 (−6.5, −4.6), p<0.001 for both erenumab groups. Corresponding mean changes in the placebo, 70 mg, and 140 mg dose-groups were −7.5 days (−12.4, −2.7) vs −19.4 days (−25.2, −13.6), p=0.002 and −19.8 days (−25.6, −14.0), p=0.001 for MIDAS days of lost productivity, −5.2 days (−8.0, −2.4) vs −10.3 days (−13.6, −6.9), p=0.023 and −10.2 days (−13.6, −6.8), p=0.024 for MIDAS-absenteeism, and −1.9 days (−4.7, 0.8) vs −9.3 days (−12.6, −6.1), p<0.001 and −9.9 days (−13.2, −6.7), p<0.001 for MIDAS-presenteeism.

**Conclusions**

Erenumab-treated CM patients experienced consistent and clinically significant improvements in patient-reported outcomes including migraine-specific QoL and reductions in headache impact and disability.

### P101 Treatment-Induced Improvement in Migraine Classification in the Fremanezumab HFEM Study

#### Robert Noble^1^, Ernesto Aycardi^2^, Marcelo Bigal^3^, Pippa Loupe^4^

##### ^1^Statistics, Teva Global Medical Affairs, Hamilton; ^2^Global Clinical Development; ^3^Clinical Development, Teva Global Research and Development, Frazer; ^4^Academic Affairs and Network, Teva Global Research and Development, Overland Park , United States

###### **Correspondence:** Robert Noble

**Objectives**

Fremanezumab is a fully humanized monoclonal antibody targeting the calcitonin-gene related peptide (CGRP) ligand, a validated target for migraine preventive therapy. Fremanezumab was found to be effective and well-tolerated as a preventive treatment for migraine in a high frequency episodic migraine (HFEM) phase 2 study. Study participants included patients classified as having episodic migraine (EM) per the ICHD III beta. Herein, we determined whether there was a treatment-induced shift in the number of patients who met the criteria for classification as having high frequency EM (HFEM) to moderate frequency episodic migraine (MFEM) and low frequency episodic migraine (LFEM) during the HFEM study.

**Methods**

Patients were randomized to receive either fremanezumab doses (225mg or 675mg) or placebo every 28 days for 12 weeks. Headache information was captured daily using an electronic headache diary. For the post-hoc analysis, the frequency of headache days (days of headaches lasting > 4 hours) and migraine days (days with headaches classified as migraine, probable migraine or treated with triptan or ergot compounds) per month were categorized into four types of migraine classification: Chronic migraine (CM) as having ≥15 headache days with 8 migraine days; HFEM 8 to 14 headache days with 8 migraine days; MFEM 4 to 7 headache days and 4-7 migraine days; LFEM 0 to 3 headache days and 0-3 migraine days. Analyses on the shifts for migraine classification from baseline to month 3 were performed to determine the percent of patients who showed improvement (HFEM to MFEM or LFEM), worsening (HFEM to CM) and those who remained classified as HFEM.

**Results**

Overall, the fremanezumab arms showed significant improvement in migraine classification compared to the placebo arm at month 3 (Patients on 225mg 73% vs 49% for placebo, 95% CI: 0.098 to 0.358 and patients on 675mg 71% vs 49% placebo, 95% CI: 0.079 to 0.341, Table 1A). Chi square analyses indicated that the shift of migraine classification during the study was not independent of treatment, X^2^ =31.64, p=1.91E-05. As shown in Table 1B, 45% and 52% of patients on fremanezumab 225mg and 675mg showed a shift in migraine category from HFEM to LFEM in 3 months compared to 20% of placebo patients.

**Conclusion**

As treated patients were more likely to improve and less likely to worsen compared to those on placebo, this study suggests that fremanezumab may potentially prevent the progression of migraine to more chronic forms.

**Table 1 (abstract P101).** Patient classification in migraine categories during the HFEM study

Table 1A. Overall shift in migraine category^a^	Placebo n = 104	Fremanezumab 225 mg n = 95	Fremanezumab 675 mg n = 96
Worsen	7 (7%)	2 (2%)	3 (3%)
Stable	42 (40%)	14 (15%)	17 (18%)
Improve	51 (49%)	69 (73%)	68 (71%)
Discontinued	4 (4%)	10 (11%)	8 (8%)
Table 1B: Migraine Categories Num (%) patients in 3^rd^ month	Placebo (n = 98)^b^	Fremanezumab 225 mg (n = 88)	Fremanezumab 675 mg (n = 91)
CM	7 (7%)	2 (2%)	2 (2%)
HFEM	41 (42%)	14 (16%)	17 (19%)
MFEM	28 (29%)	22 (25%)	21 (23%)
LFEM	20 (20%)	40 (45%)	47 (52%)

^a^Shift in migraine category, worsen = HFEM to CM, stable = HFEM to HFEM, improve = HFEM to MFEM or HFEM to LFEM, discontinued = left study ^b^n values indicate the patients per treatment group meeting HFEM classification at baseline

**Trial registration**

Clinicaltrials.gov NCT02025556

**Competing Interest**

Fremanezumab HFEM Study supported by Teva Pharmaceutical Industries Global Research and Development, Netanya Israel. RN, EA, MEB, and PL are employees of Teva Pharmaceutical Industries, Israel.

### P102 Analysis of blood pressure following short-term and long-term treatment with erenumab

#### Stewart J Tepper^1^, Julio Pascual^2^, Uwe Reuter^3^, Hernan Picard^4^, Frank Hong^5^, Marie-Louise Trotman^4^, Fei Xue^4^, Dan Mikol^4^, Jan Klatt^5^

##### ^1^Geisel School of Medicine at Dartmouth, Hanover, NH, USA; ^2^Service of Neurology, University Hospital Marqués de Valdecilla and IDIVAL, Santander, Spain; ^3^Department of Neurology, Charité Universitätsmedizin Berlin, Berlin, Germany; ^4^Amgen Inc., Thousand Oaks, CA, USA; ^5^Novartis Pharma AG, Basel, Switzerland

###### **Correspondence:** Stewart J Tepper

**Background**

Erenumab is a fully human monoclonal antibody that selectively targets and inhibits the calcitonin gene-related peptide (CGRP) receptor and is under investigation for migraine prevention. As the ligand CGRP is a vasodilator, inhibition of the CGRP pathway might theoretically increase blood pressure (BP). Addressing this theoretical risk through ancillary safety studies has been an important focus during the clinical development of erenumab. This report summarises BP findings from the erenumab clinical development programme.

**Methods**

Systolic and diastolic BP values were obtained at baseline and every 4 weeks in four placebo-controlled erenumab clinical trials (clinicaltrials.gov NCT01952574, NCT02066415/NCT02174861, NCT02456740, NCT02483585). Blood pressure was recorded as the average of ≥2 measurements taken ≥5 minutes apart with subjects lying supine and rested for ≥5 minutes. Pooled placebo-controlled data from baseline and treatment Weeks 4, 8, and 12 are presented. In categorical analyses, a notable increase in BP was defined as an increase from baseline of ≥10 mmHg in diastolic and/or ≥20 mmHg in systolic BP. In a separate Phase 1 study (NCT01723514), 24-hour continuous BP was assessed in healthy volunteers receiving erenumab monthly over 12 weeks.

**Results**

Blood pressure was similar across treatment groups at baseline, and remained stable throughout the 12-week period (Fig. 1), with no difference observed between erenumab and placebo. Mean (SD) change from baseline at 12 weeks in systolic BP was −0.4 (9.6), −0.5 (10.0), and −1.0 (9.8) mmHg, and mean change in diastolic BP was −0.6 (7.1), −0.3 (7.7), and −0.4 (7.2) mmHg, in subjects treated with placebo, erenumab 70 mg, and erenumab 140 mg, respectively. The proportion of subjects with a notable increase at any visit was similar across treatment groups. In a 24-hour continuous BP assessment, erenumab 70 mg and 140 mg did not increase BP and had no effect on diurnal rhythm (Fig. 2).

**Conclusion**

Selectively blocking the CGRP receptor with erenumab 70 and 140 mg had no relevant effect on BP in subjects with migraine over 12 weeks versus placebo, and was not associated with significant 24-hour BP changes in healthy volunteers.

### P103 The role of peripheral CGRP on the vasculature in a preclinical mouse model of migraine

#### Bianca Mason

The neuropeptide calcitonin gene-related peptide (CGRP) is a key player in migraine. While migraine can be induced by peripherally administered CGRP (intravenous) and can be treated using CGRP antagonists that act peripherally, the relevant sites of CGRP action remain unknown. To address the role of CGRP both within and outside the central nervous system, we used a mouse model of photophobia. Photophobia is an abnormal discomfort to non-noxious levels of light and is experienced by approximately 90% of migraine patients. We have previously shown that peripheral (intraperitoneal, IP) injection of CGRP resulted in light aversive behavior in wild-type CD1 mice similar to aversion previously seen following central (intracerebroventricular, ICV) injection. Importantly, two clinically effective migraine drugs, the 5-HT_1B/D_ agonist sumatriptan and a CGRP-blocking monoclonal antibody, attenuated the peripheral CGRP-induced light aversion and motility behaviors. To begin to address the mechanism of peripheral CGRP action, first we used transgenic CGRP-sensitized mice that have elevated levels of the CGRP receptor hRAMP1 subunit in nervous tissue (*nestin/hRAMP1*). Surprisingly, sensitivity to low light was not seen after IP CGRP injection, but was only seen after ICV CGRP injection. Next, we used transgenic CGRP-sensitized mice that have globally elevated levels of hRAMP1 (global *hRAMP1*) in all tissues. Interestingly, sensitivity to low light after IP CGRP in these mice was observed. These results suggest that CGRP can act in both the periphery and the brain by distinct mechanisms. This also suggests that peripheral CGRP actions may be transmitted to the CNS via indirect sensitization of peripheral nerves and likely not on CGRP receptors in the nervous system to cause migraine-like photophobia. We have now begun investigating the role of the vasculature in peripheral CGRP-induced light aversion and allodynic pain responses by using two approaches (1) injection of phenylephrine to minimize vasodilation induced by CGRP (2) genetic overexpression of the CGRP receptor in the vasculature. We have found that CGRP has actions on smooth muscle cells that may contribute to the induction of migraine-like photophobia and that vasodilation may also, in part, play a role in this migraine-like behavior.

### P104 Peripheral vagal nerve stimulation modulates the nociceptive withdrawal reflex in healthy subjects: a cross-over sham-controlled study

#### Roberto De Icco^1,2^, Daniele Martinelli^1,2^, Eric Liebler^3^, Marta Allena^2^, Vito Bitetto^1^, Grazia Sances^2^, Giorgio Sandrini^1,2^, Giuseppe Nappi^1,2^, Cristina Tassorelli^1,2^

##### ^1^Department of Brain and Behavioral Sciences, University of Pavia, 27100, Pavia, Italy; ^2^ Headache Science Center, C. Mondino National Neurological Institute, 27100, Pavia, Italy; ^3^ electroCore LLC, Basking Ridge, NJ, 07920, USA

###### **Correspondence:** Roberto De Icco (rob.deicco@gmail.com)

**Objectives**: Peripheral non-invasive vagal nerve stimulation (nVNS) has become a target for the treatment of primary headaches, though its exact mechanisms are unclear. Different studies showed that nVNS modulates both spinal and supra-spinal nociceptive pathways in an inhibitory direction. The nociceptive flexion reflex paradigm is widely used to investigate modulation of nociception and represents a reliable objective measure of the functional activation of the nociceptive network. The aim of our study is to evaluate the effect of nVNS on the nociceptive withdrawal reflex in healthy subjects.

**Materials and Methods**: We enrolled 10 healthy subjects (5 males, age 26.5±2.2 years) in a cross-over sham-controlled study. Subjects were randomly assigned to: 1) nVNS: one 120-s electrical stimulation on each side of the neck using the gammaCore device and b) Sham: one 120-s electrical stimulation of the median nerve on each wrist using the gammaCore device. Nociceptive withdrawal reflex was evaluated in the right lower limb according to a standardized paradigm: electrical stimulation delivered at the sural nerve and electromyographic muscular response recorded from the ipsilateral biceps femoris. The single stimulus reflex threshold (RT-SS) was the lowest intensity (mA) needed to elicit a stable muscular response. The temporal summation threshold (RT-TS) was evaluated using a train of 5 stimuli at 2 Hz.

**Results**: At T0 the neurophysiological parameters were comparable between groups. In particular RT-SS was 13.86±3.67 and 16.15±3.53 in nVNS and Sham groups, respectively (p=0.086), while RT-TS was 11.0±2.79 in nVNS group and 12.64±3.67 in Sham group (p=0.107).

nVNS caused a significant increase in the RT-SS at T5 (+14.5%±4.2, p=0.023) and T30 (+25.9%±6.6, p=0.011). Also RT-TS significantly increased following nVSN at T30 (+21.7±6.7, p=0.031). Sham stimulation did not induce any significant modification on the reflex parameters. When comparing groups, we found that the increase of RT-SS at T5 and T30 was significantly higher in nVNS vs. Sham (p=0.008 and p=0.007 respectively). The percentage increase of RT-TS at T30 was significantly higher in the nVNS vs. SHAM (p=0.013).

**Conclusion**: Using a reliable method, we demonstrated that nVNS induces a rapid onset analgesia in healthy subjects. Because of its neurophysiological signatures, this analgesic activity is likely related to the inhibition of pain facilitation mechanisms occurring at the spinal and/or supra-spinal level. The mechanistic bases of this activity are to be elucidated, but the present observation provides additional evidence for the role of nVNS in the acute and prophylactic treatment of primary headaches.

### P105 Non-invasive vagal nerve stimulation reduces provoked cranial parasympathetic output in healthy volunteers

#### Celina F. Schroeder*, Maike Moeller*, Arne May

##### Department of Systems Neuroscience, University Medical Center Eppendorf, Hamburg, 20246, Germany

*The authors contributed equally to this article.

**Background**

Cranial autonomic symptoms such as lacrimation and conjunctival injection are characteristic features in some primary headache disorders [1]. Recent studies proposed non-invasive vagal nerve stimulation (nVNS) as a novel treatment approach for migraine and cluster-headache patients [2-4]. However, nVNS seems to be more efficient when autonomic symptoms are present and the underlying mechanisms of action remain to be elucidated. Here, we aimed to investigate the effect of nVNS on provoked cranial autonomic symptoms (lacrimation) in healthy volunteers.

**Methods**

Healthy volunteers were recruited and participated in a single-blind, placebo-controlled within-subject design (Fig. 1). nVNS was performed using the gammaCore device (electrocore LLC, Basking Ridge, NJ, USA) and kinetic oscillation stimulation (KOS) (Chordate Medical AB, Stockholm, Sweden) of the nasal mucosa was used to generate quantifiable cranial parasympathetic output (lacrimation) [5]. Lacrimation was quantified using a Schirmer’s II test. Sham controlled stimulation was applied in a pseudorandomized order for 3x2 minutes after baseline-measurement. As an indicator of autonomic activation, the lacrimation difference between baseline and KOS after each of the three experimental conditions was calculated.

**Results**

KOS-induced lacrimation was significantly reduced after nVNS compared to sham stimulation and no stimulation. This effect was only observed on the ipsilateral but not the contralateral eye. During nVNS- treatment only small side effects, including facial muscle convulsion and tingling sensations were reported by the volunteers.

**Conclusion**

These data show a physiologically measurable effect of nVNS on the trigeminal autonomic reflex in humans, suggesting an interaction between the trigeminal and the vagal system. These results support previous findings that the vagal system modulates central structures of the trigeminal autonomic reflex [6] that play a crucial role in various primary headache disorders.

**Fig. 1 (abstract P121).**
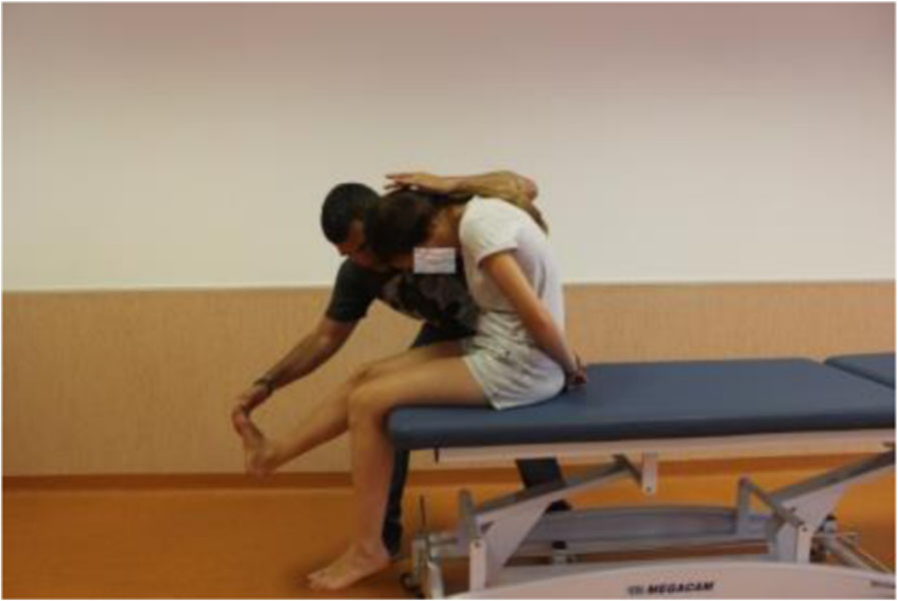
Slump test

**Acknowledgement**

This study was supported by an unrestricted scientific grant from Chordate medical.

**References**

1. Headache Classification Committee of the International Headache Society. The International Classification of Headache Disorders, 3rd edition (beta version). Cephalalgia. 2013;33(9):629-808.

2. Goadsby PJ, Grosberg BM, Mauskop A, Cady R, Simmons KA. Effect of noninvasive vagus nerve stimulation on acute migraine: an open-label pilot study. Cephalalgia. 2014;34(12):986-93.

3. Gaul C, Diener HC, Silver N, Magis D, Reuter U, Andersson A, et al. Non-invasive vagus nerve stimulation for PREVention and Acute treatment of chronic cluster headache (PREVA): A randomised controlled study. Cephalalgia. 2016;36(6):534-46.

4. Silberstein SD, Calhoun AH, Lipton RB, Grosberg B, Cady R, Dorlas S, et al. Chronic migraine headache prevention with noninvasive vagus nerve stimulation The EVENT study. Neurology. 2016;87:529-538.

5. Moeller M, Haji A, Hoffmann J, May A. Peripheral provocation of cranial autonomic symptoms is not sufficient to trigger cluster headache attacks. Cephalalgia. in press.

6. Akerman S, Simon B, Romero-Reyes M. Vagus nerve stimulation suppresses acute noxious activation of trigeminocervical neurons in animal models of primary headache. Neurobiol Dis. 2017;102:96-104.

### P106 Obesity is more prevalent in chronic migraine than in episodic migraine

#### Brad D Torphy^1^, Nicole Montes Buckley^2^

##### ^1^Diamond Headache Clinic, Chicago, IL, 60642, USA; ^2^Adler University, Chicago, IL, 60602, USA

###### **Correspondence:** Brad D Torphy

**BACKGROUND:** Migraine and obesity are two major public health problems in the USA and around the world. In the USA 12% of individuals have migraine and 37% of adults are obese^1^. Evidence suggests a possible link between migraine and obesity, but research has been mixed. There are several potential mechanisms for this link, including physiological, psychological, and behavioral.

**PURPOSE OF STUDY:** The purpose of our study was to determine if obesity is more prevalent in chronic migraine than in episodic migraine.

**RESULTS:** We analyzed the body mass index (BMI) at presentation of all new patients seen by the lead author in a tertiary headache center for a period of one year. The mean BMI of chronic migraine (CM) patients was 28.8% versus 25.3% for episodic migraine (EM) patients, both of which are defined as “overweight.” 34.4% of CM patients and 5.4% of EM patients were obese (BMI ≥ 30).

**CONCLUSIONS:** Obesity was prevalent in CM patients than in EM patients treated at a tertiary headache center. Furthermore, BMI was significantly higher in patients with CM than in EM patients.

**References**

1. Bigal M, Lipton R. The epidemiology, burden, and comorbidities of migraine. Neurol Clin. 2009; 27:321-34.

### P107 The Impact of Fremanezumab on Migraine-Specific Health-Related Quality of Life in Episodic Migraine

#### Richard B Lipton^1^; Sanjay K Gandhi^2^; Timothy Fitzgerald^2^; Paul P Yeung^2^; Joshua M Cohen^2^; Yuju Ma^2^; Ernesto Aycardi^2^

##### ^1^Albert Einstein College of Medicine, Bronx, New York, 10461, USA; ^2^Teva Pharmaceutical Industries, Frazer, Pennsylvania, 19355, USA

###### **Correspondence:** Richard B Lipton

**Background**

As the seventh most disabling condition globally, migraine adversely affects the health-related quality of life (HRQoL) of sufferers. In clinical trials, fremanezumab, a fully humanized monoclonal antibody that selectively targets calcitonin gene-related peptide, reduced the frequency of headaches and migraines in patients with episodic migraine (EM). For a comprehensive understanding of the impact of fremanezumab, this study compared HRQoL, as measured by the Migraine-Specific Quality of Life (MSQoL) questionnaire, in patients with EM who were treated with two dose regimens of fremanezumab versus placebo.

**Materials and Methods**

In this Phase III, multicenter, randomized, double-blind, placebo-controlled study, eligible patients with prospectively confirmed EM (headache on 6–14 days and ≥4 days fulfilling migraine criteria per month) were randomized 1:1:1 to receive subcutaneous injections of fremanezumab quarterly dosing (675 mg at baseline and placebo at Weeks 4 and 8), fremanezumab monthly dosing (225 mg at baseline, and Weeks 4 and 8), or placebo at each time point over a 12-week treatment period. The mean change from baseline (day 0) in HRQoL, using the MSQoL questionnaire, was evaluated as an exploratory endpoint. The MSQoL questionnaire (version 2.1) assessed three domains: the role function-restrictive (RFR), role function-preventive (RFP), and emotional function (EF) domains. Scores range from 0 to 100, with higher scores indicating better HRQoL. Statistical analyses were performed using an analysis of covariance approach and a mixed-effects model for repeated-measures, both with years since onset of migraine and baseline MSQoL domain score as covariates.

**Results**

A total of 865 patients were included in analyses (fremanezumab quarterly, N=288, fremanezumab monthly, N=287; placebo, N=290). Compared with placebo, the mean MSQoL RFR domain score for each fremanezumab dosing regimen was significantly increased from baseline to 4 weeks after the final dose of study drug (least-squares mean ± standard error for quarterly: 4.1±1.4; *P*=0.0028; monthly: 7±1.4, *P*<0.0001). The mean RFP domain scores were also significantly improved for both fremanezumab dose regimens compared with placebo (*P*≤0.001) as was the EF domain score for the fremanezumab monthly group (*P*<0.0001). Significant improvements in each domain of MSQoL were observed as early as 4 weeks after the first dose and was sustained at all pre-defined assessments (*P*<0.05).

**Conclusions**

These results indicate that fremanezumab improves the migraine-specific QoL and functioning of patients with EM.

**Trial Registration**

ClinicalTrials.gov NCT02629861

**Competing Interest**

**Richard B Lipton:** Consultant to Teva Pharmaceutical Industries.

**Sanjay K Gandhi:** Employee of Teva Pharmaceutical Industries.

**Timothy Fitzgerald**: Employee of Teva Pharmaceutical Industries.

**Paul P Yeung**: Employee of Teva Pharmaceutical Industries.

**Joshua M Cohen**: Employee of Teva Pharmaceutical Industries.

**Yuju Ma**: Employee of Teva Pharmaceutical Industries.

**Ernesto Aycardi**: Employee of Teva Pharmaceutical Industries.

### P108 The Impact of Fremanezumab on Headache-related Disability in Patients With Episodic Migraine Using the Migraine Disability Assessment

#### Paul K Winner^1^; Timothy Fitzgerald^2^; Sanjay K Gandhi^2^; Paul P Yeung^2^; Joshua M Cohen^2^; Yuju Ma^2^; Ernesto Aycardi^2^

##### ^1^Palm Beach Neurology, West Palm Beach, Florida, 33407, USA; ^2^Teva Pharmaceutical Industries, Frazer, Pennsylvania, 19355, USA

###### **Correspondence:** Paul K Winner

**Background**

Episodic migraine (EM), characterized by attacks on ≤15 days per month, is a risk for transition to chronic migraine and adversely affects daily functioning. In clinical trials, fremanezumab, a fully humanized monoclonal antibody that selectively targets calcitonin gene-related peptide, reduced the frequency of headaches in patients with EM. The impact of migraine can be more comprehensively understood through disability assessment. In this study, the Migraine Disability Assessment (MIDAS) questionnaire was used to assess the effect of fremanezumab versus placebo on headache-related disability.

**Materials and Methods**

In this multicenter, randomized, double-blind, placebo-controlled study, patients with EM were randomized 1:1:1 to receive subcutaneous injections of fremanezumab quarterly (675 mg at baseline and placebo at Weeks 4 and 8), fremanezumab monthly (225 mg at baseline, Weeks 4 and 8), or placebo at each time point over a 12-week treatment period. As a secondary endpoint, the change in MIDAS score was evaluated from baseline to 4 weeks after administration of the last dose of study drug. The MIDAS questionnaire assessed the lost days of activity in three domains (work, household work, and nonwork) over the previous 3 months. Scores of 0–5, 6–10, 11–20, and ≥21 were interpreted as disability grades 1 (little or no disability), 2 (mild), 3 (moderate) and 4 (severe). Efficacy analyses were performed in the full analysis set (FAS; all randomized patients who received at least one dose of study drug and had at least 10 days of post-baseline efficacy assessment on the primary endpoint) and repeated for the per-protocol analysis set (PPS). The data were analyzed using analysis of covariance, with baseline disability score and years since migraine onset as covariates. *P*-values for treatment comparisons were based on the Wilcoxon rank-sum test.

**Results**

A total of 875 patients were randomized to either fremanezumab quarterly (N=291), fremanezumab monthly (N=290) or placebo (N=294). The least-squares mean ± standard error changes from baseline with fremanezumab quarterly dosing (–23.0±1.60 points) and monthly dosing (–24.6±1.59 points) were larger than with placebo (–17.5±1.57 points); this resulted in significant treatment differences versus placebo in MIDAS score change for fremanezumab-treated patients (quarterly: –5.4±1.78 points, *P*=0.0023; monthly: –7.0±1.78 points, *P*<0.0001). Similar treatment differences were observed in the PPS (quarterly: –5.5±1.85 points, *P*=0.0030; monthly: –7.3±1.83 points, *P*<0.0001).

**Conclusions**

In this Phase III study, fremanezumab treatment demonstrated a significant improvement in headache-related disability in patients with EM.

**Trial Registration**

ClinicalTrials.gov NCT02629861

**Competing Interest**

**Paul K Winner** has been an investigator in clinical trials sponsored by Teva, Amgen, Genetech, Novartis, Allergan, AstraZeneca, Biogen Idec, and Ipsen. He has participated in advisory boards for Teva, Amgen, Avinar, Novartis, and Allergan, and has been on a speaker’s bureau for Allergan, Avinar and Teva.

**Timothy Fitzgerald**: Employee of Teva Pharmaceutical Industries.

**Sanjay K Gandhi:** Employee of Teva Pharmaceutical Industries.

**Paul P Yeung**: Employee of Teva Pharmaceutical Industries.

**Joshua M Cohen**: Employee of Teva Pharmaceutical Industries.

**Yuju Ma**: Employee of Teva Pharmaceutical Industries.

**Ernesto Aycardi**: Employee of Teva Pharmaceutical Industries.

### P109 The positive impact of fremanezumab on work productivity and activity impairment in patients with chronic migraine

#### Richard B Lipton^1^; Sanjay K Gandhi^2^; Timothy Fitzgerald^2^; Paul P Yeung^2^; Joshua M Cohen^2^; Yuju Ma^2^; Ernesto Aycardi^2^

##### ^1^Albert Einstein College of Medicine, Bronx, New York, 10461, USA; ^2^Teva Pharmaceutical Industries, Frazer, Pennsylvania, 19355, USA

###### **Correspondence:** Richard B Lipton

**Background**

Migraine is a debilitating disease with substantial indirect cost burden. In the EU, the total annual cost of migraine among persons aged 18–65 years was estimated at €111 billion. Mean per-person annual costs were €1222, with 93% of these costs attributed to indirect costs. Chronic migraine (CM) creates an especially high burden. In clinical trials, fremanezumab, a fully humanized monoclonal antibody that selectively targets calcitonin gene-related peptide, reduced the frequency, severity, and duration of headaches in patients with CM. This analysis evaluated the effect of fremanezumab on productivity loss and activity impairment in patients with CM, as measured by the Work Productivity and Activity Impairment (WPAI) questionnaire.

**Methods**

In this Phase III, multicenter, randomized, double-blind, placebo-controlled, parallel-group study, eligible patients with CM (≥15 headache days and ≥8 migraine days per month), were randomized 1:1:1 to receive subcutaneous injections of fremanezumab quarterly dosing (675 mg at baseline and placebo at Weeks 4 and 8), fremanezumab monthly dosing (675 mg at baseline and 225 mg at Weeks 4 and 8), or placebo at each time point over a 12-week treatment period. Change in WPAI score from baseline to 4 weeks after administration of the last dose of study drug was an exploratory endpoint. The WPAI questionnaire assesses the impact of health on the extent of work loss and productivity impairment during work and other activities, with higher scores indicating greater impairment.

**Results**

The study included 375 patients in each fremanezumab treatment group and 371 patients in the placebo group. Patients with CM treated with fremanezumab reported larger reductions from baseline in overall work productivity loss (composite of absenteeism and impairment while working [presenteeism]) than those given placebo (–16.6%±2.09% [quarterly] and –15.9%±2.02% [monthly] versus –9.1%±2.02% [placebo]), resulting in significant treatment differences for each fremanezumab group versus placebo (quarterly: –7.5%±2.24%, *P*=0.0009; monthly: –6.8%±2.26%, *P*=0.0026). The change from baseline in presenteeism was also greater with quarterly (–15.7%±1.89%) and monthly (–14.9%±1.82%) fremanezumab than with placebo (–10.0%±1.82%), resulting in significant treatment differences (quarterly: –5.7%±2.03%, *P*=0.0049; monthly: –4.9%±2.05%, *P*=0.0169). In addition, fremanezumab quarterly dosing significantly reduced impairment of activity outside of work versus placebo (–15.0%±1.70% versus –11.0%±1.7%; treatment difference: –4.0%±1.85%, *P*=0.0311).

**Conclusion**

In this Phase III study, fremanezumab treatment resulted in significant improvements in WPAI scores, demonstrating the positive impact of fremanezumab on the ability of patients with CM to function both at and outside of work.

**Trial registration**

ClinicalTrials.gov NCT02621931.

**Competing Interest**

**Richard B Lipton** is a consultant for Teva Pharmaceutical Industries.

**Sanjay K Gandhi:** Employee of Teva Pharmaceutical Industries.

**Timothy Fitzgerald**: Employee of Teva Pharmaceutical Industries.

**Paul P Yeung**: Employee of Teva Pharmaceutical Industries.

**Joshua M Cohen**: Employee of Teva Pharmaceutical Industries.

**Yuju Ma**: Employee of Teva Pharmaceutical Industries.

**Ernesto Aycardi**: Employee of Teva Pharmaceutical Industries.

### P110 Surface electromyografy of masticatory and neck muscles in patient with temporomandibular disorders and headeache: a case report

#### Novi S^1^, SaviL^2^

##### ^1^Gnathologist, freelance, Brunico –Italy; ^2^ Headeache Center- City of Healthe Science University Hospital-Torino- Italy Headeache Center LBS- Lugano-Switzerland

**Background:** Headeaches and temporomandibular disorders (TMD) are highly prevalent conditions that frequently co-exist in the same patient often associated whit myofascial pain of masticatory, pericranial and neck muscles and dental occlusion alterations.In order to determine the correct management and treatment, clinical examination should be coupled whit functional assessment. Surface electromyography(sEMG) can make an objective recording of the masticatory and neck muscles function and disfunction induced by dental occlusion as possible cause or aggravation factor of TMD associated with headeache before and after treatment.

**Case repor**t: Woman 53 years old affected by Chronic tension-type headeache ( according to ICHD3- beta criteria ) with pericranial tenderness and TMD whit myofascial long lasting pain, dental occlusion alteration, dual bite and bruxism, was submitted to sEMG. To verify the neuromuscolar equilibrium induced by dental contact, the standardized sEMG activities of right and left anterior temporal, masseter and sternocleidomastoidal (SCM) muscles were recorded during maximum voluntary clench.

Before treatment, sEMG data show an increased and more asymmetric standardized activity of their temporalis anterior muscles and anterior position of occlusal barycentre, presence of right mandibular torque ( precontact dental ), asymmetric activities of SCM with cervical load and dercreased muscle work values compatible with pain and muscolar fatigue probably as a consequence of nociceptive imputs. After one month with treatment combining drugs, stabilization appliance and couseling, there was the complete remission of muscle symptoms and a news sEmg objective the normalization of all indexes to confirm successful outcome of the therapy

**Conclusion**: sEMG, joins a set of clinical and morfological test, allow to object to any relationship between clinical symptom (pain related to muscles and palpation) and dental occlusion on masticatory and neck muscle activity in TDM associated with headeache.

**Consent for publication:** The authors declare that written informed consent was obtained for publication.

### P111 Headache secondary to subarachnoid haemorrhage due to arteriovenous malformation can be presented as migraine in its clinical phenotype

#### Srdjan Ljubisavljevic (srljub@gmail.com)

##### Faculty of Medicine, University of Nis, Nis, Serbia, Clinic for Neurology, Clinical Center Nis, Nis, Serbia

**Background:** The diagnosis and acute management of subarachnoid hemorrhage (SAH) is still representing a challenge to physicians who encounter these patients in everyday clinical practice. Headache is recognized as a cardinal symptom, although headache does not even occur in SAH patients. Although the severity and suddenness of onset is the most characteristic feature of headache secondary to SAH, little is known about other headaches attributes in reference to SAH origin and its pathogenesis.

**Methods**: The medical records of 431 consecutive non traumatic SAH patients (264 females and 167 males), ages from 19 to 91 years, presenting with headache (70.3%) and without headache (29.7%) during period of 11 years have been reviewed. Data have been analyzed in reference to headache attributes according to arteriovenous malformation (AVM) persistence.

**Results:** The persistence of AVM was not in association with headache occurrence (OR 0.71 [95%CI: 0.41-1.21], p=0.213), although the persistence of AVM were significantly associated with previous history of headache with migraine features, OR 1.74 [95%CI: 1.11-3.03], p=0.046. Also, the findings of AVM was significantly associated with the patients definition of headache intensity as the worst headache of life, OR 2.24 [95%CI: 1.29-3.89], p=0.004. Headache assigned symptoms such as vomiting/nausea and/or phono/photophobia were significantly frequent in SAH patients with AVM, OR 2.08 [95%CI: 1.13-3.83], p=0.018 and OR 1.75 [95%CI: 1.1-2.15], p=0.03, respectively. The positive association was observed in relation to AVM persistence and localized/unilateral head pain, OR 18.76 [95%CI: 9.68-36.37], p<0.0001. The association between AVM findings and headache features such as time from onset to peak of pain intensity, pain quality and state at time of headache onset in SAH were not observed, p>0.05.

**Conclusions:** From the clinical point of view, it could be said that the presence of AVM is not recognized as predictor for headache occurrence in spontaneous SAH but its existence could be associated with previous history of headache with migraine features. These facts could provide physicians to good evidence to consider SAH in patients with previous history of headache with migraine features newly presented with worst headache of their life, as well as consider cerebral angiography in all SAH suggestive headaches which are accompanied with migraine symptoms.

***Keywords:*** Migraine, Subarachnoid Hemorrhage

### P112 Decreased medial prefrontal cortex volume in migraine without aura: a multispectral MRI study

#### Sourena Soheili-Nezhad^1^, Alireza Sedghi^2^, Ferdinand Schweser^3,4^, Neda Jahanshad^1^, Paul Thompson^1^, Amir Eslami Shahr Babaki^5^, Aida Tabrizi^6^, Mansoure Togha^6^

##### ^1^ Imaging Genetics Center, USC Stevens Neuroimaging and Informatics Institute, Keck School of Medicine of USC, University of Southern California, Marina del Rey, CA 90292, United States of America; ^2^ Medical Informatics Laboratory, School of Computing, Queen’s University, Kingston, ON K7L2N8, Canada; ^3^ Department of Neurology, Buffalo Neuroimaging Analysis Center, Jacobs School of Medicine and Biomedical Sciences, University at Buffalo, Buffalo, NY 14203, United States of America; ^4^ Translational Imaging Center, Clinical and Translational Science Institute, University at Buffalo, Buffalo, NY 14203 United States of America; ^5^ Chronic Diseases Research Center, Endocrinology and Metabolism Population Sciences Institute, Tehran University of Medical Sciences, Tehran, Iran; ^6^ Iranian Center of Neurological Research, Neuroscience Research Institute, Tehran University of Medical Sciences, Tehran, Iran

###### **Correspondence:** Mansoure Togha (toghae@sina.tums.ac.ir)

**Background**

Migraine is a common and debilitating disease with a prevalence of 17.5% in women, which is more than two-fold of its prevalence in the male population [1]. Although migraine is classically considered an episodic disorder with no long-term sequels, new evidence suggests that at least a subset of patients may suffer progressive neuropathology and inter-ictal changes in brain function. Similar to various neurodevelopmental and neurodegenerative disorders, migraine is predisposed by a strong genetic component (>50% [2]). Quantitative neuroimaging of migraine has recently revealed disrupted functional brain networks and abnormal changes in brain structure, and discovering such neuroimaging biomarkers of migraine may enlighten disease mechanisms and guide future clinical trials.

**Materials and methods**

We enrolled 36 female migraine without-aura patients (36.6 ± 8.8 years) and 33 age-matched healthy subjects of the same sex (36.4 ± 8.8 years). Study subjects underwent MRI using a 3T Siemens Magnetom Trio scanner and a 12-channel head coil. Pulse sequences were as follows: 1) a 1mm isotropic T1-weighted MPRAGE anatomical scan 2) an 11-min resting-state fMRI scan with the parameters TR=2.57s and voxel size of 3.4×3.4×3.0mm. 3) DTI with 64 diffusion gradient directions and b-max=1000s/mm^2^ 4) an eight-echo gradient-echo pulse sequence with a voxel size of 0.8×0.8×1.5mm and further parameter optimization for quantitative susceptibility mapping (QSM). Changes in resting-state functional brain networks [3], volumetric differences in brain anatomy [4], and white-matter microstructure [5] were explored in order to characterize MRI correlates of migraine by using various statistical neuroimaging toolboxes including ANTS, FSL, MRtrix3 and in-house developed software. The HEIDI algorithm was used to quantify variations in regional brain tissue susceptibility [6].

**Results**

At the time of preparation of this abstract, tensor-based morphometry analysis has been completed and revealed significant loss of brain volume in right medial prefrontal cortex (mPFC) of migraine patients (corrected p<0.05, Fig. 1) and suggestive loss of volume in bilateral inferior frontal sulcus (uncorrected p=2.4×10^-5^). Resting functional networks including the default-mode network were decomposed from 17,250 preprocessed fMRI volumes of the study cohort in the common template space (Fig. 2). Preprocessed diffusion images revealed white-matter tracts and their crossing in study subjects (Fig. 3). Changes in brain tissue magnetization due to regional deposition of iron and other susceptibility-inducing materials were assessed by QSM (Fig. 4).

**Conclusions**

As one of the largest multispectral MRI studies of headache conducted to date, our results demonstrate promise of quantitative neuroimaging in exploring pathophysiology of migraine and identifying the dysfunctional pain network in patients.

### P113 Chronic paroxysmal hemicrania in paediatric age: report of four cases

#### Laura Papetti, Samuela Tarantino, Barbara Battan, Federico Vigevano, Massimiliano Valeriani

##### Headache Center, Department of Neuroscience, Bambino Gesù Children Hospital, Rome, Italy

###### **Correspondence:** Laura Papetti (laura.papetti@opbg.net)

**Objectives**. Chronic paroxysmal hemicrania (CPH) is a rare and well-characterised headache, classified amongst the trigeminal autonomic cephalgias (TACs). CPH has been only rarely and incompletely described in the developmental age. The objective of the present report was to describe the features of chronic paroxysmal migraine in four pediatric patients.

**Results**. We detected 4 patients with CPH. Our children presented with a long history of severe and unilateral pain, which occurred in the fronto-orbital region without side shift. Attacks were accompanied by at least one autonomic symptom, ipsilateral to pain. During the attacks, besides conjunctival injection, eyelid oedema and rhinorrhea and all children showed a dramatic response to indomethacin.

**Conclusions**. Clinical symptoms and pain characteristics of our children are similar to those found in typical adult CPH. However our patients showed some atypical features, not fully meeting the ICHD-III beta criteria. First, although the ICHD criteria require an attack frequency higher than 5 attacks per day, in our patients, the attack frequency was lower. Second, attack duration was variable in all our children, but in three of four children it was sometimes longer than 30 min, which represents the maximal duration for a CPH attack.If attack duration and frequency can make the diagnosis more difficult, especially in paediatric age, the absolute response to indomethacin represents the diagnostic key for CPH in both adults and children (indotest).

### P114 Early Onset of Action with Fremanezumab Versus Placebo for the Preventive Treatment of Episodic Migraine

#### Paul Yeung^1^, Ernesto Aycardi^1^, Marcelo Bigal^1^, Tricia Blankenbiller^1^, Melissa Grozinski-Wolff^1^, Ronghua Yang^1^, Yuju Ma^1^, Jan Brandes^2^

##### ^1^Teva Pharmaceutical Industries, Frazer, Pennsylvania, 19355, USA; ^2^Nashville Neuroscience Group, Nashville, TN 37203, USA

###### **Correspondence:** Paul Yeung

**Background:** Migraine prevention is intended to reduce the frequency, severity, and disability associated with migraine attacks, and faster onset of action could increase benefit to patients with migraine. Fremanezumab is a fully humanized monoclonal antibody targeting the calcitonin gene-related peptide (CGRP) ligand, a preventive treatment designed to specifically target a pathophysiologic mechanism of migraine. This analysis assesses the early onset of action of fremanezumab in the prevention of episodic migraine.

**Methods:** A 16-week, multicenter, randomized, double-blind, placebo-controlled, parallel-group study comparing the efficacy, safety, and tolerability of 2 subcutaneous dose regimens of fremanezumab and placebo (PBO) in adults with episodic migraine (EM). Patients assigned randomly to 1:1:1 ratio to 1 of 3 treatment groups: (1)monthly dosing of fremanezumab 225 mg at months 1, 2 and 3, (2)quarterly dosing: 675 mg of fremanuzemab at month 1, followed by placebo injections at months 2 and 3, and (3)monthly administration of matching placebo. The mean change from baseline in the monthly average number of migraine days were assessed using a mixed-effect model for repeated measures.

**Results:** Statistically significant reduction in the monthly average number of migraine days was experienced during the 12-week period after 1st dose for both fremanezumab dosing regimens [monthly (-3.7 days);quarterly (-3.4 days); p<0.0001] vs. placebo (-2.2 days), and during the 4 week period after 1st dose, for both dosing regimens (p<0.0001). Fremanezumab monthly dosing resulted in significant reduction in the weekly number of migraine days:

- At Week 1, (-0.9 days; p<0.0001) versus placebo (-0.3 days)
- At Week 2, (-0.8 days; p<0.0001) versus placebo (-0.3 days)
- At Week 3, (-0.9 days; p<0.0001) versus placebo (-0.4 days)
- At Week 4, (-0.9 days; p=0.0123) versus placebo (-0.6 days)

Fremanezumab quarterly dosing resulted in significant reduction in the weekly number of migraine days:

- At Week 1, (-0.8 days; p<0.0001) versus placebo (-0.3 days)
- At Week 2, (-0.8 days; p<0.0001) versus placebo (-0.3 days)
- At Week 3, (-0.8 days; p=0.0003) versus placebo (-0.4 days)
- At Week 4, (-0.8 days; p=0.0403) versus placebo (-0.6 days)

Posthoc analysis indicated that more patients reported no migraine days with fremanezumab monthly dosing (79.4%; p=0.0002) and fremanezumab quarterly dosing (79.2%; p=0.0004) versus placebo (66.6%) by the next day following first injection.

**Conclusion:** Onset of action with fremanezumab occurred rapidly for preventive treatment of episodic migraine. Significant improvement was maintained for both monthly and quarterly subcutaneous injections.

### P115 Efficacy and Safety of 2 Dose Regimens of Subcutaneous Administration of Fremanezumab (TEV-48125) Versus Placebo for the Preventive Treatment of Episodic Migraine

#### Ernesto Aycardi MD^1^, Marcelo Bigal MD^1^, PhD, Paul Yeung MD^1^, MPH, Tricia Blankenbiller^1^, Melissa Grozinski-Wolff^1^, Ronghua Yang PhD^1^, Yuju Ma MS, Stephen Silberstein MD^2^, Peter J. Goadsby MD PhD^3^, and David Dodick MD^4^

##### ^1^Teva Pharmaceutical Industries, Frazer, Pennsylvania, 19355, USA; ^2^Jefferson Headache Center, Philadelphia, Pennsylvania, 19107, USA; ^3^Mayo Clinic, Scottsdale, Arizona, 85259, USA; ^4^NIHR-Wellcome Trust King’s Clinical Research Facility, King’s College London, UK

###### **Correspondence:** Ernesto Aycardi

**Background:** Fremanezumab, a fully humanized monoclonal antibody targeting the calcitonin gene-related peptide (CGRP) ligand, is a preventive treatment designed to specifically target a pathophysiologic mechanism of migraine; has proven efficacy in the treatment of migraine. This study evaluated the efficacy, tolerability and safety of two subcutaneous dose regimens of fremanezumab in the preventive treatment of episodic migraine (EM).

**Methods:** 16-week, multicenter, randomized, double-blind, placebo-controlled, parallel-group study in adults with EM. Patients maintained daily diaries during a 28-day baseline period. Patients were assigned randomly, 1:1:1 ratio to 1 of 3 treatment groups: monthly dosing - 225 mg of fremanezumab; quarterly dosing: a single dose of 675 mg of fremanuzemab at month 1, followed by placebo injections at months 2 and 3; and monthly administration of matching placebo. The primary efficacy endpoint, mean change from baseline (28-day pre-treatment period) to 12-week randomization period in monthly average number of migraine days was analyzed using an analysis of covariance method or the Wilcoxon rank sum test.

**Results:** The mean number of migraine days was 9.1 days during the 28-day baseline period. Patients treated with fremanezumab had significant reductions in the number of monthly migraine days during the 12-week period vs. placebo (-2.2 days from baseline=9.1 days), for both dosing regimens [monthly -3.7 days from baseline=9.2 days) and quarterly (-3.4 days from baseline=8.9 days); p<0.0001], and during the 4-week period after 1st dose, for both dosing regimens (p<0.0001). Fremanezumab-treated patients had significant reductions in the number of monthly headache days of at least moderate severity during the 12-week period for both dosing regimens [monthly (-2.9 days); quarterly (-3.0 days); vs placebo (-1.5 days); p<0.0001)], and during the 4-week period after 1st dose, for both dosing regimens (p<0.0001). Treatment with fremanezumab resulted in statistically significant reductions in the number of monthly days of acute headache medication use for both [monthly (-3.0 days); quarterly (-2.9 days); p<0.0001] versus placebo (-1.6 days). A ≥50% reduction in the monthly average number of migraine days was significantly improved with both dosing regimens [monthly (47.7%); quarterly (44.4%); p<0.0001] as compared to placebo (27.9%). The Migraine Disability Assessment (MIDAS) Scale showed an improvement with disability with monthly (-24.6; p=0.0021) and quarterly (-23.0; p=0.0023) dosing regimens compared to placebo (-17.5). The most common adverse events were injection site reactions.

**Conclusion:** These results confirm fremanezumab’s rapid efficacy, safety, tolerability, and flexible dosing profile, for the preventive treatment of episodic migraine.

**Trial Registration:**

ClinicalTrials.gov identifier: NCT02629861.

### P116 Efficacy and Safety of 2 Dose Regimens of Subcutaneous Fremanezumab Versus Placebo for the Preventive Treatment of Chronic Migraine

#### Ernesto Aycardi MD^1^, Marcelo Bigal MD^1^, PhD, Paul Yeung MD^1^, MPH, Tricia Blankenbiller^1^, Melissa Grozinski-Wolff^1^, Ronghua Yang PhD^1^, Yuju Ma MS, Stephen Silberstein MD^2^, Peter J. Goadsby MD PhD^3^, and David Dodick MD^4^

##### ^1^Teva Pharmaceutical Industries, Frazer, Pennsylvania, 19355, USA; ^2^Jefferson Headache Center, Philadelphia, Pennsylvania, 19107, USA; ^3^Mayo Clinic, Scottsdale, Arizona, 85259, USA; ^4^NIHR-Wellcome Trust King’s Clinical Research Facility, King’s College London, UK

###### **Correspondence:** Ernesto Aycardi

**Background:** Fremanezumab, a fully-humanized monoclonal antibody targeting the calcitonin gene-related peptide (CGRP) ligand, is a preventive treatment designed to specifically target a pathophysiologic mechanism of migraine; has proven efficacy in the treatment of migraine. This study evaluated the efficacy, tolerability and safety of two subcutaneous dose regimens of fremanezumab in the preventive treatment of chronic migraine (CM).

**Methods:** 16-week, multicenter, randomized double-blind, placebo-controlled, parallel-group adult CM study. Patients were assigned randomly (1:1:1 ratio to 1 of 3 treatment groups): monthly dosing=675 mg fremanuzemab followed by 225 mg of fremanezumab at months 2 and 3; quarterly dosing=fremanezumab 675 mg at month 1, followed by placebo injections at months 2 and 3; and monthly administration of matching placebo. The primary efficacy endpoint, mean change from baseline (28-day pre-treatment period) to 12-week randomization period in monthly average number of headache days was analyzed using an analysis of covariance method or the Wilcoxon rank sum test.

**Results:** The mean number of headache days of at least moderate severity was 13.1 days during the 28-day baseline period. Fremanezumab treated patients had significant reductions in the number of monthly headache days of at least moderate severity vs. placebo (-2.5 days) during the 12-week period after 1st dose, for both dosing regimens [monthly (-4.6 days; p<0.0001); quarterly(-4.3 days; p<0.0001). Patients treated with fremanezumab had statistically significant reductions in the number of monthly migraine days during the 12-week period after the 1st dose, for both dosing regimens [monthly (-5.0 days from baseline=16.0 days); quarterly (-4.9 days from baseline=16.2 days; p<0.0001)] vs. placebo (-3.2 days from baseline=16.3 days), and during the 4-week period after 1st dose, for both dosing regimens (p<0.0001). Fremanezumab was significantly superior to placebo for the prespecified secondary endpoints: reduction in the number of monthly days of acute headache medication use for both monthly (-4.2 days) and quarterly (-3.7 days) versus placebo (-1.9 days); p<0.0001). A ≥50% reduction in the monthly average number of headache days of at least moderate severity were also statistically significantly improved with both dosing regimens [monthly (40.8%); quarterly (37.6%); p<0.0001] as compared to placebo (18.1%); disability improved as measured by the Headache Impact Test with both dosing regimens [monthly (-6.8; p<0.0001); quarterly (-6.4; p=0.0001)] as compared to placebo (-4.5). The most common adverse event in the study was injection site reaction.

**Conclusion:** These results confirm fremanezumab’s rapid efficacy, safety, tolerability, and flexible dosing profile, for the preventive treatment of chronic migraine.

**Trial registration**

ClinicalTrials.gov NCT02621931

### P117 Influence of pre-treatment pain intensity on usage pattern, effectiveness and safety of Aspirin®: Subgroup analysis of six pharmacy-based surveys with 6.985 patients with headache or migraine

#### Marius Dörr, Uwe Gessner, Christoph Theurer

##### Scientific Affairs Consumer Health, Bayer Vital GmbH, Leverkusen, Germany

###### **Correspondence:** Marius Dörr

**Background**

Headache or other pain states are one of the most frequent reasons to use analgesics like Aspirin for self-treatment. Pre-treatment pain intensity may influence usage pattern, effectiveness and tolerability. The aim was to analyze real-life data from six pharmacy-based surveys in the self-treatment of headache or migraine with Aspirin.

**Materials and methods**

The individual data of 11.824 patients from seven pharmacy-based surveys performed in Germany, Switzerland and Spain were pooled. A subgroup analysis of six studies in patients with headache/migraine and information on pre-treatment pain intensity was performed (n=6.985); this subgroup consists of 687 patients with mild pain at baseline, 3.800 with moderate and 2.498 with severe pain. Patients who purchased the drug in a pharmacy were provided with a questionnaire to be filled in at home during/after treatment.

**Results**

Dose at first application was dependent from pain intensity at baseline: 77.2% with mild complaints, 72.0% with moderate and 50.8% with severe pain intensity used only one tablet at first intake. Frequency of drug intake was influenced by baseline pain severity: Single-dose treatment was reported by 62.9% of the patients with mild pain but by only 38.7% with severe pain. Accordingly, total dose, amount of tablets taken per day and treatment duration increased dependent on baseline pain intensity.

Pre-treatment pain intensity influenced also the duration of analgesia reported by the patients; an analgesic effect lasting more than 6 hours was reported by 46.8%, 35.7% and 26.3% of the patients with mild, moderate or severe pain. The percentage of patients being pain free two hours after start of treatment decreased substantially depending on pain intensity at baseline (88.5%, 66.1% and 42.4% of patients with mild, moderate or severe baseline pain). Already 30 minutes after first intake 63.7% of the patients with mild baseline pain intensity (moderate 57.1%; severe 46.3%) reported ≥ 50% on visual analogue scale compared to baseline.

The incidence of adverse events correlated with the pain intensity: Patients with severe pain at baseline reported more than twice as much adverse events than those with mild pain (11.7% vs. 5.5%).

**Conclusions**

Pre-treatment pain intensity influenced usage pattern of Aspirin in the self-medication of headache and other pain states. Stronger baseline-pain leads to higher daily dose and a longer duration of treatment. Effectiveness of the drug was also affected by pain intensity. Adverse event reporting was higher in patients with severe pre-treatment pain.

### P118 Botox is a safe and efficient treatment for cervicogenic headache

#### Monica Drottning ^1^, Sebastian Drottning^2^, Steffen Grønneberg^3^, Per Skjelbred^1^

##### ^1^Department of maxillary surgery, Oslo University Hospital, Ullevaal, University of Oslo, Oslo, 0407, Norway; ^2^Division of Paediatric and Adolecent Medicine, Oslo University Hospital, Rikshospitalet, Oslo 0424, Norway; ^3^BI Norwegian Business School, N-0442 Oslo, Norway

###### **Correspondence:** Monica Drottning

**Background**

Cervicogenic headache originates from neck pathology, but it is yet to discover the pathological substrate for the headache. Botulinum toxin type A is the treatment of choice for most forms of focal dystonia, including blepharospasm, torticollis, spasmodic dystonia and jaw dystonia. The efficacy of Botox has been established in adults with chronic migraine [1,2]. The injection procedure is superficial and does not address the main target of the botulinumtoxinA which is the muscles. Thus, it should be an efficient treatment for cervicogenic headache.

**Materials and methods**

35 patients with cervicogenic headache were included in a double blind, randomised placebo controlled study, cross over design. Each patient filled in a questionnaire concerning symptoms, sick leave, the social impact of symptoms, depression, post traumatic stress and head and full body pain drawings. The clinical examination included a relevant neurological examination, provocative tests such as Spurling’s test, stretching of neck muscles to elicit pain, identification of tender points of greater and minor occipital nerves with or without radiation to forehead, and measurements of passive neck mobility. Botox, total of 100 iu, or Placebo, was injected in defined muscular points in the neck at 0 months, then cross over at 2 months. The frequency, duration and intensity of headache and neck pain were registered before and after treatment.

**Results**

Of the 35 included patients three were excluded after entry due to encephalitis, MS and cerebral tumour. There were three drop outs. Seventy percent of the patients had a significantly reduced headache intensity after Botox injections regardless of whether the active substance was given at the first or second visit. For the remaining, but one, the headache intensity was unchanged. This was clinically significant as the majority of the patients labelled their headache moderate to mild after treatment opposed to strong before treatment. There were no serious adverse events.

**Conclusions**

Botulinum toxin type A (Botox) has been shown to reduce cervicogenic headache significantly. The treatment improves the quality of life and lifts the burden of headache in a degree that enables the persons to engage in work and society again. It is a simple procedure well accepted by the patients.

**Trial registration**

EudraCT number 2006-006700-11

**References**

1. Aurora SK, Winner P, Freeman MC. OnabotolinumtoxinA for treatment of chronic migraine: Pooled Analyses of the 56-Week PREEMT Clinical Program. Headache. 2011;51:1358-73.

2. Lipton RB, Varon SF, Grosberg B. OnabotolinumtoxinA improves quality of life and reduces impact of chronic migraine. Neurology 2011;77:1465-72.

### P119 Impaired cholinergic transmission during the migraine cycle: a short-latency afferent inhibition (SAI) study

#### Davide Di Lenola^1^, Gianluca Coppola^2^, Francesca Cortese^2^, Cherubino Di Lorenzo^3^, Francesco Pierelli^3^

##### ^1^Sapienza University of Rome Polo Pontino, Department of medico-surgical sciences and biotechnologies, Latina, Italy; ^2^G.B. Bietti Foundation IRCCS, Department of Neurophysiology of Vision and Neurophthalmology, Rome, Italy; ^3^Don Gnocchi foundation-IRCCS, Milan, Italy

###### **Correspondence:** Davide Di Lenola

**Background**

Short-latency afferent inhibition (SAI) is a form of inhibition related to the cholinergic activity in the cerebral cortex. It consists in a galvanic stimulation of the median nerve at the wrist that can suppress motor cortex excitability, as tested by transcranial magnetic stimulation (TMS), when given at a short interstimulus interval (ISI) between 18 and 21 ms. SAI is considered an in-vivo way to study the sensorimotor integration mechanisms. SAI response is influenced by the excitatory effect of acetylcholinergic thalamocortical afferents on the inhibitory GABAergic (mostly GABAa) cortical networks.

**Materials and methods**

We recruited 30 migraine without aura patients (16 between [MO] and 14 during [MI] attacks), and we compared them to a group of 16 healthy volunteers (HV). We first recorded somatosensory evoked potentials N20 latency and N20-P25 peak-to-peak amplitude at the contralateral parietal area. Afterward, SAI was recorded in all study’s participants as follows: after a conditioning single pulse delivered on the median nerve at the wrist, a TMS pulse was delivered with ISIs derived from the latency of N20 plus 2 to 8 ms in steps of 2ms and in random order. Five stimuli were delivered at each ISI. We calculated the SAI slope of the linear regression between the unconditioned motor evoked potential (MEP) amplitude and the 4-conditioned MEPs as a measure of cortical excitability.

**Results**

Compared with HV, SAI was significantly reduced in MO, but enhanced in MI patients (slope HV= +11.2, MO= +242, MI= -129). In both HV and MO groups, but not in MI, the SAI slope positively correlated with the SSEP N20-P25.

**Conclusions**

The reduction of SAI in MO patients and its enhancement in MI patients suggests a decrease and an increase respectively in facilitatory thalamocortical cholinergic activity on GABAergic network activity in the motor cortex. Since from the correlation analysis emerges that slope of SAI normally correlates with the parietal response in MO, but not in MI, we argue that more dysfunctional sensorimotor integrative mechanisms might characterize migraineurs during an attack.

### P120 BRAIN HERNIATION INTO TRANSVERSAL VENOUS SINUS: Case Report

#### Miodrag Vrcakovski^1,2^; Metodi Cepreganov^1,2^; Marija Milanovska^1,2^

##### ^1^Polyclinic NEUROMEDICA, Skopje, Macedonia (FYR Macedonia); ^2^Department of neurology and neuroradiology

**Introduction**

Brain herniations into transversal venous sinus are rare conditions.

In most of the cases are asymptomatic or they present with headaches or seizures if the herniation occurs in some of the epileptogenic regions in the brain.

In our case, we present a 32- years old female, with a history of severe headaches, during the last two years. The headaches have always been occurring on the left side, starting mainly during the night and while sleeping, with throbbing pain.

Sometimes headeaches have been so severe, ending up with loss of consciousness in period of 10 to 20 seconds.

MRI- examination of the brain performed prior in other diagnostic center showed a large filling defect in the left transversal sinus. The conclusion was clot caused by transverse sinus thrombosis. Anticoagulation therapy was started.

One year before the symptoms occur, patient started taking contraceptive pills. The conclusion of the radiologist was, that it is a condition after transverse sinus thrombosis, with bilateral local thrombotic masses in transversal sinus.

The patient came in our clinic to make new MRI- imaging of the brain.

The MRI- imaging was made with 1. 5 T Magnetom Essenza Siemens scanner.

During the MRI- examination, we used standard T2, T1 and FLAIR pulse sequences with high resolution, combined with additional and specially designed curves for reconstruction.

While processing the MRI- data, we used following pulse sequences:

T2 blade transversal ( Flair )

T2 TSE transversal

T1 SE transversal

T1MPR p2 iso 3D sagital ( gradient echo )

T2 SPC ns coronal iso ( gradient echo )

T2 3D ciss axial ( high resolution )

Ep 2D diffusion

MPR curved range

**MRI- findings**

Transversal T2, T1 and FLAIR pulse sequences showed presence of clearly demarked supstrat in right transversal sinus, 8 mm in diameter with signal characteristics similar with those of the normal brain tissue in all pulse sequences.

Arround the supstrat, there is well demarked area with signal characteristic similar with those of the liquor, wich in T2 pulse sequence, showed two tubular formations with void fenomen.

Lumen of the right transversal sinus showed few smaller defects, with signal characteristics in all pulse sequences identical with those of the liquor.

3D scans with high resolution showed that the supstratein left transversal sinus has the same characteristics with the normal brain parenchyma. Grey and white matter are clearly distigushed one from another, and near by is clearly demarked space with signal characteristics identical with those of the liquor.

Tortuouse structures are also well shown in this scans, and there is no doubt they are blood vessels. Those blood vessels indicate brain herniation, but not sinus thrombosis.

Three mounts before the patient came in our clinic, postcontrast MRI- series were made. They confirmed the findings of brain herniation into transversal sinus. On postcontrast series, the sinus is literally “shining” after the application of the contrast, and we can easily see the defect, with all components of brain herniation. Unfortunately, this finding was misdiagnosed as sinus vein thrombosis.

Thrombs have different signal characteristics, compared to the signal characteristics of brain herniation in vein sinus. They are solid and hypersignal in T1, hyposignal in T2 and they can’t have identical signal characteristics like those of the brain tissue nad cerebrospinal fluid. They don’t damage the dura and the wall of the vein sinus.

Around the thromb there is no leptomeningeal space, nor blood vessels or void phenomen.

Image 6 shows postcontrast series of right transversal sinus. The image shows enlarged postcontrast signal, but little supstrates in the lumen don,t intensify the signal, because the changes in all postcontrast series have signal characteristics identical with the signal characteristics of cerebrospinal fluid.

The question is: “How is that possible”?

The answer is down bellow:

**Discussion**

Brain herniation in dural vein sinus remains enigma.

How is it possible for defect to be made in the wall of the vein sinus? Is it congenital or posttraumatic?

The idea of congenital etiology could be accepted. This kind of changes could be listed in the group of meningoencephalocoeles, congenital conditions generated as a result of bone defect of cranial base.Through the defect, meninges and part of brain tissue exit in the surrounding area.

This idea is quite acceptable, but the crucial questionis: “Why the herniation occurs exactly in the dural sinus?” It is not about a bone defect, but about the defect in the wall of the vein sinus.

Another not logical thing in our case is, if the problem is congenital, why the first symptoms appeared two years ago, at the age of 30?

This leads us to logical explanation, that the problem is not congenital, but it appeared several years ago.

The question is why and how, and wich condition could made defect in the wall of the vein sinus?

Yellow arrows in Fig. 8 show polipoid anataomical structures, called arachnoidal granulations.

They are very thiny and small, almost not visible for human eye and they are responsible for transferring the cerebrospinal fluid from brain convexity into dural vein sinuses.

Arachnoidal granulations act like some kind of valves, providing the transfer of the cerebrospinal fluid.

During life, they can enlarge into gigant formations, expanding the wall of the vein sinus, enabling the brain tissue to herniate into the vein sinus.

Figure 11 shows the mechanism of brain herniation into the left transversal vein sinus in our case.

The final conclusion is that brain herniation into dural vein sinus is herniation of the brain tissue into gigant arachnoidal granulation.

Why arachnoidal granulations grow and hypertrophy?

Arachnoidal granulations grow and hypertrophy probably as a result of increasing the pressure and the volume of the cerebrospinal fluid.

Average cerebrospinal fluid pressure is about 10 mmHg.

Pressure in dural vein sinuses is about 0 mmHg. The difference between the pressure of cerebrospinal fluid and pressure in dural vein sinuses enables the cerebrospinal fluid to enter into vein system.

Increased cerebrospinal fluid pressure make the arachnoidal granulations to dilatate, grow and hypertrophy, wich enlarge the dural holes to the degree when brain tissue can herniate through them.

Figure 12 clearly shows that arachnoidal granulations can cause bone defects of calvary, as a result of penetration of arachnoidal granulations in the calvary.

**Conclussion**

1. Brain herniation in dural vein sinus is rare condition.

2. In most of the cases is asymptomatic.

3. Sometimes ( as in our case ) herniation of brain tissue in dural vein sinus is accompanied with headaches and seizures.

4. We have to consider theat defects in the lumen of dural vein sinus can occur as a result of thorombosis of the vein sinus or tumor invasion.

5. Hypertrophic arachnoidal granulation can also cause defects in the lumen of the dural vein sinuses, but their clinical significance is small.

6. Mr- imaging enables us to make precise diagnose, but it is necessary during the procedure to apply 3D pulse sequences with high resolutionas well as special designed curves for reconstruction.

7. Standard T1, T2 and FLAIR pulse sequences are not enogh for final evaluation and examination of *brain herniations in dural vein sinuses. 3D, T1 and T2 echo pulse* sequences with high resolution are necessary, as in our case where we gain useful informations using making reconstructions with special curves. They enabled excellent visualization of the herniated brain tissueand surrounding cerebrospinal fluid spaces with concomitant vascular structures.

8. Herniated brain tissue and surrounding cerebrospinal fluid spaces have normal signal characteristics in all pulse sequences.

### P121 Comparative study of mechanosensitivity and neural mobility in subjects with episodic tension type headache versus control group

#### Leandro H Caamaño^1,2^, Ricardo Ortega^3^, Fernando Galán^3^

##### ^1^International PhD School. Health Sciences, King Juan Carlos University, Madrid, 28922, Spain; ^2^Department of Physical Therapy, Gimbernat University School, Cantabria, 39316, Spain; ^3^Department of Physical Therapy, Occupational Therapy, Rehabilitation and Physical Medicine, King Juan Carlos University, Madrid, 28922, Spain

###### **Correspondence:** Leandro H Caamaño (leandro.caamano@eug.es)

**Background**

Headache is one of the most prevalence of neurological pathologies, being tensional type headache (TTH) the most prevalent of them. Diagnostic criteria of this primary headache were described in the International headache classification 3ird edition [1,2].

Its etiopathology has been related, among others, to the presence of myofascial trigger points in the craneo-cervical muscles, as well as phenomena related to its innervation, through which harmless signals are perceived as painful at a peripheral (peripheral sensitization) and central level, as well as an inhibition of pain control pathways (central sensitization) [3,4].

From this perspective, there are structures such as neural tissue, which have similar characteristics with the craneo-cervical muscles, such as innervation based on polymodal receptors, able to send tension signals and modify this type of information in the presence of a high tension to a nociceptive afferent, in addition to sharing a target area of these afferents, the trigeminal-cervical nucleus. It is for this reason that the meningeal tissue and specifically the dura mater could have a role in this type of headache [5,6].

The objectives of this study are: to identify if there is an alteration in neural sensitivity in subjects with tension-type headache, to identify if there is an alteration in neural mobility in subjects with tension-type headache, to establish a possible correlation between the range of motion and the perception of neural pain.

**Methods**

The sample was 40 women (n=40; age 20.4±1.9; 22.5±5.7), divided into two groups: tension headache (case group) and healthy group (control group).Inclusion criteria were: presenting TTH or not. Exclusion criteria were: to present another type of headache, to have suffered a traffic accident or previous vertebral trauma, to suffer back or neck pain for two or more consecutive days, to suffer pathologies or surgical interventions that may influence mechanical neural sensitivity, hip flexion lower than 70° degrees and psychological disorders such as anxiety or depression.

Neural provocation tests or neurodynamic tests were used to evaluate changes in neural tissue. Slump test: assessment of knee extension range and pain intensity (Fig. 1); Straight leg raise test: evaluation of the range of motion in flexion of the hip in two different positions, using an ankle orthosis to ensure plantar flexion of 0° and 30°. In these positions, two moments are evaluated: the Onset 1 (O2) as minimal perceptible pain and the Onset 2 (O2) as the maximum pain point perceptible (Fig. 2); Long seated slump Test: evaluation of sacral flexion using an apparatus designed to determine this position and cervical range of motion using a C-ROM and pain intensity (Fig. 3).

**Results**

Statistical analysis was performed using the spss 22.0 package. T-Student and U-Mann Withney tests were applied in order to compare means. Pearson's correlation was applied to assess the existence of linear correlation.

In Slump test, degrees of knee extension for cases and controls in the dominant leg were 53.75±13.06 and 63.75±7.68, p <0.05. The intensity of pain, through the NPRS scale was 6.2±1.7 and 5.4±2, p =0.176.In straight leg raise test, values for case group and control in the dominant leg were: 30° plantar flexion position 50.75±15.32 and 59.70±13.87, p =0.06 for onset 1 (O1) and 71.40±17.54 and 86.05±17.12 for onset 2 (O2) p<0.05.In 0° plantar flexion position, values for case and control group were 44.75±16.71 and 53.25±14, p =0.089 for onset1 (O1); 60.30±16.96 and 74.55±19.30, p <0.05 onset 2 (O2), respectively. The Pearson’s correlation coefficients for case group were: 30° plantar flexion position r = 0.53, p<0.05 (Fig. 4). 0° plantar flexion position r=0.52, p< 0.05(Fig. 5). Control group obtained values p>0.05. for both linear correlation tests .In long setaed slump test, values of sacral flexion degrees were 84.10±8.30 and 85,60±7.80, p> 0,05. Cervical flexion range of motion was 76.60±16.63, and 86.85±13.65, p<0.05, cases and control, respectively. The intensity of pain assessed by NPRS scales was 6.7±2.2 and 5.6±2.2, p=0.065.

**Conclusions**

Results observed through the application of neural tension maneuvers show changes in neural movement in the episodic tension type headache group versus control, being evaluated by range of motion and pain scales. All neural maneuvers show a decrease in ranges of motion evaluated: knee extension, hip flexion and cervical flexion; except sacral flexion. The amplitude of hip flexion degrees at 30 and 0 ° plantar flexion and pain are correlated in moderate and positive way, in case group. The assessment of pain intensity showed similar values in both groups

**Consent for publication:** The authors declare that written informed consent was obtained for publication.

**Fig. 2 (abstract P121).**
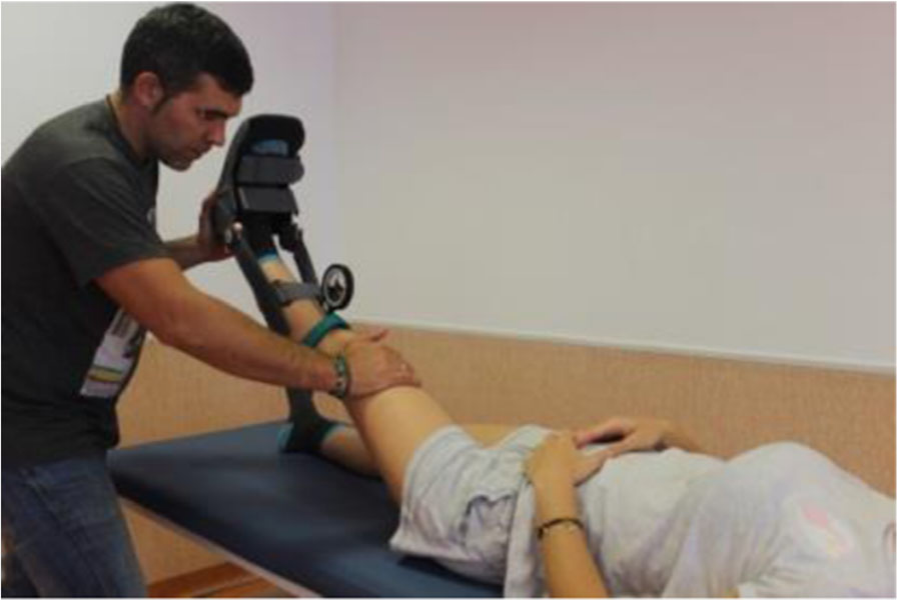
Straight leg raise test

**Fig. 3 (abstract P121).**
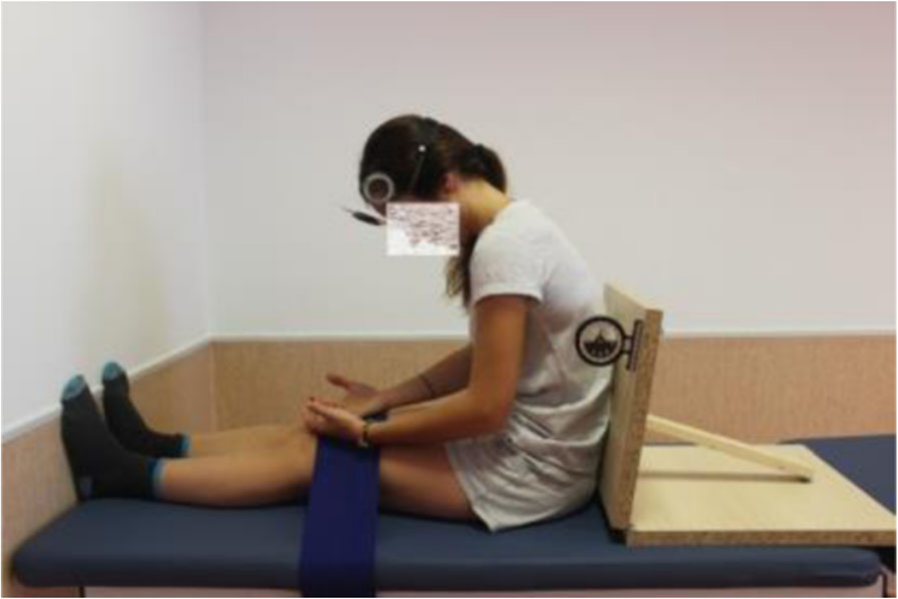
Long seated slump test

**Fig. 4 (abstract P121).**
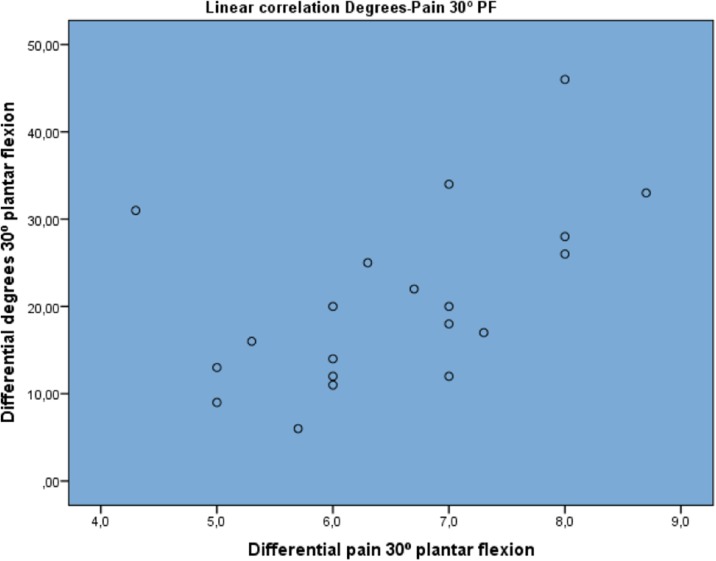
Pearson’s correlation 30° plantar flexion

**Fig. 5 (abstract P121).**
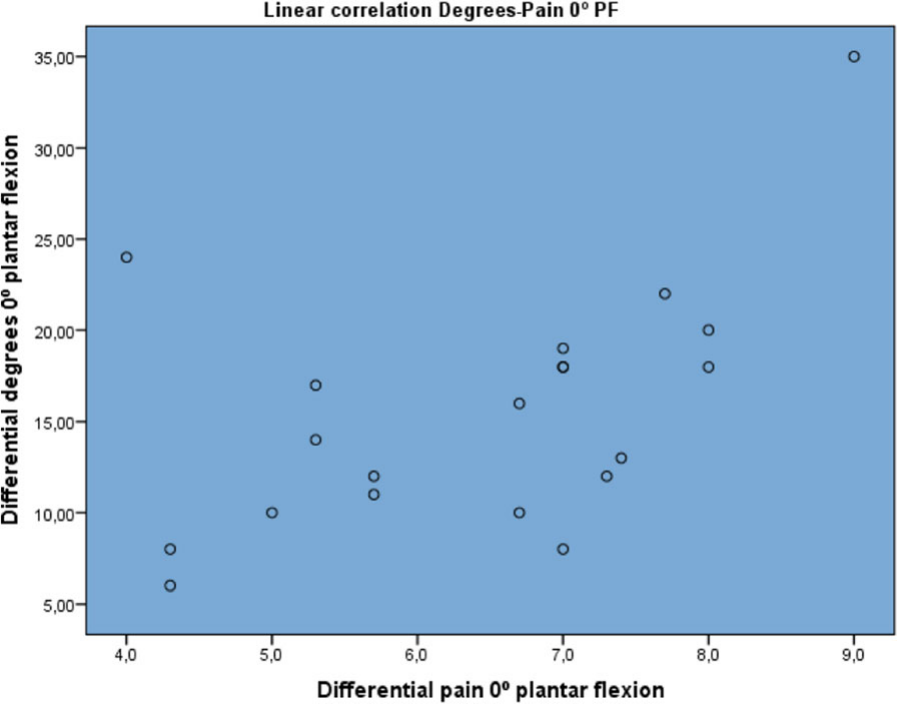
Pearson’s correlation 0° plantar flexion

**Fig. 1 (abstract P123).**
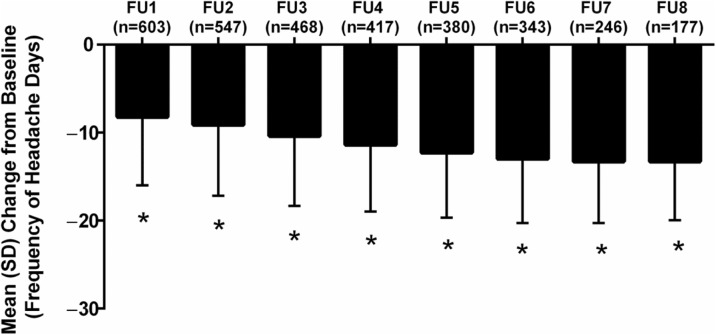
Headache-day frequency calculated using the patient-reported estimate of number of days in a month with headache. Wilcoxon signed-rank test for change versus baseline (level of significance: 5%). The number of patients in follow-up (FU) sessions 9-12 were: FU9, n=67; FU10, n=38; FU11, n=21; FU12, n=16. The mean change from baseline for FU sessions 9-12 were each significant (*P<0.001)

**References**

1. Stovner LJ, Hagen K, Jensen R, Katsarava Z, Lipton RB, Scher AI, et al. The global burden of headache: a documentation of headache prevalence and disability worldwide. Cephalalgia 2007; 27: 193-210

2. ICHD-3 Headache Classification Committee of the International Headache Society (IHS): The International Classification of Headache Disorders, 3er ed. Cephalalgia 2013;33: 629-808.

3. Bendsten L. Central sensitization in tension type headache-possible pathophysiological mechanisms. Cephalalgia. 2000;20(5): 486-508.

### P122 Physiotherapy and basic headache research: Why using a pre-post design?

#### Sarah Mingels^1,2^, Marita Granitzer^1^

##### ^1^REVAL Rehabilitation Research Centre, Biomedical Research Institute, Faculty of Medicine and Life Sciences, Hasselt University, 3590-Belgium; ^2^Musculoskeletal Research Unit, Department of Rehabilitation Sciences, Faculty of Kinesiology and Rehabilitation Sciences, Leuven University, 3000-Belgium

###### **Correspondence:** Sarah Mingels (sarah.mingels@uhasselt.be)

**Background**

In physiotherapy, headache-research is often limited to instantaneous cross-sectional measurements of e.g. cervical mobility. However, a longitudinal task might affect baseline outcomes. Understanding the influence of the latter on headache features is relevant to develop preventive guidelines. Materials and methods

Design. A pre-post design was set up to compare ‘Pressure Pain Thresholds (PPT)’ and maximal active cervical range of motion (CROM) before (pre-test) and after (post-test) a writing task between 18 females with headache (23.2±1.7 years) and 18 matched controls (23.6±2.2 years). Criteria. Headache-group inclusion-criteria were: females between 18-30 years, meeting the diagnostic criteria of episodic tension-type headache according to the International Headache Society, headache provoked by posture. Exclusion-criteria: pregnancy, physiotherapy for headache 12 months before the study, serious pathology and a history of neck or head trauma. Control-group inclusion-criteria were: healthy age-matched females. Exclusion-criteria: pregnancy, history of neck or head trauma. Measurements and outcomes. Bilateral PPT, measured with the Somedic Algometer (slope 30kPa/s/cm^2^) in the anterior temporal, suboccipital, upper trapezius and anterior tibial muscles and maximal active flexion and extension CROM, measured with the Cervical Range of Motion device (°) were primary outcomes. Headache frequency, duration and intensity were secondary outcomes extracted from a headache diary. Statistics. Data-analysis was done with a 95% confidence level (p<0.05). Normality was evaluated by the Shapiro-Wilk test, comparisons within and between groups by the Wilcoxon Signed Rank respectively Mann-Whitney U-test. Ethics. Approval by the Medical Ethical Committee of the ‘Ziekenhuis Oost-Limburg’ (B371201423025).

**Results**

At baseline only maximal active flexion CROM differed significantly (p=0.022) between groups with lower values in the Headache-group. From pre-to post-test the Headache-group compared to the Control-group showed a significant: 1) PPT reduction vs. an increase in the Control-group in the anterior temporal left and right (p=0.0290; p=0.0051) and the upper trapezius right (p=0.0237) and 2) larger drop in maximal active extension CROM (p=0.012) (Headache-group: post 59.65°±8.19; pre 69.59°±6.28 vs Control-group: post 67.39°±6.96; pre 71.85°±10.85).

**Conclusion**

In the Headache-group the performance of a simple writing task decreased PPT and maximal active CROM. These results are closely related with sensitization. Physiotherapists should be aware that baseline characteristics of patients with headache and healthy controls are comparable. However, the baseline headache profile can be influenced by a task performance. Longitudinal designs could therefore be relevant to evaluate factors contributing to headache.

**Keywords:** Headache, pre-post design, writing task.

### P123 Real-life use of onabotulinumtoxinA for the symptomatic treatment of chronic migraine: The Repose Study

#### Fayyaz Ahmed,^1^ Charly Gaul,^2^ Paolo Martelletti,^3^ Juan Carlos Garcia-Monco,^4^ Aubrey Manack Adams^5^

##### ^1^Spire Hesslewood Clinic, Hull York Medical School, Brough, Hull, UK; ^2^Department of Headache and Facial Pain, Migraine and Headache Clinic, Koenigstein, Germany; ^3^Department of Clinical and Molecular Medicine, Sapienza University, Regional Referral Headache Centre, Rome, Italy; ^4^Service of Neurology, Hospital de Galdakao, Vizcaya, Spain; ^5^Global Medical Affairs, Allergan plc, Irvine, CA, USA

###### **Correspondence:** Fayyaz Ahmed (fayyaz.ahmed@hey.nhs.uk)

**Background**

The REPOSE Study, a European, multicenter, prospective, non-interventional study, investigated the effectiveness and safety of real-life use of onabotulinumtoxinA for chronic migraine (CM).

**Materials and Methods**

Adults prescribed onabotulinumtoxinA for CM were enrolled. Patients received onabotulinumtoxinA approximately every 12 weeks according to their physician’s usual practice, guided by the Summary of Product Characteristics. OnabotulinumtoxinA injection practices, safety, headache-day frequency, and Migraine Specific Quality of Life Questionnaire (MSQ) were collected at baseline and follow-up visits.

**Results**

Among 644 patients enrolled, 633 patients received ≥1 onabotulinumtoxinA dose for a total of 3499 onabotulinumtoxinA treatments. Patients had a mean (SD) age of 45.4 (12) years, were typically women (85.3%) and had a mean of 20.6 headache days/month. The median dose and median number of injection sites of onabotulinumtoxinA per session (baseline up to follow-up session 8) was 155 U and 31 sites, respectively. Through follow-up session 8, patient-reported estimates of headache days/month (≥4 hours) were significantly reduced from baseline (*P*<0.001 at each follow-up session, Fig. 1). The MSQ domain scores (Role Restrictive, Preventive, and Emotional) were significantly reduced from baseline at each follow-up session. Adverse drug reactions, typically of mild to moderate severity, were reported by 18.3% of patients; eyelid ptosis (5.4%), neck pain (3.0%), and musculoskeletal stiffness (2.7%) were most frequently reported.

**Conclusion**

Preventive treatment of CM with onabotulinumtoxinA in a longer-term (24-month) real-world setting sustains a reduction in the frequency of headache days and significantly improves quality of life relative to baseline. No new safety concerns were identified.

**Fig. 1 (abstract P131).**
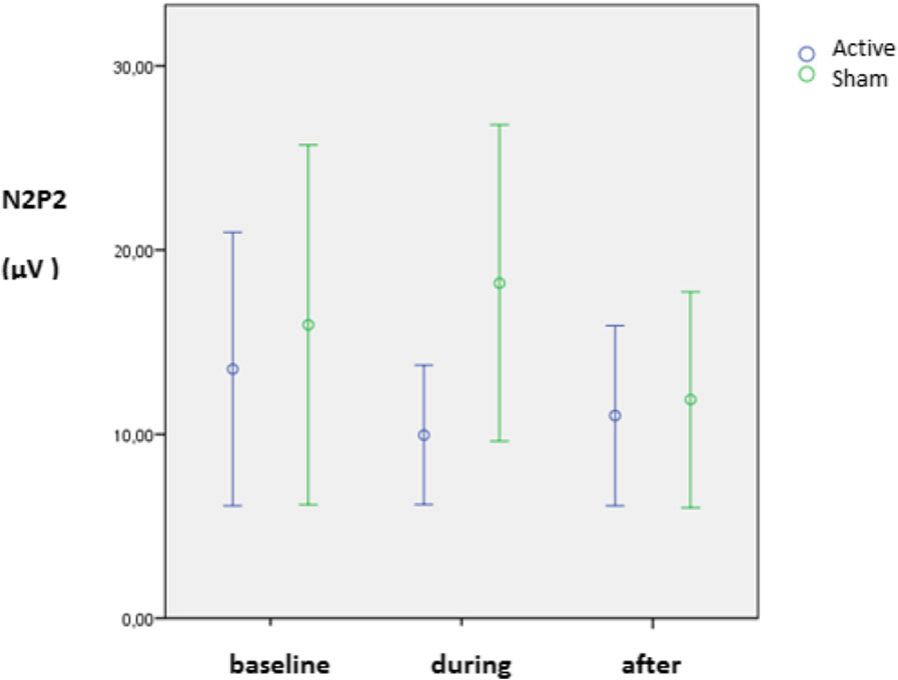
N2P2 complex amplitude

**Acknowledgments**

Editorial support for development of this abstract was provided by Lee B. Hohaia, PharmD, and Dana Franznick, PharmD, at Complete Healthcare Communications, LLC (Chadds Ford, PA), a CHC Group company, and funded by Allergan, plc (Dublin, Ireland).

**Funding**

Allergan plc.

### P124 Simulated airplane headache: a proxy towards identification of underlying mechanisms

#### Torben Petersen

**Background**

Airplane Headache(AH) is a short lasting but intense headache which occurs during flights, most often during take-off and landing. Literature on this condition is sparse, especially surrounding the pathological mechanisms underlying the occurrence of the headache. Diagnosis and treatment of the condition are also lacking, and would benefit greatly if potential pathways involved in the pathogenesis of AH were to be discovered. This study is aimed at examining a simulated airplane condition as a proxy towards identification of the underlying mechanisms of the condition.

**Methods**

A total of fourteen participants were included in the study. Seven of these suffered from AH and would act as the control group, whereas the remaining seven were healthy matched controls. A simulation of conditions as those experienced in a real flight, were achieved by using a pressure chamber, thereby enabling the development of AH. A selection of potential biomarkers were to be measured, including cortisol, blood pressure, salivary prostaglandin E2(PGE2), pulse, facial thermos-images and saturation pulse oxygen (SPO). All biomarkers were defined beforehand, and samples were collected before, during and after exposure to the pressure chamber. The saliva samples were subsequently analyzed using ELISA techniques, whereas statistical tests and data analysis were performed using SPSS version 22.0.

**Results**

As expected, the non-AH-group did not develop any headache during the session. All participants in the AH-group did experience a headache, bearing resemblance to an AH experience during flight. Analysis of data showed that the levels for cortisol, SPO and PGE2, were significantly different between the two groups during the flight simulation.

**Conclusion**

Not only did the pressure chamber enable us to evoke an AH-like attack, it also allowed us to examine potential biomarkers for AH. We noted a difference in values when comparing healthy controls with the affected individuals, as cortisol levels, SPO and PGE2 were dysregulated during the AH-attack. These findings are promising, and gives rise to possible use of a pressure chambers as model for inducing AH, for all kinds of future AH studies.

### P125 Chronic Headache Education and Self-management Study (CHESS) – a feasibility study

#### Kimberley White, Rachel Potter, Martin Underwood, On behalf of the CHESS team

##### Warwick Clinical Trials Unit, Warwick Medical School, University of Warwick, Coventry. CV4 7AL, UK

###### **Correspondence:** Kimberley White (Kimberley.white@warwick.ac.uk)

**Background and aims**

The Chronic Headache Education and Self-management Study (CHESS) is a five year programme of work leading to a large multi-centre, randomised controlled trial. As part of the programme we have conducted a feasibility study to 1. Develop and test strategies for recruiting participants with chronic headache from primary care 2. Validate a telephone classification interview that can be used by a non-headache specialist to classify chronic headache disorders 3. Pilot a group education and self- management intervention for chronic headache.

**Methods**

We recruited participants from 14 general practices in the West Midland region of the UK. The study team developed a search strategy to identify patients with chronic headache from general practice databases and screened potential participants for eligibility. Participants completed questionnaires at baseline, 2 weeks and 12 weeks. Participants received a chronic headache classification interview with a specially trained nurse and had a second interview with a doctor specialised in headache. A sub-sample of participants were invited to attend an education and self-management intervention.

**Results**

From combined database searches 1643 (1.3% of total list size) were invited to take part in the study. In total 393(24%) expressed an interest in the study, 174 were eligible and 131 provided written consent. All participants completed baseline questionnaires, 115 (88%) two week and 103 (79%) 12 week questionnaires. We completed 100 paired telephone chronic headache classification interviews and 18 participants attended the self-management intervention.

**Conclusion**

We successfully recruited general practices and people living with chronic headache from primary care settings. Our biggest challenges were being able to contact participants for eligibility and classification telephone interviews and delivering the group intervention at times convenient for a largely working population. We have used the findings to inform the design and delivery of the RCT.

### P126 Management of headache in the acute medical unit: what can we improve and how?

#### Hannah C. Johnson^1^, Sophie L. Main^1^, Oliver A.T. Seglah^1^, Johann R. Selvarajah^1,2^

##### ^1^School of Medicine, Dentistry and Nursing, University of Glasgow, Glasgow, G12 8QQ, United Kingdom; ^2^Institute of Neurological Sciences, Queen Elizabeth University Hospital, Glasgow, G51 4TF, United Kingdom

###### **Correspondence:** Oliver A.T. Seglah (oliverseglah@doctors.org.uk)

**Background**

Acute management of headache is challenging and clinicians vary in their approach and expertise to this problem [1,2]. We explored practice on acute medical wards in an urban teaching hospital to improve efficiency and quality of care.

**Methods**

We conducted a retrospective analysis of clinical data in all patients with a primary complaint of non-traumatic headache admitted to an acute medical service in Glasgow, UK, in 2012. Hospital re-attendances were recorded over 3 years follow-up. We excluded any patient that did not require admission to a medical bed. From a total population of 345 patients, 108 patients were randomly selected for this study and data were collated from clinical records.

**Results**

78% (n=84) of patients were female and the mean age was 44 years (range 15 – 91 years). Median duration of admission was 3 days. 28% (n=30) presented with acute headache. In 44% (n=47), documented clinical features were inadequate for classification of headache. Past history of headache was unexplored in 57% (n=62) and in 31% (n=34) the bedside examination was incomplete.

Brain imaging, mainly with CT, was performed in 82% (n=89). Only 3 patients with acute headache underwent initial imaging within 6 hours of symptom onset. Brain imaging identified a secondary cause in only 5 patients. Lumbar puncture was performed in 54% (n=58) and identified a secondary cause in only 6 patients.

In 39% (n=42) no diagnosis was made on discharge from hospital. Final diagnosis was a primary headache syndrome in 31% (n=33), but discharge prescriptions included appropriate therapies in only 4 patients. Final diagnosis was a secondary headache in 31% (n=33), but in approximately half of these cases review of clinical data suggested that the diagnosis was not secure. Only one subarachnoid haemorrhage was identified. On follow-up, 33% (n=36) patients attended outpatient headache services and 6% (n=7) were readmitted with headache.

**Conclusions**

In these patients hospitalised with headache, we suggest that bedside assessment could be refined, investigations are overused, diagnosis is often incomplete and treatment strategies can be unsophisticated. Addressing these issues may improve clinical efficiency in diagnosis and treatment, reduce bed occupancy and reduce the rate of recurrent headache. We have developed various service interventions to achieve these goals, including an algorithm for investigation of acute headache, an admission clerking proforma and a clinical handbook for junior doctors. These will be available for review at the conference.

**References**

1. Dobb B, Cooper J. A pilot survey of decisions by acute medicine staff after thunderclap headache. J R Coll Physicians Edinb. 2013; 43:207-214.

2. Binks S, Nagy A, Ganesalingam J, Ratnarajah A. The assessment of headaches on the acute medical unit: is it adequate and how could it be improved? Clin Med (Lond). 2017; 17(2):114-120.

### P127 The relationship between sleep disorders and migraine: Results from the Chronic Migraine Epidemiology and Outcomes (CaMEO) study

#### Dawn C. Buse,^1^Jeanetta C. Rains,^2^Jelena M. Pavlovic,^1^Kristina M. Fanning,^3^Michael L. Reed,^3^Aubrey Manack Adams,^4^Richard B. Lipton^1^

##### ^1^Department of Neurology, Albert Einstein College of Medicine, Bronx, NY, USA;^2^Elliot Hospital, Center for Sleep Evaluation, Manchester, NH, USA;^3^Vedanta Research, Chapel Hill, NC, USA;^4^Global Medical Affairs, Allergan plc, Irvine, CA, USA

###### **Correspondence:**Dawn C. Buse (dbuse@montefiore.org)

**Background**

This cross-sectional analysis from the Chronic Migraine Epidemiology and Outcomes (CaMEO) Study assessed the relationship of sleep disturbances and sleep apnea as comorbidities of episodic (EM) and chronic migraine (CM).

**Materials and Methods**

CaMEO participants were recruited from an online panel using quota sampling and completed baseline and 3-month follow-up surveys over 1 year. The Comorbidities/Endophenotypes survey assessed the risk of sleep apnea using the Berlin Scale for Sleep Apnea (“yes”=high risk; “No”=low risk) and a self-report of a physician diagnosis of sleep apnea. Sleep disturbances were measured using the Medical Outcomes Study Sleep Scale.

**Results**

16,763 (99.8%) CaMEO Study respondents received Comorbidities/Endophenotypes survey invitations, of whom 12,810 (76.4%: EM, 11,699; CM, 1,111) provided valid data. Based on the Berlin Scale, 37.0% were “at high risk” for sleep apnea (EM, 35.6%; CM, 51.8%; chi-square, 113.7; *P*<0.001). Sleep apnea risk significantly increased with higher body mass index, with 10.2%, 14.4%, 29.8%, and 74.1% of respondents at risk for sleep apnea when stratified by underweight (n=489), normal (n=4,813), overweight (n=3,534), and obese categories (n=3,974). 10.1% of respondents self-reported sleep apnea (n=1,293: EM, 9.7%; CM, 14.1%; *P*<0.001) (Table 1). Among those reporting sleep apnea, 75.7% also reported a physician diagnosis (EM, 74.7%; CM, 82.8%; *P*<0.05). Commonly reported Medical Outcomes Study sleep subscales were Snoring (EM, 32.1%; CM, 33.9%), Shortness of breath (EM, 20.6%; CM, 29.8%), (daytime) Somnolence (EM, 21.2%; CM, 23.4%), and Sleep inadequacy (EM, 22.1%; CM, 24.2%; all *P*<0.001).

**Conclusions**

Compared with reported general prevalence estimates ranging from 1.0% to 6.0% for sleep apnea, we found an increased risk and potential underdiagnosis of sleep apnea and sleep disturbances among those with migraine, especially CM.

**Table 1 (abstract P127).** Self-Reported Clinical History for Sleep Apnea

	EM (n=11,699)	CM (n=1,111)	Total (N=12,810)	Chi-square	*P*-value
*“Have you ever had sleep apnea?”* ^*a*^					
No	10,563 (90.3)	954 (85.9)	11,517 (89.9)	21.9	<0.001
Yes	1,136 (9.7)	157 (14.1)	1,293 (10.1)		
Total	11,699 (100.0)	1,111 (100.0)	12,810 (100.0)		
*Among those reporting ever having sleep apnea, “Has this condition been diagnosed or confirmed by a healthcare professional?”*					
No	287 (25.3)	27 (17.2)	314 (24.3)	4.9	<0.05
Yes	849 (74.7)	130 (82.8)	979 (75.7)		
Total	1,136 (100.0)	157 (100.0)	1,293 (100.0)		

^*a*^A response of “Yes” denotes high risk; a response of “No” denotes low risk

**Funding**

Allergan plc.

### P128 Are there many placebos? “Sugar pills” and stimulation of complex emotional networks -Pilot data

#### Maria Nicolodi, Vanessa Sandoval, Anna Torrini, Martina Irasa

##### Foundation Prevention and Therapy of Primary Pain and Headache, Florence, Italy

###### **Correspondence:**Maria Nicolodi

**Background**

Placebo effect is proved not to depend on a single transmitter. Placebo is currently considered a “thing”, may be a fake drug as currently intended in Randomized Controlled Trials. In all cases, the “thing” named placebo has to influence networks controlling affective set-up [1-3].

**Material and methods**

Observation 1 consisted in using Affective Neuroscience Personality Scales [4] in 48 chronic migraine sufferers (21 males, mean age 37.1 ±3.5 SD) for estimating of primary processes emotional traits of patients. This was propaedeutic to propose a powerful placebo [5] for Observation 2 that started in December 2016. It has to include 400 chronic migraine sufferers refractory to topiramate, known to antagonize glutamatergic receptor subgroups. Following data concern 102 chronic migraine sufferers reporting topiramate – induced

relief -14.2 ± 0.7 hours with pain, -0.9 ±1 severity of migraine attacks. Seventy-five out of them (43 females, 32 males mean age 42.3 ±3.8SD) volunteered a treatment based on outcomes of Observation 1. Volunteers were randomly divided in group A and Group B. Both groups were allocated to a nurturing approach aimed to counteract fear-rage/despair. Nurturing consisted in a guided meditation and music tracks 430 Hertz frequency. The track was to be heard 3 times/daily for 15 -150 min. Patients of Group A received topiramate that was the therapy they referred as ineffective. Group B was administered negative modulators at metabotrobic glutamatergic receptors level. Escape medication was sumatriptan 100 mg. Plan: a 14 days run-in, a 3 months-treatment.

**Results**

Observation 1 indicates that intractable chronic pain relates p> 0.00025(Odds Ratio and M-Anova) to seeking system thwart and arousal of rage/despair. Observation 2 enlightened that Group A reported –27.3 ± 04SD hours/ pain and -35.6+ 4.8 attacks severity. Group B reported a – 65.5 ± 1.2 SD hours/pain and -45.2± 3.1 SD attacks severity on 0-100 VAS. Relief of Group A, using the same drug inducing no therapeutic effect was p> 0.005 versus conventional drug alone, while Group B reported a relief larger (p>0.0001) than Group A.

**Conclusion**

It could be inferred that pain-relief observed in Group B might relate to administration of substances that likely act on rage–despair pathways [6]. On the other hand, topiramate appertains to the same drug-category. Finally, it seems interesting that an ineffective drug becomes active when associated to a placebo likely acting on specific emotional networks.

**References**

1. Siegel A. *The neurobiology of aggression and rage.* CRC Press –Taylor & Francis; 2004

2. Panksepp J, Fuchs T, Iacobucci P. The basic neuroscience of emotional experiences in mammals: the case of subcortical fear circuitry and implications for clinical anxiety. *Applied Animal Behav Sc.* 2010; 5: 328-39

3. Nicolodi M, Torrini A. From nocebo effect to the hypothesis of nocebo comparison and psychometric tests as entry criteria in headache trials. Cepalalgia; 2009; 29(1): 39

4. Barrett FS, Robins RW, Janata P. A brief form of the Affective Neuroscience Personality Scales. Psychol Assess. 2013;25(3):826-43.

5. Blease C. The principle of parity: the placebo “effect” and physician communication. J of Medical Ethics; 2012:38(4):199-201

6. Maes M, Verkerk R, Vandoolaeghe E, Lin A, Scharpé S. Serum levels of excitatory amino acids, serine, glycine, histidine, threonine, taurine, alanine and arginine in treatment-resistant depression: modulation by treatment with antidepressants and prediction of clinical responsivity. Acta Psychiatr Scand.1998;97(4):302-8.

### P129 Occipital Headaches in Children – Are They a Red Flag?

#### Jacob Genizi^1,2,3^Amal Khourieh-Matar^2^Nurit Assaf^2,3^Irena Chistyakov^2^Isaac Srugo^2,3^

##### ^1^Pediatric Neurology Unit, Bnai Zion Medical Center, Haifa, Israel;^2^Department of Pediatrics, Bnai Zion Medical Center, Haifa, Israel;^3^Bruce and Ruth Rappaport Faculty of Medicine, Technion, Haifa, Israel

**Background**: Occipital headache is considered a risk factor for serious secondary headache pathology. The purpose of our study was to assess the etiology of occipital headaches among children visiting the emergency department (ED).

**Methods**: Subjects were children aged 5-18 years who were referred to the ED due to headaches during the years 2013-2014.

**Results**: During the study period, 314 patients with headaches were seen at our ED ( 1.7%). Of these, 204 (65%) were male, and the mean patient age was 11.8 years. Thirty-nine patients (12.4%; 95% CI: 9.1-16.4%) had occipital headaches. There were no statistically significant differences in age, gender, ethnicity or medical history, including diagnosis of migraine or TTH, between patients with occipital headaches and other headache locations. There were no differences between patients with occipital headaches and those with other headache locations in duration, headache type, ability to describe their pain, and headache that wakes up from sleep.

Of the 314 patients, 78 underwent CT or MRI at hospitalization or after discharge (24.8%). Among the occipital group, 13 patients (33%) underwent imaging; however, none had a tumor or other pathological intracranial findings. There was no statistically significant difference in the percentage of patients who underwent imaging between the two groups. Viral infections were the most prevalent final diagnosis (97; 31%), followed by migraine (37; 11.8%). Among the occipital group, viral infection was also the most prevalent final diagnosis (14; 35%). Twenty percent of the occipital group had primary headache, and half of these had migraine.

**Conclusions**: The most common causes of occipital headaches are viral infections and primary headaches. Serious intracranial disorders presenting solely as occipital headaches and not accompanied by other neurological signs are uncommon. Thus, occipital headaches should be evaluated in the same manner as other headache locations.

### P130

**Withdrawn**

### P131 Effect of non-invasive Vagus Nerve Stimulation on Laser Evoked Potentials in Migraine

#### Eleonora Vecchio^1^, Katia Ricci^1^, Anna Montemurro^1^, Lia Spitzer^2^, Eric J. Liebler^2^, Marina de Tommaso^1^

##### ^1^Applied Neurophysiology and Pain Unit, SMBNOS Department, Bari Aldo Moro University (Italy);^2^electroCore LLC, Scientific, Medical and Clinical Affairs, Basking Ridge, NJ 07920 USA

###### **Correspondence:**Eleonora Vecchio (eleonora.vecchio@uniba.it)

**Background:** non-invasive Vagus Nerve Stimulation (nVNS) is a new promising treatment of migraine. The efficacy of cervical nVNS (gammaCore®) in primary headache prevention or acute therapy is currently being assessed in large studies. Cervical nVNS was recently shown to rapidly inhibit cortical spreading depression in rodents, providing important insights into the mode of action of nVNS [1]. We aimed to test the effects of cervical nVNS on pain perception and cortical responses induced by painful laser stimuli delivered to the right forehead and the right hand in a cohort of migraine patients.

**Materials and methods:** Twenty-eight migraine patients were selected and randomly assigned to active or sham nVNS stimulation on the right and left side of the neck with gammaCore®. Laser evoked responses (LEPs), elicited bilaterally from the hand and the forehead, were recorded from 64 scalp electrodes in basal condition, during nVNS and sham stimulation and also two minutes after stimulation. We evaluated amplitude and latency of N1 from temporal channels and N2P2 from Cz channel.

**Results:** We did not find significant acute changes in LEPs parameters and pain perception during active and sham VNS. During and after active nVNS we observed a slight reduction of amplitude of the N2P2 complex (Fig. 1). After nVNS there was also an increase of N2 and P2 latency evoked by the forehead laser stimulation.

**Conclusions:** Active and sham nVNS do not seem to have a clear and immediate acute effect on the transmission of trigeminal pain. Further studies are needed to explore its effect on cortical excitability by studying basal EEG rhythms.

**Fig. 1 (abstract P132).**
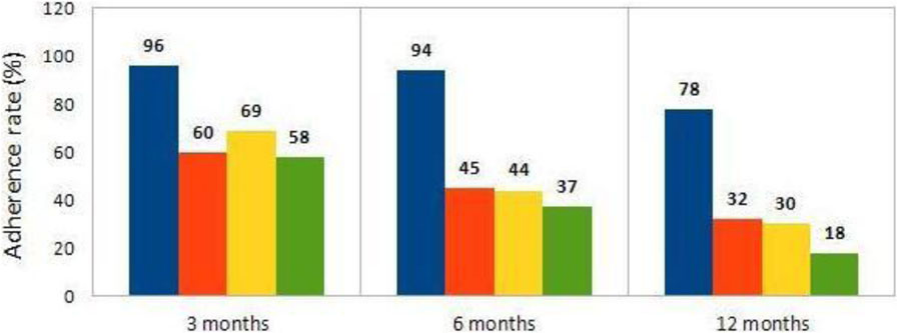
Adherence rate to MOH preventive treatments by class. Blue: OnabotulinumtoxinA, red: antidepressants, yellow: antihypertensives, green: anticonvulsants

**References**

1. Chen S-P, Ay I, Lopes de Morais A, Qin T, Zheng Y, Sadhegian H, et al. Vagus nerve stimulation inhibits cortical spreading depression. *Pain*. 2015;157:1

### P132 Adherence to prophylaxis pharmacotherapies in patients with Medication Overuse Headache: a 10-year experience in a single-centre

#### Lanfranco Pellesi^1^, Simona Guerzoni^1^, Carlo Baraldi^1^, Maria M Cainazzo^1^, Luigi A Pini^1^

##### ^1^Medical Toxicology and Headache Centre, University of Modena and Reggio Emilia, Modena, 41124, Italy

###### **Correspondence:**Lanfranco Pellesi (lanfranco.pellesi@gmail.com)

**Background**

Medication overuse headache (MOH) is a chronic headache associated with the overuse of one or more classes of headache abortive medications, including triptans, NSAIDs, paracetamol and the analgesics combination [1]. MOH is one of the most costly neurological disorders [2] and it is characterized by a low productivity at work and unwillingness to social activities [3]. Patients with MOH may benefit from preventive treatments, such as OnabotulinumtoxinA and oral pharmacotherapies, but they require adherence to dosing regimens [4]. Therapeutic adherence is crucial in chronic disorders, such as hypertension, depression and chronic pain, because the poor adherence compromises the treatment effectiveness [5]. Currently there are few data about adherence to preventive treatments in chronic migraine [6], no one is aimed specifically to MOH patients.

**Materials and methods**

Retrospective analysis of our centre’s electronic database was conducted to identify patients between 18 and 75 years of age, diagnosed with MOH according to International criteria [1], initiated a preventive treatment – OnabotulinumtoxinA or an oral preventive medication (OPM) – and followed up for at least one year. Proportion of day covered (PDC) was calculated, with an adherence cut-off ≥ 80%. Adherence and adverse events rates were compared with 2-sample test of proportion, using large sample statistics (STATAIC 13 software).

**Results**

At the time of writing, 253 patients met the inclusion/exclusion criteria (mean age: 47.6, female: 84%). 93 annual therapies of OnabotulinumtoxinA and 760 OPMs were followed up. After twelve months, the anticonvulsants were associated with a lower adherence than antihypertensives (p = 0.03662), antidepressants (p = 0.00124) and OnabotulinumtoxinA (p < 0.001) (Fig. 1). Among the causes of poor adherence to oral pharmacotherapies, the number of adverse events reported for anticonvulsants is significantly higher at 12 months, compared to antidepressants (p = 0.00362) and antihypertensives (p = 0.0035).

**Conclusions**

In the clinical context, the prophylaxis therapies administered in MOH are differently tolerated. OnabotulinumtoxinA prophylactic therapy have the highest adhesion rate, whereas the anticonvulsants drugs seem to be the less tolerated, because of a higher incidence of adverse events. We expect to increase the number of patients to confirm these already interesting data.

**Fig. 1 (abstract P135).**
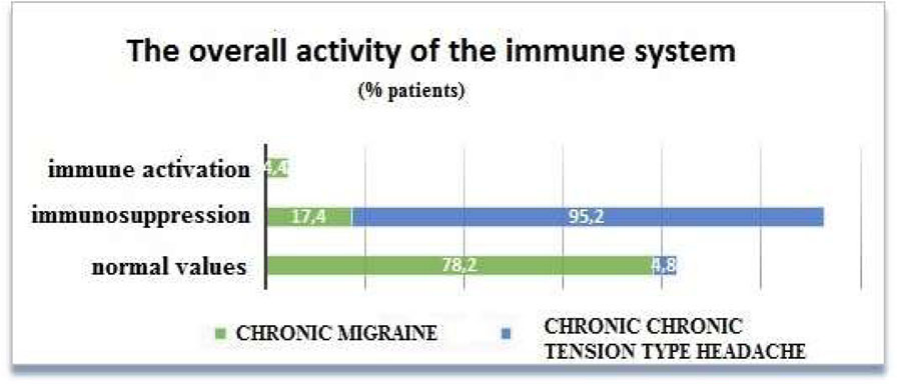
The Overall activity in patients with CTTH and CM

### P133 Cerebral hemodynamics in the anterior and posterior circulation in patients with migraine with aura, stroke and controls

#### Altamura Claudia^1^, Paolucci Matteo^2^, Brunelli Nicoletta^2^, Cascio Rizzo Angelo^2^, Vernieri Fabrizio^1^

##### ^1^Neurology Department, Headache and Neurosonology Unit, Campus Bio-Medico University of Rome;^2^Neurology Department, Campus Bio-Medico University of Rome

**Background**

Patients affected by migraine present a higher cerebrovascular risk and a more sever load of white matter hyperintensities than general population, especially in the posterior circulation [1]. Vasomotor Reactivity (VMR) is a marker of efficiency of cerebral hemodynamics: it reflects the arterial capability to dilate in response to a vaso-dilatatory stimulus such as hypercapnia. Migraneurs present a preserved VMR in the anterior circulation [2] but a poor hemodynamics in the posterior circulation [3]. We aimed at comparing VMR in patients with MA, patients with stroke and controls.

**Materials and Methods**

We consecutively enrolled 32 MA patients, 10 young (under 55 years) patients with cryptogenetic stroke and 16 controls. To assess VMR, Breath Holding Index (BHI - percent increase of blood flow mean velocity after 30 second apnea) was simultaneously calculated for middle (MCA) and posterior (PCA) cerebral arteries in all subjects. In stroke patients, VMR was assessed within 7 days from stroke onset.

**Results**

MA patients were more frequently female (F/M=5:1) than controls (F/M=2:1) and stroke patients (F/M=1:1). Stroke patients (49 ys) on average were older than the MA patients (36 ys) and controls (37 ys) (p=.002). In the three groups, VMR in the anterior (MCA) circulation did not differ from VMR in the posterior (PCA) circulation. VMR was significantly different in three groups (p<05) in MCA and in PCA, MA patients displaying the highest VMR (MCA 1,72; PCA 1,73) and stroke patients the poorest (MCA 1,34; PCA 1,38). Controls VMR resulted 1,47 in MCA and 1,45 PCA.

**Conclusions**

Differently from stroke patients, migraneurs with aura present a higher VMR to hypercapnia compared with controls. This finding suggests that the hemodynamic impairment is not the physiopathological link between MA and stroke. Conversely, an excessive dilatation of cerebral vessels in response to minor vasodilatatory stimuli can represent a possible mechanism leading to the migraneur pain. Another, not mutually exclusive, hypothesis theorizes that recurrent cortical spreading depression induces an adaptive vascular over reactivity to protect brain from frequent ischemic events.

**References**

[1] Kurth T, Chabriat H, Bousser MG. Migraine and stroke: a complex association with clinical implications. Lancet Neurol. 2012; 11:92-100.

[2] Vernieri F, Tibuzzi F, Pasqualetti P, Altamura C, Palazzo P, Rossini PM, Silvestrini M. Increased cerebral vasomotor reactivity in migraine with aura: an autoregulation disorder? A transcranial Doppler and near-infrared spectroscopy study. Cephalalgia. 2008; 28:689-95.

[3] Silvestrini M, Baruffaldi R, Bartolini M, Vernieri F, Lanciotti C, Matteis M, Troisi E, Provinciali L. Basilar and middle cerebral artery reactivity in patients with migraine. Headache. 2004; 44:29-34.

### P134 Development of a claims-based algorithm for use in patients with migraine to identify potentially undiagnosed chronic migraine patients

#### Jelena M. Pavlovic^1^, Justin S. Yu^2^, Stephen D. Silberstein^3^, Michael L. Reed^4^, Steve H. Kawahara^5^, Robert P. Cowan^6^, Firas Dabbous^7^, Karen L. Campbell^2^, Anand S. Shewale^2^, Riya Pulicharam^5^, Jonathan W. Kowalski^2^, Hema N. Viswanathan^2^, Richard B. Lipton^1^

##### ^1^Montefiore Medical Center, Bronx, NY, USA;^2^Health Economics and Outcomes Research, Allergan plc, Irvine, CA, 92612, USA;^3^Jefferson Medical Center, Philadelphia, PA, USA;^4^Vedanta Research, Chapel Hill, NC, USA;^5^DaVita Medical Group, El Segundo, CA, USA;^6^Stanford University School of Medicine, Stanford, CA, USA;^7^Independent Consultant, La Jolla, CA, USA

###### **Correspondence:**Justin S. Yu (Justin.Yu@Allergan.com)

**Background**

Published surveys have demonstrated that 75-80% of persons meeting the criteria for chronic migraine (CM) do not report having received an accurate diagnosis [1,2]. The primary objective of this study was to develop a claims-based algorithm for use in patients with migraine to identify potentially undiagnosed CM patients.

**Materials and Methods**

An observational study using claims data and survey data was conducted in a large United States medical group. Eligible patients had continuous enrollment and a migraine diagnosis (ICD-9/10 code of 346.xx/G43.xxx) in the 12-months prior to the screening date. Patients were excluded if they had a prior CM diagnosis (346.7x/G43.7xx) or migraine-related onabotulinumtoxinA claim. The Semi-structured Diagnostic Interview (SSDI) served as the gold standard for identifying CM and was administered to a convenience sample by trained clinicians. The SSDI included 31 questions related to headache frequency, symptoms, disability, medication use, and diagnosis.

A multivariate logistic regression model was used to identify potential predictors of CM based on claims data obtained in the 12 months prior to the screening date. Over 40 potential predictors for CM, identified from the literature and headache expert input, were evaluated for model inclusion. Variables that were significantly different in bi-variate analyses (p<0.05) between SSDI+ (CM) and SSDI- (non-CM) patients were included; each variable was categorized based on the data distribution and clinical relevance. The c-statistic, sensitivity, and specificity were calculated.

**Results**

Of the 108 patients who were included, 64 were SSDI+ and 44 were SSDI- for CM. Four statistically significant predictors of CM status were identified. Patients with ≥15 claims for acute treatment of migraine (including opioids) were nearly six times as likely to have CM as those with <15 claims (OR=5.87, 95% CI=1.34, 25.63); patients with ≥24 visits of any type (outpatient, inpatient, and emergency room visits) were nearly three times as likely to have CM as those with <24 visits (OR=2.80, 95% CI=1.08-7.26); females were 9 times as likely to have CM (OR=9.17, 95% CI=1.26-66.51); patients with claims for ≥2 unique migraine preventive classes were more than 4 times as likely to have CM as those without claims for preventive treatments (OR=4.40, 95% CI=1.19, 16.22). The c-statistic, sensitivity, and specificity for the model were 0.80, 78%, and 73%, respectively.

**Conclusions**

The claims-based algorithm for identification of undiagnosed CM patients demonstrated acceptable sensitivity and specificity, and can be used in health care settings to optimize the diagnosis and management of CM patients who may not be otherwise detected.

**Trial registration**

Not applicable.

**Consent to publish**

Not applicable.

**References**

1. Dodick D, Loder E, Manack Adams A, Buse D, Fanning K, Reed M, Lipton R. Assessing Barriers to Chronic Migraine Consultation, Diagnosis, and Treatment: Results from the Chronic Migraine Epidemiology and Outcomes (CaMEO) Study. Headache. 2016;56(5):821-834.

2. Bigal M, Serrano D, Reed M, Lipton R. Chronic migraine in the population. Neurology. 2008;71:559-566.

### P135 Antibodies in patients with chronic tension type headache and chronic migraine: is there a link between headache and autoimmunity?

#### Anastasia Prishchepa, Alexey Danilov

##### Department of neurology, I.M. Sechenov First Moscow State Medical University, Moscow, 119435, Russia

###### **Correspondence:**Anastasia Prishchepa (prischepa@intermeda.ru)

**Backgrond.** It is noted that the main risk of cephalalgia progression is associated with comorbid disorders including the immunity disturbances [1,2]. Neuroimmunological impairments are not only an important link in the pathogenesis of the headache, but also can be a risk factor for the headache chronization, its atypical course and formation of resistance to pharmacotherapy [3,4]. However, the impact of autoimmunity is poorly covered in literature. In the present study, we measured IgG autoantibodies (a-Abs) against different autoantigens in the serum of patients with chronic tension-type headache (CTTH) and chronic migraine(CM) in order to examine the impact of autoimmune reaction in such subject.

**Materials and methods:** The survey of 44 patients with CTTH (n=21) and CM (n=23) was conducted by a single scheme for both types of headache: physical and neurological examination, psychological evaluation of stress level and stress resistance, immune assay “ELI-Test” (Medical Research Center “Immunculus”) to determine serum levels of 33 a-Abs against antigens of major organs and systems of the human body.

**Results.** The majority of CTTH patients has laboratory immunosuppression 95,2%, in contrast to CM group 17,4% (p<0,01) (Fig. 1).These data correlate with higher stress level by RSM-25 (р<0,01) in CTTH patients (135,48± 20,93) compared to the CM group (112,74± 16,84) and lower stress resistance level (p <0.05) in patients with CTTH (29 ± 8.3) compared with CM group (25.13 ± 6.23). In patients with CM we found significantly more abnormalities in a-Abs level against neurofilament NF-200, Voltage-dependent Ca channels, double-stranded DNA, β2-glycoprotein-1, kidney glomerular cell antigen, peripheral insulin receptors, thyroglobulin, thyroid-stimulating hormone receptor and membrane antigen of adrenal medulla cells. Patients with CTTH presented a significantly more deviation of a-Abs level against μ-opioid receptors, β-endorphin, myocardiocyte membrane antigen and membrane antigen of large intestine (Table 1).

**Conclusions.** We have found specific differences in immune imbalance of chronic tension headaches and chronic migraine. Laboratory data confirm the significant contribution of disturbance of psychoneuroendocrine-immune interactions, peripheral and central sensitization in the pathogenesis of chronic migraine; disturbance of balance in the autonomic nervous system, “bodily distress syndrome” (immunological signs of the large intestine pathology) [5], pronounced muscular spasm and insufficiency of descending inhibitory influences in the pathogenesis of chronic tension headache. Based on results of the experiment we can suggest the significant role of stress in the pathogenesis and development of headache and neuroimmunological imbalance.

**Fig. 1 (abstract P150).**
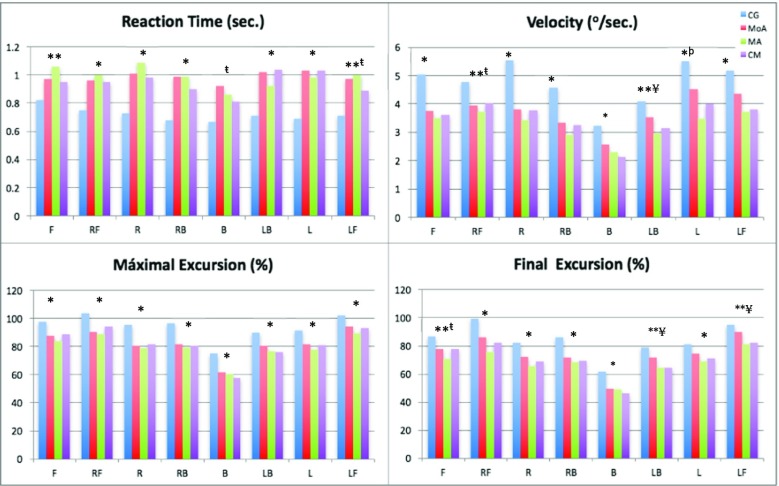
LOS reaction time, velocity, maximal excursion and final excursion of the group control (CG), migraine without aura (MoA), migraine with aura (MA) and chronic migraine (CM) in the eight test directions: forward (F), right forward (RF), right (R), right back (RB), back (B), left back (LB), left (L) and left forward (LF). *p<0.03 CG *versus* MoA, MA e CM; **p=0.02 CG *versus* MA; ^ŧ^p<0.02 CG *versus* MoA; ^¥^ p=0.01 CG *versus* CM; ^þ^MoA *versus* MA

**Table 1 (abstract P135).** Differences in immune imbalance in patients with CM and CTTH

Identified changes of autoantibodies level against:	Total number of patients	Chronic migraine	Chronic tension type headache
NF-200	11 (25%)	9^*^ (39,1%)	2 (9,5%)
Protein of intercurrent as trocytes’ filaments GFAP	7 (15,9%)	3 (13%)	4 (19%)
Protein S100	13 (29,5%)	7 (30,4%)	6 (28,6%)
Myelin basic protein MBP	11 (25%)	5 (21,7%)	6 (28,6%)
Voltage-dependent Ca channels	11 (25%)	9^*^ (39,1%)	2 (9,5%)
Acetylcholine Receptor	7 (15,9%)	4 (17,4%)	3 (14,3%)
Glutamat Receptor	15 (34,1%)	5 (21,7%)	10 (47,6%)
GABA-Receptor	17 (38,7%)	6 (26,1%)	11 (52,4%)
Dopamin Receptor	12 (27,3%)	4 (17,4%)	8 (38,1%)
5HT-Receptor	8 (18,2%)	2 (8,7%)	6 (28,6%)
μ-Opioid Receptor	10 (22,7%)	2 (8,7%)	8^*^ (38,1%)
β -Endorphin	21 (50%)	3 (13%)	18^**^ (85,7%)
dsDNA	15 (34,1%)	14^**^ (60,9%)	1 (4,8%)
Beta2-glycoprotein I	13 (29,5%)	13^**^ (56,5%)	0
Fc-fragment of immunoglobulins IgG	11 (25%)	7 (30,4%)	4 (19%)
Collagen	10 (22,7%)	7 (30,4%)	3 (14,3%)
Membrane antigen of myocardial cells	12 (27,3%)	3 (13%)	9^*^ (42,9%)
b-adrenoreceptors	18 (40,9%)	12 (52,2%)	6 (28,6%)
Membrane antigen of platelets	8 (18,2%)	5 (21,7%)	3 (14,3%)
Anionic antigen of endothelium	6 (13,6%)	3 (13%)	3 (14,3%)
Membrane antigen of kidney	11 (25%)	9^*^ (39,1%)	2 (9,5%)
Membrane antigen of lungs	14 (31,8%)	5 (22,7%)	9 (42,9%)
Membrane antigen of stomach	11 (25%)	8 (34,8%)	3 (14,3%)
Membrane antigen of small intestine	13 (29,5%)	4 (17,4%)	9 (42,9%)
Membrane antigen of large intestine	18 (40,9%)	4 (17,4%)	16^**^ (76,2%)
Cytoplasmic antigen of liver	5 (11,3%)	3 (13%)	2 (9,5%)
Antigen of liver mitochondria	8 (18,2%)	2 (8,7%)	6 (28,6%)
Insulin	19 (43,2%)	11 (47,8%)	8 (38,1%)
Insulin receptors	5 (11,3%)	5^*^ (21,7%)	0
Thyroglobulin	13 (29,5%)	13^**^ (56,5%)	0
Receptor of TSH	9 (20,5%)	8^*^ (34,8%)	1 (4,8%)
Membrane antigen of adrenal gland AdrM-D/C	15 (34,1%)	13^**^ (56,5%)	2 (9,5%)
Membranous antigen of pros tate/spermatozoids	8 (18,2%)	4 (17,4%)	4 (19%)

^*^ p≤0.05, ^**^p≤0.01

**Acknowledgements**

We would like to thank the Medical Research Centre (MRC) “Immunculus” and personally Prof. Alexander Poletaev for providing with the ELI-Viscero-Test-24 and ELI-Neuro-Test-12 and interpreting their results.

**References**

1. Bigal, M.E. & Lipton, R.B., 2006. Modifiable Risk Factors for Migraine Progression. Headache: The Journal of Head and Face Pain, 46(9), pp.1334

2. Cannons, K., 2015. Pain Comorbidities: Understanding and Treating the Complex Patient Giamberardino Maria Adele and Jensen Troels Staechelin Pain Comorbidities: Understanding and Treating the Complex Patient/International Association for the Study of Pain 9780931092923 0931092922. Emergency Nurse, 23(8), pp.13

3. Karpova MI. Rol’ immunnoi sistemy v progressirovanii golovnoi boli napryazheniya i migreni. [dissertation] Chelyabinsk, 2011. (In Russ.)

4. Arumugam M, Parthasarathy V. Reduction of CD4+CD25+ regulatory T-cells in migraine: Is migraine an autoimmune disorder? Journal of Neuroimmunology. 2016, 290:54.

5. Fink P, Schröder A. One single diagnosis, bodily distress syndrome, succeeded to capture 10 diagnostic categories of functional somatic syndromes and somatoform disorders. Journal of Psychosomatic Research. 2010, 68(5):415.

### P136 Non-invasive vagus nerve stimulation for acute treatment of episodic and chronic cluster headache: pooled analysis of data from 2 randomised, double-blind, sham-controlled clinical trials

#### Ilse F de Coo^1^, Juana Marin^2^, Stephen D Silberstein^3^, Deborah I Friedman^4^, Charly Gaul^5^, Alok Tyagi^6^, Eric Liebler^7^, Stewart J Tepper^8^, Michel D Ferrari^1^, Peter J Goadsby^2^

##### ^1^Leiden University Medical Centre, Leiden, the Netherlands;^2^NIHR-Wellcome Trust CRF, King’s College Hospital, London, UK;^3^Jefferson Headache Center, Philadelphia, PA, USA ;^4^UT Southwestern Headache and Facial Pain Program, Dallas, TX, USA;^5^Migraine and Headache Clinic, Königstein, Germany;^6^The Southern General Hospital, Glasgow, UK;^7^electroCore, LLC, Basking Ridge, New Jersey, USA;^8^Geisel School of Medicine at Dartmouth, Hanover, NH, USA

###### **Correspondence:**Michel D Ferrari (M.D.Ferrari@lumc.nl)

**Background**

Non-invasive vagus nerve stimulation (nVNS; gammaCore®) has demonstrated efficacy, safety, and tolerability in 2 large clinical studies of the acute treatment of cluster headache (CH) attacks [1,2]. We conducted a pooled analysis of data from these studies.

**Methods**

Data for 252 patients with CH according to ICHD-II criteria were pooled from 2 prospective, randomised (1:1), double-blind, multicentre, sham-controlled clinical trials. In both studies, patients administered 3 consecutive 120-second vagus nerve stimulations at attack onset. In ACT2, patients were permitted to administer an additional set of 3 stimulations if they were not pain-free by 9 minutes after initiation of the first treatment. Treatments were applied to the right cervical vagus nerve in ACT1 and ipsilateral to the pain in ACT2.

**Results**

In models estimating the proportion of patients who were responders at 15 minutes after treatment initiation for the first CH attack (the ACT1 primary end point), the first-order interaction between treatment group and CH subtype was significant (*P*<0.01) for the ACT1, ACT2, and pooled populations. The first-order interaction between treatment group and CH subtype was also significant (*P*<0.05) for all 3 populations in models estimating the proportion of all treated attacks that were pain-free at 15 minutes (the ACT2 primary end point). Results are therefore presented overall and by CH subtype.

The proportion of patients who achieved the ACT1 primary end point significantly differed among the nVNS and sham groups for episodic CH (eCH) in ACT1 (34% vs 11%; *P*=0.01) and the pooled analysis (39% vs 12%; *P*<0.01) but not in ACT2 (50% vs 15%; *P*=0.07). The proportion of all treated attacks that achieved the ACT2 primary end point significantly differed among the nVNS and sham groups for eCH patients in ACT1 (15% vs 6%; *P*<0.05), ACT2 (35% vs 7%; *P*<0.05), and the pooled analysis (24% vs 7%; *P*<0.01). These end points did not significantly differ among treatment groups for the total CH or chronic CH (cCH) populations in ACT1, ACT2, or the pooled analysis. No serious adverse device effects were reported.

**Conclusions**

This pooled analysis is the largest investigation of a drug or device for the acute treatment of CH attacks and supports the acute use of nVNS as a viable, safe, and effective option for patients with eCH. This analysis also provides the impetus for further research to explore the role of nVNS in the treatment of patients with cCH.

**Acknowledgements**

This study was sponsored by electroCore, LLC. We present this abstract on behalf of the ACT1 and ACT2 Study Groups.

**Trial registrations**

NCT01792817, NCT01958125

**References**

1. Silberstein SD, Mechtler LL, Kudrow DB, et al. Non-invasive vagus nerve stimulation for the acute treatment of cluster headache: findings from the randomized, double-blind, sham-controlled ACT1 study. *Headache*. 2016;56(8):1317-1332.

2. Goadsby PJ, de Coo I, Silver N, et al. Non-invasive vagus nerve stimulation for the acute treatment of episodic and chronic cluster headache: findings from the randomized, double-blind, sham-controlled ACT2 study [AHS abstract IOR03]. *Headache*. 2017;57(suppl 3):113-226.

### P137 The profile of super-responders to onabotulinumtoxin A for chronic migraine: data from an observational study

#### Luana Evangelista^1^, Simona Guezoni^2^, Lanfranco Pellesi^2^, Francesca Pistoia^1^, Cindy Tiseo^1^, Luigi Alberto Pini^2^, Simona Sacco^1^

##### ^1^Department of Applied Clinical Sciences and Biotechnology, Section of Neurology, University of L’Aquila, L’Aquila, Italy;^2^Headache and Drug Abuse Research Centre, Policlinico Hospital, University of Modena e Reggio Emilia, Modena, Italy

###### **Correspondence:**Simona Sacco (simona.sacco@univaq.it)

**Background**

Onabotulinumtoxin-A (BoNT-A) is the only specific preventative treatment approved for adult patients with chronic migraine (CM) [1-3]. Both experimental and real-life data show that treatment with BoNT-A is safe and effective. In most real-life studies, response to BoNT-A is considered good in the presence of a ≥50% reduction of headache symptoms; however, in our experience, several patients experience a much greater improvement and have ≤4 headache days/monthly (super-responders). Aim of the present study was to evaluate frequency of super-responders after treatment with BoNT-A and identify the characteristics of those patients.

**Methods**

We included consecutive subjects aged ≥18 years with CM attending the Headache Centers of L’Aquila and Modena from October 2013 to June 2017 who were treated with BoNT-A. Baseline characteristics were collected through structured questionnaires and scales.

BoNT-A 155-195U was administered according to the PREEMPT protocol, i.e. every 12 weeks for 56 weeks (5 treatment cycles). We planned to stop treatment after a minimum of 3 doses in case of inadequate response, except when patients expressed their will to continue treatment. In case of CM remission we planned to stop treatment after the achievement of <8 headache days/month. We defined super-responders those patients who had ≤4 headache days /monthly within the 56-week follow-up and non-responders those who had a decrease <30%.

**Results**

We treated 127 subjects (106 women and 21 men) with a mean age of 49.9±10.8 years. Among the 109 patients who completed the 56-week follow-up, 27 (24.8%) were super responders and 40 (36.7%) non-responders. Three (11.1%) patients became super-responders after 1 dose, 4 (14.8%) after 2 doses, 7 (25.9%) after 3 doses, 6 (22.2%) after 4 doses, and 7 (25.9%) after 5 doses. When assessing baseline characteristics of the included patients, we found a lower proportion of patients with medication overuse in super-responders compared with non-responders (63.0% vs 84.6%; P=0.044) (Table 1); the same difference was evident when comparing super-responders with other patients completing the PREEMPT protocol (63.0% vs 87.2%; P=0.010) (Table 2). We found a trend towards lower chronic migraine duration and more depressive and anxiety symptoms in super-responders compared with non-responders (Tables 1 and 2). Follow-up was available for 62 subjects; among “super-responders”, one year after BoNT-A withdrawal, 3 patients (15.8%) presented recurrence of CM whereas 16 (84.2%) had episodic migraine; among this latter group, 14 (87.5%) patients had still ≤4 headache days/monthly whereas 2 (12.5%) had 5-14 headache days/monthly.

**Conclusions**

Our preliminary findings suggest that a consistent proportion of patients with CM treated with BoNT-A has an excellent response to treatment, and that response is sustained over time. Absence of medication overuse is a predictor of excellent responde to BoNT-A treatment as also shorter migraine duration and lower burden in terms of psychiatric symptoms. Our data may indicate that if we want to achieve better and sustained response we should no delay treatment in eligible subjects.

**Table 1 (abstract P137).** Univariate comparison of the characteristics of patients with ≤4 headache days per month (super responders) vs patients who had a decrease <30% in headache days per month (non-responders)

	Super responders (n=27)	Non-responders (n=40)	P value	OR
Age, median (IQR)	54.4 (53-56)	47 (45-54)	0.638	-
Female sex, n (%)	21 (77.6)	37 (92.5)	0.083	0.52 (0.20-1.34)
Migraine years, median (IQR)	44.5 (38-51)	27 (23-45)	0.144	-
Chronic migraine duration (months), median (IQR)	8.5 (5-12)	120 (24-120)	0.056	-
Monthly headache days, median (IQR)	30 (23.5-30)	30 (25-30)	0.146	-
Medication overuse, n (%)	17 (63.0)	33 (84.6)	0.044	1.84 (1.07-3.16)
Allodynia, n (%)	10 (41.7)	16 (47.1)	0.684	0.91 (0.59-1.41)
Unilateral headache, n (%)	12 (44.4)	16 (41.0)	0.782	1.06 (0.70-1.60)
Throbbing headache, n (%)	18 (66.7)	21 (53.8)	0.298	1.24 (0.84-1.84)
Baseline MIDAS score, median (IQR)	106.5 (81-132)	92 (41-203)	0.923	-
Baseline HIT-6 score, median (IQR)	77 (72-82)	71 (64-72)	0.154	-
Baseline VAS score, median (IQR)	9.5 (9-10)	10 (8-10)	0.301	-
Baseline BDI score, median (IQR)	33.5 (30-37)	9 (6-21)	0.051	-
Baseline GAD-7 score, median (IQR)	19 (14-24)	4 (3-12)	0.051	-

**Table 2 (abstract P137).** Univariate comparison of the characteristics of patients with ≤4 headache days per month (super responders) vs all other treated patients

	Super responders (n=27)	Others (n=82)	P value	OR
Age, median (IQR)	54.4 (53-56)	51 (45-54)	0.774	-
Female sex, n (%)	21 (77.6)	71 (86.6)	0.274	0.83 (0.58-1.21)
Migraine years, median (IQR)	44.5 (38-51)	30 (23-45)	0.204	-
Chronic migraine duration (months), median (IQR)	8.5 (5-12)	60 (24-120)	0.061	-
Monthly headache days, median (IQR)	30 (23.5-30)	30 (25-30)	0.093	-
Medication overuse, n (%)	17 (63.0)	68 (87.2)	0.010	0.63 (0.40-0.98)
Allodynia, n (%)	10 (41.7)	24 (34.8)	0.546	0.81 (0.40-1.61)
Unilateral headache, n (%)	12 (44.4)	36 (45.4)	0.960	1.02 (0.53-1.96)
Throbbing headache, n (%)	18 (66.7)	52 (65.0)	0.875	0.95 (0.47-1.89)
Baseline MIDAS score, median (IQR)	106.5 (81-132)	92 (41-180)	0.824	-
Baseline HIT-6 score, median (IQR)	77 (72-82)	66 (61-72)	0.132	-
Baseline VAS score, median (IQR)	9.5 (9-10)	9 (8-10)	0.134	-
Baseline BDI score, median (IQR)	33.5 (30-37)	9 (8-22)	0.059	-
Baseline GAD-7 score, median (IQR)	19 (14-24)	5 (3-14)	0.088	-

**References**

1. Aurora SK, Dodick DW, Turkel CC, DeGryse RE, Silberstein SD, Lipton RB, Diener H-C, Brin MF. PREEMPT 1 Chronic Migraine Study Group. OnabotulinumtoxinA for treatment of chronic migraine: results from the double-blind, randomized, placebo-controlled phase of the PREEMPT 1 trial. Cephalalgia 2010;30:793-803.

2. Diener H-C, Dodick DW, Aurora SK Turkel CC, DeGryse RE, Lipton RB, Silberstein SD Brin MF. PREEMPT 2 Chronic Migraine Study Group. OnabotulinumtoxinA for treatment of chronic migraine: results from the double-blind, randomized, placebo-controlled phase of the PREEMPT 2 trial. Cephalalgia 2010;30:804-814.

3. Lipton RB, Varon SF, Grosberg B, McAllister PJ, Freitag F, Aurora SK, Dodick DW, Silberstein SD, Diener H-C, De Gryse RE, Nolan ME and Turkel CC. OnabotulinumtoxinA improves quality of life and reduces impact of chronic migraine. Neurology 2011;77:1465-1472.

### P138 Personalized Medicine, Drug-Drug Interactions (DDIs) and Adverse Events (AEs) in Migraine Treatment. Rational, Strategy and Targets

#### A. Negro^1,2^, L. Lionetto^3^, C. Cicione^3^, A. Koverech^1,2^, C.M. De Marco^2^, G. Gentile^4,5^, M. Borro^4,5^, M. Simmaco^4,5^, P. Martelletti^1,2^

##### ^1^Department of Clinical and Molecular Medicine, Sapienza University of Rome, Italy;^2^Regional Referral Headache Center, Sant’Andrea Hospital, Rome, Italy;^3^Advanced Molecular Diagnostics Unit, IDI Istituto Dermopatico dell'Immacolata - IRCSS, Rome, Italy;^4^Department of Medical-Surgical Sciences and Translational Medicine, Sapienza University of Rome, Italy;^5^Advanced Molecular Diagnostics Unit, Sant'Andrea Hospital, Sapienza University of Rome, Italy

###### **Correspondence:**A. Negro (andrea.negro@uniroma1.it)

**Background**

Migraine usually requires acute and preventative poly-therapy to target the source of headache pain as well as mitigate the symptomatology of existing comorbidities [1]. In real life setting, migraine patients can be affected by various different diseases not-related to migraine, requiring additional treatment to different multi-targeted mechanisms. This creates a drug-drug interaction (DDI) network, which is one of the commonest cause of the development of Adverse Events (AEs) and reduction of clinical efficacy of the administered drugs. In addition, DDIs can be elided or exacerbated by the individual genetic and phenotypic differences, so genotype and phenotype characterizations might be crucial for the appropriate choice of combination therapies.

**Materials and Methods**

In this study we considered the available guidelines for therapy choices in migraine (level of recommendation I & II) and we proposed a strategy to reduce the most common AEs on the basis of the acute/preventative drug interactions. The potential genotype-phenotype correlations and the pharmacogenomic considerations were based on the data available at the following free databases: DrugBank, SuperCyp, PharmaGkb [1].

**Results**

Among the several polymorphic genes involved in drugs metabolism we focused on the superfamily of cytochrome CYP450, responsible for metabolism of more than 70% of prescribed drugs [1,2]. The developed approach permitted us to analyze the pharmacokinetic and pharmacodynamic interactions between different classes of drugs, to highlight the AEs of specific combination of molecules and finally to suggest a recommended combination therapy.

**Conclusions**

The analysis and the prediction of DDIs is one of the main step of the innovative approach called “Personalized Medicine”, which takes into account each patient as a unique entity. Personalized Medicine applied to migraine requires new models that consider the comorbidity with other neurological and internal pathologies in order to drive the correct prescription of multi-therapies, leading to a reduction of DDIs/AEs [3]. The main goal of this approach is to propose a scientific method to physicians to obtain better performances of their multiple prescriptions with the aim of ensuring the safest and most efficacious treatment to patients [4].

**References**

1. Lionetto L, Borro M, Curto M, Capi M, Negro A, Cipolla F, Gentile G, Martelletti P. Choosing the safest acute therapy during chronic migraine prophylactic treatment: pharmacokinetic and pharmacodynamic consideration. *Exp Opin Drug Metab Toxicol.* 2016;12:399-406

2. Lionetto L, Gentile G, Bellei E, Capi M, Sabato D, Marsibilio F, Simmaco M, Pini LA, Martelletti P. The omics in migraine. *J Headache Pain.* 2013;14:55

3. Borro M, Gentile G, Cipolloni L, Földes-Papp Z, Frati P, Santurro A, Lionetto L, Simmaco M. Personalized healthcare: the DiMA clinical model. *Current Pharmaceutical Biothechnology.* 2017;18:242-252

4. Simmaco M, Borro M, Missori S, Martelletti P. Pharmacogenomics in migraine: catching biomarkers for a predictable disease control. *Expert Rev Neurother.* 2009;9:1267-1269

### P139 **Withdrawn**

### P140 Non-invasive vagus nerve stimulation for the acute treatment of episodic and chronic cluster headache: findings from the randomised, double-blind, sham-controlled ACT2 study

#### Peter J Goadsby^1^, Ilse F de Coo^2^, Nicholas Silver^3^, Alok Tyagi^4^, Fayyaz Ahmed^5^, Charly Gaul^6^, Rigmor H Jensen^7^, Hans-Christoph Diener^8^, Andreas Straube^9^, Eric Liebler^10^, Juana Marin^1^, Michel D Ferrari^2^

##### ^1^NIHR-Wellcome Trust CRF, King’s College Hospital, London, UK;^2^Leiden University Medical Centre, Leiden, the Netherlands;^3^Walton Centre for Neurology and Neurosurgery, Liverpool, UK;^4^The Southern General Hospital, Glasgow, UK;^5^Hull Royal Infirmary, Hull, UK;^6^Migraine and Headache Clinic, Königstein, Germany;^7^Glostrup Hospital, Glostrup, Denmark;^8^West German Headache Centre, Essen, Germany;^9^University of Munich, Munich, Germany;^10^electroCore, LLC, Basking Ridge, New Jersey, USA

###### **Correspondence:**Peter J Goadsby (peter.goadsby@kcl.ac.uk)

**Background**

Non-invasive vagus nerve stimulation (nVNS; gammaCore®) is a novel potential option for the acute and prophylactic treatment of cluster headache (CH). We aimed to evaluate the efficacy, safety, and tolerability of acute nVNS treatment in the sham-controlled ACT2 study of episodic CH (eCH) and chronic CH (cCH).

**Methods**

For the 2-week double-blind period, adults with CH were randomly assigned (1:1) to receive nVNS or sham treatment. Patients self-administered 3 consecutive 120-second stimulations to the cervical branch of the vagus nerve at CH attack onset. A second set of 3 stimulations was permitted if an attack was not aborted (pain-free) within 9 minutes of treatment initiation. Patients were instructed to refrain from using rescue treatments for 15 minutes after treatment initiation.

**Results**

Across 9 EU sites, 102 patients (eCH, n=30; cCH, n=72) were randomly assigned to receive nVNS (n=50) or sham (n=52) treatment. The intent-to-treat population included 48 nVNS-treated patients (eCH, n=14; cCH, n=34) and 44 sham-treated patients (eCH, n=13; cCH, n=31). In the total cohort, percentages of treated attacks that were pain-free (pain score=0) at 15 minutes were not significantly different between groups (nVNS, 14%; sham, 12%; primary end point). Significant benefits of nVNS over sham were seen in the eCH subgroup (nVNS, 48%; sham, 6%; *P*<0.01) but not in the cCH subgroup (nVNS, 5%; sham, 13%). The mean decrease in pain intensity score (scale, 0-4 points) from attack onset to 15 minutes after treatment initiation was not significantly different between treatments in the total cohort (nVNS, −1.3; sham, −0.9). For the eCH subgroup, the pain intensity decrease was significantly more pronounced with nVNS (−1.7) than with sham (−0.6) (*P*=0.01); the cCH subgroup showed no significant treatment difference (nVNS, −1.2; sham, −1.0). The percentage of patients who had a response (pain score=0 or 1) for ≥50% of treated attacks at 15 minutes was significantly higher with nVNS in the total cohort (nVNS, 40%; sham, 14%; *P*<0.01) and the eCH subgroup (nVNS, 64%; sham, 15%; *P*<0.01) but not in the cCH subgroup (nVNS, 29%; sham, 13%). A similar percentage of patients in each treatment group had ≥1 adverse device effect (ADE; nVNS, 18%; sham, 19%); no serious ADEs were reported.

**Conclusions**

Acute nVNS treatment was superior to sham in the eCH subgroup but not in the cCH subgroup or total cohort, 71% of whom had cCH. These results confirm the efficacy and safety of acute nVNS treatment for eCH.

**Acknowledgements**

This study was sponsored by electroCore, LLC. We present this abstract on behalf of the ACT2 Study Group.

**Trial registration**

NCT01958125.

### P141 Integrating*Learning to Cope with Triggers*into a cognitive behavioural therapy program for primary headaches: Can it enhance efficacy?

#### Paul R Martin^1^, John Reece^2^, Sharon Mackenzie^1^, Siavash Bandarian-Balooch^1^, Arissa Brunelli^1^, Peter J Goadsby^3^

##### ^1^School of Applied Psychology and Menzies Health Institute Queensland, Griffith University, Mount Gravatt, Queensland, Australia, 4122;^2^School of Psychological Science, Australian College of Applied Psychology, Melbourne, Victoria, Australia, 3000;^3^King’s College London, Institute of Psychiatry, United Kingdom

###### **Correspondence:**Paul R Martin (paul.martin@griffith.edu.au)

**Background**

In a series of reviews we have argued against the traditional advice of counselling avoidance of the triggers of headache and migraine [1,2]. Problems with this approach include the danger of sensitising the headache sufferer to the trigger. We have developed an alternative approach called *Learning to Cope with Triggers* (LCT) that involves exposure to some triggers with the goal of desensitisation, whilst retaining avoidance of triggers that are detrimental to health and wellbeing. We have demonstrated this approach to be superior to counselling avoidance of triggers in a randomised controlled trial [3]. Trigger management is only one component of a comprehensive treatment program for headaches and so we have integrated LCT into a cognitive behavioural therapy (CBT) program of proven efficacy [4].

**Methods**

This study compared the new integrated program (LCT/CBT), with CBT combined with advice to avoid triggers (Avoid/CBT), and a waiting-list control condition (WL), and the protocol has been published [5]. Participants were included if they had suffered from migraine or tension-type headache over a period of at least 12 months. 116 participants, aged 18 to 75 years, were randomly allocated to the three conditions, and 87 completed the post-treatment assessment. Treatment consisted of 12 60-minute sessions scheduled weekly. The main measures used were derived from a validated e-diary [6]. The trial was registered with the Australian and New Zealand Clinical Trials Registry (ACTRN12614000435684).

**Results**

The primary measure was frequency of headaches and the following reductions were recorded from pre- to post-treatment: LCT/CBT, 52.1%; Avoid/CBT, 41.4%; and WL, 16.2%. An Analysis of Covariance on this data revealed a highly significant difference between the three groups, *F* = 7.19(2, 83), *p*<.001. Bonferroni adjusted post hoc pairwise comparisons showed significant differences between LCT/CBT and WL (*p*<.001), and Avoid/CBT and WL (*p*<.05), with the difference between the two treatment groups failing to reach significance. This pattern of results, with LCT/CBT associated with the largest change but no significant difference between the treatment groups was repeated for the secondary measures of peak headache intensity and average headache intensity. However, for the measure of average headache duration, LCT/CBT significantly differed from WL (*p*<.05) whereas Avoid/CBT did not significantly differ from WL.

**Conclusions**

This exploratory randomised controlled trial provided some support for using LCT rather than avoidance as the trigger management component of CBT, but further research with a larger sample is needed to consolidate the findings.

**Acknowledgements**

This study was funded by the National Health & Medical Research Council of Australia (Number: 1046745).

**References**

1. Martin PR, MacLeod C. Behavioral management of headache triggers: Avoidance of triggers is an inadequate strategy. Clinical Psychology Review. 2009; 29:483-495.

2. Martin PR. Managing headache triggers: Think ‘coping’ not ‘avoidance’. Cephalalgia. 2010; 30:634-637.

3. Martin PR, Callan M, Reece J, MacLeod C, Kaur A, Gregg K, Goadsby PJ. Behavioral management of the triggers of recurrent headache: A randomized controlled trial. Behaviour Research and Therapy. 2014; 61:1-11.

4. Martin PR, Forsyth MR, Reece, J. Cognitive-behavioral therapy versus temporal pulse amplitude biofeedback training for recurrent headache. Behavior Therapy. 2007; 38:350-363.

5. Martin PR, Mackenzie S, Bandarian-Balooch S, Brunelli A, Broadley S, Reece J, Goadsby, PJ. Enhancing cognitive-behavioural therapy for recurrent headache: Design of a randomised controlled trial. BMC Neurology. 2014; 14:233.

6. Bandarian-Balooch S, Martin PR, McNally B, Brunelli A, Mackenzie S. Electronic-Diary for Recording Headaches, Triggers and Medication Use: Development and Evaluation. Headache. In press.

### P142 Sleep Disturbance in Children with primary headache

#### Ayse Kartal (kartalays@gmail.com)

##### Department of Child Neurology, Selçuk University Faculty of Medicine, Konya

**Objective**: The aim in the present study was to investigate the prevalence of sleep disturbances in children with migraine and TTH using a validated sleep screening instrument, and the relationship between sleep disturbances and headache features (eg, frequency, duration, intensity). A relationship between headaches and sleep disturbances has been suggested in both children and adults, but there exists limited data of the specific headache features and the range of sleep behaviors in children.

**Methods**: One hundred ninety children aged 6 to 18 years (mean, 13.2; standart deviation, 2.6) were evaluated for headaches at Selcuk University Pediatric Neurology Department (60% migraine; 40% tension-type headache diagnoses) (Table 1). Parents completed the Sleep Disturbances Scale for Children (SDSC) and a standardized questionnaire regarding headache characteristics.

**Results**: Children with migraine scored significantly higher than children with tension type headache on Total SDSC and five SDSC subscales scores -difficulty in initiating and maintaining sleep, disorders of arousal/nightmares, sleep-wake transition disorders, disorders of excessive somnolance, and sleep hyperhidrosis. No difference was found for the Sleep Breathing Disorders subcale scores (Table2). Statistically significant relationships between headache characteristics (eg, frequency, pain intensity) and children sleep behaviors also emerged (Table 3).

Sleep disorders were frequent in all age groups, with no significant difference in preva-lence or total scores of the SDSC, ASWS, PSQI, or ESS between the patients and the controls.

In T1D children, SDSC score was significantly higher in those using continuous glucose moni-toring (CGM) vs glucose meters (P = .042). The score of disorders related to “initiating and maintaining sleep” was significantly higher in those treated with pumps vs patients treated with injections (P = .014), in those using CGM vs glucose meters (P = .02), and in those with noctur- nal hypoglycemia vs those without (P = .023). The percentage of children with excessive day- time sleepiness was significantly lower in patients vs controls (P = .035). No significant differences were found in the other two age groups.

**Conclusions**: Children with migraine headaches have a high prevalence of sleep disturbances. This information may provide further understanding of the nature and course of the patient’s headache experience, as well as facilitate treatment planning to include recommendations for promoting good sleep hygiene. Sleep disorders should be routinely queried and appropriate advice on sleep hygiene provided.

**Table 1 (abstract P142).** Characteristics of the Study Population

	Migraine n (%)	TTH n (%)	Total n (%)	P value
Age years	13.2±2.6	13.7±2.6		0.206^a^
Gender				
Female	85 (74.6)	49(64.5)	134 (70.5)	0.183^b^
Male	29 (25.4)	27 (35.5)	56 (29.5)	
Localisation				
Unilateral	46 (40.4)	40 (52.6)	86 (45.3)	
bilaterally	68 (59.6)	36 (47.4)	104 (54.7)	
Nausea	109 (95.6)	5 (6.6)	114 (60	<0.001^c^
Vomiting	42 (36.8)	2 (2.6)	44 (23.2)	<0.001^c^
photophobia	108 (94.7)	40 (52.6)	148 (77.9)	<0.001^c^
phonophobia	110 (96.5)	53 (69.7)	163 (85.8)	<0.001^c^
osmophobia	60 (52.6)	17 (22.4)	77 (40.5)	<0.001^c^
allodynia	77 (67.5)	25 (32.9)	102 (53.7	<0.001^c^
Headache frequency mean (SD)	8.6±7.8	6.3±5.8		
1 per month	11 (9.6)	13 (17.1)	24 (12.6)	0.006^d^
1 per week	19(16.7)	22 (28.9)	41 (21.6)	
2-3 per week	49 (43.0)	27 (35.5)	76 (40)	
>3 per week	26 (22.8)	10 (13.2)	36 (18.9)	
Daily	9 (7.9)	4 (5.3)	13. (6.8)	
Headache duration (h) mean (SD)	9.2±8.8	2.6±1.8		
<1 h	5 (4.4)	39 (51.4)	44 (25.2)	0.006^d^
1-6	47 (41.2)	28 (36.8)	75 (39.5)	
6-12	60 (52.6)	8 (10.5)	68 (25.8)	
>12	2 (1.8)	1 (1.3)	3 (1.6)	
Pain severity, 0-10 scale, mean (SD)	6.2±5.8	3.4±2.1		
Grade 1	2 (1.8)	16 (21.1)	18	0.001^d^
Grade II-III	27 (23.6)	56 (73.6)	83 (	
Grade IV	85 (74.6)	4 (5.3)	89	

^a^Student's t test

^b^Continuity-Adjusted Chi-Square Test

^c^Chi-square test

^d^Mann Whitney U test

**Table 2 (abstract P142).** Total Sleep Disturbance Score and Subscale Scores for Cases With Migraine and TTH

	Migraine (n=114)	TTH (n=76)	p-value
DIMS			
*mean±SD*	15.5±4.8	13.3±3.9	<0.001^a^
*T score >70, n (%)*	46 (40.4)	18 (23.7)	0.017^b^
SBD			
*mean±SD*	3.9±1.5	3.9±1.7	>0.999^a^
*T score >70, n (%)*	9 (7.9)	6 (7.9)	1.000^c^
DA			
*mean±SD*	4.7±1.8	4.0±1.4	<0.001^a^
*T score >70, n (%)*	30 (26.3)	10 (13.2)	0.046^c^
SWTD			
*mean±SD*	11.8±4.1	9.7±3.7	<0.001^a^
*T score >70, n (%)*	39 (34.2)	10 (13.2)	0.002^c^
DOES			
*mean±SD*	13.1±4.8	10.0±4.1	<0.001^a^
*T score >70, n (%)*	59 (51.8)	19 (25.0)	<0.001^b^
SHY			
*mean±SD*	3.6±2.4	2.7±1.3	<0.001^a^
*T score >70, n (%)*	17 (14.9)	2 (2.6)	0.012^c^
TOTAL			
*mean±SD*	52.6±11.9	43.5±9.4	<0.001^a^
*T score >70, n (%)*	59 (51.8)	17 (22.4)	<0.001^b^

*TTH* Tension type headache

^**a**^Student's t test

^**b**^Pearson chi-square test

^**c**^Continuity-Adjusted Chi-Square Test

### P143 Validity of the ICHD-IIIb criteria in the diagnosis of migraine with aura in children and adolescents

#### Martina Balestri, Alessandro Capuano, Laura Papetti, Samuela Tarantino, Barbara Battan, Federico Vigevano, M. Valeriani

**Objective.** Though common in pediatric age, migraine with aura (MA) has been scarcely studied in children. Our main aim was to test whether the International Classification of Headache Disorders criteria 3^rd^ edition (ICHD-IIIb) are useful to diagnose MA in children and adolescents. Moreover, we aimed also at investigating: 1) the clinical characteristics of the aura in a cohort of MA children, and 2) the features of the headache associated with the aura.

**Methods.** The present study was based on data retrospectively collected from 164 MA children referred to our 3rd level Headache Centre.

**Results.** In our patients, aura mainly included visual symptoms, which were far more frequent (93%) than somatosensory, motor, and speech disturbances. Aura preceded the headache onset in most cases (69.1%) and its duration ranged from 5 to 60 minutes. We divided our patients in 4 different age groups (less than 7 years, between 7 and 10 years, between 11 and 14 years, more than 14 years). No difference in the aura characteristics was found between the groups. On the other hand, when the headache type was classified according to the ICHD-IIIb criteria, migraine was diagnosed only in 40.2% of patients and the diagnosis remained undetermined in 4.3% of children. However, if headache duration was not considered, the headache could be classified as migraine in 67% of patients and in no child the diagnosis was undetermined.

**Conclusions.** Our pediatric population showed aura features that did not depend on the age and were similar to those of adult patients. Although the headache type was difficult to be classified if headache duration was considered, the new criteria reduce the importance of the headache type associated with the aura, thus allowing the diagnosis of MA also in children and adolescents.

### P144 Headache and personality assessed with the NEO-Five-Factor-Inventory

#### Louise S Mose^1, 2^, Susanne S Pedersen^3, 4^, Rigmor H Jensen^5^, Bibi V Gram^2^

##### ^1^Department of Neurology, Hospital South West Jutland, Esbjerg, Denmark;^2^Department of Regional Health Research, University of Southern Denmark/ Hospital South West Jutland, Esbjerg Denmark;^3^Department of Psychology, University of Southern Denmark, Odense, Denmark;^4^Department of Cardiology, Odense University Hospital, Odense, Denmark;^5^Danish Headache Centre, Department of Neurology, Rigshospitalet-Glostrup, University of Copenhagen, Denmark

###### **Correspondence:**Louise S Mose (Louise.schlosser@rsyd.dk)

**Background**

The association between personality and headache has received considerable interest in the literature. However, findings on the role of personality in the development of headaches across diagnoses are inconsistent. Knowledge of the role of personality could potentially be useful in order to develop individually tailored treatment. We investigated if the NEO Five-Factor-Inventory (NEO-FFI-3) personality questionnaire can be used to differentiate between migraineurs and patients with medication overuse headache (MOH), and whether patients with either of the two headache diagnoses have a different personality profile as compared to a representative Danish group of healthy adults (RG).

**Materials and Methods**

Eighty migraine patients without medication overuse and 80 MOH patients seen at the Hospital of South West Jutland, Denmark, and matched on age, completed the NEO-FFI-3 questionnaire. The questionnaire is standardized and validated and taps into the five basic personality domains; 1)*Neuroticism* covering inter alia emotional stability, 2)*Extraversion* including the interaction with people, 3)*Openness* which includes tendency to be creative and imaginative, 4)*Agreeableness* which encompasses traits regarding emphatic capability, and 5)*Conscientiousness* covering capacity of behavioral and cognitive control. Unpaired t-tests were used to compare the personality profiles of the two headache groups and also to the RG group with 1032 healthy adults.

**Results**

A significant greater proportion of men were identified in the MOH group 32.5% vs. migraine 12.5% (p=0.002). There were no statistically significant differences between the two headache groups with respect to personality. However, compared to the RG group both headache groups had significantly lower Extraversion scores (MOH: 27.5±8.5, RG: 29.8±6.2, p=0.002 vs. Migraine: 26.6±7.3, RG: 29.8±6.2, p<0.001) and significantly higher conscientiousness scores (MOH: 34.5±6.2, RG: 31.5±6.5, p<0.001; Migraine: 34.8±6.0, RG: 31.5±6.5, p<0.001). Furthermore migraineurs had significantly higher Agreeableness scores (Migraine: 31.5±6.9, RG: 29.9±5.5, p=0.02). There were no statistically significant differences between the three groups on Neuroticism and Openness.

**Conclusion**

We found no differences in personality between migraineurs and MOH patients using the NEO-FFI-3. This may partly be attributed to the fact that MOH often develops from migraine. Compared to the RG group, headache patients had lower score on Extraversion which may be construed as they are more introvert. Additionally, headache patients scored higher on Conscientiousness, suggesting that they have a high need for behavioral and cognitive control. Migraineurs’ high Agreeableness score indicated that they are emphatic. If confirmed in larger studies, this in-formation could be used in patient education and the management of headache patients in clinical practice.

### P145 Botulinum toxin A in the Treatment of Chronic Cluster Headache – a pilot study

#### Christian Lampl, Mirjam Rudolph, Elisabeth Bräutigam

##### Headache Medical Center, Seilerstätte, Ordensklinikum Linz Barmherzige Schwestern, Austria

**Background**

For the treatment of refractory Chronic Cluster Headache (rCCH) only limited treatment options are available. The objective of this open single-centre study was to evaluate the efficacy and tolerability of Botulinum Toxin Type-A (BTX-A) as add-on therapy in the prophylactic treatment of rCCH.

**Methods**

rCCH is described as a situation that fulfills the criteria of ICHD-3 beta for CCH^(1)^ with at least three severe attacks per week despite at least three consecutive trials of adequate preventive treatments^(2)^. Between March 2014 und September 2016 rCCH patients, 18-65 years of age, were identified. Exclusion criteria were the standard ones for BTX therapy. According to the PREEMPT I und II study protocol^(3,4)^ 150 Allergan IU were administered. Treatment period was 48 weeks. We compared the sum of headache minutes per headache day at baseline (last 4 weeks before BTX treatment) vs week 12, 24, 48.

**Results**

We identified 15 patients, age 41±6 (mean±SD) years, mean duration of the disease was 11,7 years. 7 patients experienced total cessation of attacks within the first 3 treatment periods; in 5 patients the primarily chronic form changed into an episodic one with improvement in attacks frequency and intensity. 3 patients did not improve.

**Conclusion**

Our data suggest that the injection of BTX -A could be beneficial as add-on in some patients with otherwise drug rCCH. Usefulness of BTX-A as a new alternative therapeutic tool in the treatment of rCCH has to be confirmed in double-blind, ran- domised, controlled studies.

**References**

1. Headache Classification Committee of the International Headache Society (IHS). The International Classification of Headache Disorders, 3rd edition (beta version) Cephalalgia 2013; 33: 629-808.

2. Mitsikostas DD1, Edvinsson L, Jensen RH, Katsarava Z, Lampl C, Negro A, Osipova V, Paemeleire K, Siva A, Valade D, Martelletti P. Refractory chronic cluster headache: a consensus statement on clinical definition from the European Headache Federation. J Headache Pain 2014; 15:79.

3. Aurora S K, Dodick D W, Turkel C C et al. OnabotulinumtoxinA for treatment of chronic migraine: results from the double-blind, randomized, placebo-controlled phase of the PREEMPT 1 trial. Cephalalgia 2010; 30:793-803.

4. Diener H C, Dodick D W, Aurora S K et al. OnabotulinumtoxinA for treatment of chronic migraine: results from the double-blind, randomized, placebo-controlled phase of the PREEMPT 2 trial. Cephalalgia 2010; 30:804-814.

### P146 Characteristics of 5000 patients in a headache clinic registry

#### David García-Azorín, Marina Ruiz, María I Pedraza, Laura Blanco, Raquel Moreno, Angel L Guerrero

##### Headache Unit. Neurology Department. Hospital Clínico Universitario de Valladolid

**BACKGROUND:** The burden of different types of headache varies when considering epidemiological studies or registries based on primary care, general neurology or headache clinic series. We aimed to analyze characteristics of first five thousand patients of a Headache Clinic registry and the incidence of their different headaches codified accordingly to International Classification of Headache Disorders (ICHD).

**MATERIALS AND METHODS:** On January 2008 a headache clinic was established in a tertiary hospital. Patients might be sent from general practitioners accordingly to predefined criteria, and also from general neurology and other outpatient hospital physicians. In our registry we prospectively gathered age and sex, referral source, and symptomatic or prophylactic therapies previously prescribed. When a patient fulfilled criteria for more than one type of headache, all of them were diagnosed and classified.

**RESULTS:** In June 2017, 5000 patients (3690 women, 1310 men) had been included. Age at inclusion was 44.7 ± 17.6 years (9-94). In 2950 cases (59%) preventatives had not been previously used. A total of 7120 headaches were diagnosed in the 5000 patients and they were codified accordingly to ICHD-II until March 2013 and ICHD-III beta from them until now. Classification of headaches is hereby presented considering ICHD-III beta. Among primary headaches, 3765 (52.9%) were included in Group 1 (Migraine), 687 (9.6%) in Group 2 (Tension-type headache), 231 (3.2%) in Group 3 (Trigeminal autonomic cephalalgias), and 467 (6.5%) in Group 4 (Other primary headache disorders). Among secondary headaches, the more represented were Group 8 (Headache attributed to a substance or its withdrawal) with 831 (11.6%), Group 11 (Headache or facial pain attributed to disorders of cranial, facial, cervical structures) with 64 (0.9%) and Group 5 (Headache attributed to trauma or injury to the head and/or neck) with 61 (0.9%). 269 (3.7%) headaches were recorded in Group 13 (Painful cranial neuropathies and other facial pains), 286 (4%) in the research appendix and 356 (4.9%) corresponded to Group 14 (Other headache disorders). Considering the transition between ICHD-II and ICHD-III beta, most cervicogenic headaches were diagnosed accordingly with ICHD-III beta criteria, nummular headache cases were reclassified in Group 4 and 70 neuralgias of terminal branches, lost their code in Group 13 and were moved to Group 14.

**CONCLUSION:** Migraine is most frequent diagnosis in headache clinics registries. Transitions between ICHD-II and ICHD-III beta classifications have led to significant changes among non infrequent syndromes. Anyway, most headaches could be codified accordingly to ICHD criteria.

### P147 Life traumas and stressful events in Chronic Migraine and Medication Overuse Headache: What is the relation with the outcome of a detoxification therapy?

#### Bottiroli Sara^1^, Viana Michele^1^, Sances Grazia^1^, De Icco Roberto^1^, Vito Bitetto^1, 2^, Guaschino Elena^1^, Ghiotto Natascia^1^, Pazzi Stefania^1^, Giuseppe Nappi^1^& Tassorelli Cristina^1,2^

##### ^1^Headache Science Centre, C. Mondino National Neurological Institute, 27100 Pavia, Italy;^2^Department of Brain and Behavioral Sciences, University of Pavia, 27100 Pavia, Italy

###### **Correspondence:**Bottiroli Sara (sara.bottiroli@mondino.it)

**Background**

Withdrawal from overused drug is the treatment of choice for subjects with Chronic Migraine and Medication Overuse Headache (CM+MOH) [1, 2], reverting the headache pattern from chronic to episodic within two months in the majority of subjects. Many factors are involved in the prognosis and outcome of these subjects, and their understanding is a topic of interest. CM+MOH patients experience increased psychiatric comorbidity, such as anxiety, depression, or personality disorders [3, 4], even if a cause-effect relationship still needs to be clearly delineated. Even less is known about the role of psychiatric factors in the response to detoxification treatments. In the present study we focused on early traumatic experiences and recent stressful events by investigating their association with the outcome of detoxification in a 2-month follow-up.

**Materials and methods**

This study was conducted at the Headache Center of the C. Mondino National Neurological Institute in Pavia, Italy. All consecutive patients with chronic migraine and medication overuse headache undergoing an inpatient detoxification program were enrolled and followed-up in a prospective study. Diagnosis was operationally defined according to ICHD-IIIβ. The protocol consisted in inpatient detoxification treatment and a 2-month follow-up. Data on early life traumatic experiences – of the physical or emotional type – and recent stressful events – rated according to the impact on quality of life, from mild to very serious – were collected by means of self-report questionnaires. Data were analyzed with the analysis of variance.

**Results**

Of the 166 patients who completed the 2-month follow-up, 118 (71%) stopped overuse and their headache reverted to an episodic pattern (Group A), 19 (11%) kept overusing and did not experience any change in headache frequency (Group B); and 29 (18%) stopped overuse without any benefit on headache frequency (Group C). At the multivariate analyses, a higher number of emotional traumas (OR 11.096; p = 0.037) emerged as a prognostic for the outcome in Group B; whereas having had history of major depression (OR 3.703; p = 0.006) and higher number of very serious stressful events (OR 1.679; p = 0.045) were prognostic for the outcome of Group C.

**Conclusions**

Our findings show the impact of life traumas and stressful events on the outcome of a detoxification program. The failure to cease overuse is related to the existence of childhood (mostly emotional) traumas, whereas recent life events, especially when very serious, do not influence the capacity of the patient to stop overuse, but are associated to the persistence of chronic headache. These observations underscore the need of a thorough psychological assessment of CM+MOH subjects and have possible implications in the nosographic framing of chronic headaches.

**Acknowledgements**

This research was supported by a Grant of the Ministry of Health to Mondino Institute (Current Research 2014-2016)

**Conflicts of interests**

None

**References**

1. Evers S and Jensen R. Treatment of medication overuse headache – guideline of the EFNS headache panel. *Eur J Neurol.* 2011; 18: 1115–1121.

2. Olesen J. Detoxification for medication overuse is the primary task. *Cephalalgia*. 2012; 32:420-422.

3. Sances G et al. Factors associated with a negative outcome of medication overuse headache - a three-year follow-up (the “care” protocol). *Cephalalgia.* 2013, 33: 1-13.

4. Bottiroli S et al. Psychological factors associated to failure of detoxification treatment in chronic headache associated with medication overuse. *Cephalalgia.* 2016; 36: 1356-1365.

### P148 Comorbidities in chronic and episodic migraine

#### Mansoureh Togha^1^, Zahra Yari^1,2^, Reza Rahmanzadeh^1^, Soodeh Razeghi Jahromi^2^, Zeinab Ghorbani^1^

##### ^1^Headache department, Iranian Center of Neurological Research, Neuroscience Institute, Tehran University of Medical Sciences, Tehran, Iran, Islamic Republic Of;^2^School of Nutritional Sciences and Dietetics, Tehran University of Medical Sciences, Tehran, Iran, Islamic Republic Of

###### **Correspondence:**Mansoureh Togha

**Background:** It has been known for years that migraine is comorbid with a number of other diseases, which understanding is so important from a number of different perspectives. Some of these comorbidities are well-known, and others remain neglected such as thyroid disorders. In present study, we aimed to investigate and comprise the most frequent concomitant disorders occurring in two main categories of migraine, chronic and episodic.

**Methods:** This cross-sectional study included 610 migraineurs who referred to a tertiary headache clinic, Tehran, Iran. A total of 370 (60%) episodic migraine and 240 (40%) chronic migraine (with and without aura) were diagnosed according to ICHD III β criteria. 30- day headache diaries were applied in order to collecting data on headache characteristics.

**Results:** The overall mean age of participants was 37.5 ± 11.3. Sixty-six subjects (10.8%) experienced aura. Among patients with chronic migraine, 210 cases (34.4%) suffered from medication overuse headache. No significant difference was seen between two types of migraine, including chronic and episodic, in terms of gastrointestinal diseases (including IBS, constipation, heartburn, peptic ulcer disease, dyspepsia and non-alcoholic fatty liver disease), thyroid disorders (hypo- and hyperthyroid) and having past medical history of surgery (any kinds). However, interesting results were obtained via analyzing the association between number of headache days and comorbidities among episodic migraineurs. After adjusting for potential confounders including age, sex, and intensity of headache in the multivariate regression models, the risk of gastrointestinal complaints among episodic migraineurs with 8-14 days of headache per month is 1.9 fold greater than those with less than 8 days of headache (CI= 1.10-3.17; *P value*= 0.014).

**Conclusion:** The most comorbidities among participants were GI diseases, having surgery and thyroid disorders, respectively. The results of present study indicate that there is no significant difference between chronic and episodic type of migraine regarding comorbidities but considering the number of headache days can be helpful in predicting the odds of gastrointestinal complaint among migraineurs. In other words, the greater the number of headaches days, the more digestive problems occur. These findings represent the importance of migraine management and its effect on general health. Further studies should investigate the etiology of the relationship between frequency and intensity of different subtypes of migraine and its comorbidities.

**Keywords:** Comorbidity, Migraine, Chronic, Episodic, Days of Headache.

### P149 Features of chronic primary headaches (CPH) in children and adolescents referred to two third level headache centers

#### Massimiliano Valeriani^1^, Laura Papetti^1^, Beatrice Bartoli^2^, Cristiano Termine^2^, Irene Salfa^1^, Barbara Battan^1^, Federico Vigevano^1^

##### ^1^Neuroscience, Bambino Gesù Children Hospital, Rome, Italy;^2^Neuropsychiatric, Insubria University, Varese, Italy

###### **Correspondence:**Massimiliano Valeriani (m.valeriani@tiscali.it)

**Background:** Chronic migraine (CM), Chronic tension-type headache (CTTH) and new daily persistent headache (NDPH) are the main forms of CPH reported in the ICHD-III beta version. Medication-overuse headache (MOH) is classified among secondary headache. Our aim was to investigate the clinical features of CPH in a cohort of pediatric patients.

**Materials and Methods:** We retrospectively reviewed the charts of patients attending the Headache Centre of Bambino Gesú Children and Insubria University Hospital. The ICHD-III criteria were used for diagnosis. Statistical analysis was conducted by SPPS version 22.0 and χ2 test was used to study possible correlations between: - CPH and population features (age and sex); - CPH and headache qualitative features; - CPH and risk of MOH; -CPH and response to prophylactic therapies.

**Results:** We included 377 patients with CPH (66.4% female, 33.6% male, age between 0 and 18 years). The most frequent CPH type was CM (73.5%), followed by CCTH (13.5%) and NDPH (13%). MOH was detected in 10.9% of total patients. CPH are less frequent under 6 years of age (0.8%; p <0.05); significant greater frequency in females than in males was found in the age group between 0-6 years (23/31 F, 8/31 M) and between 15-18 years (41/51 F, 10/51 M) (p<0.05). No correlations between age/sex and different CPH types were detected. Possible development of MOH has been found correlated with CM types (p<0.05) and age above 15 years (p<0.05). Our results show that 272 (72.1%) out of 377 CPH patients received a prophylactic therapy. Among them, 190 patients received amitriptyline, 29 patients topiramate, 15 patients L-5 hydroxytryptophan, and 8 patients flunarizine. Thirty patient performed two or more drugs. Positive response to therapy (reduction of attacks by at least 50% in a month) was detected in 54% of patients, while no outcome data were obtained from 29.4% of cases. Amitriptyline and topiramate had the highest efficacy (p<0.05). We found that 59.2% of patients who received amitriptyline showed significant reduction in the attack frequency, while 48.4% patients receiving topiramate improved their headache attack frequency (p>0.05).

**Conclusions:** Our results showed that CPH presented a correlation with patients’ age and sex. No significant differences were found between CPH types and population/pain features. Development of MOH was related with CM onset and adolescent age. Amitriptyline and topiramate had the best effectiveness. However, it is to be underlined that follow up data could not be issued from a moderate percentage of patients.

### P150 Postural control impairment in patients with migraine with and without aura and chronic migraine

#### Gabriela Ferreira Carvalho^1^; Lidiane L Florêncio^1^, Carina Ferreira Pinheiro^1^, Fabiola Dach^2^, Débora Bevilaqua-Grossi^1^

##### ^1^Department of Biomechanics, Medicine and Locomotor Apparatus Rehabilitation –Ribeirão Preto Medical School, University of São Paulo, Ribeirão Preto-SP, Brazil;^2^Department of Neurosciences and Behavioral Sciences – Ribeirão Preto Medical School, University of São Paulo, Ribeirão Preto-SP, Brazil

###### **Correspondence:**Gabriela Ferreira Carvalho

**Background:** Migraine could be associated to subclinical balance impairment due malfunctioning of the vestibular and visual systems. Posturography changes have been demonstrated in such population, specially in migraineurs with aura. However, detailed balance protocol tests and comparison with other forms of migraine, such as chronic migraine, are still lacking.

**Objective:** To investigate the performance of patients with different subtypes of migraine in two clinical balance tests.

**Methods**: Women with migraine were screened from a tertiary headache center and sorted in four groups according with the ICHD-III. It was included migraineurs with aura (n=35), without aura (n=35), chronic migraine (n=35) and headache-free controls (n=35). Subjects with systemic diseases, vestibular disease history, presence of other headache diagnosis, BMI over 30 or any musculoskeletal impairment were excluded. A blinded examiner conducted the Limits of Stability test (LOS) in the Balance Master System (Neurocom®) and the static posturography test on a force platform (AMTI - OR6-7-1000). At the LOS test, subjects were instructed to intentionally move the center of pressure to the maximal distance without changing their support basis in eight directions. The LOS outcomes were reaction time (sec.), velocity (degrees/sec.), maximal excursion (%) and final excursion (%). Posturography displacement area was assessed in four conditions: firm and foam surface with eyes opened and closed (firmOE, firmCE, foamOE and foamCE) three times during 30 seconds each. Groups were contrasted using Ancova analysis with Bonferroni’s *post-hoc* test in the SAS 9.2 software, with a fixed significant level at 5%.

**Results:** Patients with migraine presented slower LOS reaction time for all directions, with differences between all the three migraine groups compared to controls for 5 of 8 directions, p<0.03 (Fig. 1). The LOS velocity was greater in controls compared to all migraine groups for 6 of 8 directions (p<0.04). Furthermore, for the left direction, it was observed differences between migraineurs with and without aura, p=0.04 (Fig. 1). The LOS maximal excursion was greater for all directions and the final excursion for 5 of 8 directions in controls compared to all migraine groups, p<0.02 (Fig. 1). At the condition firmOE of the posturography test, it was not observed differences among groups. At the firmCE condition, migraine with aura and chronic migraine groups presented larger displacement area than controls (p<0.02). For the both foamOE and foamCE conditions, migraine with aura and chronic migraine groups also presented larger displacement area than controls (p<0.005) and than migraineurs without aura (p<0.01) (Table 1).

**Conclusion:** These results highlight the presence of significant changes of the limits of stability and static balance in migraineurs, mostly in chronic migraine and migraine with aura, for almost all outcomes assessed. The balance impairment may have functional impact and it might be addressed with proper clinical interventions.

**Fig. 1 (abstract P154).**
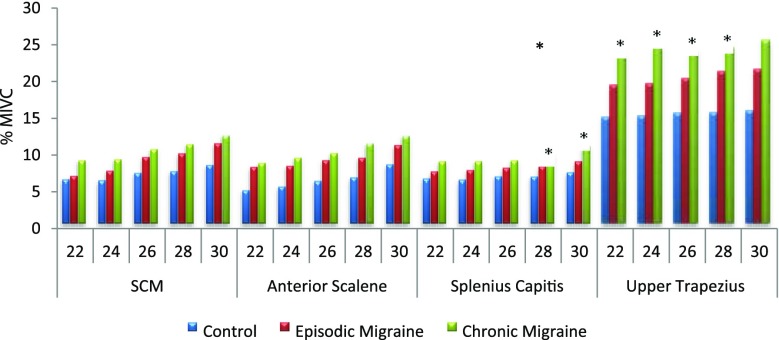
Normalized activity of the neck muscles during performance of a low load test named craniocervical flexion test. *p<0.05

**Table 1 (abstract P150).** Average and 95%CI of the posturography displacement area (cm^2^) in controls (CG), patients with migraine without aura (MoA), with aura (MA) and chronic migraine (CM)

	CG	MoA	MA	CM
FirmOE	1.20 (1.05 to 1.34)	1.52 (1.26 to 1.77)	2.47 (1.97 to 2.97)	3.23 (2.21 to 4.24)
FirmCE	1.53 (1.31 to 1.74)^*^	2.10 (1.61 to 2.59)	4.49 (1.97 to 2.97)	4.53 (2.21 to 4.24)
FoamOE	5.09 (4.63 to 5.56)^*^	5.58 (5.03 to 6.14)^**^	8.75 (7.31 to 10.18)	8.82 (7.66 to 9.97)
FoamCE	14.82 (13.72 to 15.92)^*^	17.27 (15.39 to 19.14)^**^	21.87 (19.09 to 24.66)	22.44 (19.92 to 24.95)

*Firm* firm surface, *Foam* foam surface, *OE* opened eyes, *CE* closed eyes

^*^p<0.02 GC *versus* MA e MC; ^**^p<0.01 M *versus* MA e MC

### P151 Perivascular neurotransmitters: Role in primary headaches

#### Simona D Frederiksen^1^, Kristian A Haanes^1^, Karin Warfvinge^1,2^, Lars Edvinsson^1,2^

##### ^1^Department of Clinical Experimental Research, Glostrup Research Institute, Rigshospitalet Glostrup, Glostrup, Denmark;^2^Department of Clinical Sciences, Lund University, Lund, Sweden

###### **Correspondence:**Simona D Frederiksen (simona.denise.frederiksen@regionh.dk)

In order to understand the nature of the relationship between cerebral blood flow (CBF) and primary headaches, we have conducted a literature review to assess the current findings in this area, with a particular emphasis on the role of perivascular neurotransmitters in both phenomena. Migraine, a primary headache, has been associated with increased CBF in the somatosensory cortex, brainstem and thalamus during different phases of the attack, imposing a risk for alterations in vascular shear stress. Elevated CBF has also been observed in headache-free periods possibly as a response to functional modifications. The basic regulation of CBF is known to be regulated through autoregulatory, chemical, metabolic and neurogenic factors which are crucial for optimal brain homeostasis. During headache attacks, ganglia such as the trigeminal and sphenopalatine ganglia (located outside the blood-brain barrier) are variably activated and sensitized which give rise to vasoactive neurotransmitter release and secondarily activates innervation of the cerebral vasculature. The innervation of the cerebral vasculature is via sympathetic (ATP, noradrenaline, neuropeptide Y), parasympathetic (acetylcholine, VIP [vasoactive intestinal peptide]/peptide histidine methionine, PACAP [pituitary adenylate cyclase-activating polypeptide], NO [nitric oxide]) and sensory nerves (CGRP [calcitonin gene-related peptide], substance P/NKA [neurokinin A]/NKB [neurokinin B], PACAP, NO). Their individual roles in regulation of CBF are partly known but much needs to be unraveled in headache disorders. Acute attacks of migraine and cluster headache are both associated with CGRP and PACAP release with the additional release of VIP in cluster headache. Even during headache-free periods, the neurotransmitter levels are elevated in some studies during episodic and chronic migraine compared to healthy human subjects. Pathophysiological alterations (e.g. functional and structural changes, release of neurotransmitters and inflammatory markers) are more pronounced in chronic headache and a link between headache frequency and chronification has been observed. Another possible link between headaches and vascular function comes from recent genome-wide association studies investigating migraine and cluster headache. Several underlying candidate headache genes (e.g. HTRA1, JAG1, RNF213 and HCRTR2) are also implicated in vascular disorders/diseases, regulation of vascular tone and smooth muscle contractility. Together these findings support a role for the vasculature in primary headache disorders.

**Acknowledgement**

The work was supported by grants from the Lundbeck foundation (Denmark), the Swedish research council (grant no 5958) and the Heart-Lung foundation.

### P152*Headache attributed to aeroplane travel*and*Aereoplane headaches sine aereoplane travel*: Headache attributed to imbalance between intrasinusal and external air pressure

#### Federico Mainardi^1^, Ferdinando Maggioni^2^, Giorgio Zanchin^2^

##### ^1^Headache Centre, Neurological Division, SS Giovanni e Paolo Hospital, Venice, Italy;^2^Headache Centre, Department of Neurosciences, Padua University, Padua, Italy

###### **Correspondence:**Federico Mainardi (fmainardi@iol.it)

**Background**: Our previous survey on *Headache attributed to aeroplane travel* (AH) [1], which is currently codified in Chapter 10.1.2 of the International Classification of Headache Disorders 3 beta [2], has been extended to 200 subjects.

Material and methods: Following a preliminary contact by mail, people affected by AH agreed to fill in a comprehensive anonymous questionnaire which aimed at a further detailed description of the AH characteristics.

**Results**: Most of the previously reported clinical profile of AH (including duration of the acute phase and headache features) was confirmed, while other aspects were partially modified. In particular: 1. A milder, postictal headache phase persisted after the remission of the severe pain in (55 patients, 27.5%); 2. Associated symptoms during AH attacks were reported by 59 (29.5%), the most common being restlessness (n=40) and ipsilateral tearing (n=28).

A consistent number of patients reported the occurrence of headache sharing the identical AH features, but triggered by ascent during free/snorkeling or scuba diving (21 among the 46 subjects who reported a diving experience) and the rapid descent by car from high mountain altitude (n=20, 16.6%).

**Discussion**: The extension of the sample confirmed most of our previous observations on AH clinical features, and it allowed to identify subgroups of sufferers who presented different conditions in which AH-like attacks could occur, that is ascent during free/snorkeling or scuba diving (*Diving ascent headache, DAH*) and rapid descent by car from high mountain altitude (*Mountain descent headache, MDH*). The shared denominator between the three different circumstances appears to be the imbalance between intrasinusal and external air pressure which could be considered, therefore, as the common causal condition.

**Conclusion**: The coexistence of AH, DAH and MDH and the identical clinical features of the respective attacks suggests to gather them together, within the Chapter 10^th^ “*Headache attributed to disorders of homoeostasis”,* in an unique heading “*Headache attributed to imbalance between intrasinusal and external air pressure”* in the next revision of ICHD.

**References**

1. Mainardi F, Lisotto C, Maggioni F et al. Headache attributed to airplane travel (“airplane headache”): clinical profile based on a large case series. Cephalalgia. 2012, 32: 592-599.

2. Headache Classification Committee of the International Headache Society. The International Classification of Headache Disorders: 3^rd^ edition (beta version). Cephalalgia. 2013; 33: 629-808.

### P153 Thunderclap headache caused by spontaneous pneumocephalus associated with intracranial hypotension

#### Angela Cervellino^1^, Valeria Coppola^2^, Antonietta Romaniello^1^, Giovanni Gonnella^1^, Nicola Paciello^1,^Enrico Ferrante^1^

##### ^1^Neurology and^2^Neuroradiology Department, AOR San Carlo, Potenza, Italy

###### **Correspondence:**Enrico Ferrante (enricoferrante@libero.it)

**BACKGROUND:** Thunderclap headache (TH) is defined as a paroxysmal and excruciating severe headache presenting with extreme abruptness [1]. Spontaneous pneumocephalus (SP) is the accumulation of intracranial air in the absence of pathological condition or iatrogenic intervention [2]. The typical causes of SP are barotraumas, Valsalva manoeuvers, adjiacent air sinus infections, bacteriemia and air cell hyperpneumatization. The developmental mechanism of pneumocephalus is mainly based on two factors: a reduction in intracranial pressure and the presence of a defect in the dura. It is caused by either a ball-valve mechanism that allows air to enter but not to exit or by CSF leakage which creates a negative pressure with subsequent air entry.

Spontaneous intracranial hypotension (SIH) is characterized by orthostatic headache, low CSF pressure and diffuse pachymeningeal enhancement (DPE) on brain MRI. SIH results from spontaneous CSF leakage. Although the spontaneous leak may occur at the level of the skull base, in the large majority of patients, it occurs at the spinal level [3].

**CASE REPORT:** A 68- years-old man presented to our department complaining of sudden severe headache with nausea, vomiting, and confusion, worsening by upright position, movement and Valsalva manoeuvers. He had no recent history of trauma, head surgery or lumbar puncture. He reported having flu with frequent rhinorrea and nose blowing and sneezing. Brain CT revealed a large bilateral frontal pneunocephalus. He was prescripted bed rest in Trendelenburg position, overhydration and analgesics with gradual headache disappearance over several days. Brain MRI showed bilateral chronic subdural hematoma, DPE compatible with SIH. Spinal MRI failed to identify the CSF leak site. The possible site of air leak into the brain was thought to be the posterior wall of the left frontal sinus. The facial bones CT showed a mucous membrane thickening of the maxillary sinus and the mastoid air cells, and a thinning of the internal wall of the left frontal sinus with a possible small bone defect, without hyperpneumatization. The patient was discharged four weeks after the development of pneumocephalus. After one month follow-up he was asymptomatic and brain MRI showed pneumocephalus resolution.

**CONCLUSIONS:** To the best of our knowledge, this is the first case described in literature reporting spontaneous pneumocephalus associated with SIH causing TH. In these cases if there are non signs of infection or dural defect, the conservative management (bed rest in Trendelenburg position and overhydration) would be the treatment of choice.

**Consent for publication:** The authors declare that written informed consent was obtained for publication.

**References**

1. Ferrante E, Tassorelli C, Rossi P, Lisotto C, Nappi G (2011) Focus on the management of thunderclap headache: from nosography to treatment J Headache Pain 12:251–258

2. Pishbin E, Azarfardian N, Salarirad M, Ganjeifar B (2015) Spontaneous nontraumatic pneumocephalus : a case report. Iran Red Crescent Med J. July; 17 (7): e 23920.

3. Ferrante E, Arpino I, Citterio A, Wetzl R, Savino A (2010) Epidural blood patch in Trendelenburg position pre-medicated with acetazolamide to treat spontaneous intracranial hypotension. Eur J Neurol 17:715–719.

### P154 Women with migraine present hyperactivity of neck extensors muscles during maximal and submaximal tasks

#### Lidiane L. Florencio^1^, Gabriela F. Carvalho^1^, Fabiola Dach^2^, Anamaria Siriani de Oliveira^1^, César Fernández-de-las-Peñas^3^, Débora. Bevilaqua-Grossi^1^

##### ^1^Department of Biomechanics, Medicine and Locomotor Apparatus Rehabilitation –Ribeirão Preto Medical School, University of São Paulo, Ribeirão Preto-SP, Brazil; ^2^Department of Neurosciences and Behavioral Sciences – Ribeirão Preto Medical School, University of São Paulo, Ribeirão Preto-SP, Brazil; ^3^Department of Physical Therapy, Occupational Therapy, Physical Medicine and Rehabilitation, Universidad Rey Juan Carlos, Alcorcón, Spain

###### **Correspondence:** Lidiane L. Florencio

**Background:** Despite of the confirmed association between neck pain and migraine investigations of neck muscles controls in this population is scarce. The aim of this study was to compare activity of neck muscles in women with migraine, chronic migraine and non-headache women during low load and maximal contractions.

**Materials and methods:** Thirty one women without history of headache (age: 31 years old); 31 with migraine (age: 33 years old) and 21 with chronic migraine (age: 34 years old), diagnosed according to the third edition of International Headache Classification, were assessed. Exclusion criteria were the presence of concomitant headaches, pregnancy, history of head or neck trauma, cervical spine diseases, fibromyalgia syndrome, medication overuse, or anesthetic blocks in the past month. The submaximal task performed was composed by five progressive stages of the craniocervical flexion test using a pressure biofeedback unit (Stabilizer, Chattanooga South Pacific). After a 15 min of rest, they performed three repetitions, sustained for 3s, of maximal isometric voluntary contraction (MIVC) of the neck in flexion and extension. Surface electromyography of sternocleidomastoid, anterior scalene, splenius capitis and upper trapezius muscles were acquired using a Trigno TM Wireless System (Delsys Inc). Sensors were positioned by a blind examiner after proper skin cleaning according to standard recommendations. Raw signal was filtered (4dB; band pass 20-500Hz) and Root Mean Square (RMS) was calculated by a customized MATLAB code. Muscles activity during submaximal task was normalized by the RMS of MIVC and; antagonist activity was normalized by its own activity while acting as agonist during MIVC. Groups were compared using ANOVA with Bonferroni’s test as post hoc by the SPPS version 20.0 adopting a significance level of 0.05.

**Results:** In the low load task, chronic migraine group demonstrated greater activity of upper trapezius in all stages of the craniocervical flexion test and greater activity of splenius capitis in the last two stages compared to control (p<0.001) (Fig. 1). No differences could be observed between migraine and control groups not even among three groups regarding superficial flexors activity (Fig. 1). Extensors activities of both migraine groups were significant higher than controls during MIVC in flexion (p=0.03), but no differences were observed among groups during MIVC in extension (Fig. 2).

**Conclusion:** Migraine is associated with higher activity of neck extensor muscles during maximal tasks. However, for low load tasks, this pattern of hyperactivity of neck extensor can be observed only in chronic migraine group.

**Fig. 2 (abstract P154).**
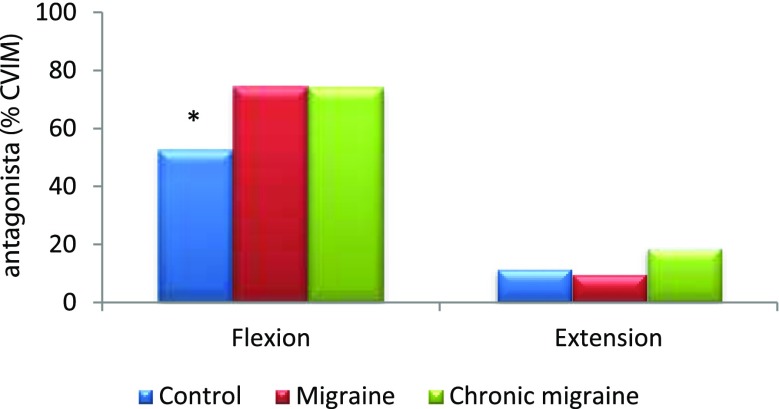
Normalized activity of the antagonist muscles during maximal isometric voluntary contraction (MIVC) in flexion and extension. *p<0.05

**Fig. 1 (abstract P157).**
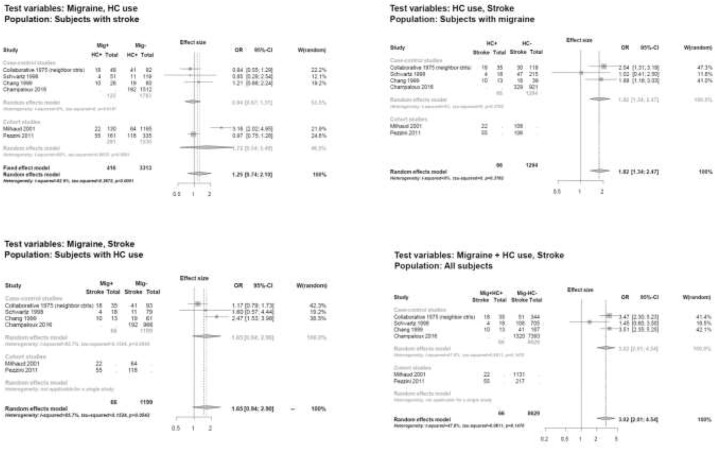
Forest plots of the meta-analyses

### P155 The role of sexual dysfunction and attachment style in migraine related quality of life

#### Maria Pia Prudenzano^1^, Maria Fara De Caro^1^, Maria Colucci^1^, Jolanda Losole^1^, Simona Lamberti^1^, Maria Elena Roca^1^, Domenico Laera^1^, Alessandro Taurino^2^, Maria Trojano^1^

##### ^1^Department of Basic Medical Sciences, Neuroscience and Sense Organs University of Bari; ^2^Department of Education, Psychology, Communication University of Bari

###### **Correspondence:** Maria Pia Prudenzano (centrocefalee.neurologia@uniba.it)

**Background**: Migraine is a disabling condition affecting quality of life and social relations worldwide. To date few studies have focused on the occurrence and possible role of sexual dysfunction in migraine patients quality of life [1,2]. Attachment behaviour is supposed to influence psychological and physical well-being, coping, illness behaviour and social functioning [2].

**Objectives**: This study was conducted to investigate the association among sexual dysfunction, psychological factors, attachment style and quality of life, in a sample of migraine patients.

**Patients and Methods**: 70 adult migraine outpatients (14 males; 56 females, mean age 39,56±12,55) consecutively referred to Headache Centre, were recruited and submitted to: a) the International Index of Erectile Function (IIEF) for male patients, b) the Female Sexual Function Index (FSFI) for female patients, c) 36-Item Short Form Health Survey (SF-36), d) Symptom Check List 90 Response (SCL-90R), e) Attachment Style Questionnaire (ASQ).

**Results:** FSFI- IIEF Sexual dysfunction was found in 54,30% of the whole sample (males: 42,85% vs females: 57,14%, p=0,38), without difference according to age, marital status, job and migraine type (with or without aura). Sexual Dysfunction was observed more frequently in chronic migraine patients than in episodic ones (73,07% vs 40,62%, p=0.02).

Migraine patients with sexual dysfunction showed significantly lower scores in SF-36 Mental Component Summary (41,52+10,88 vs47,12+9,41, p=0,02) and in ASQ Confidence subscale (31,39+8,15 vs 35,87±5,55, p=0,01). No difference was found between patients with and without Sexual dysfunction both in SCL-90R Anxiety (57,93±12,26 vs 51,69+14,37, p=0,07) and Depression (53,93±15,80 vs 46,79+12,71, p=0,08) subscales scores.

A positive bivariate correlation was observed in the whole sample between SF-36 Mental Component Summary and ASQ Confidence subscale(r=0,37, p=0,001) whereas a negative correlation was found between SF-36 Mental Component Summary and both ASQ subscales: Discomfort with Closeness (r=-0,23, p=0,04), and Preoccupation with relationships (r=-0,37,p=0.001).

**Conclusions**: This study supports the role of Sexual dysfunction as an independent factor than anxiety and depression in migraine related quality of life. Migraine patients with insecure attachment both with and without sexual dysfunction showed worse quality of life. Future longitudinal studies including larger populations are requested to confirm these results.

**References**

1. Eraslan D, Yalınay Dikmen P(1), Ilgaz Aydınlar E, Incesu C. The relation of sexual function to migraine-related disability, depression and anxiety in patients with migraine. J Headache Pain. 2014; 15:32.

2. Romeo A, Tesio V, Castelnuovo G and Castelli L. Attachment Style and Chronic Pain: Toward an Interpersonal Model of Pain, Front. Psychol. 2017; 8(284):1-6.

### P156 Migraine: a possible risk factor for the development of hypothyroidism in women

#### Carlo Lisotto^1^, Federico Mainardi^2^, Ferdinando Maggioni^1^, Giorgio Zanchin^1^

##### ^1^Headache Centre, Department of Neurosciences, University of Padua, Padua, Italy;^2^Headache Centre, Department of Neurology, Hospital of Venice, Venice, Italy

###### **Correspondence:**Carlo Lisotto

**Background** Migraine and hypothyroidism (HT) are frequent disorders. Migraine occurs in about 15% of the general population, while the prevalence of HT varies between 0.2 and 2.0% [1]. Both conditions are more common in women and are associated with significant comorbidities.

**Materials and methods** We retrospectively evaluated the clinical records of 4,583 patients with primary headaches referred to our Headache Centre from 2006 to 2016. We studied the prevalence of HT in the different groups of headache sufferers.

**Results** The population consisted of 2,640 patients with migraine without aura (MO), 303 with migraine with aura (MA), 235 with MO + MA, 207 with chronic migraine, 566 with tension-type headache (TTH), 388 with MO + TTH, 155 with cluster headache, and 89 with other primary headaches. Overall, 192 cases (185 females and 7 males) with HT requiring hormone therapy were observed. Of these cases, 166 (5 males) were migraineurs and 25 suffered from TTH (2 males). The prevalence of HT was 5.4% in migraine women, while it was 0.6% in men. The onset of migraine occurred prior to HT in 97.5% of women, whereas in TTH group HT preceded the headache in 78.2% of female patients. The mean age of migraine onset was 23.9 ± 8.5 in women and 25.6 ± 10.8 in men, whereas the mean age of HT onset was 36.2 ± 10.1 in women and 36.2 ± 9.7 in men.

**Conclusions** In this study HT was found in a significant proportion of migraineurs, particularly in women. Our results are consistent with past clinic-based studies demonstrating migraine and HT to be comorbid conditions [2]. In migraineurs the headache onset occurred before the appearance of HT in almost all cases (97.5%), whereas in patients with TTH an opposite trend was noted, HT occurring prior to headache in 78.2% of cases. In a recent cohort study, patients with pre-existing migraine showed a 41% increased risk of developing new onset HT, thus suggesting migraine to be a possible risk factor for this endocrinopathy [3]. A plausible proposed mechanism to explain the association between migraine and HT include alterations in the immune system, predisposing to thyroid autoimmunity. Autoimmune diseases are well known to be more common in women. The two disorders may share genetic, environmental and hormonal factors, which can explain why this association occurs almost exclusively in women. These represent interesting results that will require to be confirmed in future epidemiologic studies.

**References**

1. Chaker L, Bianco AC, Jonklaas J, Peeters RP. Hypothyroidism. Lancet. 2017; doi: 10.1016/S0140-6736(17)30703-1 [Epub ahead of print].

2. Lisotto C, Mainardi F, Maggioni F, Zanchin G. The comorbidity between migraine and hypothyroidism. J Headache Pain. 2013; 1:P138.

3. Martin AT, Pinney SM, Xie C, Herrick RL, Bai Y, Buckholz J, Martin VT. Headache disorders may be a risk factor for the development of new onset hypothyroidism. Headache. 2017; 57:21-30.

### P157 Contribution of hormonal contraceptives to the risk of ischemic stroke in women with migraine: a meta-analysis of current data

#### Simona Sacco^1^, Gabriele S Merki-Feld^2^, Karen Lehrmann Ægidius^3^, Johannes Bitzer^4^, Marianne Canonico^5^, Tobias Kurth^6^, Christian Lampl^7^, Øjvind Lidegaard^8^, E Anne MacGregor^9^, Antoinette MaassenVanDenBrink^10^, Dimos-Dimitrios Mitsikostas^11^, Rossella Elena Nappi^12^, George Ntaios^13^, Per Morten Sandset^14^, Paolo Martelletti^15,16^

##### ^1^Department of Applied Clinical Sciences and Biotechnology, University of L’Aquila, Italy; ^2^Clinic for Reproductive Endocrinology, Department of Gynecology, University Hospital, Zürich, Switzerland; ^3^Department of Neurology, Bispebjerg Hospital and University of Copenhagen, Denmark; ^4^Department of Obstetrics and Gynecology, University Hospital of Basel, Basel, Switzerland; ^5^Université Paris-Saclay, University Paris-Sud, UVSQ, CESP, Inserm UMRS1018, France; ^6^Institute of Public Health, Charité – Universitätsmedizin Berlin, Germany; ^7^Headache Medical Center Seilerstaette Linz, Austria; Department of Geriatric Medicine Ordensklinikum Linz, Austria; ^8^Department of Obstetrics & Gynaecology, Rigshospitalet, Faculty of Health Sciences, University of Copenhagen, Denmark; ^9^Centre for Neuroscience & Trauma, BICMS, Barts and the London School of Medicine and Dentistry, London, UK; Barts Sexual Health Centre, St Bartholomew's Hospital, London, UK; ^10^Erasmus Medical Center Rotterdam, Department of Internal Medicine, Division of Vascular Medicine and Pharmacology, Rotterdam, The Netherlands; ^11^Department of Neurology, Aeginition Hospital, National and Kapodistrian University of Athens, Athens, Greece; ^12^Research Centre for Reproductive Medicine, Gynecological Endocrinology and Menopause, IRCCS S. Matteo Foundation, Department of Clinical, Surgical, Diagnostic and Pediatric Sciences, University of Pavia, Italy; University Consortium for Adaptive Disorders and Head Pain (UCADH), University of Pavia, Italy; ^13^Department of Medicine, University of Thessaly, Larissa, Greece; ^14^Department of Haematology, Oslo University Hospital and University of Oslo, Norway; ^15^Department of Clinical and Molecular Medicine, Sapienza University of Rome, Italy; ^16^Regional Referral Headache Centre, Sant’Andrea Hospital, Rome, Italy

###### **Correspondence:** Simona Sacco (simona.sacco@univaq.it)

**Background**

According to current literature, both migraine and hormonal contraceptive (HC) use are risk factors for ischemic stroke (IS). HC use and migraine likely interact in increasing the risk of IS in young women; however, studies assessing that interaction have heterogeneous designs. We aimed to perform a pooled analysis of the available data regarding the association among migraine, IS, and HC use.

**Materials and methods**

A systematic literature search was conducted to identify key papers addressing the association between migraine and IS in women using HC. The reference lists and Google Scholar citations of the selected articles were also screened. Unadjusted data were pooled by means of a meta-analysis of binary outcome data with a random effects model.

**Results**

The systematic review of the literature identified 12 studies, among which 6 were excluded ( not reporting the number of subjects in the subgroups), while 6 (4 case-control and 2 cohort studies) were included in the analysis of binary outcome data [1-6]. When considering female patients with IS, migraine and HC use were not associated in cohort or in case-control studies (overall odds ratio [OR] 1.25; 95% confidence interval [CI], 0.74-2.10; P=0.40 (Fig. 1). When considering women using HC, migraine and IS were not associated in case-control studies (OR 1.65; 95% CI, 0.94-2.90; P=0.08), while when considering women with migraine, HC use and IS were associated (OR 1.82; 95% CI, 1.34-2.47; P<0.001) (Fig. 1). The combination of migraine and HC use was strongly associated with IS in case-control studies (OR 3.02; 95% CI, 2.01-4.54; P<0.001).

**Conclusions**

Our findings suggest that the combination between HC use and migraine contributes to increase the risk of IS in women. Notably, our results are from unadjusted analyses and do not take into account potential confounders, including cardiovascular risk factors. Further studies are needed; 1) to formally assess the interaction between HC use and migraine in the risk of IS in women; 2) to ascertain the role of cardiovascular risk factors in the IS risk of women with migraine using HC; 3) to clarify whether migraine with aura and migraine without aura carry the same risk of IS in women using HC; 4) to determine whether some formulation of HC are safer than others in women with migraine. More robust data will help better to improve decision making in HC use among women with migraine.

**Fig. 1 (abstract P158).**
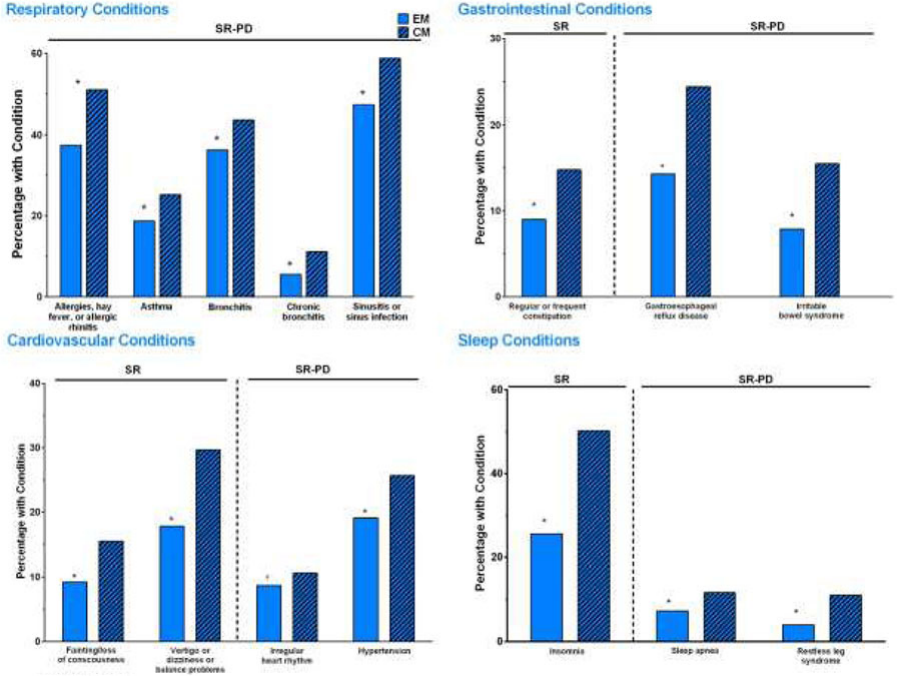
EM and CM respondents self-reporting (SR) an assessed symptom or a physician diagnosis (SR-PD) of a condition

**References**

1. Collaborative group for the study of stroke in young women. Oral contraceptives and stroke in young women. Associated risk factors. JAMA 1975; 231:718-722.

2. Schwartz SM, Petitti DB, Siscovick DS, Longstreth Jr WT, Sidney S, Raghunathan TE, Quesenberry Jr CP, Kelaghan J. Stroke and use of low-dose oral contraceptives in young women: a pooled analysis of two US studies. Stroke. 1998; 29:2277–2284.

3. Chang CL, Donaghy M, Poulter N. Migraine and stroke in young women: case–control study. The World Health Organization Collaborative Study of Cardiovascular Disease and Steroid Hormone Contraception. BMJ. 1999; 318:13–18.

4. Milhaud D, Bogousslavsky J, van Melle G, Liot P. Ischemic stroke and active migraine. Neurology. 2001; 57:1805-1811.

5. Pezzini A, Grassi M, Lodigiani C, Patella R, Gandolfo C, Casoni F, Musolino R, Calabrò RS, Bovi P, Adami A, Delodovici ML, Del Zotto E, Rota LL, Rasura M, Del Sette M, Giossi A, Volonghi I, Zini A, Cerrato P, Costa P, Magoni M, Iacoviello L, Padovani A; Italian Project on Stroke in Young Adults Investigators. Predictors of migraine subtypes in young adults with ischemic stroke: the italian project on stroke in young adults. Stroke. 2011; 42:17-21

6. Champaloux SW, Tepper NK, Monsour M, Curtis KM, Whiteman MK, Marchbanks PA, Jamieson DJ. Use of combined hormonal contraceptives among women with migraines and risk of ischemic stroke. Am J Obstet Gynecol. 2017; 216:489.e1-489.e7.

### P158 Medical (respiratory, sleep, cardiovascular and gastrointestinal) comorbidities of migraine: Results from the Chronic Migraine Epidemiology and Outcomes (CaMEO) study

#### Richard B. Lipton, MD,^1^Vincent T. Martin, MD,^2^Michael L. Reed, PhD,^3^Kristina M. Fanning, PhD,^3^Aubrey Manack Adams, PhD,^4^Dawn C. Buse, PhD^5^

##### ^1^The Saul R. Korey Department of Neurology, Albert Einstein College of Medicine, Bronx, NY, USA;^2^University of Cincinnati Headache and Facial Pain Center, University of Cincinnati College of Medicine, Cincinnati, OH, USA;^3^Vedanta Research, Chapel Hill, NC, USA;^4^Global Medical Affairs, Allergan plc, Irvine, CA, USA;^5^Montefiore Headache Center, Bronx, NY, USA

###### **Correspondence:**Richard B. Lipton (Richard.Lipton@einstein.yu.edu)

**Background**

Many comorbidities associated with migraine have a higher relative frequency in chronic migraine (CM) than episodic migraine (EM). The objective of this study was to replicate and extend work on comorbid medical conditions in a systematically recruited sample of people with migraine.

**Materials and Methods**

Data from the prospective web-based baseline survey of the Chronic Migraine Epidemiology and Outcomes (CaMEO) Study were used to identify people, recruited from an online panel using quota sampling, with EM and CM based on criteria modified from the International Classification of Headache Disorders, third edition, beta version. Participants completed a Comorbidities/Endophenotypes module that assessed 64 symptoms and conditions. Respondents were asked (1) if they ever had a specific symptom (“Self-Reported [SR]”) and, if present, (2) if the SR symptom or condition had been confirmed/diagnosed by a “doctor” (“SR-physician diagnosis [SR-PD]”). Chi-square analysis was used to compare the proportion of people with each symptom or condition among respondents with EM vs. CM. This report presents data on the Respiratory, Sleep Disorder, Cardiovascular, and Gastrointestinal comorbidity categories.

**Results**

Available CaMEO respondents with migraine (16,763) were sent the Comorbidities/Endophenotype module and 12,810 (76.4%) provided valid responses: 11,699 with EM; 1,111 with CM. Compared with the EM group, the CM group had a similar mean age (EM, 41.3 years; CM, 41.9 years), was more likely to be female (EM, 74.2%; CM, 81.5%; *P*<0.001) and white (EM, 84.0%; CM, 88.7%; *P*<0.001), and had a mean higher body mass index (EM, 27.7 kg/m^2^; CM, 28.7 kg/m^2^; *P*<0.001). The relative frequencies were significantly higher for 29 (93.5%) of the 31 SR symptoms and SR-PD conditions assessed. Conditions or groups of conditions with relative frequencies >10% higher in CM than EM included allergies/hay fever/allergic rhinitis (EM, 37.4%; CM, 51.0%), sinusitis/sinus infection (EM, 47.3%; CM, 58.8%), insomnia (EM, 35.6%; CM, 50.2%), vertigo/dizziness/balance problems (EM, 17.8%; CM, 29.7%), and gastroesophageal reflux disease (EM, 14.3%; CM, 24.4%; Fig. 1).

**Conclusions**

Overall, significantly more respondents with CM vs. EM reported medical comorbidities. Mechanisms explaining this association might include manifestations of migraine, direct causality (e.g., CM causes the comorbidity), reverse causality (e.g., the condition increases the risk of CM), and shared genetic or environmental risk factors. Confounding or detection bias (i.e., “Berkson’s Bias”) could also contribute. Future analyses will address naturally occurring subgroups (taxa) defined by migraine phenotypes and comorbidities and assess the relationships of these groups to external validators such as treatment response and clinical course.

**Fig. 1 (abstract P166).**
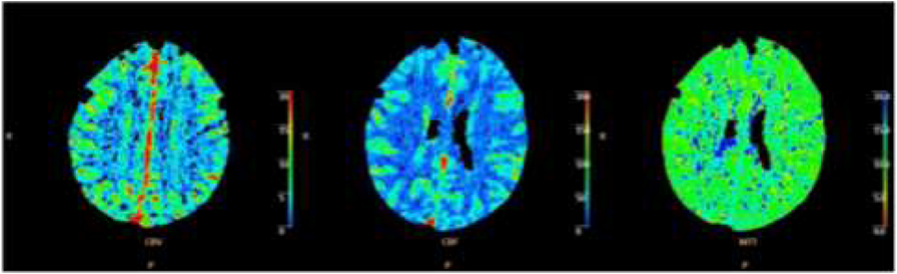
Tomography computed perfusion during migrainous aura

**Acknowledgments**

Editorial support for development of this abstract was provided by Lee B. Hohaia, PharmD, at Complete Healthcare Communications, LLC (Chadds Ford, PA), a CHC Group company, and funded by Allergan, plc (Dublin, Ireland).

### P159 The Effects of probiotic supplementation on chronic migraine(CM) headache: a randomized placebo-controlled double-blind study

#### Maryam Seyfi-shahpar^1^, Fahimeh Martami^1^, Mansoureh Togha^2^, Zeinab Ghorbani^2,3^, Soodeh Razeghi Jahromi^1,2^, Hossein Ansari^4^

##### ^1^Department of Clinical Nutrition and Dietetics, Faculty of Nutrition and Food Technology, Shahid Beheshti University of Medical Sciences, Tehran, Iran;^2^Headache Department, Iranian Center of Neurological Research, Neuroscience Institute, Tehran University of Medical Sciences, Tehran, Iran;^3^School of Nutritional Sciences and Dietetics, Tehran University of Medical Sciences, Tehran, Iran;^4^Department of Neurology, University of California San Diego (UCSD), La Jolla, CA, USA

###### **Correspondence:**Soodeh Razeghi Jahromi

**Background:** Migraine is a neurobiological disabling disorder with an estimated prevalence of 11% to 16% worldwide.

**Methods:** 50 CM patients, diagnosed according to the International Headache Society criteria (ICHD-IIIβ), were recruited. Subjects in intervention group (n=21) received 2 capsules of multispecies probiotic product Bio-kult-protexin daily, while patients in control group (n=18) took 2 placebo capsules daily for 8 weeks. Due to ethical considerations, participants were advised to continue their previous medications without changes during the study. Also they were asked not to change their dietary and exercise habitats during the intervention. A 30-day headache diary was given to patients to report the migraine characteristics including the intensity of headaches, the number of headache days, duration of each headache episodes and the number of abortive drugs that they have taken. In order to assess the disability, the Migraine Disability Assessment Scale (MIDAS) questionnaire was applied. Serum levels of CRP and TNF-alfa of patients were also measured. Data analysis performed using SPSS 19.

**Results:** After the intervention, the number of abortive drugs significantly reduced in probiotic group compared with baseline (-1.029±0.93, *P value* ≤0.000). Probiotic supplementation also significantly decreased the frequency of headache days per month (- 9.67±6.69, P value ≤0.000). The migraine severity (VAS) and attacks duration also significantly reduced with mean ±SD of changes of -2.69± 2.19 and -0.59± 1.19; for each variable respectively; P value ≤0.034). In addition, the MIDAS score showed a significant reduction in probiotic supplemented subject’ disability compared to baseline (-1±1.1; P value ≤0.000). However, no significant differences were found between the groups in CRP and TNF-alfa serum concentrations. Although there was a slight increase in serum CRP levels in probiotic group compare with baseline.

**Conclusion:** The results indicate that multispecies probiotic supplementation lead to decrease the number of abortive drugs, headache days and severity of headaches and it may also improve MIDAS score. However, more clinical trials with longer durations are needed to confirm these results and to show the effects of probiotic on inflammatory markers in patients with CM.

**Keywords**: chronic migraine headache, probiotic, MIDAS, abortive drugs, frequency of headache days.

### P160 Capsaicin versus zingember to shed some light on the therapeutic role of CGRP exhaustion versus counter-irritation - Preliminary data

#### Maria Nicolodi, Vanessa Sandoval, Silvia Mega

##### Foundation prevention and Therapy Primary Pain, Florence, Italy

###### **Correspondence:**Maria Nicolodi

**Background**

A capsaicin treatment is known to reduce by 60%-70% CGRP-LI in nerve endings included in rodents [1-2] and to block transient vanilloid receptors (TVR)-1 (capsaicin) receptor [3]. We previously evidenced a tachykinins increased release at trigeminal nerve endings during both cluster and migraine attacks [4]. Nonetheless, tachykinins’ release seems not pivotal in promoting vegetative or painful symptoms of cluster headache attack [5]. Zingember, active at vanilloid receptors [6], was matched versus capsaicin, since the zingember is suggested, but not proved to be a pain medication [7]. Comparison was aimed to debate whether capsicum-induced headache relief was completely related to capsaicin-induced tachykinins exhaustion and TVR-1 block.

**Material and methods**

Since capsaicin low concentration has been indicated as not useful when treating pain [8], we used an emulsion with a 7% capsaicin content or zingember essential oil (Ternelli Laboratory). Observation -April 2016- March 2017. Entry criteria: Diagnosis of chronic migraine (IHCD III b), exemption from other diseases. Drugs:100 μl pure zingember or 100 μl emulsion 7% capsaicin were administered into the right nostril by using 1 ml graduated pipette. Observation 1 consisted in test-retest acutely performed during headache attacks of chronic migraine sufferers (n= 48, 20 males, mean age 31.8 ± 2.9SD). Observation 2: Enrolled subjects (n=168) were randomly allocated to zingember –Group A (n= 99, 38males, mean age 31.9 ± 3.6SD) or capsaicin -Group B (n=89, 49 males, mean age 32.2 S ± 7.9SD.

**Results**

In Observation 1, capsaicin induced a pain decrease mean -72,3 ± 4.8 SD versus -28.4 ± 3.1SD zingember (p>0.005). Observation 2: A 30-days capsaicin treatment resulted in a pain relief –67.1 ± 6.3SD hours with pain. Zingember treatment did not decrease hours with pain. Capsaicin treatment adverse events: sneezing, cough and redness of treated mucosa, transient local pain. Three patients dropped out because of these side-effects. Zingember had similar adverse-effects but milder (p>0.05) than capsaicin.

**Conclusions**

A 30 days capsaicin treatment as well as a capsaicin acute treatment induced a pain relief significantly higher (p >0.0001) than zingember. Zingember also induced lower side effects regarding itching and redness- i.e. it seemingly exerts a lower (p>0.05) thermal stimulus. All together, these results seemingly indicate that, at the given doses, capsaicin, but not zingember may acutely induce counter-irritation and TVR-1 refractoriness in the time.

**References**

1. Lundberg JM, Franco-Cereceda A, Alving K, Delay-Goyet P, Luou P. Release of CGRP from sensory neurons. Ann NY Acad Sci. 1992;30: 657-178-193.

2. Franco-Cereceda A, Henke H, Lundberg JM, Peterman JB, Hokfelt, Fischer JA. Calcitonin gene-related peptide (CGRP) in capsaicine-sensitive substance P –immunoreactive sensory neurons in animal and man: distribution and released by capsaicin2] Naunyn Schmiedeberges’ Arch Phrmacol.1988; 337, 6:, 649-53.

3. Szallasi A. Vanilloid (capsaicin) receptors in health and disease. Am J Clin Pathol. 2002; 118: 110-21.

4. Nicolodi M, Del Bianco E. Sensory neuropeptides (substance P, calcitonin gene-related peptide) andvasoactive intestinal polypeptide in human saliva: their pattern in migraine and cluster headache. Cephalalgia 1990;10: 39-50.

5. Nicolodi M. Nostril capsaicin application as a model of trigeminal primary sensory neurons activation. Cephalalgia 1994; 14:134-138, 1994.

6. ConnorM, MacDonald J, Christie J, 1 Sravan Mandadi V, Basil D,. Roufogalis D. Gingerols: a novel class of vanilloid receptor (VR1) agonists 1 British Journal of Pharmacology. 2002; 137: 793 -798.

7. Terry R, Posadskzi P, Watson LK, Ernest E. The use of ginger (Zingember officinalis)f or the treatment of pain: a systematic review of clinical trials. Pain Med.2011; 12-12:1808-18.

8. Derry S, Moore AR. Topical capsaicin (low concentration) for chronic neuropathic pain in adults] Cochrane Database of systematic reviews, 2012.

### P161 Pediatric Mixed Headache - The relationship between Migraine, Tension-Type Headache and Learning disabilities - in a Clinic-based Sample

#### Jacob Genizi

##### Pediatric Neurology Unit, Bnai Zion Medical Center, Israel

**Background**: Headache is a common complaint among children. The most common primary headache syndromes in childhood are migraine and TTH. However many times they seem to overlap. The purpose of our study was to assess the relationship between pediatric migraine, tension-type headache (TTH) and learning disabilities

**Methods** *:* Children presenting with headache to three pediatric neurology clinics in the last 5 years were assessed. 262 children, 5-18 years of age, who met the criteria for migraine were included.

**Results**: Of 262 children (54% female) who had migraine, 26.2% had migraine with aura. 59 children (22.5% of the full sample) reported also having headaches that met the criteria for episodic TTH /mixed headaches. Females were more than 2.8 times more likely to experience mixed headaches than males (OR: 2.81, 95% CI: 1.43-5.54; p<.003). Multiple logistic regression analysis revealed that older age (p<0.02), family history of aura (p<.02), and (lack of) TTH (p<.003) were significant predictors of aura, whereas gender was not significant (p>0.20). Children who had migraine with aura were less likely to have mixed headaches than children who did not have aura (OR: 0.26, 95% CI: 0.11-0.63; p<.003). Children with mixed headaches were 2.7 times more likely to have a learning disability than children with migraine alone.

**Conclusions**: Episodic TTH and migraine without aura (mixed headaches) in children might be part of a continuum, which can explain the high incidence of their co-occurrence as opposed to migraine with aura. Children with mixed headaches have a higher incidence of learning disability compare to those with migraine alone.

## SISC POSTER PRESENTATIONS

### P162 Clinical features of headache attributed to Idiopathic intracranial hypertension: results from a prospective study

#### Domenico Cassano^1^, Vincenzo Pizza^2^, Vincenzo Busillo^3^

##### ^1^Headache Centre, Distretto N. 60, Via S. Giordano, 7 – 84014 Nocera Inf. (SA);^2^Headache Centre, S. Luca Hospital, Vallo della Lucania, ASL Salerno;^3^Headache Centre, Maria SS. Addolorata Hospital, Eboli, ASL Salerno

###### **Correspondence:**Domenico Cassano (081-5157425, info@domenicocassano.it)

**Introduction**

Headache is the most common presenting symptom (over 90% of cases) reported in Idiopathic intracranial hypertension (IIH), a challenging disorder characterized by increased intracranial pressure (ICP) in the absence of identifiable cause. Recent advances in neuroimaging are providing valuable insights into its etio-pathogenesis.

**Material and Methods**

Aim of this study is to describe the clinical features of headache related to IIH in a sample of 16 patients (13 women and 3 men) aged from 22 to 48 years with a mean of 31 years at diagnosis.

The management included general physical examination (with evaluation of body mass index), neurologic examination (with assessment of cognitive functions), study of the visual field, endocrinology visit and techniques of neuroimaging - CT scan of the brain, combined MR imaging with angiography (MRA) and magnetic resonance venography (MRV).

Clinical features of headache are referred to the starting period of observation (lasting 1-2 months). Data were recorded on headache diary. Follow-up was performed every month for a long-lasting period (at least 1 year).

**Results**

In more than 60% of cases, the headache is described as unilateral with focal pain, rather than generalized; 50% reported as retrobulbar and 22% referred worsening pain with extraocular movements.

In 85% the quality of pain is throbbing, exacerbated by coughing, straining and physical activity. Typical and very common is *pulsing tinnitus*, a symptom not often reported by the patients but to be specifically investigated. 78 % evaluated their headache as the most preeminent symptom. 75% reported headache as the initial symptom related to their diagnosis.

70% reported daily or nearly daily symptoms usually costant; 85% reported the pain gradually increasing in intensity.

In 80% headache lasted tipically > 1 hour. In 50% headache features are similar to migraine con nausea-vomiting, photophobia and phonophobia. In addition, patients may report associated neck stiffness and shoulder or arm pain (due to the dilatation of spinal nerve root sleeves).

**Conclusions**

This study about modalities of presentation of headache secondary to IIH, even if referred to a small sample of patients, consents to underline the lack of specific features, with the most common phenotypes represented by chronic tension type headache, chronic migraine and chronic daily headache. A phenotypic classification is useful in the management especially to comparing pre- to post-treatment headache.

**Keywords:** Idiopathic intracranial hypertension (IIH), headache related to IIH

**References**

Francis C. E., Quiros P. A., Headache Management in Idiopathic Intracranial Hypertension. Int Ophthalmol Clin. 2014;54(1):103-114. Julayanont P., Karukote A, Ruthirago D., Panikkath D., Panikkath R.

### P163 Safranal, a major constituent of*Crocus sativus*, attenuates pain-like responses and neurogenic inflammation via TRPA1 desensitization

#### Simone Li Puma, Ilaria M. Marone, Francesco De Logu, Elisabetta Coppi, Romina Nassini, Silvia Benemei, Pierangelo Geppetti, Serena Materazzi

##### Department of Health Sciences, Section of Clinical Pharmacology and Oncology, University of Florence, Florence, Italy

###### **Correspondence:**Simone Li Puma

Safranal is an aromatic compound contained in high quantities in the *Crocus sativus* (saffron). Safranal exhibits a series of biological responses including, antioxidant, antinociceptive, and antiinflammatory properties that possibly are responsible for its therapeutic effects reported by traditional medicine. Here, we investigated the hypothesis that safranal could act, at least in part, by targeting transient receptor potential channels expressed by primary sensory neurons, thus limiting pain and neurogenic inflammation. Calcium imaging, electrophysiology (both in HEK293 cells and trigeminal neurons), organ bath studies/rat urinary bladder and CGRP release experiments were performed *in vitr*o. Behavioral tests, acute nociception, and mechanical (von Frey) and cold (acetone test) allodynia were performed in mice.

Calcium imaging and electrophysiology experiments showed that, among the different TRPs expressed in peptidergic nociceptors, safranal stimulated the ankyrin 1 subtype (TRPA1), but not the vanilloid 1 and 4 (TRPV1 and TRPV4, respectively) subtypes. In the rat urinary bladder strips safranal evoked a TRPA1- and neurogenic inflammation-dependent moderate contraction that indicated partial agonism. Safranal evoked release of CGRP from rat dorsal spinal cord slices. However, after exposure to safranal the ability of TRPA1 agonists to evoke CGRP release underwent desensitization. Safranl evoked acute nociception and mechanical and cold allodynia, all effects that were attenuated in TRPA1 deleted mice or after TRPA1 antagonism. After exposure to safranal TRPA1 pain-like responses were attenuated, indicating desensitization.

Although safranal is a TRPA1 agonist, its action is weak and its behavior resembles that of a partial agonist that, depending from the specific circumstances, reduces channel activity by an antagonist or desensitizing action. Recently we have reported that two anti-migraine compounds, parthenolide [1] and isopetasin [2], are TRPA1 partial agonists and desensitizing agents. Here, we propose that the alimentary use of saffron, because of the antagonistic and desensitizing actions on TRPA1 of safranal may be of help in migraine.

**References**

1. Materazzi S, Benemei S, Fusi C, Gualdani R, De Siena G, Vastani N, Andersson DA, Trevisan G, Moncelli MR, Wei X, Dussor G, Pollastro F, Patacchini R, Appendino G, Geppetti P, Nassini R. Parthenolide inhibits nociception and neurogenic vasodilatation in the trigeminovascular system by targeting the TRPA1 channel. *Pain* 2013 Dec;154(12):2750-8.

2. Benemei S, De Logu F, Li Puma S, Marone IM, Coppi E, Ugolini F, Liedtke W, Pollastro F, Appendino G, Geppetti P, Materazzi S, Nassini R. The anti-migraine component of butterbur extracts, isopetasin, desensitizes peptidergic nociceptors by acting on TRPA1cation channel. *Br J Pharmacol* 2017 Sep;174(17):2897-2911.

### P164 The use of Symptomatic Pharmacological Treatment is Associated with the Degree of Sensitization in Patients with Tension Type Headache

#### Matteo Castaldo^1^, Lars Arendt-Nielsen^1^, Maria Palacios-Ceña^1,2^, Kelun Wang^1^, Paola Torelli^3^, Paolo Pillastrini^4^, Antonella Catena^1^, Cesar Fernández-de-las-Peñas^1,2^

##### ^1^Department of Health Science and Technology. Aalborg University, Aalborg, Denmark;^2^Departamento de Fisioterapia, Terapia Ocupacional, Rehabilitación y Medicina Física. Universidad Rey Juan Carlos, Alcorcón, Madrid, Spain;^3^Department of Medicine and Surgery, Headache Center, University of Parma, Italy;^4^Department of Biomedical and Neurological Sciences, University of Bologna, Italy

###### **Correspondence:**Matteo Castaldo (matteo.castaldo@poliambulatoriofisiocenter.com)

**Background and aims**

Our aim was to investigate the differences in clinical features and widespread pressure pain sensitivity according to the use of symptomatic medication in tension type headache (TTH).

**Methods**

Individuals with TTH diagnosed according to the International Classification of Headache Disorders (ICHD-III) criteria participated. Exclusion criteria included other primary headaches, medication overuse headache, whiplash, fibromyalgia or any neurological disorder. A 1-month headache diary was used to collect clinical data and symptomatic medication. Pressure pain thresholds (PPT) were assessed over the temporalis muscle, C5-C6 zygapophyseal joint, second metacarpal, and tibialis anterior muscle.

**Results**

One hundred and sixty eight patients (72% women, age: 45±14 years; headache frequency: 14±8 days/month; headache intensity: 5.7±1.3; headache duration: 6.1±3.2 hours) participated. One hundred and thirty-six (80%) reported use of symptomatic medication for headache (73% NSAIDs); 58 (43%) took the medication at the beginning of headache whereas 78 (57%) took the medication when the headache intensity was intense. No differences in clinical features and widespread pressure pain sensitivity was observed depending on the use of medication intake (all, P>0.157). However, patients taking the symptomatic medication when the headache was intense exhibited lower PPTs than those taking the medication at the beginning of the attack (all, P<0.05).

**Conclusions**

This study found that the use of symptomatic medication intake was not related to clinical and widespread pressure pain sensitivity in TTH, but it seems that consuming symptomatic medication at the beginning of the headache could be related to lower widespread pressure pain sensitivity.

### P165 Recurrent painful ophthalmoplegic neuropathy (RPON), can cerebrospinal fluid (CSF) analysis aid the diagnosis, where neuroimaging is still blind? A case report

#### Ciro De Luca, Filippo Baldacci, Elisa Dini, Martina Cafalli, Sara Gori

##### Neurology Unit, Department of Clinical and Experimental Medicine, University of Pisa, Pisa, Italy

**Background**

Recurrent painful ophthalmoplegic neuropathy (RPON) represents a condition, characterized by recurring headaches and palsy of the third, fourth or sixth cranial nerves [1].

The etiopathogenesis of this disease is still unrevealed and some authors propose a migraine-based mechanism while others support its neuropathic nature [2,3]. Here we report a case of RPON without migraine-like features, with negative neuroimaging and inflammatory characteristics evidenced with cerebrospinal fluid (CSF).

**Case report**

A 55-year-old man came to our department with headache and diplopia. Pain started two days earlier and was initially referred to the left fronto-orbital region, described as stabbing with severe intensity. In two days, pain became moderate, pressure-like and holocranial with diplopia in all gaze directions and ptosis of the left eye. The patient described a similar episode 4 months before on the right side with complete remission in 3 weeks, without therapy. He had no history of migraine and never suffered of headache with similar characteristics. He also had no history of diabetes. On examination we found a left third nerve palsy without pupil asymmetry, an incomplete ptosis and inability to adduct or lower his left eye. Diplopia was found in all gaze directions. The rest of the neurologic exam was normal.

Two brain magnetic resonance imaging (MRI) with contrast, few days after the episodes of ophthalmoplegia, were normal, even focusing specifically on the orbital region and studying both arterial and venous circulation. Cerebrospinal fluid (CSF) analysis performed after the second episode showed slight damage of the blood brain barrier (BBB) with elevated albumin ratio (total proteins 59 mg/dl, albumin 30.9 mg/dl). Blood routine tests and other investigations including autoimmune, sarcoidosis, tuberculosis and venereal disease screens, viral genome on CSF, antiganglioside and paraneoplastic antineuronal antibodies, HbA1c dosage and exams for tumor screening were normal.

The International Classification of Headache Disorders 3 Beta (ICHD-3Beta) criteria for the diagnosis of RPON were met [1]. He started corticosteroid therapy with progressive remission of the symptoms.

**Conclusion**

Neuro-inflammatory pathogenesis of RPON is usually supported by MRI finding of third cranial nerve post-contrast enhancement and/or nerve thickening [4,5]. In our case, two MRI were negative so we suggest CSF evidence of BBB damage as a supportive element of diagnosis that reinforces the hypothesis of RPON inflammatory nature, even when unverified with advanced neuroimaging. Moreover, the effective treatment with corticosteroids, together with the previously enlisted exams, further validates the diagnosis of RPON.

**Consent for publication**

Consent was obtained from the patient for this abstract publication.

**References**

1. (IHS), H.C.C.o.t.I.H.S. The international classification of headache disorders, 3rd edition (beta version). *Cephalalgia : an international journal of headache* 2013, *33*, 629-808.

2. Huang, C.; Amasanti, M.; Lovell, B.; Young, T. Recurrent painful ophthalmoplegic neuropathy. *Practical neurology* 2017, *17*, 318-320.

3. Margari, L.; Legrottaglie, A.R.; Craig, F.; Petruzzelli, M.G.; Procoli, U.; Dicuonzo, F. Ophthalmoplegic migraine: Migraine or oculomotor neuropathy? *Cephalalgia : an international journal of headache* 2012, *32*, 1208-1215.

4. Gelfand, A.A.; Gelfand, J.M.; Prabakhar, P.; Goadsby, P.J. Ophthalmoplegic “migraine” or recurrent ophthalmoplegic cranial neuropathy: New cases and a systematic review. *Journal of child neurology* 2012, *27*, 759-766.

5. Zhang, X.; Levy, D.; Noseda, R.; Kainz, V.; Jakubowski, M.; Burstein, R. Activation of meningeal nociceptors by cortical spreading depression: Implications to migraine with aura. *The Journal of neuroscience : the official journal of the Society for Neuroscience* 2010, *30*, 8807-8814.

### P166 Computed perfusion tomography in migrainous aura: a case series and review of literature

#### Paola Polverino^1^, Antonio Granato^1^, Mariana Ridolfi^1^, Maja Ukmar^2^, Paolo Manganotti^1^

##### ^1^Department of Medical, Technological and Translational Sciences, Headache Centre, University of Trieste, Italy;^2^Department of Radiology, Cattinara Hospital, University of Trieste, Italy

###### **Correspondence:**Paola Polverino (polverinopaola@gmail.com)

**Background**

Migrainous aura (MA) occurs with acute-onset of neurological symptoms ant it can be erroneously mistaken for an acute stroke (AS) [1]. A complete diagnostic workup in Emergency Department (ED) is important for differential diagnosis between MA and AS [2]. Perfusion computed tomography (PCT) is a readily available tool and it may assist the clinician in a prompt differential diagnosis of AS, considering its high sensitivity and specificity and the low probability of false-negatives [3,4]. Only four patients performed PCT during MA. In these cases patients underwent PTC after 70-120 minutes from symptoms onset. Two patients presented with aphasia [5, 6], one patient with aphasia and right hemiparesis [7] and the last one with left hemiparesis [7], always followed by headache. Three patients suffered from headache in the past. In all cases reduced CBF, normal CBV and increased MTT were recorded in the left hemisphere and in the right frontal lobe respectively. These findings were consistent with hypoperfusion. Focal neurological symptoms gradually resolved within 30 minutes-24 hours. No thrombolysis was given. Patient were discharged with final diagnosis of migraine with aura.

**Cases report**

We report six cases accessed to ED for acute-onset of focal neurological symptoms, two patients had mild headache (Table 1). All patients had history of migraine in the past. Unenhanced CT, Angio-CT of intra and extracranial vessels and PCT were performed 30-90 minutes after neurological symptoms onset. No acute lesions were found and PCT showed normal perfusion pattern (Fig. 1). Neurologic symptoms fully recovered within 2-10 hours. Only one patient met criteria for thrombolysis. A control brain-CT scan, brain MRI, diagnostic tests for cerebrovascular diseases were normal in all cases. Patients were discharged with diagnosis of migraine with aura, probable sporadic hemiplegic migraine and probable first attack of migraine with visual aura, according with ICHD-3 beta criteria.

**Conclusions**

Perfusion findings during MA are still controversial in literature. Four cases of PCT performed during MA have been described and all findings showed cerebral hypoperfusion. Otherwise we recorded six patients in which PCT showed normal perfusion maps during MA. These contrasting findings should be considered in the decision-making process. We suggest that clinical history of migraine, rapid improvement of symptoms and normal imaging results, in particular PCT, may indicate a MA status. Our data suggest that PCT could be useful in discriminating between MA and AS, avoiding unnecessary reperfusion therapies.

**Fig. 1 (abstract P170).**
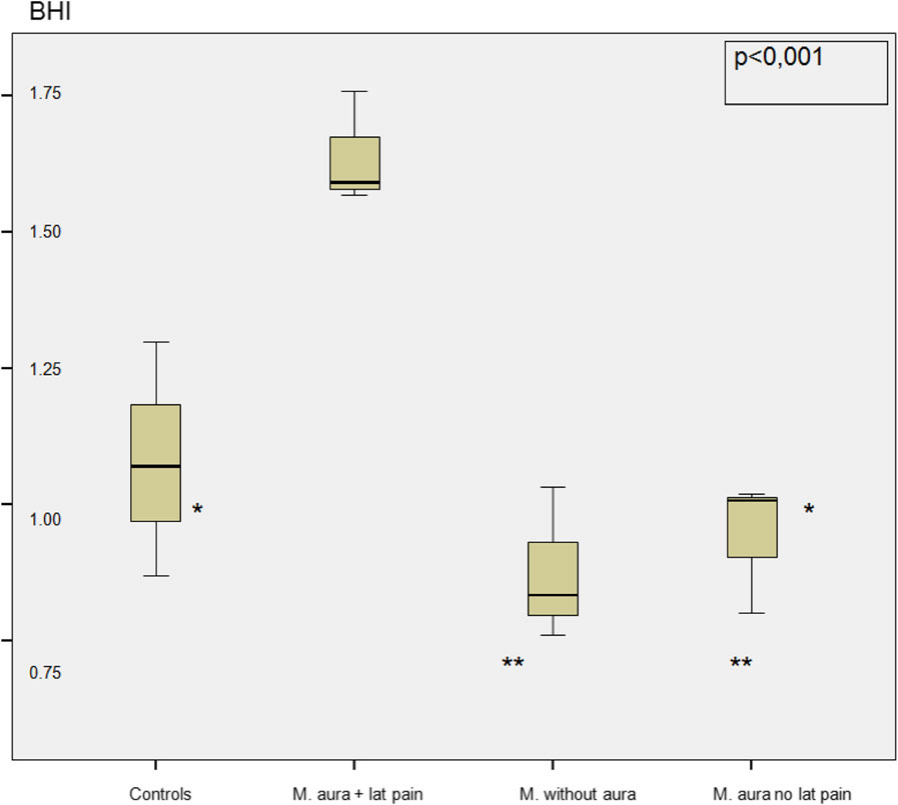
See text for description.

**Table 1 (abstract P166).** Patient Characteristics

	Age	Sex	History of Migraine	Presenting symptoms in ED	Headache at the onset of symptoms	NIHSS	Time to PCT after symptoms onset	Thrombolysis	Symptoms duration
*1*	22	M	yes (without aura)	right hemiparesis and blurred vision in the right hemifield	yes	8	60 minutes	no	2 hours
*2*	26	F	yes (without aura)	blurred vision in the right hemifield	no	2	60 minutes	no	2 hours
*3*	43	M	yes (with aura)	left emipharesis	yes	6	90 minutes	yes	10 hours
*4*	50	F	yes (without aura)	left hemiparesis, dyrarthria and left facial palsy	no	2	60 minutes	no	2 hours
*5*	81	F	yes (with aura)	aphasia	no	4	60 minutes	no	2 hours
*6*	91	F	yes (with aura)	aphasia	no	3	90 minutes	no	10 hours

**References**

1. Long B, A Koyfman A. Clinical mimics: an emergency medicine-focused review of stroke mimics. The Journal of Emergency Medicine, 2017 Feb;52(2):176-183.

2. Goyal N, Tsivgoulis G, Male S, Metter EJ, Iftikhar S, Kerro A, et al. FABS: An Intuitive Tool for Screening of Stroke Mimics in the Emergency Department. Stroke 2016; 47: 2216-20.

3. Miller C,Goldberg MF. Susceptibility-weighted imaging and computed tomography perfusion abnormalities in diagnosis of classic migraine. Emerg Radiol 2012; 19: 565-9.

4. Biesbroek JM, Niesten JM, Dankbaar JW, Biessels GJ, Velthuis BK, Reitsma JB, Van der Schaaf I C, Diagnostic Accuracy of CT Perfusion Imaging for Detecting Acute Ischemic Stroke: A Systematic Review and Meta-Analysis Departments of Radiology, and b Neurology, Rudolf Magnus Institute of Neuroscience, and c Julius Center for Health Sciences and Primary Care, University Medical Center Utrecht, Utrecht, The Netherlands 2004; 24: 533-539.

5. Nieuwkamp DJ, van der Schaaf IC, Biessels GJ. Migraine aura presenting as dysphasia with global cognitive dysfunction and abnormalities on perfusion CT. Cephalalgia 2010; 30: 1007-9

6. Miller C, Goldberg MF. Susceptibility- weighted imaging and computed tomography perfusion abnormalities in diagnosis of classic migraine. Emerg Radiol, 2012; 19:565-569.

7. Hansen JM, Schytz HW, Larsen VA, Iversen HK, Ashina M. Hemiplegic Migraine Aura Begins With Cerebral Hypoperfusion: Imaging in the acute phase. Headache, 2011; 51:1289-1296.

### P167 Prevalence and clinical characteristics of neuropsychiatric disorders in children with headache

#### Chiara Raviola, Antonia Versace, Barbara Lauria, Giulia Grasso, Ausilia Enea, Roberta Rossi, Emanuele Castagno, Antonio Francesco Urbino

##### A.O.U. Città della Salute e della Scienza di Torino, Regina Margherita Children’s Hospital, Department of Pediatric Emergency, Pediatric Headache Centre, Turin, Italy

###### **Correspondence:** Antonia Versace

**Background**

The coexistence of headache and neuropsychiatric disorders affects not only health and quality of life, but also the behaviour and the acquisition of knowledge. So far, only a few studies evaluated the association between headache and neuropsychiatric disorders, especially as regards specific learning disorders and cognitive performance. On the contrary, the association between headaches and sleep disorders, which have a high prevalence in the pediatric population, has been deeply investigated.

**Materials and methods**

We analysed retrospectively 242 children (age range: 2 – 18 years) with primary headache, seen between November 1^st^, 2015 and November 30^th^, 2016 at the Regina Margherita Children’s Hospital Headache Center of Turin, Italy. We collected personal and family history, type and characteristics of headache and neuropsychiatric disorders, the results of neuroimaging and blood tests. Statistical significance was set at p<0.05.

**Results**

On the total of 242 children (mean age: 10.6 ± 2.71 years; 116 males and 126 females), 152 (62.81%) had migraine (23 with aura), 24 (9.92%) had tension-type headache, 32 (13.22%) mixed headache and 34 (14.05%) unclassified headache. The prevalence of children with at least one neuropsychiatric disorder was 61.16%. Among 148 patients with neuropsychiatric disorders, 120 (81.08%) had at least one sleep disorder, 14 (9.46%) a neuropsychiatric disorder without any sleep disturbance and 14 (9.46%) reported both. The prevalence of specific learning disorders was 6.61%, while the prevalence of sleep disturbances was 55.37%. The percentage of patients with at least one neuropsychiatric disorder was significantly higher in the group of patients with migraine with aura: this applies to both neuropsychiatric disorders considered as a whole (p 0.026) – in particular sleepwalking (p 0.0072) – and neuropsychiatric disorders, sleep disturbances excluded (p 0.0029). In addition, a borderline association (p 0.0678) between tension-type/mixed headache and specific learning disorders was found.

**Conclusions**

In this study we demonstrated that children with migraine with aura significantly more probably have neuropsychiatric disorders in general and sleepwalking in particular, while the association between tension-type headache and specific learning disorders needs to be confirmed on a wider population. A careful assessment of neuropsychiatric disorders in children with headache is essential for optimal control of headache itself. On the other hand, identifying the association with specific neuropsychiatric disorders may be useful for their earlier diagnosis and better control, both pharmacological and non-pharmacological.

**References**

[1] Bellini et al. Headache and comorbidity in children and adolescents. The Journal of Headache and Pain. 2013.

[2] Gilman et al. Primary headache and sleep disturbances in adolescents. Headache. 2007.

[3] Genizi et al. Primary headaches, attention deficit disorder and learning disabilities in children and adolescents. The Journal of Headache and Pain. 2013.

### P168 Prophylactic treatment with TanacethumPartenium, 5-Hydrossitriptophan (5-Http) and Magnesium (AURASTOP®) in children with episodic migraine without aura: an observational study

#### Antonia Versace, Barbara Lauria, Giulia Grasso, Chiara Raviola, Roberta Rossi, Ausilia Enea, Emanuele Castagno, Antonio Francesco Urbino

##### A.O.U. Città della Salute e della Scienza di Torino, Regina Margherita Children’s Hospital, Department of Pediatric Emergency, Pediatric Headache Centre, Turin, Italy

###### **Correspondence:**Antonia Versace

**Background**

The synergistic effects of TanacetumParthenium, GriffoniaSimpliciofila and Magnesium (Aurastop ®) have been retrospectively analyzed. Until now, the nutraceutical preparation have been given to adults with migraine with aura as symptomatic treatment and as prophylactic treatment in those with episodic migraine without aura, with several benefits. In this study we prospectively evaluate the efficacy of prophilactic administration of Aurastop ® in terms of reduction of intensity, duration, frequency and analgesics use in children with episodic migraine without aura.

**Materials and methods**

In this open prospective study, we selected 21 children (age range: 6-16 years) suffering from episodic migraine without aura for at least 6 months followed at the Headache Centre of the Regina Margherita Children’s Hospital of Turin, Italy.The diagnosis of migraine without aura was done on HIS criteria (ICH-III b) and the recruitment was voluntary. Informed consent was obtained by the parents of the patients before their inclusion. The study protocol has been approved by our Ethical Committee. Clinical information about duration, intensity, frequency of the episodes and need of analgesics (number of medication) has been recorded for each patient on personal headache diary before the treatment and three months later. In 6 cases the follow-up was stopped after one month and in 3 cases after two months because of insufficient compliance.

**Results**

We report the descriptive analysis of our study. On the total of 21 patients (mean age: 12.38 ± 2.11 years; 4 males and 17 females), 7 (33.33%) showed a reduction in frequency and 4 (19.05%) in duration of migraine episodes. A decrease in intensity was detected in 16 patients (76.19%). In 13 (61.90%) cases, lower use of analgesics was reported. Only 3 patients (14.29%) didn’t obtain any benefit and in reverse 2 children (9.52%) showed improvements in all the variables; both of them had been under Aurastop® for at least three months.

**Conclusions**

Our observational study suggested that the treatment with TanacethumPartenium, 5-Hydrossitriptophan (5-Http) and Magnesium (Aurastop®) could represent an efficient prophylactic therapy also for children with episodic migraine without aura. However, a large randomised control trial should be designed to confirm the efficacy of this treatment.

**References**

1. Rajapakse, T. and Pringsheim, T. (2016) Nutraceuticals in Migraine: A Summary of Existing Guidelines for Use. Headache, 56, 808-816.

2. Tassorelli, C., et al. (2005) Parthenolide Is the Component of Tanacetum parthenium That Inhibits Nitroglycerin-Induced Fos Activation: Studies in an Animal Model of Migraine. Cephalalgia. An International Journal of Headache, 25, 612-621.

3. Zavarise P et al. (2017) A Combination of *Tanacetum parthenium*, *Griffonia simplicifolia* and Magnesium (Aurastop®) as Symptomatic Acute Treatment for Migraine Aura: A Retrospective Cohort Study. Int J Neurol Brain Disord, 4: Issue 3.

### P169 An unusual case of SUNCT responding to Carbamazepine: possible overlap among TACs and Trigeminal Neuralgia

#### E. Dini, F. Baldacci, C.De Luca, M. Cafalli, S. Gori

##### Neurology Unit, Department of Clinical and Experimental Medicine, University of Pisa, 56100, Pisa, Italy

###### **Correspondence:**E. Dini (dini.elisa87@gmail.com)

**Background:** Trigeminal autonomic Cephalalgias (TACs), including SUNCT (short –lasting neuralgiform headache), SUNA(short-lasting neuralgiform headache attacks with cranial autonomic symptoms), HC (Hemicrania Continua), PH (Paroxysmal Hemicrania) and CH (Cluster Headache); according to the International Classification of Headache Disorders (third edition, beta version, ICHD-3 beta) are considered different disorders other than trigeminal neuralgia (TN) ^1^. However clinical and terapheutic similarites let us suppose a possible physiopatology overlap among TACs and NT.

**Case Report:** A 62 years-old male, with no familial history of headache, came to our Headache Centre, describing daily episods of severe stabbing sharp pains, centred to retro-orbital and temporal right region which had begun 2 months previously. Headache attacks were accompanied by ipsilateral lacrimation, ptosis, eyelid edema and conjunctival injection, lasted between 15 seconds and one minute and occurred on average 20-30 times daily. The pain could be triggered by touching the periorbital or temple area and the others trigger points. The attacks occurred mainly during the daytime, and were sometimes followed by an achy feeling along the right side of the face. He performed a TC scan and a MRI of the brain, which resulted normal and was treated with corticosteroids without any benefit on the symptoms. His medical history was remarkable for shoulders periarthritis and renal colics. After the assessment in our Centre, a diagnosis of SUNCT was performed; however because of some atipical clinical features (for example trigger points presence) Carbamazepina 400 mg/day was prescrived, with excellent response to therapy.

**Conclusion:** Interestingly, whereas Carbamazepina is the drug of choice in TN therapy, it has been successfully used to treat this SUNCT. Open label evidence, updated now, suggests only a partial effect of Carbamazepina in patient with SUNCT, as the first therapeutic choice is Lamotrigina or intravenous lidocaine ^2,3^. The therapeutic and clinical similarities in SUNCT and NT suggest a possible similar physiopatological mechanism. Some Authors have described that lesions located in the medullary can cause simultaneous SUNCT and TN in the same patient^4,5^. The proposed physiopatological mechanism for SUNCT/SUNA is the activation of the trigemino-autonomic reflex due to activation of posterior hypothalamus which results in autonomic features, possibly produced by the connection of posterior hypothalamus to trigeminal nucleus caudalis. The exact mechanism and causes of this reflex arc are yet not understood; but the available information at present time suggests a close connection between SUNC/SUNA and TN, which could be considered different manifestations of a single pathology^6^.

**Consent to Publish.** The patient gives his consent to puplish information about itself in Journal of Headache and Pain, understand that the information will be published without his name attached, but that full anonymity cannot be guaranteed.

He understands that the text and any pictures or videos published in the article will be freely available on the internet and may be seen by the general public. The pictures, videos and text may also appear on other websites or in print, may be translated into other languages or used for commercial purposes. He has been offered the opportunity to read the manuscript. Signing the consent form does not remove patient rights to privacy.

**References**

1. Headache Classification Committee of the International Headache Society. The International Classification of Headache Disorders, 3rd edition (beta version). Cephalalgia. 2013;33:629-808.

2. Cohen AS. Short-lasting unilateral neuralgiform headache attacks with conjunctival injection and tearing.Cephalalgia 2007;27: 824-832.

3. Pomeroy JL, Nahas SJ. SUNCT/SUNA: A review. Curr pain headache Rep. 2015;19:38.

4. Lambru G and Maharu MS. SUNCT/SUNA and trigeminal neuralgia: Different disorder or variants of the same disorder? Curr opin Neurol 2014, 27: 325-331.

5. Medullary infarction causing coexistent SUNCT and trigeminal neuralgia.Cephalalgia. 2017 Apr;37(5):486-490. doi: 10.1177/0333102416652093.

6. Christian W. Tics in TACs: a step into avalanches? Systematic literature review and conclusion. Headache current 2017, 10.1111/head.13099.

### P170 Cerebral vasoreactivity in migraine patients affected by Ehlers-Danlos syndromes

#### Giada Giuliani^1^, Massimiliano Toscano^1^, Francesca Puledda^2^, Alessandro Viganò^1^, Marco Ruggiero^1^, Tommaso B Jannini^1^, Claudia Celletti^3^, Filippo Camerota^3^, Edoardo Vicenzini^1^, Vittorio Di Piero^1^

##### ^1^Department of Neurology and Psychiatry, “Sapienza” University of Rome – Italy;^2^Basic and Clinical Neuroscience, King’s College of London – UK;^3^Physical Medicine and Rehabilitation Division, Umberto I Hospital, Rome, Italy

###### **Correspondence:**Giada Giuliani (giuliani.1378642@studenti.uniroma1.it)

**Background**

Ehlers–Danlos syndromes (EDS) are a heterogeneous group of heritable connective tissue disorders mainly characterized by joint hypermobility, skin hyperextensibility and tissue fragility. We observed that EDS patients have a substantially stronger headache syndrome with respect to normal migraineurs, regarding both headache frequency and its characteristics [1].

Altered autoregulation in migraine with aura patients, possibly secondary to impaired cerebrovascular autonomic control has been demonstrated [2]. Because EDS patients may have a different vascular connective tissue elasticity, we investigate by Transcranial Doppler if EDS patients with migraine had the same vasoreactivity than controls.

**Materials and methods**

We recruited EDS patients with migraine and sex and age matched healthy controls.

Patients had to be in the interictal period and free from vasoactive drugs. Cerebral vasoreactivity was evaluated with the Breath-holding Index (BHI). Haemodynamic parameters have been recorded in basal conditions and after hypercapnic stimulus with a 2 MHz probe through acoustic temporal bones windows.

**Results**

Twenty-one patients (mean age 32±12 years, F/M:17/4) affected by EDS and migraine (9 without and 12 with aura) and twenty healthy controls (mean age 37±6 years, F/M:15/5) have been enrolled.

At rest, EDS migraine patients presented a significant higher mean flow velocities (p<0.01) and a reduced pulsatility index (p<0.01) than controls (Table 1).

After hypercapnia, vasomotor response (BHI) of EDS migraine patients differ with respect to controls according to the presence or not of aura and the presence of lateralized pain: BHI was lower in those without aura (p<0.001) whereas it was higher in EDS with migraine with aura and lateralized pain (p<0.001) (Fig. 1).

**Conclusions**

At rest, changes of TCD parameters are probably related to the different vascular connective tissue elasticity, owed to connective tissue disorder.

After hypercapnia, TCD variations are more suggestive for the migraine features. Increased BHI in patients affected by aura and pain with side predominance reflects alterations of intracranial vessel tone already observed in migraine with aura [2].

In conclusion, in EDS migraine patients TCD study showed two different responses, mainly reflecting the EDS disease at rest and the migraine component during hypercapnia.

**Fig. 1 (abstract P171).**
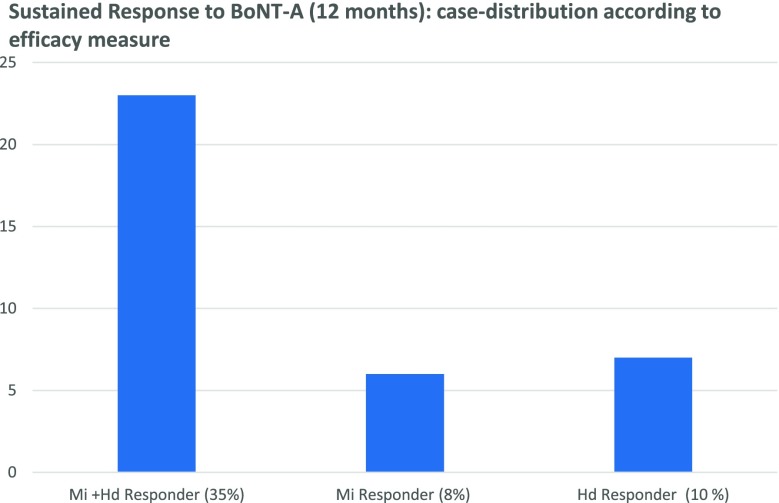
Sustained Response to BoNT-A (12 months): case-distribution according to efficacy measure

**Table 1 (abstract P170).** Haemodynamic parameters in EDS patients and controls

	EDS (mean ±SD)	Controlli (mean ±SD)	p-value
MFV at rest	73,42 ±16,21	60,54 ±11,73	< 0,01
Pi	0,76 ±0,15	0,9 ±0,15	< 0,01
Ri	0,54 ± 0,09	0,6 ±0,05	0,02
Bh MFV	94,76 ±21,1	79,36 ±13,56	< 0,01
BHI	1,22 ±0,44	1,19 ±0,17	0,707

**References**

1. Puledda F, Viganò A, Celletti C, Petolicchio B, Toscano M, Vicenzini E, et al. A study of migraine characteristics in joint hypermobility syndrome a.k.a. Ehlers–Danlos syndrome, hypermobility type. Neurol. Sci. 2015 36(8):1417-1424.

2. Vernieri F, Tibuzzi F, Pasqualetti P, Altamura C, Palazzo P, Rossini PM, Silvestrini M. Increased cerebral vasomotor reactivity in migraine with aura: an autoregulation disorder? A transcranial Doppler and near-infrared spectroscopy study. Cephalalgia. 2008 Jul;28(7):689-95.

### P171 How the therapeutic response to BoNT-A in CM-MO is increased through adjustments of treatment protocols and more accurate evaluation and management of patients

#### Valentina Rebecchi, Lucia Princiotta Cariddi, Megi Meneri, Angelo Maurizio Clerici, Marco Mauri

##### Department of Neurology and Stroke Unit – ASST-Sette Laghi - University of Insubria, Varese

**Background:** BoNT-A effectiveness in chronic migraine (CM) with or without medication overuse (MO) has been confirmed since 2010 [1]. Further observation have better defined the role of this prophylactic agent [2] as well as the optimal doses and paradigms of injection [3].

**Material and Methods:** In the present study we analyzed data from patients selected within a wider group of CM patients admitted to BoNT-A treatment at the Headache Center-Varese University Hospital–Italy, between Jan 2014 and Sept 2016. Besides diagnosis of CM-MO, admission criteria included a follow-up of at least 12 months, reliable monthly records on headache diaries, adherence to the follow-up schedule, disability and psychological assessment (MIDAS, HIT-6, SDS) [3].

N. 60 CM-MO patients (F: 51/ M:9, mean age 42.5 ± 9.9) were considered for a preliminary descriptive analysis of the parameters related to effectiveness: ≥ 30 % reduction of Headache days/Month (Hd) and/or Medication intake/Month (Mi). Our case series was then divided into two groups (G1 and G2) according to the different paradigms of treatment administered: wide range of doses and number of sites injected in the early series of patients (G1: 2014-15/N.30 cases) vs. standard dose and “follow- the- pain” paradigm of injection in the most recently treated group (G2: 2016-17/N. 30 cases).

Statistical descriptive and analytical methods were used: T- test for paired data, Chi–Square test and one-way ANOVA.

**Results**: Different percentages of response to BoNT-A treatment are shown according to the items considered as measures of efficacy: Hd/Mi /Hd +Mi (Fig. 1). The comparison between groups shows a significant decrease of Hd and Mi in favour of the most recently treated group (G2): p< 0.029 for Hd and Mi p< 0.053 for Mi (Fig. 2). In the G1 group the number of patients with lower compliance during follow-up was significantly higher than in G2 group (p< 0.001). No significant difference in term of side effects was found. Patients showing a significant decrease in Hd and Mi scored higher baseline values at HIT-6 scale ( p< 0.026); SDS score was also significantly more elevated ( p< 0.03) among cases with lower adherence to the treatment protocol. Group comparison for SDS and HIT-6 was also significant (Fig. 3)

**Conclusions**: BoNT-A for CM-MO shows sustained responses in 53 % of patients: 10 % for Hd, 8% for Mi, 35% for Mi + Hd. The percentage of responders increases trough the time with the growing accuracy of patient’s management and optimal set-up of protocols.

**Fig. 2 (abstract P171).**
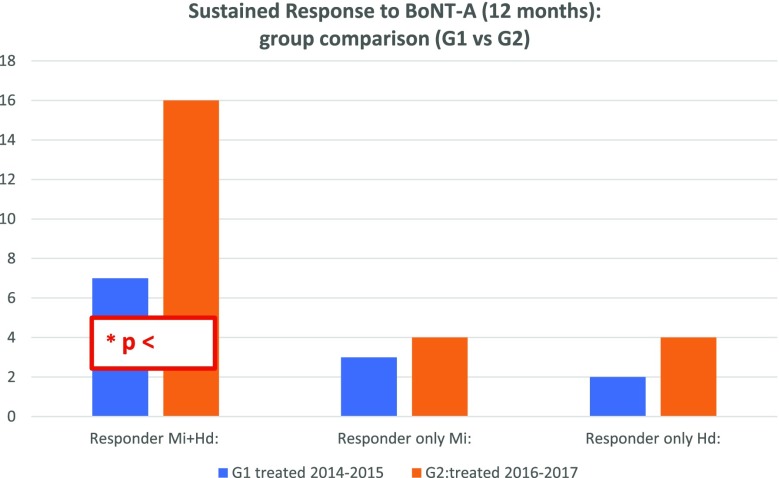
Sustained Response to BoNT-A (12 months): group comparison (G1 vs G2)

**Fig. 3 (abstract P171).**
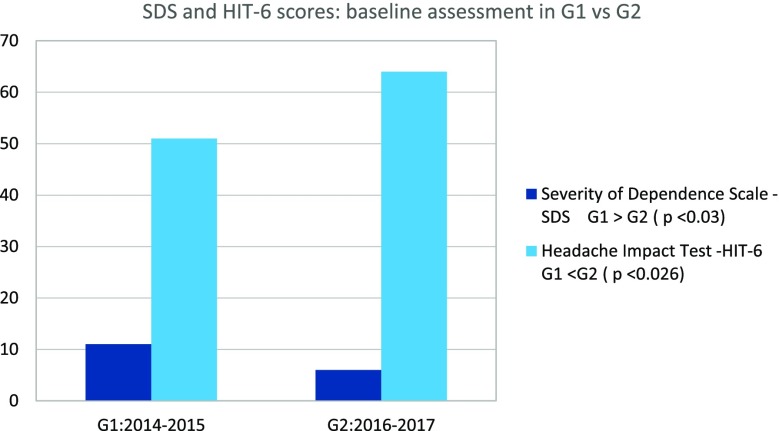
SDS and HIT-6 scores: baseline assessment in G1 vs G2

**Fig. 1 (abstract P177).**
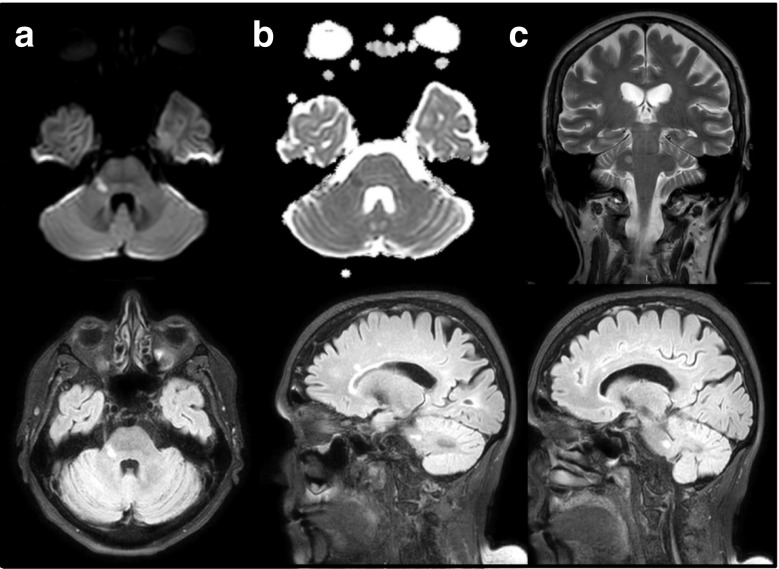
Ischemic stroke of the right brachium pontis, corresponding to the trigeminal root entry zone. **a**-**b** show high signal on DWI sequence which is confirmed by ADC maps to represent abnormal restricted diffusion. FLAIR sequence (**c** to **f**) display marked hyperintensity at the right trigeminal nucleus and sensitive root

**References**

1. Andrew M Blumenfeld, MD; Stephen D. Silberstein, et al. Insights into the Functional Anatomy behind the PREEMPT injection paradigm: guidance on achieving optimal outcomes. Headache 2017;00:00-00.

2. Becker W. J MD. The Diagnosis and Management of Chronic Migraine in primary Care. Headache 2017

3. Negro A, Curto M, Lionetto L, Martelletti P (2016) A two years open-label prospective study of Onabotulinumtoxin A 195 UI in medication overuse headache: a real world experience. J Headache Pain 17:1-9

### P172 Pharmacologically- controlled withdrawal from medication overuse in chronic migraine does not improve the therapeutic response to long-term onabotulinumtoxin type-a prophylaxis

#### Valentina Rebecchi, Lucia Princiotta Cariddi, Angelo Maurizio Clerici, Federico Carimati, Marco Gallazzi, Marco Mauri

##### Headache Center –Neurology and Stroke Unit, Osp di Circolo, Varese, Università degli Studi dell’Insubria

**Background**

Aim of the study was to assess the efficacy of Onabotulinumtoxin Type-A (BoNT-A) in the long-term treatment of Chronic Migraine (CM) with Medication Overuse (MO), and to verify if a preliminary pharmacological program for controlled withdrawal program may influence the therapeutic response to this prophylactic agent (1-2).

**Material and Methods**

We analyzed N. 49 CM-MO patients (ICHD-III beta criteria), with chronic headache and substained abuse from at least 3 yrs. Main efficacy variables included: headache days/month, medication intake/month, MIDAS, HIT-6, SDS, FSS, HADS-A/D scores. All variables were assessed at baseline and at 12 months, and analyzed by statistical descriptive and analytical methods: Wilcoxon and T- test for paired data, Chi–square test and one-way ANOVA. We divided our case series into two groups: group A - N. 29/49 pts (59%) underwent a preliminary detoxification (infusion of vitamin complex, glutatione and clordemetildiazepam: 0,25 mg for the first 3days, then gradually reduced until withdrawal in 5 days) before being treated with “follow the pain paradigma” (BoNT-A: 195 UI) every 12 weeks for up to12 months, based on PREEMPT trial experience; group B - N.20/49 pts (41%) were treated only with BoNT-A, without any preliminary pharmacological wash-out.

CM-MO treatment was considered successful when the improvement, obtained after BoNT-A - preceded or not by detoxification - resulted in more than 30% decrease from baseline in the frequency of headache days at the 12 month control.

**Results**

The two groups didn’t shown any significant difference in terms of overuse patterns, baseline psychological, disability assessment and in the frequency of side effects to injections. BoNT-A was effective in improving clinical outcome in both groups without evidence of significant differences in the clinical response between patients submitted to detoxification followed by BoNT-A treatment (group A) and subjects treated only with BoNT-A (group B).

In general, more than 55% of patients had a good clinical outcome: HIT-6 and MIDAS score showed a statistically significant improvement at 12 months, mean medications intake/month decreased significantly (mean at T0:30±23 to n 18,9±21,5 at T1; p≤0,0001) and frequency of headache days decreased from baseline (mean at T0:22,5±5,9 at T0 to 13,8±8,5 at T1; p≤ 0,0001). About 28% of pts were non responder.

The group comparison between responder and non responder to BoNT-A showed significantly elevated SDS score (p≤ 0,05), more elevated scores for anxiety (p≤0,1) and more stress factors (p≤0,01) among non-responders.

**Conclusion**

Our data confirm that CM-MO pts are responsive BoNT-A showing an early discontinuation of the overused medications without significant differences between those submitted to a preliminary pharmacological detoxification program vs those simply advised about overuse mechanisms and risks(3). These results support the role of a structured approach to CM-MO able to integrate the start of BoNT-A prophylaxis with non-pharmacological interventions aimed to counteract and prevent symptomatic drug overuse.

**References**

1) Sandrini G, Perotta A, Tassorelli C, Torelli P, Brighina F, Sances G, et al. Botulinum toxin type-A in the prophylactic treatment medication overuse headache: a multicenter, double- blind,randomized, placebo-controlled, parallel group study.J Headache Pain.2011; 12:427-33

2) Munksgaard S, Bendtsen L, Jensen R, Detoxification of medication-overuse headache by a multidisciplinary treatment program is highly effective: a comparison of two consecutive treatment methods in an open -label design. Cephalalgia.2012; 32:834-44

3) Butera C, Colombo B, Bianchi F, Cursi M, Messina R, Amadio S, Guerriero R, Como G, Del Carro U, Refractrory chronic migraine : is drug withdrawal necessary before starting a therapy with onabotulinum toxin type A? Neurol Sci (2016) 37: 1701-1706

### P173 The antimigraine butterbur ingredient, isopetasin, desensitizes peptidergic nociceptors*via*the TRPA1 channel activation*in vivo*

#### De Logu F^1^, Benemei S^1^, Li Puma S^1^, Marone IM^1^, Coppi E^1^, Ugolini F^1^, Liedtke W^2^, Pollastro F^3^, Appendino G^3^, Geppetti P^1^, Materazzi S^1^and Nassini R^1^

##### ^1^Department of Health Sciences, Section of Clinical Pharmacology and Oncology, University of Florence, Florence, Italy;^2^Departments of Neurology, Anesthesiology and Neurobiology, Clinics for Headache, Head-Pain and Trigeminal Sensory Disorders, Duke University, Durham, NC 27710 USA;^3^Department of Pharmaceutical Sciences, University of Eastern Piedmont, Novara, Italy

###### **Correspondence:**Nassini R

Clinical evidence of beneficial action of petasin/isopetasin, major constituents of butterbur [*Petasites hybridus* (L.) Gaertn.], in migraine prevention has been obtained with a preparation that contains standardised amounts (minimum 15%, corresponding to 7.5 mg) of the compounds. Several hypotheses have been advanced to explain the antimigraine action of petasin/isopetasin, However, none of these actions seems to be relevant for migraine pathophysiology. Petasin and its cross-conjugated isomer, isopetasin, contain electrophilic double bonds and can potentially interact with bionucleophiles. TRPA1 belongs to the larger family of the TRP channels and, together with the TRP vanilloid 1 (TRPV1) and vanilloid 4 (TRPV4), is expressed by a subpopulation of primary sensory neurons also characterised by their dual function of signaling pain and releasing the neuropeptides substance P (SP) and calcitonin gene-related peptide (CGRP), which mediate neurogenic inflammation.

The possible role of TRPA1 in migraine is supported by its co-expression in the same nociceptor subpopulation with CGRP, the neuropeptide that markedly contributes to migraine pain. Given the role of electrophilic compounds for the paradoxical induction/prevention of headache *via* modulation of the activity of the TRPA1 channel, we wondered if the butterbur sesquiterpenoids could target the channel.

Isopetasin produced facial rubbing that recapitulated the features of the TRPA1 selective agonist, AITC, being abrogated by HC-030031, and unaffected by TRPV1 and V4 selective antagonists. In addition, systemic isopetasin administrations for 3 consecutive days reduced rubbing responses to AITC, demonstrating desensitization of peptidergic TRPA1 expressing neurons.. With more direct relevance for headaches and migraine, we tested whether this would also affect CGRP-mediated dilation of meningeal arteries, which we subsequently assessed in rats. After isopetasin administrations for 5 consecutive days, increases in blood flow produced by the TRPA1 agonist, acrolein, and the TRPV1 agonist, ethanol were reduced.

Present findings add the butterbur constituent, isopetasin, to the list of TRPA1-tropic agents, which, like parthenolide, stimulate the channel, thereby causing defunctionalisation of peptidergic trigeminal nerve terminals, and attenuating their ability to release CGRP and signal pain. Successful treatment and prevention of migraine by targeting of TRPA1 ion channels and the ensuing peptidergic sensory neuron defunctionalisation by parthenolide and isopetasin, provides a solid basis for future basic and translational-medical investigations of TRPA1-tropic approaches for migraine.

### P174 Nervus intermedius neuralgia: a case report with unusual MRI findings

#### Flora Govone, Annalisa Gai, Alessandro Vacca, Milena Zucca, Paola De Martino, Salvatore Gentile, Maria Teresa Giordana, Innocenzo Rainero, Elisa Rubino

##### Neurology I - Headache Center, Department of Neuroscience “Rita Levi Montalcini”, University of Torino, Italy

###### **Correspondence:**Flora Govone (floragovone@gmail.com)

**Backgroud**

Nervus intermedius neuralgia (NIN), also called geniculate neuralgia, is a rare cause of otalgia, more common in people over 65, characterized by paroxysmal pain, lasting from seconds to minutes, localized to the auditory canal and retro auricular area, that may radiate to temporal or parieto-occipital regions during severe episodes [1].

Despite its unclear aetiology, a neurovascular compression (NVC) is one of the most advocated causes of secondary NIN, leading to a hypothetical demyelination at the root entry zone. There are anecdotal reports of herpes zoster, schwannoma or Lyme disease involving nervus intermedius [2].

Nervus intermedius is usually undetectable on MRI because of its small diameter and course along with the vestibular and facial nerves, but a NVC can be revealed as a vascular structure in contact with brainstem and the aforementioned cranial nerves.

**Case Report**

A 77-year-old caucasian male was referred to our Neurology Clinic because of episodes of deep left-ear pain, lasting from 4 months. This paroxysm occurred several times a day, especially by night, frequently triggered by cold air and chewing. The patient suffered also from episodic migraine without aura, gastritis and he recently underwent dental implantation. Different clinical and radiological evaluations were performed, including an otolaryngologist and a maxillofacial surgeon visit and an orthopantomography, all negative. Our neurologic examination, performed in an asymptomatic period, was normal, except for left-sided mild hearing loss. A 1.5 T MRI with gadolinium, focused on posterior fossa and temporal bone, was normal, except for a nodular area of contrast enhancement at left geniculate ganglion level, possible expression of blood-brain barrier damage or demyelination.

The patient started gabapentin until a dose of 300 mg/bid, with a complete resolution of symptoms in 1 month. A further MRI is planned at 6 months in order to assess the neuroradiological evolution.

**Conclusions**

This case report suggests that NIN, although rare, should be considered in the differential diagnosis of atypical facial pain disorders. To the best of our knowledge this is the first case report that postulate a possible demyelinating lesion of geniculate ganglion as causative of NIN.

The first-line therapy of NIN is pharmacological, as in more common craniofacial neuralgias, including anticonvulsivants, such as carbamazepine, lamotrigine or gabapentin. A surgical approach is reserved for refractory cases and consists in microvascular decompression or neural and geniculate ganglion transaction (3).

**Consent for publication:** The authors declare that written informed consent was obtained for publication.

**References**

1. Headache Classification Committee of the International Headache Society (IHS). The International Classification of Headache Disorders, 3rd edition (beta version). Cephalalgia. 2013 Jul;33(9):629-808.

2. Tubbs RS, Steck DT, Mortazavi MM, Cohen-Gadol AA. The nervus intermedius: a review of itsanatomy, function, pathology, and role in neurosurgery. World Neurosurg. 2013; 79(5-6):763-7.

3. Inoue T, Shima A, Hirai H, Suzuki F, Matsuda M. Nervus intermedius neuralgia treated with microvascular decompression: a case report and review of the literature. NMC Case Rep J. 2017 Jun 8;4(3):75-78.

### P175 Impact of visceral pain on migraine symptoms in comorbid patients

#### Giannapia Affaitati^1^, Raffaele Costantini^2^, Domenico Lapenna^3^, Francesco Cipollone^4^, Maria Adele Giamberardino^1^

##### ^1^Headache Center, Geriatrics Clinic, Department of Medicine and Science of Aging and Ce.S.I.-Met, Chieti University, Chieti, Italy;^2^Institute of Surgical Pathology, Chieti University, Chieti, Italy;^3^Department of Medicine and Science of Aging, Chieti University, Chieti, Italy;^4^Medical Clinic, Department of Medicine and Science of Aging, Chieti University, Chieti, Italy

###### **Correspondence:**Giannapia Affaitati (gp@unich.it)

**Background**

Migraine (M), particularly with a high frequency of attacks or chronic, very often coexists with visceral pain of various origin, e.g., irritable bowel syndrome (IBS), primary dysmenorrhea (Dys) or pelvic pain from endometriosis (Endo) [1,2]. The clinical observation suggests that visceral pain episodes in comorbid patients trigger/exacerbate migraine symptoms but systematic studies are still lacking with this respect. This study assessed the impact of visceral pain comorbidities and their treatment on migraine symptoms and pain sensitivity in somatic tissues of extracranial sites in patients with M+IBS, M+Dys and M+Endo as compared to M-only patients, before and after specific therapy of the visceral comorbidity.

**Materials and methods**

Female patients [18-43 years] with high frequency episodic migraine were examined, who presented concurrent: IBS [n.37], Dys [n.35] or Endo [n.26] in comparison with Migraine-only patients [n.30]. They underwent pain threshold measurement to pressure and electrical stimulation of skin, subcutis and muscle in nonpainful extracranial areas (trapezius, deltoid, quadriceps) in basal conditions. In comorbid patients the sensory evaluation was repeated after treatment [6-month diet for IBS (n.22), 6-month hormonal therapy for Dys (n.23), laser for Endo (n. 12)] or no treatment (n.15,12,14)] of the visceral pain syndromes; values before and after treatment or no treatment were compared [3]. Migraine symptoms [n. of monthly attacks, symptomatic drug consumption] after visceral pain treatment, or no treatment, were compared with those preceding treatment.

**Results**

In basal conditions all comorbid patients vs M-only patients presented significantly higher migraine pain and somatic tissue hypersensitivity (0.05<P<0.01). After visceral therapy there was a significant reduction of number of monthly migraine attacks and relative symptomatic drug consumption [0.05<P<0.01] and pain hypersensitivity [increased pain thresholds in all tissues and body sites (P<0.03)]. No improvement occurred in comorbid patients not undergoing visceral treatment.

**Conclusions**

Visceral pain significantly contributes to migraine pain, probably through enhancement of the level of central hyperexcitability by the visceral nociceptive input. Its specific and systematic treatment is thus recommended in comorbid patients as a complementary step towards optimal management of migraine.

**References**

1. Cámara-Lemarroy CR, Rodriguez-Gutierrez R, Monreal-Robles R, Marfil-Rivera A. Gastrointestinal disorders associated with migraine: A comprehensive review. World J Gastroenterol. 2016;22(36):8149-8160. doi: 10.3748/wjg.v22.i36.8149.

2. Spierings EL, Padamsee A.Menstrual-Cycle and Menstruation Disorders in Episodic vs Chronic Migraine: An Exploratory Study.Pain Med. 2015;16(7):1426-1432.

3. Giamberardino MA, Costantini R, Affaitati G, Fabrizio A, Lapenna D, Tafuri E, Mezzetti A.Viscero-visceral hyperalgesia: characterization in different clinical models. Pain. 2010;151(2):307-322.

### P176 Disappearance in the spa area in cephalalgic patients in chronic polytherapy. “tabula rasa” project

#### E. Pucci^1^, S. Cristina^2^, N. Ghiotto^2^, F. Antonaci^1^, A. Costa^1^, Massimo Radaelli^3^

##### ^1^Headache Science Center - University Consortium for the Study of Adaptive Disorders and Headache (UCADH), Department of Brain and Behavioral Sciences, University of Pavia, IRCCS “C.Mondino” Pavia;^2^Department of Brain and Behavioral Sciences, University of Pavia, IRCCS “C.Mondino” Pavia. Italy;^3^Saint George School. Boario Terme/Milano

Four our Country Thermalism is an essential resource, available to National Health System. The idea to use thermal facilities indetoxification of chronic headache patients, who need to clean out complex pharmacological interaction, by combined treatments leads to the Projet “Tabula Rasa” launch. These patients need complex therapeutic strategies directed to enzymatic normalization. The Project consider an enzymatic nutritional supplements and drugs withdrawal, under direct medical monitoring, during a period of two weeks in thermal setting. The patients will continue at home the treatment with biodynamic agents and will be addressed to follow-up. The daily monitoring by Headache Specialist during the stay in thermal setting will allow to establish tailored therapeutic stretegies.

The “Tabula Rasa” Project

After the initial visit to the patient, a 3-day period of 10 to 10 ml of Citozym will be recommended for a total of 20 ml daily, which will be taken from the fourth to the fourteenth day at a total dosage of 0.5 ml / kg of body weight to be taken in small sips of 500 ml of water over the course of the day. On the basis of our pilot experiences already at this stage, the gradual reduction of previously taken medications will be possible, based on an evaluation (by Ad-hoc Diary) of the Psychological-Behavioral Profile, with a general evaluation by the Specialist and, necessary, with massotherapy sessions.

At the end of the “thermal” stay, patients will be asked to continue for two months a protocol based on the use of 0.5 ml / kg / day of Citozym, to which another biodynamic agent (Propulzym) will be added to the dosage of 10 ml / day, mixed with Citozym aqueous solution, and 10 ml in the morning of a third biodynamic agent (Ergozym Plus) aimed at energy balance and necessary vitamin supplementation. According to the basic design of CARE I protocol. After the first months of home treatment, scheduled periodic checks are planned, monitoring by the clinician / ambulatory / Reference Center; oriented towards the progressive elimination of previous drugs and the possible introduction of new ones.

The problem of the wash out of previous therapies represents a potential obstacle to the establishment of rational therapeutic strategies in chronic headache patients: with the help of new biodynamic preparations, which can favorably alter the “enzyme soil”, it is considered possible to exit the empirical operation and set up rational therapeutic strategies.

### P177 A migraine attack as onset of trigeminal root entry zone ischemic stroke: a case report

#### VirginiaBozzoni, Elena Pegoraro, Ferdinando Maggioni

##### Department of Neurosciences, University of Padua, Padua, Italy

###### **Correspondence:**VirginiaBozzoni (virginia.bozzoni@gmail.com)

**Background.** Painful trigeminal neuropathy and “orofacial pain” due to brain stem stroke have already been described^[1,2,3]^. We report a case of pontine stroke presented as a migrainous attack with subsequent onset of trigeminal sensory deficit.

**Case report.** A 74-year-old, migrainous woman (ICHD-III) [4] presented with sudden onset of a severe right migraine attack with nausea, phono and photophobia stopped after NSAIDs treatment. The attack was followed by persistent hypoesthesia in the distribution of mascellar and mandibular branch of ipsilateral trigeminal nerve. No cardiovascular risk factors were known in her medical history. Brain CT was normal. Neurological examination showed right facial hypoesthesia, reduced right blink reflex and mild right hypoacusia. Brain MRI highlighted a recent ischemic lesion in right pons, corresponding to the trigeminal entry root zone. We therefore started antiplatelet and statin treatment. Routine blood tests, echocardiogram, Holter ECG monitoring were normal. Neck and transcranial doppler ultrasound detected mild bilateral carotid stenosis and intracranial Angio-CT was normal. During the recovery the patient reported another right migrainous episode, resolved with acetaminophen treatment, while right face hypoestesia showed some initial improvement in following weeks.

**Conclusions.** Pons infarction is known to cause pseudo-peripheral trigeminal symptoms and to determine a post-stroke painful trigeminal neuropathy^[3]^. Here we described a trigeminal sensory deficit due to selective trigeminal root entry zone ischemic lesion onset after a migraine attack.

**Consent for publication:** The authors declare that written informed consent was obtained for publication.

**Fig. 1 (abstract P192).**
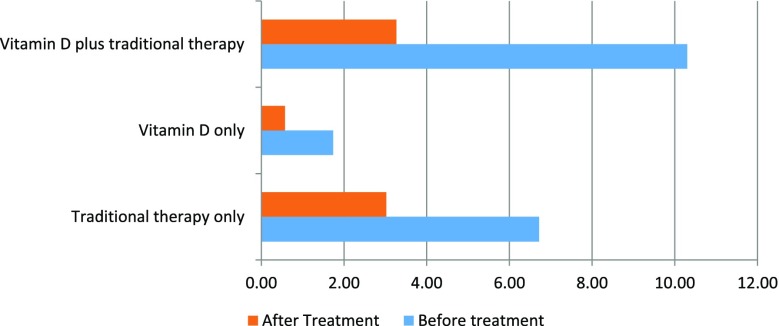
See text for description

References

1. Ching-Tang H., Chung-Ping L., Ying-Chu C., Min-Chien T. A Rare Case of Painful Trigeminal Neuropathy Secondary to Lateral Medullary Infarct: Neuroimaging and Electrophysiological Studies. Acta Neurol Taiwan. 2015; 24(2):63-8.

2. Friedman D. Unilateral headache associated with a pontine infarction. Cephalalgia. 2010;30(12):1524-6.

3. Iizuka O., Hosokai Y., Mori E. Trigeminal neuralgia due to pontine infarction. Neurology. 2006;66(1):48.

4. The International Classification of Headache Disorders, 3rd edition (beta version). Cephalalgia 2013; 9: 627–808.

### P178 The importance of a multidisciplinary approach in the management of pediatric migraine

#### Marcuzzi E^1^, Saretta F^1^, Savi L^2^

##### ^1^Pediatric division, Hospital of Palmanova, AAS2 Bassa Friulana-Isontina;^2^Headache Center, Città della salute, Torino

Migraine is very common in childhood, and it has been reported in about 10% of school-age children. Unfortunately, there aren't international guidelines yet and pharmacological therapies differ in Europe and USA. Therefore, a proper management relies on a multidisciplinary approach, which could improve outcomes and patients quality of life, especially in pediatric age. [1] We strongly believe that in every visit all aspects of management should be addressed and reviewed.

First of all, physicians must educate the patient and the family regarding the physiology of headache and migraine, how to fulfill the migraine's diary and what are the goals of chosen therapies. The “red flags” for migraine must be particularly highlighted and indications, on when and how a clinical evaluation is needed, must be given.

Is very important to underline some aspects of lifestyle, such as the importance of a balanced diet, a good sleep hygiene, the maintenance of an ideal body weight, regular sports and no smoking and no drinking alcohol (especially in adolescence).

Then physician must focus on the symptomatic therapies and the correct use of analgesics, in particular the timely administration of age and weight appropriate dose. Overuse and abuse of analgesics should be avoided to prevent chronic headache. In childhood, paracetamol and ibuprofen are drugs of choice. Triptans are approved over twelve years of age and different triptans are available in different countries: e.g. sumatriptan and zolmutriptan in Italy and almotriptan and rizatriptan in USA.

Finally, preventive therapy should be discussed with all patients, even though only patient with chronic headache could benefit from it. A real challenge in pediatric age is when and how to start a preventive therapy in children with frequent and debilitating episodes, since there isn't a international consensus on the drug of choice (even on the superiority of drug compared to placebo). Non pharmacological therapy, such as integrators and vitamins (magnesium and tryptophan), is usually recommended as first choise. Behavior therapy and biofeedback could also be suggested, although there aren't complete data to support their use, yet.

In our opinion the multidisciplinary approach plays an irreplaceable role in the management of headache and migraine in childhood on which physician should be properly educated.

**References**

[1] Kabouche MA, Powers SW, Vockell AL, et al. Outcome of a multidisciplinary approach ti pediatric migraine at 1,2 and 5 years. Headache 2005; 45:1298-303.

### P179 Investigating metacognitive executive dysfunctions in patients with medication-overuse headache

#### Milena Zucca, Elisa Rubino, Alessandro Vacca, Flora Govone, Annalisa Gai, Paola De Martino, Silvia Boschi, Maria T. Giordana, Innocenzo Rainero

##### Neurology I, Department of Neuroscience “Rita Levi Montalcini”, University of Turin

**Background:**

Medication-overuse headache (MOH) is one of the most common chronic disorders characterized by headache occurring more than 15 days per month and by regular overuse of symptomatic medications. Several studies suggested a causative role of the mesocorticolimbic dopaminergic system in the development of MOH [1]. Several areas of this network, as the ventral striatal medial prefrontal circuit, are primarily associated with metacognitive executive functions described as both the self-evaluation of cognitive abilities and the subsequent achivement of self-directed behaviors. The aim of our study was to analyze the role of metacognitive executive dysfunctions in the pathogenic mechanism that underlies substance abuse in MOH.

**Material & Methods:**

Fifty-two patients were selected for the study: 30 patients [M/F=5/25; age (mean±SD)=46.6±10.9 yrs] fulfilled the criteria for MOH and 22 [M/F=7/15; age=42.6±12.2 yrs] for episodic migraineurs (EM) [2]. Twenty-five healthy control (HC) were also included in the study. All the subjects were recruited at the Headache Center of the Department of Neuroscience in Turin (Italy). Metacognitive executive functions were assessed using the metacognitive version of the Wisconsin Card Sorting Test (M-WCST) [3] which differs from the standard version for the presence of two requests: (I) the level of confidence expected from the response; (II) the decision of subjects to gamble. Different metacognitive indexes were achieved: accuracy score (AS); free-choice improvement (FCI); global monitoring (GM); monitoring resolution (MR); control sensitivity (CS) and monetary gains (MG). Metacognitive aspects were compared between groups using ANOVA test. All statistical analyses were performed with SPSS (version 21.0).

**Results:**

Metacognitive examination of M-WCST showed that patients with MOH present worse performances than HC in FCI (p=.033), GM (p=.006), MG (p=.039) and CS (p<.001). Intriguingly we also found statistically significant differences between MOH and EM in the same metacognitive indices, as FCI (p=.024), GM (p=.003), MG (p=.025), and CS (p<.001). No differences were found among groups either in the AS or in the MR.

**Conclusions:**

Our data show that patients with MOH present the same self-monitoring ability as HC and EM but worse performances in indices that assess the patients’ ability to use a proper monitoring process to guide an adequate behavior. In particular, our MOH patients show low scores in CS, given by the correlation calculated across all sorts between the level of confidence and the decision to bet. In conclusion we speculate that a disadaptive coping behavior predisposes MOH patients to a relapse of chronic substance abuse. Additional studies are needed to confirm our data.

**References**

1. Ferraro S, Grazzi L, Muffatti R, et al. In medication-overuse headache, fMRI shows long-lasting dysfunction in midbrain areas. Headache. 2012;52:1520–1534.

2. Headache Classification Committee of the International Headache Socie ty. The International Classification of Headache Disorders, 3rd edition (betaversion). Cephalalgia. 2013;33:629-808.

3. Koren D, Seidman LJ, Goldsmith M, Harvey PD. Real-world cognitive– and metacognitive– dysfunction in schizophrenia: A new approach for measuring (and remediating) more “right stuff”. Schizophrenia Bulletin. 2006; 32(2), 310–326.

### P180 Headache and the female gender

#### Anna Ambrosini

##### Headache Clinic - IRCCS Neuromed, Pozzilli (Isernia), Italy

Prevalence of migraine in women is almost three times greater than in men. This is true particularly in the childbearing age, whereas the migraine prevalence tends to be similar in both genders during the childhood and in the elderly patients. Although the migraine disease is known to run in families, and it is thus suggested to have a strong genetic predisposition, migraine attacks are usually started by external/internal triggers, among which the hormonal cycle plays a major role. Menstrual-related migraine attacks are known to be longer and more severe than the ones occurring during other phases of the hormonal cycle, and most women report a worsening of their disease in the premenopausal phase, when hormonal levels tend to be less stable, or during oral contraceptive treatments, and a significant amelioration of their migraine frequency and severity during pregnancy and in menopause. This strict correlation between migraine and the hormonal cycle represents usually a therapeutic challenge to the headache specialists, as most of the common pharmacological preventative and symptomatic strategies are unsuccessful when applied to control hormonal-related migraine attacks.

### P181 Role of the transient receptor potential ankyrin type-1 (TRPA1) channel in migraine pain: evaluation in an animal model

#### Chiara Demartini^1,2^, Rosaria Greco^1^, Anna Maria Zanaboni^1,2^, Germana Tonsi^1,2^, Barbara Richichi^3^, Oscar Francesconi^3^, Cristina Nativi^3,4^, Cristina Tassorelli^1,2^

##### ^1^Laboratory of Neurophysiology of Integrative Autonomic Systems, Headache Science Center, “C. Mondino” National Neurological Institute, 27100, Pavia, Italy;^2^Department of Brain and Behavioral Sciences University of Pavia, Pavia, Italy;^3^Department of Chemistry ‘Ugo Schiff’, University of Florence, 50019, Sesto Fiorentino, Florence, Italy;^4^FiorGen, University of Florence, 50019 Sesto Fiorentino, Florence, Italy

**Introduction -** To date, the pharmacological treatment of migraine remains somewhat unsatisfactory, partly because the pathophysiology of this disabling disease is still poorly understood. Clinical and experimental studies have pointed to the possible involvement of the transient receptor potential ankyrin type-1 (TRPA1) channels in migraine pain. To further investigate the role of TRPA1 in the mechanisms of migraine pain in an animal model of migraine using a novel TRPA1 antagonist (ADM_12) as a probe.

**Methods -** The effects of ADM_12 on nitroglycerin-induced hyperalgesia at the trigeminal level were investigated in male rats using the quantification of nocifensive behavior in the orofacial formalin test. The expression levels of the genes coding for c-Fos, TRPA1, calcitonin gene-related peptide (CGRP) and substance P (SP) in peripheral and central areas relevant for migraine pain were analyzed. CGRP and SP protein immunoreactivity was also evaluated in nucleus trigeminalis caudalis (NTC).

**Results -** The findings show that in the nitroglycerin-induced hyperalgesic rats, ADM_12 has an anti-hyperalgesic effect in the second phase of orofacial formalin test. This effect is associated to a significant inhibition of nitroglycerin-induced increase in c-Fos, TRPA1 and neuropetides mRNA levels in medulla-pons, cervical spinal cord and in trigeminal ganglion. Whereas, no differences between groups were seen as regards CGRP and SP protein expression at NTC level.

**Conclusion –** The present findings support a critical involvement of TRPA1 channels in the pathophysiology of migraine, and show their active role in counteracting hyperalgesia at the trigeminal level.

### P182 Chronic and Intermittent administration of systemic nitroglycerin in the rat induces an increase in c-Fos and CGRP gene expression in areas involved in migraine pain

#### Rosaria Greco^1^, Chiara Demartini^1,2^, Anna Maria Zanaboni^1,2^, Germana Tonsi^1,2^, Attilio Iemolo^3^, Giuseppe Nappi^1^, Giorgio Sandrini^1,2^, Cristina Tassorelli^1,2^

##### ^1^Laboratory of Neurophysiology of Integrative Autonomic Systems, Headache Science Center, C. Mondino National Neurological Institute, 27100 Pavia, Italy;^2^Department of Brain and Behavioral Sciences, University of Pavia, 27100, Pavia, Italy;^3^Laboratory of Functional Neurochemistry, Center for Research in Neurodegenerative Diseases, Center for Research in Neurodegenerative Diseases, “C. Mondino” National Neurological Institute, 27100, Pavia, Italy

**Background:** Calcitonin gene related peptide (CGRP) is a key neuropeptide involved in the activation of the trigeminovascular nociceptive system and it is likely implicated in migraine chronicization. In the present study we investigated the role of CGRP in migraine chronicization in an animal model that mimics the condition of chronic migraine.

**Methods:** The animal model was based on chronic and intermittent administration of nitroglycerin (NTG). Male Sprague-Dawley rats were injected with NTG (5mg/kg, i.p.) or vehicle, every two days over a 10-day period (5 injections total). A group of animals was injected with Topiramate (30mg/kg, i.p.) or vehicle every day for 9 days. Twenty-four hours after the last administration of NTG or vehicle, animals underwent a tail flick test for the evaluation of nociceptive threshold and a Von Frey test, for the evaluation of orofacial mechanical allodynia. Rats were subsequently sacrificed and their medulla-pons region, cervical spinal cord (C1-C2) and trigeminal ganglia were immediately chopped into parts for the evaluation of c-Fos and CGRP gene expression by real-time polymerase chain reaction (RT-PCR).

**Results:** Chronic and intermittent NTG administration caused a condition of hyperalgesia and orofacial allodynia, detected respectively as a reduction in the latency of the tail flick test and as a reduction in the threshold of mechanical sensitivity when compared either to baseline values. NTG administration also induced a significant increase in c-Fos and CGRP gene expression in the medulla-pons region, cervical spinal cord and trigeminal ganglia. Topiramate treatment prevented the development of NTG-induced pain hypersensitivity, allodynia and alteration of gene expression in the above areas.

**Conclusion:** These findings describe the occurrence of neuronal activation and CGRP synthesis in the trigeminal ganglia and in specific areas of the CNS that are relevant for trigeminal pain processing in an animal model of chronic migraine. The inhibitory effect of topiramate, whose mechanisms of action involve blockade of voltage-dependent sodium channels, potentiation of GABAergic transmission and inhibition of glutamatergic transmission, points to an important role for these mechanisms in CGRP mediated processes underlying pain chronification. From a translational clinic of view, these findings underline the possibility and opportunity to pharmacologically intercept and prevent migraine chronification.

### P183 Psychopathological profile of Medication Overuse Headache patients, drug assumption and degree of disability

#### Simone Migliore, Matteo Paolucci, Livia Quintiliani, Claudia Altamura, Fabrizio Vernieri

##### Policlinico Universitario Campus Bio-Medico, Roma, Italia

Patients with Medication Overuse Headache (MOH) show a complex psychopathological profile characterized by high rate of mood and anxiety disorders, obsessive–compulsive disorder and dependence [1, 2]; this profile assessment should be included in patients’ evaluation [3].

We arranged a set of questionnaires to assess psychological profile of MOH patients followed by our Headache Centre. This set is composed by Beck Depression Inventory II Edition (BDI-2), trait subtest of State-Trait Anxiety Inventory (STAI-Y), Difficulties in Emotion Regulation Scale (DERS), Barratt Impulsiveness Scale (BIS-11), Toronto Alexithymia Scale (TAS-20).

Our aim was to correlate patients’ psychological profile both with number of medications taken per month and degree of disability measured by Headache Impact Test (HIT-6).

From November 2015 to July 2016 we recruited 22 patients (F=73%; M=27%), mean age 46,5 +/- 12,5.

Patients showed a median of 24,5 days of headache per month (IQR 10) and a median of 30 medications taken per month (IQR 28,5), without significant difference between men and women. Median total HIT-6 score was 69 (IQR 9), higher in female (median 70, IQR 7; males showed a median of 63,5, IQR 8; p=,040). The percentage of MOH patients showing normal score on questionnaires was 38% for BDI-2 (83% for males and only 20% for female, p=,005), 68% for STAI-Y trait, 50% for TAS-20 and only 32% for DERS.

We found a significant correlation between total HIT-6 score and depression (BDI, p=,012) regulation of emotion (DERS, p=,014), trait anxiety (STAI-Y trait p=,012). We failed to find a correlation with alexithymia (TAS-20, p=,22) and with impulsivity (BIS-11, p=,15). No correlation was found between psychological questionnaires and medications taken per month.

Our results showed a high rate of depression and anxiety in MOH patients; moreover, they seem unable to recognize/regulate their own emotions: the analysis of subcomponents of DERS scale demonstrated that, in response to a negative emotion, MOH patients have difficulties in acceptance, concentration and maintaining control of their own behavior. Patients with DERS pathological score present higher score of impulsivity (BIS-11, p=0,08).

Conclusions: Assessing psychopathological profile in MOH patients is mandatory. We confirmed the comorbidity with mood and anxiety disorders in our group of patients suffering MOH. We showed that DERS scale correlates with the degree of disability and is useful for highlight specific maladaptive behaviors in MOH patients. We need a larger number of patients to be assessed by our set of questionnaires to confirm those preliminary results.

**References**

1. Radat F, Lanteri-Minet M. What is the role of dependence-related behavior in medication-overuse headache? Headache 2010, 50:1597–611.

2. Cupini LM, De Murtas M, Costa C, Mancini M, Eusebi P, Sarchielli P, et al. Obsessive-compulsive disorder and migraine with medication-overuse headache. Headache 2009, 49:1005–13.

3. Sarchielli P, Corbelli I, Messina P, Cupini LM, Bernardi G, Bono G, et al. Psychopathological comorbidities in medication-overuse headache: a multicentre clinical study. Eur J Neurol 2016, 23:85–91.

### P184 Education of the patient with migraine

#### Barbara Vitrani^1^, Lidia Savi^2^

##### ^1^Master’s Degree in Headache Medicine – City of Health and Sciences University Hospital - Turin- Italy;^2^Headache Center- City of Health and Sciences University Hospital - Turin- Italy/Headache Center LBS - Lugano – Switzerland

Migraine is associated with a decrease in quality of life and a heavy socioeconomic burden. In patients with migraine, education is essential to improve the clinical conditions, to reduce the use/overuse of analgesics and to increase adherence to both acute and preventive medications .

Aims of effective patient education should be: strengthen the doctor-patient relationship; improve medication adherence and reduce drop-outs; encourage patient participation to decision making favouring a joint responsibility approach; tailor personalized therapies.

We propose the following educational programme.

Pre-visit: ask to the patient to recall and note down relevant facts of his/her medical history, the main features of headache, possible triggering factors, treatments previously used and their efficacy.

During the visit: discuss the impact of headache on quality of life; communicate adequately and thoroughly the diagnosis; investigate patient expectations and needs; examine the possibilities and goals of treatment; reach an agreement on therapeutic strategy; hand over the headache diary and instruct the patient to fill it in; provide educational brochures as educational tools, if available; schedule follow-up visits.

Post-visit: recommend filling in the headache diary to monitor the effectiveness or failure of prescribed treatment, the clinical course, possible adverse effects, medication adherence, and use/overuse of rescue drugs; supervise positive changes of lifestyle; encourage contact with the specialist at any change of clinical conditions; ensure complete and prompt availability to any request of information; in case of drug abuse, provide explanations on specific treatment and possible hospitalization.

### P185 The Psycopathological comorbidities in children with chronic headaches

#### E.Tozzi, C.Gammella, A.Onofri, M.Mazzilli

##### Clinic of Child Neuropsychiatric- Regional center of Headache (Abruzzo), Hospital San Salvatore-University of L’Aquila Italy

###### **Correspondence:**E.Tozzi

**Background:** The trend in the prevalence of headaches shows an increase over the past 10 years, with a gap between the primary headaches underlying a significant increase chronic headaches. The quality of life is compromised and 1% of school absences are due to headache.[Tozzi et al, 2012]. The present study proposed to verify in the childhood that is more present in adulthood literature

**Materials and methods:** The sample of 121 children was divided on the basis of diagnosis of headache made according to ICHD-II and III criteria. Patients were selected in to Abruzzo Regional Headache Center,during 2015 and 2016 years. Investigated the following diseases: Sleep Disorders such as Parasomnie and Insomnie, Learning and/or School, Emotional and Neuropsychological Disorders. Used Tests: Cornoldi AC-MT Test, SAFA Test, Leiter, Corsi, CBCL.

**Results:** In the sample, 91 patients(pt) are suffering from Chronic Migraine(CM) (80%), 30 M and 61 F; 30 pt from Chronic Tension Type Headache(CTT) (20% ), 17 M and 13 f. In all the sample the percentage of sleep disorders is high : 57% of M and 63% of F with Migraine (CM), 56% of M and 71% of F with CTT. In M is greater the Bruxism as in M as in F, but in CTT, as in M as in F, the insomnia prevails ( 30% of F with M and 29% of F). The insomnia and the bruxism are factors of chronification and aggravation of headache.We found the significant correlation between headache and emotional disorders.In the M with CM there are externalizing disorders (10%), 6% of CTT show both internalizing and externalizing disorders, and 18% have externalizing disorders. Through the SAFA scales relative to the psychic state emerged that only groups with M have a psychopathological disorder.Only 3% are positive in the Depression assessment (D) scale and Psychogenic Disorder (P) scale, while 10% have only the risk of these pathologies in the same scale (DP) and the scale for symptoms obsessive compulsive (O). 5% of females has a risk in the scale of psychogenic eating disorders and 3% is positive on the same scale. In CCT 28% of children has a specific learning disorder; in Migraine learning disorders are present in 6% of M and 8% of F.

**Conclusions:** The sample studied shows significantly comorbility with Sleep Disorders. This relationship is controversial since Headache and Sleep Disorders can be a single disease.

### P186 Headache in the Pediatric Emergency Department: the importance of collaboration with Headache Centre

#### Roberta Rossi^1^, Antonia Versace^1^, Barbara Lauria^1^, Giulia Grasso^1^, Emanuele Castagno^1^, Fulvio Ricceri^2,3^, Rosaura Pagliero^1^, Antonio Francesco Urbino^1^

##### ^1^A.O.U. Città della Salute e della Scienza di Torino, Regina Margherita Children’s Hospital, Department of Pediatric Emergency, Pediatric Headache Centre, Turin, Italy;^2^Unit of Cancer Epidemiology, Department of Medical Sciences, University of Turin, Turin, Italy;^3^Unit of Epidemiology, Regional Health Service, ASL TO3, Grugliasco (TO), Italy

**Abbreviations:** Emergency Department (ED), Pediatric Headache Centre (PHC)

**Background:** The majority of headache episodes of childhood are benign and self-limiting, anyway they frequently cause multiple accesses to the Pediatric Emergency Department (ED) [1-4]. The collaboration between the Pediatric Headache Centre (PHC) and the ED of our hospital aims to limit such visits: according to the decision of the ED pediatricians, the patients with primary headache and those with repeated episodes of headache can be referred to the PHC within few days. Therefore, we have analyzed the data about the follow-up at our PHC and in particular the possible effect on repeated visits at the ED for headache, as there are very few studies investigating such relation [3].

**Methods**: All the patients ≤18 years referred to the ED of the Regina Margherita Children’s Hospital of Turin, Italy between January 2011 and December 2015, reporting headache as main symptom, were retrospectively reviewed. We screened the medical record databases of our ED and also the one of our PHC for information relative to those children who had been referred there after discharge from the ED.

**Results**: During the study period, 1833 patients accessed ED 2086 times: 1659 had one single access, 139 had 2 visits, and 25 had >2 visits; the median time between consecutive ED visits was 42 days (range 0-1433). On discharge, 646 patients (30.9%) received the indication to refer to the PHC, and 259 of them (40.0%) actually did.

A total of 115 patients had repeated visits within 3 months since the previous access, corresponding to 272 accesses (59.3% of repeated visits); 90 of such visits occurred within 10 days with a peak within 3 days. As regards the follow-up of the children who had multiple ED accesses for headache, 32 patients who had repeated visits within 3 months received the indication to refer to the PHC; 24 of them were actually followed-up, and only 1 of them accessed the ED again because of headache after starting the follow-up.

**Discussion**: Almost all the repeated visits within 3 months occurred in patients who had not been referred to the PHC after the first visit at our ED or in patients who received specific indication to follow-up, but did not attend it. Such observation suggests that the collaboration between the ED and the PHC is very important and might play a role reducing the so-called “revolving doors”.

**References**

[1] Lewis DW. Headache in the pediatric emergency department. Semin Pediatr Neurol. 2001;8:46-51.

[2] Kan L, Nagelberg J, Maytal J. Headaches in a pediatric emergency department: etiology, imaging, and treatment. Headache. 2000;40:25-9.

[3] Scagni P, Pagliero R. Headache in an Italian pediatric emergency department. J Headache Pain. 2008;9:83-7.

[4] Conicella E, Raucci U, Vanacore N, Vigevano F, Reale A, Pirozzi N, Valeriani M. The child with headache in a pediatric emergency department. Headache. 2008;48:1005-11.

### P187 Efficacy of KUZIK® in the prophylaxis of migraine without aura: a retrospective observational study

#### E.Pucci^1^, F. Solinas^2^, C. Mostardini^3^, M. Loiero^4^, D. Soragna^5^, R. Niego^6^, R. Galante^7^

##### ^1^Headache Science Center - University Consortium for the Study of Adaptive Disorders and Headache (UCADH), Department of Brain and Behavioral Sciences, University of Pavia, IRCCS “C. Mondino” Pavia;^2^Private neurological outpatient clinic, Sassari;^3^Center for Diagnosis and Treatment of Headache and Facial Neuralgia ,Ostia Hospital, Rome;^4^U.O. of Neurology, G. Pini Hospital, Milan; 5. Neurologist ASST Lariana;^6^Private neurological outpatient clinic, Verona;^7^Gam Farma Srl. Milan

###### **Correspondence:**E.Pucci

**Background**

A migraine without aura is the most common type of migraine headache ( about 60% to 80% of all migraines). In recent years there is growing interest in the use of nutraceuticals for the prevention of migraine, as there are no specific drugs for this indication. A new kudzu based food supplement (Kuzik), thanks to its innovative mechanism of action, has attracted our interest in this use.

**Materials and Methods**

Between January and April 2017, 30 patients with migraine without aura were treated with Kuzik, once a day for 2 months, recruited in various neurological structures including university centers, general hospitals, territorial structures and private outpatient clinic; for all patients, the diary was completed and full fill the headache card ( IHS criteria). Patients were evaluated at baseline and after 2 months regarding number of crisis, headache days and VAS (Visual Analogue Scale ): data relative to efficacy and tolerability were collected. Patient's characteristics: 25 female and 5 male; median age 34 (range 22-56 ); median years of disease 9,4 ( range 2-30 ) and previous treatments 1,2 ( range 1-2). At the baseline average number of crisis was 4 (range 2-10), headache days 7,4 ( range 3-14) and medium VAS 7,1 (range 5-10) .

**Results**

Kuzik was well tolerated: in a numerical scale from 1 to 10 the average score was 8 ( range 6 -10); the compliance of patients was optimal, no side effect related to product were reported and no weight gain in patients occurred. Regarding efficacy, at the end of treatment, all parameters analyzed were significantly improved : average number of crisis was 1,2 ( range 0-3 ), headache days 2 ( range 0-5 ) and medium VAS 1,9 ( range 0-4 ) .

**Conclusions**

Our study has clearly demonstrated the benefits, safety, and good tolerability of Kuzik in the prophylaxis of migraine without aura and the use of this medication should be taken in consideration as a good practice in this setting of patients. Data from our study are encouraging to be confirmed by further investigations.

### P188 Dural arteriovenous fistulas: clinical presentation, angiographic features and long-term outcome

#### Ilenia Corbelli MD^1^, Michele Romoli^1^, Francesca DeMaria^1^, Paolo Eusebi^1^, Gabriela Cardaioli^1^, Mohammed Hamam^2^, Piero Floridi^3^, Paola Sarchielli^1^, Paolo Calabresi^1,4^

##### ^1^Clinica Neurologica, Dipartimento di Medicina, Ospedale S.M. Misericordia, Università degli Studi di Perugia, Italy;^2^Servizio di Angiografia Interventistica, Ospedale S.M. Misericordia, Italy;^3^Servizio di Neuroradiologia, Ospedale S.M. Misericordia, Università degli Studi di Perugia, Italy; ^4^IRCCS Fondazione “S. Lucia”, Roma, Italy

###### **Correspondence:**Ilenia Corbelli (corbelli.ilenia@gmail.com)

**Background and aim of the study**

Dural arteriovenous fistulas (DAVFs) are intracranial vascular malformations. Their low presentation rate[1] justifies the limited availability of data about clinical features of these lesions.Aim of the study is to show a 10-year single institution experience with diagnosed and/or treated DAVFs, analyzing their clinical presentation and angiographic features, as well as their long-term outcome.

**Materials and Methods**

Looking for patients discharged from our Hospital in a 10-year period with the diagnosis of a “cerebral-vascular system abnormality”, we found 964 cases: 922 was excluded because they were vascular malformations of other subtype than DAVFs.Finally, we analyzed 42 intracranial DAVFs.For each one, we collected data about demographic characteristics, anamnesis and risk factors, clinical presentation, location and other neuroimaging features, as well as treatment and outcome.

**Results**

We found 42 DAVFs in 40 patients aged between 25-89 years at the time of the diagnosis. Twenty-one (52.5%) patients were women. Dividing all 42 DAVFs according to the angiographic features [2], we found 14 (33.3%) Carotid-Cavernous Fistulas (CCFs), 6 (14.3%) anterior cranial fossa (ethmoidal) DAVFs, 1 (2.4%) superior petrosal sinus DAVF, 18 (42.9%) transverse sigmoid junction DAVFs and 3 (7.1%) tentorial DAVFs.The most common complained symptom was headache (45.2%).Besides, a cerebellar/hearing/vestibular dysfunction was present in 28.6% of cases, especially in other DAVFs different from CCFs, although without a statistically significant difference between the two subgroups.

**Discussion and Conclusions**

Headache is a common onset symptom of DAVF, usually described as localized to the same site of the lesion, becoming generalized as a result of the dural stretching [2].Therefore, IHS has drawn up the classification criteria for the headache associated with DAVF [3]. Nevertheless, none of the previous studies on DAVFs have classified headaches according to the IHS classification criteria [3]. In our series of DAVFs, 45.2% of patients complained headache as a presentation symptoms, without a significant difference among the DAVFs subtype. Analyzing clinical features of headache, we found that 12/19 (63.2%) of patients had migraine-like headache, looking like a typical characteristic of other DAVFs different from CCFs (p=0.036). On the other hand, 7/19 (36.8%) patients complained not migraine-like headache, characteristics typical of CCFs (p=0.003).These findings suggest a link between the neuroradiological site of the lesion and the clinical features of the headache, symptom that led to hospitalization. Our study confirmed the majority of literature data about DAVFs, but it also provided significant insight about presentation symptoms, in particular regarding the characteristics of headache, which are suggestive of further dedicated studies.

**References**

1. Gandhi D, Chen J, Pearl M. Huang J, Gemmete JJ, Kathuria S (2012). Intracranial dural arteriovenous fistulas: classification, imaging findings, and treatment. Am J Neuroradiol. 33 (6): 1007-13.

2. Santillan A, Nanaszko M, Burkhardt JK, Patsalides A, Gobin YP, Riina HA. Endovascular management of intracranial dural arteriovenous fistulas: a review. Clin Neurol Neurosurg. 2013 Mar;115(3):241-51. doi: 10.1016/j.clineuro.2012.11.021. Epub 2012 Dec 31.

3. Headache classification committee of the international headache society (2013). The international classification of headache disorders, 3rd edition (beta version). Cephalalgia. 33:629-808.

### P189 Headache and the pregnant patient

#### Chiara De Pinto^1^, Lidia Savi^2^

##### ^1^U.O.Anestesia e Rianimazione 4, P.O.S.Anna, Città della Salute e della Scienza di Torino Corso Bramante 89, 10121 Torino, Italy;^2^U.O. Headache Center S.Giovanni Molinette University Hospital -Turin-Italy and Headache Center LBS, Lugano-Switzerland

###### **Correspondence:**Chiara De Pinto (cdepinto@cittadellasalute.to.it)

**Object**: we have analyzed headache during pregnancy, especially in puerperium.

**Content**: In pregnant patient, headache is a symptom caused frequently by primary forms but also by secondary forms [1]. During puerperium, about 30-40% of women experience headache in the first week after delivery; and the 5% of women feel headache for the first time in the first month after delivery [2,3,]. We have found in literature many studies about the physiopathology and clinical effects involved ,related and typical in the pregnant patient [4,5,6.7,8]. More it would be studied and understood yet.

**Methods**: we examined international literature from 1997 since the beginning of 2017, using Pubmed, Cochrane Library, EMBASE; we found meta-analysis, case reports, prospective and retrospective studies.

**Conclusions**: evaluation, knowledge and treatment of headache in obstetrical population are very important in the course of care: it would be multidisciplinary for the occurrence of comorbilities and to avoid near miss, it would be useful having a protocol, in rare but serious clinical situation.

**References**

1. Sanchez G et al.Course of migraine in pregnancy and postpartum:a prospective study. Cephal 2003;23:197-205.

2. oldszmithE et al. The incidence and etiology of postpartum headaches:a prospective cohort study.Can J Anesth 2005 Nov.;52:971-7.

3. Melhado EM, Maciel JA, Guerreiro CAM. Headache during gestation: evaluation of1101women.Can J Neurol Sci. May;2007;34:187-192.

4. Kvisik E et al Headache and migraine during pregnancy and puerperium :the MIGRA-study.J Headache Pain 2011 12: 443-451.

5. Marozio L et al. Headache and adverse pregnancy outcomes:a prospective study. Eur J Obst Gynec April2012 Vol161 pp140-143.

6. Levine RJ et al. CPEP study Group. Soluble endoglin and other circulating antiangiogenic factors in preeclampsia. NEJM 2006;355:992-1005

7. Maynard SE et al. Excess placental soluble fms-like tyrosine kinase 1 may contribute to endothelial dysfunction in preeclampsia J Clin Invest 2003;11: 649-658.

8. Kuklina EV et al. Trends in pregnancy hospitalization that included a stroke in USA from1994 to 2007 Stroke 2011;42: 2564-2570.

### P190 Botulinum toxins for the prevention of migraine in adults: the experience in headache Unit of Cassino

#### Ottavio Di Marco, Stefania Di Mauro, Fernando Ferrauti

##### ASL Frosinone

###### **Correspondence:**Ottavio Di Marco

**Background**: migraine is a severely debilitating desease, causing significant morbidity and negative impact on sufferers’ quality of life (Bigal 2008). Botulinum toxin was shown to be effective in treatment of chronic migraine (Kollowe, 2016)

**Materials and methods**: 24 patients, suffering from chronic migraine as defined by ICHD-3 ß criteria, subjected to at least 3 botulinum toxin treatments, were enrolled in the period between June 2015 and June 2017. All subjects were examinated at T0 and every six months with the following scales: SF-36, for assessing the perceived state of health, DISS, for the degree of disability and a diary, where patients recorded the intensity and frequency of pain and the number of days / month of migraine.

**Results**: the sample was mostly female (79.16%), had an average age of 42.3 years (ds ± 5.86) and mean time since diagnosis with CM was 10.5 years (ds ± 2,88). At T0 the sample had 13,6 days/month with migraine and hight vaue of disability expecially in work and family activities. SF-36 showed at T0 showed low value in physical role, general health and vitality, with a higher level of disability at DISS and average frequency of migraine episodes of 15.87 (ds ± 4.2) days per month. After 6 month we observed a reduction in the frequency and intensity of migraine, as well as an improvement in the quality of life. Such improvement becomes statistically significant to the 12-month evaluation in all the sub-dimensions of SF-36 (p < 0,05). After the fourth treatment, monthly headache days were reduced from 15.87 (ds ± 3.9) to 4.92 (ds ± 3.24) (*p* < 0.05), with statistically significant improvement in DISS values (p < 0,05).

**Conclusions**: The results showed the efficacy of botulinum toxins for the prevention of cronich migraine, with a improvement in quality of life. Significant improvement in the scales was obtained after 12 months of treatment.

**References**

Bigal ME, Serrano D, Reed M, Lipton RB. Chronic migraine in the population - Burden, diagnosis, and satisfaction with treatment. *Neurology* 2008; 71(8):559-66.

Kollewe K, Escher CM, Wulff DU, Fathi D, Paracka L, Mohammadi B, KarstM. Long-term treatment of chronic migraine with OnabotulinumtoxinA: efficacy, quality of life and tolerability in a real-life setting. Journal of Neural Transmission 2016; 123 (5): 533–540.

### P191 Maternal alexithymia and attachment style: which relationship with their children's headache features and psychological profile?

#### Samuela Tarantino^1^, Laura Papetti^1^, Cristiana De Ranieri^2^, Angela Rocco^2^, Valeria Valeriano^2^, Francesca Boldrini^2^, Barbara Battan^1^, Maria Francesca Paniccia^2^, Federico Vigevano^1^, Simonetta Gentile^2^, Massimiliano Valeriani^1,3^

##### ^1^Headache Center, Division of Neurology;^2^Unit of Clinical Psychology, Ospedale Pediatrico Bambino Gesù, IRCCS, Piazza Sant’Onofrio 4, Rome, Italy;^3^Center for Sensory-Motor Interaction, Aalborg University, Aalborg, Denmark

###### **Correspondence:**Samuela Tarantino (samuela.tarantino@gmail.com)

**Background.** A growing body of literature has showed a relationship between insecure “attachment style” and somatic symptoms. In a recent study, we found an association between ambivalent attachment style, migraine severity and psychological symptoms in children/adolescents. There is evidence that caregivers’ attachment styles and their way of management/expression of emotions can influence children’s psychological profile and pain expression. To date, data dealing with headache are scarce. We aimed to study the role of maternal alexithymia and attachment style on their children’s migraine severity (intensity and frequency) and psychological profile (anxiety, depression, somatization and attachment style).

**Materials and methods.** We enrolled 84 consecutive patients suffering from migraine without aura (female: 45, male: 39; age range 8-18 years; mean age 11.8 ± 2.4 years). Patients were divided into two groups according to frequency of the migraine episodes (high or low). According to headache attack intensity, patients were classified into two groups (mild and severe pain). Children’s psychological profile was assessed by SAFA Anxiety, Depression and Somatization scales. Attachment style was measured by the semi-projective test SAT for patients and ASQ questionnaire for mothers. Maternal alexithymia levels were evaluated by TAS-20.

**Results**. We found a significant higher score in maternal alexithymia levels in children classified as “ambivalent”, compared to those classified as “avoiding” (Total scale: p= 0.011). Alexithymia levels also correlated with children’s psychological profile. A positive correlation has been identified between mothers’ TAS-20 Total score and the children's SAFA-A Total Score (p=0.026). In particular, positive correlations were found between maternal alexithymia and children's “separation anxiety” subscale (p=0.009) and “school anxiety” (p=0.015). Maternal “externally oriented thinking” subscale correlated with SAFA-A “school anxiety” subscale (p=0.050). Moreover, we found a correlation between TAS-20 Total score and SAFA-D “Feeling of guilt” subscale (p=0.014). Our data did not show any relationship between TAS-20 and ASQ questionnaires and children’s migraine intensity and frequency.

**Conclusions**. Maternal alexithymia and attachment style have no impact on children's migraine frequency and intensity. However, our results suggest that, although maternal alexithymic traits don’t play a causative role on children’s migraine severity, they show a relationship with patients’ attachment style and psychological symptoms, which in turn may impact on migraine severity.

### P192 Vitamin D as a possible new treatment for migraine

#### Baoran Yang^1,2^, Giorgia Leodori^1,2^, Emma De Maio^1,2^, Guido Granata^1,2^, Massimo Granata^1,2^

##### ^1^Headache Centre, UOC Clinical Immunology A, Department of Clinical Medicine;^2^Department of Radiology, Policlinico Umberto I, “Sapienza” University of Rome

###### **Correspondence:**Massimo Granata (massimo.granata@uniroma1.it)

**Introduction:**

A number of studies recently reported an association between lower vitamin D levels and the worsening of the symptoms of migraine.

This association has been explained with the reduced neurotrophicity and the altered levels of CGRP, dopamine, noradrenaline and adrenaline observed with lower vitamin D levels.

Moreover, the presence of vitamin D receptors (VDR) and of 1-αhydroxylase (1a-OHase), the enzyme responsible for the formation of the active vitamin, has been recently demonstrated in many areas of the central nervous system[1].

As overviewed by Humble[2] et al., the vitamin D-endocrine system acts within the central nervous system as a neurosteroid, mediating multiple actions.

**Study aim:**

To evaluate the efficacy of vitamin D administration in mild migraine.

To evaluate the efficacy of a combined treatment with vitamin D administration plus traditional therapy in moderate and severe migraine.

To evaluate the effect of vitamin D administration on the mood tone in the study group.

**Material and Methods:**

45 patients aged 18-50 years and suffering from primary migraine were enrolled in the study.

Patients were divided into three main subgroup:

Group 1: patients suffering from mild migraine (no more than 4 days of disability per month and less than 5 attacks monthly). This group was treated with vitamin D administration only.

Group 2: patients with moderate-severe migraine (more than 4 days of disability per month or more than 5 attacks monthly). This group was treated with traditional therapy only.

Group 3: patients with moderate-severe migraine, treated with the combination of traditional therapy plus vitamin D administration.

Patients were followed for a period of nine months.

MIgraine symptomatology was monitored throughout the compilation of a diary in which patients reported the number and the intensity (using a Numeric Pain Rating scale ranging from 0 to 10) of the migraine attacks during the month. These two values were then combined in unified score, called intensity–weighted frequency score (IwF).

**Results:**

In the group 1, treated with vitamin D administration only, a reduction in migraine severity of 67% was observed at the end of the study.

The combined treatment (group 3) resulted 1,24 more effective in reducing migraine severity than traditional therapy alone (group 2) (Fig. 1).

The effect on the mood tone observed during the study was not statistically significative.

**Conclusion:**

Currently, mild migraine is normally treated with once-a-need anti-inflammatory drugs. In our study Vitamin D administration for the treatment of mild migraine resulted effective, with a decrease in the number of the attacks of 67%.

Study results did not support the hypothesis that vitamin D supplementation has an effect on the mood tone.

Combined treatment with vitamin D plus traditional therapy improved migraine symptoms of 69%, and this result suggests that vitamin D administration may improve the efficacy of traditional therapy alone for severe migraine (Fig. 2).

**Fig. 2 (abstract P192).**
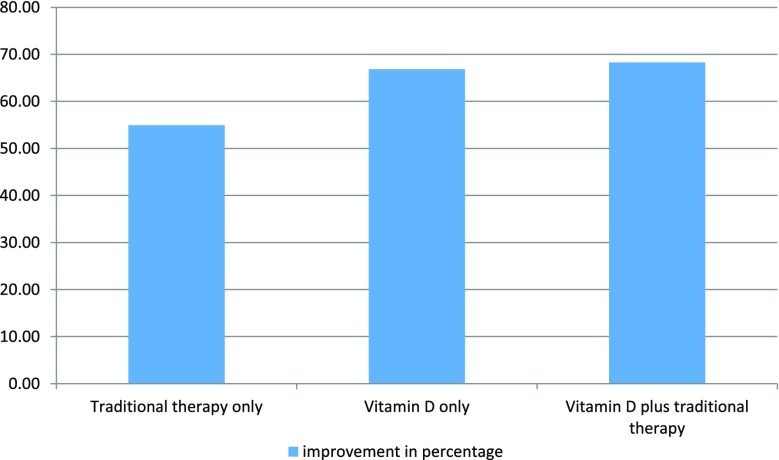
See text for description

**Fig. 1 (abstract 198).**
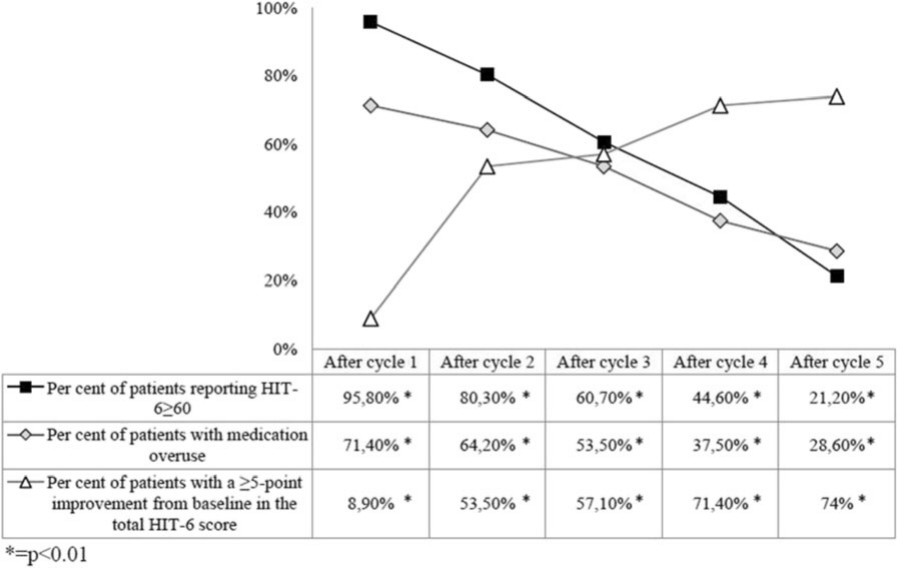
Evolution of HIT-6 scores and medication overuse with treatment cycles

**References**

1. Eyles DW, Smith S, Kinobe R, Hewison M, McGrath JJ (2005) Distribution of the vitamin D receptor and 1 alpha-hydroxylase in human brain. J Chem Neuroanat 29(1):21–30

2. Humble MB. Vitamin D, light and mental health. J Photochem Photobiol B 2010;101:142–149.

### P193 Clinical presentation and diagnostic evaluation of idiopathic intracranial hypertension in children and adolescents

#### Barbara Battan, Laura Papetti, Irene Salfa, Federico Vigevano, Massimiliano Valeriani

##### Headache Center, Child Neurology Unit, Bambino Gesu’ Children’s Hospital, Rome, Italy

###### **Correspondence:**Barbara Battan (barbara.battan@opbg.net)

**Background**

Idiopathic intracranial hypertension (IIH) or pseudotumor cerebri is a syndrome characterized by signs and symptoms of increased intracranial pressure in the absence of a secondary cause. The aim of the study is to report the IIH clinical presentation in children and adolescents presenting to our hospital during a 10-year period.

**Materials and methods**

Retrospective study, between January 2007 and January 2017, of IIH patients, younger than 15 years, was conducted. Modified Dandy criteria were used for IIH diagnosis. The patients were analysed according to age (≤10 and 11-15 years).

**Results**

Thirty-four patients, ranging from 3.8 to 15 years, were included. Thirteen patients were younger than 11 years (38.2%), while twenty-one patients were 11-15 years old (61.7%). Twenty-nine patients (85.2%) were obese (weight centile ≥ 90%). Mean cerebrospinal fluid opening pressure was 422 mm H2O (260-890 mmH2O). The most common presenting symptoms were headache (94.1%), vomiting (29.4%), dizziness (11.76%), blurred vision or diplopia (67.6%). Sixth nerve palsy occurred in 11 children (32.3%). In general, headache did not respond to pain medication. All our patients showed papilledema. Diagnostic evaluation included neuroimaging studies and ultrasound-based optic nerve sheath diameter (ONSD) measurement. In 6 patients (15%), MRI or CT showed signs of empty sella syndrome, while in 9 patients (26.4%) ultrasound ONSD measurement showed optic nerve sheath distension. There were no significant differences between the age groups in both clinical presentation and instrumental findings. Treatment included weight loss and acetazolamide (maximum 5mg/kg/die) in 28 patients (82.3%). Furosemide was added to acetazolamide in 2 patients (5.8%) and in 2 other patients was necessary added Delatcortene (5.8%). All patients fully recovered and none of them complained visual loss in the follow-up.

**Conclusion**

IIH should be considered in children with new-onset headache. Clinical headache presentation can be variable, although vomiting and visual symptoms are frequently associated. To exclude a secondary cause, neuroimaging should be performed. ONSD measurement may be useful as an additional tool to identify patients with IIH. Early diagnosis and treatment for IIH can prevent potential visual loss that remains the major morbidity. Acetazolamide and weight loss remain the most effective treatments in children.

### P194 Chronic paroxysmal hemicrania in paediatric age: report of four cases

#### Laura Papetti, Samuela Tarantino, Barbara Battan, Federico Vigevano, Massimiliano Valeriani

##### Headache Center, Department of Neuroscience, Bambino Gesù Children Hospital, Rome, Italy

###### **Correspondence:**Laura Papetti (laura.papetti@opbg.net)

Objectives. Chronic paroxysmal hemicrania (CPH) is a rare and well-characterised headache, classified amongst the trigeminal autonomic cephalgias (TACs). CPH has been only rarely and incompletely described in the developmental age. The objective of the present report was to describe the features of chronic paroxysmal migraine in four pediatric patients.

Objectives. Chronic paroxysmal hemicrania (CPH) is a rare and well-characterised headache, classified amongst the trigeminal autonomic cephalgias (TACs). CPH has been only rarely and incompletely described in the developmental age. The objective of the present report was to describe the features of chronic paroxysmal migraine in four pediatric patients.

Results. We detected 4 patients with CPH. Our children presented with a long history of severe and unilateral pain, which occurred in the fronto-orbital region without side shift. Attacks were accompanied by at least one autonomic symptom, ipsilateral to pain. During the attacks, besides conjunctival injection, eyelid oedema and rhinorrhea and all children showed a dramatic response to indomethacin.

Conclusions. Clinical symptoms and pain characteristics of our children are similar to those found in typical adult CPH. However our patients showed some atypical features, not fully meeting the ICHD-III beta criteria. First, although the ICHD criteria require an attack frequency higher than 5 attacks per day, in our patients, the attack frequency was lower. Second, attack duration was variable in all our children, but in three of four children it was sometimes longer than 30 min, which represents the maximal duration for a CPH attack.If attack duration and frequency can make the diagnosis more difficult, especially in paediatric age, the absolute response to indomethacin represents the diagnostic key for CPH in both adults and children (indotest).

### P195 Amitriptyline plasma level and its possible correlation with cognitive impairment and driving performance in migraine patients; pilot study

#### Matteo Bellamio^1^, Federico Mainardi^2^, Michele Barp^3^, Alberto Terrin^3^, Giorgio Zanchin^3^, Ferdinando Maggioni^3^

##### ^1^Headache Centre, Neurological Division, Dell’Angelo Hospital, Venezia-Mestre (VE), 30121 Italy;^2^Headache Centre, Neurological Division, SS Giovanni e Paolo Hospital, Venezia, Italy;^3^Headache Centre, Department of Neurosciences, University of Padua, Padova, Italy

###### **Correspondence:** Matteo Bellamio (matteo.bellamio@gmail.com)

**Background**

Headache is one of the most frequent neurological disorders; 90% of general population have at least one episode per year which is disabling in the 40% of cases. Most prevalent primary forms are tension type headache and migraine without aura and there are specific criteria and guidelines for their diagnosis and treatment [1]. When several days of pain occur, initiation of a prophylactic therapy is necessary. SISC Italian guidelines [2] suggest to use amitriptyline as one of first choices, which is a tricyclic antidepressant with well known possible disabling cognitive and effects and for this reason it is included in a specific list of drugs forbidden to drive car. The aim of this study is to validate a multimodal approach to assess the possible cognitive and driving effects of prophylactic therapy with amitriptyline in relation to its plasma level.

**Material and Methods**

We administered neuropsychological tests such as Minimental Status Examination, Continuous Performance Task and Berg version of Wisconsin Card Sorting Test to analyse cognitive profile. Driving skills were evaluated with a simulation in virtual-reality setting assessing the passive fatigue’s degree (Karolinska Sleepiness Scale, Standard Deviation of Lateral Position) and the reaction-time to visual and acoustic stimuli. Plasma assay were set to determine level of amitriptyline and its active metabolites in patients’ blood.

**Results**

Sample population (n=10) is mainly composed by females (80%) with mean age of 26 years; average body mass index and surface area are respectively 20,31 and 1,65 m^2^. Patients are all treated with amitriptyline 16 mg per os (most employed dosage in our Headache Centre) for at least three months. Preliminary results of all variables of the tests have been compared with normative values and with a control population (n=30) showing no significant changes on cognitive profile (p> 0,05). Plasma levels are at the lower limits of the means of detection sensitivity. Driving variables were compared with two control populations (n_1_=61, n_2_=24) and preliminary results seem to be not significantly influenced by daily administration of therapeutic dose of amitriptyline.

**Conclusions**

These results need to be confirmed studying a larger number of patients in order to give them a more specific and adequate information about effects of therapies in their daily-life activities, especially on driving. Moreover, our results will increase and will improve the community's toxicological and scientific knowledge offering a better service to the population.

**References**

1. Headache Classification Committee of the International Headache Society. The International Classification of Headache Disorders, 3rd edition (beta version). Cephalalgia 2013; 33: 629–808

2. Sarchielli P, Granella F, Prudenzano MP, Pini LA, Guidetti V, Bono G, Pinessi L, Alessandri M, Antonaci F, Fanciullacci M, Ferrari A, Guazzelli M, Nappi G, Sances G, Sandrini G, Savi L, Tassorelli C, Zanchin G. Italian guidelines for primary headaches: 2012 revised version. J Headache Pain. 2012 May; 13 Suppl 2:S31-70.

### P196 Subthalamic Deep Brain Stimulation performed for Parkinson disease treatment: effects on Chronic Migraine

#### Terrin A, Toldo G, Zanchin G, Mainardi F, Maggioni F

Deep brain stimulation (DBS) is an invasive therapeutic option for many neurologic disorders: epilepsy, Parkinson disease, obsessive-compulsive disorders, chronic cluster headache, neuropathic pain, depression resistant to pharmacological treatments (1).

The site of the implantation is the key for the therapeutic response: in each disorder, it is carefully selected after advanced neuroimaging evaluations (fMRI and PET studies) that highlight precisely the parts of the brain involved in the pathologic process (1). In Parkinson disease, as in many others conditions, these areas have been widely analyzed and the DBS has given remarkable results (1).

Among painful head diseases, DBS of the posterior hypothalamus has been investigated for the treatment of chronic cluster headache in medication-refractory patients (2). The posterior hypothalamus is thought to play an essential role in the pathophysiology of cluster headache, and likely accounts for the circadian and diurnal rhythmicity of both cluster periods (i.e. the months during which patients with episodic cluster have cluster attacks) and individual cluster attacks. Probably, the posterior hypothalamus covers an important role in the pathophysiology of the other trigeminal autonomic cephalalgias, as well (2).

As far as migraine pathophysiology is studied, it remains poorly defined and partially understood: although the areas involved in the development of migraine attacks are mainly localized in the brainstem (3), other structures, included the basal ganglia, seem to play a role during migraine attacks (4).

Because of this pathogenetic complexity, there are not well-defined targets to hit with DBS in migrainous patients. Therefore the few observations we have about DBS in migraine derived from case reports conducted on patients with different underlying conditions requiring treatment with DBS. For example, a recent case reported a significant improvement of chronic migraine after anterior thalamic DBS, performed for drug-resistant idiopathic generalized seizure (5).

We report the case of a 55 year-old-woman, who underwent DBS for Parkinson disease. The implantation interested both the subthalamic nuclei and produced an important improvement in her parkinsonian symptomatology, in particular in the movement impairment. The patient suffered from chronic migraine too: prophylactic treatments with topiramate 50 mg x 2 die and botulin injection were on course. Painful pattern did not present any positive or negative modification after DBS, as confirmed by a one-year clinical follow-up.

We believe useful to report this observation even if negative, since it allows collecting relevant and uncommon data: as this case suggests, subthalamic system stimulation seems not to determine significant changes on chronic migraine.

**References**

1) Lozano AM, Lipsman N Probing and Regulating Dysfunctional Circuits. Neuron 2013;77: 407-424.

2) Leone M, Cecchini AP Deep brain stimulation in headache. Cephalalgia 2016; 36:1143-1148.

3) Spenger T, Borsook D Migraine changes the brain. Neuroimaging imaging makes its marks. Curr Opin Neurol 2012; 25:252-262.

4) Maleki N, Becerra L, Nutile L, Pendse G, Brawn J et al. Migraine attacks the Basal ganglia. Molecular Pain 2011; 7:71.

5) Lendvai IS, Kinfe TM. Migraine Improvement After Anterior Thalamic Deep Brain Stimulation for Drug-Resistant Idiopathic Generalized Seizure: A Case Report. Headache 2017; 57:964-966.

### P197 Communication ed information of an Cephalagic patient in contemporary society

#### Gregorio Iannone^1^, Carlo Piccolini^2^, Sandro Bartoli^3^

##### ^1^Deputy coordinator S.I.S.C. Macroregione Umbria Abbruzzo Terni Italy;^2^A.O”S.Maria” Neuroscience and neurorehabilitation Department Terni Italy;^3^Dr. sociology Of communication us Perugia head office Terni Italy

The authors after history reference at communication in medicine do dwell among patient (and interacting people) with health figure (Not only medical) in the temporal dimensions of the past, present and future.

Detect than a correct interpretation is indispensable today, work’s tool fot the doctor.

Do dwell in “medicine narrative” for take the “experienced” framework painfull and it projection in the prospects of the future of the patient and the figures at it next.

Shall analyse on the specific the directives to respecton the patient with disorders pain that tend make chronic and the consequent aspects psychic they put on guard against risks of communication contemporary easily forgery-resistan and manipulable.

### P198 Stopping Onabotulinum treatment after the first 2 cycles might not be justified: results of a real-life monocentric prospective study in chronic migraine

#### Michele Romoli^1^, Ilenia Corbelli^1^, Laura Bernetti^1^, Angela Verzina^1^, Elona Brahimi^1^, Stefano Caproni^1^, Paolo Eusebi^2^, Cinzia Costa^1^, Paolo Calabresi^1,3^, Paola Sarchielli^1^

##### ^1^Neurology Clinic, University Hospital of Perugia, Perugia, Italy; ^2^Regional Health Authority, Public Health Regional Department, Perugia, Italy; ^3^IRCCS Santa Lucia, Rome, Italy

###### **Correspondence:** Michele Romoli (romoli.mic@gmail.com)

**Background**

Chronic migraine (CM) is a highly disabling headache disorder (1). Onabotulinum toxin A (OnabotA) is approved for the prophylactic treatment of CM, and consists of cyclic treatment every 12 weeks. Although response to OnabotA treatment varies among patients, current guidelines suggest to stop treatment after cycle 2 if no response has been experienced (2). This prospective study aimed to define, in real-life setting, the evolution of the response to OnabotA over 5 cycles of treatment among patients non-responders to cycle 1. The results of this study might help in decision making, in particular whether prosecuting OnabotA further or not, when facing a patient not responding to the first cycle of OnabotA.

**Materials and methods**

Continued OnabotA treatment among patients non-responders to cycle 1. Key outcomes: (i) a ≥50% reduction in moderate/severe headache days, (ii) a ≥50% reduction in total cumulative hours of headache on headache days and (iii) a ≥5-point improvement in HIT-6 scores.

**Results**

Overall, 56 patients were included, all non-responders to cycle 1. Mean age was 45.7 years (female 79.7%). Severe (≥60) HIT-6 score was reported at baseline by 95.8% of patients, with 75% of them incurring in symptomatic medications overuse. The headache days per month decreased significantly during the one year treatment period from cycle 1 to cycle 5 (overall from 23.3 ± 5.7 to 9.2 ± 3.6; p<0.001). During 12 months (5 cycles), migraine days per month progressively abated (from 18.5 to 8.7; p<0.001), symptomatic medications intake per day consistently decreased (from 17.4 to 8.1; p<0.001), and mean HIT-6 score lowered progressively (from 72.4 ± 5.7 to 50.2 ± 4.3; p<0.001) (Fig. 1).

Responders increased progressively from cycle 2 to cycle 5 (from 27% to 48%) (Fig. 2).

Over 5 cycles, patients regressing from CM to episodic migraine doubled (from 32% to 66%), with 78% of them reaching less than 9 migraine days/month.

**Conclusions**

The positive effect of OnabotA treatment seems to spread over the course of the treatment, and might also manifest late in treatment course among patients with no benefit after the first 2 cycles. The results of this real-life study suggest to consider extending OnabotA treatment further, beyond cycle 2, to avoid premature withdrawal in patients who would have become responders at cycle 3, 4 or 5.

**Fig. 2 (abstract 198).**
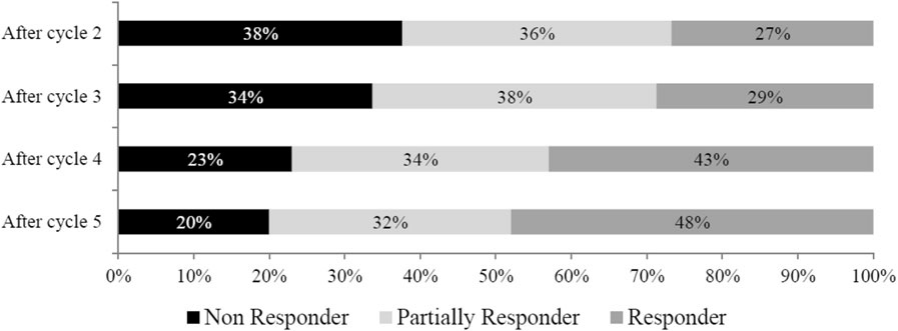
Evolution in response to OnabotA treatment over time

**Fig. 1 (abstract P207).**
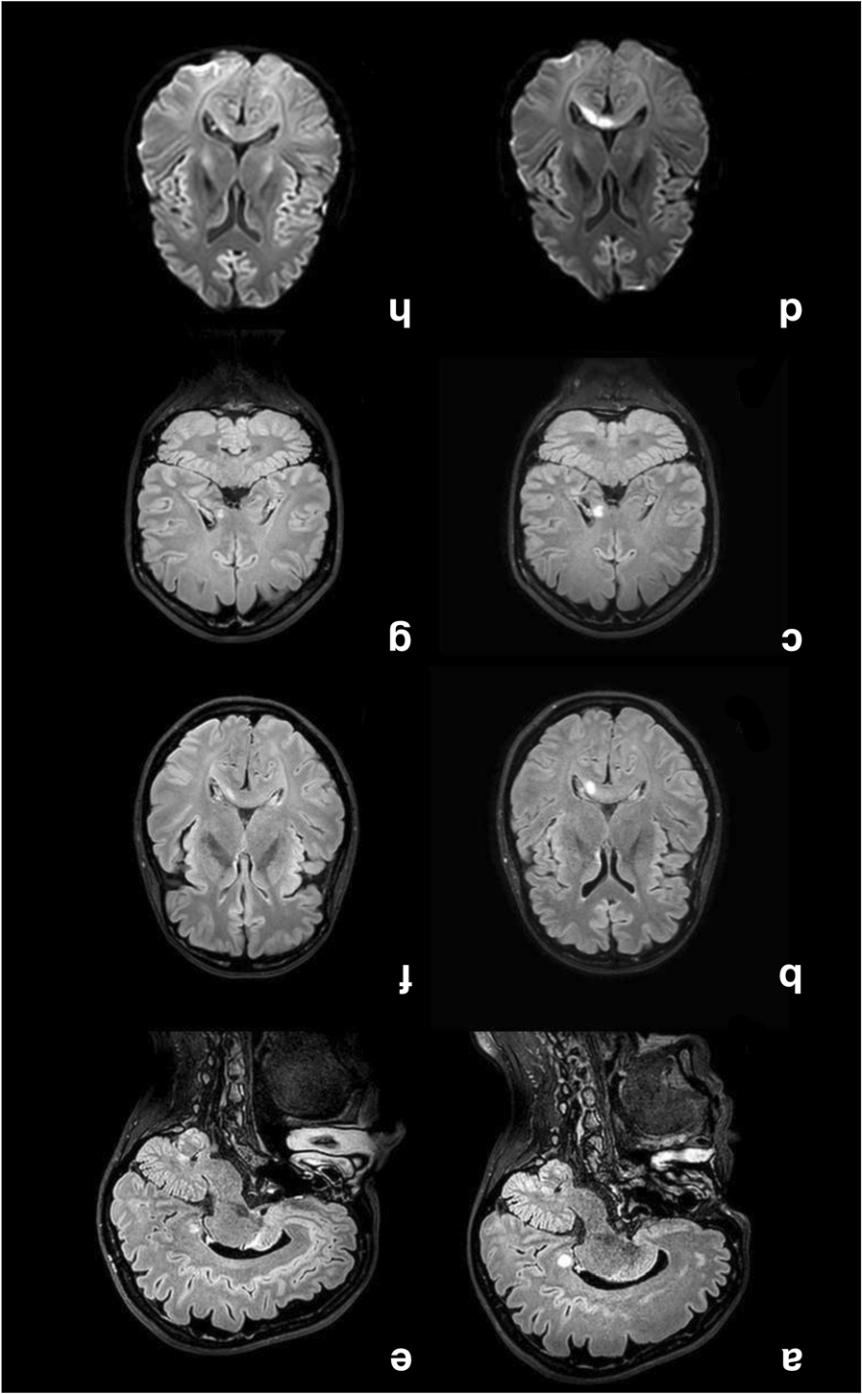
Neuroradiological evolution of the splenial lesion at repeated brain MRI scans, performed during hospitalization (**a**-**d**) and 6 months after the clinical onset (**e**-**h**). Fluid-attenuated inversion recovery sequences on a sagittal section (**a**, **e**), on a coronal one (**b**, **f**) and on an axial plane (**c**, **g**). Diffusion restricted images (**d**, **h**) show a resolution of the cytotoxic edema on follow-up MRI scans

**References**

1. Natoli J, Manack A, Dean B, Butler Q, Turkel CC, Stovner L et al. Global prevalence of chronic migraine: a systematic review. Cephalalgia 2010; 30:599–609.

2. Loder E, Burch R, Rizzoli P. The 2012 AHS/AAN guidelines for prevention of episodic migraine: a summary and comparison with other recent clinical practice guidelines. Headache 2012; 52(6):930-45.

### P199 Tanacethum Parthenium, 5-hydroxy tryptophan and magnesium (Aurastop®) in the prophylaxis of episodic migraine without aura in a adult population

#### Alessandra Mandelli, Natascia Beretta, Paola Merlo

##### U.O. Neurologia- Centro Cefalee Humanitas Gavazzeni, Beragamo

###### **Correspondence:**Alessandra Mandelli (alessandra. mandelli@gavazzeni.it)

**Background**

Tanacethum parthenium, 5 - hydroxy tryptophan and magnesium have an effect on various pathological mechanisms of migraine.

5 - hydroxy tryptophan (by his metabolite, kynurenic acid) and magnesium inhibit the activity of glutamate receptor NMDA, reducing cortical hyperexcitability; NMDA is also involved in peripheral and central sensitisation. Partenolide, the active component of Tanacethum parthenium, is an antagonist of TRP, the receptor that contributes to neurogenic inflammation by releasing CGRP. Moreover, it blocks 5HT2A and 5HT2B serotonin receptor and inhibits serotonin release by platelets, biosynthesis of prostaglandins and NOS expression.

Aim of this study is to prove the effectiveness of a novel combination between Tanacethum parthenium 150 mg, 5 - hydroxy tryptophan 20 mg and magnesium 185 mg (TP-5HT-M) Aurastop ® in the prophylaxis of migraine without aura with an episodic pattern.

**Materials and methods**

Twenty consecutive patients (F: n=12, M: n=8) of an age between 19-and 55 years (mean age 32.9) presenting with a ICHD 3 βeta diagnosis of migraine without aura with a frequency of less than 10 headache days a month were enrolled in the survey. Patients were suffering from episodic migraine for at least 6 months and they were not assuming any migraine preventive therapy. Patients were asked to report in the headache diary card frequency, intensity, and duration of attacks for 3 months after taking Aurastop ® tablet bid for the following 3 months. The reduction of migraine attacks per month was assessed as the primary end-point, while the secondary end-points considered were the reduction of headache days per month, the intensity of the headache (VAS scale) and the patient’s satisfaction using a scale of 0-5 where 5 was the maximum satisfaction.

**Results**

A significant reduction of migraine attacks per month (mean 2.8 vs 6.6) and number of headache days per month (mean 5.2 vs 8.5) was observed; positive the results regarding the intensity (VAS value 3,5 vs 8,5) and the satisfaction of the patients (4.5). No side effects were reported.

**Conclusion**

In this observational open study, the combining action of Tanacethum partenum, Mg and 5-HTP show to have a good therapeutic profile in headache control without any major side effects. Further studies are needed to confirm these preliminary results.

**References**

- Tassorelli C et al. Parthenolide is the component of Tanacethum partenum that inhibits nitroglycerin-induced Fos activation:studies in an animal model of migraine. Chepalalgia 2005; 25:612.

- Johnson ES, Kadam NP, Hylands PJ. Efficacy of feverfew as prophylactic treatment of migraine. Br Med J (Clin Res Ed) 1985, 291.

- Geppetti et al.: CGRP receptors and TRP channels in migraine. The Journal of Headache and Pain 2015 16 (Suppl 1):A21.

### P200 Migraine aura treatment with the association of Tanacethum Parthenium, 5-Hydroxytryptophan and Magnesium (Aurastop®): an observational study

#### Paola Merlo^1^, Grazia Sances^2^, Valentina Rebecchi^3^, Fabio Antonaci^2,4,5^, Andrea Giorgetti^6^, Franco Di Palma^7^, Edgar Matta^8^, Carlo Dall’Occhio^9^, Cristina Tassorelli^2,5^, Giorgio Dalla Volta^10^, on behalf of Società Italiana per lo Studio delle Cefalee (SISC–Lombardia) - Italia

##### ^1^U.O.Neurologia - Centro Cefalee, Humanitas Gavazzeni, Bergamo;^2^Headache Science Centre, Istituto Neurologico Nazionale Mondino, Pavia;^3^Centro Cefalee UOC Neurologia -Varese- ASST Settelaghi – Univ. Insubria;^4^UC Neurologia Speciale d'Urgenza, Istituto Neurologico Nazionale, Pavia;^5^Dipartimento di Scienze del Sistema Nervoso e del Comportamento Università di Pavia;^6^Centro Cefalee, Dipartimento di Neuroscienze H di Legnano ASST Ovest milanese;^7^Centro Cefalee UOC Neurologia della ASST Lariana-Ospedale S. Anna di Como;^8^Centro Cefalee UOC neurologia ASST Bergamo ovest;^9^UO Neurologia, ASST Pavia. Ospedale Civile, Voghera;^10^Centro Cefalee U.O Neurologia - Istituto Clinico Citta’ di Brescia, Brescia

###### **Correspondence:**Paola Merlo (paola.merlo@gavazzeni.it)

**Background**

A new phytotherapic combination of Tanacethum Parthenium (150 mg), 5-Hydroxy tryptophan (20 mg) and Magnesium (185 mg) (Aurastop®) is now available for migraine patients. The three components may tackle the main mechanisms involved in the pathophysiology of migraine with aura: Cortical Spreading Depression, sensitization of trigeminal vascular system, central sensitization.

The purpose of this open study was to evaluate the efficacy of the combined action of Tanacethum Parthenium, 5-Hydroxy tryptophan and magnesium in the reduction/disappearance of the aura phenomenon and reduction of its disability when taken at the aura onset.

**Materials and Methods**

Study of a population of 200 patients aged between 18 and 65 years (mean age:33.00 yrs), 117 F and 83 M, suffering from migraine with aura referred to 8 Lombardia Headache Centers. Patients were either without preventive therapy or without changing the prophylaxis during the aura treatment. Inclusion criteria: suffering of aura with at least 20 minutes duration. Patients retrospectively filled a aura diary for the description of the aura features in the past 3 episodes. Then they treated 3 consecutive aura attacks with a tablet of Aurastop® at the onset of the aura. In case of headache they were instructed to take another 1 tablet at the headache onset. The effect of the Aurastop® was prospectively recorded using the aura diary in order to evaluate: duration of the aura, disability caused by the aura, number/efficacy of habitual symptomatic treatment.

**Results**

We recorded a total of 600 aura episodes from a total of 200 subjects: Aurastop® produced more than 50% reduction in duration in 180 patients (90% of the total) and disability in 185 patients (92,5%). Notably 35% of the patients did not use other symptomatic treatment due to the fact that headache intensity was less severe after Aurastop®.

**Conclusion**

The combination of tanacethum partenum, Mg and 5-HTP (Aurastop®) could be an effective symptomatic treatment for migraine aura. Randomised controlled trials are still required to confirm these results.

**References**

- Curto M, Lionetto L, Negro A, Capi M, Fazio F, Giamberardino MA, Simmaco M, Nicoletti F, Martelletti P. Altered kynurenine pathway metabolites in serum of chronic migraine patients. Journal of Headache pain 2015 Dec; 17(1):47.

- Geppetti et al.: CGRP receptors and TRP channels in migraine. The Journal of Headache and Pain 2015 16(Suppl 1):A21

- Pietrobon D, Moskowitz MA. Chaos and commotion in the wake of cortical spreading depression and spreading depolarizations. Nat Rev Neurosci. 2014;15: 379–93.

### P201 Benign intracranial hypertension in children can be due to hypoparathyroidism: a case-report

#### Giorgia Sforza^1,3^, Annalisa Deodati^2^, Laura Papetti^1^, Barbara Battan^1^, Paolo Curatolo^3^, Federico Vigevano^1^, Massimiliano Valeriani^1^

##### ^1^Headache Center, Child Neurology Unit, Bambino Gesu’ Children’s Hospital, Rome, Italy;^2^Child Endocrinology Unit, Bambino Gesu’ Children’s Hospital, Rome, Italy;^3^Child Neurology and Psychiatry Unit, Tor Vergata University of Rome, Italy

###### **Correspondence:**Giorgia Sforza (sforzagiorgia@gmail.com)

**BACKGROUND**

To present the rare case of a 9-year-old girl with idiopathic intracranial hypertension (IIH) secondary to hypoparathyroidism (HPTH) and our work-up, including physical examination, blood tests, diagnostic imaging, and lumbar puncture.

**CASE REPORT:**

We present a 9-year old female patient who was hospitalized for headache associated with nausea and vomiting for 3 weeks. She underwent ophthalmologic examination which showed papilledema. She had never had cramps, paraesthesias or tetany. Lumbar puncture (LP) revealed an opening pressure of 65 cm H_2_O. CSF analysis and brain CT scan were normal. The patient was started on acetazolamide 375 mg/die. However, a low serum calcium level (6.3 mg/dL) was found, thus leading us to suspect HPTH. Indeed, phosphorus was 10.2 mg/dL, parathormone was very low (3 pg/mL). Chvostek and Trousseau signs scored positive. Neck ultrasonography showed normal thyroid, while parathyroids were not viewable. Oral supplementation with calcitriol (0.50 mcg/day) and calcium (500 mg/day) was started.

**CONCLUSIONS**

IIH is defined as an elevated intracranial pressure (>25 cmH2O) without clinical, laboratory or radiological evidence of hydrocephalus, infection, tumor or vascular abnormality. Annual incidence is 1-2 per 100,000. Several hypotheses have been proposed for the IIH pathophysiology, but none of them has reached a general consensus. Rare cases of IIH secondary to HPTH have been described ^[1]^. It is supposed that hypocalcemia causes a decrease in the CSF absorption at level of the arachnoidal granulations ^[2].^ Interestingly, our patient did not present with the typical neurological HPTH symptoms, such as tetany, cramps, paraesthesias, seizures, behavioral disorders, and intracranial calcifications. Only the serum calcium dosage led us to suspect this condition. Therefore, we recommend that possible HPTH should be always checked in children with clinical findings of benign intracranial hypertension.

**Consent to publish**

Written informed consent has been obtained from the parents.

**References**

1. Aragones JM, Alonso-Valdés F. Hipertensiòn intracraneal benigna secundaria a hipoparatiroidismo. Rev Neurology 2014; 58: 94.

2. Sambrook MA, Hill LF. Cerebrospinal fluid absorption in primary hypoparathyroidism. J Neurol Neurosurg Psychiatry 1977; 40:1015-7.

### P202 Efficacy in episodic migraine prevention of a combination of Tanacethum Parthenium, 5 - hydroxy tryptophan and magnesium (Aurastop©) A multicentric observational study

#### Paola Merlo^1^, Ferdinando Maggioni^2^, Giorgio Zanchin^2^, Federico Mainardi^3^, Giorgio Dalla Volta^4^

##### ^1^Headache Centre of Neurological Division of Gavazzeni Hospital, Bergamo;^2^Headache Centre, Department of Neurosciences, Padua University, Padua;^3^Headache Centre, Neurological Division, SS Giovanni e Paolo Hospital, Venice;^4^Headache Center of Neurological Unit of Istituto Clinico Citta’ di Brescia, Brescia

###### **Correspondence:**Giorgio Dalla Volta (dalla@numerica.it)

**Background**: Each component of the novel phytotherapic combination of Tanacethum Parthenium (150 mg), 5-hydroxy tryptophan (20 mg) and magnesium (185 mg) (Aurastop©) acts on a different target among the main mechanisms involved in the pathophysiology of migraine: sensitization of trigeminal vascular system, central sensitization and activation of the “migraine generator” located in the brainstem, through glutammate and kynurenine pathway. Aim of this study is to test the effectiveness of Aurastop© in the prophylaxis of migraine without aura.

**Materials and methods**: Sixty consecutive patients (F: n=37, M: n=23, mean age: 37.5±17.1) presenting with an ICHD-3 beta diagnosis of migraine without aura (MO) were enrolled in the survey and treated with Aurastop© twice a day for a period of 3 months. Diary cards were filled in during a 3-months period prior the beginning of the survey and during the 3-months duration of the study. A preventative treatment had been started previously and continued during the study in 5 cases (propranolol: n=2; amitriptyline: n=2; onabotulinumtoxin A: n=1).The reduction of MO attacks per month was assessed as the primary end-point; the reduction of headache days per month, the intensity of the pain and the patient’s satisfaction were considered as secondary end-points.

**Results**: A statistically significant reduction of both MO attacks and number of headache days per month was observed. Moreover, a sensible reduction of the intensity of the pain was reported. The secondary end-point regarding the satisfaction of the patients was achieved, as participants agreed when a new cycle of Aurastop© was proposed. No side effects were reported. The effectiveness appeared since the first month of intake and was maintained during the three months of therapy .

**Conclusion**: In this observational open study, Aurastop© appears to be effective and safe in the preventive treatment of MO.

**References**

Curto M, Lionetto L, Negro A, Capi M, Fazio F, Giamberardino MA, Simmaco M, Nicoletti F, Martelletti P. Altered kynurenine pathway metabolites in serum of chronic migraine patients. J Headache Pain. 2015; 17: 47.

Geppetti P, Bernabei S, De Cesaris F. CGRP receptors and TRP channels in migraine. J Headache Pain. 2015; 16(Suppl 1): A21.

Diener HC, Pfaffenrath V, Schnitker J, Friede M, Henneicke-von Zeppelin HH. Efficacy and safety of 6,25 mg t.i.d feverfew CO2-extract ( MIG-99) in migraine prevention – a randomized, double blind, multicenter, placebo controlled study. Cephalalgia. 2005; 25: 1031-41.

### P203 Palmitoylethanolamide: a possible new option for the prophylactic treatment of cluster headache

#### Carlo Lisotto^1^, Edoardo Mampreso^1^, Federico Mainardi^2^, Ferdinando Maggioni^1^, Giorgio Zanchin^1^

##### ^1^Headache Centre, Department of Neurosciences, University of Padua, Padua, Italy;^2^Headache Centre, Department of Neurology, Hospital of Venice, Venice, Italy

###### **Correspondence:**Carlo Lisotto

**Background** The aim of prophylactic treatment of cluster headache (CH) is to produce a suppression of attacks and to maintain a remission until the cluster bout is over, or for a longer period in patients with chronic CH. Verapamil is the drug of choice in both episodic and chronic CH. For short-term transitional therapy, corticosteroids are the most commonly prescribed medications [1]. Unfortunately, attacks almost invariably relapse when steroids are tapered.

Palmitoylethanolamide (PEA) is an endogenous fatty acid amide, which has been tested as an analgesic compound in a variety of patients with several pain conditions [2].

**Materials and methods** We evaluated 18 patients, previously treated with verapamil and note responding to transitional steroids. The study group included 10 males and 8 females, 13 suffering from episodic CH (7 M and 6 F) and 5 from chronic CH. The patients mean age at first observation was 49,3 ± 13,9, while the mean age at onset was 29,7 ± 15,5. The subjects with episodic CH had frequent bouts, at least one per year; the patients kept a diary card to record the number of attacks during the last two active periods in episodic CH and for the last three months in chronic CH. In episodic CH, patients refractory to steroids in previous clusters were treated at the onset of a new active period with a daily dose of 240-480 mg verapamil, associated with PEA in a single dose of 600 mg/day. In chronic CH the same regimen was commenced after the inefficacy of steroids for the previous three months was clinically demonstrated.

**Results** A reduction of attacks by at least 30%, as compared to baseline, was observed in 12 cases (66%). In two patients (one with chronic CH) the response was dramatic, with an almost complete headache remission. On average the cluster attacks decreased by 36%; no adverse events were reported.

**Conclusions** Patients with CH sometimes fail to respond to conventional treatments; therefore, due to this severely disabling condition, new emerging therapies for medically refractory CH patients are under active investigation [3]. PEA may be a promising option for CH refractory to steroids. The anti-inflammatory and analgesic effects of PEA have been confirmed in models of inflammation and neuropathic pain. Limitations of this study are the open-label design and the small patient population. Despite these caveats, our results warrant further investigations to test the possible efficacy of PEA for CH prevention.

**References**

1. Robbins MS, Starling AJ, Pringsheim TM, Becker WJ, Schwedt TJ. Treatment of cluster headache: the American Headache Society evidence-based guidelines. Headache. 2016; 56:1093-106.

2. Hesselink JM, Hekker TA. Therapeutic utility of palmitoylethanolamide in the treatment of neuropathic pain associated with various pathological conditions: a case series. J Pain Res. 2012; 5:437-42.

3. Tepper SJ, Stillman MJ. Cluster headache: potential options for medically refractory patients (when all else fails). Headache. 2013; 53:1183-90.

### P204 Association of Tanacethum Parthenium, 5 - hydroxy tryptophan and magnesium ( Aurastop) versus Mg tablet impact on aura phenomena and its evolution : an observational study

#### P. Zavarise, M. Manfredi, G. Ngonga, G.Dalla Volta

##### Headache Center. U.O Neurologia - Istituto Clinico Citta’ di Brescia - Brescia

Background

A new phytotherapic combination of Tanacethum Parthenium ( 150 mg ), 5 - hydroxy tryptophan ( 20 mg ) and magnesium ( 185 mg ) (Aurastop @) is now available for migraneous patients. The three components act on the four main mechanisms involved in the pathophysiology of migraine with aura: Cortical Spreading Depression, sensitization of trigeminal vascular system, central sensitization and activation of “migraine generator” at the brainstem’s level. Since many years Magnesium is well known to interact with the aura phenomena and migraine itself.With this study we want to compare the efficacy on the aura phenomenon and its disability of the combination of Tanacethum Parthenium, 5 - hydroxy tryptophan and magnesium versus magnesium alone, when taken at the beginning of the aura.

**MATERIALS AND METHODS:** We selected from the Headache Center of Istituto Clinico Citta’ di Brescia a population of 50 patients aged from 18 to 55 years (mean 31 years ), 27 women and 23 men, suffering from migraine with aura, not assuming migraine preventive therapy. They have to refer of an aura with a duration of at least 20 minutes to be included. We gave to the patients a form where they have to describe the aura features of the 4 aura episodes following the administration of 1 tablet of Aurastop at the beginning of the aura and 1 tablet at the beginning of the headache ( if present ) in the first 2 aura and 1 tablet of magnsium 2,25 gr in the same modality at the 3° and 4° episodes of aura. Patients were evaluated for duration and disability of the aura, need and response to their habitual analgesic drug

**RESULTS:** A reduction in duration greater than 50% in 48 patient versus 7 and of disability >50% in 48 patients against 5 were observed respectively after taking Aurastop o Magnesium alone .Furthermore 35% of the aurastop group did not have to take pain reliever after the aura as the headache intensity was more tolerable, only 5% of the magnesium group. We also noted a marked improvement in the benefit of the usual pain killer quite similar in both groups.

**CONCLUSIONS:** by the fact that these combination pass very quickly the ematoencephalic barrier Aurastop has been shown to have a quick impact on the evolution of aura reducing the duration and disability of symptoms than the magnesium alone.

**References**

Curto M, Lionetto L, Negro A, Capi M, Fazio F, Giamberardino MA, Simmaco M, Nicoletti F, Martelletti P. Altered kynurenine pathway metabolites in serum of chronic migraine patients. Journal of Headache pain 2015 Dec; 17(1):47.

Geppetti et al.: CGRP receptors and TRP channels in migraine. The Journal of Headache and Pain 2015 16(Suppl 1):A21

Pietrobon D, Moskowitz MA. Chaos and commotion in the wake of cortical spreading depression and spreading depolarizations. Nat Rev Neurosci. 2014;15:379–93.

### P205 Mother-child agreement on headache reports in a non-clinical sample

#### Cristiano Termine^1,2^, Matteo Chiappedi^3^, Chiara Luoni^4^, Micaela De Simone^5^, Sara Crugnola^4^, Beatrice Bartoli^1^, Andrea E. Cavanna^7,8,9^, Umberto Balottin^2,3,5^

##### ^1^Child Neuropsychiatry Unit, Department ofMedicine and Surgery, University of Insubria, Varese, Italy;^2^University Centre for Adaptive Disorders and Headache (UCADH), Sections of Varese and Pavia, Italy;^3^C. Mondino National Neurological Institute, Pavia, Italy;^4^Child and Adolescence Neuropsychiatry Unit, ASST SetteLaghi, Varese, Italy;^5^Department of Brain and BehavioralSciences, University of Pavia, Italy;^6^Child Neuropsychiatry Unit, CasimiroMondinoNational Neurological Institute, Pavia, Italy;^7^Department of Neuropsychiatry, BSMHFT and University of Birmingham, Birmingham, UK;^8^School of Life and Health Sciences, Aston University, Birmingham, UK;^9^University College London and Institute of Neurology, London, UK

###### **Correspondence:**Cristiano Termine (cristiano.termine@uninsubria.it)

**Background**

The prevalence of recurrent headache in children and adolescents, as reported in the literature, ranges from 4-20% in pre-schoolers to 57-82% in adolescents aged 15 years. This variability is related to differences in bothassessment methods (diagnostic criteria, data collection tools)and samples[1]. We set out to assess the level of agreement between mothers’ and children’s reports of headache (characteristics and frequency) in a non-clinical sample of children, through compilation of a medical questionnaire.

**Materials and methods**

We recruited a sample of 374 schoolchildren (mean age 9.58±0.2). All children were administered a standardised battery ofmedical questionnaires individually by a clinical expert, while their mothers filled in the questionnaires independently.

**Results**

Reports of at least one previous episode of headache were considerably more frequent in children’s than mothers’ questionnaires (68.7% vs 52.4%, k=0.01). For all the items considered in this group of subjects, the level of agreement was “poor” (k<0.20) or “fair” (k<0.40).

**Conclusions**

The low correlations found between the mothers’ and children’s reports on the frequency and characteristics of childhood headache suggest that questionnaire compilation by a single subject does not allow accurate estimation of the prevalence of headache, or a precise description of its features. The high variability in published prevalence estimates of childhood headache from non-clinical samples could be explained, at least in part, by these findings. Combined information from children/adolescents and their mothers could allow better estimation of the prevalence of headache in this age group.

**References**

1. Guidetti V, Galli F, Termine C. Headache in children. HandbClin Neurol. 2010; 97: 739-54.

### P206 Tanacethum Parthenium, 5-hydroxy tryptophan and magnesium in the prophylaxis of migraine without aura

#### Federico Mainardi^1^, Giorgio Zanchin^2^, Carlo Lisotto^3^, Ferdinando Maggioni^2^

##### ^1^Headache Centre, Neurological Division, SS Giovanni e Paolo Hospital, Venice;^2^Headache Centre, Department of Neurosciences, Padua University, Padua;^3^Headache Centre, San Vito al Tagliamento Hospital

###### **Correspondence:**Federico Mainardi (fmainardi@iol.it)

**Background**: Tanacethum parthenium, 5-hydroxy tryptophan and magnesium have an extensive literature supporting their role in the prophylaxis of migraine without aura. Aim of this study is to prove the effectiveness of a novel combination between tanacethum parthenium 150 mg, 5 - hydroxy tryptophan 20 mg and magnesium 185 mg (TP-5HT-M) in the prophylaxis of migraine without aura.

**Materials and methods**: Twenty consecutive patients (F: n=17, M: n=3) presenting with a ICHD 3 beta diagnosis of migraine without aura (MO) were enrolled in the survey. Age at onset of MO and mean age at the observation were respectively 18.7±9.4 and 45.8±13.1. Patients were asked to report in the headache diary card the frequency, intensity, and duration of MO attacks for 3 months; thereafter, such information was recorded and TP-5HT-M bid was administrated for the following 3 months. A preventative treatment had been started previously and continued during the study in 5 cases (propranolol: n=2; amitriptyline: n=2; onabotulinumtoxin A: n=1). The reduction of MO attacks per month was assessed as the primary end-point, while the secondary end-points considered were: *i*. reduction of headache days per month; *ii*. tolerability; *iii*. patient’s satisfaction.

**Results**: A significant reduction of MO attacks per month (mean 5.0 vs 7.9) and the number of headache days per month (mean 6.5 vs 9.8) were observed. No side effects were reported. All the subjects were satisfied and answered positively when a new cycle of TP-5HT-M therapy was proposed.

**Conclusion**: In this observational open study, TP-5HT-M combination appears to be safe and effective in the prophylaxis of MO. Further studies are needed to confirm these preliminary results.

### P207 Reversible splenial lesion of the corpus callosum in status migrainosus: a case report

#### Alberto Terrin^1^, Federico Mainardi^2^, Ferdinando Maggioni^1^

##### ^1^Headache Centre of the Veneto Region, Department of Neurosciences, University of Padova, Padova, Italy;^2^Headache Centre – Hospital SS. Giovanni and Paolo, Venice, Italy

###### **Correspondence:** Alberto Terrin (alberto.terrin89@gmail.com)

**Background**

Reversible splenial lesions of the corpus callosum (CC) have been reported in different central nervous system (CNS) disorders [1]. The prevalence of acquired CC lesions on brain MRI is estimated to be about 3% [2]. Migraine with aura and status migrainosus rarely correlate with this type or neuroradiological findings [3-5].

**Case report**

A 52-year-old woman was admitted for a status migrainosus. She was previously followed for episodic migraine without aura (ICHD-3). During the month preceding hospital-admission, the patient suffered from a gradual and significant worsening of her migrainous headache: it became daily and non-responsive to usual non-steroid-antinflammatory drugs. Subjective-objective dizziness appeared, followed by an episode of visual aura (never experienced before): she went to the Emergency Room (ER), where a brain and a cervical CT scan were performed, with no pathological findings. The hematological profile as the neurological examination were unremarkable. In the following days, further episodes of visual auras brought her back to the ER and to the admission to our ward. Her past medical history was unremarkable. She took no continuative therapy at home. A therapy with Ketoprofene, Diazepam and Indomethacin was started, with resolution of headache. Before dismission, a gadolinium-enhanced brain magnetic resonance imaging (MRI) showed an alteration of the signal of the splenium of the CC: a high-signal lesion on T2-weighted and fluid-attenuated inversion recovery images, with hyperintensity on diffusion-weighted images and minimal contrast-enhancement was present (Fig. 1a-d). CSF analysis was unremarkable except for an IgG mirror pattern at immunoelectrofocusing. A detailed immunological blood screening test was unremarkable. A PET-MRI brain imaging showed no hypermetabolism of the splenial lesion. After an efficacious composite prophylactic therapy with Candesartan, amitriptyline and Baclophene, and no other visual auras, a six-month control brain MRI scan showed an almost complete resolution of the splenial lesion (Fig. 1e-h).

**Conclusions**

For the first time we describe a reversible splenial lesion in an adult patient with status migrainosus, characterized by substantial reversibility only at a long-term MRI follow-up: a slow temporal evolution that has never been reported before. The pathophysiology of this type of lesion remains quite obscure: reversible demyeliation, extrapontine osmotic myelinolysis, intramyelinic edema, relative lack of adrenergic tone (with consequent susceptibility to hypoxic vasodilation), breakdown of the blood-brain barrier and high vulnerability to extracellular glutamate are some of the postulated hypotheses.

**Consent to publish**

The patient gave her written informed consent for the publication of this case report.

**Fig. 1 (abstract P211).**
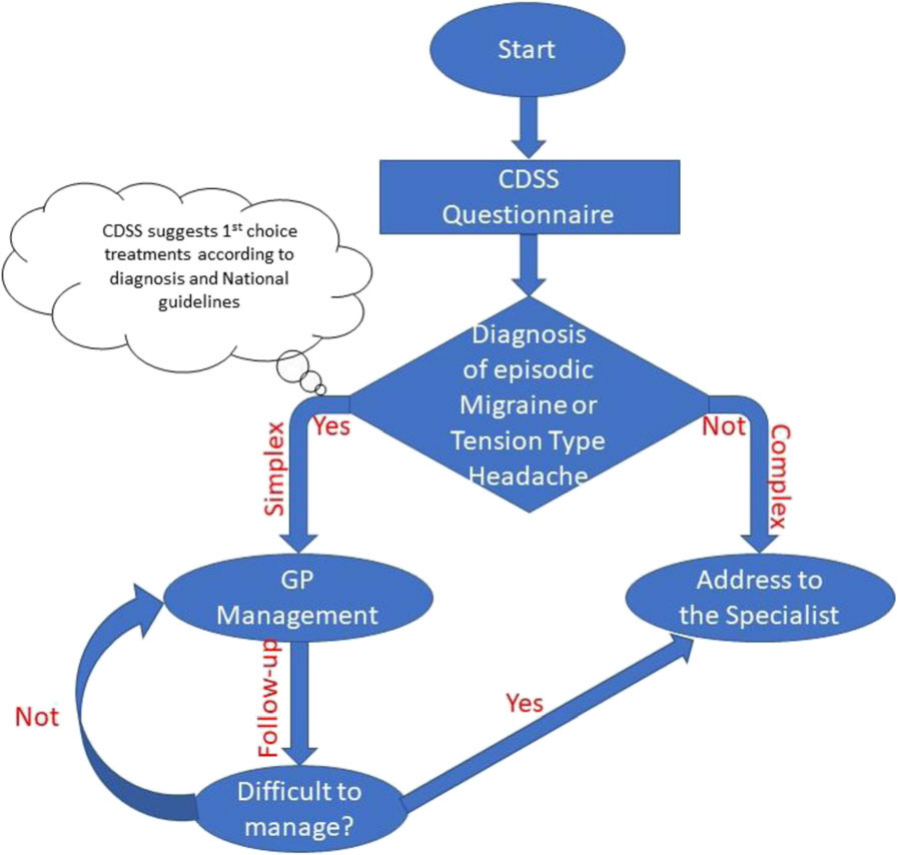
See text for description

**References**

1. Garcia-Monco JC, Cortina IE, Ferreira E et al. Reversible splenial lesion syndrome (RESLES): what's in a name? *J Neuroimaging*. 2011; 21:e1-e14.

2. McLeod NA, Williams JP, Machen B, et al. Normal and abnormal morphology of the corpus callosum. *Neurology.* 1987; 37:1240-1242.

3. Lin FY, Yang CY. Reversible splenial lesion of the corpus callosum in migraine with aura. *Neurologist.* 2011; 17:157-159.

4. Agarwal A, Kanupriya V, Maller V. Transient restricted diffusion in the splenium of the corpus callosum in migraine with aura. *Wien Klin Wochenschr*. 2012; 124:146-147.

5. Samanta D. Transient lesion in the splenium of the corpus callosum in status migrainosus. *Acta Neurol Belg*. 2015; 115:397-398.

### P208 Headache and its pharmacologic treatment during pregnancy: preliminary data of a retrospective cohort study (ATENA study)

#### Chiara Lupi^1*^, Elisabetta Gambassi^2*^, Tommaso Susini^2^, Pierangelo Geppetti^1^, Silvia Benemei^1^

##### ^1^Headache Centre, Careggi University Hospital, Department of Health Sciences, University of Florence, Florence, Italy;^2^Maternal and Child Department, Careggi University Hospital, Department of Health Sciences, University of Florence, Florence, Italy

###### **Correspondence:**Chiara Lupi

*Authors equally contributed

**Background**

During pregnancy, headache is mostly due to primary disorders, including migraine and tension-type headache. Pregnancy tends to ameliorate migraine that however may also begin or worsen in this condition [1]. Headache medications, because of the potential risks to the foetus, should be avoided during pregnancy. However, severe migraine is often not controlled by nonpharmacologic strategies, and many women, in the end, treat it with drugs, including paracetamol, nonsteroidal anti-inflammatory drugs (NSAIDs), and triptans [2]. Notwithstanding the large portion of women affected by headache hence candidate to drug consumption in pregnancy, only scarce evidence is available. Our study aims to investigate headache and its treatment during pregnancy.

**Materials and methods**

From July 12, 2017 to September 2, 2017, women either in pregnancy or who have delivered in the previous 7 days were prospectively screened at the Maternal and Child Department, Careggi University Hospital for enrolment in a retrospective cohort study, still ongoing. Patients who reported having experienced headache before and/or during pregnancy were asked for filling a self- administered questionnaire, adapted from Amundsen et al [3]. The study was approved by the competent Ethics Committee, and all patients gave their informed consent before enrolment.

**Results**

One-hundred (37%, median age 35) out of 271 screened patients reported a history of headache, prior to and/or during pregnancy, and filled the questionnaire. Headache onset was reported to be, on average, at the age of 17.3±6.3. Few women before pregnancy received a diagnosis by a physician of migraine (n=15) or tension-type headache (n=3). The mean frequency of headache attacks per month before pregnancy was 3.5, and in the first, second, and third trimesters of gestation, the mean frequencies were 2.3, 1.2, and 1.4, respectively. Eighty-six (86%) women reported pre-pregnancy use of drugs for headache, including NSAIDs (69 out of 86, 80%), paracetamol (26 out of 86, 30%), analgesic combinations (2 out of 86, 2%), and triptans (2 out of 86, 2%). In pregnancy, the percentage of women using pharmacotherapy decreased to 38% (38 out of 100), with a shift in the pattern of medication use in favour of paracetamol (35 out of 86, 41%).

**Conclusions**

Headache is common in pregnant women, especially in the first trimester. Although in pregnancy there was a reduction in the consumption of medicines with a change in the pattern of use, more efforts to improve diagnosis and pharmacologic treatment of pregnant women with headache should be undertaken.

**References**

1. Aubé M. Migraine in pregnancy. Neurology. 1999;53(4 Suppl 1):S26-8.

2. Nezvalová-Henriksen K, Spigset O, Nordeng H. Maternal characteristics and migraine pharmacotherapy during pregnancy: cross-sectional analysis of data from a large cohort study. Cephalalgia. 2009; 29:1267-76.

3. Amundsen S, Øvrebø TG, Amble NM, Poole AC, Nordeng H. Use of antimigraine medications and information needs during pregnancy and breastfeeding: a cross-sectional study among 401 Norwegian women. Eur J Clin Pharmacol. 2016; 72:1525-1535.

### P209 RegistRare: a retro-prospective registry of rare primary headaches in Italian tertiary Headache centres

#### Chiara Lupi^1^, Roberto De Icco^2^, Luana Evangelista^3^, Valentina Favoni^4^, Antonio Granato^5^, Edoardo Mampreso^6^, Matteo Paolucci^7^, Lanfranco Pellesi^4^, Andrea Negro^9^, Raffaele Ornello^3^, Antonio Russo^10^, Martina Ulivi^7^, Sabina Cevoli^4^, Simona Guerzoni^8^, Silvia Benemei^1^

##### ^1^Headache Centre, Careggi University Hospital, Department of Health Sciences, University of Florence, Florence, Italy;^2^Headache Science Center, C. Mondino National Neurological Institute, Pavia, Italy;^3^Headache Centre, San Salvatore Hospital, ASL Abruzzo 1, Avezzano-Sulmona-L’Aquila, Italy;^4^Institute of Neurological Sciences of Bologna, Bellaria Hospital,Bologna, Italy;^5^Department of Medical, Technological and Translational Sciences, Headache Centre, University of Trieste, Italy;^6^Headache Centre, Medical Department, AULSS 6 Euganea, Padova, Italy;^7^Institute of Neurology, Università Campus Bio-Medico, Rome, Italy;^8^Headache and Drug Abuse Research Centre, Policlinico Hospital,University of Modena e Reggio Emilia, Modena, Italy;^9^Regional Referral Headache Center, Sant'Andrea Hospital, Rome, Italy;^10^Headache Centre, Neurology Department, University of Naples II, Naples, Italy

###### **Correspondence:** Silvia Benemei

**Background**

Little is known about most rare primary headaches. Their diagnostic criteria are summarized in Chapter 3 and Chapter 4 of part one of the International Classification of Headache Disorders [1]. Due to the paucity of research on these disorders, which mainly comes from case reports or small cohort studies, and except for cluster headache, recommendations on diagnostic and therapeutic management are lacking [2]. In order to favour research, thus improving the knowledge, on these disorders and to promote a consensus about their management, we started up a registry aimed to collect nationwide data about patients that from May 1, 2014 have been diagnosed with a rare primary headache in Italian tertiary Headache Centres.

**Materials and methods**

We planned to collect data from May 1, 2014 and afterwards, by means of both retrospective and prospective approaches. All patients who have received or will receive a diagnosis of rare primary headache will be asked to participate and, to this aim, to give their informed consent. Each participating Centre should have received the approval of the competent Ethics Committee before commencing any study procedures. The registry will contain anagraphic information and data about diagnosis, treatment, comorbidity, and clinical features of the headache. Patients’ data will be coded through the Italian Health System unique identifier, in order to avoid duplications of patients referring to more than one Centre and also to monitor multiple referrals. A web-based open source platform (SurveyMonkey.org) will be used to collect data.

**Results**

Eleven Headache Centres were asked to participate and 10 have agreed to contribute to the retrospective portion of data collection as requested information can be easily retrieved from electronic sources used for clinical practice. Eight participating centres have obtained ethical approval and started study procedures, however up to date only 6 centres have already collected data with the retrospective approach. According to this latter dataset, in the period going from May 1, 2014 to April 30, 2017 a total of 15847 patients referred to the Centres, and 724 (4.5%) of them were affected by rare headaches.

**Conclusions**

Data collected in a portion of participating Centres make us confident that in a short period a quite large amount of data may be collected and analysed. The availability of a nation-based registry will importantly contribute to our knowledge of rare headaches and their management in the setting of Headache Centres.

**References**

1. Headache Classification Committee of the International Headache Society (IHS). The International Classification of Headache Disorders, 3rd edition (beta version). Cephalalgia. 2013; 33:629-808.

2. Holle D, Obermann M. Rare primary headaches. Curr Opin Neurol. 2014; 27:332-336.

### P210 Ketogenic diet is ineffective in chronic tension-type headache treatment

#### Cherubino Di Lorenzo^1^, Giulio Sirianni^2^, Gianluca Coppola^3^, Francesco Pierelli^4,5^

##### ^1^Don Carlo Gnocchi Onlus Foundation, Milan, Italy;^2^Delle Medical Center, Roma, Italy;^3^G.B. Bietti Foundation – IRCCS, Department of Neurophysiology of Vision and Neurophthalmology, Rome, Italy;^4^Department of Medico-Surgical Sciences and Biotechnologies, Sapienza University of Rome, Rome, Italy;^5^IRCCS – Neuromed, Pozzilli (IS), Italy

###### **Correspondence:** Cherubino Di Lorenzo (cherub@inwind.it)

**Background.** Since the 1928, ketogenic diet (KD) was proposed as possible preventive treatment for migraineurs.[1] Unfortunately, no data are still now available about the effect of KD in patients with tension-type headache (TTH), both episodic and chronic (CTTH). TTH is the most diffused form of primary headache. Usually CTTH disability is not severe; however, it is an open challenge in the field of headache management: other than amitriptyline, there are very few effective prophylactic treatments. Likewise, effective symptomatic options are very limited, and restricted to the non-steroidal anti-inflammatory drugs (NSAIDs), pending a class of drugs ad hoc developed, like triptans for migraine. In order to evaluate if, similarly to migraine, CTTH responds to KD, we observed a group of overweighed CTTH patients that underwent to a weight-loss KD.

**Methods**. Among overweighed patients self-referred to a dietician, with a positive anamnesis for headache ≥ 15 days/month, a neurologist specializing in headache recruited 29 CTTH patients. After one month of headache diary recording, they started a 1-month weight-loss program named very low-calorie ketogenic diet (VLCKD), characterized by a dramatic caloric restriction (<800 Kcal/day) and a very low-carbohydrate intake, able to lead to ketone bodies (KBs) production. At the end of the VLCKD, they started a weaning phase, to go out from ketogenesis. To verify variations in headache frequency during the month of VLCKD, we used as baseline the month before the diet.

**Results**. Out 29 enrolled patients, no one was regarded as responder (at least, 50% of headache reduction). At the baseline, headache frequency was 21.5 ± 4.4 days/month, while at the end of the VLCKD month it was 19.3 ± 5.6 (p=0.07). About the consumption of symptomatic drug doses, at the baseline, it was 11.9 ± 6.2 doses/month, while at the end of the VLCKD month it was 8.2 ± 5.6 (p=0.002).

**Conclusions**. Our results are suggestive for the absence of efficacy of KD in CTTH patients, unlike in episodic migraineurs. In fact, no one patient has halved the monthly headache frequency during the diet, nor did revert its chronic form of headache in episodic one. In terms of days/month with headache, there was a trend of reduction, unable to achieve the statistical significance. It is in line with a previous report on chronic daily headache.[2] On the contrary, the drug consumption improved, but it is unclear if it could depends by the medical advice to avoid ineffective drug treatments.

**References**

1. Schnabel T. An Experience with a Ketogenic Dietary in Migraine. Ann Intern Med. 1928;2(4):341–7.

2. Kossoff EH, Huffman J, Turner Z, Gladstein J. Use of the modified Atkins diet for adolescents with chronic daily headache. Cephalalgia. 2010;30(8):1014-6.

### P211 Clinical decision support software in headache management: a new tool for General Practitioners

#### Cherubino Di Lorenzo^1^, Fabio Adipietro^2^, Mario D’Uva^3^, Grazia Semeraro^4^, Gianluca Coppola^5^, Francesco Pierelli^4,6^

##### ^1^Don Carlo Gnocchi Onlus Foundation, Milan, Italy;^2^Freelance Engineer;^3^Medicina Primaria, Distretto 1, Azienda Sanitaria Locale, Latina, Italia;^4^Department of Medico-Surgical Sciences and Biotechnologies, Sapienza University of Rome, Rome, Italy;^5^G.B. Bietti Foundation – IRCCS, Department of Neurophysiology of Vision and Neurophthalmology, Rome, Italy;^6^IRCCS – Neuromed, Pozzilli (IS), Italy

###### **Correspondence:** Cherubino Di Lorenzo (cherub@inwind.it)

**Background.** The high prevalence of headache disorders is a challenge for the National Health System: not all the sufferers should be addressed to specialist consultation but general practitioners (GPs) are often not well trained to discriminate among their patients who needs neurological evaluation. To support the GPS in the headache patients’ management, we have created a clinical decision support software (CDSS). Aim of this study is perform the validation of this tool.

**Methods**. The CDSS consists of an 8 answers questionnaire (Table 1). According to the reply, patients were divided in 2 categories (Fig. 1): complex (who needs specialist consultation) and simplex (who can be managed by GPs). In the first group, we have comprised patients with chronic (>15 day/month), atypical, and trigeminal-autonomic headaches. In the second, episodic forms of tension type headache or migraine (with or without aura).

We have tested the CDSS by the direct interview to 201 headache patients referred to the Headache Clinic of Polo Pontino. An independent operator interviewed patients prior to the clinical evaluation, blind to the further diagnosis performed by the specialist. After the clinical evaluation, according to the diagnosis, also the specialist allocated patients in one of the two groups. The results of the CDSS were matched with the ones of the specialist.

**Results**. Out 201 patients, 115 received by specialist a diagnosis of episodic forms of tension type headache or migraine with or without aura (Simplex category); 86 patients have other forms of headache (Complex category). The results of CDSS were 98 Simplex forms, 103 Complex forms. The sensibility of the test was 96%, specificity 82%

**Conclusions**. The CDSS is an easy questionnaire that can be self-administrated by an informatic device, or by the GPs. Our results show that CDSS is a sensible test, able to detect patients needing to be addressed to a specialist consultation. In fact, only the 4% of patients with a Complex form of headache did not receive the indication to be addressed to the specialist visit. On the contrary, the 18% of patients with a Simplex form were anyway addressed by CDSS to the specialist. The underestimation of Simple forms reduces the specificity of the test; however, it makes possible to have a high sensibility, reducing the number of false positive cases. If implemented in the GPs clinical practice, this questionnaire could be a useful tool in support of clinical decisions about headache patients.

**Fig. 1 (abstract P219).**
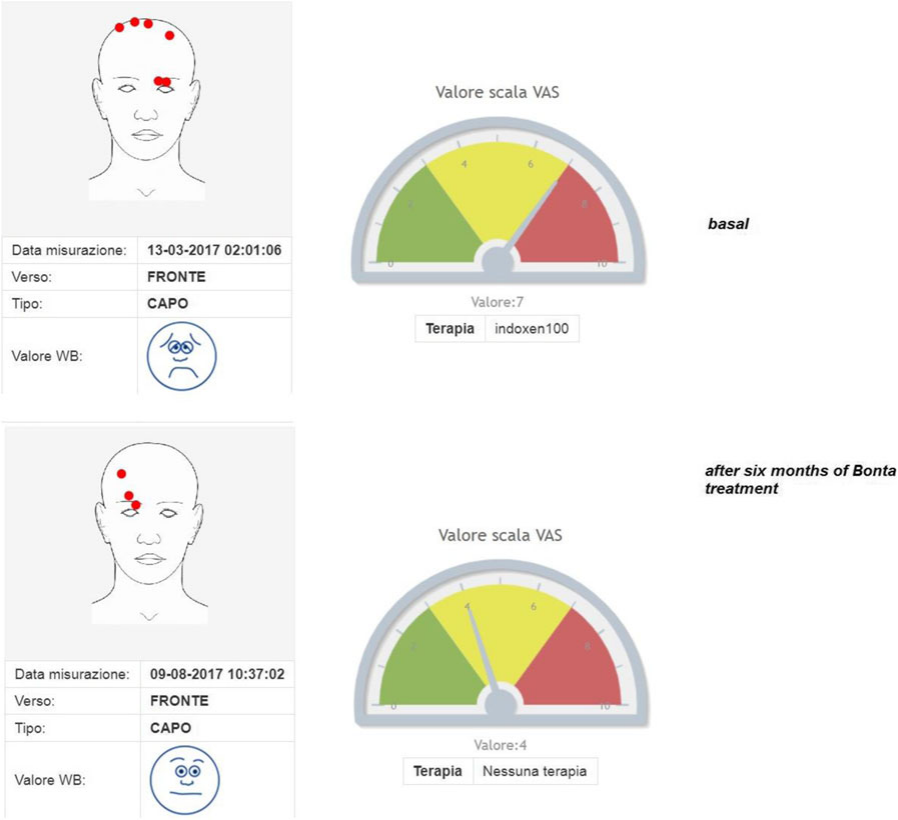
A representative report of a single chronic migraine patient, female, 43 years old, showing the reduction of headache extension and subjective sufferance after two cycles of Bonta treatment

**Table 1 (abstract P211).** See text for description

Questions	Answer
Nausea or Vomiting	- Yes - Not
Photophobia	- Yes - Not
Degree of disability	- Mild - Moderate - Severe
Gets worse with physical activity	- Yes - Not
Aura	- Always - Sometimes - Never
Duration	- <4h - 4-72h - >72h
Localization	- Unilateral, always on the same side - Unilateral, alternating - Other (median, bilateral, variable…)
Chronic (>15 days/month)	- Yes - Not

### P212

**Withdrawn**

### P213 Non pharmacological preventive therapy to chronic migraine in the real world: a Short-Term Psychodynamic Psychotherapy approach

#### Barbara Petolicchio^1^, Alessandro Viganò^1^, Federica Turchi^1^, Martina Squitieri^1^, Romina Di Giambattista^2^, Marta Altieri^1^,Edmond Gilliéron^2^, Vittorio Di Piero^1^

##### ^1^Department of Neurology and Psychiatry, Sapienza University of Rome, 00185 - Rome, Italy;^2^Istituto Europeo di Psicoterapia Psicoanalitica (IREP), 00187 - Rome, Italy

###### **Correspondence:**Barbara Petolicchio (barbara.petolicchio@uniroma1.it)

**Background**

Chronic migraine (CM) represents one of the leading causes of disability. It occurs in 2-5% of general population, often associated with medication overuse (MOH) [1,2]. Pharmacological preventive therapies in CM patients are often unsatisfactory for both efficacy and tolerance. The short-term psychodynamic psychotherapy (STPP), inspired by freudian psychoanalysis, associated with drug therapy, was more effective in patients with MOH to reduce headache symptoms and relapse rate than only pharmacological prophylaxis [3]. Moreover, in a pilot study on CM preventive therapy, STPP alone seems not to be inferior to sodium valproate [4]. We investigated a large cohort of CM patients treated with STPP to confirm the feasibility and clinical usefulness of this non-pharmacological approach.

**Materials and methods**

We recruited CM patients, with or without MOH, according to the ICHD-III beta criteria, attending to our Headache Clinic. Patients underwent a Brief Psychodynamic Investigation (BPI) formed by 4 meetings. BPI provides a diagnosis of psychodynamic profiles according to different mentalizing levels, i.e. ability to process the emotional content. In the following 2 months, 8 psychotherapy meetings were conducted. Follow-up controls were planned at 30 and 60 days. At this time, if patient still present a high headache burden, the most appropriate additional treatment was started. A final follow-up evaluation was planned after 6 months.

**Results**

We recruited 117 patients with CM (66,6% with MOH). Mean age was 40.3±14 years, headache/month number was 24±6.4, with a HIT-6 score of 63.7±9.8. By BPI, patients resulted having low mentalization. The STPP treatment was completed by 64 (55%) cases. After 2 months, they showed a significant improvement of headache: -33% headache days (14.7±8.7), -19% pain intensity, -39% MIDAS score, -7% HIT-6 score, -21% HAM-A and -21% HAM-D scores, and 91% of cases had no more medication overuse. Forty-two (65%) still had a high headache frequency (17±8.8 headache days/month; HIT-6 score:62±9.1) requiring additional preventive therapy.

At the 6-month control, patients showed a significant global clinical improvement with respect to baseline (-12.1 headache days/month). Patients treated with STPP alone had a reduction of -14.6 headache days/month with respect to -10.6 of those treated with STPP plus additional therapy at 6 months.

**Conclusions**

Our results suggest that STPP is an effective preventive treatment of CM, with or without MOH, either alone or in combination with other prophylaxis therapies. This therapeutic approach seems particularly effective in resolving drug abuse and its effect seems to remain constant over time.

**References**

1. Lipton RB. Chronic migraine, classification, differential diagnosis, and epidemiology. Headache. 2011; 51(2):77-83.

2. Antonaci F, Nappi G, Galli F, Manzoni GC, Calabresi P, Costa A. Migraine and psychiatric comorbidity: a review of clinical findings. J Headache Pain. 2011;12(2):115-25.

3. Altieri M, Di Giambattista R, Di Clemente L, Fagiolo D, Tarolla E, Mercurio A, Vicentini E, Tarsitani L, Lenzi GL, Biondi M, Di Piero V. Combined pharmacological and short term psychotherapy for probabile medication overuse headache: a pilot study. Cephalalgia. 2009;29(3): 29-39

4. Petolicchio B, Viganò A, Di Giambattista R, Squitieri M, Zanoletti N, Tortora D’Amato P, Spensierato A, Baldassarre M, Di Piero V. Short-term psychodynamic psychotherapy versus pharmacological treatment in chronic headache: an observational study. J Headache Pain 2013; 14 (S13-S41): 31

### P214 Chronic migraine preventive treatment by prefrontal-occipital transcranial direct current stimulation (TDCS): a pilot study on the effect of psychiatric comorbidities

#### Giulio Mastria^&^, Alessandro Viganò^&^, Alessandra Corrado, Cristina Pirillo, Valentina Mancini, Simone Badini, Barbara Petolicchio, Massimiliano Toscano, Roberto Delle Chiaie, Vittorio Di Piero

##### ^1^Neurology and Psychiatry, Sapienza - University of Rome, Rome, Italy

###### **Correspondence:**Alessandro Viganò (alessandro.vigano@uniroma1.it)

&Equally contributed

**Background**

Chronic Migraine (CM), with or without medication overuse headache (MOH), represents a therapeutic challenge in migraine management. These patients have frequently psychiatric comorbidities that may influence clinical outcome [1]. We investigated the correlation between psychiatric comorbidities and the effect of a non-invasive neurostimulation treatment, by transcranial direct current stimulation (TDCS), in patients with CM with or without MOH.

**Materials and Methods**

We recruited consecutive CM patients, who had had an unsatisfactory responses to at least 3 pharmacological preventive therapies, including OnabotulinumtoxinA. All patients underwent a psychopathological assessment (including Structured Clinical Interview, Akiskal’s Temps-A for bipolar spectrum, Young Mania Rating Scale [YMRS]). They were treated with anodal right prefrontal and cathodal occipital TDCS (intensity: 2mA, time: 20 min, three times a week for four weeks). Clinical migraine data were recorded at baseline (t-0), at the end of the treatment (t+30 days) and during follow-up (t+60 days). Post treatment psychiatric evaluation was planned within 2 weeks after the end of the TDCS.

**Results**

Sixteen patients (F=12, mean age=54 y.o.) with CM, with or without MOH were studied. We found 6 patients affected by bipolar disorder (BD), and 5 other patients with bipolar spectrum. In our series, the prevalence of BD and bipolar spectrum in resistant CM patients was approximately 70% compared to 11% reported in the literature for migraineurs. At t+60 days, patients showed a significant decrease of both severe headache days/month (6.13 to 4.56, -25%, p=0.005) and headache days/month (18.25 to 14.31, -22%, p=0.04). By considering the days of severe headache per month, patients with CM and BD had a baseline worse condition (11±5.05 vs. 3.91±5.10, p=0.02). At t+60 days, however, patients with CM and BD showed a greater response to TDCS for severe headache days (-3.4 vs. -0.73; -30% vs. -19%, p=0,009). Post stimulation psychiatric evaluation showed an improvement in YMRS score with a significant halving of baseline scores (p=0.03).

**Conclusions:**

TDCS seems to be effective in the treatment of CM patients, with or without MOH, which have a poor response to different classes of prophylactic pharmacological therapies. The prevalence of BD and bipolar spectrum in patients CM is higher than expected in both general population and migraine patients. BD patients showed a worse baseline headache burden as well as a better response to TDCS, both for headache and psychiatric parameters. In conclusion, BD psychiatric status might influence the response to TDCS, a non-pharmacological neuromodulation therapy.

**References**

1 May A, Schulte LH. Chronic migraine: risk factors, mechanisms and treatment. Nat Rev Neurol. 2016 Aug;12(8):455-64.

### P215 Prophylaxis of primary headaches with acupuncture, supplements or drug in children and adolescents: the Padua experience

#### Elena Piretti^1^, Pier Antonio Battistella^1^, Maria Paola Rossaro^1^, Margherita Nosadini^1^, Michela Gatta^2^, Stefano Sartori^1^, Irene Toldo^1^

##### ^1^Juvenile Headache Centre, Department of Woman's and Child’s Health, University Hospital of Padua, Via Giustiniani 3, 35128 Padua, Italy;^2^Socio-sanitary district, “Struttura Complessa Infanzia Adolescenza Famiglia” (SCIAF), ULSS 6 Euganea Padua, Via Dei Colli 4, 35143 Padua, Italy

###### **Correspondence:**Irene Toldo (irene.toldo@unipd.it)

**Background**

Prophylaxis of primary pediatric headaches has some limitations: poor efficacy, adverse effects, contraindications for comorbidity, off-label use of drugs in pediatric age, limited knowledge and few controlled studies.

The aim of the study is to describe the effect and tolerability of three types of treatments (acupuncture, supplements or drug) for prophylaxis of primary headaches in children and adolescents.

**Materials and methods**

Observational and prospective clinical study conducted at the Juvenile Headache Centre of Paduabetween October 2016 and August 2017.

Inclusion criteria: age 6-17 years; headache for at least 6 months; diagnosis according to the diagnostic criteria of the current International Classification of Headaches Disorders (ICHD-3 beta, 2013) of migraine without or with aura (> 4 attacks/month), chronic migraine, frequent episodic tension-type headache (> 6 attacks/month), chronic tension-type headache; prophylactic therapy with acupuncture or supplements (magnesium with multivitamins) or pizotifen. The last is the only drug licensed in Italy for migraine prophylaxis in children.

Outcomes: headache diary; PedMidas and CBCL 6-18 questionnaires; adverse events and unpleasant aspects; level of total self perceived satisfaction of the patient and the parents. Statistical analysis conducted with software SAS 9.3.

**Results**

73 patients were enrolled: 27 (acupuncture), 23 (supplements) and 23 (pizotifen). Significant results were summarized in Table 1.

**Conclusions**

Acupuncture improved both episodic and chronic headaches, associated symptoms, overall functioning and quality of life, and it was very well tolerated. Supplements had a good effect in reducing headache's frequency and drugs’ consumption, in both episodic and chronic headaches; however, this treatment was perceived as unpleasant by some subjects. Pizotifen showed limited positive effects on headache and on overall functioning and it did not reduce the analgesics’ consumption. Patients complained more side effects with pizotifen than the other two treatments.

In conclusion our data showed that acupuncture is a good prophylactic treatment of primary headaches in the developing age. Further studies are needed on larger series and with longer follow-up to assess the effect of these treatments on natural history of primary headaches.

**Table 1 (abstract P215).** Summary of the significant results obtained with the three treatments

	Acupuncture (N=27)	Supplements (N=23)	Pizotifen (N=23)
Frequency of the headache (gg/30 gg) pre e post-T (median)	From 9 to 3,5 (p=0.0002)^*^	From 12 to 1 (p<0.0001)^*^	From 4 to 3 (p=0.02)^*^
Duration of the headache (h) pre e post-T(median)	From 8 to 5,4 (p=0.0002)^*^	From 3,4 to 3 (p=0.58)	From 3,5 to 3 (p=0.18)
Nausea pre e post-T(median)	From 78,9% to 0% (p=0.0005)^*^	From 0% to 0% (p=0.49)	From 26,7% to 6,7% (p=0.22)
Photophobiapre e post-T(median)	From 32,5% to 26,5% (p=0.08)	From 28,6% to 0% (p=0.07)	From 75% to 0% (p=0.03)^*^
Phonophobia pre e post-T (median)	From 40% to 0% (p=0.005)^*^	From 37,5% to 0% (p=0.85)	From 100% to 25% (p=0.003)^*^
Symptomatic drug consumption pre e post-T (median)	From 4 to 1 (p=0.003)^*^	From 8 to 1 (p=0.0002)^*^	From 4,5 to 4 (p=0.15)
Items PedMidas°	^*^4	^*^2	^*^1
Items CBCL 6-18°	^*^1	^*^1	^*^1
Adverse and unpleasant effects (p=0.05)	6 (24%)	7 (70%)	4 (31%)
Satisfaction (*)	8,75	6	7
Positive secondary effects	7 (28%)	0 (0%)	0 (0%)

Legend: ^*^=p<0.05; T=treatment. Pre-T=2 months before, post-T=1 month after the end of T. Duration of T =3 months. °Number of items PedMidas e CBCL which improved after treatment. Satisfaction: reported on scale from 0 (unsatisfied) to 10 (extremely satisfied); (*) the differences between the 3 groups were statistically significant

### P216 Life traumas and stressful events in Chronic Migraine and Medication Overuse Headache: What is the relation with the outcome of a detoxification therapy?

#### Bottiroli Sara^1^, Viana Michele^1^, Sances Grazia^1^, De Icco Roberto^1^, Vito Bitetto^1, 2^, Guaschino Elena^1^, Ghiotto Natascia^1^, Pazzi Stefania^1^, Giuseppe Nappi^1^, Tassorelli Cristina^1,2^

##### ^1^Headache Science Centre, C. Mondino National Neurological Institute, 27100 Pavia, Italy;^2^Department of Brain and Behavioral Sciences, University of Pavia, 27100 Pavia, Italy

###### **Correspondence:**Bottiroli Sara (sara.bottiroli@mondino.it)

**Background**

Withdrawal from overused drug is the treatment of choice for subjects with Chronic Migraine and Medication Overuse Headache (CM+MOH) [1, 2], reverting the headache pattern from chronic to episodic within two months in the majority of subjects. Many factors are involved in the prognosis and outcome of these subjects, and their understanding is a topic of interest. CM+MOH patients experience increased psychiatric comorbidity, such as anxiety, depression, or personality disorders [3, 4], even if a cause-effect relationship still needs to be clearly delineated. Even less is known about the role of psychiatric factors in the response to detoxification treatments. In the present study we focused on early traumatic experiences and recent stressful events by investigating their association with the outcome of detoxification in a 2-month follow-up.

**Materials and methods**

This study was conducted at the Headache Center of the C. Mondino National Neurological Institute in Pavia, Italy. All consecutive patients with chronic migraine and medication overuse headache undergoing an inpatient detoxification program were enrolled and followed-up in a prospective study. Diagnosis was operationally defined according to ICHD-IIIβ. The protocol consisted in inpatient detoxification treatment and a 2-month follow-up. Data on early life traumatic experiences – of the physical or emotional type – and recent stressful events – rated according to the impact on quality of life, from mild to very serious – were collected by means of self-report questionnaires. Data were analyzed with the analysis of variance.

**Results**

Of the 166 patients who completed the 2-month follow-up, 118 (71%) stopped overuse and their headache reverted to an episodic pattern (Group A), 19 (11%) kept overusing and did not experience any change in headache frequency (Group B); and 29 (18%) stopped overuse without any benefit on headache frequency (Group C). At the multivariate analyses, a higher number of emotional traumas (OR 11.096; p = 0.037) emerged as a prognostic for the outcome in Group B; whereas having had history of major depression (OR 3.703; p = 0.006) and higher number of very serious stressful events (OR 1.679; p = 0.045) were prognostic for the outcome of Group C.

**Conclusions**

Our findings show the impact of life traumas and stressful events on the outcome of a detoxification program. The failure to cease overuse is related to the existence of childhood (mostly emotional) traumas, whereas recent life events, especially when very serious, do not influence the capacity of the patient to stop overuse, but are associated to the persistence of chronic headache. These observations underscore the need of a thorough psychological assessment of CM+MOH subjects and have possible implications in the nosographic framing of chronic headaches.

**Acknowledgements**

This research was supported by a Grant of the Ministry of Health to Mondino Institute (Current Research 2014-2016).

**Conflicts of interests**

None.

**References**

1. Evers S and Jensen R. Treatment of medication overuse headache – guideline of the EFNS headache panel. *Eur J Neurol.* 2011; 18: 1115–1121.

2. Olesen J. Detoxification for medication overuse is the primary task. *Cephalalgia*. 2012; 32:420-422.

3. Sances G et al. Factors associated with a negative outcome of medication overuse headache - a three-year follow-up (the “care” protocol). *Cephalalgia.* 2013, 33: 1-13.

4. Bottiroli S et al. Psychological factors associated to failure of detoxification treatment in chronic headache associated with medication overuse. *Cephalalgia.* 2016; 36: 1356-1365.

### P217 Thrombophilic disorders in migraineurs

#### Cinzia Cavestro^1^, Molinari Filippo^2^, Micca Gianmatteo^3^, Mandrino Silvia^1^, Diana Degan^4^, Francesca Pistoia^4^, Aloi Raffaele^5^, Frigeri MariaCristina^5^, Simona Sacco^4^

##### ^1^Headache Center, Dep. Of Neurology, “S.Lazzaro” Hosp, ASL CN2 Alba (Italy);^2^Main Laboratory and Hematology and Coagulation Disorders Laboratory, ASL CN2 Alba (Italy);^3^Main Laboratory and Hematology and Coagulation Disorders Laboratory, “Santa Croce” Hosp, Cuneo (Italy);^4^Department of Applied Clinical Sciences and Biotechnology, Section of Neurology, University of L’Aquila, L’Aquila, Italy;^5^Occupational Medicine Service, ASL CN2 Alba (Italy);^6^Administrative Medical Office, “S. Lazzaro” Hosp., ASL CN2 Alba (Italy)

###### **Correspondence:**Simona Sacco (simona.sacco@univaq.it)

**Background**

Migraine is often comorbid with several conditions including cardiovascular diseases [1,2]. The association between migraine and several thrombophilic alterations has been studied in migraineurs, giving variable results [3-5]. Aim of the present study was to assess the association between migraine and some thrombophilic disorders.

**Methods**

We included consecutive migraine patients referring to a tertiary headache center between September 1^st^ 2008 and March 31^st^ 2010. For each migraine patient a control subject without migraine, matched by age (±2 years) and gender, was selected among hospital employees. We excluded those people who were using anticoagulants. Selected thrombophilic disorders included deficiency in protein C (activity <60%), deficiency in protein S (activity <62%), activated protein C resistance (APCR; if APC ratio below 2.0), antiphospholipid antibodies (aPL) positivity (lupus anticoagulant in plasma and/or anticardiolipin antibodies titre > 99% and/or Ab-antiβ2GP1 titre > 99%, on two or more occasions at least 12 weeks apart and within 6 months), and hyperhomocysteinemia (above 15 μmol/L).

**Results**

We included in the study 329 subjects with migraine (37.7% with aura) and 329 controls. Mean age±SD was 40.9±12.0 for migraineurs and 41.1±10.4 for controls. In both groups 254 subjects (77.2%) were women. Characteristics of included subjects are shown in Table 1.

Deficiency in protein S (5.5% vs 1.2%; OR 4.7, 95% CI 1.6-14.0; P= 0.002) and aPL positivity (12.5% vs 5.2%; OR 2.6; 95% CI 1.5-4.7; P=0.001) were more common in migraine patients than in control subjects whereas we found no differences in deficiency in protein C (0.3% vs 0.6%; OR 0.5, 95% CI 0.1-5.5; P= 0.556), APCR (3.3% vs 3.4%; OR 1.0, 95% CI 0.4-2.3; P=0.977), and hyperhomocisteinemia (15.9% vs 15.1%; OR 1.1, 95% CI 0.7-1.6; P=0.784) between the two groups. Migraine patients having deficiency in protein S were younger than those without the deficiency and had more often history of cerebrovascular diseases (OR 7.1, 95% CI 2.0-24.9; P=0.002) whereas other comorbid conditions and headache characteristics (age at onset, frequency or severity) were not different. Migraine patients having aPL positivity had more often history of cerebrovascular diseases (OR 4.8, 95% CI 1.6-16.9; P=0.004) and ischemic heart disease (OR 22.7, 95% CI 2.3-223.4; P=0.008) than those not having the condition. In an exploratory analysis we did not find any difference in the thrombophilic disorders between subjects with migraine with and without aura.

**Discussion**

Migraine is comorbid with some thrombophilic disorders and particularly with deficiency in protein S and aPL positivity. Migraine patients with those thrombophilic comorbidities reported more often history of vascular disorders in the cerebral or cardiac districts. Our data may indicate that some thrombophilic disorders may be associated with occurrence of vascular events in a subset of migraineurs patients.

**Funding**

This study was funded by Regione Piemonte funds for targeted research.

**Conflict of Interests**

The authors do not have any conflict of interest.

**References**

1. Sacco S, Ornello R, Ripa P, Tiseo C, Degan D, Pistoia F, Carolei A. Migraine and risk of ischaemic heart disease: a systematic review and meta-analysis of observational studies. Eur J Neurol 2015;22:1001-1011.

2. Sacco S, Ricci S, Carolei A. Migraine and vascular diseases: a review of the evidence and potential implications for management. Cephalalgia 2012;32:785-795.

3. Cavestro C, Mandrino S. Thrombophilic disorders in migraine. Front Neurol 2014;5:120.

4. Cavestro C, Micca G, Molinari F, et al. Migraineurs show a high prevalence of antiphospholipid antibodies. J Thromb Haemost 2011;9:1350-1354.

5. Tietjen GE, Collins SA. Hypercoagulability and Migraine. Headache 2017;doi: 10.1111/head.13044.

### P218 Clinical pharmacokinetics of oral cannabis FM2 preparations in medication overuse headache (MOH) patients

#### Lanfranco Pellesi^1^, Simona Guerzoni^1^, Manuela Licata^2^, Luigi A Pini^1^

##### ^1^Medical Toxicology and Headache Centre, University of Modena and Reggio Emilia, Modena, 41124, Italy;^2^Forensic Toxicology Laboratory, University of Modena and Reggio Emilia, Modena, 41124, Italy

###### **Correspondence:**Lanfranco Pellesi (lanfranco.pellesi@gmail.com)

**Background**

The plant Cannabis sativa has a long history as an analgesic medication in chronic pain, neuralgia and migraine [1]. It contains more than 60 phytocannabinoids, all ligands of the endogenous cannabinoid receptors. The active substance decisive for the psychoactive and analgesic effects is delta-9-tetrahydrocannabinol (Δ^9^-THC, or THC). At the same time, other cannabinoids, such as cannabidiol (CBD), have shown promising anti-nociceptive properties [2]. Currently, the interest in cannabinoids for the treatment of neuropathic pain is increasing, because they have improved post-surgical pain, as well as migraine symptoms, with a safety profile comparable to the most widely used pharmacological therapies [3]. However, the fear of dependence and psychiatric disorders limits their use and investigating. Given the recent possibility to prescribe an Italian cannabis strain (FM2, 5-8% THC and 7-12% CBD), we plan to study the pharmacokinetics and clinical effects of a single oral dose of Cannabis FM2 taken as a decoction or in olive oil.

**Material and Methods**

In an open-label two-period crossover study, 7 patients with MOH diagnosed according to International Criteria [4] received a single oral dose of cannabis decoction and, two weeks later, a single oral dose of cannabis oil. Blood samples for THC, CBD and their metabolites in whole blood were collected up to 24 hours after the intake of each preparation. THC, CBD and their metabolites were determined using chromatography coupled to mass spectrometry (LC-MS/MS). Tolerability and adverse events were assessed by numerical rating scale (NRS) about defined sensations. Pharmacokinetic parameters were determined by non-compartmental methods, using PK solver [5]. Statistical analysis was performed using STATAIC-13.

**Results**

The pharmacokinetics properties of the Cannabis edible products were significantly different (Table 1). Specifically, the active ingredients THC and CBD have shown different kinetic properties depending on the oral solution in which they are taken. The cannabis oil showed lower THC whole blood AUC_24 h_ and C_max_ than the cannabis decoction (respectively, p=0.01128 and p<0.01). On the other hand, the median CBD whole blood T_max_ was higher for the cannabis decoction, compared to cannabis oil (p=0.04653). No side effects were observed, except for a high drowsiness one hour after the administration of cannabis oil (p<0.01).

**Conclusions**

A single oral dose of Cannabis FM2 demonstrated different pharmacological properties depending on whether it is taken as a decoction or in olive oil. Further studies are needed to assess the real usefulness of these therapies in clinical practice.

**Table 1 (abstract P218).** Descriptive summary of cannabinoid pharmacokinetic parameters

Parameters (mean ± SD)	Cannabis decoction (200 ml)	Cannabis oil (1 ml)	*p*-value
Δ^9^-THC			
T_lag_, hours	0.57 ± 0.19	0.71 ± 0.27	0.356
T_max_, hours	1.07 ± 0.35	1.29 ± 0.39	0.200
C_max_, ng/ml	1.58 ± 0.72	3.76 ± 1.15	**< 0.01**
AUC_0-24_, ng/ml · min	3.63 ± 1.84	8.39 ± 2.83	**0.011**
t_1/2_, hours	1.68 ± 1.55	1.65 ± 0.75	0.964
11-OH-THC			
T_lag_, hours	0.86 ± 0.63	1.21 ± 0.49	0.283
T_max_, hours	1.00 ± 0.71	1.43 ± 0.45	0.143
C_max_, ng/ml	0.49 ± 0.41	1.19 ± 0.85	0.054
AUC_0-24_, ng/ml · min	0.85 ± 0.84	1.98 ± 1.41	**0.044**
t_1/2_, hours	0.70 ± 0.51	1.90 ± 2.70	0.33
THC-COOH			
T_lag_, hours	1.07 ± 0.35	1.21 ± 0.27	0.356
T_max_, hours	2.00 ± 0.76	2.14 ± 1.03	0.752
C_max_, ng/ml	4.74 ± 1.70	8.13 ± 4.74	**0.037**
AUC_0-24_, ng/ml · min	22.61 ± 15.05	44.25 ± 42.26	0.203
t_1/2_, hours	3.80 ± 3.99	6.29 ± 6.38	0.452
THC-glucuronate			
T_lag_, hours	1.36 ± 0.24	1.21 ± 0.27	0.356
T_max_, hours	4.43 ± 1.13	3.14 ± 0.69	**0.035**
C_max_, ng/ml	30.47 ± 8.23	31.92 ± 9.34	0.471
AUC_0-24_, ng/ml · min	453.69 ± 86.48	556.66 ± 258.97	0.208
t_1/2_, hours	20.75 ± 5.69	20.05 ± 7.23	0.843
CBD			
T_lag_, hours	0.50 ± 0.00	0.79 ± 0.39	0.103
T_max_, hours	0.57 ± 0.19	0.93 ± 0.35	0.047
C_max_, ng/ml	5.32 ± 2.80	3.49 ± 2.81	0.256
AUC_0-24_, ng/ml · min	5.16 ± 2.46	3.35 ± 2.09	0.157
t_1/2_, hours	0.53 ± 0.19	0.52 ± 0.31	0.923

Data were analysed with paired T-test (2 dependent means)

*SD* standard deviation

**References**

1. Baron EP. Comprehensive review of medicinal marijuana, cannabinoids, and therapeutic implications in medicine and headache: what a long strange trip it's been …. Headache 2015; 55 (6): 885-916.

2. Boychuk DG, Goddard G, Mauro G, Orellana MF. The effectiveness of cannabinoids in the management of chronic nonmalignant neuropathic pain: a systematic review. J Oral Facial Pain Headache 2015; 29 (1): 7-14.

3. Whiting PF, Wolff RF, Deshpande S, et al. Cannabinoids for medical use: a systematic review and meta-analysis. JAMA 2015; 357: 2456-2473.

4. Headache Classification Committee of the International Headache Society (IHS). The International Classification of Headache Disorders, 3rd edition (beta version). Cephalalgia 2013; 33 (9): 629-808.

5. Zhang Y, Huo M, Zhou J, Xie S. PKSolver: An add-in program for pharmacokinetic and pharmacodynamic data analysis in Microsoft Excel. Comput Methods Programs Biomed 2010; 99 (3): 306-314.

### P219 Time-related changes of clinical symptoms induced by Onabotulintoxin A (BONTA) in chronic migraine: data from an innovative smartphone application

#### Antonio Santoro^1^, Marianna Delussi^2^, Eleonora Vecchio, Olimpia Difruscolo^3^, Laura De Rocco^4^, Maurizio Leone^1^, Marina de Tommaso^2^

##### ^1^Neurology Division, IRCSS Ospedale Sollievo dalla Sofferenza, San Giovanni Rotondo, Italy;^2^Applied Neurophysiology and Pain Unit, SMBNOS Department, Bari Aldo Moro University (Italy);^3^Division of Neurology, Di Venere Hospital, Bari, Italy;^4^IRIS s.n.c. - Consorzio Stabile Terin- Parco Tecnologico Cittadella Della Ricerca - Mesagne - 72100 Brindisi

###### **Correspondence:**Marina de Tommaso (marina.detommaso@uniba.it)

**Background** Onabotulintoxin A (BONTA) is a treatment recommended for chronic migraine CM). Its efficacy in reducing migraine frequency and disability was fully demonstrated (1), though its effect on other invalidating features as allodynia, vegetative symptoms and pain topographical distribution is still unclear. Recent preliminary studies suggested the utility of an easy-to-use smartphone-based electronic pain diary (IHCS AID Diary) which enables assessment of clinical features of pain over time. (2) This study aimed to monitor the effect of single doses of BONTA in CM patients, by the used of the electronic diary ( )-

**Materials and methods:** Twelve CM patients were selected at three different Apulian Headache Centers. Each patient was submitted to 2 consecutive BONTA treatment sessions, according to the PREEMPT study, over a a total time of six months. The subject's task, during migraine, was to indicate the location and topographical extension of pain (on a bodymap), the intensity of pain (on a visual analogue scale - VAS), the state of discomfort (on the Wong-Baker FACES pain rating scale), vegetative symptoms and therapeutic response by using the AID Diary. All subjects also completed paper pain diaries.

**Results.** A significant and progressive reduction of migraine episodes was observed from the paper and the electronic diary, in respect to the average frequency computed in the three months preceding BONTA intake. The time- regression analysis showed no relevant change of number of allodynia symptoms and vegetative symptoms. The topographical extension of headache and the state of discomfort, went into progressive reduction during the six months of observation, being mutually correlated (Fig. 1).

**Conclusions**

The use of an innovative electronic diary, suggested a positive action of BONTA on the topographical extension of headache, with a consequent subjective clinical improvement. This peculiar effect could confirm that the clinical efficacy of BONTA is mediated by its action on the mechanism of central sensitization at trigeminal level.

**Fig. 1 (abstract P225).**
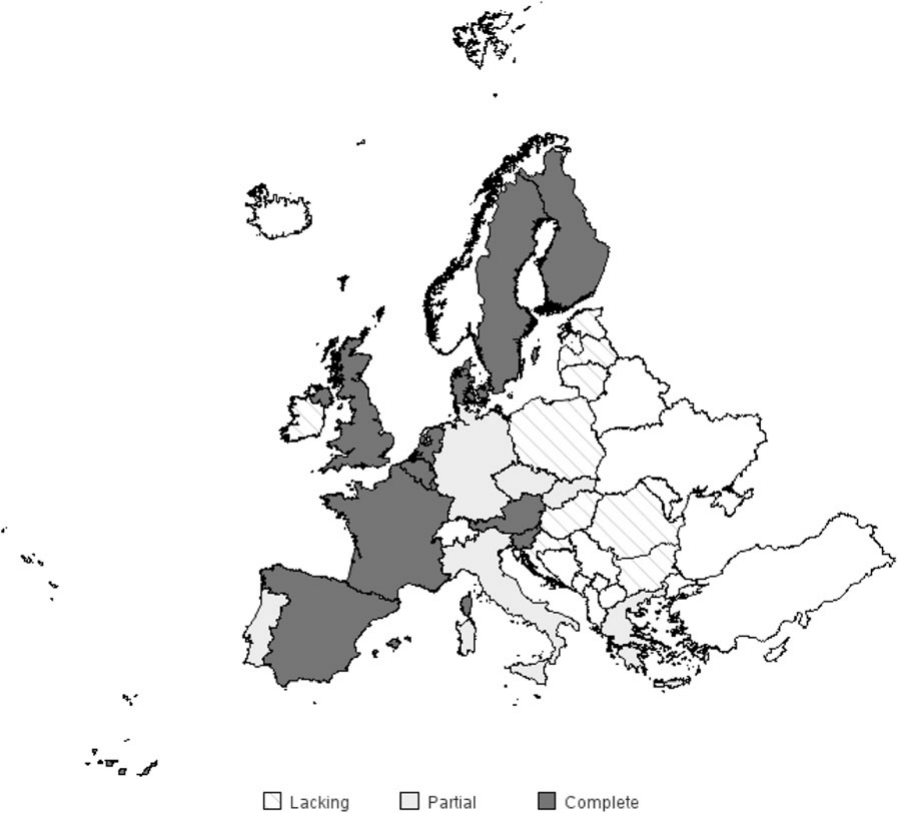
Availability of CH symptomatic effective treatments in the EU countries

**References**

1) Dodick, D.W.; Turkel, C.C.; DeGryse, R.; Aurora, S.K.; Silberstein, S.D.; Lipton, R.B.; Diener, H.C.; Brin, M.F.; PREEMPT Chronic Migraine Study Group. OnabotulinumtoxinA for treatment of chronic migraine: Pooled results from the double-blind, randomized, placebo-controlled phases of the PREEMPT clinical program. Headache 2010, 50, 921–936.

2) Franco G, Delussi M, Sciruicchio V, Marani W, De Rocco L, de Tommaso M. P011. The use of electronic pain diaries via telemedicine for managing chronic pain. J Headache Pain. 2015 Dec;16(Suppl 1):A190.

### P220 Clinical evaluation in two sub-populations of cluster headache patients with neprilysin (NEP) and pituitary-adenyl cyclase activating polipeptide (PACAP) receptor gene variants

#### Maria Michela Cainazzo^1^, Elena Bacchelli^2^, Carlo Baraldi^1^, Simona Guerzoni^1^, Lanfranco Pellesi^1^, Michele Zoli^3^, Elena Maestrini^2^, Luigi Alberto Pini^1^

##### ^1^Headache and Drug Abuse Unit, Policlinico Hospital, University of Modena and Reggio Emilia, Via del Pozzo 71, 41100 Modena, Italy;^2^Department of Pharmacy and Biotechnology, University of Bologna, Via Selmi 3, 40126 Bologna, Italy;^3^Department of Biomedical, Metabolic and Neural Sciences, Center for Neuroscience and Neurotechnology, University of Modena and Reggio Emilia, Via G. Campi 242, 41100 Modena, Italy

###### **Correspondence:**Maria Michela Cainazzo (cainazzo.michela@policlinico.mo.it); Luigi Alberto Pini (luigialberto.pini@unimore.it)

**Background**

Cluster Headache (CH) is a severe primary headache that affect around 0.1% of general population. Twin and family studies provided the importance of genetic factors in CH [1]; recently, our study suggested an association between CH with both a common variant of the PACAP (pituitary adenyl-cyclase activated peptide) receptor gene (ADCYAP1R1) and a rare potentially damaging missense variant in the MME gene, encoding for the membrane metallo-endopeptidase neprilysin [2]. The aim of this study is to evaluate the clinical characteristic of those patients with mutations and to compare them to unmutated CH suffers.

**Materials and methods**

One hundred patients with CH were enrolled at the Headache and Drug Abuse Centre, University of Modena and Reggio Emilia (Italy). CH diagnosis was made following the ICHD-III beta criteria. All participants provided a written informed consent to partecipate; the study was approved by the local Ethical Committee. For each patient a blood sample was collected and analyzed through a genome-wide association study (GWAS) using the Infinium PsychArray (Illumina) which combines common highly-informative genome-wide tag SNPs and exonic SNPs [3]. Furthermore, patients reported in a specific format, the clinical characteristics of headache, the vital parameters, the results of psychological tests and their smoke habits.

**Results**

The data collected suggest a higher age for the NEP unmutated group compared with the mutated one [58.64±17.34 vs 46.96±12.56; p<0.001]. Moreover, the statistical analysis point out that there is a tendency to have a higher age of CH onset in the NEP-mutated patients (n=7) than in the unmutated one (n=93) [36±14.1 vs 30±12.53; p=0.053]; this trend is also evident in PACAP-R mutated group (n=36) than in unmutated ones (n=64) [33.31±13.61 vs 28.56±11.63; p: 0.049]. Furthermore, a higher number of smoked cigarettes was registered for the PACAP-R unmutated group, both at the beginning of the habit [19±10.56 vs 14.22±10.59; p=0.016] and during CH active phases [10.11±10.9 vs 14 ± 9.44; p=0.047].

**Conclusions**:

ADCYAP1R1 and MME genes are involved in pain processing; and, their gene variants seems to have a role in CH susceptibility [4,5]. Also our data suggest an involvement and a potential protective effect of these gene variants, in fact, the patients with PACAP-R unmutated smoke a higher number of cigarettes (it is well known that smoke and CH are closely related); both PACAP and NEP mutated group show a higher age of CH onset. However, further and larger investigation are needed to confirm these hypothesis.

**References**

1. Russell MB. Epidemiology and genetics of cluster headache. Lancet Neurol. 2004; 3:279–283

2. Bacchelli E, Cainazzo MM, Cameli C, Guerzoni S, Martinelli A, Zoli M. Maestrini E., PiniLA. A genome-wide analysis in cluster headache points to neprilysin and PACAP receptor gene variants. J Headache and Pain. 2016; 17: 114

3. Infinium PsychArray-24 Kit | Psychiatric predisposition microarray .2016 http://www.illumina.com/products/psycharray.html. Accessed 30 Sept 2016.

4. Tuka B, Szabo N, Toth E et al. Release of PACAP-38 in episodic cluster headache patients - an exploratory study. J Headache Pain. 2016; 17:69

5. Auer-Grumbach M, Toegel S, Schabhuttl M et al. Rare variants in MME, encoding metalloprotease neprilysin, are linked to late-onset autosomal-dominant axonal polyneuropathies. Am J Hum Genet. 2016; 99:607–623

### P221 Medication-overuse headache management: data from SAMOHA study

#### Angela Verzina^1^, Ilenia Corbelli^1^, Paolo Eusebi^1^, Letizia Maria Cupini^2^, Stefano Caproni^3^, Paola Sarchielli^1^and Paolo Calabresi^1^

##### ^1^Neurological Clinic, Department of Medicine, University of Perugia, Italy;^2^Headache Center and Cerebrovascular Disease, S. Eugenio Hospital, Roma, Italy;^3^Neurological Clinic, Department of Neuroscience, University of Terni, Italy

###### **Correspondence:**Angela Verzina (verzinaangela@hotmail.it)

**Background and aim of the study**

Medication Overuse Headache (MOH) is a chronic disorder with a prevalence of 1-2%, and a peak of 5% in women aged 40-50 years [1].There is no established consensus concerning MOH standard of care, but advice and educational intervention has proved to be effective likewise structured inpatient/outpatient detoxification programmes in reducing medication overuse in uncomplicated MOH [2].This multicentre study aimed to assess any differences in phenotypical characteristics, type and amount of drugs overused and comorbidities between MOH patients who respond to an advice and educational intervention and who did not.

**Materials and Methods**

Our group previously demonstrated the efficacy and tolerability of sodium valproate (VPA) in a 12-week treatment period of MOH patients after detoxification, within the multicenter placebo-controlled SAMOHA study [3].At V1 demographic and clinical data of the patients were collected.Then, they filled out a daily headache diary for an observational period of 4 weeks.At V2, therefore, all patients were divided in two subgroups:the one who could continue the study (randomized group-R group) and the drop-out one (non-randomized group-NR group).

**Results**

One-hundred and thirty patients were screened nine participating centers [104 (80%) women;mean age was 42 years old].At V2, 88 (67.7%) patients continued meeting the inclusion/exclusion criteria and were, then, randomized to placebo/VPA (R group), while 42 patients drop-out the study(NR-group).Analyzing in detail the drop-out reasons,we find that 34 of the 42 NR patients left the study at V2 because they were no more satisfying the inclusion/exclusion criteria(screening failure group-SF group). The remaining 8 patients left the study before reaching V2 for other reasons.Comparing the clinical and demographic differences between the R and SF groups,R group was significantly older and with more years of migraine in history than SF group.Moreover, in R group, the headache has become chronic for more than twice of years, compared to SF group.

**Conclusions and Discussion**

The first conclusion is that in every MOH trial, after an educational session (simple advice) on this disease, an observational period must precede the assignment to any type of treatment, in order to confirm the diagnosis of MOH.Obtaining a sample representative of “pure” MOH will certainly improve the reliability of the results.On the other hand, in the clinical field, our findings demonstrate that early diagnosis (young age and/or few years of episodic and chronic migraine in history) of MOH is needed to ensure a high remission rate. Necessarily, early diagnosis must start from the general practitioner and before that by the pharmacist.

**References**

1. Westergaard ML, Hansen EH, Glümer C, Olesen J, Jensen RH. Definitions of medication-overuse headache in population-based studies and their implications on prevalence estimates: a systematic review. Cephalalgia. 2014 May;34(6):409-25. doi: 10.1177/0333102413512033. Epub 2013 Nov 29.

2. Rossi P, Faroni JV, Nappi G. Short-term effectiveness of simple advice as a withdrawal strategy in simple and complicated medication overuse headache. Eur J Neurol. 2011;18:396-401.

3. Sarchielli P, Messina P, Cupini ML, et al. Sodium Valproate in Medication Overuse Headache Treatment: a placebo-controlled randomized trial. Eur Neuropsychopharmacol. 2014; 24: 1289–1297.

### P222 Orthostatic headache from spontaneous intracranial hypotension (SIH) complicated with convexity subaracnoid haemorragy (SAH) in patient with lung cancer

#### Francesca Marsili^1^, Antonio Matera^1^, Luca Onofrio Scappatura^1^, Umberto Giulio Sica^1^, Monica Pace^1^, Valeria Coppola^2^, Enrico Ferrante^1^

##### ^1^Neurology;^2^Neuroradiology Department, AOR San Carlo, Potenza, Italy

###### **Correspondence:**Enrico Ferrante (enricoferrante@libero.it)

**Background**

Non traumatic convexity SAH may rarely complicate SIH and can be attributed to stretching/rupture of a cortical bridging vein. We describe one patient with lung cancer who had orthostatic headache secondary to SIH complicated with convexity SAH

**Case report**

A 64 years old male undergone to left pneumectomy for lung cancer one year before, presented with one month history of severe orthostatic headache following a strong coughing. Headache kept on worsening in following days changing from orthostatic to non positional and persistent one. Brain CT showed left frontal anterior SAH and bilateral fronto-parietal hygromas. Brain MRI revealed diffuse pachymeningeal enhancement typical sign of SIH and confirmed the SAH. One month after symptoms beginning cerebral angiography revealed no aneurisms or arterio-venous malformations and normal visualization of cortical veins. Spinal MRI showed no CSF leak site. We treated this patient with conservative measures: Trendelenburg bed rest for 3 weeks, overhydration and analgesic therapy. Within 3 weeks his headache progressively disappeared and brain CT was normal

**Conclusions**

Non traumatic convexity SAH is uncommon and may be associated with isolated cortical veins thrombosis, which in turn, may rarely complicate SIH [1]. Reduced intracranial pressure and brain descent can result in dilatation and distortion of cerebral veins and finally rupture of cortical veins leading to convexity SAH in SIH, as in our case. Our case underlines the importance of differential diagnosis between SIH pachymeningitis and meningeal carcinomatosis. Brain MRI showed thickening of dura mater with diffuse symmetric and linear pachymeningeal enhancement, typical and specific sign of SIH [2], which is different from meningeal carcinomatosis conversely characterized by focal and or nodular pachymeningeal enhancement but also by leptomeningeal enhancement, which is absent in SIH

**Consent for publication:** The authors declare that written informed consent was obtained for publication.

**References**

1. Ferrante E, Citterio A, Valvassori L, Arpino I, Tiraboschi P. A case of convexity subaracnoid haemorrhage treated with epidural blood patch. Neurogical Sciences. 2012; 33:715-716

2. Ferrante E, Arpino I, Citterio A, Wetzl R, Savino A. Epidural blood patch in Trendelenburg position pre-medicated with acetazolamide to treat spontaneous intracranial hypotension. Eur J Neurol 2010; 17:715-719

### P223 Awareness of migraine in Neo-Latin countries: a study in 12 headache centers over 7 countries

#### Michele Viana^1^, Farihah Khaliq^1^, Grazia Sances^1^, María De Lourdes Figuerola^2,3^, Vittorio Di Piero^4^, Pierangelo Geppetti^5^, Rosario Iannacchero^6^, Ferdinando Maggioni^7^, Mauro Eduardo Jurno^8^, Ecaterina Chiriac^9^, Alejandro Marfil^10^, Filippo Brighina^11^, Nelson Barrientos Uribe^12^, Cristina Pérez Lago^13^, Carlos Bordini^14^, Franco Lucchese^4^, Valerio Maffey^4^, Giuseppe Nappi^1^, Giorgio Sandrini^1,15^, Cristina Tassorelli^1,15^

##### ^1^Headache Science Center, C. Mondino National Neurological Institute, Pavia, Italy;^2^Hospital de Clínicas José San Martín;^3^Hospital Alemán, Buenos Aires, Argentina,^4^Sapienza University, Rome,^5^University of Florence, Florence,^6^A.O. “ Pugliese – Ciaccio“, Catanzaro,^7^Padua University, Padua, Italy,^8^FAME/FUNJOB and FHEMIG, Barbacena, Brazil,^9^National Headache Center - Republic of Moldova, Chisinau city, Moldova, Republic of,^10^Hospital Universitario, Monterrey, Mexico,^11^Policlinico Universitario, Palermo, Italy,^12^Hospital DIPRECA, Santiago, Chile,^13^Hospital Maciel, Montevideo, Uruguay,^14^Clínica Neurológica Batatais, Batatais, Brazil,^15^University of Pavia, Pavia, Italy

###### **Correspondence:**Michele Viana

**Objectives**

To assess the awareness of migraine (M) and previous diagnostic and therapeutic paths in naïf migraineurs visited by headache specialists in several neo-Latin countries.

**Methods**

This is a multicentre study was conducted in parallel in 12 headache centers located over 7 neo-Latin speaking countries and coordinated by Mondino Institute, Pavia, Italy. Each center recruited up to 100 consecutive M patients aged 18 to 75 years who had been referred for a first visit. Patients answered questions about the type of headache they thought to suffer from, previous diagnosis received and previous visits/investigations/treatments for M.

**Results**

1161 patients were enrolled. 326 patients (28%) knew that they suffered from M, while 72% patients did not. 64% of patients simply called their M “headache”. Other common names were cervical pain (4%, mostly in Italy), tension-type headache (3%, mostly in Mexico, Chile and Uruguay), sinusitis (1%). After multivariate analysis factors associated with the awareness of M were 6 (Table 1).

Only variables which reached a statistical significance (p<0.05) after multivariate analysis were reported.

Mexico had the highest rate of M awareness (51%) followed by Chile (39%), Argentina (34%), Brazil (30%), Italy (25%), Moldova (17%) and Uruguay (12%). All our patients had previously visited by a GP for M, but only 8% of them diagnosed it as M. The majority of patients (80%) has been visited by at least one specialist for their M, but only 35% of them formulated the correct diagnosis.

High rates of M diagnosis were observed in Moldova (53%), Argentina (68%) and Uruguay (52%), but a minority of patients in these countries was aware to suffer from M: 17%, 34% and 11% respectively. 50% of patients were prescribed a X-ray and/or CT and/or MRI of the cervical spine. 76% of patients underwent to imaging of brain and/or cervical spine that exposed them to radiation. 28% of patients had previously received a symptomatic migraine specific medication and 29% had received at least one M preventative medication.

**Conclusion**

Although M is the 3rd most common pathology worldwide and the 7th for disability, there is poor awareness of it among patients even after consultation with physician(s). These findings speak in favour of the importance of educating doctors and patients in the field of M in order to reduce its burden worldwide.

**Table 1 (abstract P223).** Association between sociodemographical and clinical factors and awarness of migraine

	Sign	OR (95% CI)
High educational level	<0.001	1.97 (1.43-2.78)
Number of family members with migraine	0.005	1.17 (1.04-1.31)
Duration of attacks (hours)	0.001	0.98 (0.97-0.99)
Throbbing pain	0.005	2.02 (1.23-3.31)
Localization of pain: lateral	0.043	1.36 (1.01-1.83)
Vomiting	0.018	1.43 (1.06-1.93)

**Aknowledgment**

This work was developed by the Italian Linguistic Group of IHS and supported by Mondino Institute (grant of the Italian Ministry of Health RC 2013-2015).

**Disclosure of Interest**

None Declared.

### P224 Guideline adherence reduces neuroimaging utilization in Headache Center

#### Alessandro Panconesi (alessandro.panconesi@uslcentro.toscana.it)

##### Headache Center, Department of Neurology, Health Authority 11, Empoli, Italy

**Background**

Headache neuroimaging (NI) is commonly ordered even in absence of red flags and despite guideline recommendations. Enhancing the continuity of care was suggested as one potential way to reduce unnecessary NI [1]. The aim of the study was to evaluate if a different headache care organization and strict observation of guidelines may optimize headache NI practices.

**Materials and methods**

Integration between a general practitioner (GP), specialized in internal medicine, particularly expert in the headache management due to previous long lasting experience in a Headache Center (HC), and neurologists in the conduction of a HC was experimented for the first time in Italy in the Health Authority 11 of Empoli, covering about 240000 residents. The aim was to optimize the headache care through a strict collaboration between GPs and HC for better continuity of care. Only non-acute referrals are seen by HC specialists and the waiting time for appointment was less than 3 months. Headache specialists had access to health information of previous specialist visits, hospital recovery or emergency department access, contained in the electronic records of Health Authority. The aim of this study was to evaluate the NI investigation rate in patients referred for the first time in 2011-2013 years to HC. To reduce the unnecessary and overused NI, the GP, basing on clinical history and first level neurological examination, could only order magnetic resonance imaging (MRI) or computed tomography (CT) in presence of red flags considered in the principal guidelines, which were specified in the consultation record. NI for reassurance or patient request was not ordered. The rate of NI request was compared to that of the two consultant neurologists of HC (χ^2^ test).

**Results**

There were large differences in the proportion of headache patients imaged by the consultants. Neurologist visits were associated with increased NI (26.3% vs. 10.9%, p <0.001) (Table 1). In patients visited by GP, considering only the 676 (62%) subjects whom had never undergone NI, the percentage was 14.6.

**Conclusions**

This study highlights that, following guideline recommendations, the rate of NI utilization was largely inferior to that of neurologist consultation in HC, and also to that previously reported in various clinical settings [2]. In this study the correlation of reasons for investigation with neuroradiological findings shows significant abnormalities possibly related to headache in only 2.2% [2], in agreement with the literature data. Therefore, optimizing headache NI practices should be a major priority. It would be necessary a more exact definition of which changes in headache pattern, and their onset interval, require investigation.

**Table 1 (abstract P224).** See text for description

	Patients (n)	Mean age ± SD (range)	♀	♂	RMI/CT (n)	Imaged (%)
Neurologists	520	41.6 ± 18 (4-91)	75.2%	24.8%	127/10	26.3
GP	1078	41.0 ± 15 (4-90)	74.4%	25.6%	107/11	10.9

**References**

1) Callaghan BC, Kerber KA, Pace RJ, Skolarus L, Cooper W, Burke JF. Headache neuroimaging: Routine testing when guidelines recommend against them. Cephalalgia 2015,35:1144-52.

2) Panconesi A, Bartolozzi ML, Guidi L, Santini S, Mennuti N, Carini V. P023.Reasons for headache investigation and findings in an experimental headache center. J Headache Pain 2015,16(Suppl 1):A189.

### P225 Availability of effective evidence-based symptomatic treatments for cluster headache in the EU countries: A survey of the European Headache Alliance and European Headache Federation

#### Paolo Rossi^a,b^, Elena Ruiz De La Torre^a^, Dimos Mitsikostas^c^, Aurore Palmaro^d,e,f*^

##### ^a^European Headache Alliance;^b^Headache Clinic INI Grottaferrata, Italy;^c^Aeginition Hospital, National & Kapodistrian University of Athens, Athens, Greece;^d^Medical and Clinical Pharmacology department, Toulouse University Hospital, Toulouse, France;^e^UMR INSERM 1027, University of Toulouse, Toulouse, France;^f^CIC 1436, Toulouse University Hospital, Toulouse, France

###### **Correspondence:**Paolo Rossi

**Background**: Treating cluster headache can be tricky because the pain becomes extremely severe very quickly and only few evidence based treatments can work. Recent data from IHS suggest that oxygen is not universally reimbursed or available for CH patients. The aim of this study was to assess the reimbursement option and accessibility of 3 effective medicines for CH (sumatriptan s.c, oxygen zolmitriptan spray) across EU

**Materials and Methods**: A brief survey investigating the availability of symptomatic treatments for CH was send on e-mail on January 2017 to at least one headache specialist for every single country of the EU. For a complimentary point of view. In the countries where active CH patients’ associations exist the survey was completed by CH expert patients.

**Results**: The questionnaire was completed by 26 headache specialists (93% of the EU countries representing 99.75% of the European population) and 10 CH expert patients (representing 72% of the European population). The answers provided by the headache specialists and expert patients were coherent in every country. Availability of ETs was defined as: a) complete: both oxygen and sumatriptan s.c fully reimbursable and accessible; b) restricted: partial reimbursment or inaccessibility of one between Oxy and Suma s.c; c) lacking: both oxygen and sumatriptan s.c not reimbursable and not accessible Oxygen was reimbursable for 62.68% of the CH population. Oxygen device was reimbursable for 49% of the CH population. Sumatriptan s.c. was reimbursable for 66% and accessible without restrictions for 45% of the CH population. Zolmitriptan spray was reimbursable for 23.7% and accessible without restrictions for 30.9% of the CH population. Availability of CH effective treatments resulted complete, restricted or lacking for 47%, 35.2% and 18% respectively of the CH European patients (Fig. 1)

**Conclusion**: Based on this survey only 47% of the EU population had an unrestricted access to CH effective treatments with unacceptable inequalities between eastern countries and the rest of Europe. Headache societies and patients’associations should pressure European and national health authorities to improve the availability of effective symptomatic treatments for CH

**Consent for publication:** The authors declare that written informed consent was obtained for publication.

**Fig. 1 (abstract O4).**
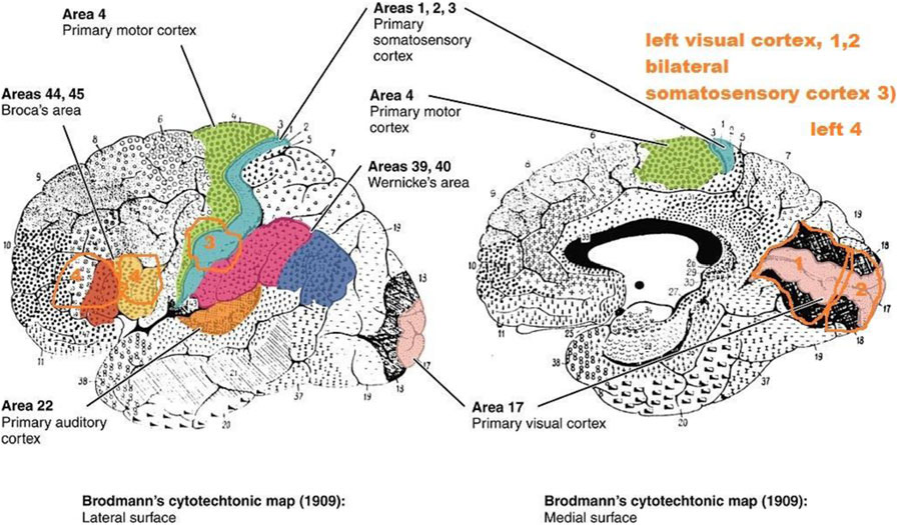
Simulation of CSD progression in a single patient with reported scotomas in the right visual hemifield, followed by bilateral brachial paresthesia and speech disturbances. The time of progressive cortical areas involvement in the simulation model, was in accord with the reported clinical evolution of aura symptoms.

### P226 The evaluation of short-latency afferent inhibition discloses abnormal fluctuations of cholinergic transmission during the migraine cycle

#### Davide Di Lenola^1^, Gianluca Coppola^2^, Francesca Cortese^2^, Cherubino Di Lorenzo^3^, Francesco Pierelli^3^

##### ^1^Sapienza University of Rome Polo Pontino, Department of medico-surgical sciences and biotechnologies, Latina, Italy;^2^G.B. Bietti Foundation IRCCS, Department of Neurophysiology of Vision and Neurophthalmology, Rome, Italy;^3^Don Gnocchi foundation-IRCCS, Milan, Italy

###### **Correspondence:**Davide Di Lenola

**Background**

Short-latency afferent inhibition (SAI) is a form of inhibition related to the cholinergic activity in the cerebral cortex. It consists in a galvanic stimulation of the median nerve at the wrist that can suppress motor cortex excitability, as tested by transcranial magnetic stimulation (TMS), when given at a short interstimulus interval (ISI) between 18 and 21 ms. SAI is considered an in-vivo way to study the sensorimotor integration mechanisms. SAI response is influenced by the excitatory effect of acetylcholinergic thalamocortical afferents on the inhibitory GABAergic (mostly GABAa) cortical networks.

**Materials and methods**

We recruited 30 migraine without aura patients (16 between [MO] and 14 during [MI] attacks), and we compared them to a group of 16 healthy volunteers (HV). We first recorded somatosensory evoked potentials N20 latency and N20-P25 peak-to-peak amplitude at the contralateral parietal area. Afterward, SAI was recorded in all study’s participants as follows: after a conditioning single pulse delivered on the median nerve at the wrist, a TMS pulse was delivered with ISIs derived from the latency of N20 plus 2 to 8 ms in steps of 2ms and in random order. Five stimuli were delivered at each ISI. We calculated the SAI slope of the linear regression between the unconditioned motor evoked potential (MEP) amplitude and the 4-conditioned MEPs as a measure of cortical excitability.

**Results**

Compared with HV, SAI was significantly reduced in MO, but enhanced in MI patients (slope HV= +11.2, MO= +242, MI= -129). In both HV and MO groups, but not in MI, the SAI slope positively correlated with the SSEP N20-P25.

**Conclusions**

The reduction of SAI in MO patients and its enhancement in MI patients suggests a decrease and an increase respectively in facilitatory thalamocortical cholinergic activity on GABAergic network activity in the motor cortex. Since from the correlation analysis emerges that slope of SAI normally correlates with the parietal response in MO, but not in MI, we argue that more dysfunctional sensorimotor integrative mechanisms might characterize migraineurs during an attack.

### P227 The clinical efficacy of short-lasting ketogenic diet in migraine is due to a general normalization of the interictal cortical hyperresponsivity rather than to a direct modulation of the subcortical brainstem activity

#### Cherubino Di Lorenzo^1^, Gianluca Coppola^2^, Martina Bracaglia^3^, Ilaria Bove^3^, Davide Di Lenola^3^, Mariano Serrao^3^, Vincenzo Parisi^2^, Francesco Pierelli

##### ^1^Don Carlo Gnocchi Onlus Foundation, Milan, Italy;^2^G. B. Bietti Foundation-IRCCS, Rome, Italy;^3^“Sapienza” University of Rome Polo Pontino, Latina, Italy;^4^INM Neuromed IRCCS, Pozzilli (IS), Italy

**Background -** We previously reported that a short-lasting period of ketogenic diet (KD) regimen can help to prevent migraine and can normalize its interictal abnormal cortical hyperresponsivity. Here, we aimed to verify whether cerebral cortex is the primary site of KD-related changes or if the latter are the expression of ketones ability to modulate brainstem subcortical structures.

**Methods -** We simultaneously recorded the nociceptive specific blink reflex (nBR, a marker of the brainstem trigeminal activity) and cortical pain-related evoked potentials (PREP) elicited by the stimulation of the right supraorbital division of the trigeminal nerve in 18 migraine without aura patients before and after 1-month of KD, during ketogenesis. We measured nBR R2 component area-under-the-curve as well as PREP amplitude habituations as the slope of the linear regression between the 1^st^ and the 2nd block of 5 averaged responses.

**Results -** We confirmed the ability of 1-month KD of significantly decreasing mean attack frequency and duration. KD significantly induced normalization of the interictally reduced PREP habituation (pre: +1.8, post: -9.1), while nBR habituation remained unchanged.

**Conclusion -** The results of the present study suggest that the clinical efficacy of a short-lasting KD regimen in migraine can be primarily due to a general normalization of the interictal cortical dysfunction, and not to a direct modulation of the subcortical brainstem activation.

### P228 Cerebral grey matter density is reduced in chronic migraine patients: correlations with clinical features

#### Gianluca Coppola^1^, Barbara Petolicchio^2^, Antonio Di Renzo^1^, Emanuele Tinelli^2^, Cherubino Di Lorenzo^3^, Vincenzo Parisi^1^, Mariano Serrao^4^, Valentina Calistri^2^, Stefano Tardioli^2^, Gaia Cartocci^2^, Francesca Caramia^2^, Vittorio Di Piero^2^, Francesco Pierelli^4,5^

##### ^1^G.B. Bietti Foundation IRCCS, Research Unit of Neurophysiology of Vision and Neurophthalmology, Rome, Italy;^2^“Sapienza” University of Rome, Department of Neurology and Psychiatry, Rome, Italy;^3^Don Carlo Gnocchi Onlus Foundation, Milan, Italy;^4^“Sapienza” University of Rome Polo Pontino, Department of Medico-Surgical Sciences and Biotechnologies, Latina, Italy;^5^IRCCS-Neuromed, Pozzilli (IS), Italy

###### **Correspondence:** Gianluca Coppola

**Background**

Few neuroimaging studies have explored brain morphometry in patients affected by chronic migraine (CM). Most of these studies were performed in patients with medication overuse. Here, we performed voxel-based morphometry (VBM) analysis to investigate the grey matter (GM) density of the whole brain in patients affected by CM without medication overuse. Our aim was to investigate whether there are fluctuations in the GM densities in relation to the patients’ clinical features.

**Materials and methods**

Twenty untreated CM patients without a past medical history of medication overuse underwent 3T MRI scans and were compared to a group of 20 healthy volunteers (HV). SPM12 and CAT12 toolbox were used to process MRI data and to perform VBM analysis of structural T1-weighted MRI scans. The patients’ versus HV relative GM density was assessed with an uncorrected threshold of p < 0.005. To check for possible correlations, patients’ clinical features and GM maps were regressed.

**Results**

Compared to HV, CM patients presented 4 clusters of significantly lower GM densities: I) right cerebellar lobules (VIIIa, CrusII), II) the left occipital areas (BA17/BA18), III) the left middle temporal gyrus, and IV) the left temporal pole /amygdala /pallidum /orbitofrontal cortex. The GM density of cerebellar hemispheres correlated negatively with the years of headache disease, and positively with the number of tablets intake per month.

**Conclusions**

To summarize, CM is associated with lower GM density in several brain areas known to be involved in antinociception, multisensory integration, and analgesic dependence. The GM density within the cerebellum was significantly related to longer duration of headache disease and to higher consumption of acute headache medications. We hypothesize that this morphological pattern we identified may be considered as a predisposing ground on which to develop medication overuse headache.

## EHF ORAL PRESENTATIONS

### O1 Efficacy of erenumab in subjects with episodic migraine with prior preventive treatment failure(s)

#### Koen Paemeleire^1^, Gregor Broessner^2^, Jan Brandes^3^, Jan Klatt^4^, Feng Zhang^5^, Hernan Picard^5^, Daniel D Mikol^5^, Robert A Lenz^5^

##### ^1^Ghent University Hospital, Ghent, Belgium;^2^Medical University of Innsbruck, Innsbruck, Austria;^3^Nashville Neuroscience Group and Vanderbilt University School of Neurology, Nashville, TN, USA;^4^Novartis, Basel, Switzerland;^5^Amgen Inc., Thousand Oaks, CA, USA

###### **Correspondence:** Koen Paemeleire (Koen.Paemeleire@uzgent.be)

**Background**

There is a high unmet need for new preventive migraine treatments, especially for patients who have failed existing migraine therapies. Erenumab is a fully human monoclonal antibody that blocks the calcitonin gene-related peptide receptor. In a large, multicenter, double-blind, placebo controlled, phase 3 study (STRIVE), erenumab 70 mg and 140 mg demonstrated efficacy in subjects with episodic migraine and showed a safety profile similar to placebo. Here we report efficacy results in a subgroup of trial subjects with prior preventive treatment failure(s).

**Methods**

Subgroup analyses were conducted in subjects from the STRIVE trial who had failed ≥1 (n=369) or ≥2 (n=161) prior preventive treatments due to lack of efficacy and/or intolerability. Analyses included change from baseline in mean monthly migraine days (MMDs) and achievement of ≥50% reduction from baseline in MMDs, assessed over weeks 13–24 (months 4, 5, and 6). In the full trial, subjects (N=955) were randomized 1:1:1 to subcutaneous monthly placebo or erenumab 70 mg or 140 mg for 24 weeks (6 months). *P* values for subgroup analyses are descriptive and not adjusted for multiple comparisons.

**Results**

Greater reductions from baseline in MMDs were observed for the erenumab 70 mg and 140 mg groups compared with placebo in both treatment failure subgroups (Table 1). More subjects who received erenumab achieved ≥50% reduction in MMD in both subgroups compared with placebo. For the 70 mg group, the odds (95% confidence interval) of achieving ≥50% reduction in MMD were 2.9 times higher than that of placebo for both treatment failure subgroups. For the 140 mg group, the odds were 3.1 and 4.5 times higher than placebo, respectively.

**Conclusion**

Robust treatment effects were observed for both 70 mg and 140 mg erenumab in subjects who had previously failed preventive migraine treatments. For 140 mg, effects were numerically greater in this subpopulation than in the overall trial population, and as in the overall population, erenumab 140 mg showed numerically greater efficacy than erenumab 70 mg. These results suggest that erenumab may have particular utility in this subgroup of patients.

### O2 GLP-1 Reduces Cerebrospinal Fluid Secretion And Intracranial Pressure: A Novel Treatment For Idiopathic Intracranial Hypertension?

#### Hannah Botfield^1,2^, Maria Uldall^3^, Connar Westgate^1,2^, James Mitchell^1,4^, Snorre Hagen^3^, Ana Maria Gonzalez^5^, David Hodson^1,6^, Rigmor Jensen^3^, Alexandra Sinclair^1,4^

##### ^1^Institute of Metabolism and Systems Research, University of Birmingham, Edgbaston;^2^Centre for Endocrinology, Diabetes and Metabolism, Birmingham Health Partners, Birmingham, United Kingdom;^3^Danish Headache Center, Clinic of Neurology, Rigshospitalet-Glostrup, University of Copenhagen , Glostrup, Denmark;^4^Department of Neurology, University Hospitals Birmingham NHS Foundation Trust, Birmingham;^5^Institute of Inflammation and Ageing, University of Birmingham, Edgbaston;^6^Centre of Membrane Proteins and Receptors (COMPARE), University of Birmingham, Edgbaston, United Kingdom

###### **Correspondence:** Alexandra Sinclair (a.b.sinclair@bham.ac.uk)

**Background**

Current therapies for reducing raised intracranial pressure (ICP) in conditions such as idiopathic intracranial hypertension have limited efficacy and tolerability. As such, there is a pressing need to identify novel drugs. Glucagon-like peptide-1 receptor (GLP-1R) agonists are used to treat diabetes and promote weight loss but have also been shown to affect fluid homeostasis in the kidney. Here, we investigate whether exendin-4, a GLP-1R agonist, is able to modulate cerebrospinal fluid (CSF) secretion at the choroid plexus and subsequently reduce ICP.

**Methods**

GLP-1R mRNA and protein was assessed by quantitative PCR, immunohistochemistry and fluorescently tagged exendin-4 in human and rat choroid plexus. The effect of exendin-4 on GLP-1R activation and CSF secretion was evaluated in cultured rat choroid plexus epithelial cells using cAMP assays and a Na+ K+ ATPase activity assay. The effect of Exendin-4 on ICP was assessed in adult female rats with normal and raised ICP.

**Results**

We demonstrated that the GLP-1R is present in human and rat choroid plexus. Exendin-4 significantly increased cAMP levels (2.14 ± 0.61 fold, P<0.01) part of the GLP-1R signalling pathway, in a concentration-dependant manner and this response could be inhibited by the addition of the GLP-1R antagonist exendin 9-39. Exendin-4 also significantly reduced Na+ K+ ATPase activity, a marker of CSF secretion (39.3±9.4% of control; P<0.05). Finally, *in vivo* ICP recording in female adult rats demonstrated that subcutaneous administration of 20μg/kg exendin-4 significantly reduced ICP in normal (65.2 ± 6.6% of baseline; P<0.01) and raised ICP rats (56.6 ± 5.7% of baseline; P<0.0001).

**Conclusion**

We demonstrate that exendin-4 reduces CSF secretion by the choroid plexus, and ICP in normal rats and rats with raised ICP. Repurposing existing GLP-1 drugs may represent a novel therapeutic strategy for conditions of raised ICP such as idiopathic intracranial hypertension. Additionally, GLP-1R agonist therapy promotes weight loss, which would be advantageous in idiopathic intracranial hypertension.

### O3 Phase 3, randomised, double-blind, placebo-controlled study to evaluate the efficacy and safety of erenumab (AMG 334) in migraine prevention: Primary results of the STRIVE trial

#### Uwe Reuter^1*^, Jo Bonner^2^, Gregor Broessner^3^, Yngve Hallstrom^4^, Feng Zhang^5^, Sandhya Sapra^6^, Hernan Picard^7^, Daniel D Mikol^7^, Robert A Lenz^7^

##### ^1^Dept of Neurology, Charité Universitätsmedizin Berlin, Berlin, Germany;^2^Mercy Research, St Louis, MO, USA;^3^Dept of Neurology, Medical University of Innsbruck, Innsbruck, Austria;^4^Stockholm Neuro Center, Stockholm, Sweden;^5^Global Biostatistical Science, Amgen Inc., Thousand Oaks, CA, USA;^6^Global Health Economics, Amgen Inc., Thousand Oaks, CA, USA;^7^Global Development, Amgen Inc., Thousand Oaks, CA, USA

###### **Correspondence:**Uwe Reuter (uwe.reuter@charite.de)

**Background**

Efficacy and safety/tolerability of erenumab, a human anti-CGRP receptor monoclonal antibody, were evaluated in episodic migraine (EM) subjects in a multinational, phase 3 trial (NCT02456740).

**Methods**

Adults with EM (n=955) were randomised 1:1:1 to subcutaneous monthly placebo or erenumab 70 mg or 140 mg for 24 weeks. The primary endpoint was change from baseline in mean monthly migraine days (MMDs) over weeks 13-24. Secondary endpoints were ≥50% reduction in MMDs; change in acute migraine-specific medication days; change in Physical Impairment (PI) and Impact on Everyday Activities (EA) (as measured by the Migraine Physical Function Impact Diary [MPFID]). P-values are for pairwise comparisons of each erenumab dose with placebo, statistical significance determined after multiplicity adjustment.

**Results**

Subjects reported 8.3 MMDs at baseline and experienced -3.2, -3.7, and -1.8-day reductions in the 70 mg, 140 mg, and placebo groups, respectively (p<0.001). A ≥50% reduction in MMDs was achieved by 43%, 50%, and 27% in the 70 mg, 140 mg, and placebo groups (p<0.001), and monthly acute migraine- specific medication was reduced by -1.1, -1.6, and -0.2 days (p<0.001). Subjects had improved PI scores (-4.2, -4.8, -2.4 points in the 70 mg, 140 mg, and placebo groups; p<0.001) and EA scores (-5.5, -5.9, and -3.3 points; p<0.001). The safety/tolerability profile of erenumab was similar to placebo; subjects most frequently reported nasopharyngitis, upper-respiratory-tract infection, and sinusitis.

**Conclusion**

Erenumab 70 mg and 140 mg significantly reduced migraine frequency and use of migraine-specific medications, reducing migraine’s impact on physical impairment and everyday activities in this EM trial. Numerically greater efficacy was observed for the 140 mg dose consistently across endpoints.

**Trial registration**

Clinical trials.gov NCT02456740.

### O4 Modelling cortical spreading depression by a computational algorithm of distributed neural excitability: correlation with clinical features in single migraine with aura patients

#### Marina de Tommaso^1,2^, Julia Maria Kroos^3^, Eleonora Vecchio^1^, Nicola Burdi^4^, Sebastiano Stramaglia^2,5^, Luca Gerardo Giorda^3^

##### ^1^Applied Neurophysiology and Pain Unit, SMBNOS Department, Bari Aldo Moro University (Italy);^2^TIRES Center, Bari Aldo Moro University (Italy);^3^BCAM -Basque Center for Applied Mathematics-Center, Bilbao, Spain;^4^Neuroradiology Unit, SS.Annunziata General Hospital, Taranto (Italy);^5^Physic Department, Bari Aldo Moro University , Bari (Italy)

###### **Correspondence:**Marina de Tommaso (marina.detommaso@uniba.it)

**Background**

Cortical spreading depression (SD) is thought to underlie migraine aura but mechanisms of triggering SD and propagation in the structurally normal cortex of migraine patients is still unknown. Some studies showed that the sensory predominance of migraine aura is likely due to a greater susceptibility of primary sensory cortex to CSD probably for their structural complexity [1]. We used MRI imaging registration in migraine with aura patients to elaborate a CSD propagation logarithm that takes into account the morphology of the various cortical areas involved.

**Materials and methods**

We process the data sets with the Freesurfer software to obtain the geometry and labelling for the different brain regions (Desikan-Kiliani Atlas or Brodman areas 1, 2, 3, 4, 44, 45, 17, 18) in five migraine with aura patients. According to the map of the disturbed areas suggested by the aura symptoms, we then initialize the region first affected in the respective hemisphere. Starting the propagation of the CSD in this region we recorded the arrival time in each point of the brain cortex.

**Results**

We obtained the different brain geometries and the Brodman areas 1, 2, 3, 4, 44, 45, 17 and 18 of the five subjects. In order to run a simulation of the CSD propagating on the cortex we need to identify an initial region as a starting point for the wave. We take a bearing on the map of the disturbed regions provided along with the MRI data from the patients. For the simulation of the CSD we started the wave in these regions and recorded the propagation. In each point we recorded the arrival time of the wave. We visualize the arrival times of the wave in the Fig. 1. This was in accord with the reported evolution of the clinical symptoms.

**Conclusions**

The way CSD propagates can change in relation to brain morphology. The specific algorithm was able to provide for a simulation of the electrical phenomenon, explaining the progressive evolution of clinical symptoms. Further tests in larger series, could give an aid in understanding the pathophysiological peculiarities of migraine-with-aura brain.

**Fig. 1 (abstract O13).**
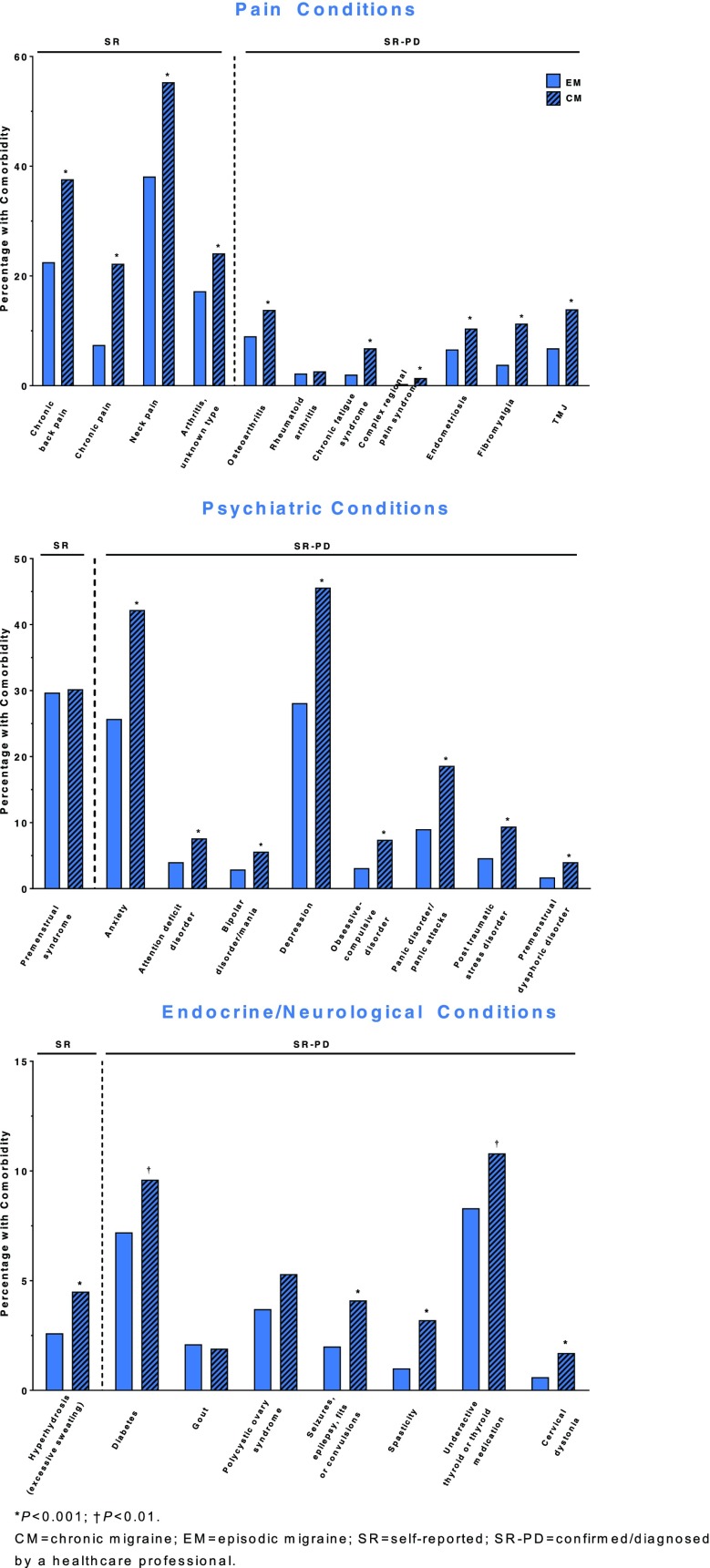
EM and CM respondents self-reporting (SR) an assessed symptom or a physician diagnosis (SR-PD) of a condition

**References**

1. Bogdanov VB, Middleton NA, Theriot JJ, Parker PD, Abdullah OM, Ju YS, Hartings JA, Brennan KC. Susceptibility of Primary Sensory Cortex to Spreading Depolarizations*. J Neurosci.* 2016 ;36(17):4733-43.

### O5 Interventional management in refractory headache disorders; Tertiary Center Based Experiences supported by application videos and a model for interactive practice of attenders

#### Aynur Özge, Derya Uludüz, Ömer Karadaş, Osman Özgür Yalın, Hayrunnisa Bolay

##### **Correspondence:**Aynur Özge (aynurozge@gmail.com)

Interventional treatment is an important but underevaluated issue for neurologist. However peripheral nerve blocks have been used for both acute and preventive treatment of headaches for decades and are safe and effective therapeutic options for many patients with headache disorders. They can cause a long lasting (several weeks to months) pain relief. This long lasting effect is thougt to be due to central pain modulation. Nerve blocks may be an option for the patients who have failed their home medications or who doesn’t want to use drugs. Blocks may also be used to treat patients who need relief between onabotulinum toxin A injections and in medication overuse headache during their acute therapy. Nerve blocks are safe and can be appropriate for children and pregnant patients, and also in patients with kidney-liver diseases. In pregnancy, lidocaine is considered a category B drug. Greater occipital nerve blockade is the most common procedure in peripheral nerve blocks. But lesser occipital nerve blockades, sphenopalatine ganglion blockades, supratrochlear, auriculotemporal, supraorbital, infraorbital and mental nerve blockades, cervical root blockades, their combinations can be used in patients with headache disorders. Local anestetics and steroids are being used for nerve blockades.

Botulinum toxin injections are also approved effective methods for migraine all over the World. There is some other indications under search and experineces increases the applications in every day.

We want to show the injection techniques and efficacy by sharing our cases. Deepending on the time, after the presentation some audience keep a chance to applicate tehmselves on the model provided by authors.

### O6 A phase 3 placebo-controlled study of galcanezumab in patients with chronic migraine: results from the 3-month double-blind treatment phase of the REGAIN study

#### Holland C. Detke, Shufang Wang, Vladimir Skljarevski, Jonna Ahl, Brian A. Millen, Sheena K. Aurora, Jyun Yan Yang

##### Eli Lilly and Company, Indianapolis, IN USA

**Background**

A study was conducted to determine if galcanezumab (120 or 240 mg monthly), a humanized monoclonal antibody that selectively binds to calcitonin gene-related peptide, was superior to placebo in the prevention of chronic migraine.

**Materials and methods**

Eligible patients 18-65 years of age with chronic migraine (≥15 headache days per month, of which at least 8 met criteria for migraine) were randomized 2:1:1 to subcutaneous monthly injections of placebo (N=558), galcanezumab 120 (N=278) or 240 mg (N=277). The primary endpoint was the overall mean change-from-baseline in the number of monthly migraine headache days (MHD) during the 3-month double-blind treatment period. Key secondary measures included rates of ≥50%, ≥75%, and 100% reduction in monthly MHD, as well as the changes in monthly MHD with acute migraine treatments, the Role Function-Restrictive domain score of the Migraine-Specific Quality of Life Questionnaire (MSQ-RFR), and the Patient Global Impression-Severity of Illness (PGI-S).

**Results**

Baseline mean number of monthly MHD was 19.4. Both galcanezumab doses demonstrated a statistically significant difference (p<.001) compared with placebo in overall mean reduction in number of monthly MHD during the treatment period (placebo= -2.74; galcanezumab 120-mg= -4.83; galcanezumab 240-mg= -4.62), with statistical separation from placebo starting at Month 1. The rate of patients with ≥50% reduction from baseline in MHD was significantly greater for both galcanezumab doses compared with placebo (both p<.001). Compared with placebo, the 240-mg dose-group also had significantly higher rates of ≥75% response (p<.001), greater reductions in monthly MHD with acute migraine treatment (p<.001), and greater improvement in the MSQ-RFR (p<.001) and PGI-S (p<.001). There were no clinically meaningful differences between either galcanezumab dose-group and placebo on any safety parameters except for a higher incidence of injection site reaction (p<.05), injection site erythema (p<.01), and sinusitis (p<.05) all in the 240-mg dose-group relative to placebo.

**Conclusions**

Both doses of galcanezumab were superior to placebo in the reduction of monthly MHD, with significantly more patients reducing their monthly MHD by ≥50%. The 240-mg dose was also superior to placebo on most other key secondary measures. Both galcanezumab doses appeared to be efficacious, safe, and well-tolerated for the preventive treatment of chronic migraine.

**Acknowledgements**

This study is registered as NCT02614261 at ClinicalTrials.gov.

### O7 Improving the differential diagnosis between migraine with aura and transient ischemic attacks

#### Elena R Lebedeva^1,2^, Natalia M Gurary^3^, Denis V Gilev^4^, Anne F Christensen^5^, Jes Olesen^5^

##### ^1^Department of Neurology, the Ural State Medical University, Repina 3, Yekaterinburg, 620028, Russia;^2^International Headache Center “Europe-Asia”, Yekaterinburg, 620144, Russia;^3^Medical Union “New Hospital”, Yekaterinburg, Russia;^4^Department of econometrics and statistics, the Graduate school of Economics and Management, the Ural Federal University;^5^Danish Headache Center, Department of Neurology, Glostrup Hospital, University of Copenhagen, Copenhagen, Denmark

###### **Correspondence:**Elena R Lebedeva (cosmos@k66.ru)

**Background**

The differential diagnosis between migraine with aura (MA) and transient ischemic attacks (TIA) is difficult. Misclassification occurs often and has grave consequences for the patients. In the International classification of headache disorders 3^rd^ edition beta (ICHD-3beta) this was recognized and appendix criteria were given in the hope that they might better discriminate between these diseases. Conversely, misclassifications occur often in TIA clinics because of a lack of explicit diagnostic criteria for TIA.

**Methods**

We prospectively included 120 patients with TIA who were diagnosed according to the tissue based TIA definition and 1390 Danish patients and 152 Russian patients with MA. TIA was defined as new neurologic deterioration lasting less than 24 hours with no infarction in the relevant area on neuroimaging [1]. All received extensive semi structured face to face interviews about relevant clinical characteristics. Eligible patients with TIA had focal brain or retinal ischemia with resolution of symptoms without presence of new infarction on magnetic resonance imaging with diffusion weighted imaging (n=112) or computed tomography (n=8). We then tested main body and appendix criteria for MA for specificity against TIA patients and for sensitivity against the migraine materials. We developed explicit diagnostic criteria for TIA and tested them for sensitivity in the TIA patients and for specificity in the MA patients.

**Results**

The appendix criteria for MA had specificity versus TIA of 0.91 while the main body criteria were much less specific: 0.73. There was no major difference in sensitivity and we therefore recommend that the appendix criteria should replace the current main body criteria for MA. Our proposed explicit diagnostic criteria for TIA had a sensitivity of 1.0 which means that all 120 TIA patients fulfilled the criteria. The specificity of the TIA criteria tested against the Danish material of MA was 95% and tested against the Russian material it was 96%.

**Conclusion**

The field testing has led to preference for the appendix criteria for MA. We were able to design explicit diagnostic criteria for TIA with a very high sensitivity and specificity. We strongly recommend implementation of these criteria in migraine and cerebrovascular clinics.

**References**

1. Easton JD, Saver JL, Albers GW, Alberts MJ, Chaturvedi S, Feldmann E, et al. Definition and evaluation of transient ischemic attack. Stroke. 2009; 40: 2276–2293.

### O8 Rare primary headaches in Italian tertiary Headache centres: a nationwide retrospective epidemiologic survey (RegistRare study)

#### Chiara Lupi^1^, Roberto De Icco^2^, Luana Evangelista^3^, Valentina Favoni^4^, Antonio Granato^5^, Edoardo Mampreso^6^, Matteo Paolucci^7^, Lanfranco Pellesi^4^, Andrea Negro^9^, Raffaele Ornello^3^, Antonio Russo^10^, Martina Ulivi^7^, Sabina Cevoli^4^, Simona Guerzoni^8^, Silvia Benemei^1^

##### ^1^Headache Centre, Careggi University Hospital, Department of Health Sciences, University of Florence, Florence, Italy;^2^Headache Science Center, C. Mondino National Neurological Institute, Pavia, Italy;^3^Headache Centre, San Salvatore Hospital, ASL Abruzzo 1, Avezzano-Sulmona-L’Aquila, Italy;^4^Institute of Neurological Sciences of Bologna, Bellaria Hospital, Bologna, Italy;^5^Department of Medical, Technological and Translational Sciences, Headache Centre, University of Trieste, Italy;^6^Headache Centre, Medical Department, AULSS 6 Euganea, Padova, Italy;^7^Institute of Neurology, Università Campus Bio-Medico, Rome, Italy;^8^Headache and Drug Abuse Research Centre, Policlinico Hospital,University of Modena e Reggio Emilia, Modena, Italy;^9^Regional Referral Headache Center, Sant'Andrea Hospital, Rome, Italy;^10^Headache Centre, Neurology Department, University of Naples II, Naples, Italy

###### **Correspondence:** Silvia Benemei (silvia.benemei@unifi.it)

**Background**

Primary headache disorders are classified as rare when their prevalence in the general population is below 1%. The criteria for the diagnosis of rare primary headaches are detailed in Chapter 3 and Chapter 4 of part one of the International Classification of Headache Disorders, 3^rd^ edition (ICHD-3) [1]. For the majority of rare headaches, prevalence and incidence have been estimated by case reports or underpowered observational studies. This paucity of evidence also concerns the Italian population. Our study aims to estimate specific and overall prevalence and incidence of rare headaches, according to ICHD-3, in patients referred to tertiary Headache Centres in a 3-year period.

**Material and method**

Data regarding patients referred to tertiary Headache Centres from April 30, 2017 to May 1, 2014 were retrospectively collected, by means of a web-based open source platform (SurveyMonkey.org). Each participating Centre received the approval of the competent Ethics Committee before commencing any study procedures. Patients were asked to give their consent according to Italian law. Patients were identified through Italian Health System unique identifier, in order to avoid duplications of patients referred to more than one Centre.

**Results**

Eleven Headache Centres were asked to participate and 10 participated in the study as data can be easily retrieved from electronic sources used for clinical practice. To date, data from 6 centres have been collected. In the study period, a total of 15847 patients referred to the Centres, and 724 (4.5%) of them had a diagnosis (prevalence) of rare headaches. Headache disorders with higher prevalence were cluster headache, either episodic or chronic (n=393, 2.4%), primary stabbing headache (n=49, 0.3%) and new daily persistent headache (n=26,0.2%). Three hundred-ninety four (2.5%) patients received their first diagnosis of a rare headache in the same period.

**Conclusions**

Data collected in participating Headache Centres allow to initially depict rare headaches epidemiology. Retrospective data will be integrated by prospective data collected in a registry that has been started by participating Centres. These epidemiologic data will serve for investigations about clinical features, diagnostic and therapeutic management of rare, often neglected, primary headaches in Italy.

**References**

1. Headache Classification Committee of the International Headache Society (IHS). The International Classification of Headache Disorders, 3rd edition (beta version). Cephalalgia. 2013; 33:629-808.

2. Holle D, Obermann M. Rare primary headaches. Curr Opin Neurol. 2014; 27:332-336.

### O9 Non-invasive vagus nerve stimulation (nVNS) for the acute treatment of migraine: the randomised controlled PRESTO trial

#### Cristina Tassorelli^1^, Licia Grazzi^2^, Marina de Tommaso^3^, Giulia Pierangeli^4^, Paolo Martelletti^5^, Innocenzo Rainero^6^, Pierangelo Geppetti^7^, Anna Ambrosini^8^, Paola Sarchielli^9^, Eric Liebler^10^, Piero Barbanti^11^

##### ^1^Headache Science Centre, National Neurological Institute C. Mondino Foundation and University of Pavia, Pavia, Italy;^2^Headache Center, Carlo Besta Neurological Institute and Foundation, Milano, Italy;^3^Neurophysiology and Pain Unit, University of Bari Aldo Moro, Bari, Italy;^4^IRCCS Istituto delle Scienze Neurologiche di Bologna, Bologna, Italy;^5^Department of Clinical and Molecular Medicine, Sapienza University, Rome, Italy;^6^Department of Neuroscience, University of Turin, Turin, Italy;^7^Headache Centre, University Hospital of Careggi, Florence, Italy;^8^IRCCS Neuromed, Pozzilli (IS), Italy;^9^Neurologic Clinic, Santa Maria della Misericordia Hospital, Perugia, Italy;^10^electroCore, LLC, Basking Ridge, New Jersey, USA;^1^Headache and Pain Unit, IRCCS San Raffaele Pisana, Rome, Italy

###### **Correspondence:**Cristina Tassorelli (cristina.tassorelli@mondino.it)

**Background**

The safety, tolerability, and preliminary efficacy of non-invasive vagus nerve stimulation (nVNS; gammaCore®) have been demonstrated in clinical practice and pilot studies of acute migraine treatment [1,2]. The ease of use, flexibility, and favourable adverse event profile of nVNS make it an attractive option for patients. The aim of this study was to explore the efficacy, safety, and tolerability of nVNS for the acute treatment of migraine.

**Methods**

This multicentre, double-blind, randomised, controlled trial (RCT) included 248 patients with episodic migraine with or without aura from 10 Italian tertiary headache centres. Entry criteria and efficacy end points were consistent with existing guidelines and those of previous nVNS studies. Within 20 minutes from migraine pain onset, patients self-administered bilateral 120-second stimulations to the right and left sides of the neck. Patients were instructed to repeat both stimulations if pain did not improve by 15 minutes. Patients administered an optional additional set of stimulations at 120 minutes if not pain-free. Rescue medication use before 120 minutes was considered treatment failure. Up to 5 migraine attacks were treated in the double-blind period.

**Results**

Acute nVNS treatment (n=120) led to significantly higher pain-free rates than sham (n=123) for the first treated migraine attack at 30 minutes (12.7% vs 4.2%; *P*=0.012) and 60 minutes (21.0% vs 10.0%; *P*=0.023) but not at 120 minutes (30.4% vs 19.7%; *P*=0.067; primary end point; sensitivity analysis). To address the inconsistency between the 120-minute finding and the 2 earlier findings, a post hoc repeated-measures test confirmed that nVNS was superior to sham through 120 minutes (odds ratio: 2.3; 95% CI: 1.2, 4.4; *P*=0.012). Superiority of nVNS over sham was also seen for the rate of mild/no pain at 120 minutes (40.8% vs 27.6%; *P*=0.030) and ≥50% responder rates for no pain (32.4% vs 18.2%; *P*=0.020) and mild/no pain (47.6% vs 32.3%; *P*=0.026). Adverse event incidences were low, and most were mild and transient.

**Conclusions**

This RCT demonstrates that nVNS is rapidly effective, extremely well tolerated, and practical for the acute treatment of episodic migraine with or without aura. Significant benefits of nVNS versus sham were observed for pain freedom at 30 and 60 minutes but not at 120 minutes (primary end point). A repeated-measures test validated the primary end point, indicating the superiority of nVNS over sham through 120 minutes. Findings provide a clinical rationale for acute nVNS treatment in episodic migraine.

**Acknowledgements**

This study was sponsored by electroCore, LLC. We present this abstract on behalf of the PRESTO Study Group.

**Trial registration**

NCT02686034.

**References**

1. Goadsby PJ, Grosberg BM, Mauskop A, Cady R, Simmons KA. Effect of noninvasive vagus nerve stimulation on acute migraine: an open-label pilot study. *Cephalalgia*. 2014;34(12):986-993.

2. Barbanti P, Grazzi L, Egeo G, Padovan AM, Liebler E, Bussone G. Non-invasive vagus nerve stimulation for acute treatment of high-frequency and chronic migraine: an open-label study. *J Headache Pain*. 2015;16:61-65.

### O10 Differences between episodic and chronic migraine in white-matter tracts: a diffusion-tensor imaging study

#### Claudia Marsecano^1^, Alessandra Splendiani^1^, Riccardo Cornia^2^, Federico Bruno^1^, Luana Evangelista^2^, Francesca Pistoia^2^, Simona Sacco^2^

##### ^1^Radiology section, Department of Applied Clinical Science and Biotechnology, University of L’Aquila, L’Aquila, Italy;^2^Neurology section, Department of Applied Clinical Science and Biotechnology, University of L’Aquila, L’Aquila, Italy

###### **Correspondence:**Simona Sacco (simona.sacco@univaq.it)

**Background**

Several studies indicated that migraine patients have tract-specific alterations in several white-matter tracts as compared to controls [1-12]. We investigated differences in white-matter tracts between episodic migraine (EM) patients and chronic migraine (CM) patients.

**Materials and methods**

We included consecutive eligible patients with EM or CM and referring to a tertiary headache center. We selected Caucasian women aged from 30 to 60 years. EM and CM patients were matched by age ±1. Imaging was conducted on a single 3-Tesla magnetic resonance imaging (MRI) scanner. Diffusion tensor data (DTI) data were processed using TRActs Constrained by Underlying Anatomy (TRACULA) for automatic reconstruction of a set of major white-matter pathways from diffusion-weighted MRI.

**Results**

We included 20 EM patients (mean age±SD 43.2±6.9) and 20 CM patients (mean age±SD 43.8±6.9). Years lived with migraine were similar in EM (mean±SD 14.3±7.0) and CM (mean±SD 18.6±9.0) patients (P=0.118). Mean±SD number of days with headache per month was 3.3±1.8 for EM; 9 (45%) CM patients had drug abuse.

We found differences in mean diffusivity (MD) between EM and CM patients in the left superior longitudinal fasciculus temporal (Table 1). We found differences in mean fractional anisotropy (FA) between EM and CM patients bilaterally in the right inferior longitudinal fasciculus and in the left superior longitudinal fasciculus temporal (Table 1).

EM patients showed a negative age-adjusted correlation between years lived with migraine and FA in the uncinate fasciculus (r=-.501; P=.048). CM patients showed a positive age-adjusted correlation between years lived with migraine and MD in the corpus callosum forceps major (r=.500; P=.049) and in the right anterior thalamic radiation (r=.492; P=.050). We did not find any correlation between frequency of migraine attacks and MD or FA nor in the EM nor in the CM group.

**Conclusions**

We found differences in white matter tracts between EM and CM patients. Our results also indicate that the longer the years lived with migraine the greater is the impairment in selected white-matter tracts. In summary, our data suggest that migraine type and number of years with migraine affects white-matter tract status.

**References**

1. Chong CD, Schwedt TJ. Migraine affects white-matter tract integrity: A diffusion-tensor imaging study. Cephalalgia 2015;35:1162-1171.

2. Coppola G, Tinelli E, Lepre C, Iacovelli E, Di Lorenzo C, Di Lorenzo G, Serrao M, Pauri F, Fiermonte G, Bianco F, Pierelli F. Dynamic changes in thalamic microstructure of migraine without aura patients: A diffusion tensor magnetic resonance imaging study. Eur J Neurol 2014;21:287-e13.

3. Granziera C, DaSilva AF, Snyder J, Tuch DS, Hadjikhani N. Anatomical alterations of the visual motion processing network in migraine with and without aura. PLoS Med 2006;3:e402.

4. Kara B, Kiyat Atamer A, Onat L, Ulusoy L, Mutlu A, Sirvanci M. DTI findings during spontaneous migraine attacks. Clin Neuroradiol 2013;23:31-36.

5. Liu J, Lan L, Li G, Yan X, Nan J, Xiong S, Yin Q, von Deneen KM, Gong Q, Liang F, Qin W, Tian J. Migraine-related gray matter and white matter changes at a 1-year follow-up evaluation. J Pain 2013;14:1703-1708.

6. Rocca MA, Colombo B, Inglese M, Codella M, Comi G, Filippi M. A diffusion tensor magnetic resonance imaging study of brain tissue from patients with migraine. J Neurol Neurosurg Psychiatry 2003;74:501-503.

7. Rocca MA, Pagani E, Colombo B, Tortorella P, Falini A, Comi G, Filippi M. Selective diffusion changes of the visual pathways in patients with migraine: A 3-T tractography study. Cephalalgia 2008;28:1061-1068.

8. Schmitz N, Admiraal-Behloul F, Arkink EB, Kruit MC, Schoonman GG, Ferrari MD, van Buchem MA. Attack frequency and disease duration as indicators for brain damage in migraine. Headache 2008;48:1044-1055.

9. Yu D, Yuan K, Zhao L, et al. Yuan K, Zhao L, Dong M, Liu P, Yang X, Liu J, Sun J, Zhou G, Xue T, Zhao L, Cheng P, Dong T, von Deneen KM, Qin W, Tian J. White matter integrity affected by depressive symptoms in migraine without aura: a tract-based spatial statistics study. NMR Biomed 2013;26:1103-1112.

10. Yu D, Yuan K, Qin W, Zhao L, Dong M, Liu P, Yang X, Liu J, Sun J, Zhou G, von Deneen KM, Tian J. Axonal loss of white matter in migraine without aura: A tract-based spatial statistics study. Cephalalgia 2013; 33: 34-42.

11. Yuan K, Qin W, Liu P, Zhao L, Yu D, Zhao L, Dong M, Liu J, Yang X, von Deneen KM, Liang F, Tian J. Reduced fractional anisotropy of corpus callosum modulates inter-hemispheric resting state functional connectivity in migraine patients without aura. PloS One 2012;7:e45476.

### O11 Headache following head injury: A population-based longitudinal cohort study (HUNT)

#### Lena Hoem Nordhaug^1^, Knut Hagen^1,2^, Anne Vik^1,3^, Lars Jacob Stovner^1,2^, Turid Follestad^4^, Torunn Pedersen^5^, Gøril Bruvik Gravdahl^2^, Mattias Linde^1,2^

##### ^1^Department of Neuromedicine and Movement Science (INB), Faculty of Medicine and Health Sciences, Norwegian University of Science and Technology, Trondheim, Norway;^2^Norwegian Advisory Unit on Headaches, St. Olavs University Hospital, Trondheim, Norway;^3^Department of Neurosurgery, St. Olavs University Hospital, Trondheim, Norway;^4^Department of Public Health and Nursing, Faculty of Medicine and Health Sciences, Norwegian University of Science and Technology, Trondheim, Norway;^5^Division of Mental Health and Addiction, Oslo University Hospital, Oslo, Norway

###### **Correspondence:**Lena Hoem Nordhaug (lena.h.nordhaug@ntnu.no)

**Objective**

To explore whether subjects exposed to head injury more often developed a new headache or experienced exacerbation of previously reported headache compared to non-exposed.

**Methods**

This population-based historical cohort study included headache data from two large epidemiological surveys performed with an 11-year interval. This was linked with data from hospital records on exposure to head injury occurring between the health surveys. Participants in the surveys who had not been hospitalized because of a head injury comprised the control group. The head injuries were classified according to the Head Injury Severity Scale (HISS). Multinomial logistic regression was performed to investigate the association between head injury and new headache or exacerbation of pre-existing headache in a population with known pre-injury headache status, controlling for potential confounders.

**Results**

The exposed group consisted of 294 individuals and the control group of 25,662 individuals. In multivariate analyses, adjusting for age, sex, anxiety, depression, education level, smoking and alcohol use, mild head injury increased the risk of new onset headache suffering (OR 1.74, 95% CI 1.05 – 2.87), stable headache suffering (OR 1.70, 95% CI 1.15 – 2.50) and exacerbation of previously reported headache (OR 1.93, 95% CI 1.24 – 3.02). The reference category was participants without headache in both surveys.

**Conclusion**

Individuals exposed to a head injury were more likely to have new onset and worsening of pre-existing headache and persistent headache, compared to the surrounding general population. The results support the entity of the ICHD-3 beta diagnosis “persistent headache attributed to traumatic injury to the head”.

### O12 Cerebral grey matter density is abnormally reduced in chronic migraine patients: correlations with clinical features

#### Gianluca Coppola^1^, Barbara Petolicchio^2^, Antonio Di Renzo^1^, Emanuele Tinelli^2^, Cherubino Di Lorenzo^3^, Vincenzo Parisi^1^, Mariano Serrao^4^, Valentina Calistri^2^, Stefano Tardioli^2^, Gaia Cartocci^2^, Francesca Caramia^2^, Vittorio Di Piero^2^, Francesco Pierelli^4,5^

##### ^1^G.B. Bietti Foundation IRCCS, Research Unit of Neurophysiology of Vision and Neurophthalmology, Rome, Italy;^2^“Sapienza” University of Rome, Department of Neurology and Psychiatry, Rome, Italy;^3^Don Carlo Gnocchi Onlus Foundation, Milan, Italy;^4^“Sapienza” University of Rome Polo Pontino, Department of Medico-Surgical Sciences and Biotechnologies, Latina, Italy;^5^IRCCS-Neuromed, Pozzilli (IS), Italy

###### **Correspondence:**Gianluca Coppola

**Background**

Few neuroimaging studies have explored brain morphometry in patients affected by chronic migraine (CM). Most of these studies were performed in patients with medication overuse. Here, we performed voxel-based morphometry (VBM) analysis to investigate the grey matter (GM) density of the whole brain in patients affected by CM without medication overuse. Our aim was to investigate whether there are fluctuations in the GM densities in relation to the patients’ clinical features.

**Materials and methods**

Twenty untreated CM patients without a past medical history of medication overuse underwent 3T MRI scans and were compared to a group of 20 healthy volunteers (HV). SPM12 and CAT12 toolbox were used to process MRI data and to perform VBM analysis of structural T1-weighted MRI scans. The patients’ versus HV relative GM density was assessed with an uncorrected threshold of p < 0.005. To check for possible correlations, patients’ clinical features and GM maps were regressed.

**Results**

Compared to HV, CM patients presented 4 clusters of significantly lower GM densities: I) right cerebellar lobules (VIIIa, CrusII), II) the left occipital areas (BA17/BA18), III) the left middle temporal gyrus, and IV) the left temporal pole /amygdala /pallidum /orbitofrontal cortex. The GM density of cerebellar hemispheres correlated negatively with the years of headache disease, and positively with the number of tablets intake per month.

**Conclusions**

To summarize, CM is associated with lower GM density in several brain areas known to be involved in antinociception, multisensory integration, and analgesic dependence. The GM density within the cerebellum was significantly related to longer duration of headache disease and to higher consumption of acute headache medications. We hypothesize that this morphological pattern we identified may be considered as a predisposing ground on which to develop medication overuse headache.

### O13 The relationship between pain, psychiatric, and endocrine/neurological comorbidities of migraine: Results from the Chronic Migraine Epidemiology and Outcomes (CaMEO) study

#### Richard B. Lipton^1^, Vincent T. Martin^2^, Michael L. Reed^3^, Kristina M. Fanning^3^, Aubrey Manack Adams^4^, Dawn C. Buse^1^

##### ^1^The Saul R. Korey Department of Neurology, Albert Einstein College of Medicine, Bronx, NY, USA;^2^University of Cincinnati Headache and Facial Pain Center, University of Cincinnati College of Medicine, Cincinnati, OH, USA;^3^Vedanta Research, Chapel Hill, NC, USA;^4^Global Medical Affairs, Allergan plc, Irvine, CA, USA

###### **Correspondence:**Richard B. Lipton (Richard.Lipton@einstein.yu.edu)

**Background**

Migraine is comorbid with various conditions, many with a greater relative frequency in chronic migraine (CM) versus episodic migraine (EM). The objective of this study was to replicate and extend work on the comorbidity of pain, psychiatric, and endocrine/neurological symptoms and conditions in a systematically recruited sample of people with EM and CM.

**Materials and Methods**

Data from the prospective web-based baseline survey of the Chronic Migraine Epidemiology and Outcomes (CaMEO) Study were used to identify people, recruited from an online panel using quota sampling, with EM and CM based on criteria modified from the International Classification of Headache Disorders, third edition, beta version. Participants completed a Comorbidities/Endophenotypes module that assessed 64 symptoms (e.g., neck pain) and conditions (e.g., rheumatoid arthritis). Respondents were asked (1) if they ever had a specific symptom (“Self-Reported [SR]”) and, if present, (2) if the SR symptom or condition had been confirmed/diagnosed by a “doctor” (“SR-physician diagnosis [SR-PD]”). Chi-square analysis was used to compare the relative frequency of symptoms and conditions in respondents with EM versus CM. This report presents data on symptoms and conditions from the Pain, Psychiatric, and Endocrine/Neurological comorbidity categories.

**Results**

Available CaMEO respondents with migraine (16,763) were sent the Comorbidities/Endophenotype module and 12,810 (76.4%: EM, 11,699; CM, 1,111) provided valid responses. Compared with the EM group, the CM group had a similar mean age (EM, 41.3 years; CM, 41.9 years), was more likely to be female (EM, 74.2%; CM, 81.5%; *P*<0.001) and white (EM, 84.0%; CM, 88.7%; *P*<0.001), and had a mean higher body mass index (EM, 27.7 kg/m^2^; CM, 28.7 kg/m^2^; *P*<0.001). The relative frequencies were significantly higher for 24 (85.7%) of the 28 SR symptoms and SR-PD conditions assessed (Fig. 1). 5 of these conditions had relative frequencies >10% higher in CM than EM: chronic back pain (EM, 22.5%; CM, 37.6%), chronic pain (EM, 7.4%; CM, 22.2%), neck pain (EM, 38.1%; CM, 55.3%), anxiety (EM, 25.7%; CM, 42.2%), and depression (EM, 28.1%; CM, 45.6%).

**Conclusions**

Overall, significantly more respondents with CM versus EM reported having specific symptoms or conditions. Mechanisms explaining this association might include direct causality (e.g., CM causes the comorbidity), reverse causality (e.g., the condition increases CM risk), and shared genetic or environmental risk factors. Confounding, or detection bias (i.e., “Berkson’s Bias”) could also contribute.

**Fig. 1 (abstract O16).**
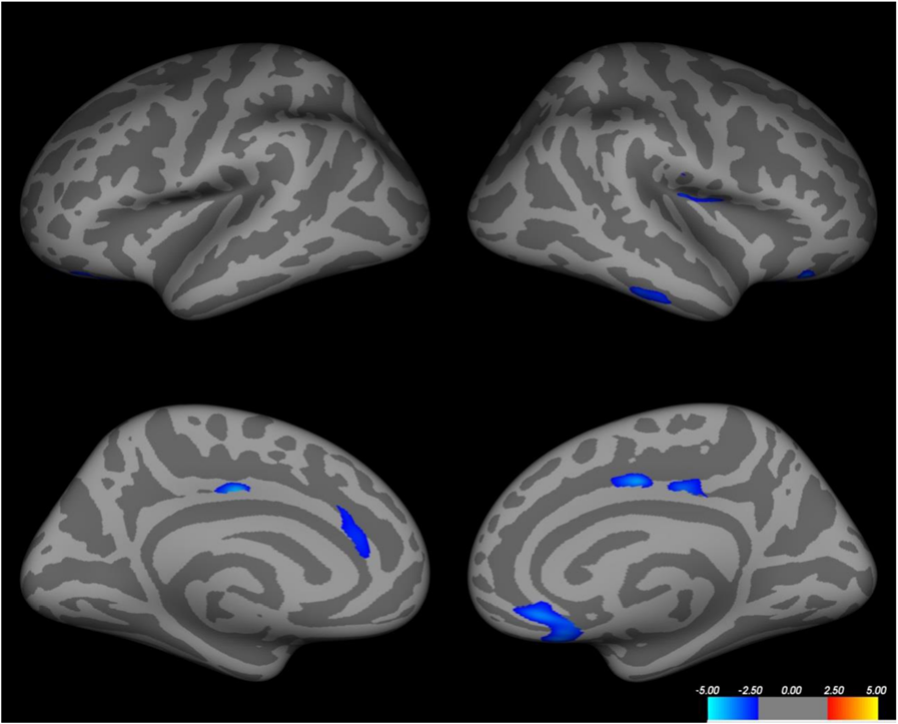
Significant differences between CM and EM groups expressed in log10p and following the application of the False Discovery Rate (FDR) model

**Acknowledgments**

Editorial support for development of this abstract was provided by Lee B. Hohaia, PharmD, and Dana Franznick, PharmD, at Complete Healthcare Communications, LLC (Chadds Ford, PA), a CHC Group company, and funded by Allergan, plc (Dublin, Ireland).

### O14 Effects of calcitonin gene-related peptide and sildenafil on CNS metabolism: A pharmacological proton magnetic resonance spectroscopy study at 3.0 T

#### Samaira Younis1, Anders Hougaard1, Casper Emil Christensen1, Mark Bitsch Vestergaard2, Esben Thade Petersen3, Olaf Bjarne Paulson4, Henrik Bo Wiberg Larsson2, Messoud Ashina1

##### ^1^Danish Headache Center and Department of Neurology, Rigshospitalet Glostrup, Faculty of Health and Medical Sciences, University of Copenhagen, Denmark;^2^Functional Imaging Unit, Department of Clinical Physiology, Nuclear Medicine and PET, Rigshospitalet Glostrup, Faculty of Health and Medical Sciences, University of Copenhagen, Denmark;^3^Danish Research Centre for Magnetic Resonance, Centre for Functional and Diagnostic Imaging and research, Amager and Hvidovre Hospital, Denmark;^4^Neurobiology Research Unit, Department of Neurology, Rigshospitalet, Copenhagen, Denmark and Department of Clinical Medicine, University of Copenhagen, Denmark

###### **Correspondence:**Messoud Ashina

**Background**

Human pharmacological imaging has been used with great success to study the cerebrovascular effects of headache-inducing substances, including calcitonin gene-related peptide (CGRP) and sildenafil. The role of the glutamate alterations in the deep brain structures pons and thalamus is of emerging interest but to date, methods to non-invasively study the pharmacologically induced biochemical effects in these areas have not been validated.

**Materials and methods**

We applied an optimized *in vivo* human pharmacological proton (1H) magnetic resonance spectroscopy (MRS) protocol in a randomized, placebo-controlled, double-blinded, double-dummy, three-way cross-over design, using the headache-inducing substances calcitonin gene-related peptide (CGRP) and sildenafil, with peripheral and central modes of action on the nervous system respectively. Seventeen healthy volunteers were scanned with our optimized 1H-MRS protocol to investigate the CGRP- and sildenafil-induced glutamate+glutamine (Glx) changes in both pons and thalamus. In addition, we systematically investigated the Glx reproducibility and variability in pons and thalamus, based on repeated measurements on the same day and three separate days, in order to validate our method.

**Results**

We found that spectral quality was good for measurements in pons and thalamus with relatively low spectral line widths and high signal-noise-ratio. The Glx measurement variation was overall low in both pons and thalamus. The Glx level increased in pons at 40 – 70 min after sildenafil administration, when compared to placebo (5.6%, P=0.037). We found no sildenafil-induced Glx changes in thalamus, and no CGRP-induced Glx changes in pons or thalamus.

**Conclusions**

Our findings demonstrate that Glx measurements can be reliably obtained from pons and thalamus. The sildenafil-induced changes in pons likely reflect increased excitability of the pontine neurons. CGRP did not induce pontine or thalamic metabolic changes, indicating that it does not modify the neuronal excitability in these central deep brain structures, but rather exerts its effects on the peripheral nervous system and/or the trigeminal ganglion.

### O15 Effect of calcitonin gene-related peptide and sildenafil on intracranial dural arteries: A 3T MR- angiography study in healthy volunteers

#### Casper Emil Christensen^1^, Faisal Mohammad Amin^1^, Samaira Younis^1^, Ulrich Lindberg^2^, Patrick de Koning^3^Esben Thade Petersen^4^, Olaf Bjarne Paulson^5^; Henrik Bo Wiberg Larsson^2^; Messoud Ashina^1^

##### ^1^Danish Headache Center and Department of Neurology, Rigshospitalet Glostrup, Faculty of Health and Medical Sciences, University of Copenhagen, Denmark;^2^Functional Imaging Unit, Department of Clinical Physiology, Nuclear Medicine and PET, Rigshospitalet, Faculty of Health and Medical Sciences, University of Copenhagen, Denmark;^3^Division of Image Processing, Department of Radiology, Leiden University Medical Center, Leiden, Netherlands;^4^Danish Research Centre for Magnetic Resonance, Centre for Functional and Diagnostic Imaging and research, Amager and Hvidovre Hospital, Denmark;^5^Neurobiology Research Unit, Department of Neurology, Rigshospitalet, Copenhagen, Denmark and Department of Clinical Medicine, University of Copenhagen, Denmark

###### **Correspondence:**Messoud Ashina

**Background**

Calcitonin gene-related peptide (CGRP) and sildenafil are vasoactive substances that induce migraine attacks in patients. The intradural arteries are thought to be involved, but these have never been examined *in vivo*. Furthermore, sildenafil is the only migraine inducing compound, where cephalic dilation is not reported. Here, we investigate the effect of CGRP and sildenafil on the extracranial and intradural parts of the middle meningeal artery (MMA).

**Methods**

In a double-blind, randomized, three-way crossover, placebo-controlled head-to-head comparison of CGRP and sildenafil, MR-angiography was recorded in healthy volunteers at baseline and twice after study drug administration. Circumferences of extracranial and intradural MMA segments were measured using semi-automated analysis software. The area under the curve (AUC) for circumference change was compared using paired *t*-tests between study days.

**Results**

Twelve healthy volunteers completed the study. The AUC_Baseline-120min_ was significantly larger on both the CGRP *and* sildenafil days in the extracranial MMA (CGRP, *p*=0.0003; sildenafil, *p*=0.021) and the intradural MMA (CGRP, *p*=0.013; sildenafil, *p*=0.027) compared to placebo.

Peak dilation of the extracranial MMA after CGRP was 15.7% (95% CI [11.2-20.1]) and 18.9% (95% CI [12.8-24.9]) after sildenafil. Peak intradural MMA dilation was 12.5% (95% CI [8.1-16.8]) after CGRP and 9.9% (95% CI [2.9-16.9]) after sildenafil.

**Conclusion**

An important novel finding of the present study is that both CGRP and sildenafil dilate intradural arteries, supporting the notion that *all* pharmacological migraine triggers, so far, do indeed dilate cephalic vessels. We suggest that intradural artery dilation is associated with headache induced by CGRP and sildenafil.

### O16 Volumetric differences between episodic and chronic migraine: a volumetric neuroimaging study

#### Francesca Pistoia^1^, Riccardo Cornia^1^, Claudia Marsecano^2^, Federico Bruno^2^, Luana Evangelista^1^, Alessandra Splendiani^2^, Simona Sacco^1^

##### ^1^Neurology section, Department of Applied Clinical Science and Biotechnology, University of L’Aquila, L’Aquila, Italy;^2^Radiology section, Department of Applied Clinical Science and Biotechnology, University of L’Aquila, L’Aquila, Italy

###### **Correspondence:**Francesca Pistoia (francesca.pistoia@univaq.it)

**Background**

Chronic migraine (CM) may be associated to specific cortical and subcortical volumetric changes [1-4]. However, it is not clear whether there is a causal relationship between these changes and the clinical course of migraine or whether they are rather an epiphenomenon. The objective of this study was to identify the main structural changes associated with CM and the relationship with the disease duration.

**Materials and methods**

We included consecutive eligible patients with episodic migraine (EM) or CM referring to a tertiary headache center. We selected Caucasian women aged from 30 to 60 years. EM and CM patients were matched by age ±1. The assessment was performed through a single 3-Tesla magnetic resonance imaging (MRI) acquisition followed by a morfovolumetric analysis through the Freesurfer software [5-7]. Groups analyses were performed through the QDec software for cortical volumes (level of significance fixed at p 0.01) and through the SPSS V22.0 software for subcortical volumes (level of significance fixed at p 0.05).

**Results**

We included 17 EM patients (mean age±SD 42.4±6.1) and 17 CM patients (mean age±SD 43.1±6.4). Years lived with migraine were not significantly different in EM (mean±SD 18.2±10.6) as compared to CM (mean±SD 21.4±11.6) patients (p=0.749).

CM patients showed a selective cortical volume loss involving the insular cortex (p<0.005), the medial (p<0.005) and lateral (p<0.001) orbitofrontal cortex, the posterior cingulate cortex (p<0.001) and the inferior temporal cortex (p<0.005) in the right hemisphere, and the lateral orbitofrontal cortex (p<0.001), the posterior cingulate cortex (p<0.001) and the caudal anterior cingulate cortex (p<0.001) in the left hemisphere. At subcortical level volume differences involved the left and the right cerebellar cortex (p<0.05) and the medium and posterior portion of the corpus callosum (p<0.05).

With respect to disease duration, a negative age-adjusted correlation between years lived with migraine and volumetric reduction was found in right (p<0.001) and left (p<0.005) middle temporal cortex in EM patients and in the right superior frontal cortex (p<0.001), cuneus (p<0.005) and lingual cortex (p<0.005), and left superior frontal cortex (p<0.001) in CM patients. No correlations between disease duration and subcortical changes were found in the two groups.

**Conclusions**

Regions showing a volumetric reduction might represent crucial hubs in migraine activity and pathophysiology. A better understanding of their role in the basal disease as well as in the process of chronification can provide a model to improve treatment options in patients with migraine [8].

**References**

1. Bilgiç B, Kocaman G, Arslan AB, Noyan H, Sherifov R, Alkan A, Asil T, Parman Y, Baykan B. Volumetric differences suggest involvement of cerebellum and brainstem in chronic migraine. Cephalalgia 2016;36:301-8.

2. Yu ZB, Peng J, Lv YB, Zhao M, Xie B, Liang ML, Li HT, Zhou ZH. Different mean thickness implicates involvement of the cortex in migraine. Medicine (Baltimore) 2016;95:e4824.

3. Neeb L, Bastian K, Villringer K, Israel H, Reuter U, Fiebach JB. Structural Gray Matter Alterations in Chronic Migraine: Implications for a Progressive Disease? Headache 2017;57:400-416.

4. May A, Matharu M. New insights into migraine: application of functional and structural imaging. Curr Opin Neurol 2007;20:306-9.

5. Dale AM, Fischl B, Sereno MI. Cortical surface-based analysis. I. Segmentation and surface reconstruction. Neuroimage 1999; 9: 179-94.

6. Fischl B, Dale AM. Measuring the thickness of the human cerebral cortex from magnetic resonance images. Proc Natl Acad Sci USA 2000; 97:11050-5.

7. Fischl B, Sereno MI, Dale AM. Cortical surface-based analysis. II: Inflation, flattening, and a surface-based coordinate system. Neuroimage 1999; 9:195-207.

8. Hubbard CS, Becerra L, Smith JH, DeLange JM, Smith RM, Black DF, Welker KM, Burstein R, Cutrer FM, Borsook D. Brain Changes in Responders vs. Non-Responders in Chronic Migraine: Markers of Disease Reversal. Front Hum Neurosci 2016;10:497.

### O17 A population based survey for headaches in Greece

#### Dimos D. Mitsikostas^1^, Chrisanthy Arvanity^2^, Theodoros Constantinidis^3^, Manolis Dermitzakis^4^, Nikolaos Fakas^5^, Jobst Rudolf^6^, Michail Vikelis^7^, on behalf of the Hellenic Headache Society

##### ^1^First Neurology Department, Aeginition Hospital, School of Medicine, National & Kapodistrian University of Athens, Athens, Greece;^2^Second Neurology Department, Attikon Hospital, School of Medicine, National & Kapodistrian University of Athens, Athens, Greece;^3^Private Headache Clinic, Korinthos, Greece;^4^Department of Neurology, “Geniki Kliniki” Euromedica, Thessaloniki, Greece;^5^401 Army General Hospital of Athens, Neurology Department, Athens, Greece;^6^Neurology Department, Papageorgiou Hospital, Thessaloniki, Greece;^7^Headache Clinic, Mediterraneo Hospital, Glyfada, Greece

We aimed to investigate the prevalence of headache in General Population (adults 18-70 years old) in Greece. A quantitative study, using the form of computer-assisted telephone interviews (C.A.T.I.) was designed. A draft questionnaire consisting of 37 questions was delivered in 145 headache sufferers in a pre-study work to evaluate the diagnosis of the primary headache disorder according to ICH-3beta diagnostic criteria. After the analysis of this questionnaire the specific 37-item questionnaire was decided. In total, N=10,008 interviews, representative of the population of Greece in terms of gender, age, and area, based on the most recent census (ELSTAT, 2011) were performed using the structured evaluated questionnaire. Based on the above contacts, n=1,197 respondents (12% of the sample) were found to suffer from headaches that reduce their performance. The one-year prevalence of Migraine that reduces activity was 8.2% (n=0.6m population) of Tension-Type Headache (TTH) 3.8% (n=0.28m of population) and of Cluster Headache 0.01% (n=0.74K of population). Chronic migraine one-year prevalence was 1% (n=0.7K of population). Females tend to suffer more from migraines and TTH as well as ages 35-54. The average patients has been suffering from headaches for 12 years. Headaches typically occur once a month or more frequently, 8 days per month on average. Although patients rarely misss work due to headaches, they do report headache-induced reductions in performance around 3 days per month. Slighly less than half patients have felt bad/ humiliated because of headaches, while social/family obligations are affected 3 days per month on average. About one fifth of patients seek professional treatment for headaches, most of them in the private sector. The most popular specialty for headache treatment is neurologist, followed by internist. Regarding both prophylactic and acute treatment, patients prefer oral medication to injection, even if the former is administered more frequently. They also prefer oral medication/ injection to a stimulation device. The stimulation device seems to be more attractive to males. Painkillers also are by far the most common acute treatment for headaches and the vast majority of patients have never taken prophylaxis for headaches. Only a small fraction have stopped taking a prophylactic treatment due to adverse effects. Interstingly, patients would be willing to spend 20€ on average per month for headache treatment, on average.

### O18 Phase 3 Studies (EVOLVE-1 & EVOLVE-2) of Galcanezumab in Episodic Migraine: Results of 6-Month Treatment Phase

#### Vladimir Skljarevski^1^, Virginia L. Stauffer^1^, Qi Zhang^1^, Holland C. Detke^1^, Brian A. Millen^1^, Jyun Yan Yang^1^, Katherine J. Selzler^1^, Robert Conley^1,2^, Sheena K. Aurora^1^

##### ^1^Eli Lilly and Company, Indianapolis, IN, USA;^2^University of Maryland School of Medicine, Baltimore, MD, USA

**Objective**

Galcanezumab, a humanized monoclonal antibody that selectively binds to the calcitonin gene-related peptide, was investigated in two Phase 3 studies (EVOLVE-1 and EVOLVE-2) to determine superiority to placebo in the prevention of migraine headache.

**Methods**

EVOLVE-1 and EVOLVE-2 were double-blind, 6-month studies in patients with episodic migraine (4 to 14 monthly migraine headache days [MHD]) conducted in North America and globally, respectively. Patients were randomized 2:1:1 to monthly subcutaneous injections of placebo, galcanezumab 120 mg or 240 mg. Primary endpoint was overall mean change from baseline in the number of monthly MHD during Months 1-6. Key secondary measures included rates of ≥50%, ≥75%, and 100% reduction in monthly MHD and overall mean change from baseline in monthly MHD with acute migraine treatments, and mean change from baseline over Months 4-6 on the Role Function-Restrictive domain score of the Migraine-Specific Quality of Life Questionnaire (MSQ-RFR) and Patient Global Impression-Severity of Illness (PGI-S).

**Results**

Baseline mean number of monthly MHD was 9.1 for both studies. Both galcanezumab doses demonstrated a statistically significant improvement compared with placebo (both studies p<.001) for overall mean change in monthly MHD (EVOLVE-1: placebo=-2.81; GMB 120 mg=-4.73; GMB 240 mg=-4.57; EVOLVE-2: placebo=-2.28; GMB 120 mg=-4.29; GMB 240 mg=-4.18). Percentage of patients with MHD reductions of ≥50%, ≥75%, or 100% were significantly higher for each galcanezumab dose compared with placebo (both studies p<.001). Patients had a significantly greater overall mean reduction of monthly number of MHD with acute migraine treatment for both galcanezumab doses relative to placebo (both studies p<.001). Mean change in MSQ-RFR and PGI-S ratings were statistically significant for each galcanezumab dose versus placebo (MSQ-RFR: p<.001 and PGI-S: p<.05, in both studies). There were no statistically significant differences between galcanezumab and placebo on the most common treatment-emergent adverse events except for a greater incidence of injection-site pruritus (both studies/doses p<.01) and injection-site reaction (both studies/doses p<.05), and injection-site erythema (p<.05, galcanezumab 240 mg) in EVOLVE-2.

**Conclusions**

Both doses of galcanezumab met the primary and all key secondary objectives, after adjusting for multiplicity. Treatment effects were similar across galcanezumab doses for efficacy, and safety; however, there was a higher rate of injection-site pruritus and reaction in galcanezumab-treated patients in both studies. EVOLVE-1 and EVOLVE-2 demonstrated that galcanezumab, at either 120 mg or 240 mg monthly, provided clinical benefit and improved function in patients with episodic migraine.

**Acknowledgements**

Studies were registered as NCT02614183 and NCT02614196 at ClinicalTrials.gov.

### O19 Real-life use of onabotulinumtoxinA for the symptomatic treatment of chronic migraine: The Repose Study

#### Fayyaz Ahmed^1^, Charly Gaul^2^, Paolo Martelletti^3^, Juan Carlos Garcia-Monco^4^, Aubrey Manack Adams^5^

##### ^1^Spire Hesslewood Clinic, Hull York Medical School, Brough, Hull, UK;^2^Department of Headache and Facial Pain, Migraine and Headache Clinic, Koenigstein, Germany;^3^Department of Clinical and Molecular Medicine, Sapienza University, Regional Referral Headache Centre, Rome, Italy;^4^Service of Neurology, Hospital de Galdakao, Vizcaya, Spain;^5^Global Medical Affairs, Allergan plc, Irvine, CA, USA

###### **Correspondence:**Fayyaz Ahmed (fayyaz.ahmed@hey.nhs.uk)

**Background**

The REPOSE Study, a European, multicenter, prospective, non-interventional study, investigated the effectiveness and safety of real-life use of onabotulinumtoxinA for chronic migraine (CM).

**Materials and Methods**

Adults prescribed onabotulinumtoxinA for CM were enrolled. Patients received onabotulinumtoxinA approximately every 12 weeks according to their physician’s usual practice, guided by the Summary of Product Characteristics. OnabotulinumtoxinA injection practices, safety, headache-day frequency, and Migraine Specific Quality of Life Questionnaire (MSQ) were collected at baseline and follow-up visits.

**Results**

Among 644 patients enrolled, 633 patients received ≥1 onabotulinumtoxinA dose for a total of 3499 onabotulinumtoxinA treatments. Patients had a mean (SD) age of 45.4 (12) years, were typically women (85.3%) and had a mean of 20.6 headache days/month. The median dose and median number of injection sites of onabotulinumtoxinA per session (baseline up to follow-up session 8) was 155 U and 31 sites, respectively. Through follow-up session 8, patient-reported estimates of headache days/month (≥4 hours) were significantly reduced from baseline (*P*<0.001 at each follow-up session, Fig. 1). The MSQ domain scores (Role Restrictive, Preventive, and Emotional) were significantly reduced from baseline at each follow-up session. Adverse drug reactions, typically of mild to moderate severity, were reported by 18.3% of patients; eyelid ptosis (5.4%), neck pain (3.0%), and musculoskeletal stiffness (2.7%) were most frequently reported.

**Conclusion**

Preventive treatment of CM with onabotulinumtoxinA in a longer-term (24-month) real-world setting sustains a reduction in the frequency of headache days and significantly improves quality of life relative to baseline. No new safety concerns were identified.

**Acknowledgments**

Editorial support for development of this abstract was provided by Lee B. Hohaia, PharmD, and Dana Franznick, PharmD, at Complete Healthcare Communications, LLC (Chadds Ford, PA), a CHC Group company, and funded by Allergan, plc (Dublin, Ireland).

**Funding**

Allergan plc.

**Fig. 1 (abstract O19).**
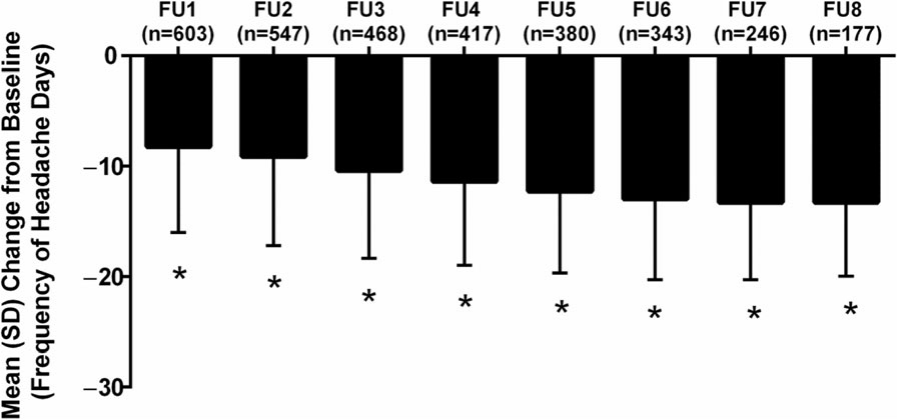
Headache-day frequency calculated using the patient-reported estimate of number of days in a month with headache. Wilcoxon signed-rank test for change versus baseline (level of significance: 5%). The number of patients in follow-up (FU) sessions 9-12 were: FU9, n=67; FU10, n=38; FU11, n=21; FU12, n=16. The mean change from baseline for FU sessions 9-12 were each significant (*P<0.001)

### O20 Abnormal peripheral and central visual processing in migraine

#### Filippo Brighina, Elisa Catalano, Viviana Firpo, Giuseppe Cosentino, Salvatore Cillino, Brigida Fierro

##### Dept of Experimental Biomedicine and Clinical Neuroscience, University of Palermo, Italy

**Objectives:** sound induced flash illusion (SIFI) is an illusory cross modal(audio-visual) phenomenon critically dependent upon excitability of visual cortex [1]. A recent study with SIFI confirmed hyperexcitability of visual cortex in migraine [2]; patients with migraine show abnormality of chromatic perception with a more relevant dysfunction in the wave-range of blu(S cones) [3]. Aims of the study were explore the relationship between peripheral chromatic and central visual dysfunction evaluating also the presence of functional receptor impairment in patients with migraine, with and without aura examined interictally.

**Subjects**: 15 migraine patients with aura (MWA) and 15 without aura(MWoA) were compared with 12 healthy controls with no familiarity for migraine, and no significant age and sex differences between groups.

**Materials**: all subjects underwent the following examinations:

1. SIFI text consisting of presentation of flashes and beeps in different combinations to generate illusions of ‘fission’ (one flash with 2 beeps perceived as 2 flashes) or ‘fusion’ (2 flashes with one beep perceived as 1 flash); SIFI has been employed to study excitability of visual cortex as such illusory phenomena critically depend upon visual cortical excitability (more active visual cortex--> less illusions). 2. colorimetric test to explore dysfunction in color perception. 3. Multifocal electroretinogram(mfERG) to evaluate the functional contribution of retinal receptor in different retinal areas.

**Results**: according to previous report, MWA and MWoA patients showed significantly reduced SIFI of fission with respect to controls(p<.01). 8 MWA and 9 MWoA patients presented dysfunction of color perception. In these patients SIFI were significantly more reduced with respect to those showing no abnormality in chromatic perception(p<.05). No significant changes emerged in mfERG when comparing patients vs control; MWA vs MWoA, patients with and without chromatic perception dysfunctions.

**Discussion**: results confirmed increased excitability of visual cortex in MWA and MWoA patients [2]. Moreover, greater excitability levels are found in patients with chromatic perception abnormalities suggesting a potential pathophysiological relationship between peripheral and central visual dysfunction in these patients. However, results of nornal mfERG response seem to exclude direct dysfunctional involvement of visual receptors, making likely abnormalities at different retinal levels of the neural pathway (bipolar cells?)

**Conclusion**: peripheral and central abnormalities of visual can be found in migraine. They could be causally linked or follow to a common basic abnormality. Further study are needed to define the precise mechanism of such dysfunction together with their potential interrelationship and their role in the pathophysiology of the disease.

**References**

1. Bolognini N, Rossetti A, Casati C, Mancini F, Vallar G. Neuromodulation of multisensory perception: a tDCS study of the sound- induced flash illusion. Neuropsycologia 2011;49:231–237.

2. Brighina F, Bolognini N, Cosentino G, Maccora S, Paladino P, Baschi R, Vallar G, Fierro B. Visual cortex hyperexcitability in migraine in response to sound-induced flash illusions. Neurology. 2015 May 19;84(20):2057-61.

3. Shepherd AJ. Color vision but not visual attention is altered in migraine. Headache. 2006 Apr;46(4):611-21.

### O21 Neurosteroids in primary headaches

#### Andrea Negro^1^, Angela Koverech^1^, Claudia Cicione^1,2^, Marta Maestri^1^, Francesco Passariello^1^, Elisabetta Sabbatini^1^, Cristiano Maria De Marco^1^, Luana Lionetto^2^, Ferdinando Nicoletti^3,4^, Maurizio Simmaco^5^, Paolo Martelletti^1^

##### ^1^Department of Clinical and Molecular Medicine, Sapienza University of Rome, Italy;^2^Advanced Molecular Diagnostics Unit, IDI Istituto Dermopatico dell'Immacolata - IRCSS, Rome, Italy;^3^IRCCS Neuromed, Pozzilli, Italy;^4^Department of Physiology and Pharmacology, Sapienza University of Rome, Italy;^5^Advanced Molecular Diagnostics Unit, Sant'Andrea Hospital, Sapienza University of Rome, Italy

###### **Correspondence:**Andrea Negro (andrea.negro@uniroma1.it)

**Background**

Perturbation of neuronal excitability contributes to migraine. Neurosteroids, i.e., allopregnanolone (AP), epiallopregnanolone (EP), dehydroepiandrosterone (DHEA) and deydroepiandrosterone sulfate (DHEAS), rapidly modulate neuronal firing rate and might be involved in the pathogenesis of migraine [1]. We sought to determine the relationship between plasma levels of neurosteroids in patients with migraine and cluster headache.

**Materials and Methods**

Patients with episodic migraine (EM, n=19), chronic migraine and medication overuse headache (CM, n=51), and cluster headache (CH, n=18) were studied. Sex- and age-matched subjects were also evalueted (i.e., 31F and 16M). Patients were clinically characterized by using validated questionnaires (BDI, MIDAS, HIT-6, SAS, as appropriate). Menopausal status was also recorded. Plasma levels of AP, EAP, DHEA and DHEAS were measured using liquid chromatography-tandem mass spectrometry (LC-MS/MS) [2]. Whether sampling was concurrent with migraine/cluster attack was registered. Data have been statistically analysed using Student’s t-test and Spearman’s correlation. Data are presented as mean±SD.

**Results**

AP levels were consistently and significantly higher in EM (1.3±0.5 ng/mL) and CM (1.1±0.3 ng/mL) than in controls (0.6±0.3 ng/mL). In contrast, AP levels were significantly reduced in CH patients vs controls (0.3±0.1 vs 0.7±0.2 ng/mL, respectively; p<0.01). DHEA levels were significantly reduced in EM (2.9±1.5 ng/mL) and CM (1.6±1.1 ng/mL) compared to controls (5.1±3.8 ng/mL; p<0.05). The profiles of DHEAS and EAP across groups were inconsistent, being significantly reduced in CM, but not different from controls in EM and CH. In CM patients, the ratio AP/EAP was significantly higher than in controls (3.0±1.7 vs 1.8±1.2, respectively; p<0.01).

**Discussion**

Changes of circulating levels of neurosteroids are associated to the presence of migraine. The GABA_A_ receptor agonist, AP, and the NMDA receptor agonist, DHEA, were higher and lower than controls, respectively. This suggests an attempt to modulate neuronal hyperexcitability in migraine patients. Changes of neurosteroids concentrations in CH patients were significant only for AP, which may contribute to the more severe clinical features of CH vs CM and EM. For the first time, we provide evidence for a role of neurosteroids in the pathogenesis of migraine. Novel and more effective therapies may now be devised by targeting neurosteroid-related pathways.

**References**

1. Reddy DS, Estes WA. Clinical potential of neurosteroids for CNS disorders. Trends Pharmacol Sci. 2016; 37:543-561.

2. Lionetto L, De Andrés F, Capi M, Curto M, Sabato D, Simmaco M, Bossù P, Sacchinelli E et al. LC-MS/MS simultaneous analysis of allopregnanolone, epiallopregnanolone, pregnanolone, dehydroepiandrosterone and dehydroepiandrosterone 3-sulfate in human plasma. Bioanalayis 2017; 9:527-539.

### O22 Lasmiditan (200 mg and 100 mg) compared to placebo for acute treatment of migraine

#### Bernice Kuca^1^; Linda Wietecha^2^; Paul H Berg^2^; Sheena K Aurora^2^

##### ^1^CoLucid Pharmaceuticals, Inc., a wholly owned subsidiary of Eli Lilly and Company, Indianapolis, IN, USA;^2^Eli Lilly and Company, Indianapolis, IN, USA

###### **Correspondence:**Sheena K Aurora (sheena.aurora@lilly.com)

**Objective**

To compare efficacy on headache pain and the patient-centric measure of most bothersome symptom (MBS; nausea, phonophobia, or photophobia) at 2 hours post dose, time course of migraine response, rescue, and safety following treatment with lasmiditan 200 mg or 100 mg or placebo.

**Materials and methods**

In this randomized, double-blind, placebo-controlled study, subjects with at least moderate disability (Migraine Disability Assessment Score [MIDAS] ≥11) were randomized 1:1:1 to a first dose of lasmiditan treatment (200 mg or 100 mg) or placebo within 4 hours of onset of a migraine attack (moderate severity or worse and not improving). Subjects took a randomly assigned second dose of either their previously assigned lasmiditan dose or placebo for rescue or recurrence of migraine if needed (2 to 24 hours post initial dose); subjects randomized to placebo received placebo as the second dose. The primary measures were comparison of the proportions of subjects (modified intent-to-treat population [mITT]) in the lasmiditan 200 mg and placebo groups who, at 2 hours post first dose, were headache pain free and MBS free. Comparisons were made via logistic regression with terms for treatment group and background migraine preventative use. Lasmiditan 100 mg and placebo were also compared and safety parameters assessed.

**Results**

At baseline, 1545 subjects assigned to lasmiditan 200 mg (n=518), 100 mg (n=503), or placebo (n=524) had a mean MIDAS score of 31.3 and 82% had ≥1 cardiovascular (CV) risk factors (in addition to migraine). At 2 hours, greater proportions were: headache pain free with lasmiditan 200 mg (32.2%) and 100 mg (28.2%) than placebo (15.3%; *P*<.001); MBS free with lasmiditan 200 mg (40.7%) or 100 mg (40.9%) than placebo (29.5%; both *P*<.001); and, had headache relief with lasmiditan (59% each group) than placebo (42.2%; *P*<.001). In the lasmiditan 200 mg, 100 mg, and placebo groups, 29%, 36%, and 58%, respectively, took a second dose for rescue. The most frequently reported adverse events (AE) with lasmiditan were dizziness, fatigue, lethargy, nausea, paresthesia, and somnolence. No notable increase in CV AEs occurred in either lasmiditan group even among subjects with ≥1 CV risk factors (eg hypertension).

**Conclusions**

Lasmiditan treatment provided more patients freedom from and reduction of headache pain and MBS at 2 hours compared with placebo. This patient population with severe disability associated with migraine, tolerated lasmiditan treatment.

**Trial registration**

ClinicalTrials.gov Identifier NCT02439320.

### O23 Activation of peripheral and central trigeminovascular neurons by seizure: Implications to post-ictal headache

#### Agustin Melo-Carrillo^1,2^, Rami Burstein^1,2^

##### ^1^Department of Anesthesia, Critical Care & Pain Medicine, Beth Israel Deaconess Medical Center;^2^Harvard Medical School, Boston, Massachusetts, USA;^3^Center for Life Science, 3 Blackfan Circle, 02215. Boston. MA

###### **Correspondence:**Agustin Melo-Carrillo (amelo1@bidmc.harvard.edu)

**Objective**

To investigate the mechanism of post-ictal headache.

**Background**

Seizures affect 50 million people worldwide and are followed by headache in up to 50% of patients. These headaches are called Post-Ictal Headaches (PIH), they are commonly migraineous in nature, can last many hours, be very painful and since they strikes repetitively, they drive patients to endure a significant hardship. Given that seizures occur in the cortex, we hypothesized that PIH are intracranial at origin and accordingly, aimed in this study to determine whether and how seizures (both generalized and focal) activate peripheral and central neurons of the meningeal sensory pathway.

**Methods**

Single-unit electrophysiological techniques were used to study the physiological properties and response profile of C- and Aδ meningeal nociceptors and high-threshold (HT) and wide-dynamic range (WDR) neurons in the spinal trigeminal nucleus – in response to occurrence of seizure underneath their dural receptive fields. Cortical electrodes were used to trace the magnitude, extent and progression of epileptiform seizure.

**Results**

The induction of seizure triggered prolonged activation in both meningeal nociceptors and central trigeminovascular neurons. In the ganglion, activity began to increase minutes after the seizure reached their receptive fields and remained elevated for as long the seizure activity continued. In the medullary dorsal horn, neurons exhibited a biphasic response following seizure onset that consisted of an initial inhibition of the spontaneous activity followed by a gradual increase in activity that remained above baseline for at least 2 hours after seizure onset. In separate experiments, we created a focal seizure that was restricted to either the parietal or occipital cortex. Induction of focal seizure in the parietal cortex did not produce any changes in neuronal activity, but induction of focal seizure in the occipital cortex produced the same neuronal changes that we observed with the generalized seizure.

**Conclusions**

This study provides evidence for activation of the trigeminovascular pathway by generalized and focal seizures. The importance of this study is that it uses a model of abnormal cortical activity to which there are ample of evidence in human subjects suffering epilepsy (and PIH), and thus, it bypass the bitter debate over the validity of studying the headache phase of migraine using the controversial cortical spreading depression model – to which evidence for actual occurrence in humans are scarce.

### O24 Association between subclinical hypothyroidism and migraine

#### Innocenzo Rainero^1,2*^, Flora Govone^1^, Francesca Garino^3^, Costanza Vicentini^1^, Alessandro Vacca^1^, Annalisa Gai^1^, Salvatore Gentile^2^, Federico Ragazzoni^3^, Lorenzo Pinessi^2^, Maria Teresa Giordana^1,2^, Paolo Limone^2^, Elisa Rubino^1^

##### ^1^Neurology I – Headache Center, Department of Neuroscience “Rita Levi Montalcini”, University of Torino, 10126 Torino, Italy;^2^Department of Neuroscience and Mental Health, A.O.U. Città della Salute e della Scienza, Torino, 10126 Torino, Italy;^3^Endocrinology, Diabetes and Metabolic Disease Unit, A.O. Ordine Mauriziano di Torino, 10128 Torino, Italy

###### **Correspondence:** Innocenzo Rainero (innocenzo.rainero@unito.it)

**Background**

In recent years, an increased attention has been devoted to the potential association between migraine and thyroid function, with conflicting results. A few studies found that migraine may be associated with an increased risk for the development of both overt and subclinical hypothyroidism (SH). On the contrary, the risk of developing migraine in patients with hypothyroidism has been scarcely investigated. Therefore, the main purpose of our study was to investigate the association between subclinical hypothyroidism and migraine in adults, using a case-control strategy.

**Materials and methods**

151 consecutive patients with SH (24 men and 127 women, mean age 48.36± 15.86 years) and 150 controls (37 men and 113 women, mean age 50.86± 9.19 years) were recruited for the study. In all subjects, migraine characteristics were collected through a direct face-to-face interview, using a structured questionnaire. Moreover, clinical characteristics and thyroid parameters (TSH, fT3, fT4, anti-thyroglobulin and anti-peroxidase antibodies) were compared between SH patients with comorbid migraine and SH patients without migraine.

**Results**

The prevalence of lifetime migraine was significantly higher in SH patients in comparison with controls (46% vs 13%, p< 0.001; OR 5.80; 95% CI= 3.35-10.34). Both migraine without aura and migraine with aura were significantly higher in SH patients than controls (38% vs. 9%, p< 0.001; and 8% vs. 4%, p= 0.01, respectively). Thyroid hormones and antibodies levels were not different between SH patients with migraine and those SH patients without migraine. Finally, a higher comorbidity for autoimmune diseases was found in SH patients with migraine respect to SH patients without migraine.

**Conclusions**

Our data suggest that migraine is significantly more frequent in patients with subclinical hypothyroidism respect to controls. Additional studies are needed in order to corroborate this association and to explore the underlying biological mechanisms.

## SISC ORAL PRESENTATIONS

### O25 Migrainous infarction in a patient with Sporadic Hemiplegic Migraine and Cystic Fibrosis

#### Valentina Mancini^1^, Giulio Mastria^1^,Massimiliano Toscano^1^, Federica Turchi^1^, Barbara Petolicchio, Alessandro Viganò^1^, Vittorio Di Piero^1^

##### ^1^Neurology and Psychiatry, Sapienza - University of Rome, Rome, Italy. Rome, Rome, Italy

###### **Correspondence:**Alessandro Viganò (alessandro.vigano@uniroma1.it)

**Background**

Cystic fibrosis (CF) is autosomal recessive genetic disease caused by mutation in the transmembrane conductance regulator (CFTR) that is involved in Cl^-^ ions transport. Coexistence of cystic fibrosis and sporadic hemiplegic migraine (SHM) has been reported in only two cases in literature so far [1]. Their co-occurrence may be due to more than a simple chance. CFTR is widely expressed in the brain and seems to be important in regulating other channels, suggesting that some shared genetic channel mutation might cause the two diseases at the same time.

**Case report**

A 32-y.o. caucasian male was diagnosed with CF at birth. He presented CFTR heterozygosis with a typical CFTR delta F508 mutation in one allele and an unknown mutation on the other allele. He also had patent foramen ovale that was surgically closed when he was 26-y.o.

The patient reported occasional cough bouts, triggering hemiplegic migraine attacks

In March 2017 a such cough bout, lasting 48 hours, led to a severe hemoptysis and followed by a 15-minutes long loss of consciousness. The patient was transported to emergency rooms (ER) and intubated. An arterial-blood gas test showed severe hypoxia with hypercapnia. The patient recovered in few days. A brain MRI+MRA, performed in ER showed diffuse DWI alterations, affecting left frontal-parietal-occipital areas, which later resolved, except for a persistent small damage in the frontal region. Both large vessel diseases and embolic sources as well were excluded by carotid duplex sonography, transcranial doppler examination and transthoracic echocardiography.

In April, he was visited in our Headache Clinic. The neurological examination showed a mild right hemiparesis with asymmetrical increased tendon reflexes and upward plantar reflex on the right.

**Conclusions**

The ischemic damage, presented by the patient, suggests a migrainous infarction, since it followed the migraine aura symtpoms and occurred in the brain regions implied in HM. Then, it may be argued that migrainous infarction was caused by the development of CSD in a brain tissue, suffering from hypoxia due to cough bout and hemoptysis.

In fact, cerebral infarction, with prompt expansion of the core infarct, following mild hypoxia, is associated to mutation in CACNA1 gene in both mice and humans, possibly related to neural excitability and the following vasoconstrictive vascular coupling in the penumbra [2-3].

In conclusion, in CF patients there is the chance of SHM comorbidity, due to a genetic overlap with a higher risk of developing migrainous infarction.

**Consent for publication:** The authors declare that written informed consent was obtained for publication.

**References**

1) Rao DS, Infeld MD, Stern RC, Chelimsky TC Cough-induced hemiplegic migraine with impaired consciousness in cystic fibrosis. Pediatr Pulmonol. 2006 Feb;41(2):171-6

2) Eikermann-Haerter K, Lee JH, Yuzawa I, et al. Migraine mutations increase stroke vulnerability by facilitating ischemic depolarizations. Circulation 2012; 125(2): 335–345.

3) Rainer Malik, Bendik Winsvold, Eva Auffenberg, Martin Dichgans and Tobias Freilinger, The migraine–stroke connection: A genetic perspective, Cephalalgia 2015, Vol 36, Issue 7, pp. 658 – 668.

### O26 Features of chronic primary headaches (CPH) in children and adolescents referred to two third level headache centers

#### Laura Papetti^1^, Irene Salfa^1^, Beatrice Bartoli^2^, Cristiano Termine^2^, Barbara Battan^3^, Massimiliano Valeriani^1^

##### ^1^Neuroscience, Bambino Gesù Children Hospital, Rome;^2^Neuropsychiatric, Insubria University, Varese;^3^Neuroscience, Bambino Gesù Children Hopsital, Rome, Italy

###### **Correspondence:** Massimiliano Valeriani

**Objectives:** Chronic migraine (CM), Chronic tension-type headache (CTTH) and new daily persistent headache (NDPH) are the main forms of CPH reported in the ICHD-III beta version. Medication-overuse headache (MOH) is classified among secondary headache but it generally affects patients with a pre-existing primary headache. CPH have been well described in adults and their diagnostic criteria were designed based on the clinical characteristics in the adult population. However, CPH are not a rare condition in children and adolescence with negative impact on their quality of life.

Our aim was to investigate the clinical features of CPH in a cohort of pediatric patients.

**Methods:** We retrospectively reviewed the charts of patients attending the Headache Centre of Bambino Gesú Children and Insubria University Hospital. The ICHD-III criteria were used for diagnosis. Statistical analysis was conducted by SPPS version 22.0 and χ2 test was used to study possible correlations between: - CPH and population features (age and sex); - CPH and headache qualitative features; - CPH and risk of MOH; -CPH and response to prophylactic therapies.

**Results:** We included 377 patients with CPH (66.4% female, 33.6% male, age between 0 and 18 years). The most frequent CPH type was CM (73.5%), followed by CCTH (13.5%) and NDPH (13%). MOH was detected in 10.9% of total patients. CPH are less frequent under 6 years of age (0.8%; p <0.05); significant greater frequency in females than in males was found in the age group between 0-6 years (23/31 F, 8/31 M) and between 15-18 years (41/51 F, 10/51 M) (p<0.05). No correlations between age/sex and different CPH types were detected. We found a more frequent incidence of vegetative symptoms (photo/phonophobia and vertigo) in female sex (p< 0.05). Nausea and vertigo are the two most frequent vegetative symptoms under 10 years of age (p<0.05) while photo/phonophobia are more frequently in patients older than 15 years (p<0.05). Possible development of MOH has been found correlated with CM types (p<0.05) and age above 15 years (p<0.05).

Our results show that 272 (72.1%) out of 377 CPH patients received a prophylactic therapy. Among them, 190 patients received amitriptyline, 29 patients topiramate, 15 patients L-5 hydroxytryptophan, and 8 patients flunarizine. Thirty patient performed two or more drugs. Positive response to therapy (reduction of attacks by at least 50% in a month) was detected in 54% of patients, while no outcome data were obtained from 29.4% of cases. Amitriptyline and topiramate had the highest efficacy (p<0.05). We found that 59.2% of patients who received amitriptyline showed significant reduction in the attack frequency, while 48.4% patients receiving topiramate improved their headache attack frequency (p>0.05). However, for both drugs more than 30% of patients did not have follow up data.

**Conclusion:** Our results showed that CPH presented a correlation with patients’ age and sex. No significant differences were found between CPH types and population/pain features. Development of MOH was related with CM onset and adolescent age. Amitriptyline and topiramate had the best effectiveness. However, it is to be underlined that follow up data could not be issued from a moderate percentage of patients. It will be useful in the future to reduce the number of missing patients by improving patients’compliance and promoting the concept of migraine as a disease that can cause relevant disability.

**Disclosure of interest**

None Declared

### O27 Clinical features, risk factors and comorbidity of chronic migraine. An Italian nationwide survey among headache centres

#### Franco Granella

##### Neurosciences Unit, Department of Medicine and Surgery, University of Parma, Parma, Italy

**Background**

Chronic migraine (CM) is a disabling neurologic condition that affects 2-3% of the general population, and is the first reason of consultation in many tertiary headache centres. Nevertheless, comprehensive descriptions of CM clinical features, risk factors and comorbidity in large case series are very scarce. Aim of this study is to depict the characteristics of CM in a large cohort of patients referred to several tertiary headache centres throughout Italy.

**Patients and methods**

Patients with the diagnosis of CM according to ICHD-3 beta, consecutively referred to 16 major Italian headache centres during the period 1^st^ October 2016 – 31^th^ March 2017 entered the study, having expressed an informed consent. An especially prepared web-based electronic medical record was filled in with anonymized patients data.

**Results**

Eight hundred and thirty-eight patients (female 86.3%; mean age 48.5±13.08 years) were included. CM mean duration was 10.7 years; mean frequency of headache days was 22.7±6.11/month. Mean headache duration was 10.5±6.81 hours; mean headache severity on a 1-10 scale was 7.7±1.56. ICHD-3 beta CM diagnostic criterion C was satisfied by having ≥8 days per month with migraine without aura by the overwhelming majority of patients (95.5%), while a not negligible percentage had migraine with aura (9.1%) or headache relieved by a triptan (9.7%). Criteria for medication-overuse headache were fulfilled in 71.4% of the cases. Most overused drugs were simple analgesics (40.6%), followed by triptans (35.1%), and combination analgesics (15.8%). Beyond medication overuse, the most frequent risk factors for CM were: high baseline headache frequency (39.1%), anxiety (30.3%), sleep disorders (26.0%), depression (22.2%), cutaneous allodynia (14.1%), life events (11.5%), excessive caffeine intake (10.4%), sleep bruxism (9.1%), panic disorder (7.2%), and obesity (6.2%). Most represented comorbid disorders were: anxiety (32.3%), sleep disorders (32.2%), depression (25.4%), hypertension (20.5%), irritable bowel (8.4%), and fibromyalgia (6.6%). CM was highly disabling in our population: mean MIDAS score was 66.6 and mean HIT-6 score 63.7. Mean monthly number of symptomatic drugs was 28.8 (range 0-270). Refractory chronic migraine (no MOH, unsuccessful use of at least 3 preventive drugs) was present in 11.3% of the patients.

**Conclusions**

The study of a large case series of patients with CM allows a precise picture of this debilitating condition, of its main clinical features, transformation to CM risk factors and comorbid disorders.

**Acnowledgements**

This study was carried out on behalf of the Independent Research section of Società Italiana per lo Studio delle Cefalee (SISC).

### O28 Prolonged migraine aura: prevalence and comparison with “normal” auras

#### Michele Viana^1^, Grazia Sances^1^, Mattias Linde^2^, Giuseppe Nappi^1^, Peter J. Goadsby^3^, Cristina Tassorelli^1, 4^

##### ^1^Headache Science Center, C. Mondino National Neurological Institute, Pavia, Italy;^2^Department of Neuroscience, Norwegian University of Science and Technology, Trondheim, Norway;^3^Headache Group – NIHR-Wellcome Trust Clinical Research Facility, King’s College London, London, United Kingdom;^4^Dept. of Brain and Behavioral Sciences, University of Pavia, Pavia, Italy

###### **Correspondence:**Michele Viana (michele.viana@ymail.com)

**Background:** There are no prospective studies on prolonged auras (PAs). i.e. an aura that includes at least one symptom lasting for >1hr.

**Objectives:** We aimed to evaluate the prevalence of PAs and compare their phenotype with NON-PAs in a prospective diary-aided study.

**Methods:** We recruited 224 consecutive patients affected by migraine aura attending the Headache Centers of Pavia and Trondheim. Patients were asked to report on an *ad hoc* diary, during three consecutive migraine with aura attacks, quality of each aura symptom (AS), headache features and to insert the time of onset/end of each AS and of the painful phase and describe the features of headache.

**Results:** 72 patients completed the diaries during three consecutive auras for a cumulative number of 216 auras recorded. Out of 216 auras, 38 (17%) were PAs. Out of 72 patients, 19 (26%) have at least one PA. When comparing PAs with non-PAs (n=178) with respect to 20 features collected, PAs were characterized by a higher total number of symptoms (p<0.001), a higher frequency of sensory symptoms (p<0.001) and a higher frequency of dysphasic symptoms (p<0.001). No other differences were found.

We performed the same analysis comparing auras including at least one symptom lasting for >2 hrs (PA>2, n=23) with the remaining ones (n=193) and auras including at least one symptom lasting for >4 hrs (PA>4, n=14) with the remaining ones (n=202). In the first comparison, the only differences were a higher frequency of sensory symptoms and a higher number of AS in PA>2 (p=0.001 and p=0.005, respectively) and in the second comparison the only difference was a higher number of AS in PA>4 (p=0.043).

**Conclusion:** PA is quite common (17% of all auras, occurring at least once in 26% of patients) and phenotypically differs from the other auras only for a higher number of non-visual symptoms (non-VSs). This latter finding is not surprising if we consider that an AS with a longer duration is likely related to a cortical spreading depression (CSD) that proceeds along a longer path on the respective brain area. Such CSD therefore is more likely to involve other adjacent brain areas, thus conferring a higher number of non-VSs to PA. The substantial phenotypical similarities between PAs and the other auras is maintained also when we increase the limit of duration to 2 and/or 4 hrs. This finding should lead to a discussion about the term “prolonged aura” and how long its duration should be.

### O29 Microvascular vasospasm of cerebral cortex in “prolonged” aura migraine

#### Stefano Viola^1*^, Paolo Viola^2^, Maria. P. Buongarzone^1^, Luisa Fiorelli^1^, Mafalda Cipulli^1^, Pasqualino Litterio^1^

##### ^1^Department of Neurology, Headache Center, via C. De Lellis 66054 Vasto (CH), Italy;^2^Emergency Medical Service, Atessa, Lama dei Peligni (CH), Italy

###### **Correspondence:**Paolo Viola

**Background**: Many studies support the presence of endothelial dysfunction in migraineurs. In a recent work, the authors have found a coronary microvascular endothelial dysfunction (CMED) in migraine patients compared with control group suggesting that there would be a common pathophysiological pathway of impaired coronary and cerebral endothelial function in migraine. CMED may lead to a coronary microvascular vasospasm.

**Aim:** The aim was to verify the presence of a microvascular vasospasm of cerebral cortex in “prolonged” aura migraine.

**Methods:** We studied the microcirculation of cerebral cortex by Near Infrared Spectroscopy

(NIRS) and cerebral macrocirculation by transcranial Doppler (TCD) in 8 subjects (3 M and 5 F, age range 21-41 years) during spontaneous “prolonged” migraine aura in according to ICHD criteria and compared the results with the headache-free periods. Duration of aura symptoms: between 2 and 8 hours.

**Results:** During aura NIRS showed a significant decrease of the Arterial Pulse Wave of Cerebral Microcirculation amplitude (-33 % ± 5.7), p<0.001 contralateral to the symptoms of aura compared with the headache-free periods; TCD showed a significant increase of Pulsatility Index (+36.5 % ± 6.5), p<0.001 and a significant decrease of the diastolic velocity in the posterior and middle cerebral artery contralateral to the symptoms of aura compared with the headache-free periods.

**Conclusions:** In conclusion during “prolonged” migraine aura we find cortical areas of microvascular vasospasm corresponding to the topography of aura symptoms and suggesting the presence of a microvascular endothelial dysfunction.

**Consent for publication:**

Written informed consent to publication was obtained from the patient(s).

### O30 A nutraceutical formulation of Magnesium+Tanacetum parthenum+Griffonia vs Amitriptyline in Migraine prophylaxis

#### Maria Pia Prudenzano, Alessandro Introna, Maria Tappatà, Simona Lamberti, Maria Elena Roca, Isabella Laura Simone, Maria Trojano

##### Headache Centre. Department of Basic Medical Sciences, Neuroscience and Sense Organs. University of Bari

###### **Correspondence:**Maria Pia Prudenzano (centrocefalee.neurologia@uniba.it)

**Background**: A wide range of medications is now available for migraine prophylaxis but some patients don’t show a significant improvement also with the best tailored drug and some other patients discontinue therapy because of side effects. The interest of clinicians and patients toward nutraceutical medicine is expanding worldwide but limited evidence is to date available in favour of and against their use in migraine prophylaxis [1,2].

**Objectives**: To compare efficacy and tolerability of a nutraceutical formulation containing Magnesium 185 mg + *Tanacetum parthenium 150 mg* + Griffonia 100 mg/die (MTG) vs Amitriptyline 25 mg/die (AMI) for 3 months, as prophylactic migraine therapy in a sample of adult outpatients.

**Patients and Methods**: This is a retrospective study conducted by reviewing the medical records of all patients consecutively referred to the Headache Centre from January 2017 to June 2017. 62 patients (52 females and 10 males), who received the diagnosis of migraine with or without aura and were submitted to a 3 months prophylactic therapy with AMI (33 cases) or with MTG (29 cases) were recruited. Primary efficacy parameters were: attack frequency and aura frequency reduction. Secondary efficacy parameters were pain intensity, pain duration and aura duration reduction. Tolerability was evaluated by means of reported side effects.

**Results:** A significant reduction of headache frequency, pain intensity, pain duration, aura frequency and aura duration was found in the whole sample after a 3 months prophylactic therapy. No difference was found between MTG group and AMI group in attack frequency reduction (4,90+6,37 vs 8,30±8,09, p=0,21) and in aura frequency reduction (3,00+4,84 vs 2,34+2,89, p=0,72). Six patients in AMI group reported side effects (weight gain 3 cases, somnolence 1 case, weight gain and somnolence in 2 cases). One patient in MTG group reported both hands paresthesias.

**Conclusions**: The results of this study suggest that the nutraceutical formulation containing Magnesium 185 mg+ *Tanacetum parthenium* 150 mg+ Griffonia 100 mg, could be a valid therapeutic option with lower occurrence of side effects than Amitriptyline. The strength of evidence of this study is low because of both the retrospective design and the little sample. Double-blind randomized larger studies are needed to correctly estimate the impact of the placebo effect in this promising therapy.

**References**

1. Sarchielli P, Granella F, Prudenzano MP, Pini LA, Guidetti V, Bono G, Pinessi L, Alessandri M, Antonaci F, Fanciullacci M, Ferrari A, Guazzelli M, Nappi G, Sances G, Sandrini G, Savi L, Tassorelli C, Zanchin G. Italian guidelines for primary headaches: 2012 revised version. J Headache Pain. 2012 May; 13 Suppl 2:S31-70.

2. D'Onofrio F, Raimo S, Spitaleri D, Casucci G, Bussone G. Usefulness of nutraceuticals in migraine prophylaxis. Neurol Sci. 2017 May;38(Suppl 1):117-120.

### O31 Comorbidity profiles of migraine sufferers in headache center

#### Alessandro Panconesi, Maria L Bartolozzi, Leonello Guidi

##### Headache Center, Department of Neurology, Health Authority 11, Empoli, Italy

###### **Correspondence:**Alessandro Panconesi (alessandro.panconesi@uslcentro.toscana.it)

**Background**

Some epidemiological studies report many comorbidities in migraine, mostly identified through questionnaires and based on self-reported diagnosis. However, the prevalence of comorbidities should be better performed by a clinical evaluation.

**Materials and methods**

We have evaluated prospectively the prevalence of some comorbidities in the first 1000 migraine patients (aged 15-65 years), afferent to Headache Center in the 2011-2013 years and resident in the district of Empoli Health Authority, classified with a diagnosis of migraine without aura (MO), migraine with aura (MA), and chronic migraine (CM) that also includes patients with medication overuse headache who had episodic MO before, according to the revised ICHD-2R classification. The specialist visits were performed by a single physician (A.P.). In addition to a detailed semistructured face to face interview, health information was researched in the database of specialist visits, hospital recovery or emergency department.

**Results**

Our clinical survey consisted of 1000 patients aged 15-64 years (mean age 39.5), 780 females (mean age 39.9) and 220 males (mean age 38.2). This population included 866 patients with episodic migraine (EM) (666 ♀ and 200 ♂, mean age 38.2) and 134 with CM (114 ♀ and 20 ♂, mean age 48.0). Out of 866 patients with EM, 767 had MO (mean age 38.4) and 99 had MA (mean age 36.7).

The prevalence of treated chronic conditions (CC) was: depression 5.6 %, hypertension, 9.3 %, diabetes 1.2 %, asthma 3.3 %, hypothyroidism 6.4 %, hypercholesterolemia 0.5 %.

Out of 56 patients assuming antidepressants only 4 assumed amitriptyline. Out of 93 patients treated for hypertension, 42 used beta-blockers (18 atenolol, 13 nebivolol, 6 propranolol, 3 bisoprolol, 1 metoprolol, 1 carvedilol).

Patients who assumed thyroid hormones were 64: they referred thyroiditis (28), thyroidectomy (13), other (primitive, post-hyperthyroidism, etc) hypothyroidisms (16), goitre in euthyroidism (7). Patients with asthma were 65: 33 in current treatment and 32 in the past. Migraine patients suffering of allergies were 198: 132 rhinitis, 42 drugs allergy and 19 food allergy.

Other somatic CC were: gallstones (5.4%), kidney stones (5.7%), inflammatory bowel disease (0.5%) including Crohn disease (3 patients) and ulcerative colitis (2 patients), celiac disease (0.5%, all females), endometriosis (1.0%), psoriasis (0.7%), cardiac diseases (1.0%), rheumatic/autoimmune diseases (0.7%) including lupus (0.2%), Behcet disease, mixed connective disease, rheumatoid arthritis, scleroderma, and Sjogren syndrome (0.1%).Vitiligo (0.2%), endometrial/breast cancer (0.5%), meningioma (0.2%), hyperthyroidism (0.3%), restless legs syndrome (0.3%), fibromyalgia (0.2%), were also detected. Other CC whit a prevalence of 0.1% are not reported.

Hypothyroidism was more frequent in females (OR 0.163, p < 0.001), and hypertension in CM compared to EM (OR 0.307, p < 0.001) but EM patients were 10 years younger than CM patients (Table 1).

**Conclusions**

The percentages of treated CC in migraine patients in our survey, in particular those involved in cardiovascular risk, were not higher than those estimated through the analysis of prescription rate of marker drugs in the general population in the same district [1,2]. In this comparison, the higher prevalence of hypothyroidism was not statistically significant when migraine patients were considered by gender (♀ p = 0.067, ♂ p = 0.38), while epilepsy, diabetes, hypercholesterolemia and asthma, on the contrary, were less frequently reported (p<0.001).

Our data are in substantial agreement to a large general practitioner’s database [see 2].

**Table 1 (abstract O31).** Percentage of treated diseases and lifetime allergies in migraine patients

	All subjects	♂	♀	EM	CM
Depression	5.6	3.1	6.2	5.4	6.7
Hypertension	9.3	8.1	9.6	7.5	20.8^*^
Diabetes	1.2	1.3	1.1	1.0	2.2
Asthma	3.3	3.2	3.3	3.0	5.7
Hypothyroidism	6.4	1.3	7.8 ^*^	6.3	6.7
Allergy	19.8	21.8	19.2	19.8	19.4

^*^p < 0.001

**References**

1) Panconesi A, Pavone E, Pagliai C, Coletta D, Mennuti N, Bartolozzi ML, Benemei S, Guidi L. Evaluation of migraine comorbidities through clinical and pharmaceutical data. J Headache Pain 2013; 14(Suppl):13-14.

2) Panconesi A, Bartolozzi ML, Guidi L. Migraineurs: seriously ill or basically healthy? J Headache Pain 2015; 16(Suppl 1):28.

### O32 Nutraceuticals and herbs in the treatment of pediatric primary headaches

#### Elena Piretti1, Irene Toldo1, Maria Paola Rossaro1, Stefano Sartori1, Michela Gatta2, Margherita Nosadini1, Pier Antonio Battistella1

##### ^1^Juvenile Headache Centre, Department of Woman's and Child’s Health, University Hospital of Padua, Italy; ^2^Socio-sanitarydistrict, “Struttura Complessa Infanzia Adolescenza Famiglia” (SCIAF), ULSS 6 Euganea, Padua, Italy

###### **Correspondence:** Antonio Battistella (pierantonio.battistella@unipd.it)

**Background**

There is an increasing use of Alternative and Complementary Medicine (CAM) for prophylaxis of pediatric primary headaches [1,2,3,4].

**Materials and methods**

We carried out a search for clinical trials in PubMed of patients aged 0-18 years with primary headaches treated with nutraceuticals or phytotherapeutic compounds.

**Results**

A) *Nutraceuticals*

The following evidences are available in the literature:

- Magnesium: significant reduction in attacks, consumption of analgesics and disability in cases with Migraine (M) [5] and Tension-Type Headache (TTH) [6]
- Riboflavin (vitamin b2): higher efficacy than placebo in the treatment of TTH [7]. In M: effective compared to baseline [8] but not superior to placebo [7, 9]
- Coenzyme Q10: in CoQ10-deficient patients, CoQ10 supplementation obtained a reduction of attacks’ frequency and disability degree [10]. A trial showed no efficacy versus placebo [11]
- Alpha lipoic acid: no study available
- Melatonin: significant reduction in frequency and duration of M or chronic TTH attacks [12]. In another study: reduction of frequency, intensity, duration of M attacks and PedMIDAS score [13]
- 5-hydroxytryptophan: no significant differences vs placebo in M (Santucci M,1986).

B) *Phytotherapeutic products*

The following evidences are available in the literature:

- Petasites hibridus: 50% reduction of attacks in 77% of cases with M in an open-label multicentre study [14]. Long term effects better than placebo in another study [15]
- Tanacetum parthenium: no study available
- Ginkgo biloba combined to Coenzyme Q10/Vitamin B2/Magnesium: reduction of the M frequency and the use of symptomatic medication in a open-label study [16]; reduction of the M frequency in another open-label study[17]; reductions of M frequency, duration, intensity, PedMIDAS score, behavioural reactions to M were significantly greater in the Ginkgolide group than in the group received L-tryptophan/5-hydroxytryptophan (from Griffonia simplicifolia)/vitamin PP and B6[18].

C) *Italian experience in specialized juvenile headache Centers*

In a multicentre study of 637 young M and TTH patients, supplements and melatonin were used respectively by 205 (32%) and 61 (10%) patients with a good/excellent efficacy in 68% and 75% of cases respectively, without differences between the two headache types[19].

**Conclusions**

Nutraceuticals and phytotherapeutics showed some positive effects on M and TTH in pediatric patients in open label studies and in some controlled studies with placebo. Further studies are needed to better address therapeutic choices in primary pediatric headaches.

**References**

1. Ledda M G, Porcu L, Cianchetti C. Complementary and alternative therapies in primary headaches. Giornale di Neuropsichiatria dell’Età Evolutiva. 2012; Volume 32. Numero 1:65-72

2. Bethell C, Kemper KJ, Gombojav N, Koch Thomas K. Complementary and Conventional Medicine Use Among Youth With Recurrent Headaches. Pediatrics. 2013;132(5):1173–83.

3. Dalla Libera D, Colombo B, Pavan G, Comi G. Complementary and alternative medicine (CAM) use in an Italian cohort of pediatric headache patients: the tip of the iceberg. Neurol Sci. 2014;35(Suppl 1):S145–8.

4. Orr SL, Venkateswaran S. Nutraceuticals in the prophylaxis of pediatric migraine: Evidence- based review and recommendations. Cephalalgia. 2014;34(8):568–83.

5. Wang F, Van DenEeden SK, Ackerson LM, Salk SE, Reince RH, Elin RJ. Oral Magnesium Oxide Prophylaxis of Frequent Migrainous Headache in Children: A Randomized, Double-Blind, Placebo-Controlled Trial. Headache. 2003;43:601–10.

6. Grazzi L, Andrasik F, Usai S, Bussone G. Magnesium as a preventive treatment for paediatric episodic tension-type headache: results at 1-year follow-up. Neurol Sci. 2007;28:148–50.

7. Bruijn J, Duivenvoorden H, Passchier J, Locher H, Dijkstra N, Arts W-F. Medium-dose riboflavin as a prophylactic agent in children with migraine: A preliminary placebo-controlled, randomised, double-blind, cross-over trial. Cephalalgia. 2010;30(12):1426–34.

8. Condò M, Posar A, Arbizzani A, Parmeggiani A. Riboflavin prophylaxis in pediatric and adolescent migraine. J Headache Pain. 2009;10:361–5.

9. MacLennan SC, Wade FM, Forrest KML, Ratanayake PD, Fagan E, Antony J. High-dose riboflavin for migraine prophylaxis in children: a double-blind, randomized, placebo-controlled trial. J Child Neurol. 2008;23(11):1300–4.

10. Hershey AD, Powers SW, Vockell A-LB, Lecates SL, Ellinor PL, Segers A, et al. Coenzyme Q10 deficiency and response to supplementation in pediatric and adolescent migraine. Headache. 2007;47(1):73–80.

11. Slater SK, Nelson TD, Kabbouche MA, LeCates SL, Horn P, Segers A, et al. A randomized, double-blinded, placebo-controlled, crossover, add-on study of CoEnzyme Q10 in the prevention of pediatric and adolescent migraine. Cephalalgia. 2011;31(8):897–905.

12. Miano S, Parisi P, Pelliccia A, Luchetti A, Paolino MC, Villa MP. Melatonin to prevent migraine or tension-type headache in children. Neurol Sci. 2008;29:285–7.

13. Fallah R, Shoroki FF, Ferdosian F. Safety and Efficacy of Melatonin in Pediatric Migraine Prophylaxis. Curr Drug Saf. 2015; 10(2): 132-5

14. Pothmann R, Danesch U. Migraine Prevention in Children and Adolescents: Results of an Open Study With a Special Butterbur Root Extract. Headache. 2005;45:196–203.

15. Oelkers-Ax R, Leins A, Parzer P, Hillecke T, Bolay H V, Fischer J, et al. Butterbur root extract and music therapy in the prevention of childhood migraine: An explorative study. Eur J Pain. 2008;12:301–13.

16. Usai S, Grazzi L, Bussone G. Gingkolide B as migraine preventive treatment in young age: results at 1-year follow-up. Neurol Sci. 2011;32(suppl 1):S197–9.

17. Esposito M, Carotenuto M. Ginkgolide B complex efficacy for brief prophylaxis of migraine in school-aged children: An open-label study. Neurol Sci. 2011;32:79–81.

18. Esposito M, Ruberto M, Pascotto A, Carotenuto M. Nutraceutical preparations in childhood migraine prophylaxis: Effects on headache outcomes including disability and behaviour. Neurol Sci. 2012;33:1365–8.

19. Toldo I, Rattin M, Perissinotto E, De Carlo D, Bolzonella B, Nosadini M, et al. Survey on treatments for primary headaches in 13 specialized juvenile Headache Centers: The first multicenter Italian study. Eur J Paediatr Neurol. 2017;1–15.

